# Transcriptomics and Neuropeptidomics of the Cockroach *Rhyparobia maderae* to Characterize the Neuropeptide Landscape of the Cockroach’s Circadian Clock

**DOI:** 10.1021/acs.jproteome.4c01069

**Published:** 2025-10-27

**Authors:** Susanne Neupert, Sohail H. Shoaib, Julia Schendzielorz, Huleg Zolmon, Lars Hering, Monika Stengl

**Affiliations:** † 9178 University of Kassel, Institute of Biology, Department of Animal Physiology and Neuroethology, Heinrich-Plett-Str. 40, Kassel 34132, Germany; ‡ University of Kassel, Graduate school: multiscale Clocks, Heinrich-Plett-Str. 40, Kassel 34132, Germany; § 9178 University of Kassel, Institute of Biology, Department of Zoology, Heinrich-Plett-Str. 40, Kassel 34132, Germany

**Keywords:** transcriptomics, neuropeptidomics, circadian clock, mass spectrometry, insect, Rhyparobia maderae

## Abstract

The circadian clock of *Rhyparobia maderae* is the accessory medulla (AME) in the optic lobes of the brain. Controlled by compound ocular photoreceptors, the cockroach clock orchestrates circadian rhythms in physiology and behavior synchronized with environmental light–dark cycles. Approximately 240 neurons, rich in partially colocalized neuropeptides, innervate the AME. Since the identity and function of most of these neuropeptides are unknown in the cockroach, the transcriptome of the Madeira cockroach was prepared, revealing 68 neuropeptides, neuropeptide-like and protein hormone genes. To identify the neuropeptidome, a combination of analyses of brain/retrocerebral complex extracts by Q-Exactive Orbitrap mass spectrometry (MS) and direct tissue profiling of different parts of the nervous system by matrix-assisted laser desorption/ionization time-of-flight (MALDI-TOF) MS was used. The resulting data set revealed 192 mature neuropeptides, with 155 identified for the first time and 57 precursor peptides. The AME neuropeptidomic profile revealed 166 mature neuropeptides with potential bioactive circadian clock functions. The precision of our MS data was validated by immunostaining, as illustrated by an example of immunoreactive patterns for proctolin in AME neurons. This peptidergic data set serves as the foundation for future research on neuropeptide-based functional studies that regulate the timing of disparate neuronal circuits.

## Graphical abstract

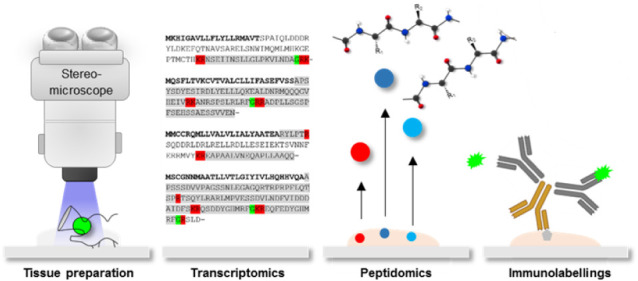

## Introduction

The Madeira cockroach, *Rhyparobia maderae* (syn. *Leucophaea maderae*), is an established model system of chronobiology, particularly suited to analysis at the cellular level.^1–4^ The circadian clock of the Madeira cockroach exhibits more structural and functional similarities to the mammalian circadian pacemaker, the *nucleus suprachiasmaticus* (SCN) than to the circadian clock of the fruit fly, *Drosophila melanogaster*.^5–9^ Early lesion and transplantation studies located the circadian clock of *Rhyparobia* (*Leucophaea*) *maderae* in the brain’s optic lobes.^1,2^ Finally, the accessory medulla (AME) in the brain’s optic lobes, a brain area innervated by approximately 240 neurons, was identified as the location of the cockroach circadian clock.^10,11^ The AME controls rest-activity (sleep-wake) cycles via neuropeptide release entrained to light−dark cycles. It is closely connected to the visual system as well as to postsynaptic circadian clock cells in the midbrain that are connected to premotor- and hormone release circuits.^5,9^ The best studied neuropeptide of the insect circadian clock is pigment dispersing factor (PDF) that controls circadian sleep-wake cycles in insects such as the *Rhyparobia maderae* and the fruitfly *Drosophila melanogaster*.^5,9–14^ The clock neurons can be distinguished according to their soma location, immunolabeling using antibodies that recognize different neuropeptide families, have revealed an abundance of partially colocalized neuropeptide immunoreactivities in the cockroach clock neurons^12,15–17^ and specific branching patterns indicative of their function.^18,19^ It has been established that distinct circadian clock neurons/neuronal tracts connect the AME to compound eye photoreceptors, with the potential to act as photic entrainment pathways, allowing circadian rhythmicity in physiology and behavior to be orchestrated in synchronization with the external 24-h cycle. Behavioral plasticity in metazoans, which include insects and humans, has been linked to changes in neuromodulators, such as neuropeptides.^20–22^ Neuropeptides represents a unique class of first messengers guiding almost all physiological processes and behavioral patterns by orchestrating neuronal networks in the central and peripheral nervous systems. The discovery that neuropeptides are produced from prohormone precursors in neurons rapidly led to the identification of the various enzymes involved in prohormone processing as well as mature neuropeptides and protein hormones with potential biological function.^23,24^

The identity and function of all potential mature neuropeptides of the circadian clock in *R. maderae* remain to be determined. In order to conduct a functional analysis of the cockroach clock, it was first necessary to identify the circadian neuropeptidome of *R. maderae*. The next step was to analyze whole brain extracts of *R. maderae* by Quadrupole Orbitrap MS Coupled to nanoflow HPLC and individual neuronal tissue samples by direct tissue profiling using MALDI-TOF/TOF MS. This was done to determine the actual set of processed neuroactive compounds, including the most common post-translational modifications (PTM) in insect neuropeptides. These included the C-terminal amidation of the hydroxyl group of glycine, cyclization of the N-terminal glutamine and aspartate to pyroglutamic acid, formation of disulfide bridges between thiol groups in two cysteine residues, and sulfation. Subsequently, the objective was to ascertain the presence of these neuropeptides within the circadian clock network of the AME of the Madeira cockroach. To this end, the AME neuropil, together with the innervating soma groups, was dissected and the resulting extracts were subjected to analysis using Orbitrap MS. Subsequently, specific soma groups of AME neurons were microdissected and analyzed by direct tissue profiling using MALDI-TOF/TOF MS, followed by tandem MS for the evaluation of the remaining peptide sequences. The findings of this study provide indispensable data for future functional peptidomics at the single-cell level, enabling the investigation of the dynamics of neuropeptides that underpin the time-dependent orchestration of diverse neuronal circuits.

## Material and Methods

### Animals

Adult male cockroaches, *Rhyparobia maderae*, were obtained from laboratory colonies. The insects were reared under a 12:12-h light-dark photoperiod, with illumination provided from 8 a.m. to 8 p.m. The humidity was maintained at 50% relative humidity, and the temperature of the room was set to 25 °C. The animals were provided with a diet of dried dog food, potatoes and water ad libitum. The animals that were used in this study were treated pursuant to the tenets of the EU Directive 2010/63/EU (Europe).

### Classification of Analytes

In this study, we employed the classification of analytes as previously outlined by Habenstein and colleagues.^25^ The term “neuropeptide” is used to describe peptide molecules of a length up to 45 amino acids that are produced by neuronal and endocrine cells from larger preproproteins that contain a signal peptide and canonical prohormone convertase processing sites. Neuropeptides can act as neurotransmitters, neuromodulators or hormones via G-protein-coupled receptor (GPCR) signaling. The term “neuropeptide-like peptides” refers to a category of endogenous peptides that exhibit most of the characteristics typically associated with neuropeptides, but whose specific functions and receptor interactions are largely unknown. Protein hormones are peptides comprising more than 45 amino acids that are produced by neuronal and endocrine cells from larger preproprotein precursors with a signal peptide. In insects, these hormones are secreted into the hemolymph, where they regulate processes in distant target organs. Precursor peptides (PP) are biologically inactive, shorter peptide sequences that are produced during the processing of preproproteins which cannot be turned into an active form by posttranslational modification.

### Transcriptome Analysis

#### RNA Extraction

Five brains (containing 85 mg RNA), Malpighian tubules (containing 40 mg RNA) and ten antennae (containing 70 mg) of *R. maderae* were pooled at different Zeitgebertimes (ZT; ZT = 1–2 and ZT = 13–14) preserved in TRIzol (Life Technologies Inc.) at 4 °C and total RNA was isolated in accordance with the manufacturer’s instructions.

#### Library Construction and Transcriptome Sequencing

RNAseq library (mRNA by polyA capture) was prepared from total RNA and sequenced at Microsynth AG (Balgach Switzerland). The library was sequenced on an Illumina NextSeq 500, v2, system (Illumina, San Diego, USA) with 2 × 150 bps paired-ends, resulting in 183 871 416 read pairs.

#### De Novo Assembly

Raw sequenced reads were initially filtered by removing adapters and subsequently trimmed of low-quality bases (Phred quality score below 25) from both ends using Trimmomatic 0.39 (ILLUMINACLIP:TruSeq3-PE-2.fa:2:30:10 LEADING:25 TRAILING:25 SLIDINGWINDOW:4:25 MINLEN:50).^26^ Remaining reads longer than 50 bp were quality checked (FastQC 0.11.9) and assembled *de novo* using IDBA-Tran 1.1.3 (−mink 19–maxk 124–step 5–max_isoforms 1–min_contig 200).^27^ Completeness of the resulting assembly was assessed using the metazoa data set of BUSCO 5.4.6.^28^ Complete BUSCOs: 97.3% [Single: 87.6%, Duplicated: 9.7%], Fragmented BUSCOs: 1.8%, Missing BUSCOs: 0.9%, Total BUSCOs: 954) in transcriptome mode and submitted to NCBI (BioProject: PRJNA1133961, BioSample: SAMN42391691, Transcriptome Shotgun Assembly (TSA): pending for release).

#### Compiling of Precursor Sequences

The tBLASTn algorithm from the BLAST+ suite command-line tool (v2.4.0.)^29^ was used to conduct database searches for *R. maderae* neuropeptide precursor sequences. As reference queries, selected sequences of known insect neuropeptide precursors from different nonpterygote hexapods^30^ and pterygote insects such as the honeybee *Apis mellifera*,^31^ the yellow fever mosquito *Aedes aegypti*,^32^ the ant *Cataglyphis nodus*,^25^ the stick insect *Carausius morosus*,^33^ and the American cockroach *Periplaneta americana* ^34^ were employed. Identified sequences were translated into proteins using ExPASy translate tool (http://web.expasy.org/translate/, Swiss Institute of Bioinformatics, Switzerland).^35^ Signal peptides (SP) were predicted using the SignalP 6.0 server (www.cbs.dtu.dk/services/SignalP/, Technical University of Denmark, Denmark). If no SP could be predicted or no stop codon was present, precursors were considered as incomplete. Cleavage sites, potential neuropeptides and precursor peptides (PPs) were calculated and manually assigned based on Veenstra.^36^ For multiple sequence alignments, the software ClustalW (https://www.genome.jp/tools-bin/clustalw) was used.

### Tissue Preparation for Mass Spectrometry

The cockroaches were anaesthetised on ice and decapitated. The body cavities and head capsules were opened and their contents were transferred to ice-cold physiological insect saline (128 mM NaCl, 2.7 mM KCl, 2 mM CaCl_2_, 1.2 mM NaHCO_3_, pH 7.25). Tissue samples (see Supporting Information S1) from different Zeitgebertimes (ZTs); beginning of the day [ZT0], mid-day [ZT6], beginning of the night [ZT12] and midnight [ZT18] were used for mass spectrometric analysis to uncover the set of neuropeptides processed in the AME as complete as possible. A semiquantification analysis of neuropeptides at different ZTs is not included.

#### Tissue Extraction

A total of 12 distinct sample sets were obtained, comprising 5 × 5 brains, 2 × 5 retrocerebral complexes (RCCs), 3 × 10 AMEs, and 2 × 20 AMEs. Each sample set was collected in a 30 μL extraction solution containing 50% methanol, 49% H_2_O and 1% formic acid (FA) on ice. The tissue samples were homogenized using an ultrasonic bath for a period of 2 h on ice. Subsequently, the samples were subjected to centrifugation for a period of 15 min at 13 000 rpm at 4 °C. Subsequently, the supernatants were separated and then evaporated in a vacuum concentrator in order to remove the methanol. The extracts were stored at −20 °C until required for use.

#### Direct Tissue Profiling

The neurohemal organs and glands (abdominal perisympathetic organs [PSOs], *n* = 10, thoracic PSOs [*n* = 20], neurohemal [*n* = 8] and glandular portion [*n* = 10] of the *corpora cardiaca*, *corpora allata* [*n* = 3]), frontal ganglion (*n* = 5), and portions of the accessory medullae (AME; *n* = 20) were dissected in accordance with the methodology described by Predel.^37^ The tissue samples were then dissected, separated, and the surrounding tissues and fat bodies were removed under a stereo microscope. Subsequently, the tissue portions were transferred using a glass capillary fitted to a tube with a mouthpiece into a drop of purified water onto a sample plate for MALDI-TOF mass spectrometric analysis, as previously described by Schachtner and colleagues.^38^ Subsequently, the water was removed with the same glass capillary, and each sample was left to dry at room temperature prior to matrix application.

### Quadrupole Orbitrap MS Coupled to Nanoflow HPLC

Before injecting the samples into the nanoLC system, extracts were desalted using self-packed Stage Tip C18 (IVA Analysentechnik e.K., Meerbusch, Germany) spin columns.^39^ For analysis, peptides were separated on an EASY nanoLC 1000 UPLC system (Thermo Fisher Scientific, Waltham, MA) using RPC18 columns 50 cm (fused Silica tube with ID 50 μm ± 3 μm, OD 150 μm ± 6 μm, Reprosil 1.9 μm, pore diameter 60 Å, Dr. Maisch, Ammerbuch-Entringen, Germany), and a binary buffer system (A: 0.1% FA, B: 80% ACN, 0.1% FA), as described for *Cimex* samples.^40^ Running conditions were as follows: linear gradient from 2 to 62% B in 110 min, 62 to 75% B in 30 min, and final washing from 75 to 95% B in 6 min (45 °C, flow rate 250 nL/min). Finally, the gradients were re-equilibrated for 4 min at 5% B. The HPLC was coupled to a Q-Exactive Plus (Thermo Scientific, Bremen, Germany) mass spectrometer. MS data were acquired in a top10 data-dependent method dynamically choosing the most abundant peptide ions from the respective survey scans in a mass range of 300–3000 *m*/*z* for HCD fragmentation. Full MS^1^ acquisitions ran with 70 000 resolution, with automatic gain control target (AGC target) at 3e6 and maximum injection time at 80 ms. HCD spectra were measured with a resolution of 35 000, AGC target at 3e6, maximum injection time at 240 ms, 28 eV normalized collision energy, and dynamic exclusion set at 25 s. The instrument was run in peptide recognition mode (i.e., from two to eight charges), singly charged and unassigned precursor ions were excluded. Raw data were analyzed with PEAKS Studio 10.5 (BSI, ON, Canada). Neuropeptides were searched against an internal database comprising *R. maderae* peptide precursor sequences with parent mass error tolerance of 0.1 Da and fragment mass error tolerance of 0.05 Da. Only predicted peptides with a minimum amino acid length of five amino acid residues, a maximum molecular weight of 10 kDa and a P-score >60 were considered. The false discovery rate (FDR) was determined by a decoy database search as implement in PEAKS 10.5 and set below 1%. The maximum number of posttranslational modifications (PTMs; sulfation, C-terminal amidation, methylation, disulfide bridge, oxidation, N-pyroglutamyl formation [Gly, Glu], phosphorylation, acetylation [K, N-terminus]), per peptide was set to three as variable PTMs. None enzyme mode was selected. In order to provide the accurate monoisotopic mass of a peptide, the Q Exactive Orbitrap RAW data were corrected prior to analysis (only precursor mass correction was performed). The fragmentation of spectra was undertaken with a peptide score of −10 log P, which is equivalent to a P-value of approximately 1%. These spectra were then subjected to manual review.

### Matrix Application

For neuropeptiomics using direct tissue profiling, two different matrices were applied: α-cyano-4-hydroxycinnamic acid (CHCA) and 2,5-dihydroxybenzoic acid (DHB). CHCA has been demonstrated to be an excellent tool for the analysis of high-resolution mass spectra of hydrophobic peptides and small proteins, at a mass range of *m*/*z* 100–2500 Da. DHB is considered to be more suitable when dealing with polar compounds, demonstrating better ionization efficiency for larger biomolecules at a mass range of greater than 2500 Da. Furthermore, CHCA is often produces stronger precursor ions for peptides. This is particularly advantageous in tandem MS (MS/MS), as it generates clearer, more interpretable fragmentation patterns (b/y ion series), especially for peptides, comparable to DHB.

The matrix stock solutions, comprising 10 mg/mL DHB (Sigma-Aldrich, Steinheim, Germany) dissolved in 20% acetonitrile/1% FA/79% water (Fluka) or 10 mg/mL CHCA (Sigma-Aldrich, Steinheim, Germany) dissolved in 60% ethanol, 36% acetonitrile, 4% water, were utilized. Dried tissue samples were covered with either 0.1 μL of a DHB solution or 0.1 μL of a mixture of one part CHCA stock solution and three parts 50% methanol/water. This was done using a 0.1–2.5 μL Eppendorf pipet (Eppendorf AB, Hamburg, Germany). All DHB spots were blow-dried with a commercially available hair dryer in order to form homogeneous crystals.

### MALDI-TOF/TOF MS

Mass spectra were acquired manually using an ultraflex TOF/TOF mass spectrometer (MS) or an ultrafleXtreme TOF/TOF MS (Bruker Daltonik GmbH, Bremen, Germany). Both instruments were operated in reflector positive ion mode within the 600–10 000 Da mass range. The instrument settings were optimized for the mass range of 600–4000 Da and 3000–10 000 Da, respectively. Proteins or peptides with a predicted ion mass exceeding 10 000 Da were not included in the present study. The following synthetic peptide mixture was used for calibration purposes: proctolin, *Drosophila melanogaster* (Drm)-sNPF-1_4–11_, *Locusta migratoria* periviscerokinin-1 (PVK-1), *Periplaneta americana* (Pea)-FMRFa-12, *Manduca sexta* allatotropin (AT), Drm-IPNa, Pea-SKN and glucagon for the mass range at *m*/*z* 600–4000. In order to calibrate the mass range at *m*/*z* 3000–10 000, a mixture of glucagon, bovine insulin-A, and ubiquitin was employed. Laser fluence was calibrated to achieve an optimal signal-to-noise ratio. The data were subsequently processed using the FlexAnalysis V.3.4 software package. The number of laser shots varied between 2000 and 20 000, contingent on the quality of the ion signal. Additionally, an ABI 4800 proteomics analyzer (Applied Biosystems, Framingham, USA) was employed for MS/MS experiments. MS/MS fragment spectra were acquired manually in gas-off mode and subsequently processed and handled using the DataExplorer V.4.10 software package. Peptide identities were verified by comparison of the masses of the theoretical fragments (http://prospector.ucsf.edu) with those of the experimentally obtained fragments.

### Immunohistochemistry

Whole mount brains were prepared as previously described and fixed with 4% formaldehyde in 0.1 M phosphate-buffered saline (PBS) for overnight at 4 °C. Following fixation, the samples were rinsed with Tris-buffered saline (PBS, pH 7.4) containing 0.3% Triton X-100 (0.3% TX) for a period of 24 h. Subsequently, the samples were dehydrated with ethanol at varying concentrations (50%, 70%, and 100%) for 10 min each. They were then incubated in Roticlear (Carl Roth, Germany) for 30 min, followed by a rehydration procedure with 100%, 70%, and 50% ethanol for 10 min, respectively. Following rehydration, the samples were rinsed with TBS (pH 7.4) for a period of 10 min. Following rinsing, the samples were incubated in a solution of 1 mg/mL collagenase/Dispase (Sigma-Aldrich, Germany) for 10 min at 37 °C. To halt the enzymatic process, the samples were rinsed in a solution comprising 9 mL of 0.85% NaCl and 1 mL of 0.1% HCl for a period of 2 h. Subsequently, the samples were preincubated overnight in a mixture of TBS 0.3% TX containing 5% normal goat serum (NGS, Jackson ImmunoResearch Laboratories, PA, USA) and 0.02% sodium azide (Sigma-Aldrich, Germany) at 4 °C. Following three washes with TBS for 8 min each, the samples were incubated with polyclonal rabbit anti-Proctolin serum at a concentration of 1:800, diluted in TBS-0.3% TX containing 2% NGS and 0.02% sodium azide, for 4 days at 4 °C. Subsequently, the samples were washed in TBS-0.3% TX overnight at 4 °C, followed by incubation with Cy3-coupled goat anti-rabbit serum at a concentration of 1:300 (Jackson ImmunoResearch Laboratories, PA, USA) for 2 days at 4 °C. After washing in TBS-0.3% TX overnight at 4 °C, the samples were embedded in Mowiol (Merck KGaA, Darmstadt, Germany).

### Confocal Laser Scanning Microscopy

The samples were scanned as image stacks with an optical thickness of 0.5–1.5 μm at a resolution of 1024 × 1024 pixels using a Leica TCS SP5 confocal laser-scanning microscope, equipped with a 20.0 × 0.7 multi-immersion objective (Leica Microsystems AG, Wetzlar, Germany). The serial optical sections were processed and merged using the maximum projection option. The images were exported and processed using the ImageJ 1.53c software (developed by Wayne Rasband, National Institutes of Health, Bethesda, Maryland, USA) and the Java 1.8.0_172 (64-bit) engine to adjust brightness and contrast.

## Results and Discussion

A combination of transcriptomics, peptidomics and immunolabeling techniques was employed to obtain a comprehensive analysis of the neuropeptidome of the Madeira cockroach’s nervous system. Based on these data, the neuropeptide composition of the cockroach circadian clock, the accessory medulla (AME) was characterized. The objective of this characterization was to enable future functional investigations, with the aim of gaining new insight into the peptidergic orchestration of the circadian clock system.

### Transcriptomics-Based Prediction of Neuropeptide, Neuropeptide-Like and Protein Hormone Precursor Genes

To identify neuropeptides associated with the circadian system, BLAST searches were conducted against the transcriptome of *R. maderae* using a data set of prepropeptides from various insect species as queries.^25,31–34^ In total, a set of 68 *R. maderae* prepropeptide sequences were identified, consisting of 43 neuropeptide genes, 8 complete neuropeptide-like genes and 18 complete protein hormone genes (Table 1, Supporting Information S2).

**Table 1:** Transcriptome of *R. maderae* Has Revealed the Presence of Prepropeptide Genes for Neuropeptides, Neuropeptide-Like and Protein Hormones^a^

designation	accession	complete
**Neuropeptides**		
Adipokinetic hormone (AKH-1)	PQ049256	+
Adipokinetic hormone-2 (AKH-2)	PQ049257	+
AKH/corazonin-like hormone precursor (ACP)	PQ049260	+
Allatotropin (AT)	PQ049261	+
Allatostatin-A (Ast-A)	PQ049262	+
Allatostatin-B/Myoinhibitory peptide (MIP)	PQ049263	+
Allatostatin-CC (Ast-CC)	PQ049264	+
Allatostatin-CCC (Ast-CCC)	PQ049265	+
Arginine–vasopressin-like peptide/Inotocin	PQ049266	+
Calcitonin B	PQ049267	(+)
Calcitonin-like diuretic hormone-31 (DH-31)	PQ049268	+
Corticotropin releasing factor (CRF)-like diuretic hormone-46 (DH-46)	PQ049269	+
CAPA/Periviscerokinin (CAPA-PVK)	PQ049270	+
CCHamide-1	PQ049271	+
CCHamide-2	PQ049272	+
CNMamide-A	PQ049273	(+)
CNMamide-B	PQ049274	+
Corazonin (Crz)	PQ049275	+
Crustacean cardioactive peptide (CCAP)	PQ049276	+
CCRFamide	PQ049277	+
Elevenin (Elv)	PQ049278	+
extended FMRFamide (FMRFa)	PQ049279	+
Hansolin	PQ049282	+
ITG-like peptide (ITG)	PQ049283	+
Kinin (K)	PQ049286	+
Myosuppressin (MS)	PQ049287	+
Natalisin (Nat)	PQ049288	+
long Neuropeptide F-1a (NPF-1a)	PQ049289	+
long Neuropeptide F-1b (NPF-1b)	PQ049290	(+)
long Neuropeptide F-2 (NPF-2)	PQ049291	+
Orcokinin-A (Orc-A)	PQ049296	+
Orcokinin-B (Orc-B)	PQ049297	(+)
Pigment dispersing factor (PDF)	PQ049298	+
Pyrokinin/FXPRLamides (PK)	PQ049299	+
Proctolin	PQ049300	+
RFLamide	PQ049301	+
RYamide	PQ049302	+
short neuropeptide F (sNPF)	PQ049303	+
SIFamide	PQ049304	+
SMYamide	PQ049305	+
Sulfakinin (SK)	PQ049306	+
Tachykinin-related peptide (TK)	PQ049307	+
Trissin	PQ049308	+
**Neuropeptide-like**		
Agatoxin-like peptide-1 (ALP-1)	PQ049258	+
Agatoxin-like peptide-2 (ALP-2)	PQ049259	(+)
Pea FERLQ-like peptide	PQ049280	+
Fliktin (Flik)	PQ049281	+
Neuropeptide-like precursor-1 (NPLP-1)	PQ049293	+
Carausius Neuropeptide-like precursor-1 (CNPLP-1)	PQ049294	+
NVP-like peptide-A (NVP-A)	PQ300805	+
NVP-like peptide-B (NVP-B)	PQ300806	+
**Protein Hormones**		
Bursicon-alpha	PQ049309	(+)
Eclosion hormone-1	PQ049310	+
Eclosion hormone-2	PQ049311	+
Glycoprotein hormone alpha	PQ049312	+
Glycoprotein hormone beta	PQ049313	+
IDL-containing peptide-A	PQ300807	+
IDL-containing peptide-B	PQ300808	+
Insulin-like peptide-1	PQ049316	+
Insulin-like peptide-2	PQ049317	+
Insulin-like peptide-3	PQ049318	(+)
Insulin-like peptide-4	PQ049319	+
Insulin-like peptide-5	PQ049320	+
Insulin-like peptide-6	PQ049321	+
Ion transport peptide	PQ049284	+
Neuroparsin	PQ049322	(+)
Prothoracicotropic hormone	PQ049323	+
Invertebrate parathyroid hormone	PQ049324	+

a The precursor sequences have been catalogued in Supplementary Information S2, which also considers partial sequences (+). precursor sequences have been annotated and submitted to GenBank (BioProject: PRJNA1133961, BioSample: SAMN42391691).

Four neuropeptide precursor genes (*calcitonin B*, *CNMamide-A*, *long neuropeptide F-1b [lnpf-1b]) orcokinin-B [orc-B])*, one neuropeptide-like gene (*agatoxin-like peptide-2 [alp-2]),* and three protein hormone precursors (*eclosion hormone-1, insulin-like peptide-3 [ilp-3], neuroparsin*) were incomplete at the N-terminus of the prepropeptides. As only amino acids at the signal peptide sequence were absent in all eight cases, no further information about mature neuropeptides or neuropeptide-like peptides was compromised. For *alp, adipokinetic hormone (akh), IDL-containing peptide*, and *lnpf-1*, alternatively spliced peptide mRNAs were obtained. Alternative splicing during gene expression is not uncommon in insects and were described also in the American cockroach *Periplaneta americana*,^34^ the stick insect *C. morosus*,^33^ locusts, termites,^41^ kissing bugs,^42^ the fruitfly *D. melanogaster*,^43^ and various ant species.^25,44^ Alternative gene-splicing dependent products of the *capa*, *nplp-1, itp*, *calcitonin* genes found in other insects^25^ was not found in the *R. maderae* transcriptome. Furthermore, the identification of two *capa* cDNA transcripts in *Periplaneta americana*,^45^ *Solenopsis invicta* ^44^ *and* *C. morosus* ^33^ has been documented. These transcripts are distinguished by a shorter and a longer variant of the N-terminal precursor sequences. However, the analysis of the *R. maderae* transcriptome yielded the identification of only a single *capa* cDNA transcript. In different insect species, *capa* is significantly more highly expressed in neurosecretory cells of the abdominal ventral nervous system than in central brain neurons.^46,47^ Thus, it is possible that a single transcript of the *capa* gene was identified in the Madeira cockroach, given that the transcriptomic analysis was conducted, without incorporating the ventral nervous system. As previously described in the transcriptome of the cockroach *P. americana*,^34^ the neuropeptide genes *hansolin* and *RFLamide* were also identified in the Madeira cockroach transcriptome. *hansolin* was initially identified in the transcriptome and peptidome of *C. morosus*.^33^ However, its function in insects remains unknown. The two neuropeptide-like precursor proteins, *carausius neuropeptide-like-precursor-1 (cnplp-1)* and *fliktin (flik)*, have also been identified in the Madeira cockroach. The *cnplp-1* was initially identified in the stick insect *C. morosus*,^33^ while the *flik* gene was previously described in the ant species *C. nodus*.^25^ A multiple sequence alignment of *R. maderae* *flik* with *C. nodus* *flik* and the previously described novel *P. americana* neuropeptide gene PaOGS36577 revealed high amino acid sequence similarity (Supporting Information S3), suggesting that they might be orthologous genes. The *R. maderae* transcriptome also revealed the presence of a *trissin* precursor, which encodes a potential single C-terminal nonamidated trissin peptide consisting of 26 amino acids. This finding corroborates previous observations regarding this neuropeptide gene in various polyneopteran orders.^48^ Neuropeptide precursors known from other insects, such as *EFLamide, calcitonin-A, ecdysis-triggering hormone (eth), 11in-like peptides 1,2,3, gonadulin, relaxin-like peptide, tryptopyrokinin* and *pyrokinin-like peptide,* were not found in *R. maderae* assembled transcriptome data (see Table 1). Except for the *pyrokinin-like peptide* gene, which has been identified previously only in the locust *Locusta migratoria*,^49^ all other precursors that were absent in *R. maderae* were found in the genome of the cockroach *P. americana*.^34^ Given the high degree of conservation observed in the production and secretion of ETH by Inka cells along the tracheal network across a diverse range of insect species,^50,51^ the absence of *eth* detection in the assembled transcriptome was an anticipated outcome. As the genome of *R. maderae* has not been completely sequenced, the search for these precursors is currently not possible.

### Neuropeptidomics *of* *R. maderae* Neuronal Samples by Quadrupole Orbitrap MS and Direct Tissue Profiling by MALDI-TOF/TOF MS

The transcriptome-predicted neuropeptides and neuropeptide-like prepropeptides, as listed in Supporting Information S2, were selected as the basis for neuropeptidomics. The present study does not investigate putative bioactive molecules encoded by protein hormone genes, with the exception of *ion transport peptide (itp).* In the initial phase of the study, extracts from entire brains (*n* = 5) and retrocerebral complexes (RCC; *n* = 5), a structure bearing similarity to the pituitary gland observed in mammals, were subjected to analysis via Quadrupole Orbitrap MS. This was followed by a comparison of the detected peptide fragments with the calculated fragmentation pattern generated by PEAKS 10.5 software. The resulting tandem MS data revealed peptide sequence identification of products from 21 genes encoding single neuropeptides or neuropeptide-like peptides (*akh 1,2, alp-1, acp, allatotropin [at], allatostatin ccc [ast-ccc], calcitonin-like diuretic hormone-31 [dh-31]*, *corticotropin-releasing-factor [CRF]-like-diuretic hormone-46 [dh-46], cch-amide-2, cnm-amide-B, corazonin [crz], crustacean cardioactive peptide [ccap], Pea ferlq-like, hansolin, itp, myosuppressin [MS], long neuropeptide F-1a, long neuropeptide F-1b, short neuropeptide F [sNPF], pdf, sif-amide)* and peptides from 17 genes containing multiple copies (*allatostatin-A [ast-A], allatostatine-B/myoinhibitory peptide, [ast-B/mip], Capa-peptides/periviscerokinin [pvk], extended fmrf-amides, flik, kinin, natalisin [nat], neuropeptide-like precursor1 [nplp1], cnplp1, nvp-like peptide, orcokinin-A [orc-A], orcokinin-B [orc-B], pyrokinin [pk]/fxprlamides, ry-amide, sulfakinin [sk] and tachykinin-related peptide [tk]* (Table 2and Supporting Information S4). The amino acid sequences of 37 products from 13 neuropeptide genes were previously reported in *R. maderae* and were confirmed in the current study. These include AKH,^52^ seven MIPs,^17^ AT and Orc-A,^53^ CAPA-PVK-1,2,3 ^74^ and CAPA-PK-1 (designed as CAPA-PK-5,^54^ corazonin,^55^ one pyrokinin,^56^ eight kinins,^57–59^ myosuppressin,^60^ PDF,^61^ SIFamide,^62^ SK-1 and SK-2,^63,64^ and 14 TKs.^65,66^ Nässel et al. employed Edman degradation and mass spectrometry during the purification of TKs from *R. maderae* brains, thereby identifying the nonamidated peptide sequence DNSQWGGFA–OH. The novel peptide was designated “baratin”.^67^ BLAST searches against the *R. maderae* transcriptome revealed sequence matches with a peptide sequence encoded by the NVP-like peptide gene, which was identified as DNSQWGGFA. A multiple sequence alignment of the *R. maderae* NVP-like precursor with the NVP-like precursors identified from *P. americana* (accession no. PaSCF32060 ^34^) and *C. morosus* (accession no. GFAX01119786 ^33^) reveals high sequence similarity in the amino acid sequences (asterisks), indicating that baratin is a product of the NVP-like precursor, particularly the truncated form of NVP-2. (Supporting Information S5A). The analysis of the brain extract (Supporting Information S4) and direct tissue profiling of AME tissue samples (Supporting Information S5B) confirmed the presence of NVP-2, which is distinguished by the inclusion of two additional amino acids, Lys and Asp, at the C-terminus of DNSQWGGFA. This indicates that Lys at position 10 may function as a cleavage site.

**Table 2:**
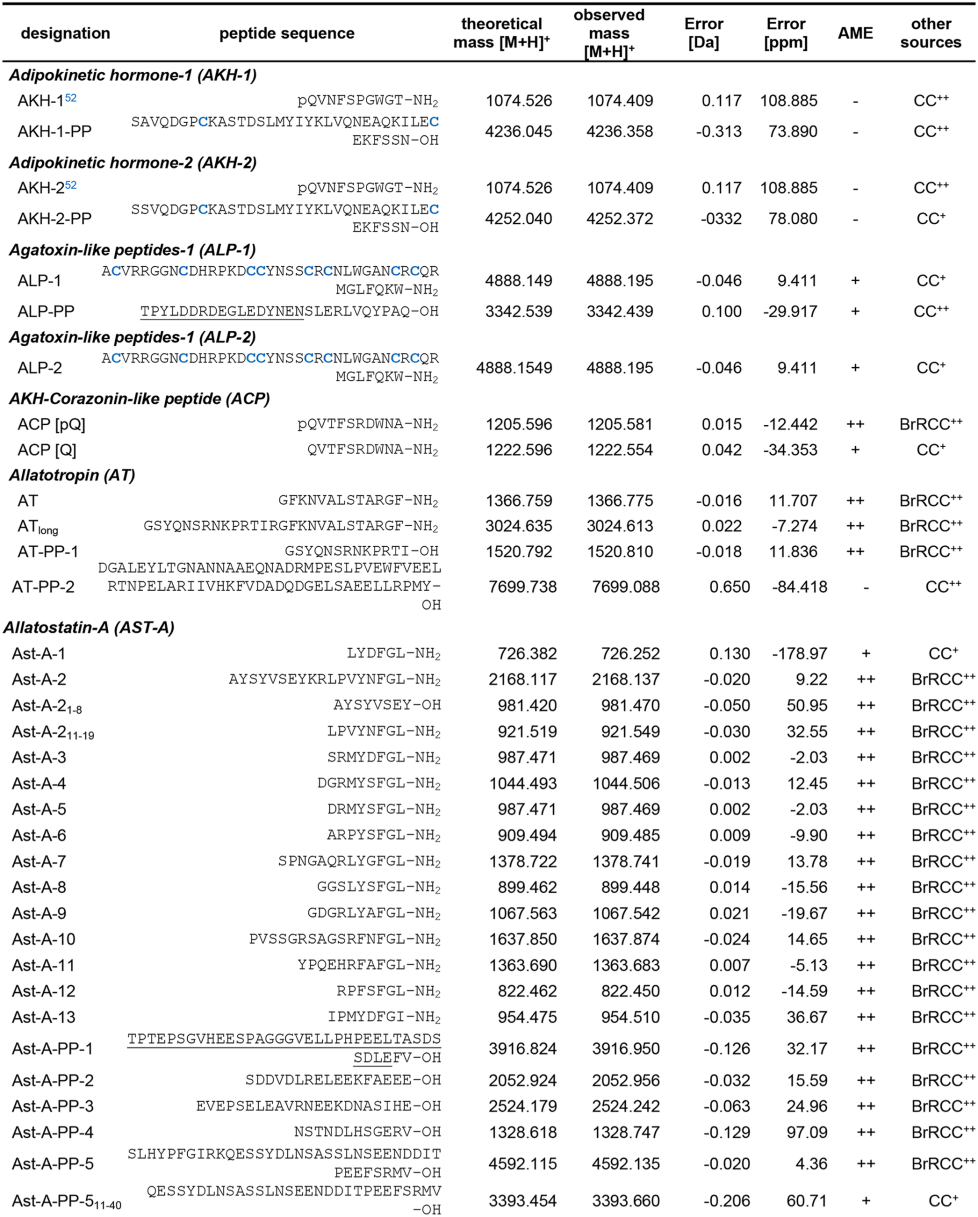

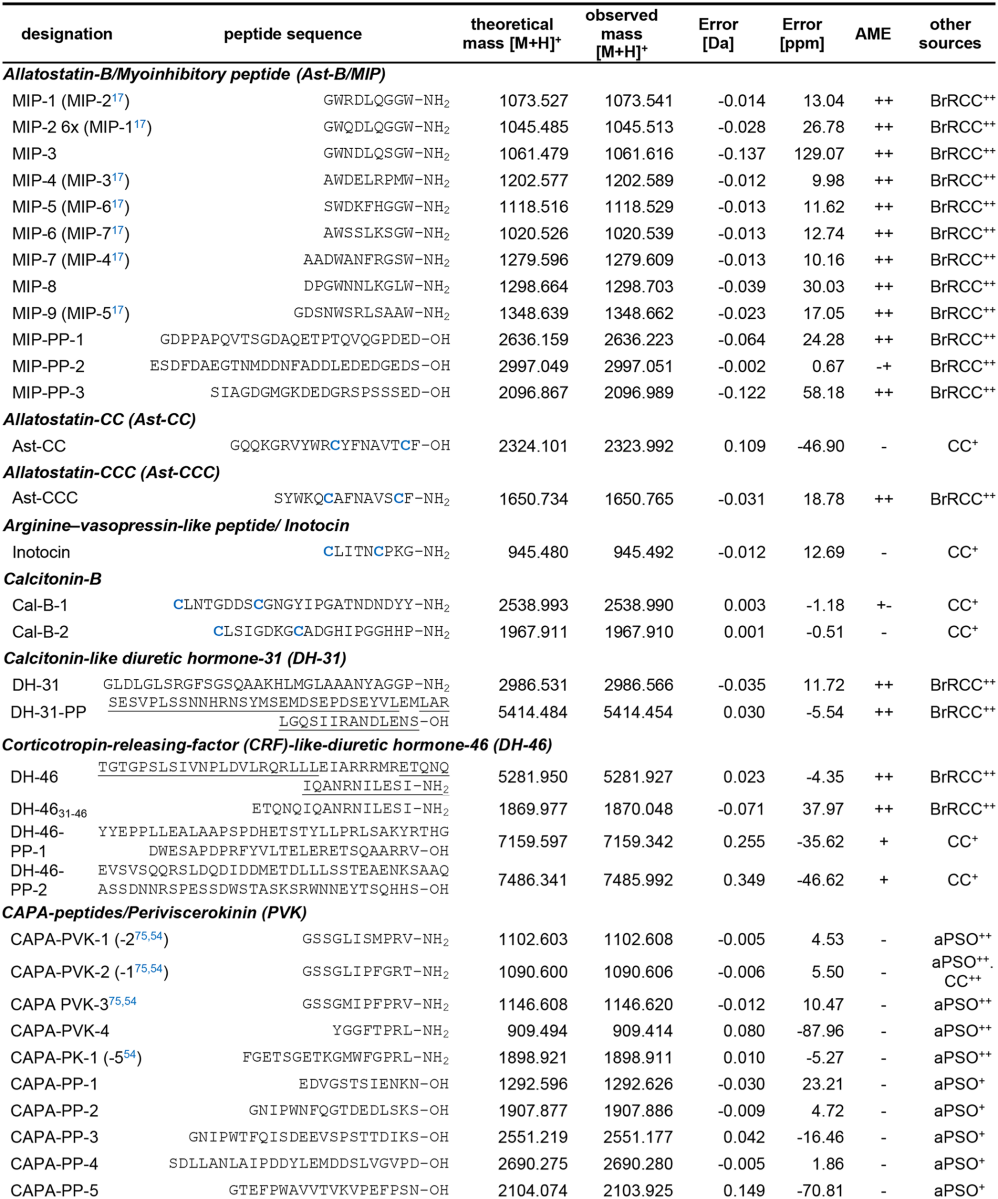

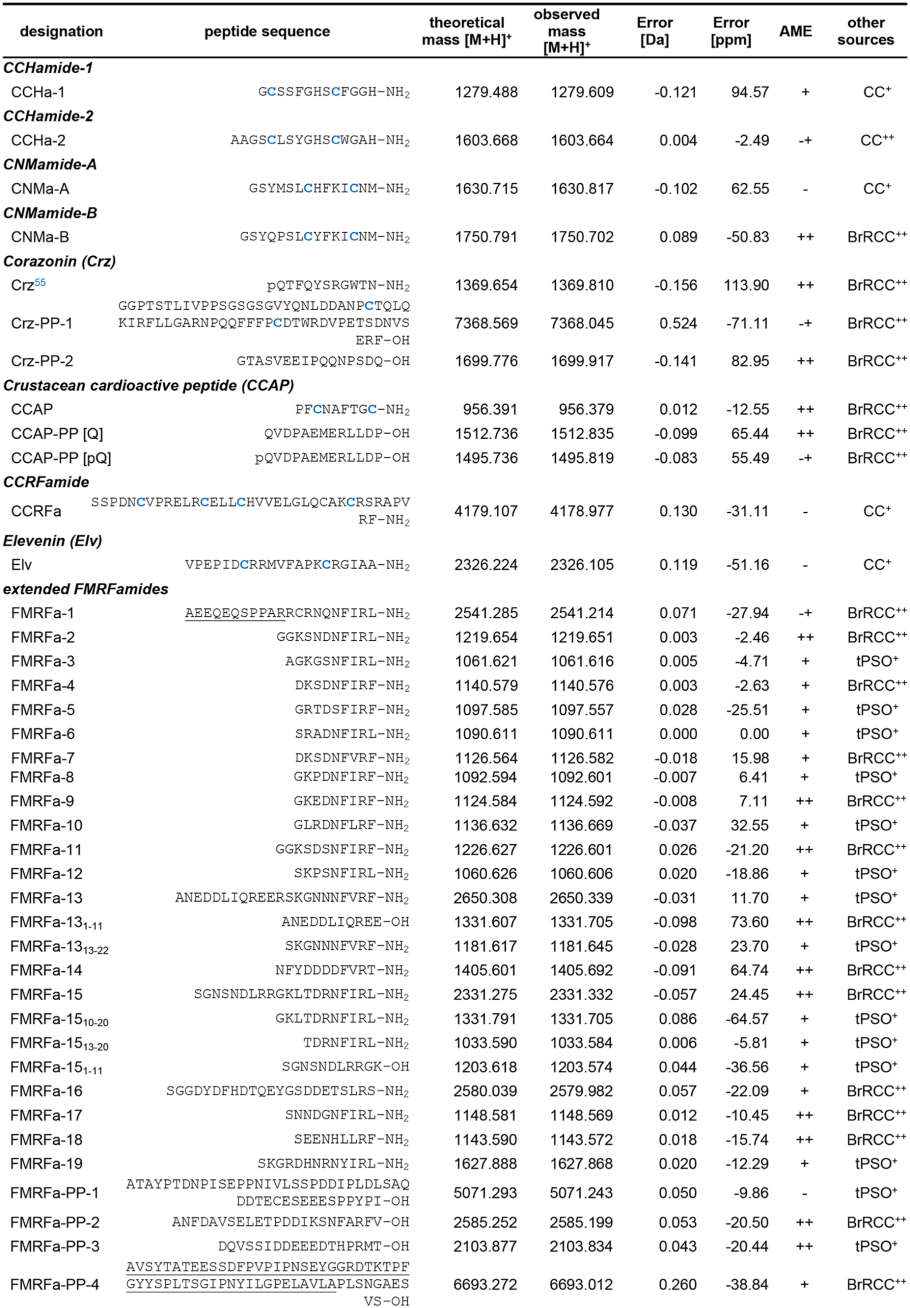

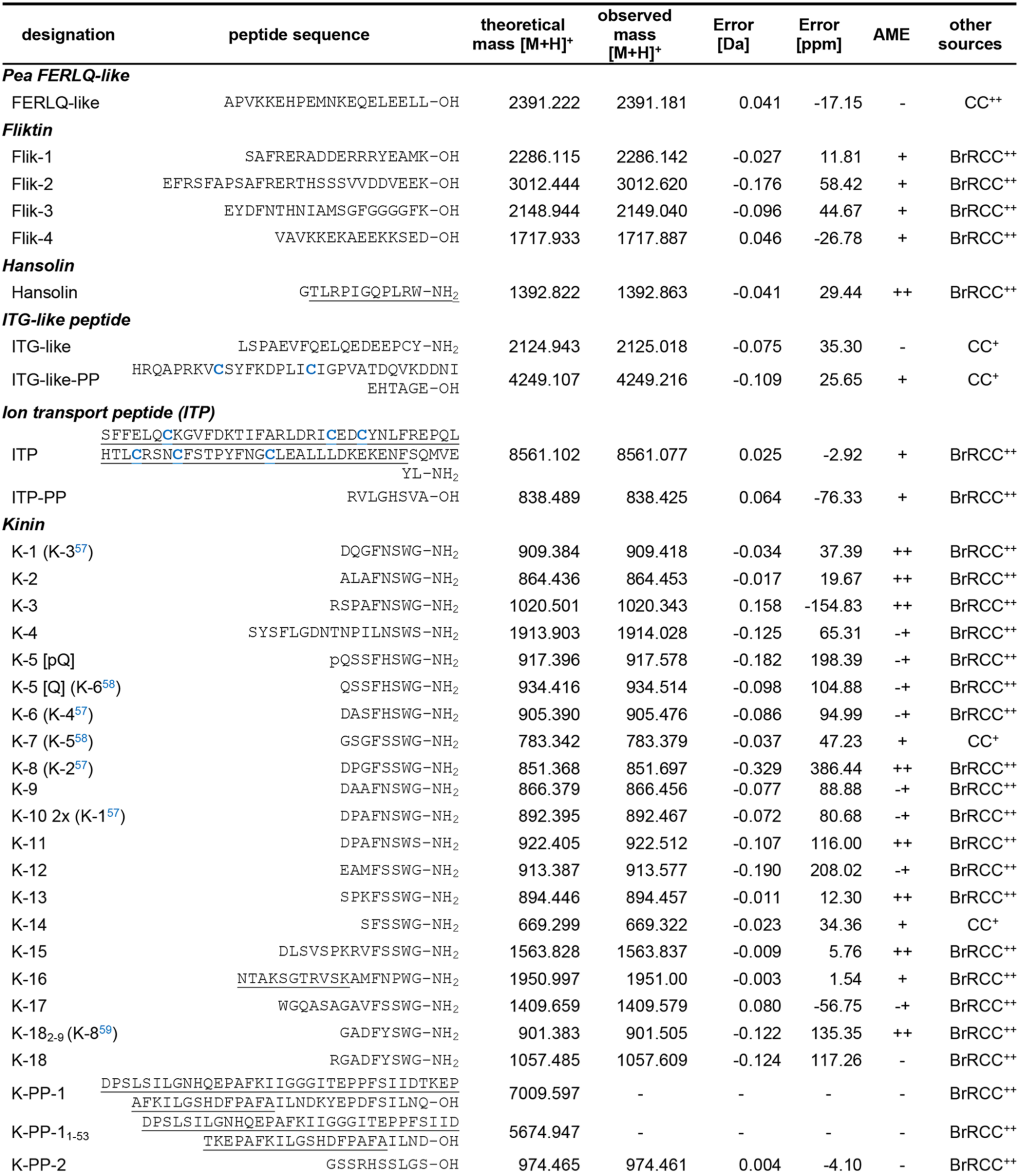

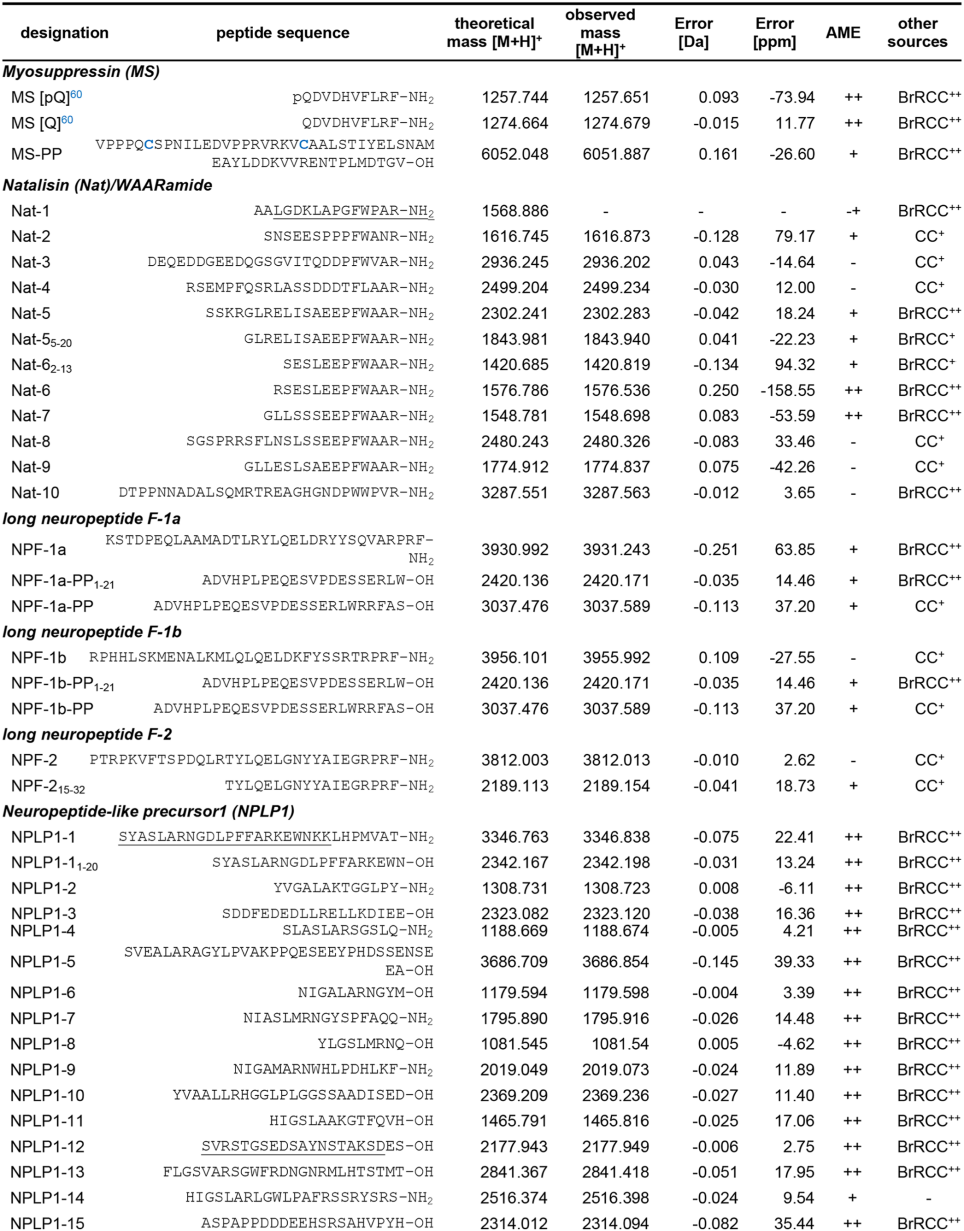

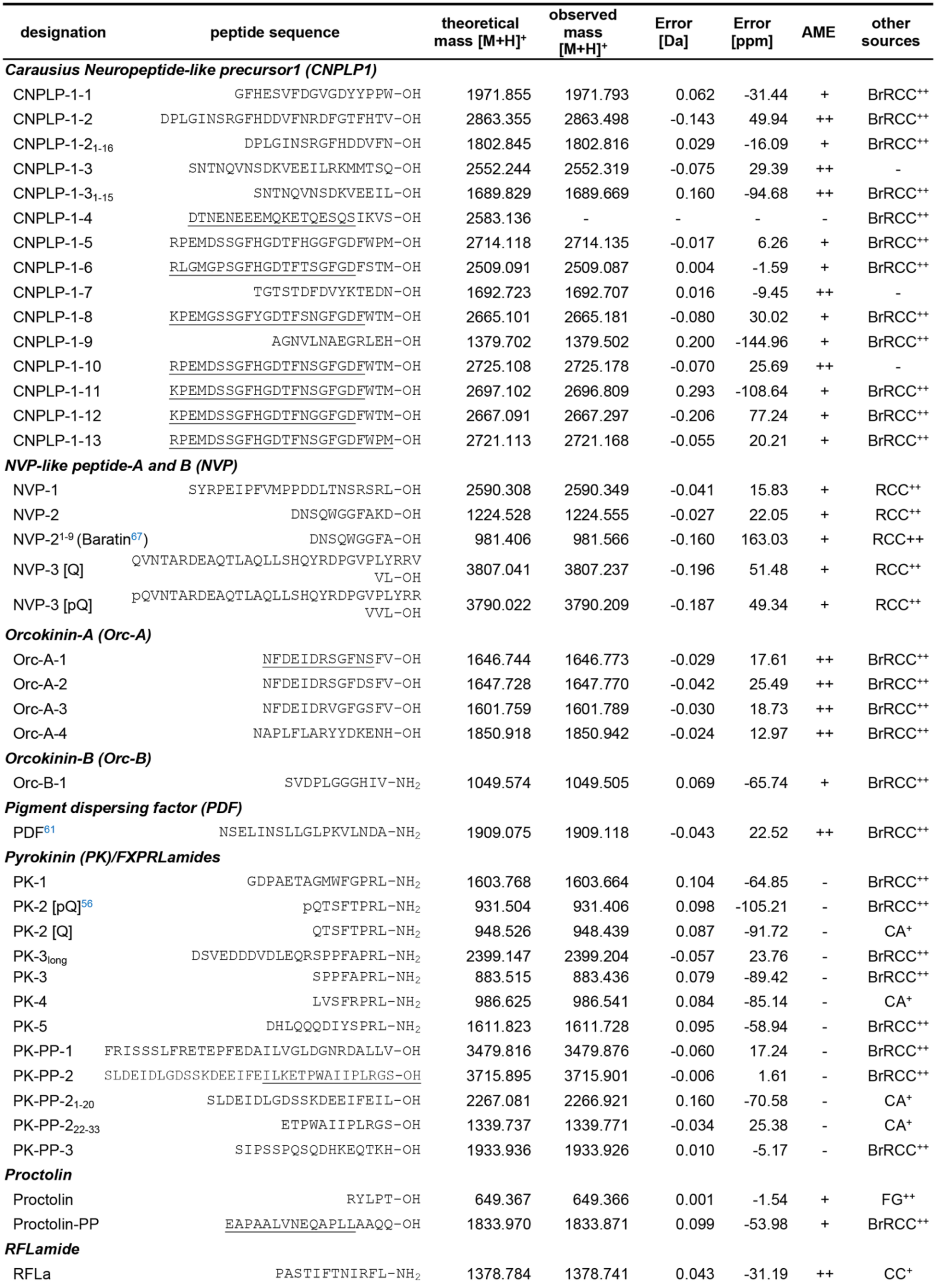

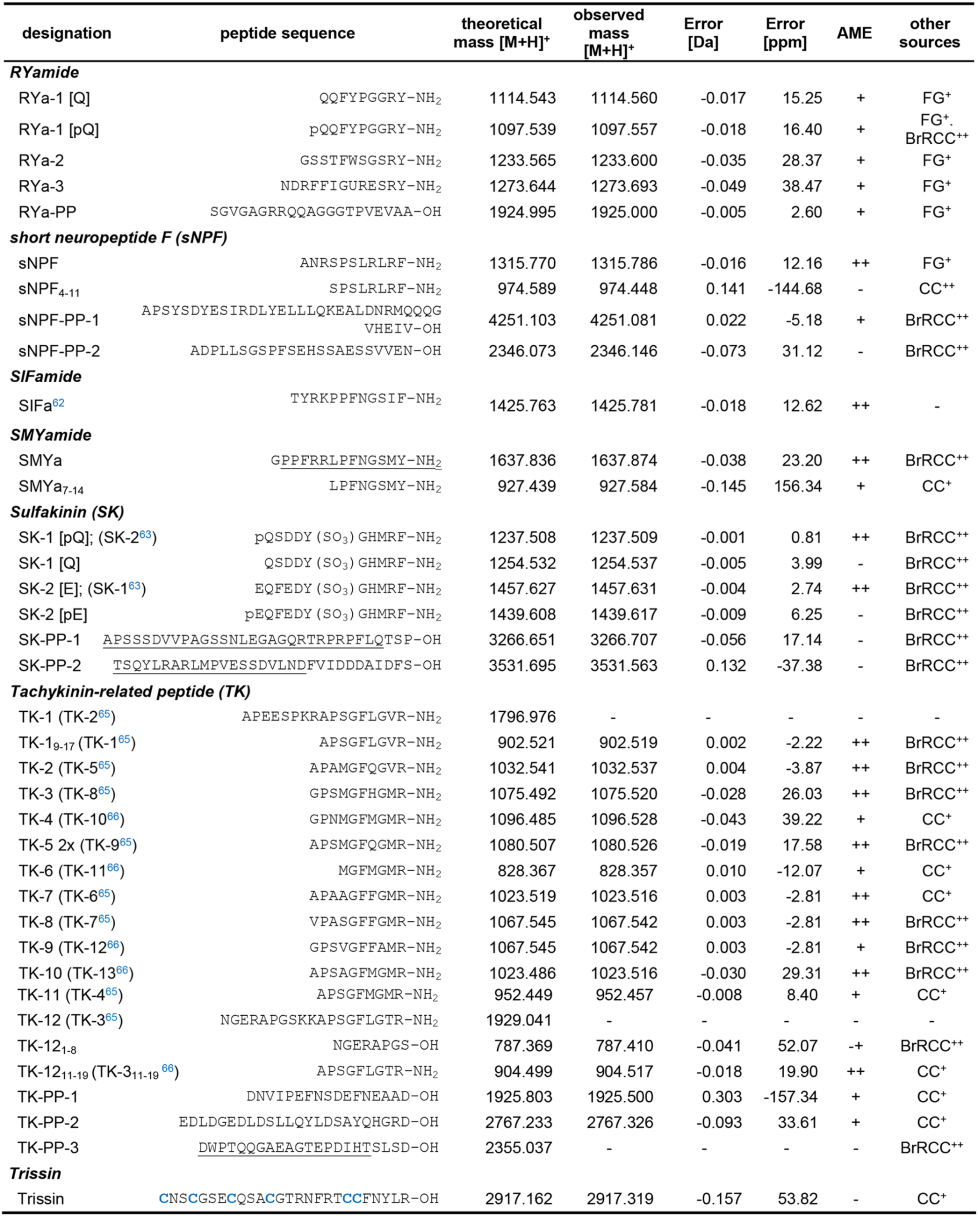
Alphabetic List of Mature Neuropeptides, Neuropeptide-Like Peptides and Precursor Peptides (PP) of the *R. maderae* Brain, AME, and Selective Neuronal Tissue Samples Analyzed Using Brain Tissue Extraction Analyzed by ESI-Q-TOF Mass Spectrometry (MS) and/or Direct Tissue Profiling by MALDI-TOF (MS) (See Supplementary Information S1 and S4)­^a^

a MSMS experiments were used for peptide sequence confirmation (bracket: a part of the peptide amino acid sequence is confirmed). Putative bioactive peptides, which were not found in the AME were observed in other neuronal tissues such as the *corpora cardiaca* (CC), *corpora allata* (CA), *ganglion frontalis* (FG), abdominal perisympathetic organ (aPSO) and or thoracic perisympathetic organ (tPSO). Due to the lack of basic amino acids , AKH is detected as sodium [M + Na]^+^ and potassium [M+K]^+^ adducts. Mass matches are represented by a +, amino acid sequence confirmation by ++, and partial amino acid sequence confirmation are underlined. BrRCC, brain/retrocerebral complex.

In a previous study, direct tissue profiling of the AME region of *R. maderae* was utilized to partially identify the amino acid sequence of five MIPs.^17^ As the MIP gene had not yet been identified, the MIPs were named according to their ion masses, from lowest to highest,^17^ as observed in the resulting MALDI mass spectrum. Here, following the established nomenclature for neuropeptides, the MIPs were renamed according to their position within the precursor gene, as has been done for previously identified products derived from the *kinin*, *pk/fxprl*-amide, *sk* and *tk* precursor genes (see Table 2). Additionally, the Madeira neuropeptidome comprises products of the *carausius neuropeptide-like precursor 1* and *hansolin* gene. These were initially identified in *C. morosus* ^33^ and subsequently validated in the American cockroach *P. americana* as well.^34^ Furthermore, the neuropeptide-like gene *fliktin* was identified in both the brain and RCC samples of the Madeira cockroach, indicating a potential hormonal function in *R. maderae*. This is consistent with previous observations in the ant *C. nodus*.^25^

To identify additional neuropeptides and peptide gene products in the mass range of 600–10 000 Da, which were not detected by brain or RCC extract using Orbitrap MS, tissue profiling from the preparation of the RCC (*n* = 20), thoracic (*n* = 20) and abdominal (*n* = 10) perisympathetic organs (PSO) was conducted using matrix-assisted laser desorption/ionization time-of-flight (MALDI-TOF) mass spectrometry. The RCC, comprising the paired *corpora cardiaca* (CC) and *corpora allata* (CA) in cockroaches, was subjected to a more detailed analysis. The CC can be subdivided into a glandular part, comprising non-neuronal endocrine cells, and a neurohemal part, which contains axonal endings of three distinct nerve bundles (*nervi corpora cardiaci-1* (NCC-1), NCC-2, NCC-3) originating from neurosecretory cells in the *pars intercerebralis* (NCC-1), the *pars lateralis* (NCC-2), and the tritocerebrum (NCC-3). Representative mass spectra of the glandular part of the CC (Figure 1A, *n* = 10) and the neurohemal part (Figure 1B, *n* = 8) are shown in the mass range of 900–3000 Da.

**Figure 1:**
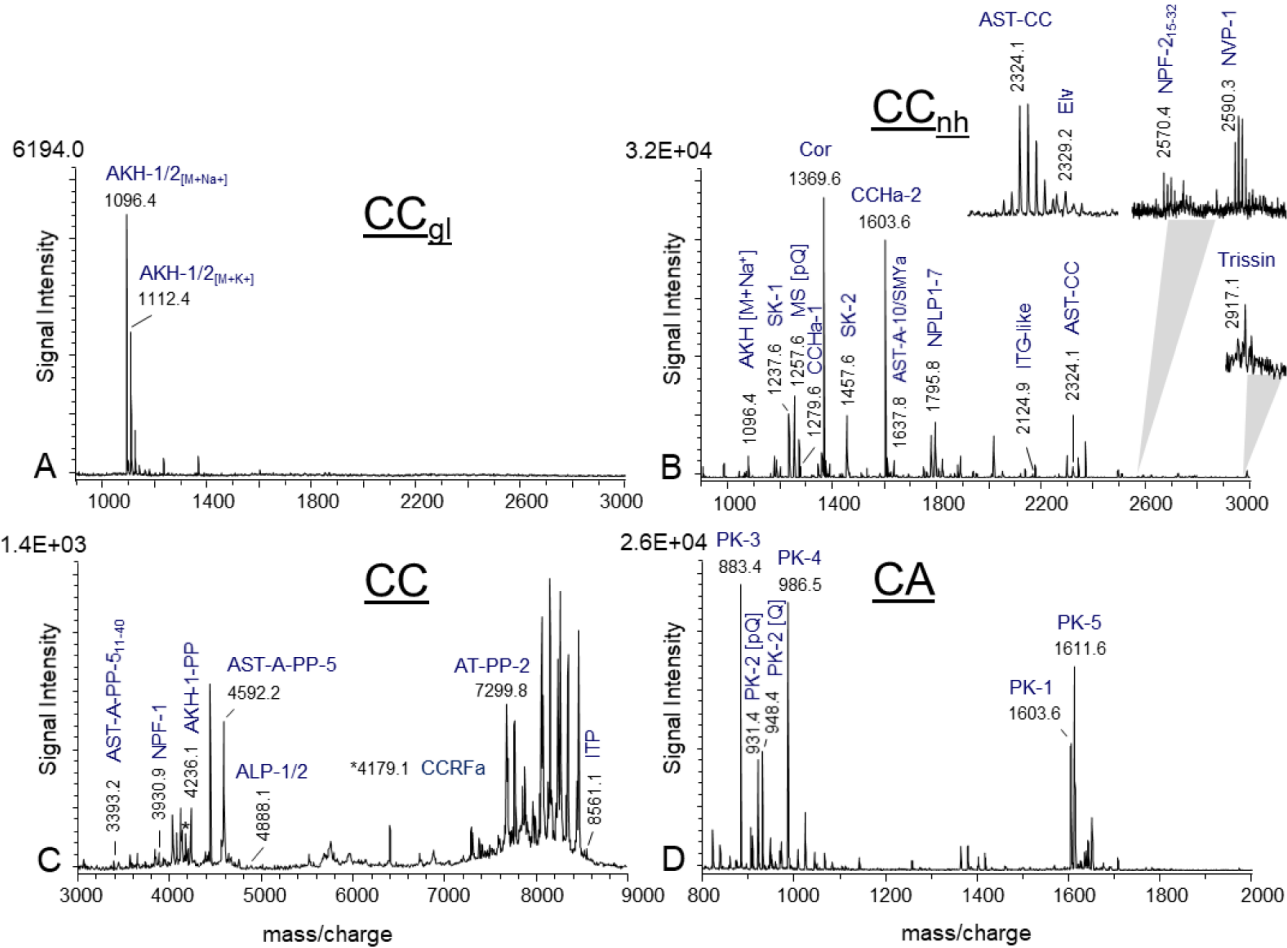
Representative MALDI-TOF mass spectra (direct tissue profiling) obtained from preparations of different parts of the retrocerebral complex (RCC, *n* = 20). (A) Mass spectrum of the glandular part of the *corpora cardiaca* (CCgl, *n* = 10), (B) the neurohemal portion of the CC (CCnh, *n* = 8), (C) tissue portion containing both CCgl and CCnh (*n* = 6), and (D) the *corpora allata* (CA, *n* = 3). Spectra were acquired with different settings which have been optimized for the given mass ranges, respectively. Ion signals are marked and represent single charged peptides [M + H]^+^. Adipokinetic hormone (AKH) is detected only as [M + Na]^+^ and [M+K]^+^. ALP, Agatoxin-like peptides; Ast-A, allatostatin-A; Ast-CC, allatostatin-CC; Cor, Corazonin; ITP, ion transport peptide; Elv, Elevenin; MS, myosuppressin; NPY, neuropeptide Y; NVP, NVP-containing peptide; NPLP-1, neuropeptide-like precursor 1; NPF, long neuropeptide F; PK, pyrokinin; PP, precursor peptide; SK, sulfakinin.

The mass spectrum of the glandular part of CC yielded confirmation of the presence of the “insect glucagon” AKH, which is exclusively detected as sodium adduct [M + Na]^+^ and potassium adduct [M+K]^+^ (Figure 1A). In *R. maderae*, two distinct transcripts of the AKH gene encode both AKH-1 and AKH-2, yet the amino acid sequences are identical. The differences between the *akh* transcripts are observed in the amino acid sequence of the precursor peptide (PP). The amino acid sequence of both AKH-PPs was confirmed by RCC extract analysis using Orbitrap MS (Table 2 and Supporting Information S4) and by mass matches using direct tissue profiling in a mass range of 3000–10 000 Da (Figure 1C). Therefore, both AKHs may be processed from both transcripts. The mass spectra generated from neurohemal CC preparations revealed the predicted ion masses of the following peptides by mass matches: Agatoxin-like peptides-1/2 (ALP-1/2), nonpyroglutamate formation of AKH-Corazonin-like peptide (ACP), Ast-A-1, Ast-CC, CCHa-1, CNMa, CCRFa, Elevenin (Elv), ITG-like peptide, long neuropeptide F-2 (NPF-2), Neuropeptide Y-like (NPY), RFLamide, SIFamide, SMYamide and Trissin (Table 2and Figure 1B,C). Interestingly, the *trissin* precursors have been identified in many species of Polyneoptera, however, a biochemical confirmation is still lacking. In this study, an ion signal with a very low signal intensity (*m*/*z* 2917.1; Figure 1B) was observed in the neurohemal CC preparations. This corresponds to a predicted mass of trissin at *m*/*z* 2917.16. However, due to the low signal intensity, it was not possible to perform sequence confirmation by subsequent fragmentation experiment. In the field of peptidomics, it is not uncommon for the calculated ion masses of different predicted peptides to be similar or even identical. To illustrate, the predicted ion masses of MIP-7 (*m*/*z* 1279.59) and CCHa-1 (*m*/*z* 1279.49) exhibit a mass error of 0.1 Da (or 78 ppm). In this instance, it was not possible to differentiate between MIP-7 and CCHa-1 through the application of either mass fingerprinting or tandem MS.

Furthermore, the resulting mass spectra of the CC exhibited mass matches for the ion signal at *m*/*z* 1637.8, which may correspond to the predicted ion masses for Ast-10 (*m*/*z* 1637.85) and/or SMYamide (*m*/*z* 1637.84). However, the ion signal intensity was insufficient for amino acid sequence identification using subsequent tandem MS. It is currently unclear whether these neuropeptides are present or whether the ion signals are corresponding to an unknown peptide. Despite the utilization of a brain/RCC extract, the amino acid sequences of all predicted products of the *pyrokinin (pk)/fxprlamides* gene could not be identified. However, a previous study of the pyrokinin/FXPRLamide system in the cockroach *P. americana* revealed that three cell clusters, located in the mandibular, maxillary, and labial neuromeres of the subesophageal ganglion, extend into the retrocerebral complex via the *nervous corpora allati-2* (NCA-2) and NCC-3. The fibers of the NCA-2 traverse the CA and continue toward the postallatal nerves.^68^ The resulting mass spectrum from CA preparations is presented in Figure 1D, which revealed mass matches corresponding to PK-2 and PK-4, thereby confirming the presence of these peptides in the neuropeptidome of *R. maderae*. The remaining processing information required for the predicted products of the *extended fmrf-amide* gene, which could not be confirmed by brain/RCC extract analysis, was obtained by MALDI direct tissue profiling of thoracic perisympathetic organ (tPSO) preparations (*n* = 20) (Figure 2).

**Figure 2:**
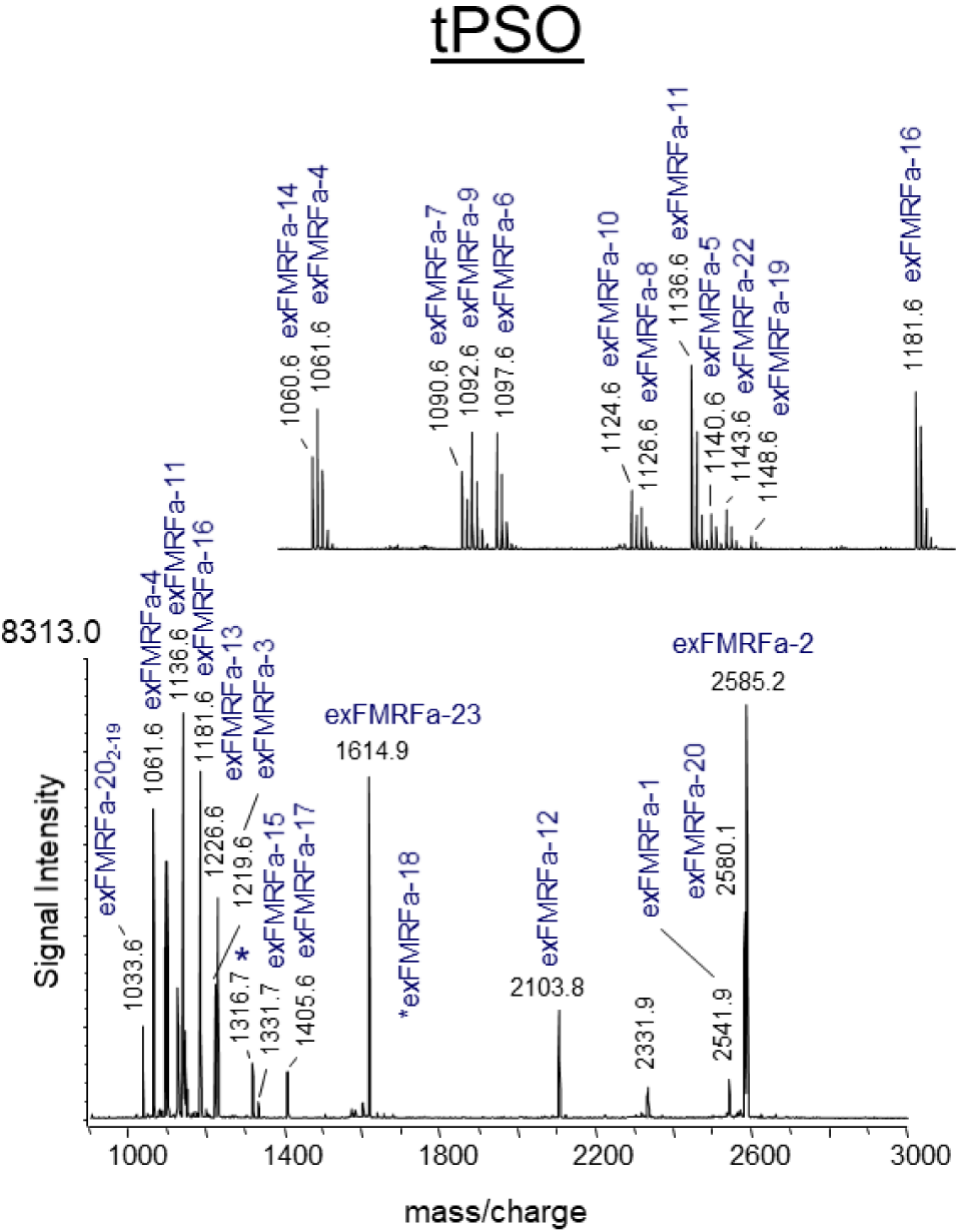
Representative MALDI-TOF mass spectrum of a thoracic perisympathetic organ (tPSO) in the mass range *m*/*z* 900–3000. The numbering of the extended FMRFamides (FMRFa) corresponds to their position in the *extended fmrfamides* gene. Mass matches to predicted peptides are highlighted and represent single charged peptides [M + H]^+^. PP, precursor peptide.

The products of the *extended fmrfamides* gene are synthesized in two groups of neurosecretory cells, located in a posterior-lateral position in each of the three thoracic ganglia.^69^ These cells are referred to as the postero-lateral cells (PLCs). The PLCs that neurosecrete the tPSO are homologous to T_v_ neurons in *D. melanogaster*.^70^ Additionally, ion signals corresponding to transcriptome-predicted sequences for *Capa-peptides/periviscerokinin (pvk)* gene were identified in preparation of the abdominal neurohemal organs, the abdominal perisympathetic organs (aPSO, *n* = 10, Figure 3). Median neurosecretory cells located in the abdominal ganglion synthesizes CAPA peptides and provide the aPSO with neurosecretion.^46^ Furthermore, the ion signal intensities of both proctolin (*m*/*z* 649.4) and pigment dispersing factor (PDF, *m*/*z* 1909.07) were insufficient to confirm their amino acid sequences by brain/RCC extract analysis.

**Figure 3:**
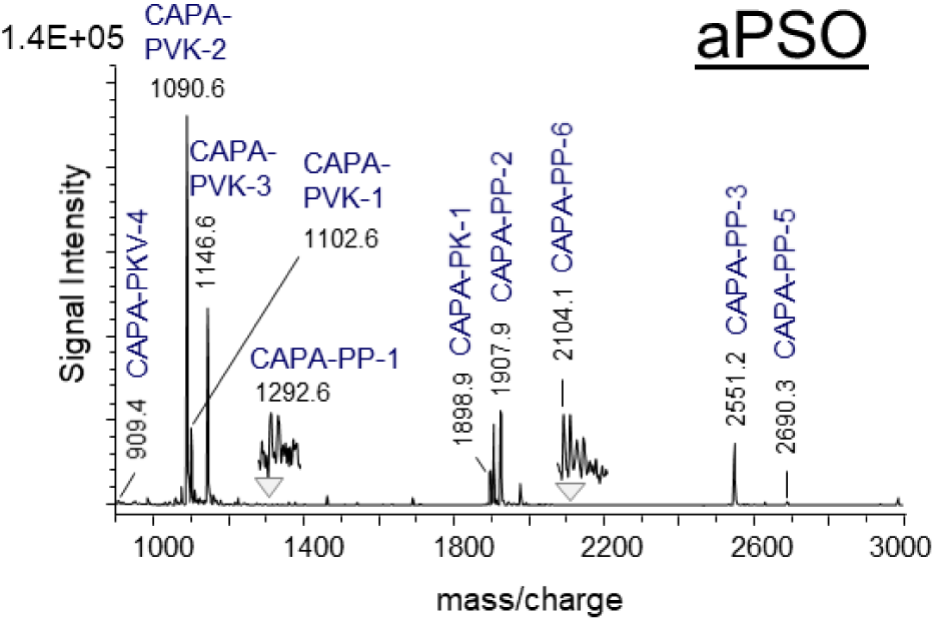
MALDI-TOF mass spectrum obtained by direct tissue profiling of the abdominal perisympathetic organ (aPSO). Ion signals mass-identical with predictable peptides from the initially identified *Capa* cDNA are labeled. Ion signals are single-charged ions and are labeled in accordance with their position in the gene. PVK, periviscerokinin; PP, precursor peptide.

However, a mass spectrum generated from a preparation of the *R. maderae* frontal ganglion revealed an ion signal corresponding to the predicted ion mass of proctolin (not shown) which was confirmed by subsequent tandem MS. (Supporting Information S4). The frontal ganglion is part of the insect stomatogastric nervous system which contains many proctolin-*like* immunoreactive neurons in insects.^71^ The absence of an ion signal corresponding to PDF, which has been previously identified in *R. maderae* brain extracts,^61^ may be attributed to the lower quantity of extract material used in this study.

### Neuropeptidomics of the Accessory Medulla (AME)

A further objective of this study is to analyze the accessory medulla (AME), which is the circadian clock controlling sleep-wake cycles in cockroaches. In previous studies, immunohistochemical staining was employed to determine the localization of putative neuropeptides to specific AME cells using a range of antibodies. These included stainings against MIP,^17,53^ corazonin,^12^ neuropeptides belonging to the RFamide family,^12,72,73^ PDF^12^ and SIFamide.^62^ However, the majority of antibodies are not highly specific for a single peptide form and frequently cross-react with peptides containing similar sequence motifs. Consequently, an immunocytochemical analysis alone cannot confirm the presence of a neuropeptide in a specific cell. To determine which neuropeptides and neuropeptide-like substances are present in the cockroach circadian clock, extracts of isolated AMEs (*n* = 3 × 10 AMEs and *n* = 2 × 20 AMEs) were analyzed using Quadropole Orbitrap MS. The neuropeptidome analysis of excised AMEs identified a total of 166 mature neuropeptides and 39 precursor peptides encoded on 41 neuropeptide and neuropeptide-like genes (Table 2).

Nineteen of the genes encode a single copy of neuropeptide/neuropeptide-like products, including *acp, at, ast-ccc, dh-31, dh-46, cch-amide-2, cnm-amide-b, crz, ccap, imf-amide, hansolin, itp, myosuppressin, lnpf-1a, pdf, rfl-amide, snpf, smy, and sif-amide*. Furthermore, ten genes encode multiple copies of neuropeptides, including *ast-a, mip/ast-b, extended FMRF-amide, natalisin, kinin, nplp-1, cnplp-1, orc-A, sk,* and *tk.* The amino acid sequences of the predicted peptidergic gene products were identified from AME extracts via tandem MS analysis (see Table 2 and Supporting Information S4).

Next, to fill in the list of potentially bioactive neuropeptides and neuropeptide-like peptides processed by AME neurons, direct tissue profiling using MALDI-TOF MS was performed on isolated AME preparations (*n* = 20). Representative mass spectra in a mass range of *m*/*z* 600–3000 (Figure 4) and *m*/*z* 3000–9000 Da (Figure 5) revealed mass matches of ion signal corresponding to predicted products from additional seven single copy (*alp1, cal-b, cch-amide-1, itg-like peptides, itp, npf-1a, proctolin*) and four multiple copy (*flik, nvp-like peptide, orc-B, ry-amide*) neuropeptides and neuropeptide-like precursors. With regard to eight precursor proteins (*akh, inotocin, capa/pvk, ccrf-amide, elv, ferlq-like, pk/fxprl, trissin*) no mass matches or confirmation of amino acid sequences of predicted neuropeptides were identified through analysis of the AME extracts nor by direct AME tissue profiling (Table 2).

**Figure 4:**
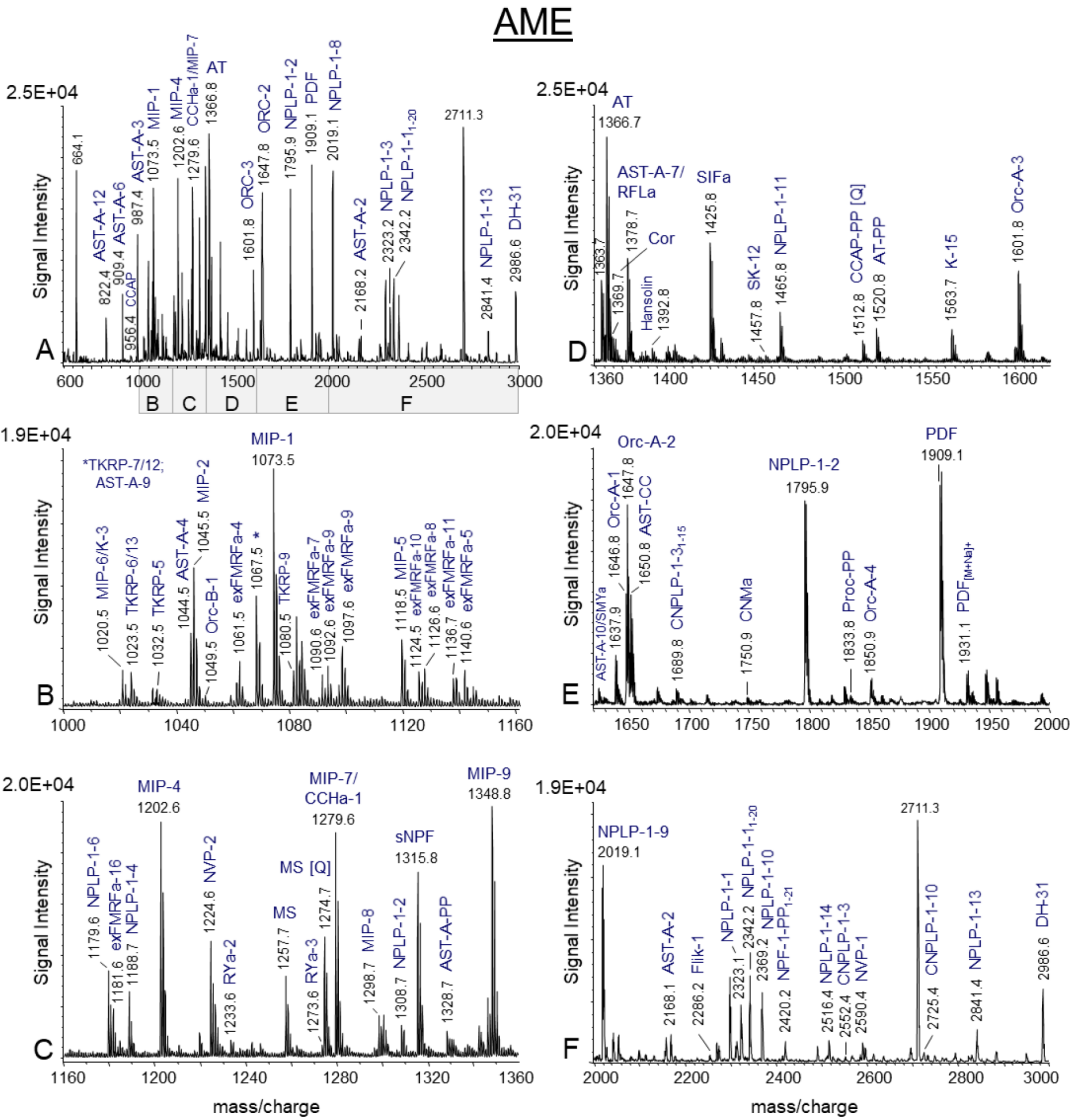
MALDI-TOF mass spectrum of a portion of the accessory medullae (AME) in (A) the mass range of 600–3000 Da. Mass spectrum presenting in (A) is subdivided in the following mass ranges: (B) *m*/*z* 1000–1160; (C) *m*/*z* 1160–1360; (D) *m*/*z* 1360–1600; (E) *m*/*z* 2000–3000, respectively. The most prominent ion signals are labeled and represent mass matches of predicted peptides encoded on neuropeptide and neuropeptide-like genes from the *R. maderae* transcriptome. Ion signals are single charged peptides [M + H]^+^. An explanation of all abbreviations is given in Table 2. PP, precursor peptide.

**Figure 5:**
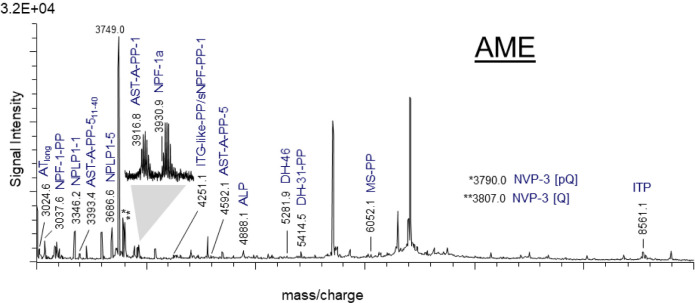
MALDI-TOF mass spectrum of a portion of the accessory medullae (AME) in the mass range of 3000–9000 Da Theoretical masses of the predicted peptides were calculated and compared with the resulting ion signals from the tissue preparation. Mass matches are highlighted and represents single charged peptides [M + H]^+^. An explanation of all abbreviations is given in Table 2. PP, precursor peptide.

Immunostaining is a valuable technique for visualizing the location of specific neuropeptides (monoclonal antibodies) or neuropeptide families (polyclonal antibodies) in particular cells and tissues. It can also be used to support MS-based neuropeptide data. In this instance, this approach was employed to confirm the presence of a neuropeptide of interest which exhibited an ion signal below of the signal-to-noise ratio (S/N) threshold of S/N > 3 for detection. As demonstrated in Figure 6A, the mass spectrum of the AME displays an ion signal at *m*/*z* 649.4, which corresponds with the predicted ion mass of proctolin (Figure 6A). In consideration of the inadequate signal-to-noise ratio (S/N) of the signal in question, the amino acid sequence of the substance could not be confirmed by tandem MS. In order to verify potential proctolin-containing neurons in the AME, immunostaining was performed using an antiserum against proctolin in conjunction with anti-PDF costaining. The role of the PDF staining is to act as a general marker for the localization of the AME, thereby facilitating the delineation of the circadian clock center in the optic lobes. The resulting staining revealed proctolin-*like* immunoreactivity in four neurons of the AME (Figure 6B), and suggesting a function of proctolin in the modulation of timing.

**Figure 6:**
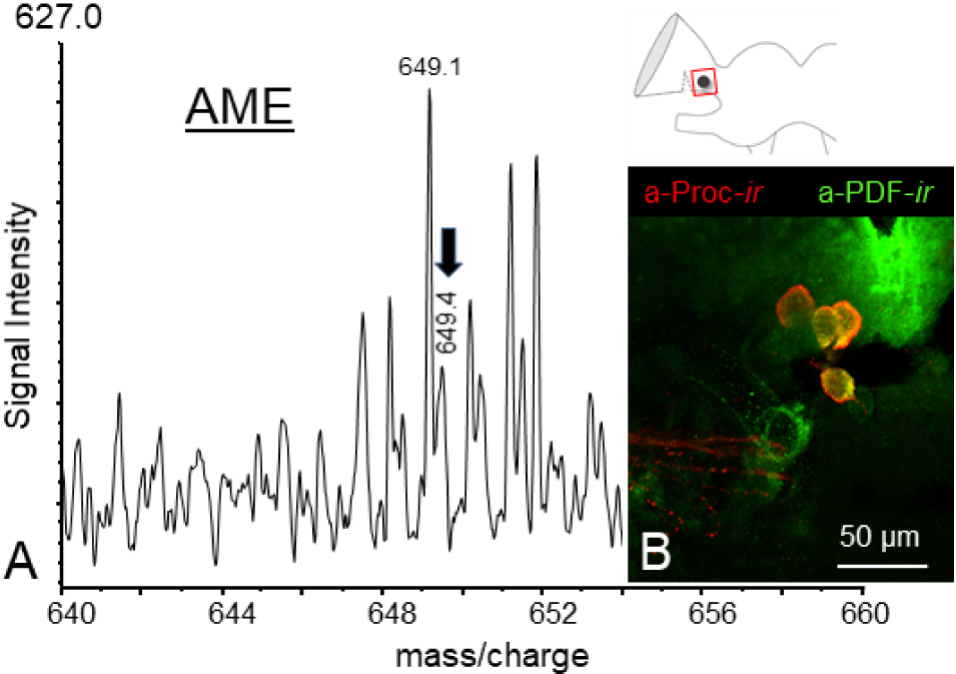
Verification of proctolin in the accessory medullae (AME). (A) Mass spectrum of an isolated portion of the AME. Despite the ion signal at *m*/*z* 649.4 lacking sufficient resolution and not exceeding the signal-to-noise ratio (S/N) of 3σ above the baseline, it corresponds to the predicted mass of the neuropeptide proctolin (Proc, black arrow). Immunostainings were therefore carried out using an anti-proctolin serum to visualize potential proctolin-containing neurons within the AME. (B) As illustrated, four proctolin-*like* immunoreactive cells were labeled in the AME (red). In determining the location of the AME within the brain, double immunostaining was employed, utilizing an antiserum against pigment dispersing factor (PDF, green, refer also to the area designated by the black dot in the scheme).

## Conclusion

This study provides the first comprehensive neuropeptidergic analysis of the circadian clock of the Madeira cockroach, an established chronobiological model system. It remains unclear why both insect and mammalian circadian clock networks are highly enriched in colocalized neuropeptides. The identification of all potential neuropeptides in the circadian clock provides a precise framework for further functional and behavioral assays to understand neuropeptide function in the circadian clock controlling sleep-wake cycles. In future experimental approaches, the integration of semiquantitative mass spectrometric methods holds potential for the analysis of the up-and-down regulation of neuropeptides within the clock network at different ZTs. This approach could facilitate the acquisition of novel insights into the specific roles of clock neurons and the interpretation of circuit-level plasticity. Many neuropeptides are involved in neurological diseases and disorders related to the clock system, including chronic pain, mood disorders, neurodegenerative diseases, and cancer. Identifying and understanding the dynamics of the full repertoire of neuropeptides in a neuronal circuit is therefore essential for the design of targeted therapies, as drugs may need to modulate specific peptide systems to achieve the desired effect.

## Supplementary Material



## Data Availability

The mass spectrometry peptidomics data have been deposited to the PRIDE Archive (http://www.ebi.ac.uk/pride/archive/) via the PRIDE partner repository with the data set identifier PXD068894 and 10.6019/PXD068894.

## Notes

**Supporting Information S1**: Schematic overview of the nervous system of *Rhyparobia maderae* showing tissues that were analyzed in this study by mass spectrometry; **Supporting Information S2**: Prepropeptide proteins for neuropeptides, neuropeptide-like peptides, and protein hormones of *Rhyparobia maderae’s* brain; **Supporting Information S3**: Multiple sequence alignment of *R. maderae fliktin*. (A) with previously described novel *P. americana* neuropeptide PaOGS36577 and the first identified *fliktin* precursor from *C. nodus*, and (B) only with PaOGS36577; **Supporting Information S4**: Quadrupole Orbitrap MS^2^ spectra generated by *R. maderae* brain tissue extract analysis and MALDI-TOF/TOF MS; **Supporting Information S5**: Multiple sequence alignment of baratin and NVP-like peptides from *P. americana* and *C. morosus*, and detection of NVP-like peptides in the AME by mass matches (PDF)

## References

[1] Nishiitsutsuji-Uwo J., Pittendrigh C. S. (1968). Central
nervous system control of the circadian rhythmicity
in the cockroach. Z. Vgl. Physiol..

[2] Page T. L. (1982). Transplantation
of the cockroach circadian pacemaker. Science.

[3] Dorn, A. ; Willis, J. H. ; Hoffmann, D. ; Hoyle, G. ; Wyatt, G. R. ; Bell, W. J. ; Prestwich, G. D. ; Usherwood, P. N. R. ; Brooks, G. T. Comprehensive Insect Physiology, Biochemistry, and Pharmacology, Kerkut, G. A. ; Gilbert, L. I. Eds.; Pergamon Press: Oxford, UK, 1985, Vol. 6, pp. 577–652.

[4] Stengl M., Werckenthin A., Wei H. Y. (2015). How does the circadian
clock tick
in the Madeira cockroach?. Curr. Opinion Insect
Sci..

[5] Homberg U., Reischig T., Stengl M. (2003). Neural organization of the circadian
system of the cockroach Leucophaea maderae. Chronobiol. Int..

[6] Helfrich-Förster C. (2005). Organization
of endogenous clocks in insects. Biochem. Soc.
Trans..

[7] Vansteensel M. J., Michel S., Meijer J. H. (2008). Organization
of cell and tissue circadian
pacemakers: a comparison among species. Brain
Res. Rev..

[8] Mohawk J. A., Green C. B., Takahashi J. S. (2012). Central and peripheral circadian
clocks in mammals. Annu. Rev. Neurosci..

[9] Stengl M., Arendt A. (2016). Peptidergic circadian
clock circuits in the Madeira
cockroach. Curr. Opinion Neurobiol..

[10] Stengl M., Homberg U. (1994). Pigment-dispersing
hormone-immunoreactive neurons in
the cockroach *Leucophaea maderae* share properties
with circadian pacemaker neurons. J. Comp. Physiol.,
A.

[11] Reischig T., Stengl M. (2003). Ectopic transplantation
of differentiated neuronal
graft restores circadian rhythmic behaviour in arrhythmic cockroaches
(*Leucophaea maderae*). J. Exp.
Biol..

[12] Petri B., Stengl M., Würden S., Homberg U. (1995). Immunocytochemical
characterization of the accessory medulla in the cockroach *Leucophaea maderae*. Cell Tissue Res..

[13] Wei H., el Jundi B., Homberg U., Stengl M. (2010). Implementation of pigment-dispersing
factor-immunoreactive neurons in a standardized atlas of the brain
of the cockroach *Leucophaea maderae*. J. Comp. Neurol..

[14] Helfrich-Förster C. (2018). Sleep in Insects. Annu. Rev. Entomol..

[15] Hofer S., Homberg U. (2006). Orcokinin immunoreactivity
in the accessory medulla
of the cockroach *Leucophaea maderae*. Cell Tissue Res..

[16] Soehler S., Neupert S., Predel R., Stengl M. (2008). Examination
of the
role of FMRFamide-related peptides in the circadian clock of the cockroach *Leucophaea maderae*. Cell Tissue Res..

[17] Schulze J., Neupert S., Schmidt L., Predel R., Lamkemeyer T., Homberg U., Stengl M. (2012). Myoinhibitory
peptides in the brain
of the cockroach *Leucophaea maderae* and colocalization
with pigment-dispersing factor in circadian pacemaker cells. J. Comp. Neurol..

[18] Reischig T., Stengl M. (2003). Ultrastructure of pigment-dispersing hormone-immunoreactive
neurons in a three-dimensional model of the accessory medulla of the
cockroach *Leucophaea maderae*. Cell Tissue Res..

[19] Arnold T., Korek S., Massah A., Eschstruth D., Stengl M. (2020). Candidates for photic entrainment
pathways to the circadian
clock via optic lobe neuropils in the Madeira cockroach. J. Comp. Neurol..

[20] Nässel D. R., Zandawala M. (2022). Endocrine cybernetics: neuropeptides as molecular switches
in behavioural decisions. Open Biol..

[21] Levine A. S., Jewett D. C., Kotz C. M., Olszewski P. K. (2022). Behavioral
plasticity: Role of neuropeptides in shaping feeding responses. Appetite.

[22] Hou L., Wang N., Sun T., Wang X. (2023). Neuropeptide regulations
on behavioral plasticity in social insects. Curr. Opin. Insect Sci..

[23] Turner A. J. (1984). Neuropeptide
processing enzymes. Trends Neurosci..

[24] Fricker, L. D. Neuropeptide-processing enzymes: applications for drug discovery Drug Addiction Springer New York, NY 2005 497–509 10.1208/aapsj070244 PMC2750981 16353923

[25] Habenstein J., Schmitt F., Liessem S., Ly A., Trede D., Wegener C., Predel R., Rössler W., Neupert S. (2021). Transcriptomic, peptidomic, and mass spectrometry imaging
analysis of the brain in the ant *Cataglyphis nodus*. J. Neurochem..

[26] Bolger A. M., Lohse M., Usadel B. (2014). Trimmomatic:
A flexible trimmer for
Illumina sequence data. Bioinformatics.

[27] Peng Y., Leung H. C., Yiu S. M., Lv M. J., Zhu X. G., Chin F. Y. (2013). IDBA-tran: a more
robust de novo de Bruijn graph assembler
for transcriptomes with uneven expression levels. Bioinformatics.

[28] Seppey M., Manni M., Zdobnov E. M. (2019). BUSCO: assessing
genome assembly
and annotation completeness. Methods Mol. Biol..

[29] Camacho C., Coulouris G., Avagyan V., Ma N., Papadopoulos J., Bealer K., Madden T. L. (2009). BLAST+: architecture and applications. BMC Bioinf..

[30] Derst C., Dircksen H., Meusemann K., Zhou X., Liu S., Predel R. (2016). Evolution of neuropeptides
in non-pterygote hexapods. BMC Evol. Biol..

[31] Hummon A. B., Richmond T. A., Verleyen P., Baggerman G., Huybrechts J., Ewing M. A., Vierstraete E., Rodriguez-Zas S. L., Schoofs L., Robinson G. E., Sweedler J. V. (2006). From the
genome to the proteome: uncovering peptides in the *Apis* brain. Science.

[32] Predel R., Neupert S., Garczynski S. F., Crim J. W., Brown M. R., Russell W. K., Kahnt J., Russell D. H., Nachman R. J. (2010). Neuropeptidomics
of the mosquito *Aedes aegypti*. J. Proteome Res..

[33] Zeng H., Qin Y., Du E., Wei Q., Li Y., Huang D., Wang G., Veenstra J. A., Li S., Li N. (2021). Genomics-
and Peptidomics-Based Discovery of Conserved and Novel Neuropeptides
in the American Cockroach. J. Proteome Res..

[34] Liessem S., Ragionieri L., Neupert S., Büschges A., Predel R. (2018). Transcriptomic and
Neuropeptidomic Analysis of the
Stick Insect, *Carausius morosus*. J. Proteome Res..

[35] Gasteiger E., Gattiker A., Hoogland C., Ivanyi I., Appel R. D., Bairoch A. (2003). ExPASy: The proteomics server for
in-depth protein
knowledge and analysis. Nucleic Acids Res..

[36] Veenstra J. A. (2000). Mono- and
dibasic proteolytic cleavage sites in insect neuroendocrine peptide
precursors. Arch. Insect Biochem. Physiol..

[37] Predel R. (2001). Peptidergic
neurohemal system of an insect: mass spectrometric morphology. J. Comp. Neurol..

[38] Schachtner J., Wegener C., Neupert S., Predel R. (2010). Direct peptide profiling
of brain tissue by MALDI-TOF mass spectrometry. Methods Mol. Biol..

[39] Rappsilber J., Mann M., Ishihama Y. (2007). Protocol for micropurification,
enrichment,
pre-fractionation and storage of peptides for proteomics using StageTips. Nat. Protoc..

[40] Predel R., Neupert S., Derst C., Reinhardt K., Wegener C. (2018). Neuropeptidomics of the Bed Bug *Cimex lectularius*. J. Proteome Res..

[41] Veenstra J. A. (2014). The contribution
of the genomes of a termite and a locust to our understanding of insect
neuropeptides and neurohormones. Front. Physiol..

[42] Sterkel M., Urlaub H., Rivera-Pomar R., Ons S. (2011). Functional Proteomics
of Neuropeptidome Dynamics during the Feeding Process of *Rhodnius
prolixus*. J. Prot. Res..

[43] Nelson J. M., Saunders C. J., Johnson E. C. (2021). The Intrinsic
Nutrient Sensing Adipokinetic
Hormone Producing Cells Function in Modulation of Metabolism, Activity,
and Stress. Int. J. Mol. Sci..

[44] Choi M. Y., Köhler R., Vander Meer R. K., Neupert S., Predel R. (2014). Identification
and expression of *Capa* gene in the fire ant, *Solenopsis invicta*. PLoS One.

[45] Neupert S., Derst C., Sturm S., Predel R. (2014). Identification of two
Capa cDNA transcripts and detailed peptidomic characterization of
their peptide products in *Periplaneta americana*. EuPA Open Proteomics.

[46] Eckert M., Herbert Z., Pollák E., Molnár L., Predel R. (2002). Identical cellular distribution of
all abundant neuropeptides
in the major abdominal neurohemal system of an insect (*Periplaneta
americana*). J. Comp. Neurol..

[47] Predel R., Wegener C. (2006). Biology of the CAPA
peptides in insects. Cell. Mol. Life Sci..

[48] Bläser M., Predel R. (2020). Evolution of Neuropeptide
Precursors in Polyneoptera
(Insecta). Front. Endocrinol..

[49] Redeker J., Bläser M., Neupert S., Predel R. (2017). Identification
and
distribution of products from novel tryptopyrokinin genes in the locust, *Locusta migratoria*. Biochem. Biophys.
Res. Commun..

[50] Adams M. E., Zitnan D. (1997). Identification of ecdysis-triggering hormone in the
silkworm *Bombyx mori*. Biochem.
Biophys. Res. Commun..

[51] Zitnan D., Zitnanová I., Spalovská I., Takác P., Park Y., Adams M. E. (2003). Conservation of ecdysis-triggering
hormone signalling in insects. J. Exp. Biol..

[52] Gäde G., Rinehart K. L. (1990). Primary structures
of hypertrehalosaemic neuropeptides
isolated from the *corpora cardiaca* of the cockroaches *Leucophaea maderae*, *Gromphadorhina portentosa, Blattella
germanica* and *Blatta orientalis* and of the
stick insect *Extatosoma tiaratum* assigned by tandem
fast atom bombardment mass spectrometry. Biol.
Chem. Hoppe Seyler.

[53] Schulze J., Schendzielorz T., Neupert S., Predel R., Stengl M. (2013). Neuropeptidergic
input pathways to the circadian pacemaker center of the Madeira cockroach
analysed with an improved injection technique. Eur. J. Neurosci..

[54] Roth S., Fromm B., Gäde G., Predel R. (2009). A proteomic approach
for studying insect phylogeny: CAPA peptides of ancient insect taxa
(Dictyoptera, Blattoptera) as a test case. BMC
Evol. Biol..

[55] Predel R., Neupert S., Russell W. K., Scheibner O., Nachman R. J. (2007). Corazonin in insects. Peptides.

[56] Holman G. M., Cook B. J., Nachman R. J. (1986). Primary
structure and synthesis of
a blocked myotropic neuropeptide isolated from the cockroach, *Leucophaea maderae*. Comp. Biochem.
Physiol. Part C.

[57] Holman G. M., Cook B. J., Nachman R. J. (1986). Isolation, primary
structure and
synthesis of two neuropeptides from *Leucophaea maderae:* Members of a new family of cephalomyotropins. Comp. Biochem. Physiol. Part C.

[58] Holman G. M., Cook B. J., Nachman R. J. (1987). Isolation,
primary structure and
synthesis of leucokinins V and VI: myotropic peptides of *Leucophaea
maderae*. Comp. Biochem. Physiol. Part
C.

[59] Holman G. M., Cook B. J., Nachman R. J. (1987). Isolation,
primary structure and
synthesis of Leucokinins VII and VIII: the final members of this new
family of cephalomyotropic peptides isolated from head extracts of *Leucophea maderae*. Comp. Biochem.
Physiol. Part C.

[60] Holman G. M., Cook B. J., Nachman R. J. (1986). Isolation, primary structure and
synthesis of Leucomyosuppressin, an insect neuropeptide that inhibits
spontaneous contractions of the cockroach hindgut. Comp. Biochem. Physiol. Part C.

[61] Hamasaka Y., Mohrherr C. J., Predel R., Wegener C. (2005). Chronobiological
analysis
and mass spectrometric characterization of pigment-dispersing factor
in the cockroach *Leucophaea maderae*. J. Insect Sci..

[62] Arendt A., Neupert S., Schendzielorz J., Predel R., Stengl M. (2016). The neuropeptide
SIFamide in the brain of three cockroach species. J. Comp. Neurol..

[63] Nachman R. J., Holman G. M., Haddon W. F., Ling N. (1986). Leucosulfakinin, a
sulfated insect neuropeptide with homology to gastrin and cholecystokinin. Science.

[64] Predel R., Brandt W., Kellner R., Rapus J., Nachman R. J., Gade G. (1999). Post-translational modifications of the insect sulfakinins: sulfation,
pyroglutamate-formation and O-methylation of glutamic acid. Eur. J. Biochem..

[65] Muren J. E., Nässel D. R. (1996). Isolation
of five tachykinin-related peptides from
the midgut of the cockroach *Leucophaea maderae*: existence
of N-terminally extended isoforms. Regul. Pept..

[66] Predel R., Neupert S., Roth S., Derst C., Nassel D. R. (2005). Tachykinin-related
peptide precursors in two cockroach species. FEBS J.

[67] Nässel D. R., Persson M. G., Muren J. E. (2000). Baratin, a nonamidated neurostimulating
neuropeptide, isolated from cockroach brain: distribution and actions
in the cockroach and locust nervous systems. J. Comp. Neurol..

[68] Predel R., Eckert M., Pollák E., Molnár L., Scheibner O., Neupert S. (2007). Peptidomics of identified
neurons
demonstrates a highly differentiated expression pattern of FXPRLamides
in the neuroendocrine system of an insect. J.
Comp. Neurol..

[69] Neupert S., Predel R. (2005). Mass spectrometric analysis of single identified neurons
of an insect. Biochem. Biophys. Res. Commun..

[70] Neupert S., Gundel M. (2007). Mass spectrometric
analysis of FMRFamide-like immunoreactive
neurons in the prothoracic and subesophageal ganglion of *Periplaneta
americana*. Peptides.

[71] Clark L., Agricola H. J., Lange A. B. (2006). Proctolin-like
immunoreactivity in
the central and peripheral nervous systems of the locust *Locusta
Migratoria*. Peptides.

[72] Soehler S., Neupert S., Predel R., Nichols R., Stengl M. (2007). Localization
of leucomyosuppressin in the brain and circadian clock of the cockroach
Leucophaea maderae. Cell Tissue Res..

[73] Soehler S., Stengl M., Reischig T. (2011). Circadian
pacemaker coupling by multi-peptidergic
neurons in the cockroach *Leucophaea maderae*. Cell Tissue Res..

[74] Predel R., Kellner R., Baggerman G., Steinmetzer T., Schoofs L. (2000). Identification of novel periviscerokinins
from single
neurohaemal release sites in insects MS/MS fragmentation complemented
by Edman degradation. Eur. J. Biochem..

