# Supplementary material for: Transcriptomics and Neuropeptidomics of the Cockroach Rhyparobia maderae to Characterize the Neuropeptide Landscape of the Cockroach’s Circadian Clock
Source: J Proteome Res. 2025 Oct 27;24(11):5370–89. doi: 10.1021/acs.jproteome.4c01069 (PMC12604039; doi:10.1021/acs.jproteome.4c01069)
Supplement: Supplementary file 1 [file pr4c01069_si_001.pdf]

SUPPLEMENTARY INFORMATION

Transcriptomics and neuropeptidomics of the cockroach *Rhyparobia maderae* to characterize the neuropeptide landscape of the cockroach's circadian clock

Susanne Neupert[1,2*], Sohail H. Shoaib[1,2], Julia Schendzielorz[1], Huleg Zolmon[1,2], Lars Hering[3], Monika Stengl[1,2*]

[1]University of Kassel, Institute of Biology, Department of Animal Physiology and Neuroethology, Heinrich-Plett-Str. 40, 34132 Kassel, Germany

[2]University of Kassel, Graduate School: Multiscale clocks, Heinrich-Plett-Str. 40, 34132 Kassel, Germany

[3]University of Kassel, Institute of Biology, Department of Zoology, Heinrich-Plett-Str. 40, 34132 Kassel, Germany

Table of contents:

Supplementary Information S1: Schematic overview of the nervous system of *Rhyparobia maderae* showing tissues that were analyzed in this study by mass spectrometry **S-1**

Supplementary Information S2: Prepropeptide proteins for neuropeptides, neuropeptide-like molecules, and protein hormones of *Rhyparobia maderae´s* brain **S-2 – S-8**

Supplementary Information S3: Multiple sequence alignment of *R. maderae fliktin* **(A)** with previously described novel *P. americana* neuropeptide PaOGS36577 and the first identified *fliktin* precursor from *C. nodus*, and **(B)** only with PaOGS36577 **S-9 – S-10**

Supplementary Information S4: Quadrupole Orbitrap MS^2 spectra generated by *R. maderae* brain tissue extract analysis and MALDI-TOF/TOF MS **S-11 - S209**

Supplementary Information S5: Multiple sequence alignment of baratin and NVP-like peptides from *P. americana* and *C. morosus*, and detection of NVP-like peptides in the AME by mass matches **S-210**

Supplementary Information S1: Schematic overview of the nervous system of *Rhyparobia maderae* showing tissues that were analyzed in this study by mass spectrometry. AG, abdominal ganglion; aPSO, abdominal perisympathetic organ; CA, *corpora allata*; CCgl, glandular *corpora cardiaca*; CCnh, neurohemal *corpora cardiaca* FG, frontal ganglion; GNG, gnathal ganglion; RCC, retrocerebral complex; tPSO, thoracic perisympathetic organ; TG, thoracic ganglion

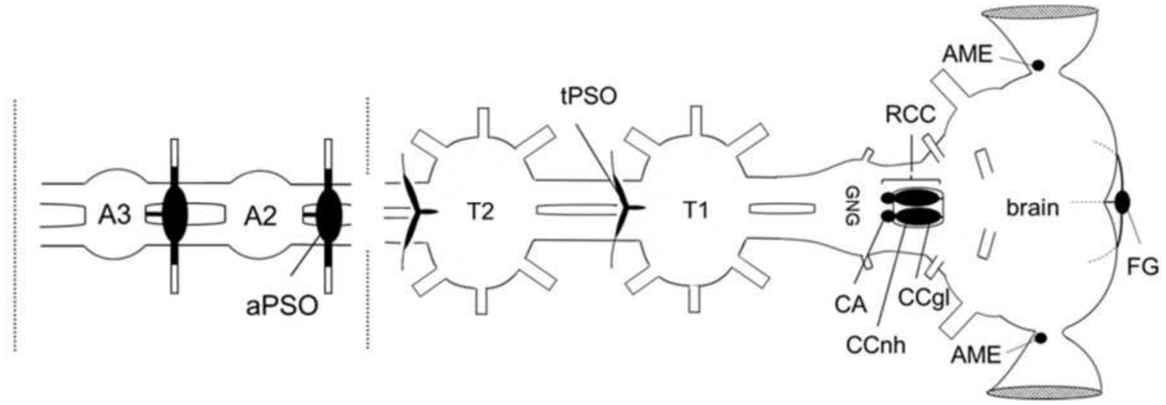

Supplementary Information S2. Alphabetic list of neuropeptide, neuropeptide-like peptide and protein hormone precursor genes obtained in *R. maderae* transcriptome. Signal peptides are predicted by SignalP 6.0 software and marked in italic/bold and predicted cleavage sides in red (including red letter). Incomplete sequences are marked with '…'. Peptide sequence confirmation by mass spectrometry focused on products encoded by neuropeptide and neuropeptide-like peptide genes. Putative bioactive molecules encoded by peptide hormone genes are not included in this peptidomic study. Peptide sequences confirmed by mass spectrometry fragmentation analysis are highlighted in yellow. Peptide mass matches are highlighted in light grey. Post-translational C-terminal glycine modifications (amidation) are shown in green and disulfide bridges in bold/blue. Truncated peptides are underlined.

>Adipokinetic hormone-1 (AKH-1)
MNQLVKVLVVVMAVALVLCEAQVNFSPGWTGKRSAVQDGPCKASTDSLMYIYKLVQNEAQKILECEKFSSN-

>Adipokinetic hormone-2 (AKH-2)
MNQLVKVLVVVMAVALVLCEAQVNFSPGWTGKRSSVQDGPCKASTDSLMYIYKLVQNEAQKILECEKFSSN-

>Agatoxin-like peptide-1 (ALP-1)
MRTHLLVLACGLLLLGQFVWPTVATPYLDDRDEGLEDYNENSLERLVQYPAQKRACVRRGGNCDHRPKDCCYNSS
CRCNLWGANCRCQRMGLFQKWGK-

>Agatoxin-like peptide-2
…EGLEDYNENSLERLVQYPAQKRSSLIYLFRRACVRRGGNCDHRPKDCCYNSSCRCNLWGANCRCQRMGLFQKWG
K-

>AKH/corazonin-like hormone precursor (ACP)
MMFRSACERALWSMLLLLTILNCLSLGQVTFSRDWNAGKRSGSPDAQCNAVIKSADEICRVMVEEFRQLAACETK
YLLRIQREFDDKQTDMYLEGQDGR-

>Allatotropin (AT)
MRQSLTVYSLIAVAAIVILVLLGSVSAGSYQNSRNKPRTIRGFKNVALSTARGFGKRDGALEYLTGNANNAAEQN
ADRMPESLPVEWFVEELRTNPELARIIVHKFVDADQDGELSAEELLRPMY-

>Allatostatin-A (AST-A)
MRGPRTSFTLPGALLLALLSLSPSALGTPTEPSGVHEESPAGGGVELLPHPEELTASDSSDLEFVKRLYDFGLGK
RAYSYVSEYKRLPVYNFGLGKRSRMYDFGLGKRDGRMYSFGLGKRDYDYYGEEDEDQQSSGDDEIQESDMGDLVD
KRDRMYSFGLGKRARPYSFGLGKRSPNGAQRLYGFGLGKRGGSLYSFGLGKRGDGRLYAFGLGKRPVSSGRSAGS
RFNFGLGKRSDDVDLRELEEKFAEEEKRYPQEHRFAFGLGKREVEPSELEAVRNEEKDNASIHEKRNSTNDLHSG
ERVKRSLHYPFGIRKQESSYDLNSASSLNSEENDDITPEEFSRMVRRPFSFGLGKRIPMYDFGIGKRSDR-

>Allatostatin-B/Myoinhibitory peptide (Ast-B/MIP)
MQYAILTGAMVWLLALVSPSSQGDPPAPQVTSGDAQETPTQVQGPDEDKRGWRDLQGGWGKRGWQDLQGGWGKRG
WQDLQGGWGKRGWQDLQGGWGKRGWQDLQGGWGKRGWNDLQSGWGKRGWQDLQGGWGKRGWQDLQGGWGKRAWDE
LRPMWGKRSWDKFHGGWGKRESDFDAEGTNMDDNFADDLEDEDGEDSKRAWSSLKSGWGKRAADWANFRGSWGKR
DPGWNNLKGLWGKRGDSNWSRLSAAWGKRSIAGDGMGKDEDGRSPSSSED-

>Allatostatin-CC (Ast-CC)
MGHRHLQHHQHCTMLHPTPFPNTGVNTLLWLLLLVCITLLTATEGTDVTSSAEPHKLSKRSATEGTMGAASDYPD
YQTGVRYDEYPVVVPKRTALLLDRIMVALQKAVDDEKGRSSYTPDMVENKMDLQRRGQQKGRVYWRCYFNAVTCF
KKK-

>Allatostatin-CCC (AST-CCC)
MSAVTTTKLMLVMLIAMLTLSWAVGKTLGQPGDKERLLNELDLVDDDGSVETALINYLFAKQVVNRLRSQMDVSD
LQRKRSYWKQCAFNAVSCFGK-

>Arginine-vasopressin-like peptide/Inotocin
MLLRTRYSIEFVVVFCFSVCSACLITNCPKGGKRSVPQSQDSFAIKQCARCGPANLGHCYGPAVCCGPQIGCLIG
SPDTKRCLSEAASLVPCTVPSGPPCGEGNFSGKCTANGVCCNRESCHIDLSCRITVNELTEGNPELTNTLYNLYH
TISSSYQEEIPKLALSPPAIEEK-

>Calcitonin_B
…DNSGLGKRCLNTGDDSCGNGYIPGATNDNDYYGKPGGLGKRHSLRHLTSQHLRSAWIRCLNTGDDSCANGFIPG
ATTDNDYINSGSGPGKRCLSIGDKGCADGHIPGGHHPGRG-

>Calcitonin-like diuretic hormone-31 (DH-31)
MNSHAALLTTALLVGAAIMLSVAHASESVPLSSNNHRNSYMSEMDSEPDSEYVLEMLARLGQSIIRANDLENSKR
GLDLGLSRGFSGSQAAKHLMGLAAANYAGGPGRRRRSSDESS-

>Corticotropin-releasing-factor-like-diuretic hormone-46 (CRF-DH-46)
MMVAVVPTLLLAALVSCSMAYYEPPLLEALAAPSPDHETSTYLLPRLSAKYRTHGDWESAPDPRFYVLTELERET
SQAARRVKRTGTGPSLSIVNPLDVLRQRLLLEIARRRMRETQNQIQANRNILESIGKREVSVSQQRSLDQDIDDM
ETDLLLSSTEAENKSAAQASSDNNRSPESSDWSTASKSRWNNEYTSQHHS-

>CAPA-peptides/Periviscerokinin (PVK)
MNEYLVCCVTVGLFLLVSTVCCDGEDVGSTSIENKNRRGSSGLISMPRVGRGNIPWNFQGTDEDLSKSRRGSSGL
IPFGRTGRGNIPWTFQISDEEVSPSTTDIKSRRGSSGLIPFGRTGRGNIPWTVQISGVDYDKSKRGSSGMIPFPR
VGRSDLLANLAIPDDYLEMDDSLVGVPDKRFGETSGETKGMWFGPRLGKRSRRGTEFPWAVVTVKVPEFPSNRRY
GGFTPRLGRESSEIEEEGLLEVEETQKTGRPTSGRKTHSFN-

>CCHamide-1
MALQRSSSILVAAMATIFVLMVQIDQLSAKRGCSSFGHSCFGGHGKRTDEDVLLLPGSDSEQQRLDLPLPSGSLE
GDDMMQAGPEFGSRSSPSQSAALSSVISPPHPYNLSPFLRQWLQSYRRSAGDIEVK-

>CCHamide-2
MMIWTSATRTSVLGFASRIAVILFIFGLAECAAGSCLSYGHSCWGAHGKRSGNSAKPDVLPVPEDSNDGIPSSDD
TRWFLSKLVRPADSGSRDMWQRPDTRHKELQWKSSPIEEEVDSPAILRENDEMPRSRGVQLEASDESAGILMPLN
GEYPEENQDGEVVFMTEEQPIRRVPKKLRVFKIMNHPGRKLDK-

>CNMamide-A
MTALDSLPLPAAIQAKLFRLSEGVNREEMNKRGSYMSLCHFKICNMGRKRNLRWNPWIRR-

>CNMamide-B
MPISRTIVNRTVLLWALVLIATLSCGVQAAPEPFHRQQGDPAIVIPPPGINDLEDLGINVNIDDPKHREEILLAI
LQMYKNEIQKQGAEDDQNVIDSGAMTALDSLPLPAAIQAKLFRLSEGVNREEMNKRGSYQPSLCYFKICNMGRKR
NIR-

>Corazonin (Crz)
MYTHSSSHRRRSQKFPGIILILSCLTGAILAQTFQYSRGWTNGRKRGGPTSTLIVPPSGSGSGVYQNLDDANPCT
QLQKIRFLLGARNPQQFFFPCDTWRDVPETSDNVSERFKRGTASVEEIPQQNPSDQ-

>Crustacean cardioactive peptide (CCAP)

MQMYHVLLGCSLATLLVILHLPLISCDDVIVQKRQVDPAEMERLLDPKRKRPFCNAFTGCGKKRSDESLGTLVEM
NSEPAVEELSRQILSEAKLWEAIQEARAELLRRRQEQASQYGNAVERSLPLPLAGYRRKRSAATGTDANSNMPRS
ELQEQMAKPWSR-

>CCRFamide
MIRLKATLLLITMLELGSFLQMDASSPDNCVPRELRCELLCHVVELGLQCAKCRSRAPVRFGKRVPETAVQTYNP
NCCGHLFNSLLRKAAVQNNL-

>Elevenin
MSGGCAQVLPSLLTTAMLVLILTTIQAVPEPIDCRRMVFAPKCRGIAAKRAFQPISGPGYILDTDRKMDGLEEVL
GLYVTPQPLSIPQQGESRVQQTRGYPSRTAWNNAPDQQGLKTDILYDWYLSNKKRTRESDLAYDY-

>extended FMRFamides
MFPVIFILVCVTASATAYPTDNPISEPPNIVLSSPDDIPLDLSAQDDTECESEEESPPYPIKRAEEQEQSPPARR
CRNQNFIRLGRANFDAVSELETPDDIKSNFARFVRGGKSNDNFIRLGRAGKGSNFIRLGRDKSDNFIRFGRGRTD
SFIRFGRSRADNFIRLGRDKSDNFVRFGRGKPDNFIRFGRGKEDNFIRFGRGLRDNFLRFGRDQVSSIDDEEEDT
HPRMTRGGKSDSNFIRFGRSKPSNFIRLGRANEDDLIQREERSKGNNNFVRFGRNFYDDDDFVRTGRSGNSNDLR
RGKLTDRNFIRLGRSGGDYDFHDTQEYGSDDETSLRSGRSNNDGNFIRLGKSEENHLLRFGRDVEQIDDMPVLSS
TESNQSDNGNNTDQHGYQSSRNKRAVSYTATEESSDFPVPIPNSEYGGRDTKTPFGYYSPLTSGIPNYILGPELA
VLAPLSNGAESVSKRSKGRDHNRNYIRLG-

>Pea FERLQ-like peptide
MKLTTCLIVLAVCLLVSVLGAPVKKEHPEMNKEQELEELLKETAIILEEPLVQAKDESLPHFPRTEVKVNDCPTC
VADSFRWERK-

>Fliktin
MGSWKAVLCCALVLVSFALGQDEQPRDSLRTAIEAVSRRQRDLAAAGPNYYRGGLSQYRYQDNEARPGEELAFLA
TPREFTGDGQPENIGYGYQKTIASPSGMFSPQTPLDGESPANLEHAAKNKIMENMLVEYLEDELKGEPDDDEDDA
YYRKRSAFRERADDERRRYEAMKKREFRSFAPSAFRERTHSSSVVDDVEEKKRKILADALVRKMEEEEEEERRDR
ERGRIDNDEEAEEEYLDVLRNVWDRYRKDNPHVIDIEDISEGDVGEILNYLGNTGLLDDEDVEGIKEEASKREYD
FNTHNIAMSGFGGGGFKKRWNQRLDGDENQKGNFLYSLKFVSPAINREAIESLKDDDDLDLPDERDEDVLRLTSN
VRREPDPWFPAFERGEAPEELFGNPSEEEYQRLLLAQQNEHQAPSRKRIASLRSHHSASSLPDVFFTPEKKYLYD
TAIMKKRYPVTKRSSNFYTSPPLLHHKNFAFTDNYETRRKKDATGTSVATTDPKVARELNQIFSSPVAEHSNEAH
SKDSAHNMDKPTADSVHVATTHSPAITASTTALSKTKENETDKINRDNTTTQHRSGSEEETVEQPVTMSRSEAPL
DIKKKSINWSDYFGIDRRRKKTSSSPPDNHPTNDEWLLNQYYKTFAMSTNPGKKRSIVYPHDAAKVKSTALQQPF
DTRVFDTDIFARTTQHEASAVKKSVQTSNLDKNEEARLNNMDTKLRNIEDEIVNEAVKFTGAHEGSTDSKEIQEV
KDKVMARLAAAYSLEKMRQALGEFRSSLMAQKMSKYNPENRQTGNVDEKKKKRVAVKKEKAEEKKSEDKKKRGDG
HAESDEDMGEFLEGPVIVQPVSEGDMGRQEINSEDDITCPVLDQIISKCRTANNIVGDHSQLFLPLCSLHQICYL
CGPELGAPSPAACDLMFITEAEMLCRNDEDCRLAARRNVAILRKSQDHIEEGQCWRSPCIAHYFLQSPLPAPLSA
SSSR-

>Hansolin
MLWPLLILGYVVLVTSRPPPSIAEDILDDLTWRELPPEMLEQASQWIPLYNNPESPGPGKIGNNRPSKRALSVLS
RWKPFSMGFSSLVGRYPPRAPLLTMMPELDFVSAETRGTLRPIGQPLRWGRR-

>ITG-like peptide
MKSLVSGTLALLAVLSGVSAWGGLFNRFSPEMLSNLGYGGHGSYRAQPFLQRLSPAEVFQELQEDEEPCYGKRCT
ANEHCCPGSVCVDVDGIVGSCLFAYGLKQGELCRRDNDCETGLLCTETGGEGRTCQPPSSNKKQYSEDCTMSSEC
DITKGLCCQLQRRHRQAPRKVCSYFKDPLICIGPVATDQVKDDNIEHTAGEKRLTGKSGSVNAFTHLRRRK-

>Ion transport peptide (ITP)
MSMEHQQMIRILSCCFLISIVLTTLVVVPASGRVLGHSVAKRSFFELQCKGVFDKTIFARLDRICEDCYNLFREP
QLHTLCRSNCFSTPYFNGCLEALLLDKEKENFSQMVEYLGKK-

>Kinin
MRLLLLVAVLTSACSQAVAWSPTYRAPGNDGIAVDDMEISRPWSQALSVHPDIYRTLPELAHLYSDTDGTEEEAG
ADSSGLTQGLHRGDNEPWSRTDIGADYELQTGEDILCAQCGYDSSSSTLLGSSPPDQNDEMKPLIRPSQIKRKQS
NRSSQHGSRYKRDAAAMETEEEMQKKDQGFNSWGGKRALAFNSWGGKRNPAFSVLGNVRRRAFTSTGNRRSPAFN
SWGGKRSYSFLGDNTNPILNSWSGRSNPTFSLSSSEENPAFTVLGDKRQSSFHSWGGKRDASFHSWGGKRDPSLS
ILGNHQEPAFKIIGGGITEPPFSIIDTKEPAFKILGSHDFPAFAILNDKYEPDFSILNQKRGSGFSSWGGKRDPG
FSSWGGKRDAAFNSWGGKRDPAFNSWGGKRDPAFNSWSGKRDPAFNSWGGKREAMFSSWGGKRDEENSDLSKRSP
KFSSWGGKRVADGDEKRSFSSWGGKRDLSVSPKRVFSSWGGKRTAATQTEEEIYKHGNSTTTKEITDEDREKEKE
TDDENKEKEETPTVEVNESEKDGRRHVGTQTSLDLQKLLEEFGKKMLLDEDKEKSESKEGEDKTEDQVETSEKAD
INNGHEEIGLEDKGAGTINTEDKSIGTSDFLAINKRNTAKSGTRVSKAMFNPWGGKRSFHTPSLFTVLARMSRGI
ERSDGFLSDLLYKRGSSRHSSLGSKKWGQASAGAVFSSWGGKRAEKGSDLRKLSPQNMGRQYRRGADFYSWGGKR
-

>Myosuppressin (MS)
MKYVCIVLIGVLSLLLACAPRRALAVPPPQCSPNILEDVPPRVRKVCAALSTIYELSNAMEAYLDDKVVRENTPL
MDTGVKRQDVDHVFLRFGRRR-

>Natalisin (Nat)/WAARamide
MSPSLVVVALAVTCGFGAVVRSSQELTVKNKTGENEVKHRVTRSDIRAALGDKLAPGFWPARGRRSNSEESPPPF
WANRGRSLRLTDDEKSLLEELMPFWKEGSSTVHNLRLRREEPLYADEPHWILLARRDEQEDDGEEDQGSGVITQD
DPFWVARGRRRSEMPFQSRLASSDDDTFLAARGRRSSKRGLRELISAEEPFWAARGRRSESLEEPFWAARGKKGN
YKALEDFRSRRGLLSSSEEPFWAARGKKSGSPRRSFLNSLSSEEPFWAARGRRGLLESLSAEEPFWAARGKKDTP
PNNADALSQMRTREAGHGNDPWWPVRGKRVADELNPEDETFWRTLDSKIKNNSRTS-

>long neuropeptide F-1a (NPF-1a)
MQNSLCWLLVLGCALTLVPRISTKSTDPEQLAAMADTLRYLQELDRYYSQVARPRFGKRADVHPLPEQESVPDES
SERLWRRFASRR-

>long neuropeptide F-1b (NPF-1b)
MADTLRYLQELDRYYSQVARPSPRSESGRPHHLSKMENALKMLQLQELDKFYSSRTRPRFGKRADVHPLPEQESV
PDESSERLWRRFASRR-

> long neuropeptide F-2(NPF-2)
MQSPMNLMVLACLCGVVICMAMPCYSDPSASAEIASRPTRPKVFTSPDQLRTYLQELGNYYAIEGRPRFGKRLAT
AGFRSGLAPVAVSSGVAEPSNYYTRYPAQSKARNDVYQMLFPYEE-

>Neuropeptide-like precursor1 (NPLP1)
MWRAILLLVAALATLPQTLCDEDKRSYASLARNGDLPFFARKEWNKKLHPMVATGKRYVGALAKTGGLPYGKRSD
DFEDEDLLRELLKDIEEKRSLASLARSGSLQGKRSVEALARAGYLPVAKPPQESEEYPHDSSENSEEAKRNIGAL
ARNGYMKRDGDELDELLEDLYEKRNIASLMRNGYSPFAQQGKRYLGSLMRNQRNIGAMARNWHLPDHLKFGKRQD
DDDAAEEEDEEEDLEDVAKRYVAALLRHGGLPLGGSSAADISEDKRHIGSLAAKGTFQVHKKSVRSTGSEDSAYN
STAKSDESKRSKRQATYLANSDEFPMPVLQNSDLFDYEDLANILNGEAAPEKRFLGSVARSGWFRDNGNRMLHTS
TMTKRHIGSLARLGWLPAFRSSRYSRSGRASPAPPDDDEEHSRSAHVPYH-

>Carausius Neuropeptide-like precursor1 (CNPLP1)
MAGHLLLLFALLRLGTAIPNLHDNMVPTDEEILRTILEQEKNSRTAEKGQPEVEDSLGLPTDEDSYNELLLALGL
PGVSGTHHFVSSSFPEVDARGFHESVFDGVGDYYPPWGRHKRDPLGINSRGFHDDVFNRDFGTFHTVKRSNTNQV
NSDKVEEILRKMMTSQRKKRDTNENEEEMQKETQESQSIKVSRDVSNSDVEVAAEKRRPEMDSSGFHGDTFHGGF
GDFWPMKKNSAQNSSAHSNYWTIHKRRLGMGPSGFHGDTFTSGFGDFSTMKRTGTSTDFDVYKTEDNKRKPEMGS

SGFYGDTFSNGFGDFWTMKRAGNVLNAEGRLEHKRRPEMDSSGFHGDTFNSGFGDFWTMKKRKPEMDSSGFHGDT
FNSGFGDFWTMKKRKPEMDSSGFHGDTFNGGFGDFWTMKKRRPEMDSSGFHGDTFNSGFGDFWPMRKRRPEMDSS
GFHGDTFRNGFGDFWTMKKRRPEMDSSGFHGDTFNSGFGDFWTMKKRKPEMDSSGFHGDTFNGGFGDFWTMKKRR
PEMDSSGFHGDTFNSGFGDFWTMKKRRPEMDSSGFHGDTFRNGFGDFWTMKKRRPEMDSSGFHGDTFRNGFGDFW
TMKKRSPEMESSGCHGDTFPTPAVCSLVQGASN-

>NVP-like-peptide-A
MMTGVVLVALLVVAVSGLPSTLLEDTKQAAQIASNSDPKSSTKDEKQEQLPTALPISISSTSKSWDPHGRSNVQY
PGGSGAQFGSDQQAEQGRGKTLSQYEKGYQYGVGKAALDKHVENALLKSELYGDPSAVNQYRYYGGASERKRNQH
LTYQPPSKRSYRPEIPFVMPPDDLTNSRSRLKRDLELDPEDFLTVLSLWEAEHRAKSENPVVDPSWYSYYGMESP
DQFQDEEDESGEEEEDPSQLEGGWLDGPVVHPSTSPHRYRLERRGGYYYPMQYPMYPAQKRDNSQWGGFAKDKRF
MVTRKRQVNTARDEAQTLAQLLSHQYRDPGVPLYRRVVL-

>NVP-like peptide-B
MMTGVVLVALLVVAVSGLPSTLLEDTKQAAQIASNSDPKSAVNQDRYYGGASERKRNQHLTYQPPSKRSYRPEIP
FVMPPDDLTNSRSRLKRDLELDPEDFLTVLSLWEAEHRAKSETPVVDPSWYSYYGMESPDQFQDEEDESGEEEEE
PSQLEGGWLDGPVVHPSTSPHRYRLERRGGYYYPMQYPMYPAPKRDNSQWGGFAKDKRFMVTRKRQVNTARDEAQ
TLAQLLSHQYRDPGVPLYRRVVL-

>Orcokinin-A (Orc-A)
MKALALLVVALAAVSVPSSANPVQSDALRESAFRDYRGDSADEDSVVRHLDTIGGGHLLRELEGLSHFPRRTRSG
LDSLSGATFGGNKRLDSLSGATFGGQKRNFDEIDRSGFNSFVKRNFDEIDRSGFDSFVKRNFDEIDRVGFGSFVK
RNAPLFLARYYDKENH-

>Orcokinin_B
…MKNLKKYLSHGHHSRLDSIGGGNIVRTLNSFKSDFLKNIDAGSLDSIGGGHVVDRSVDPLGGGHIVGRGIRNGH
RALDSIGGGNIVGRNLDGIGGGNLIRALDSIGGGHLVGRSVDPLGGGHIVRALDSIGGGNLVGRNLDGIGGGNIV
GRSIDSLGGGNLVDRNLDGIGGGHLVGRGIDSL…

>Pigment dispersing factor (PDF)
MKHIGAVLLFLYLLRMAVTSPAIQLDDDRYLDKEFQTNAVSARELSNWIMQMLMHKGEPTMCTHKRNSEIINSLL
GLPKVLNDAGRK-

>Pyrokinin (PK)/FXPRLamides
MRSRFSARTHNFILLFLTAVLSSTHGFRISSSLFRETEPFEDAILVGLDGNRDALLVKRGDPAETAGMWFGPRLG
RRDKRSLDEIDLGDSSKDEEIFEILKETPWAIIPLRGSKRQTSFTPRLGRDSVEDDDVDLEQRSPPFAPRLGRRL
VSFRPRLGRDHLQQQDIYSPRLGRSIPSSPQSQDHKEQTKH-

>Proctolin
MMCCRQMLLVALVLIALYAATEARYLPTRSQDDRLDRLRELLRDLLESEIEKTSVNNFERRMVYKREAPAALVNE
QAPLLAAQQ-

>RFLamide
MHPTLLGTILGATLMLQSLIHSVAMETVNSGNMLAAPESESDQNSLNAASNAKWDPEMELLTSYLEQILRDSDIW
DVPSGPLLYVEDTPPAYTGEVSEDDDGLPVPWKRSRYYRRYPWKRQNGRHRAQYSDASRYMCNPTREDVFQLLVA
LHEAREGNTRRTVNFCNRKRPASTIFTNIRFLGRRK-

>RYamide
MASPSTAVIIITCSLVLIACSAQQFYPGGRYGKRGSSTFWSGSRYGRSGVGAGRRQQAGGGTPVEVAARNDRFFI
GSRYGKRADESISMPETSINEVAGLADVLPSDEEGNSHMTCLYTGITNLYRCYNKKENASEE-

>short neuropeptide F (sNPF)
MQSFLTVKCVTVALCLLIFASEFVSSAPSYSDYESIRDLYELLLQKEALDNRMQQQGVHEIVRKANRSPSLRLRF
GRRADPLLSGSPFSEHSSAESSVVEN-

>SIFamide
MQKPSVATCLLLLTVLLLAELTAATYRKPPFNGSIFGKRGNVVAEYENTGKALSALCEIASEACSAWFPSSDNN-

>SMYamide
MDTMKITMIFSIVLMMVLLATCNPGPPFRRLPFNGSMYGKRTSSVLPPDYDSSNKAFSSLCEMATEVCTTWFPQQ
VENN-

>Sulfakinin (SK)
MSCGNNMAATLLVTLGIYIVLHQHHVQAAPSSSDVVPAGSSNLEGAGQRTRPRPFLQTSPRTSQYLRARLMPVES
SDVLNDFVIDDDAIDFSKRQSDDYGHMRFGKREQFEDYGHMRFGRSLD-

>Tachykinin-related peptide (TK)
MAFPCPRSRVGALILVTLSLIAVVLCAPEESPKRAPSGFLGVRGKKDNVIPEFNSDEFNEAADKRAPAMGFQGVR
GKKDQDEDLGYDKRGPSMGFHGMRGKKEQQDFLRDLVDKRGPNMGFMGMRGKKDPLDFDYYEKRAPSMGFQGMRG
KKDQWEEDADMYKRAPSMGFQGMRGKKDYFDDEDEYFKRMGFMGMRGKKEGDFEVDDYPEDGLWSEDGEGEEMDK
RAPAAGFFGMRGKKVPASGFFGMRGKKGPSVGFFAMRGKKAPSAGFMGMRGKKAPSGFMGMRGKREDLDGEDLDS
LLQYLDSAYQHGRDKRNGERAPGSKKAPSGFLGTRGKKDWPTQQGAEAGTEPDIHTSLSD-

>Tressin
MARVTQLTVLTMGLVLWCVCTWSVALSCNSCGSECQSACGTRNFRTCCFNYLRKRSAGGEEGDGPGLRLELLVLP
ELAARYWEPPQQTQVKSTDPESSETVPGRMQLVYNA-

Remaining protein hormones:

>Bursicon-alpha
…MLVVMCIAVVGGVDECQVTPVIHVLQYPGCVPKPIPSFACTGRCSSYLQVYYLWNVCVGHVRQWRRVQ-

>Eclosion hormone-1 (EH-1)
MEGRKASCFLVLALLIIFATMVSSESTSYSINVCIRNCAQCKKMFGAYFEGQLCADACVKFKGKMIPDCEDVASI
APFLNKFE-

>Eclosion hormone-2 (EH-2)
MLYHQPILYLIIFITISTQCGLASKLGVCITNCGQCKQMYGPYFQGQVCAEACLSSEGRLQPDCNNPNTLVAFLK
RLY-

>Glycoprotein hormone alpha
MFAVSWRLQCCSLVLVFVLILVVVSRTSARDAWERPGCHKVGHTRKISIPDCVEFHITTNACRGFCESWAVPSAL
DTLRVNPHQAITSVGQCCNIMDTEDVEVRVMCLDGTRDLVFKSAKSCSCYHCKKD-

>Glycoprotein hormone beta
MTLPFNRLRPCLCLVFVALWVGSECTSLQESTLSSTLECHRRVYAYKVSKTDSSGRMCWDVISVMSCWGRCDSNE
ISDWRFPYKRSYHPVCLHDNRAVSEVTLKNCEEGVEPGTDRYEYLEALSCRCMVCKSSEASCEGLRYRGQRSGPF
LSGGR-

> IDL-containing peptide-A
MVRATNPRHVVMLVVGLTALCVALPQTVMAIDLSRLYGHLNAKRNDHHPAGYAKISPPALSIKSESHFFMDTVER
EDSSESDACHPYEPFKCPGDGICISIQYLCDGAPDCPDGYDEDSRLCTAAKRPPVEETASFLQSLLASHGPNYLE
KLFGSKARDALAPLGGVEKVAIALSESQTIEDFGAALHLMRSDLEHLRSVFMAVENGDLGMLKSLGIKDSELGDV
KFFLEKLVNTGFLD-

> IDL-containing peptide-B
MVRATNPRHVVMLVVGLTALCVALPQTVMAIDLSRLYGHLNAKRNGDACHPYEPFKCPGDGICISIQYLCDGAPD
CPDGYDEDSRLCTAAKRPPVEETASFLQSLLASHGPNYLEKLFGSKARDALAPLGGVEKVAIALSESQTIEDFGA
ALHLMRSDLEHLRSVFMAVENGDLGMLKSLGIKDSELGDVKFFLEKLVNTGFLD-

>Insulin-like peptide-1 (ILP1)
MNTLRLWVQVMLLGVLCAWTLPSNSSILDHSIKKRETEHRYCGDYLISALKLLCNSTYYSPHDDDYYDEELKRST
VILKKRVPDADDTLWTEIPAKLQFPFRSRAMANSLTNKYFRRQTRTVQVVDECCIYKGCYARELMEYCAAR-

>Insulin-like peptide-2 (ILP2)
MSVGRLRNTIDKMWRLCLQFVAVAALCLCTLAQAQSDLFQFSEKRNTHKYCGRNLANMLQLVCNGNYYPMFKKSS
QDMDDVNDSGFWIQPSTMEDQQLQFPFRSRSSASALVPGTFRRRTRGVYDECCRKSCTIQEMASYCGGR-

>Insulin-like peptide-3 (ILP3)
…MQLCGTELANKLAEICSSYGYNDPFSHALRFESPPLDMRAPLRLRVRRGVADECCKTGCSLDTMEQYCSSPLTP
AERARFMQQLQDDRMNHIPQDDAAIGAELTASAEMRRPGNTKEHKNDLVSKVRGPHDKKLRRGNNRCRCRRRRRR
GKGDMEELDHHQNQIPPVIGTINPSFYGTPIILSSRVKKDETQDFKRN-

>Insulin-like peptide-4 (ILP4)
MMWRFCLCMMVLCAMCACALPESSSTSLQMVRKRETSHRYCGRRLVSTLRLVCGGNYYVDEDKRSTDDAENKSAR
DIEWMPQSPDFALEAEFPFRSRPLANSLGNKHFRRHTRSGTIVDECCIYKGCTISELTEYCQAR-

>Insulin-like peptide-5 (ILP5)
MWRLCIRLILLLATCCTLSQSQSDMYQFLEKRESKRYCGRYLVEVLQMVCNGRYNGITTSSTNYNHKKSVEVPET
DDDLWSQLQSTEEAFKFPFRSRSSAHRIFKRHPSGIAYECCISKGCTLLELRSYCSPSAS-

>Insulin-like peptide-6 (ILP6)
MWRLCIRLILLLATCCTLSQSQSDMYQFLEKRESKRYCGSYLVDVMRMICNGRYNGLNTSNTYNNHKKSVEVPET
DDDLWSQLQSTEEAFKFPFRSRSSAHRIFKRHPSGIAYECCISKGCTLLELRSYCSPSAS-

>Neuroparsin
…MHWMFLSHPGLLLDRKGSTDRLCNMDPENCEHGVVRDFCGRPVCAKGPGESCGGTNDVRGKCGDGMHCTCSKCT
GCSLSTYQCYNRHDHLIECLLSA-

>Prothoracicotropic hormone (PTTH)
MATEFCFRTEGSKMIIIVCVILLASNMWLSGAEGARYPVFWGPQTAPVFENEFEDDACLDGRCMPKNGHRIAPQD
MAFLMNYFRHDNENLAPEEAVGKRDPTSASFSADSVLFRDANPTPCSCFSDSIPRPRDLGPGVYPRYLSDVECSS
TSCGNPLYRCHSLNHTIFVLKMKNPNREDESTDMEAVDLPRSLSGKWKFEGVNITVACICQRHYSHAT-

>Invertebrate parathyroid hormone
MNTRLVLIFSILLVLALLAIPQAHGRPYRPKRGSDQRLAELETIMALRNLAGLIHTVPVGFGQVDPAKIGRRRRR
SAELLLQELLNSPAHEEASDADAILPDNTESDDELRDAVEPHRQQQWLPNWSPRVQV-

Supplementary Information S3: Multiple sequence alignment of *R. maderae fliktin* (PQ049281) with previously described novel *P. americana* neuropeptide PaOGS36577[33] and the first identified *fliktin* precursor, *C. nodus fliktin*[25]. **(A)** Alignment of *R. maderae fliktin* with PaOGS36577 shows highly structural similarities in amino acid sequences (asterisks). The novel neuropeptide obtained in *P. americana* by mass spectrometry is marked in blue. MS-identified *R. maderae* Fliktin peptides are marked in yellow (in this study). Cleavage sites are highlighted in red. **(B)** The alignment of *C. nodus fliktin* with *R. maderae fliktin* and *P. americana* PaOGS36577 revealed amino acid similarities (highlighted by asterisks), indicating that both *R. maderae fliktin* and *P. americana* PaOGS36577 transcripts may be orthologous genes.

(A)

```
PaOGS36577      MHSWTSALLCLLVLLSLSQGQDEQPSDSLRTAIEAVSRRQRDLATAGSNYYSGGLSQYRY
Rm_fliktin      MGSWKAVLCCALVLVSFALGQDEQPRDSLRTAIEAVSRRQRDLAAAGPNYYRGGLSQYRY
* **.:.* * ***:*:: ****** ******************:**.*** ********

PaOGS36577      PERDSAVPEELAFLASPRDFTGDGQPENIGYGYQKTIASPTGIFPPQAPLENDVEP--SH
Rm_fliktin      QDNEARPGEELAFLATPREFTGDGQPENIGYGYQKTIASPSGMFSPQTPLDGESPANLEH
:.::   *******:**:*********************:*:*.**:**:.:   .   .*

PaOGS36577      PSNSKVLEKILLEYLEDEMKAEDEDDDDEYLHRGSNDKRSAFRERAGEERGKLEAIKKR-
Rm_fliktin      AAKNKIMENMLVEYLEDELKGEPDDDEDDAYYR----KRSAFRERADDERRRYEAMKKRE
.::.*::*::*:******:*.* :**:*:   :*     *********.:** : **:***

PaOGS36577      -GRFGSTAFRERAHDR-LVDDVEQRKRKLFTEALVRKMEEEEDDERRERERGRLGDEDEN
Rm_fliktin      FRSFAPSAFRERTHSSSVVDDVEEKKRKILADALVRKMEEEEEEERRDRERGRIDNDEEA
*..:*****:*.   :*****::***:::**********::***:*****:.:::*

PaOGS36577      EEDYLDVLRSVWEKYRRDNPNVIDIEDISEGDVSEILNYLGESGLLDDEDMEGIKEEAEK
Rm_fliktin      EEEYLDVLRNVWDRYRKDNPHVIDIEDISEGDVGEILNYLGNTGLLDDEDVEGIKEEASK
**:******.**::**:***:************.*******::*******:*******.*

PaOGS36577      RQQGGGDYDFLVHNAAMGGWGGGGHQFRKRWNQRLDGDENQKGSFLYSLKFVSPAVNREA
Rm_fliktin      RE-----YDFNTHNIAMSGFGGGG--FKKRWNQRLDGDENQKGNFLYSLKFVSPAINREA
*:      *** .** **.*:****  *:****************.***********:****

PaOGS36577      IESLKDDDGLDLPDERDEDILRLSPDINRREPDPWFPAFERGEAPEELFGNPSEEEYQRL
Rm_fliktin      IESLKDDDDLDLPDERDEDVLRLTSNVRR-EPDPWFPAFERGEAPEELFGNPSEEEYQRL
********.**********:***:.::.* ******************************

PaOGS36577      LLAQQSDHRAGPSRKRLTSLVRPHYNAREISPIPEVFLTPEKKYLYDTAIMKKRYPVTKR
Rm_fliktin      LLAQQNEHQAP-SRKRIASLRSHHS----ASSLPDVFFTPEKKYLYDTAIMKKRYPVTKR
*****.:*:*  ****::**   *      *.:*:**:**********************

PaOGS36577      SSNFYTSPPLLHHKNFAFAD-SEPRKKKDAIGTNVATTDPKVARELNQIFSSPTASDHTH
Rm_fliktin      SSNFYTSPPLLHHKNFAFTDNYETRRKKDATGTSVATTDPKVARELNQIFSSPVAEHSNE
*****************:*  *.*:**** **.*******************.*.. ..

PaOGS36577      SESNSKQPVHAKENSVKPSVDTEHVATTHSPVTTNSTVTAKSKEGEKKTEENVTIHSDHA
Rm_fliktin      AHSKD-----SAHNMDKPTADSVHVATTHSPAIT-ASTTALSKTKENETDKINRDNTTTQ
:.*:.      : .*  **:.*: ********. * ::.** **  *::*::     ::

PaOGS36577      QRSASEEETVEQPVTMSRAETPLDIKKKSINWSDYFGIDRRRKKAGPNGGEKESIPVEHP
Rm_fliktin      HRSGSEEETVEQPVTMSRSEAPLDIKKKSINWSDYFGIDRRRKKTS------SSPPDNHP
:**.**************:*:************************:.      .* * :**

PaOGS36577      VDNEWLLNQYYKTFAMNTNPGKKRSAPHMHDHMSSSSISQAKSKKSMVQQPFDTRVFDTD
Rm_fliktin      TNDEWLLNQYYKTFAMSTNPGKKRSIVYPHD--------AAKVKSTALQQPFDTRVFDTD
.::*************.********  : **         ** *.: :************

PaOGS36577      IFARTAQREAS--KKNSQPANSEQSEESRIDSMDAKLRSIEDQIVNEAVKYTGAHEGSTD
Rm_fliktin      IFARTTQHEASAVKKSVQTSNLDKNEEARLNNMDTKLRNIEDEIVNEAVKFTGAHEGSTD
*****:*:***  **. *.:* ::.**:*::.**:***.***:*******:*********

PaOGS36577      TKEIQEVKDKVMARLAAAYSLEKMRLALGEFKSSLMAQKMSRYNPENRQADATDEKKKKR
Rm_fliktin      SKEIQEVKDKVMARLAAAYSLEKMRQALGEFRSSLMAQKMSKYNPENRQTGNVDEKKKKR
:************************ *****:**********:********:. .*******

PaOGS36577      VAVKKEKAEDKKDEDKKKRGDDRNDESEEDEGEFLDGPVVVQPLSEGDMGRRVTDMDTED
Rm_fliktin      VAVKKEKAEEKKSEDKKKRGDG-HAESDEDMGEFLEGPVIVQPVSEGDMGRQEINSED-D
*********:**.*********. : **:** ****:***:***:*******:  : :  *

PaOGS36577      DECPVLEEILDTCRSADNLVGDHGQFFLPLCSLHQICYLCGPELGAPSPTACDLMFITEA
Rm_fliktin      ITCPVLDQIISKCRTANNIVGDHSQLFLPLCSLHQICYLCGPELGAPSPAACDLMFITEA
****::*:..**:*:*:****.*:************************:**********

PaOGS36577      DSMCLGDADCQTAARRSVAVLRRGRGHEEEGQCWRSPCIAHHFLHTPLTVPQPAATGREI
Rm_fliktin      EMLCRNDEDCRLAARRNVAILRKSQDHIEEGQCWRSPCIAHYFLQSPLPAPLSASSSR--
: :* .* **: ****.**:**:.:.* *************:**::**..* .*::.*

PaOGS36577      CPPAVRGS
Rm_fliktin      --------
```

(B)

```
PaOGS36577      --MHSWTSALLCLLVLLSLSQGQDEQP-SDSLRTAIEAVSRRQRDLATAGSNYYSGGLSQ
Rm_fliktin      --MGSWKAVLCCALVLVSFALGQDEQP-RDSLRTAIEAVSRRQRDLAAAGPNYYRGGLSQ
Cn_fliktin      MASRRWMLPLLILGTGVIASVPAARQSNRESLRSALDAVARKQRSLDAADTPEYYNDLHS
*   *    . :  :     .*.  :***:*::**:*:**.* :*..   * ..* .

PaOGS36577      YRYPERDSAVPEELAFLASPRDFTGDGQPENIGYGYQKTIASPTGIFPPQAPLENDVEP-
Rm_fliktin      YRYQDNEARPGEELAFLATPREFTGDGQPENIGYGYQKTIASPSGMFSPQTPLDGESPAN
Cn_fliktin      FKYHGGGADHKFDRDFDE----------------------EDEEIEFLPGDNGQLETAGE
::*    :    :  *                          .    * *    : :

PaOGS36577      -SHPSNSKVLEKILLEYLEDEMKAEDEDDDDEYLHRGSNDKRSAFRERAGEERGKLEAIK
Rm_fliktin      LEHAAKNKIMENMLVEYLEDELKGEPDDDEDDAYYR----KRSAFRERADDERRRYEAMK
Cn_fliktin      GLQGLNNKLFERALLDYLENLPEQEEP---------------------------------
:  :.*::*. *::***:  : *

PaOGS36577      KR--GRFGSTAFRERAHDR-LVDDVEQRKRKLFTEALVRKMEEEEDDERRERERGRLGDE
Rm_fliktin      KREFRSFAPSAFRERTHSSSVVDDVEEKKRKILADALVRKMEEEEEEERRDRERGRIDND
Cn_fliktin      -------VASIFRERERSGSRKRGGADDRLNLDDKDLARLFLEELQDGSP-----YGSLD
.: **** :.      .  : : ::  . *.* : ** ::           . :

PaOGS36577      DENEEDYLDVLRSVWEKYRRDNPNVIDIEDISEGDVSEILNYLGESGLLDDEDMEGIKEE
Rm_fliktin      EEAEEEYLDVLRNVWDRYRKDNPHVIDIEDISEGDVGEILNYLGNTGLLDDEDVEGIKEE
Cn_fliktin      DMDDDEYLTALQTLYDRYR-------------AGRGNKVYETAG----------------
:  :::** .*:.::::**                *  .:: :  *

PaOGS36577      AEKRQQGGGDYDFLVHNAAMGGWGGGGHQFRKRWNQRLDGDENQKGSFLYSLKFVSPAVN
Rm_fliktin      ASKRE-----YDFNTHNIAMSGFGGGG--FKKRWNQRLDGDENQKGNFLYSLKFVSPAIN
Cn_fliktin      ------------------------------PMSWGELLS---------------------
*.: *.

PaOGS36577      REAIESLKDDDGLDLPDERDEDILRLSPDINRREPDPWFPAFERGEAPEELFGNPSEEEY
Rm_fliktin      REAIESLKDDDDLDLPDERDEDVLRLTSNVRR-EPDPWFPAFERGEAPEELFGNPSEEEY
Cn_fliktin      --KYSSLRKNDDEEIAD-QNQNILYLPPTLERRNVNGRYPIG-------REFGN------
.**:.:*. ::.* :::::* *.. :.* : :  :*          . ***

PaOGS36577      QRLLLAQQSDHRAGPSRKRLTSLVRPHYNAREISPIPEVFLTPEKKYLYDTAIMKKRYPV
Rm_fliktin      QRLLLAQQNEHQAP-SRKRIASLRSHHS----ASSLPDVFFTPEKKYLYDTAIMKKRYPV
Cn_fliktin      ---------------------------------------------------YRRLTKRYPV
:.*****

PaOGS36577      TKRSSNFYTSPPLLHHKNFAFAD-SEPRKKKDAIGTNVATTDPKVARELNQIFSSPTASD
Rm_fliktin      TKRSSNFYTSPPLLHHKNFAFTDNYETRRKKDATGTSVATTDPKVARELNQIFSSPVAEH
Cn_fliktin      AKRS--------------------------PRPTQTKLKTTDPKVAQDLGALFGTQSTDT
:***                .   *.: *******::*. :*.:   :.

PaOGS36577      HTHSESNSKQPVHAKENSVKPSVDTEHVATTHSPVTTNSTVTAKSKEGEKKTEENVTIHS
Rm_fliktin      SNEAHSKD-----SAHNMDKPTADSVHVATTHSPAIT-ASTTALSKTKENETDKINRDNT
Cn_fliktin      HNHTHKSD---------------HSHEHDHEHIPKHDHVHDHVHDHDHEHEHDHGHESTS
..:....           .: .    * *         . .:  *:: :.      :

PaOGS36577      DHAQRSASEEETVEQPVTMSRAETPLDIKKKSINWSDYFGIDRRRKKAGPNGGEKESIPV
Rm_fliktin      TTQHRSGSEEETVEQPVTMSRSEAPLDIKKKSINWSDYFGIDRRRKKTS------SSPPD
Cn_fliktin      ETPPKVTPPPKDQKENATKQVKSKFIQVRKKSVDWSQYFGIDRRKKKAT-----FTAGHN
:   .  :  :: .*  .  .  ::::***::**:*******:**:          :

PaOGS36577      EHPVDNEWLLNQYYKTFAMNTNPGKKRSAPHMHDHMSSSSISQAKSKKSMVQQPFDTRVF
Rm_fliktin      NHPTNDEWLLNQYYKTFAMSTNPGKKRSIVYPHD--------AAKVKSTALQQPFDTRVF
Cn_fliktin      TQNQDDEWMLQRYYENMAENLKP-------------------------------------
:  ::**:*::**:.:* . :*

PaOGS36577      DTDIFARTAQREAS--KKNSQPANSEQSEESRIDSMDAKLRSIEDQIVNEAVKYTGAHEG
Rm_fliktin      DTDIFARTTQHEASAVKKSVQTSNLDKNEEARLNNMDTKLRNIEDEIVNEAVKFTGAHEG
Cn_fliktin      ----------------------KNDENEGKENLEQMDSKLKYIKDLIIEEALKYA----N
* ::. : .::.**:**: *:* *::**:*::     .

PaOGS36577      STDTKEIQEVKDKVMARLAAAYSLEKMRLALGEFKSSLMAQKMSRYNPENRQADATDEKK
Rm_fliktin      STDSKEIQEVKDKVMARLAAAYSLEKMRQALGEFRSSLMAQKMSKYNPENRQTGNVDEKK
Cn_fliktin      LEDETDLQKVKDKLMARMAAAYSLQKMRKALNELRNNVAAQKEAEKMQENNQTSNFRENS
* .::*:****:***:******:*** **.*::..: *** :.   **.*:.   *:.

PaOGS36577      KKRVAVKKEKAEDKKDEDKKKRGDDRNDESEEDEGEFLDGPVVVQPLSEGDMGRRVTDMD
Rm_fliktin      KKRVAVKKEKAEEKKSEDKKKRGDG-HAESDEDMGEFLEGPVIVQPVSEGDMGRQEINSE
Cn_fliktin      NGNANSNSDNGGRTDDKRSS-------INNNIDDREFIE---------------------
: ..  :.::.  ...: ..         :.: *  **::

PaOGS36577      TEDDECPVLEEILDTCRSADNLVGDHGQFFLPLCSLHQICYLCGPELGAPSPTACDLMFI
Rm_fliktin      D-DITCPVLDQIISKCRTANNIVGDHSQLFLPLCSLHQICYLCGPELGAPSPAACDLMFI
Cn_fliktin      -ESRSCPELEAIEWRCRNVDNLAGDTFRMLYVPCVKLQICKACAQDEELTTLLPCAVNYA
.  ** *: *   **..:*:.**  :::   *   ***  *. :   .:  .* : :

PaOGS36577      TEADSMCLGD----------------ADCQTAARRSVAVLRRGRGHEEE---------GQ
Rm_fliktin      TEAEMLCRND----------------EDCRLAARRNVAILRKSQDHIEE---------GQ
Cn_fliktin      LEARKVCDALDSREGQLSSSRLTAATAETRYAERQSCANTAVLLSQLQPPTTASAKCRAA
**  :*                 : : * *:. *       .: :            .

PaOGS36577      CWRSPCIAHHFLHTPLTVPQPAATGREICPPAVRGS---------
Rm_fliktin      CWRSPCIAHYFLQSPLPAPLSASSSR-------------------
Cn_fliktin      SGRDSCIRHYHARYRYFREPPMASNHRPHHAGALDALQQTMSKR-
. *..** *:. :       . ::.:
```

Supplementary Information S4: Quadrupole Orbitrap MS^2 spectra generated by *R. maderae* brain tissue extract analysis and MALDI-TOF/TOF MS

Adipokinetic hormone-1 (AKH-1)/Adipokinetic hormone-2 (AKH-2)

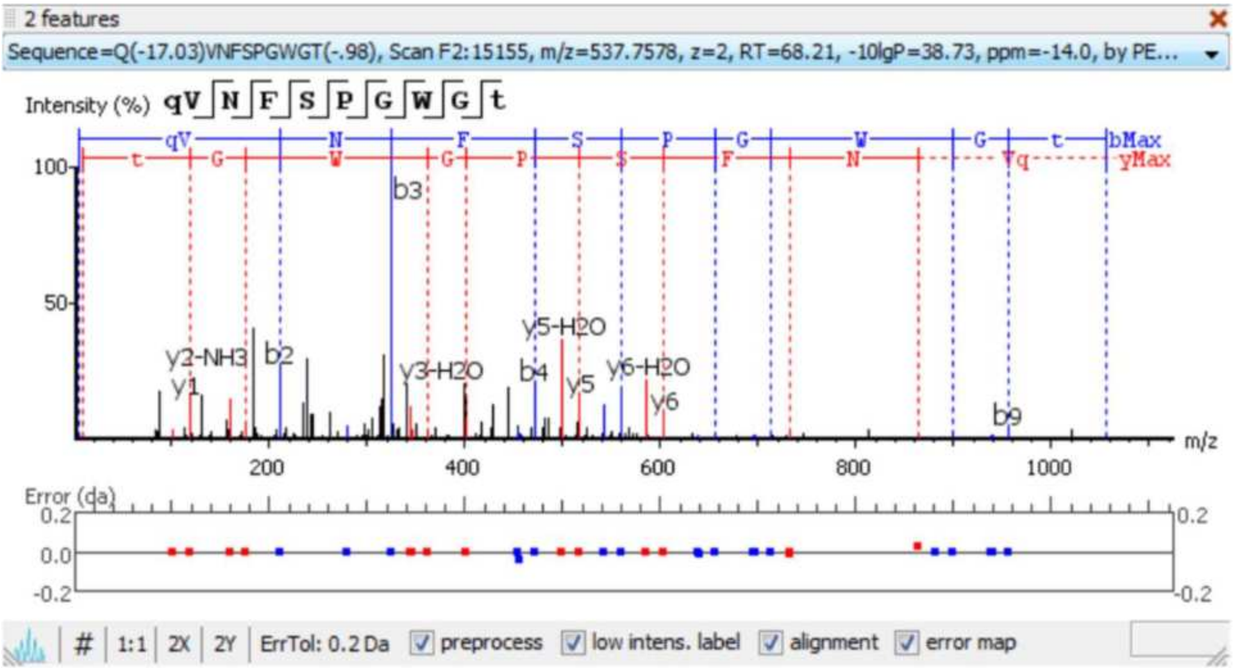

#	b	b-H2O	b-NH3	b (2+)	Seq	y	y-H2O	y-NH3	y (2+)	#
1	112.04	94.03	95.01	56.52	Q(-17.03)					10
2	211.11	193.10	194.08	106.05	V	963.47	945.46	946.44	482.23	9
3	325.15	307.14	308.12	163.08	N	864.36	846.39	847.37	432.70	8
4	472.22	454.21	455.24	236.61	F	750.36	732.34	733.34	375.68	7
5	559.25	541.24	542.22	280.13	S	603.29	585.28	586.26	302.14	6
6	656.30	638.29	639.29	328.65	P	516.26	498.25	499.23	258.63	5
7	713.33	695.32	696.30	357.16	G	419.20	401.19	402.18	210.10	4
8	899.40	881.39	882.39	450.20	W	362.18	344.17	345.16	181.59	3
9	956.43	938.42	939.40	478.71	G	176.10	158.09	159.08	88.55	2
10					T(-.98)	119.08	101.07	102.05	60.04	1

Adipokinetic hormone-1-PP (AKH-1-PP)

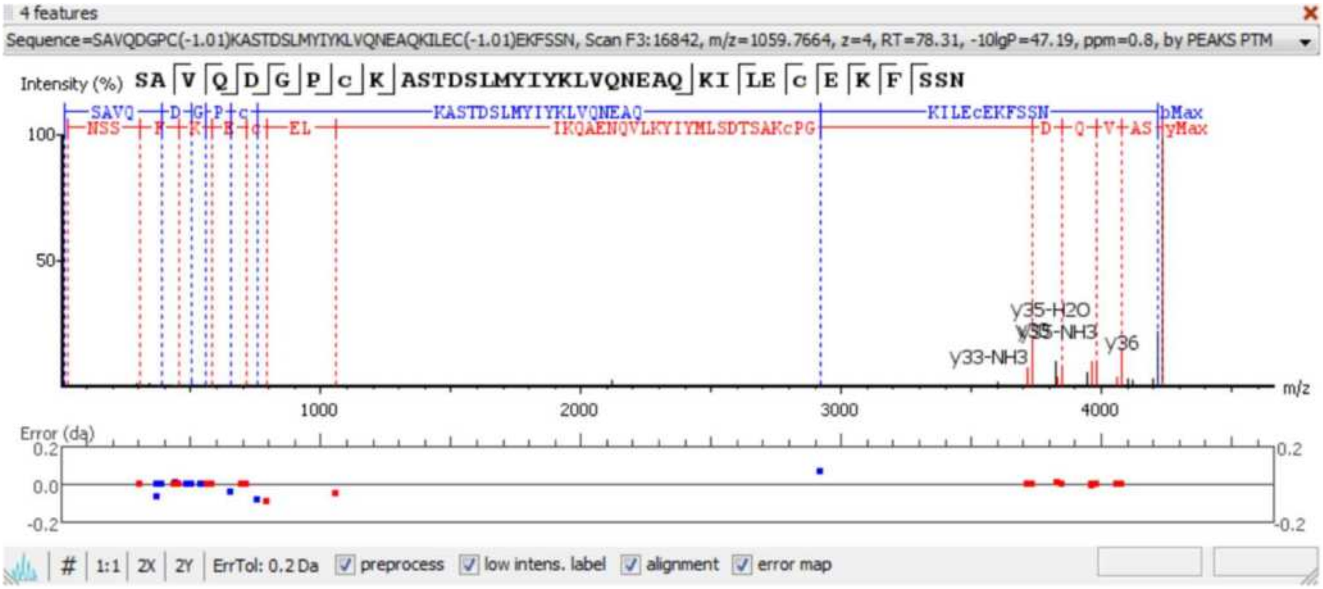

#	b	b-H2O	b-NH3	b (2+)	Seq	y	y-H2O	y-NH3	y (2+)	#
1	88.04	70.03	71.01	44.52	S					38
2	159.08	141.07	142.05	80.04	A	4149.00	4130.99	4131.97	2075.00	37
3	258.15	240.13	241.12	129.57	V	4077.97	4059.95	4060.94	2039.48	36
4	386.20	368.19	369.25	193.60	Q	3978.90	3960.88	3961.88	1989.95	35
5	501.23	483.22	484.21	251.12	D	3850.84	3832.81	3833.81	1925.92	34
6	558.25	540.24	541.23	279.63	G	3735.81	3717.80	3718.78	1868.40	33
7	655.35	637.29	638.28	328.15	P	3678.79	3660.77	3661.76	1839.89	32
8	757.40	739.30	740.28	379.15	C(-1.01)	3581.73	3563.72	3564.71	1791.37	31
9	885.40	867.39	868.37	443.19	K	3479.73	3461.72	3462.70	1740.37	30
10	956.44	938.43	939.41	478.72	A	3351.64	3333.63	3334.61	1676.32	29
11	1043.47	1025.46	1026.44	522.24	S	3280.60	3262.59	3263.57	1640.80	28
12	1144.52	1126.51	1127.49	572.76	T	3193.57	3175.56	3176.54	1597.28	27
13	1259.55	1241.53	1242.52	630.27	D	3092.52	3074.51	3075.49	1546.76	26
14	1346.58	1328.57	1329.55	673.79	S	2977.49	2959.48	2960.47	1489.25	25
15	1459.66	1441.65	1442.63	730.33	L	2890.46	2872.45	2873.43	1445.73	24
16	1590.70	1572.69	1573.67	795.85	M	2777.38	2759.37	2760.35	1389.19	23
17	1753.77	1735.75	1736.74	877.38	Y	2646.34	2628.33	2629.31	1323.67	22
18	1866.85	1848.84	1849.82	933.92	I	2483.27	2465.26	2466.25	1242.14	21
19	2029.91	2011.90	2012.89	1015.46	Y	2370.19	2352.18	2353.16	1185.59	20
20	2158.01	2140.00	2140.98	1079.50	K	2207.12	2189.11	2190.10	1104.06	19
21	2271.09	2253.08	2254.06	1136.05	L	2079.03	2061.02	2062.00	1040.02	18
22	2370.16	2352.15	2353.13	1185.58	V	1965.95	1947.94	1948.92	983.47	17
23	2498.22	2480.21	2481.19	1249.61	Q	1866.88	1848.87	1849.85	933.94	16
24	2612.26	2594.25	2595.23	1306.63	N	1738.82	1720.81	1721.79	869.91	15
25	2741.30	2723.29	2724.28	1371.15	E	1624.78	1606.77	1607.75	812.89	14
26	2812.34	2794.33	2795.31	1406.67	A	1495.73	1477.72	1478.71	748.37	13
27	2940.40	2922.32	2923.37	1470.70	Q	1424.70	1406.69	1407.67	712.85	12
28	3068.49	3050.48	3051.47	1534.75	K	1296.64	1278.63	1279.61	648.82	11
29	3181.58	3163.57	3164.55	1591.29	I	1168.54	1150.53	1151.52	584.77	10
30	3294.66	3276.65	3277.64	1647.83	L	1055.51	1037.45	1038.43	528.23	9
31	3423.71	3405.69	3406.68	1712.35	E	942.37	924.36	925.35	471.69	8
32	3525.71	3507.70	3508.68	1763.35	C(-1.01)	813.33	795.41	796.31	407.17	7
33	3654.75	3636.74	3637.72	1827.87	E	711.33	693.32	694.30	356.17	6
34	3782.84	3764.83	3765.82	1891.92	K	582.29	564.28	565.26	291.64	5
35	3929.91	3911.90	3912.89	1965.46	F	454.19	436.18	437.17	227.60	4
36	4016.94	3998.93	3999.92	2008.97	S	307.13	289.11	290.10	154.06	3
37	4103.98	4085.97	4086.95	2052.49	S	220.09	202.08	203.07	110.55	2
38					N	133.06	115.05	116.03	67.03	1

AKH-Corazonin-like peptide (ACP)

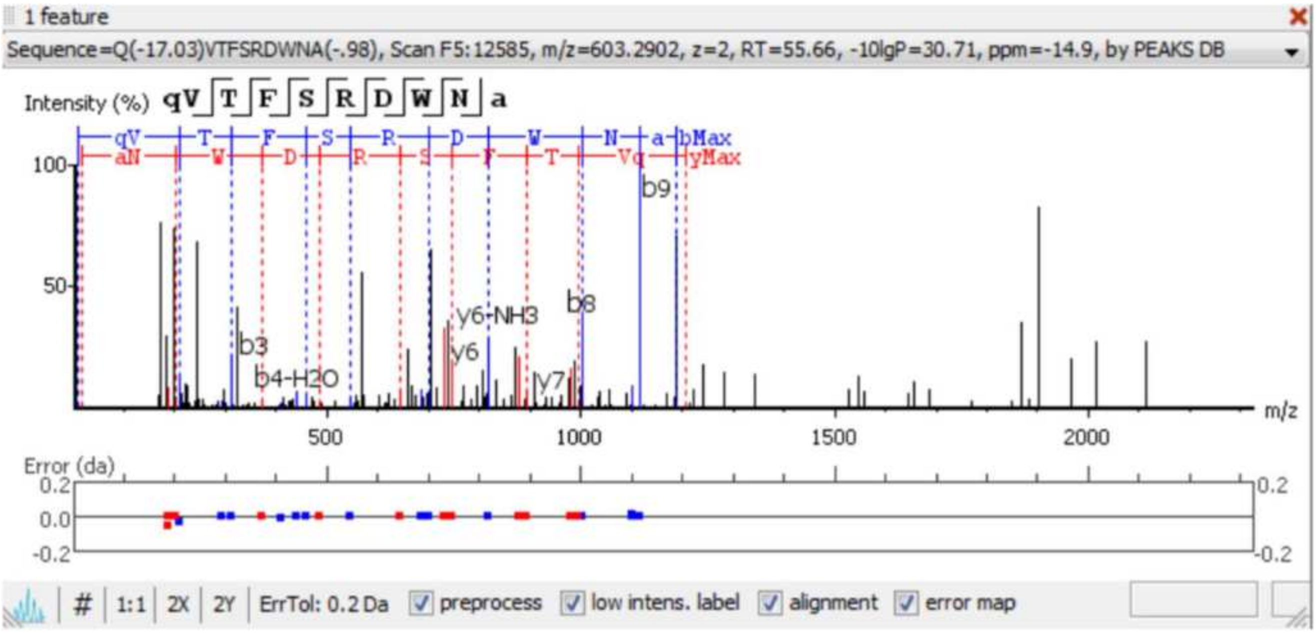

#	b	b-H2O	b-NH3	b (2+)	Seq	y	y-H2O	y-NH3	y (2+)	#
1	112.04	94.03	95.01	56.52	Q(-17.03)					10
2	211.14	193.10	194.08	106.05	V	1094.54	1076.53	1077.51	547.77	9
3	312.15	294.14	295.13	156.58	T	995.47	977.46	978.44	498.23	8
4	459.22	441.21	442.20	230.11	F	894.42	876.41	877.39	447.71	7
5	546.25	528.25	529.23	273.63	S	747.35	729.34	730.33	374.18	6
6	702.36	684.35	685.33	351.68	R	660.32	642.31	643.29	330.66	5
7	817.38	799.37	800.36	409.21	D	504.22	486.21	487.19	252.61	4
8	1003.46	985.45	986.44	502.23	W	389.19	371.18	372.17	195.10	3
9	1117.50	1099.48	1100.49	559.25	N	203.11	185.16	186.09	102.06	2
10					A(-.98)	89.07	71.06	72.04	45.04	1

Agatoxin-like peptides (ALP)-PP-1_partial

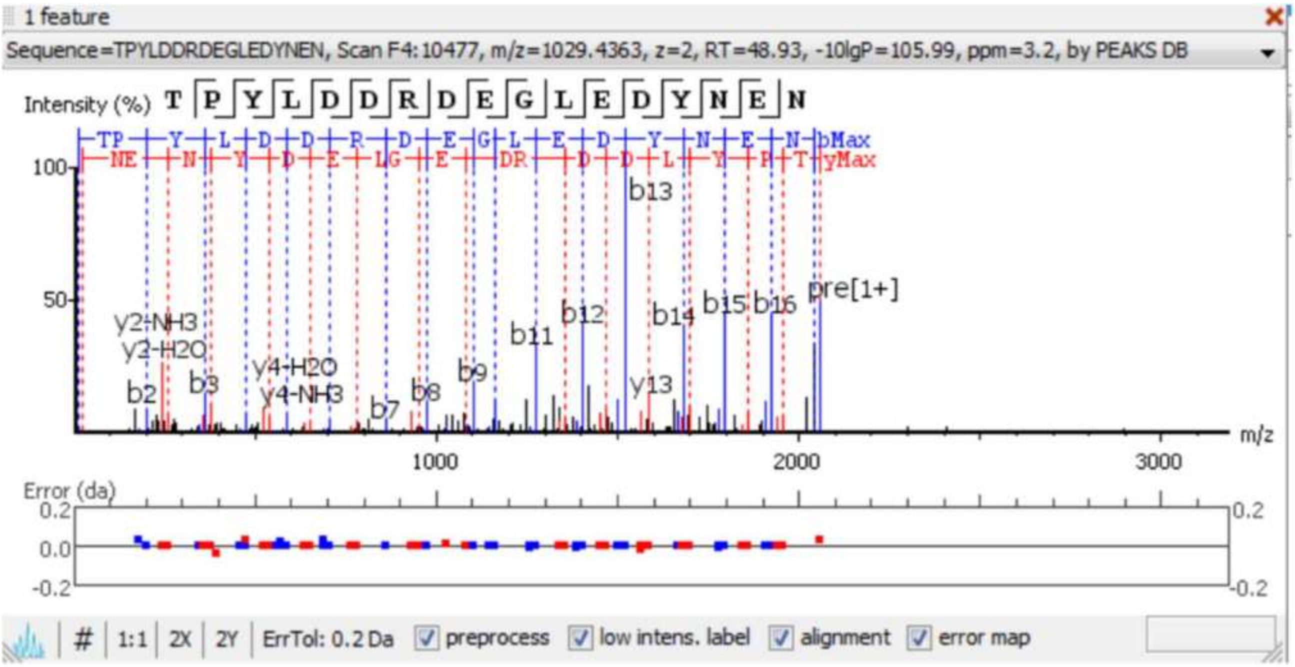

#	b	b-H2O	b-NH3	b (2+)	Seq	y	y-H2O	y-NH3	y (2+)	#
1	102.06	84.04	85.03	51.53	T					17
2	199.11	181.06	182.08	100.05	P	1956.80	1938.80	1939.78	978.90	16
3	362.17	344.16	345.14	181.59	Y	1859.76	1841.74	1842.73	930.38	15
4	475.26	457.24	458.23	238.13	L	1696.69	1678.68	1679.67	848.85	14
5	590.28	572.27	573.23	295.64	D	1583.61	1565.62	1566.58	792.30	13
6	705.31	687.30	688.25	353.15	D	1468.58	1450.57	1451.56	734.79	12
7	861.41	843.40	844.38	431.21	R	1353.56	1335.54	1336.53	677.28	11
8	976.44	958.43	959.41	488.72	D	1197.45	1179.44	1180.43	599.23	10
9	1105.48	1087.47	1088.45	553.24	E	1082.42	1064.42	1065.40	541.71	9
10	1162.50	1144.49	1145.47	581.75	G	953.38	935.37	936.36	477.16	8
11	1275.58	1257.58	1258.57	638.29	L	896.36	878.35	879.34	448.68	7
12	1404.62	1386.62	1387.62	702.81	E	783.28	765.27	766.25	392.18	6
13	1519.65	1501.64	1502.63	760.33	D	654.24	636.22	637.21	327.62	5
14	1682.72	1664.70	1665.69	841.86	Y	539.21	521.20	522.18	270.10	4
15	1796.76	1778.74	1779.75	898.88	N	376.15	358.14	359.12	188.57	3
16	1925.80	1907.79	1908.78	963.41	E	262.10	244.09	245.08	131.55	2
17					N	133.06	115.05	116.03	67.03	1

Allatotropin (AT)

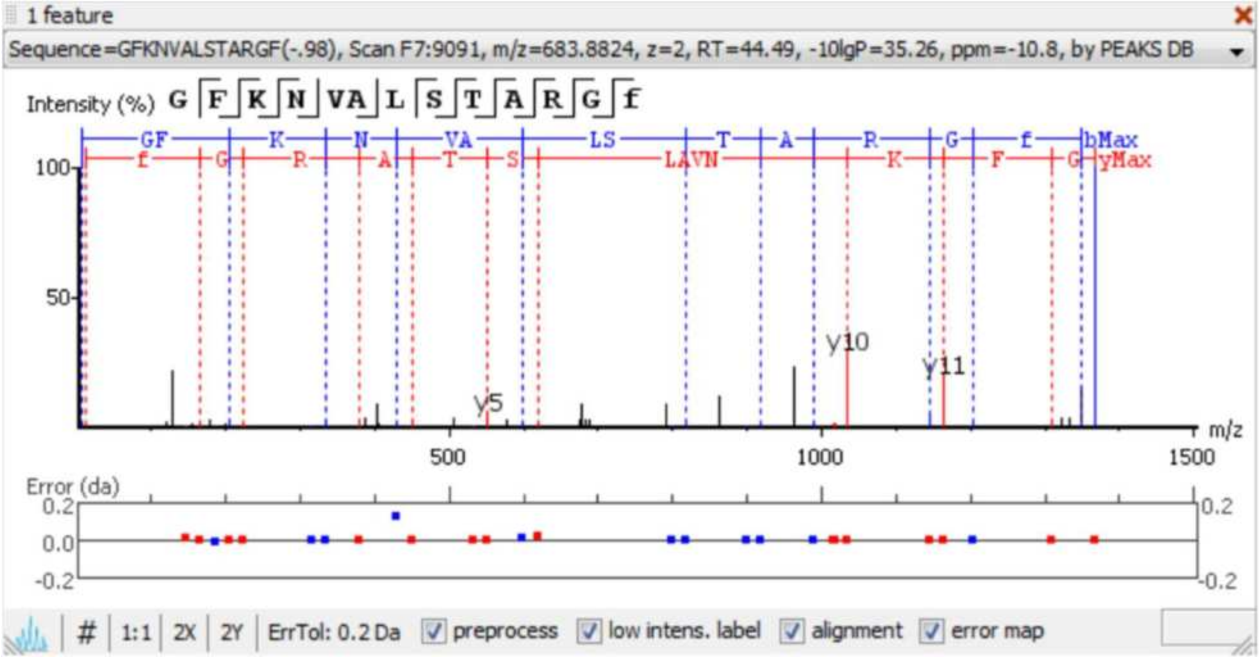

#	b	b-H2O	b-NH3	b (2+)	Seq	y	y-H2O	y-NH3	y (2+)	#
1	58.03	40.02	41.00	29.51	G					13
2	205.10	187.11	188.07	103.05	F	1309.74	1291.73	1292.71	655.37	12
3	333.19	315.18	316.17	167.10	K	1162.67	1144.66	1145.64	581.83	11
4	447.24	429.09	430.21	224.12	N	1034.57	1016.56	1017.55	517.79	10
5	546.30	528.29	529.28	273.65	V	920.53	902.52	903.50	460.77	9
6	617.34	599.31	600.31	309.17	A	821.46	803.45	804.44	411.23	8
7	730.43	712.41	713.40	365.71	L	750.43	732.42	733.40	375.71	7
8	817.45	799.44	800.43	409.23	S	637.34	619.33	620.28	319.17	6
9	918.50	900.50	901.48	459.75	T	550.31	532.29	533.28	275.65	5
10	989.55	971.53	972.52	495.27	A	449.26	431.25	432.23	225.13	4
11	1145.64	1127.63	1128.62	573.32	R	378.22	360.21	361.20	189.61	3
12	1202.66	1184.65	1185.64	601.83	G	222.12	204.11	205.10	111.56	2
13					F(-.98)	165.10	147.08	148.08	83.05	1

Allatotropin-PP-1 (AT-PP-1)

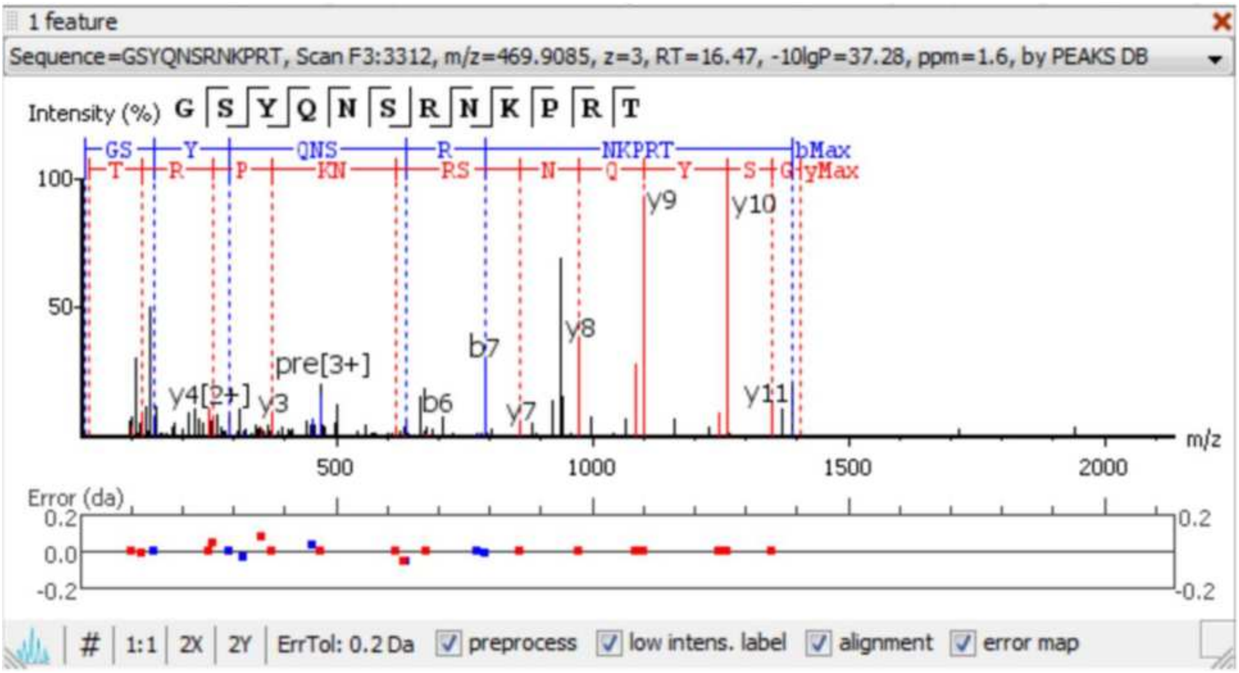

#	b	b-H2O	b-NH3	b (2+)	Seq	y	y-H2O	y-NH3	y (2+)	#
1	58.03	40.02	41.00	29.51	G					12
2	145.06	127.05	128.03	73.03	S	1350.68	1332.68	1333.66	675.85	11
3	308.12	290.11	291.10	154.56	Y	1263.65	1245.64	1246.63	632.38	10
4	436.18	418.17	419.16	218.59	Q	1100.59	1082.58	1083.57	550.80	9
5	550.23	532.22	533.20	275.61	N	972.53	954.52	955.51	486.77	8
6	637.32	619.25	620.23	319.16	S	858.49	840.48	841.46	429.75	7
7	793.37	775.34	776.33	397.18	R	771.46	753.45	754.43	386.23	6
8	907.40	889.39	890.38	454.16	N	615.35	597.35	598.33	308.18	5
9	1035.50	1017.49	1018.47	518.25	K	501.31	483.30	484.29	251.15	4
10	1132.55	1114.54	1115.52	566.77	P	373.22	355.12	356.19	187.11	3
11	1288.65	1270.64	1271.62	644.83	R	276.17	258.11	259.14	138.58	2
12					T	120.08	102.06	103.04	60.53	1

Allatotropin-PP-1 (AT-PP-1)

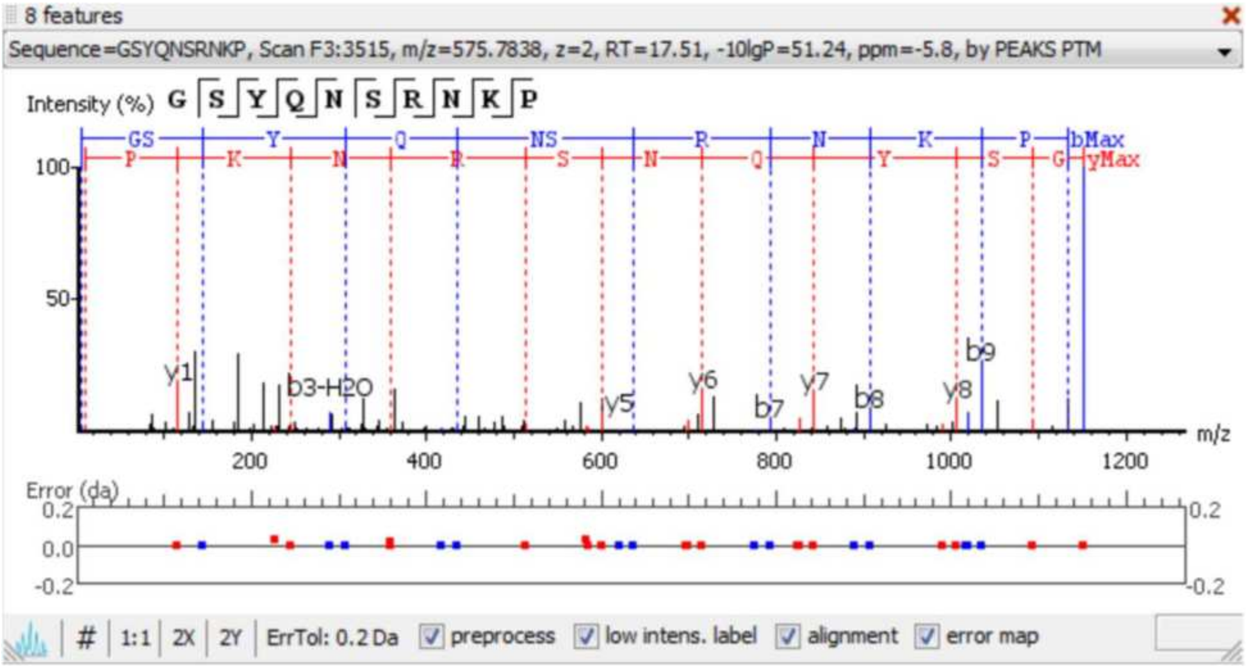

#	b	b-H2O	b-NH3	b (2+)	Seq	y	y-H2O	y-NH3	y (2+)	#
1	58.03	40.02	41.00	29.51	G					10
2	145.06	127.05	128.03	73.03	S	1093.54	1075.53	1076.51	547.27	9
3	308.12	290.11	291.10	154.56	Y	1006.51	988.50	989.48	503.75	8
4	436.18	418.17	419.16	218.59	Q	843.44	825.43	826.42	422.22	7
5	550.23	532.22	533.20	275.61	N	715.38	697.37	698.36	358.18	6
6	637.26	619.25	620.23	319.13	S	601.34	583.29	584.31	301.17	5
7	793.36	775.35	776.34	397.18	R	514.31	496.30	497.28	257.65	4
8	907.40	889.39	890.37	454.20	N	358.18	340.20	341.18	179.60	3
9	1035.50	1017.49	1018.47	518.25	K	244.17	226.12	227.14	122.58	2
10					P	116.07	98.06	99.04	58.54	1

Allatotropin-PP-2 (AT-PP-2)_part 1

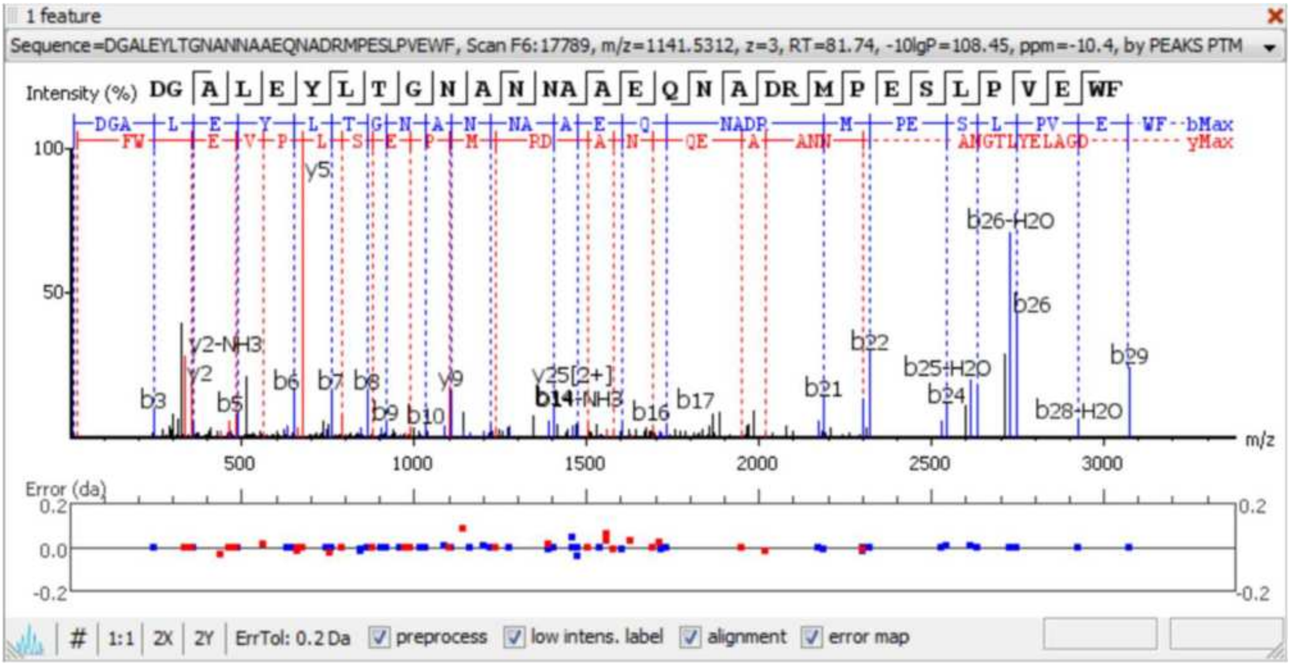

#	b	b-H2O	b-NH3	b (2+)	Seq	y	y-H2O	y-NH3	y (2+)	#
1	116.03	98.02	99.01	58.52	D					31
2	173.06	155.05	156.03	87.03	G	3307.53	3289.52	3290.51	1654.27	30
3	244.09	226.08	227.07	122.55	A	3250.51	3232.50	3233.48	1625.72	29
4	357.18	339.17	340.15	179.09	L	3179.47	3161.46	3162.45	1590.24	28
5	486.22	468.21	469.19	243.61	E	3066.39	3048.38	3049.36	1533.69	27
6	649.28	631.27	632.26	325.14	Y	2937.35	2919.34	2920.32	1469.17	26
7	762.37	744.36	745.34	381.68	L	2774.28	2756.27	2757.26	1387.62	25
8	863.42	845.42	846.41	432.21	T	2661.20	2643.19	2644.17	1331.10	24
9	920.44	902.43	903.41	460.72	G	2560.15	2542.14	2543.12	1280.58	23
10	1034.48	1016.47	1017.45	517.74	N	2503.13	2485.12	2486.10	1252.07	22
11	1105.52	1087.50	1088.49	553.26	A	2389.09	2371.08	2372.06	1195.04	21
12	1219.56	1201.54	1202.53	610.28	N	2318.05	2300.04	2301.04	1159.53	20
13	1333.60	1315.59	1316.58	667.30	N	2204.01	2186.00	2186.98	1102.50	19
14	1404.64	1386.63	1387.62	702.82	A	2089.96	2071.95	2072.94	1045.48	18
15	1475.68	1457.62	1458.64	738.34	A	2018.94	2000.92	2001.90	1009.96	17
16	1604.73	1586.71	1587.69	802.86	E	1947.89	1929.88	1930.86	974.44	16
17	1732.78	1714.77	1715.77	866.89	Q	1818.85	1800.84	1801.82	909.92	15
18	1846.82	1828.81	1829.79	923.91	N	1690.79	1672.78	1673.76	845.89	14
19	1917.86	1899.85	1900.83	959.43	A	1576.76	1558.67	1559.68	788.87	13
20	2032.88	2014.87	2015.86	1016.94	D	1505.72	1487.70	1488.68	753.39	12
21	2189.00	2170.98	2171.96	1094.99	R	1390.68	1372.67	1373.66	695.84	11
22	2320.02	2302.02	2303.02	1160.52	M	1234.59	1216.57	1217.55	617.79	10
23	2417.08	2399.07	2400.05	1209.04	P	1103.54	1085.53	1086.51	552.27	9
24	2546.11	2528.11	2529.09	1273.57	E	1006.49	988.48	989.45	503.74	8
25	2633.15	2615.13	2616.13	1317.08	S	877.45	859.43	860.42	439.26	7
26	2746.24	2728.23	2729.21	1373.62	L	790.42	772.40	773.39	395.71	6
27	2843.29	2825.28	2826.26	1422.15	P	677.33	659.32	660.32	339.16	5
28	2942.36	2924.35	2925.33	1471.72	V	580.28	562.25	563.25	290.64	4
29	3071.41	3053.39	3054.37	1536.21	E	481.21	463.20	464.18	241.10	3
30	3257.48	3239.47	3240.45	1629.24	W	352.17	334.15	335.14	176.58	2
31					F	166.09	148.08	149.06	83.54	1

Allatotropin-PP-2 (AT-PP-2)_part 2

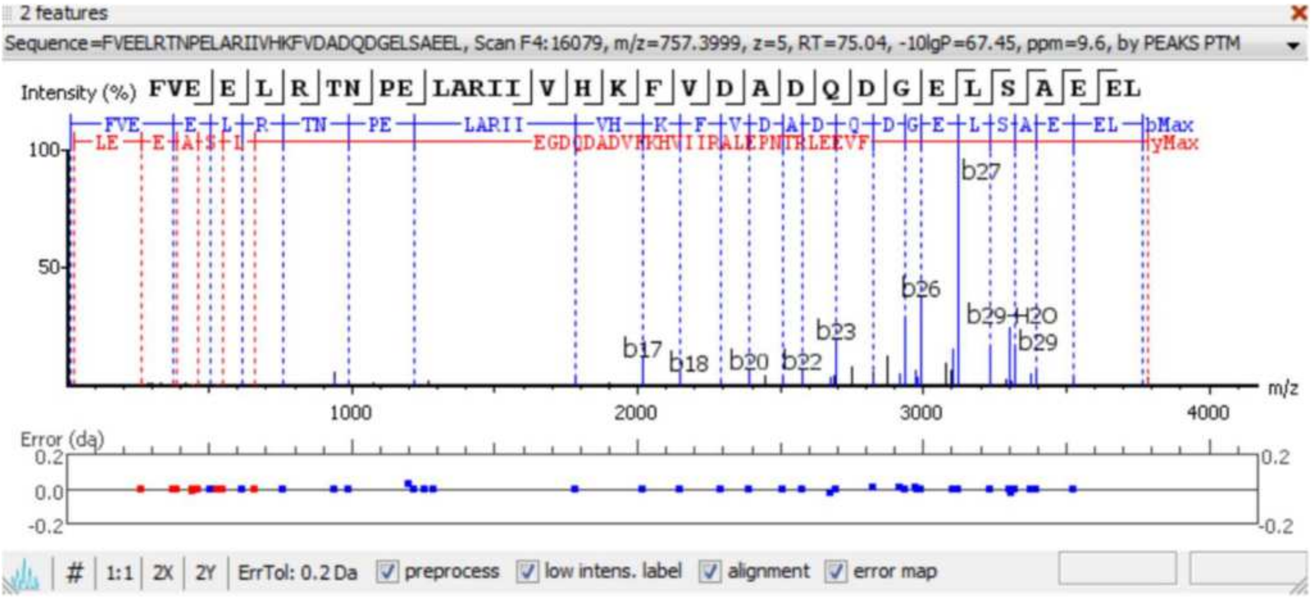

#	b	b-H2O	b-NH3	b (2+)	Seq	y	y-H2O	y-NH3	y (2+)	#
1	148.08	130.07	131.05	74.54	F					33
2	247.14	229.13	230.12	124.07	V	3635.85	3617.84	3618.83	1818.43	32
3	376.19	358.18	359.16	188.59	E	3536.79	3518.78	3519.76	1768.89	31
4	505.23	487.22	488.20	253.11	E	3407.74	3389.73	3390.72	1704.37	30
5	618.31	600.30	601.29	309.66	L	3278.70	3260.69	3261.67	1639.85	29
6	774.41	756.40	757.38	387.71	R	3165.62	3147.61	3148.59	1583.31	28
7	875.46	857.45	858.44	438.23	T	3009.52	2991.51	2992.49	1505.26	27
8	989.51	971.50	972.48	495.25	N	2908.47	2890.46	2891.44	1454.73	26
9	1086.56	1068.55	1069.53	543.78	P	2794.43	2776.42	2777.40	1397.71	25
10	1215.61	1197.55	1198.57	608.30	E	2697.37	2679.36	2680.35	1349.19	24
11	1328.68	1310.67	1311.66	664.84	L	2568.33	2550.32	2551.30	1284.67	23
12	1399.72	1381.71	1382.70	700.36	A	2455.25	2437.24	2438.22	1228.12	22
13	1555.82	1537.81	1538.80	778.41	R	2384.21	2366.20	2367.18	1192.60	21
14	1668.91	1650.90	1651.88	834.95	I	2228.11	2210.10	2211.08	1114.55	20
15	1782.00	1763.98	1764.96	891.50	I	2115.02	2097.01	2098.00	1058.01	19
16	1881.06	1863.05	1864.03	941.04	V	2001.94	1983.93	1984.91	1001.47	18
17	2018.12	2000.11	2001.09	1009.56	H	1902.87	1884.86	1885.84	951.94	17
18	2146.21	2128.20	2129.19	1073.61	K	1765.81	1747.80	1748.79	883.41	16
19	2293.27	2275.27	2276.25	1147.14	F	1637.72	1619.71	1620.69	819.36	15
20	2392.35	2374.34	2375.32	1196.68	V	1490.65	1472.64	1473.62	745.82	14
21	2507.38	2489.37	2490.35	1254.19	D	1391.58	1373.57	1374.55	696.29	13
22	2578.41	2560.40	2561.39	1289.71	A	1276.55	1258.54	1259.53	638.78	12
23	2693.45	2675.45	2676.41	1347.22	D	1205.52	1187.51	1188.49	603.26	11
24	2821.49	2803.49	2804.47	1411.25	Q	1090.49	1072.48	1073.46	545.74	10
25	2936.53	2918.51	2919.50	1468.76	D	962.43	944.42	945.40	481.72	9
26	2993.55	2975.52	2976.53	1497.27	G	847.40	829.39	830.38	424.20	8
27	3122.59	3104.58	3105.56	1561.80	E	790.38	772.37	773.36	395.69	7
28	3235.67	3217.66	3218.65	1618.34	L	661.34	643.33	644.31	331.17	6
29	3322.71	3304.70	3305.70	1661.85	S	548.26	530.25	531.23	274.63	5
30	3393.75	3375.73	3376.72	1697.37	A	461.23	443.21	444.21	231.11	4
31	3522.78	3504.78	3505.76	1761.89	E	390.19	372.18	373.16	195.59	3
32	3651.83	3633.82	3634.80	1826.41	E	261.14	243.13	244.12	131.07	2
33					L	132.10	114.09	115.07	66.55	1

Allatotropin-PP-2 (AT-PP-2)_part 3

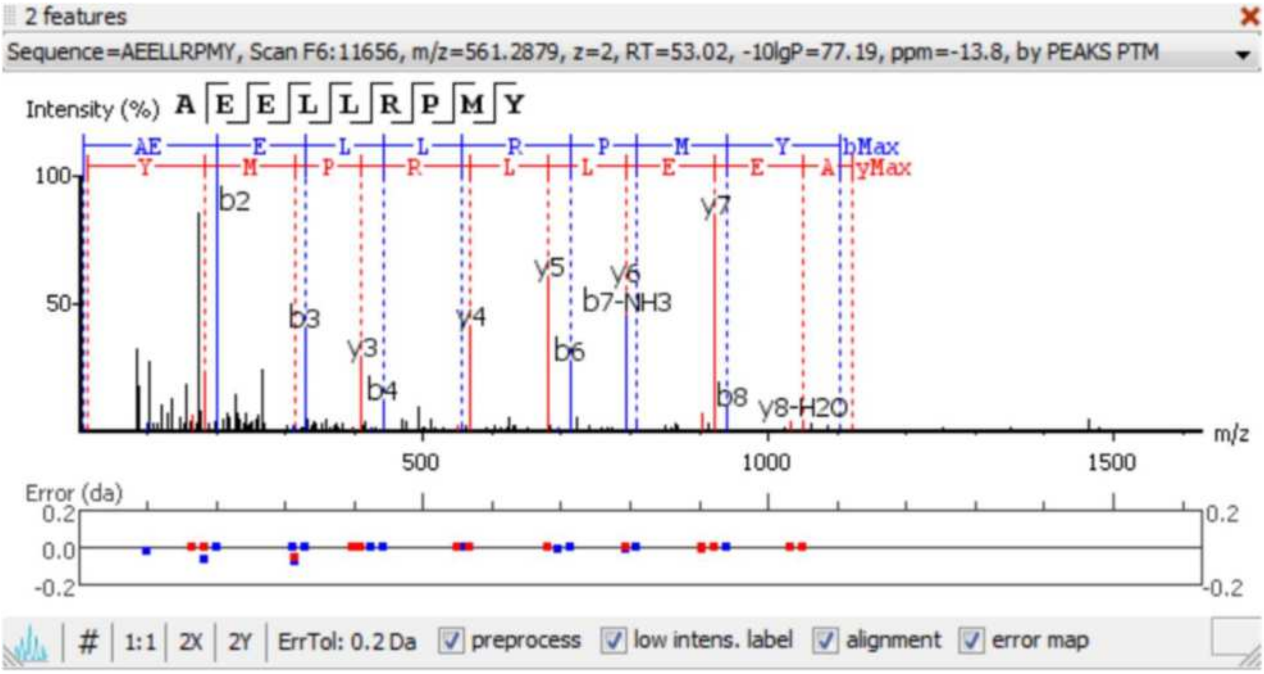

#	b	b-H2O	b-NH3	b (2+)	Seq	y	y-H2O	y-NH3	y (2+)	#
1	72.04	54.03	55.02	36.52	A					9
2	201.09	183.15	184.06	101.07	E	1050.53	1032.52	1033.50	525.76	8
3	330.13	312.12	313.19	165.57	E	921.49	903.48	904.48	461.24	7
4	443.21	425.20	426.19	222.11	L	792.44	774.43	775.42	396.73	6
5	556.30	538.29	539.27	278.65	L	679.36	661.35	662.33	340.18	5
6	712.40	694.40	695.37	356.70	R	566.28	548.27	549.24	283.64	4
7	809.46	791.44	792.44	405.23	P	410.17	392.16	393.15	205.59	3
8	940.49	922.48	923.47	470.75	M	313.19	295.11	296.09	157.06	2
9					Y	182.08	164.07	165.05	91.54	1

Allatostatin-A–PP-1 (AST-A-PP-1)

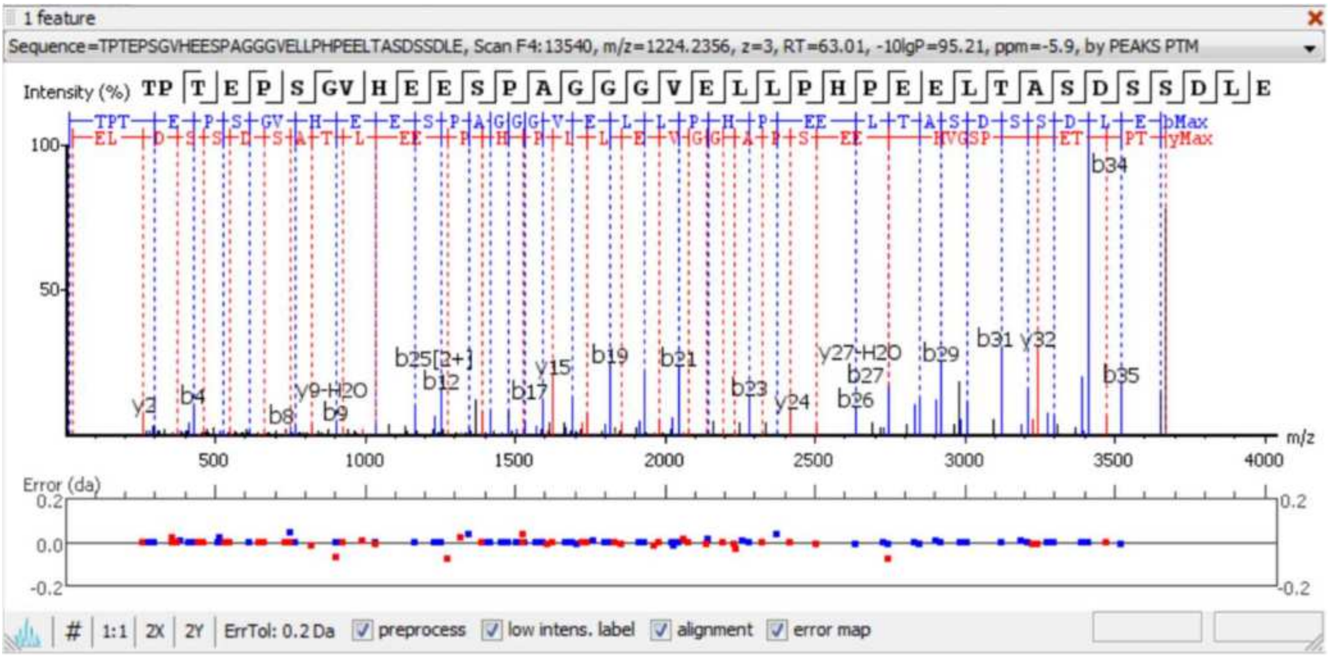

#	b	b-H2O	b-NH3	b (2+)	Seq	y	y-H2O	y-NH3	y (2+)	#
1	102.06	84.04	85.03	51.53	T					36
2	199.11	181.10	182.08	100.05	P	3569.64	3551.63	3552.61	1785.32	35
3	300.16	282.14	283.13	150.58	T	3472.59	3454.58	3455.56	1736.79	34
4	429.20	411.19	412.17	215.10	E	3371.54	3353.53	3354.51	1686.27	33
5	526.25	508.24	509.22	263.63	P	3242.51	3224.50	3225.47	1621.75	32
6	613.28	595.27	596.26	307.14	S	3145.44	3127.43	3128.42	1573.22	31
7	670.30	652.29	653.28	335.65	G	3058.41	3040.40	3041.39	1529.67	30
8	769.37	751.36	752.29	385.17	V	3001.39	2983.38	2984.36	1501.20	29
9	906.44	888.42	889.41	453.72	H	2902.32	2884.31	2885.30	1451.66	28
10	1035.48	1017.46	1018.45	518.21	E	2765.26	2747.33	2748.24	1383.13	27
11	1164.52	1146.51	1147.49	582.76	E	2636.22	2618.21	2619.19	1318.59	26
12	1251.55	1233.54	1234.52	626.27	S	2507.19	2489.17	2490.15	1254.09	25
13	1348.56	1330.59	1331.58	674.80	P	2420.15	2402.14	2403.12	1210.57	24
14	1419.65	1401.63	1402.61	710.32	A	2323.09	2305.08	2306.07	1162.05	23
15	1476.66	1458.65	1459.63	738.83	G	2252.06	2234.06	2235.07	1126.53	22
16	1533.69	1515.67	1516.66	767.34	G	2195.04	2177.02	2178.01	1098.02	21
17	1590.71	1572.69	1573.68	795.85	G	2138.02	2120.00	2120.99	1069.51	20
18	1689.78	1671.76	1672.74	845.39	V	2081.00	2062.97	2063.96	1041.00	19
19	1818.82	1800.80	1801.79	909.91	E	1981.93	1963.93	1964.92	991.45	18
20	1931.90	1913.89	1914.87	966.45	L	1852.89	1834.86	1835.85	926.94	17
21	2044.99	2026.98	2027.97	1022.99	L	1739.80	1721.79	1722.77	870.40	16
22	2142.02	2124.02	2125.01	1071.52	P	1626.72	1608.71	1609.69	813.86	15
23	2279.10	2261.07	2262.07	1140.05	H	1529.67	1511.65	1512.63	765.33	14
24	2376.11	2358.14	2359.12	1188.57	P	1392.60	1374.59	1375.57	696.80	13
25	2505.19	2487.18	2488.16	1253.10	E	1295.55	1277.62	1278.52	648.27	12
26	2634.24	2616.22	2617.21	1317.62	E	1166.51	1148.50	1149.48	583.75	11
27	2747.33	2729.31	2730.29	1374.16	L	1037.47	1019.45	1020.44	519.23	10
28	2848.37	2830.36	2831.34	1424.68	T	924.38	906.44	907.35	462.69	9
29	2919.41	2901.38	2902.37	1460.20	A	823.35	805.32	806.30	412.17	8
30	3006.43	2988.43	2989.41	1503.72	S	752.29	734.29	735.27	376.65	7
31	3121.47	3103.45	3104.43	1561.23	D	665.26	647.25	648.24	333.13	6
32	3208.50	3190.47	3191.47	1604.75	S	550.24	532.23	533.21	275.62	5
33	3295.53	3277.51	3278.50	1648.26	S	463.20	445.19	446.18	232.10	4
34	3410.56	3392.55	3393.53	1705.79	D	376.17	358.16	359.12	188.59	3
35	3523.65	3505.62	3506.61	1762.31	L	261.14	243.13	244.12	131.07	2
36					E	148.06	130.05	131.03	74.53	1

Allatostatin-A-2 (AST-A-2)

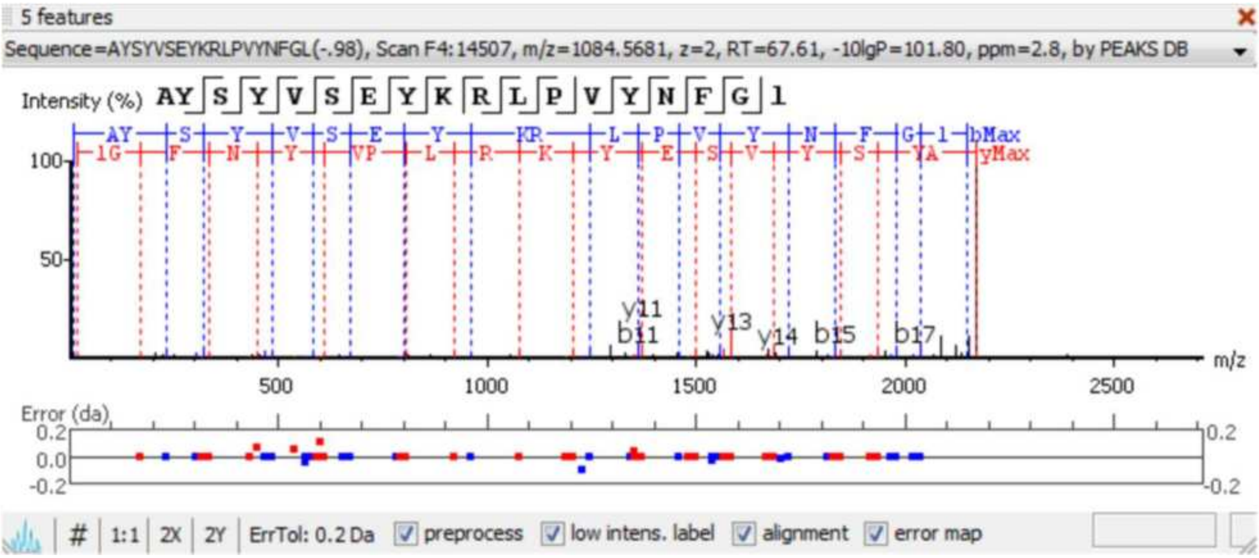

#	b	b-H2O	b-NH3	b (2+)	Seq	y	y-H2O	y-NH3	y (2+)	#
1	72.04	54.03	55.02	36.52	A					18
2	235.11	217.10	218.08	118.05	Y	2097.08	2079.07	2080.05	1049.04	17
3	322.14	304.13	305.11	161.57	S	1934.02	1916.01	1916.99	967.51	16
4	485.20	467.19	468.18	243.10	Y	1846.98	1828.97	1829.95	923.99	15
5	584.27	566.26	567.30	292.64	V	1683.93	1665.91	1666.90	842.46	14
6	671.30	653.29	654.28	336.15	S	1584.86	1566.83	1567.83	792.93	13
7	800.35	782.34	783.32	400.67	E	1497.82	1479.81	1480.80	749.41	12
8	963.40	945.40	946.38	482.20	Y	1368.78	1350.72	1351.75	684.89	11
9	1091.50	1073.49	1074.48	546.25	K	1205.72	1187.70	1188.69	603.24	10
10	1247.61	1229.69	1230.58	624.30	R	1077.63	1059.61	1060.59	539.25	9
11	1360.69	1342.68	1343.67	680.85	L	921.52	903.51	904.49	461.26	8
12	1457.75	1439.73	1440.72	729.37	P	808.44	790.42	791.41	404.72	7
13	1556.81	1538.80	1539.83	778.91	V	711.38	693.37	694.36	356.19	6
14	1719.88	1701.86	1702.86	860.44	Y	612.31	594.30	595.29	306.66	5
15	1833.92	1815.91	1816.90	917.46	N	449.17	431.24	432.22	225.13	4
16	1980.99	1962.98	1963.97	990.99	F	335.21	317.20	318.18	168.10	3
17	2038.01	2020.00	2020.99	1019.50	G	188.14	170.13	171.11	94.57	2
18					L(-.98)	131.12	113.11	114.09	66.06	1

Allatostatin-A-2$^{1-8}$ (AST-A-2$^{1-8}$)

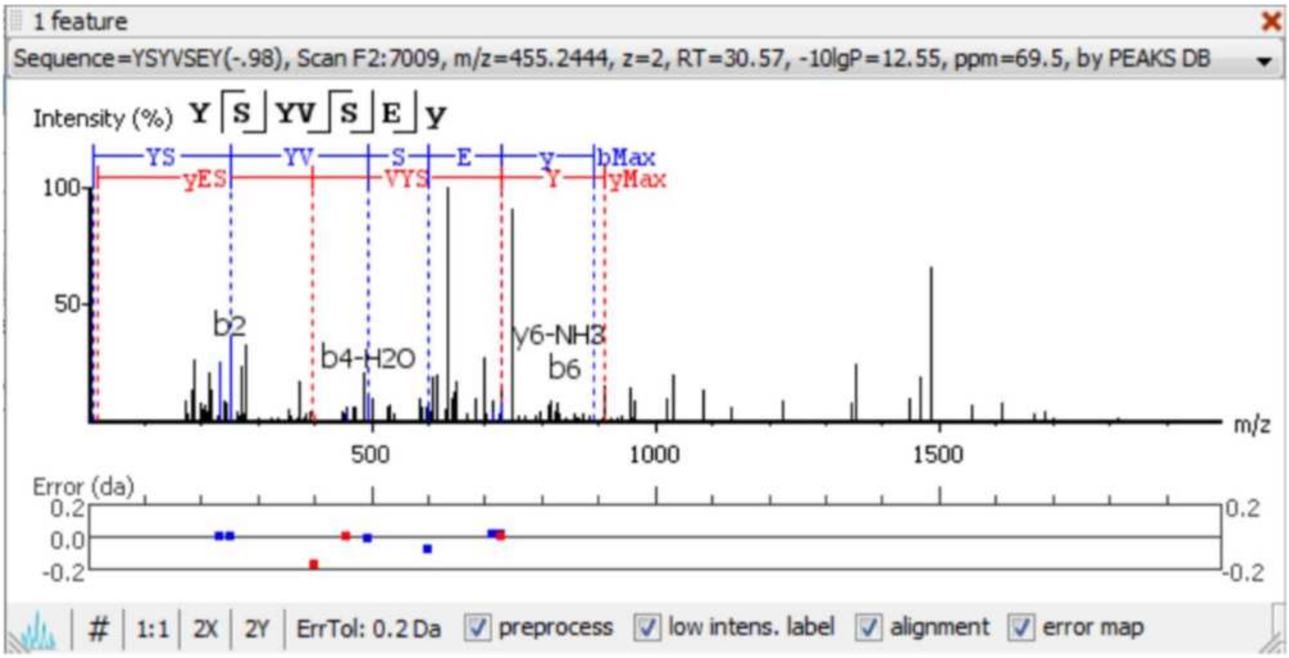

#	b	b-H2O	b-NH3	b (2+)	Seq	y	y-H2O	y-NH3	y (2+)	#
1	164.07	146.06	147.04	82.54	Y					7
2	251.10	233.09	234.08	126.05	S	746.34	728.32	729.30	373.67	6
3	414.17	396.16	397.14	207.58	Y	659.30	641.29	642.28	330.15	5
4	513.23	495.25	496.21	257.12	V	496.24	478.23	479.21	248.62	4
5	600.35	582.26	583.24	300.63	S	397.35	379.16	380.14	199.09	3
6	729.30	711.30	712.27	365.15	E	310.14	292.13	293.11	155.57	2
7					Y(-.98)	181.10	163.09	164.07	91.05	1

Allatostatin-A-2$^{11-19}$ (AST-A-2$^{11-19}$)

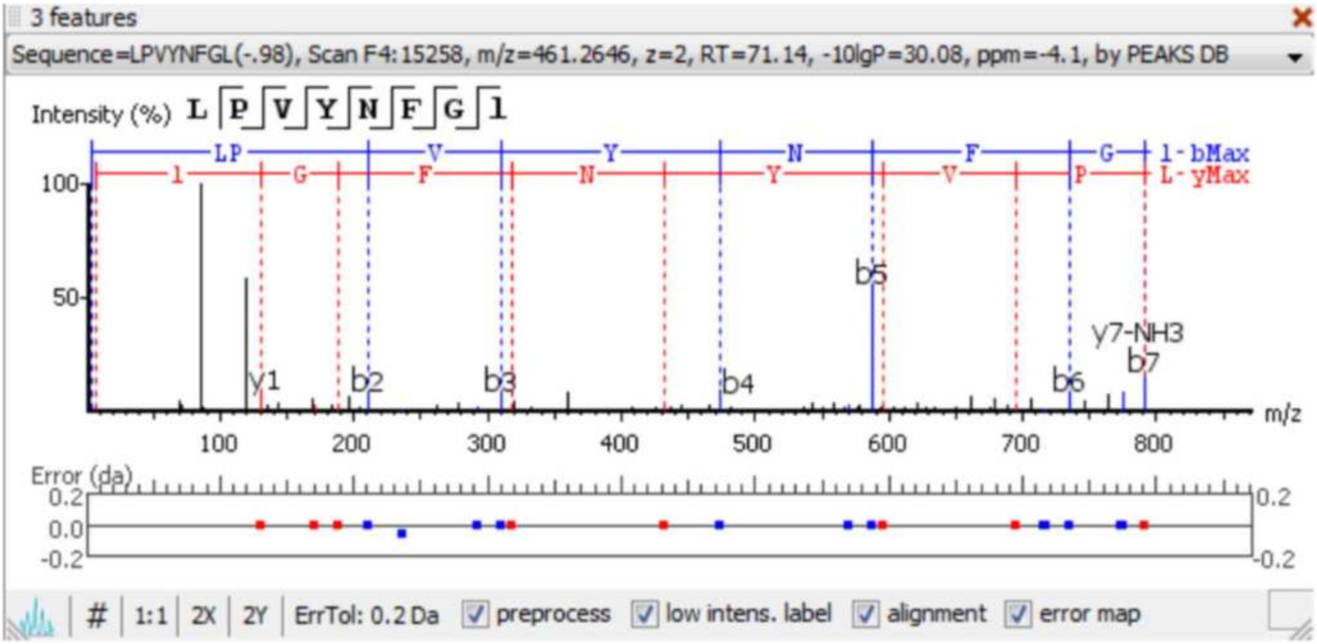

#	b	b-H2O	b-NH3	b (2+)	Seq	y	y-H2O	y-NH3	y (2+)	#
1	114.09	96.08	97.06	57.55	L					8
2	211.14	193.13	194.12	106.07	P	808.44	790.42	791.41	404.72	7
3	310.21	292.20	293.19	155.61	V	711.38	693.37	694.36	356.19	6
4	473.28	455.27	456.25	237.20	Y	612.31	594.30	595.29	306.66	5
5	587.32	569.31	570.29	294.16	N	449.25	431.24	432.22	225.13	4
6	734.39	716.38	717.36	367.69	F	335.21	317.20	318.18	168.10	3
7	791.41	773.40	774.38	396.20	G	188.14	170.13	171.11	94.57	2
8					L(-.98)	131.12	113.11	114.09	66.06	1

Allatostatin-A-3 (AST-A-3)

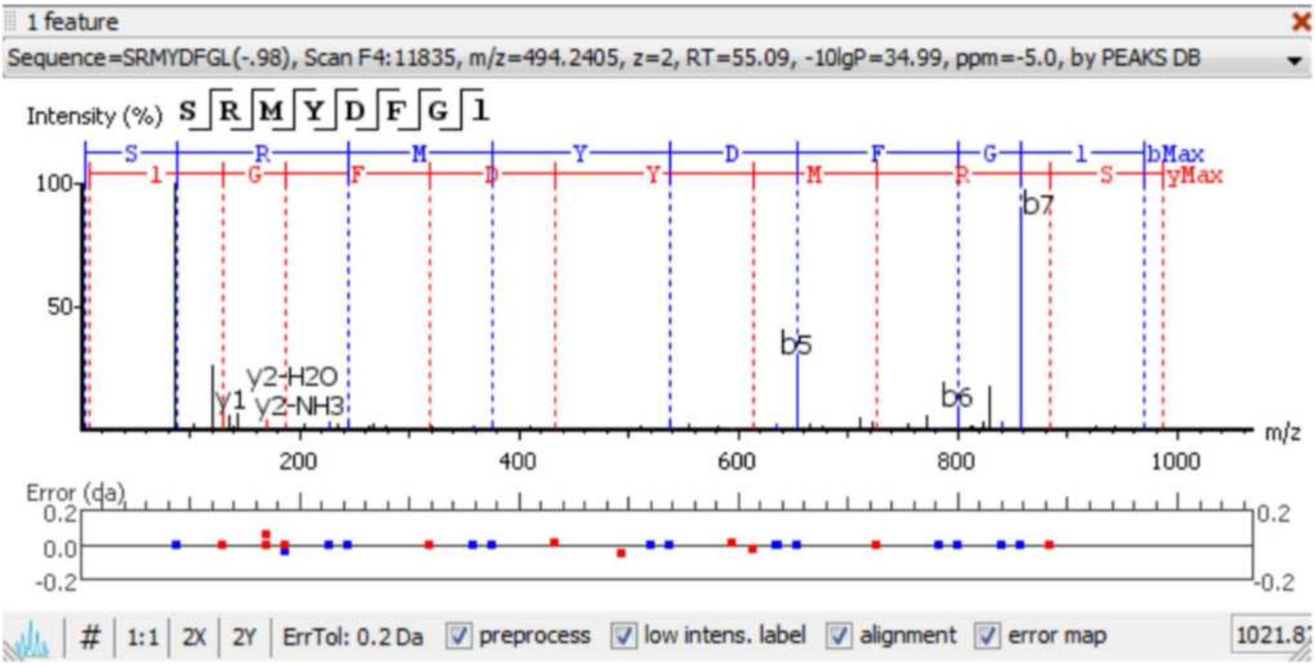

#	b	b-H2O	b-NH3	b (2+)	Seq	y	y-H2O	y-NH3	y (2+)	#
1	88.04	70.03	71.01	44.52	S					8
2	244.14	226.13	227.11	122.57	R	900.44	882.43	883.42	450.72	7
3	375.18	357.17	358.15	188.14	M	744.34	726.32	727.31	372.67	6
4	538.24	520.23	521.22	269.62	Y	613.33	595.27	596.27	307.15	5
5	653.27	635.26	636.25	327.14	D	450.23	432.20	433.21	225.62	4
6	800.34	782.33	783.32	400.67	F	335.21	317.20	318.18	168.10	3
7	857.36	839.34	840.34	429.18	G	188.14	170.06	171.11	94.57	2
8					L(-.98)	131.12	113.11	114.09	66.06	1

Allatostatin-A-4 (AST-A-4)

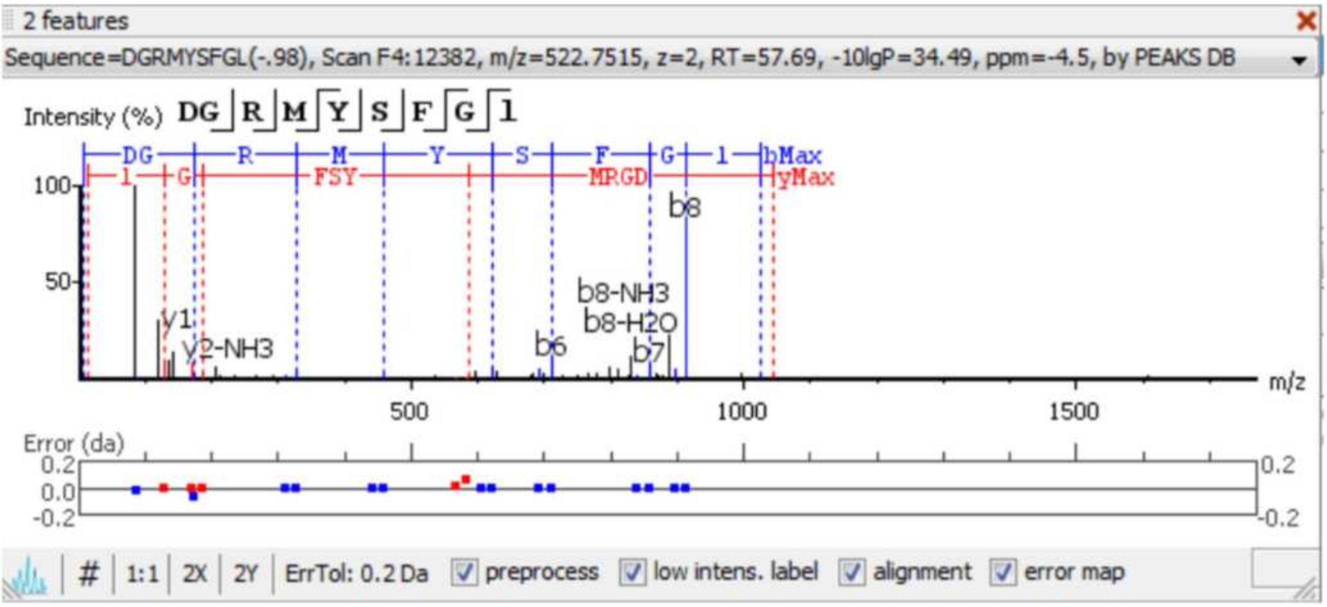

#	b	b-H2O	b-NH3	b (2+)	Seq	y	y-H2O	y-NH3	y (2+)	#
1	116.03	98.02	99.01	58.52	D					9
2	173.13	155.05	156.03	87.06	G	929.47	911.46	912.44	465.23	8
3	329.16	311.15	312.13	165.08	R	872.44	854.43	855.42	436.72	7
4	460.20	442.19	443.17	230.60	M	716.34	698.33	699.32	358.67	6
5	623.26	605.25	606.23	312.13	Y	585.24	567.27	568.28	293.15	5
6	710.29	692.28	693.27	355.65	S	422.24	404.23	405.21	211.62	4
7	857.36	839.35	840.33	429.18	F	335.21	317.20	318.18	168.10	3
8	914.38	896.37	897.36	457.69	G	188.14	170.13	171.11	94.57	2
9					L(-.98)	131.12	113.11	114.09	66.06	1

Allatostatin-A-5 (AST-A-5)

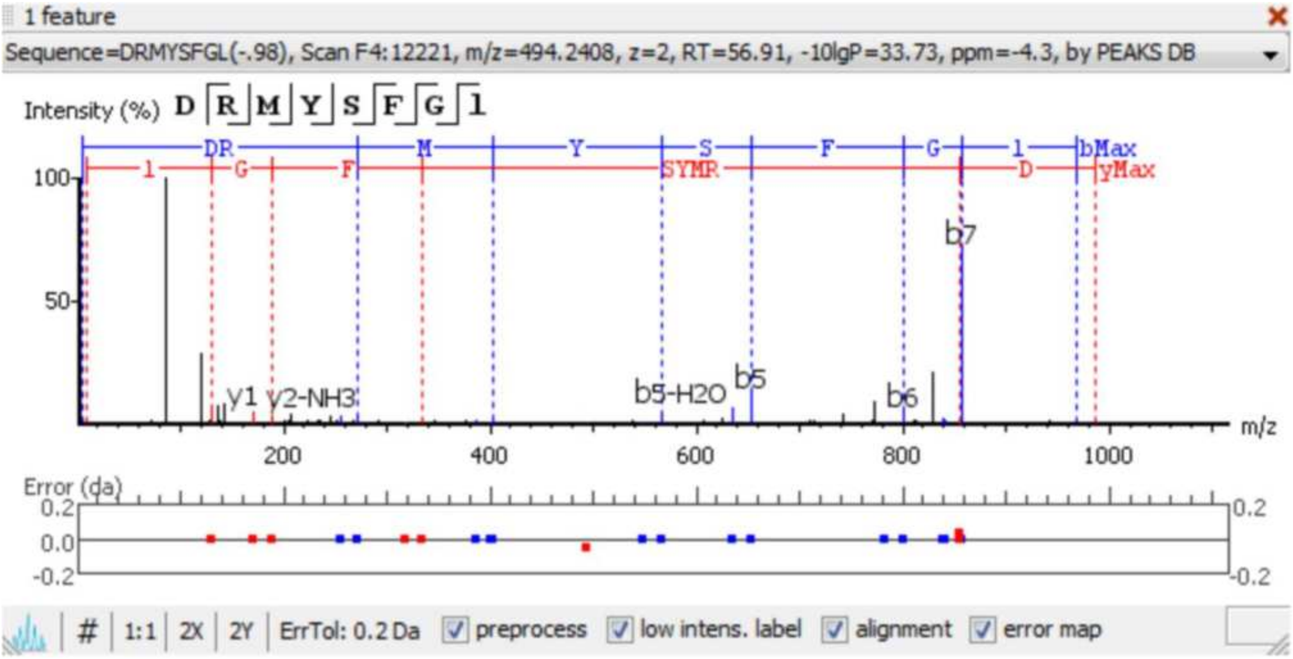

#	b	b-H2O	b-NH3	b (2+)	Seq	y	y-H2O	y-NH3	y (2+)	#
1	116.03	98.02	99.01	58.52	D					8
2	272.14	254.13	255.11	136.57	R	872.44	854.39	855.42	436.72	7
3	403.18	385.17	386.15	202.09	M	716.34	698.33	699.32	358.67	6
4	566.24	548.23	549.21	283.62	Y	585.30	567.29	568.28	293.15	5
5	653.27	635.26	636.24	327.14	S	422.24	404.23	405.21	211.62	4
6	800.34	782.33	783.31	400.67	F	335.21	317.20	318.18	168.10	3
7	857.36	839.35	840.34	429.18	G	188.14	170.13	171.11	94.57	2
8					L(-.98)	131.12	113.11	114.09	66.06	1

Allatostatin-A-6 (AST-A-6)

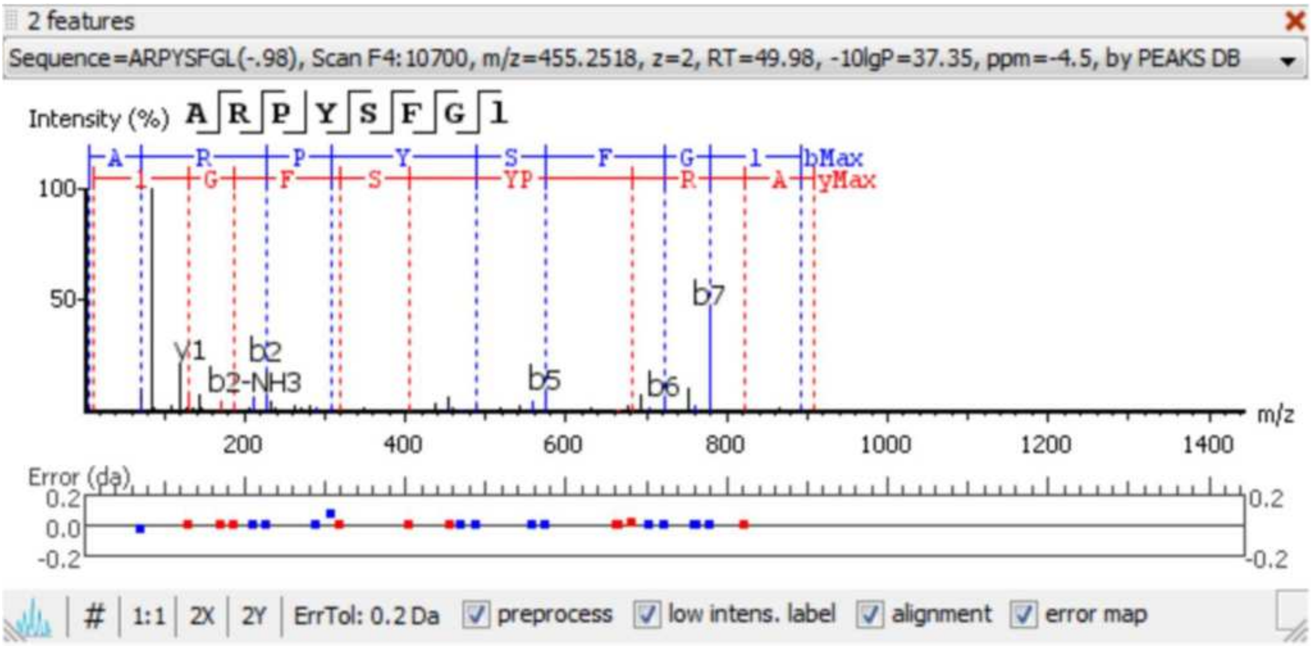

#	b	b-H2O	b-NH3	b (2+)	Seq	y	y-H2O	y-NH3	y (2+)	#
1	72.08	54.03	55.02	36.52	A					8
2	228.15	210.14	211.12	114.57	R	838.46	820.45	821.43	419.73	7
3	325.20	307.11	308.17	163.10	P	682.33	664.35	665.33	341.68	6
4	488.26	470.24	471.24	244.63	Y	585.30	567.29	568.28	293.15	5
5	575.29	557.28	558.27	288.15	S	422.24	404.23	405.21	211.62	4
6	722.36	704.35	705.34	361.68	F	335.21	317.20	318.18	168.10	3
7	779.38	761.37	762.36	390.19	G	188.14	170.13	171.11	94.57	2
8					L(-.98)	131.12	113.11	114.09	66.06	1

Allatostatin-A-7 (AST-A-7)

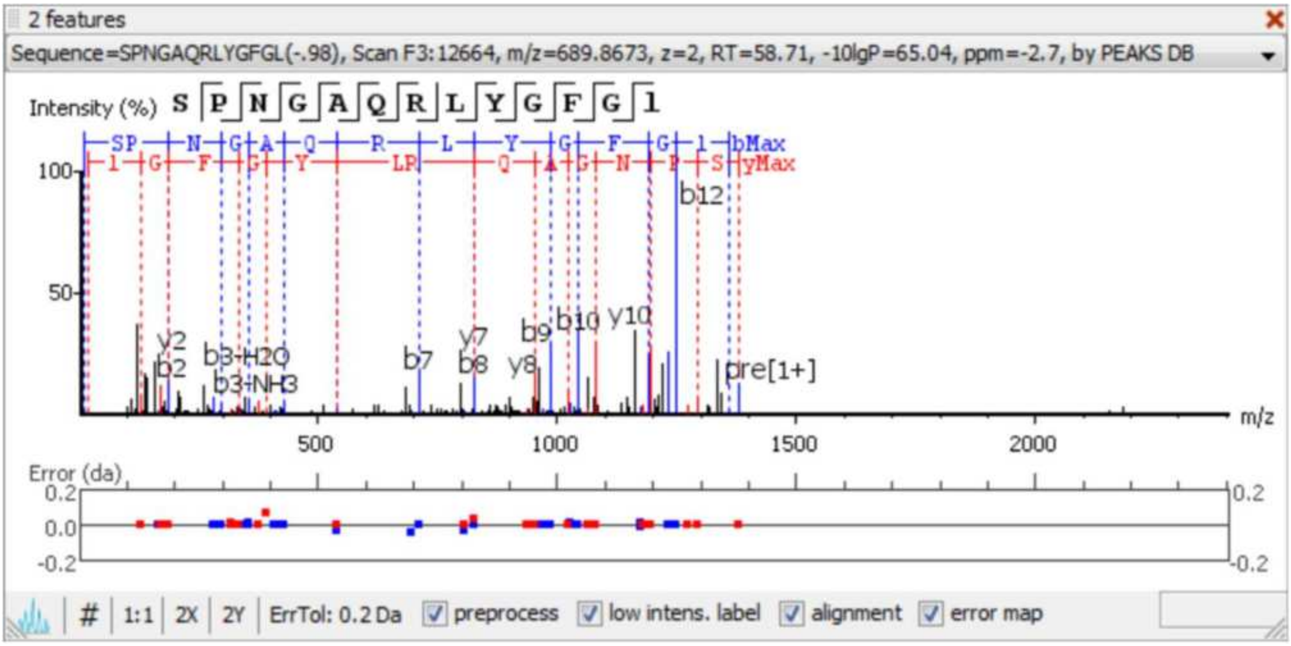

#	b	b-H2O	b-NH3	b (2+)	Seq	y	y-H2O	y-NH3	y (2+)	#
1	88.04	70.03	71.01	44.52	S					13
2	185.09	167.08	168.07	93.05	P	1291.69	1273.68	1274.64	646.35	12
3	299.14	281.12	282.11	150.07	N	1194.64	1176.63	1177.61	597.82	11
4	356.16	338.15	339.13	178.58	G	1080.60	1062.58	1063.57	540.80	10
5	427.19	409.18	410.17	214.10	A	1023.58	1005.56	1006.55	512.29	9
6	555.25	537.24	538.27	278.13	Q	952.54	934.53	935.51	476.77	8
7	711.35	693.34	694.33	356.16	R	824.44	806.47	807.42	412.74	7
8	824.44	806.43	807.42	412.72	L	668.38	650.37	651.35	334.69	6
9	987.50	969.49	970.48	494.25	Y	555.29	537.28	538.27	278.15	5
10	1044.52	1026.50	1027.50	522.76	G	392.15	374.22	375.20	196.61	4
11	1191.59	1173.57	1174.57	596.30	F	335.21	317.20	318.18	168.10	3
12	1248.61	1230.60	1231.60	624.81	G	188.14	170.13	171.11	94.57	2
13					L(-.98)	131.12	113.11	114.09	66.06	1

Allatostatin-A-8 (AST-A-8)

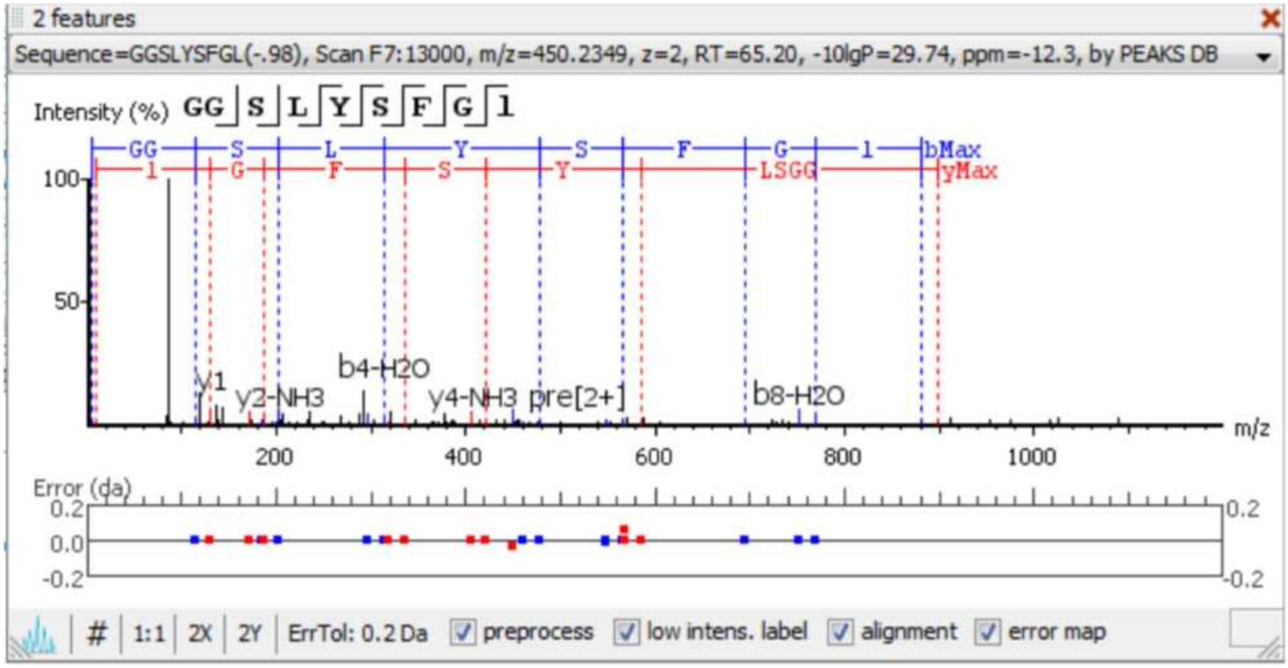

#	b	b-H2O	b-NH3	b (2+)	Seq	y	y-H2O	y-NH3	y (2+)	#
1	58.03	40.02	41.00	29.51	G					9
2	115.05	97.04	98.02	58.03	G	842.44	824.43	825.41	421.72	8
3	202.08	184.07	185.06	101.54	S	785.42	767.41	768.39	393.21	7
4	315.17	297.16	298.14	158.08	L	698.39	680.38	681.36	349.69	6
5	478.23	460.22	461.20	239.62	Y	585.30	567.23	568.28	293.15	5
6	565.26	547.25	548.26	283.13	S	422.24	404.23	405.21	211.62	4
7	712.33	694.32	695.30	356.67	F	335.21	317.20	318.18	168.10	3
8	769.35	751.34	752.33	385.18	G	188.14	170.13	171.11	94.57	2
9					L(-.98)	131.12	113.11	114.09	66.06	1

Allatostatin-A-9 (AST-A-9)

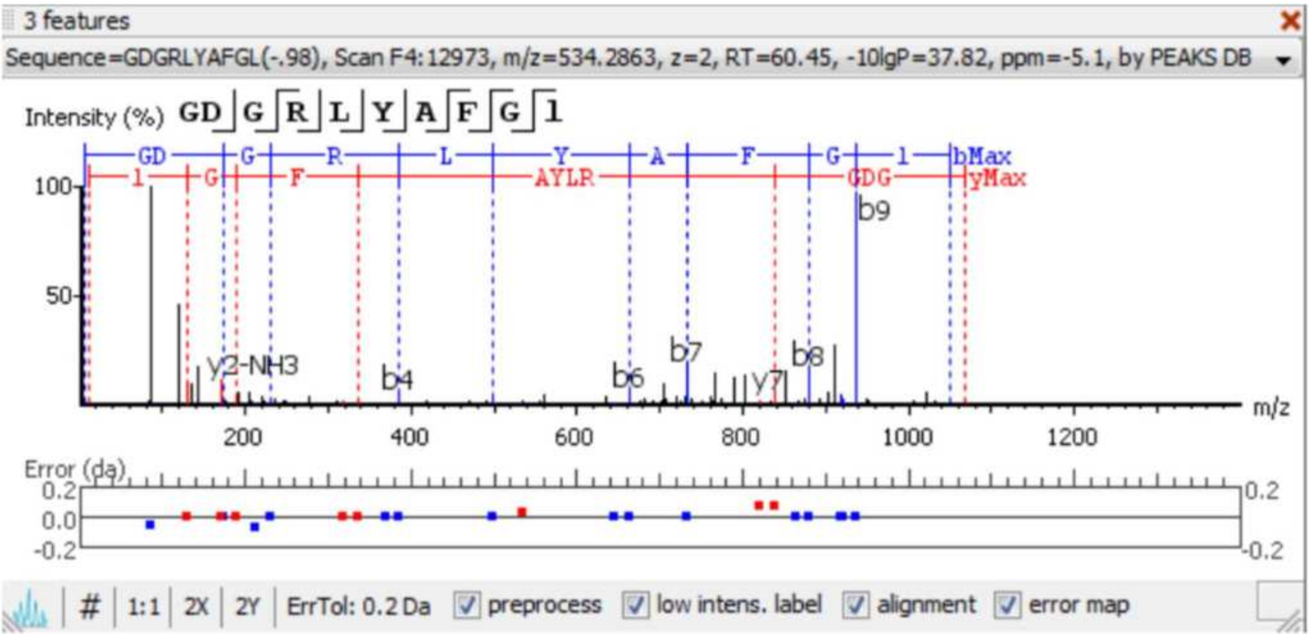

#	b	b-H2O	b-NH3	b (2+)	Seq	y	y-H2O	y-NH3	y (2+)	#
1	58.03	40.02	41.00	29.51	G					10
2	173.06	155.05	156.03	87.06	D	1010.54	992.53	993.51	505.77	9
3	230.08	212.10	213.16	115.54	G	895.52	877.50	878.49	448.26	8
4	386.18	368.17	369.15	193.59	R	838.42	820.48	821.39	419.75	7
5	499.26	481.25	482.24	250.13	L	682.39	664.38	665.37	341.70	6
6	662.33	644.32	645.30	331.66	Y	569.31	551.30	552.28	285.15	5
7	733.36	715.35	716.34	367.18	A	406.24	388.23	389.22	203.62	4
8	880.43	862.42	863.41	440.72	F	335.21	317.18	318.18	168.10	3
9	937.45	919.44	920.42	469.23	G	188.14	170.13	171.11	94.57	2
10					L(-.98)	131.12	113.11	114.09	66.06	1

Allatostatin-A-10 (AST-A-10)

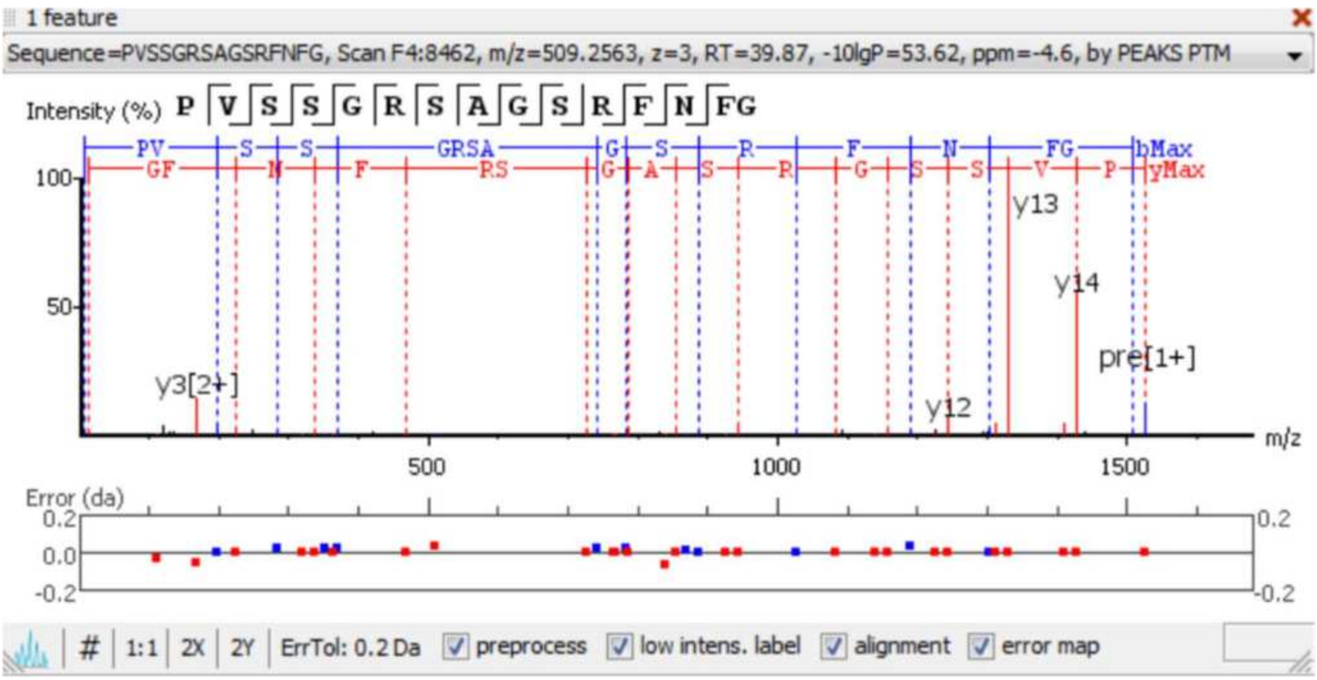

#	b	b-H2O	b-NH3	b (2+)	Seq	y	y-H2O	y-NH3	y (2+)	#
1	98.06	80.05	81.03	49.53	P					15
2	197.13	179.12	180.10	99.06	V	1428.69	1410.69	1411.67	714.85	14
3	284.14	266.15	267.13	142.58	S	1329.63	1311.62	1312.61	665.31	13
4	371.17	353.16	354.17	186.10	S	1242.60	1224.59	1225.57	621.80	12
5	428.21	410.20	411.19	214.61	G	1155.56	1137.55	1138.54	578.28	11
6	584.32	566.31	567.29	292.66	R	1098.54	1080.53	1081.51	549.77	10
7	671.35	653.34	654.32	336.17	S	942.44	924.44	925.42	471.72	9
8	742.36	724.37	725.36	371.69	A	855.41	837.40	838.46	428.21	8
9	799.41	781.40	782.35	400.20	G	784.37	766.36	767.35	392.69	7
10	886.44	868.41	869.41	443.72	S	727.35	709.34	710.33	364.17	6
11	1042.54	1024.53	1025.51	521.77	R	640.32	622.31	623.29	320.66	5
12	1189.57	1171.60	1172.58	595.30	F	484.22	466.21	467.19	242.61	4
13	1303.65	1285.64	1286.62	652.33	N	337.15	319.14	320.12	169.13	3
14	1450.72	1432.71	1433.69	725.86	F	223.11	205.10	206.08	112.09	2
15					G	76.04	58.03	59.01	38.52	1

Allatostatin-A-PP-2 (AST-A-PP-2)

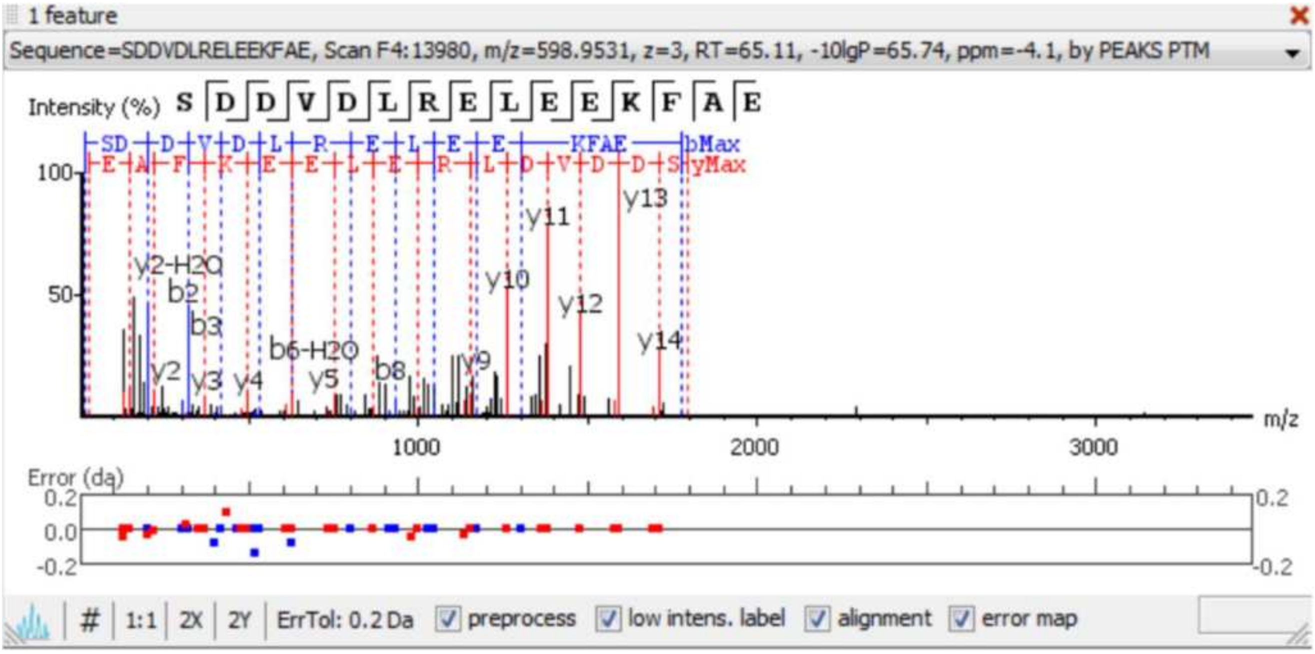

#	b	b-H2O	b-NH3	b (2+)	Seq	y	y-H2O	y-NH3	y (2+)	#
1	88.04	70.03	71.01	44.52	S					15
2	203.07	185.06	186.04	102.03	D	1707.81	1689.79	1690.78	854.40	14
3	318.09	300.08	301.07	159.55	D	1592.78	1574.77	1575.74	796.89	13
4	417.16	399.24	400.14	209.08	V	1477.75	1459.74	1460.73	739.38	12
5	532.19	514.18	515.31	266.59	D	1378.69	1360.67	1361.66	689.84	11
6	645.27	627.35	628.25	323.14	L	1263.65	1245.65	1246.63	632.33	10
7	801.37	783.36	784.35	401.19	R	1150.57	1132.60	1133.55	575.79	9
8	930.42	912.40	913.39	465.71	E	994.48	976.46	977.50	497.74	8
9	1043.50	1025.50	1026.47	522.25	L	865.43	847.42	848.40	433.12	7
10	1172.54	1154.53	1155.52	586.77	E	752.35	734.34	735.32	376.67	6
11	1301.58	1283.58	1284.56	651.29	E	623.30	605.29	606.28	312.12	5
12	1429.68	1411.67	1412.65	715.34	K	494.26	476.25	477.23	247.63	4
13	1576.75	1558.74	1559.72	788.87	F	366.17	348.16	349.14	183.58	3
14	1647.79	1629.78	1630.76	824.39	A	219.11	201.12	202.07	110.05	2
15					E	148.06	130.05	131.08	74.53	1

Allatostatin-A-11 (AST-A-11)

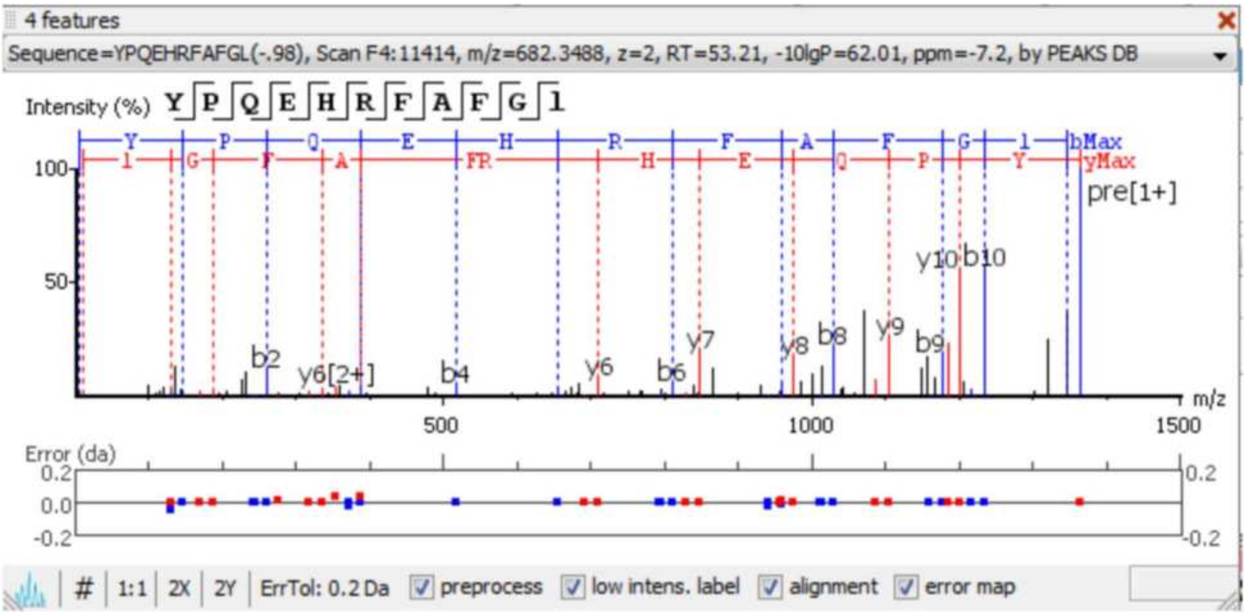

#	b	b-H2O	b-NH3	b (2+)	Seq	y	y-H2O	y-NH3	y (2+)	#
1	164.07	146.06	147.04	82.54	Y					11
2	261.12	243.11	244.10	131.12	P	1200.63	1182.61	1183.60	600.81	10
3	389.18	371.17	372.19	195.09	Q	1103.57	1085.57	1086.55	552.29	9
4	518.22	500.21	501.20	259.61	E	975.52	957.51	958.47	488.26	8
5	655.28	637.27	638.26	328.14	H	846.47	828.46	829.45	423.74	7
6	811.38	793.37	794.36	406.19	R	709.41	691.40	692.39	355.16	6
7	958.47	940.48	941.43	479.73	F	553.31	535.30	536.29	277.14	5
8	1029.49	1011.48	1012.47	515.25	A	406.24	388.23	389.18	203.62	4
9	1176.56	1158.55	1159.53	588.78	F	335.21	317.17	318.18	168.10	3
10	1233.58	1215.56	1216.56	617.29	G	188.14	170.13	171.11	94.57	2
11					L(-.98)	131.12	113.11	114.09	66.06	1

Allatostatin-A-PP-3 (AST-A-PP-3)

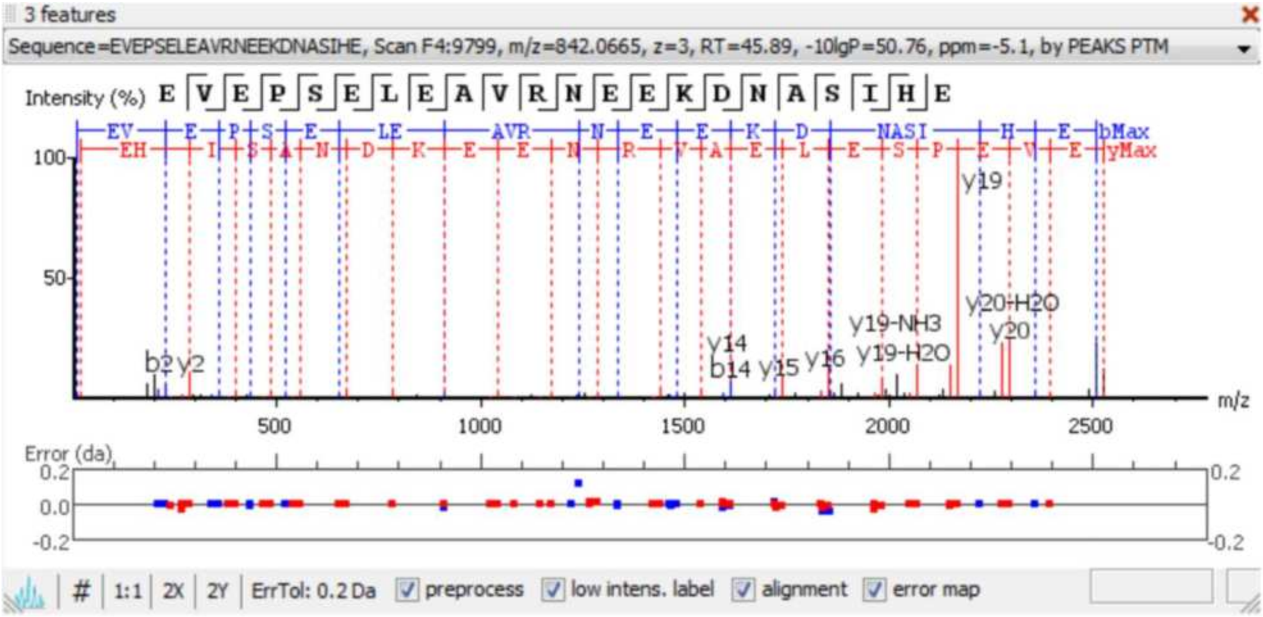

#	b	b-H2O	b-NH3	b (2+)	Seq	y	y-H2O	y-NH3	y (2+)	#
1	130.05	112.04	113.02	65.53	E					22
2	229.12	211.11	212.09	115.06	V	2395.15	2377.13	2378.11	1198.07	21
3	358.16	340.15	341.13	179.58	E	2296.07	2278.06	2279.04	1148.54	20
4	455.21	437.20	438.20	228.11	P	2167.02	2149.01	2150.01	1084.02	19
5	542.25	524.23	525.22	271.62	S	2069.98	2051.96	2052.95	1035.49	18
6	671.29	653.28	654.26	336.14	E	1982.96	1964.93	1965.95	991.97	17
7	784.37	766.36	767.35	392.69	L	1853.92	1835.89	1836.89	927.45	16
8	913.44	895.40	896.39	457.21	E	1740.83	1722.81	1723.81	870.91	15
9	984.45	966.44	967.43	492.73	A	1611.76	1593.75	1594.75	806.39	14
10	1083.52	1065.51	1066.49	542.26	V	1540.73	1522.72	1523.71	770.87	13
11	1239.51	1221.61	1222.60	620.31	R	1441.67	1423.67	1424.65	721.33	12
12	1353.67	1335.65	1336.66	677.33	N	1285.55	1267.53	1268.55	643.28	11
13	1482.71	1464.70	1465.70	741.85	E	1171.52	1153.51	1154.50	586.26	10
14	1611.76	1593.75	1594.75	806.38	E	1042.48	1024.47	1025.45	521.74	9
15	1739.85	1721.83	1722.81	870.42	K	913.44	895.43	896.41	457.22	8
16	1854.92	1836.89	1837.90	927.94	D	785.34	767.33	768.32	393.17	7
17	1968.92	1950.90	1951.89	984.96	N	670.32	652.30	653.28	335.66	6
18	2039.95	2021.94	2022.93	1020.48	A	556.27	538.25	539.25	278.64	5
19	2126.98	2108.97	2109.96	1063.99	S	485.24	467.23	468.21	243.13	4
20	2240.07	2222.06	2223.04	1120.53	I	398.20	380.19	381.18	199.60	3
21	2377.13	2359.13	2360.10	1189.06	H	285.12	267.11	268.13	143.06	2
22					E	148.06	130.05	131.03	74.53	1

Allatostatin-A-PP-4 (AST-A-PP-4)

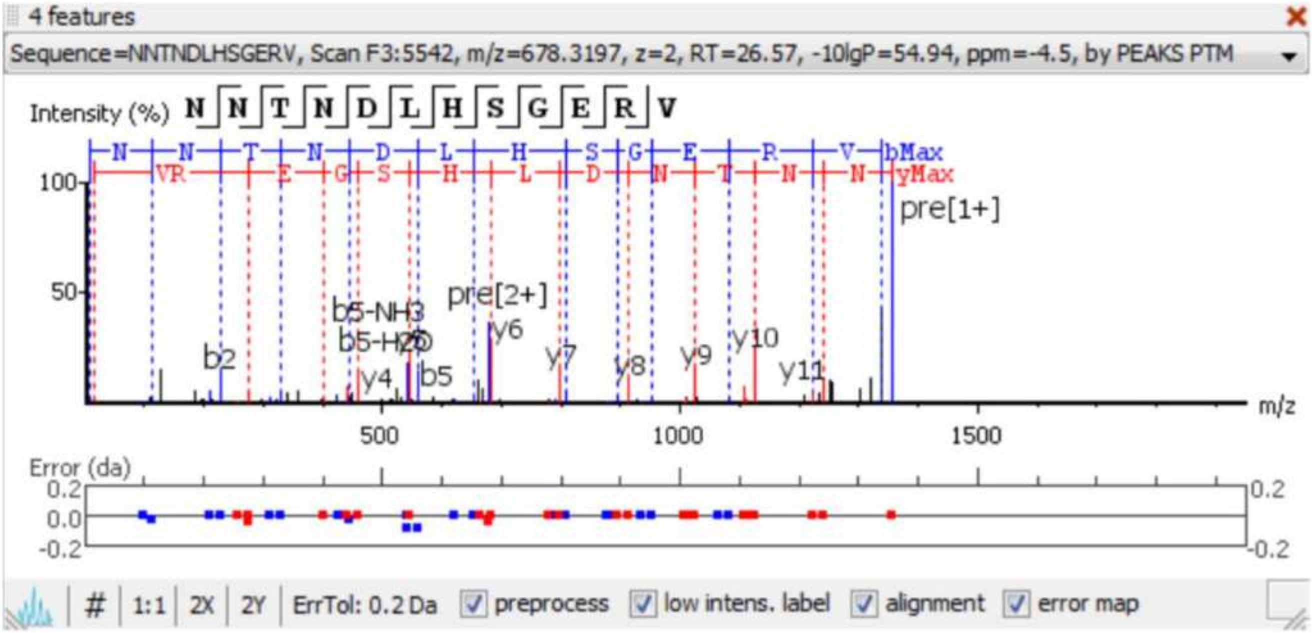

#	b	b-H2O	b-NH3	b (2+)	Seq	y	y-H2O	y-NH3	y (2+)	#
1	115.09	97.04	98.02	58.03	N					12
2	229.09	211.08	212.07	115.09	N	1241.59	1223.58	1224.56	621.29	11
3	330.14	312.13	313.11	165.57	T	1127.54	1109.53	1110.53	564.27	10
4	444.22	426.17	427.16	222.59	N	1026.50	1008.48	1009.47	513.75	9
5	559.31	541.30	542.18	280.11	D	912.45	894.44	895.43	456.73	8
6	672.30	654.28	655.27	336.65	L	797.43	779.42	780.40	399.21	7
7	809.35	791.34	792.33	405.18	H	684.34	666.33	667.32	342.67	6
8	896.38	878.38	879.36	448.69	S	547.28	529.27	530.26	274.19	5
9	953.41	935.40	936.39	477.20	G	460.24	442.23	443.22	230.63	4
10	1082.45	1064.44	1065.43	541.73	E	403.23	385.22	386.20	202.11	3
11	1238.54	1220.54	1221.51	619.78	R	274.19	256.18	257.16	137.59	2
12					V	118.09	100.08	101.06	59.54	1

Allatostatin-A-PP-4 (AST-A-PP-4) NSTNDLHSGERV-OH check

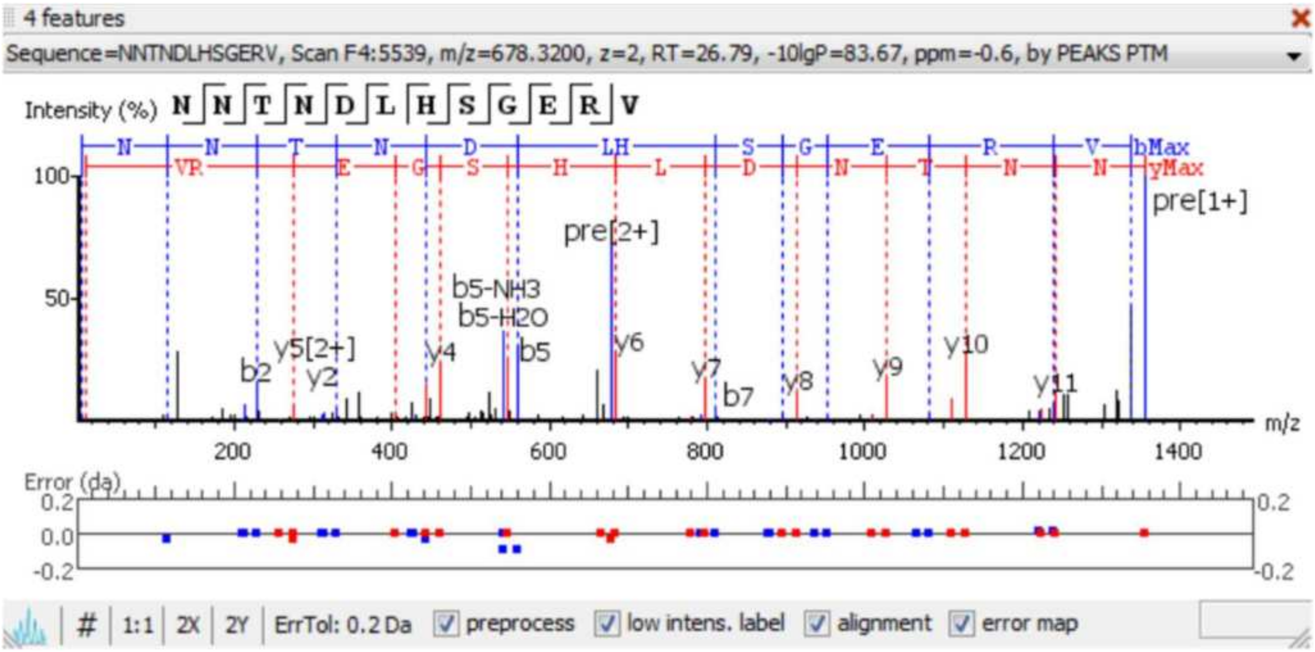

#	b	b-H2O	b-NH3	b (2+)	Seq	y	y-H2O	y-NH3	y (2+)	#
1	115.09	97.04	98.02	58.03	N					12
2	229.09	211.08	212.07	115.09	N	1241.59	1223.58	1224.56	621.29	11
3	330.14	312.13	313.11	165.57	T	1127.54	1109.53	1110.53	564.27	10
4	444.22	426.17	427.16	222.59	N	1026.50	1008.48	1009.47	513.75	9
5	559.31	541.30	542.18	280.11	D	912.45	894.44	895.43	456.73	8
6	672.30	654.28	655.27	336.65	L	797.43	779.42	780.40	399.21	7
7	809.35	791.34	792.33	405.18	H	684.34	666.33	667.32	342.67	6
8	896.38	878.38	879.36	448.69	S	547.28	529.27	530.26	274.19	5
9	953.41	935.40	936.39	477.20	G	460.24	442.23	443.22	230.63	4
10	1082.45	1064.44	1065.43	541.73	E	403.23	385.22	386.20	202.11	3
11	1238.54	1220.54	1221.51	619.78	R	274.19	256.18	257.16	137.59	2
12					V	118.09	100.08	101.06	59.54	1

Allatostatin-A-PP-5 (AST-A-PP-5)_partial

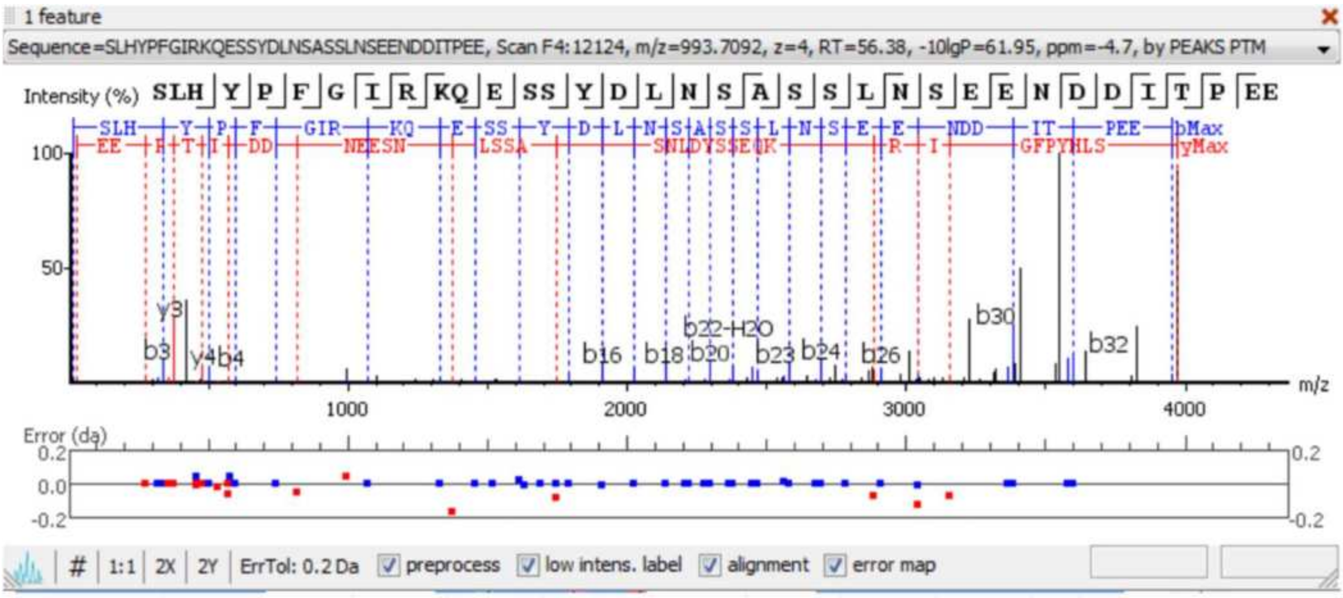

#	b	b-H2O	b-NH3	b (2+)	Seq	y	y-H2O	y-NH3	y (2+)	#
1	88.04	70.03	71.01	44.52	S					35
2	201.12	183.11	184.10	101.06	L	3884.77	3866.76	3867.75	1942.89	34
3	338.18	320.17	321.16	169.59	H	3771.69	3753.68	3754.66	1886.34	33
4	501.25	483.23	484.22	251.12	Y	3634.63	3616.62	3617.60	1817.82	32
5	598.30	580.24	581.27	299.65	P	3471.57	3453.56	3454.54	1736.28	31
6	745.37	727.36	728.34	373.18	F	3374.51	3356.50	3357.49	1687.76	30
7	802.39	784.38	785.36	401.69	G	3227.45	3209.44	3210.42	1614.22	29
8	915.47	897.46	898.45	458.19	I	3170.42	3152.41	3153.48	1585.71	28
9	1071.57	1053.56	1054.55	536.29	R	3057.34	3039.33	3040.45	1529.17	27
10	1199.67	1181.66	1182.64	600.33	K	2901.24	2883.23	2884.29	1451.12	26
11	1327.72	1309.72	1310.70	664.36	Q	2773.14	2755.13	2756.12	1387.07	25
12	1456.77	1438.76	1439.74	728.89	E	2645.09	2627.08	2628.06	1323.04	24
13	1543.80	1525.79	1526.78	772.40	S	2516.04	2498.03	2499.02	1258.52	23
14	1630.83	1612.82	1613.78	815.92	S	2429.01	2411.00	2411.98	1215.01	22
15	1793.89	1775.89	1776.87	897.45	Y	2341.98	2323.97	2324.95	1171.49	21
16	1908.94	1890.91	1891.90	954.96	D	2178.92	2160.91	2161.89	1089.96	20
17	2022.01	2004.00	2004.98	1011.50	L	2063.89	2045.88	2046.86	1032.44	19
18	2136.05	2118.04	2119.02	1068.53	N	1950.80	1932.79	1933.78	975.90	18
19	2223.08	2205.07	2206.06	1112.04	S	1836.76	1818.75	1819.73	918.88	17
20	2294.13	2276.11	2277.09	1147.56	A	1749.82	1731.72	1732.70	875.36	16
21	2381.16	2363.15	2364.13	1191.08	S	1678.69	1660.68	1661.67	839.85	15
22	2468.18	2450.17	2451.16	1234.59	S	1591.66	1573.65	1574.63	796.33	14
23	2581.28	2563.24	2564.24	1291.13	L	1504.63	1486.62	1487.60	752.81	13
24	2695.32	2677.29	2678.28	1348.16	N	1391.54	1373.53	1374.69	696.27	12
25	2782.35	2764.33	2765.32	1391.67	S	1277.50	1259.49	1260.47	639.25	11
26	2911.39	2893.38	2894.36	1456.19	E	1190.47	1172.46	1173.44	595.73	10
27	3040.45	3022.42	3023.40	1520.72	E	1061.43	1043.42	1044.40	531.24	9
28	3154.47	3136.46	3137.44	1577.74	N	932.38	914.37	915.36	466.69	8
29	3269.50	3251.49	3252.47	1635.27	D	818.40	800.33	801.31	409.67	7
30	3384.53	3366.51	3367.50	1692.77	D	703.31	685.30	686.29	352.16	6
31	3497.61	3479.60	3480.58	1749.30	I	588.29	570.28	571.33	294.64	5
32	3598.66	3580.64	3581.63	1799.83	T	475.20	457.19	458.19	238.10	4
33	3695.71	3677.70	3678.68	1848.35	P	374.16	356.15	357.13	187.58	3
34	3824.75	3806.74	3807.73	1912.88	E	277.10	259.09	260.08	139.05	2
35					E	148.06	130.05	131.03	74.53	1

Allatostatin-A-12 (AST-A-12)

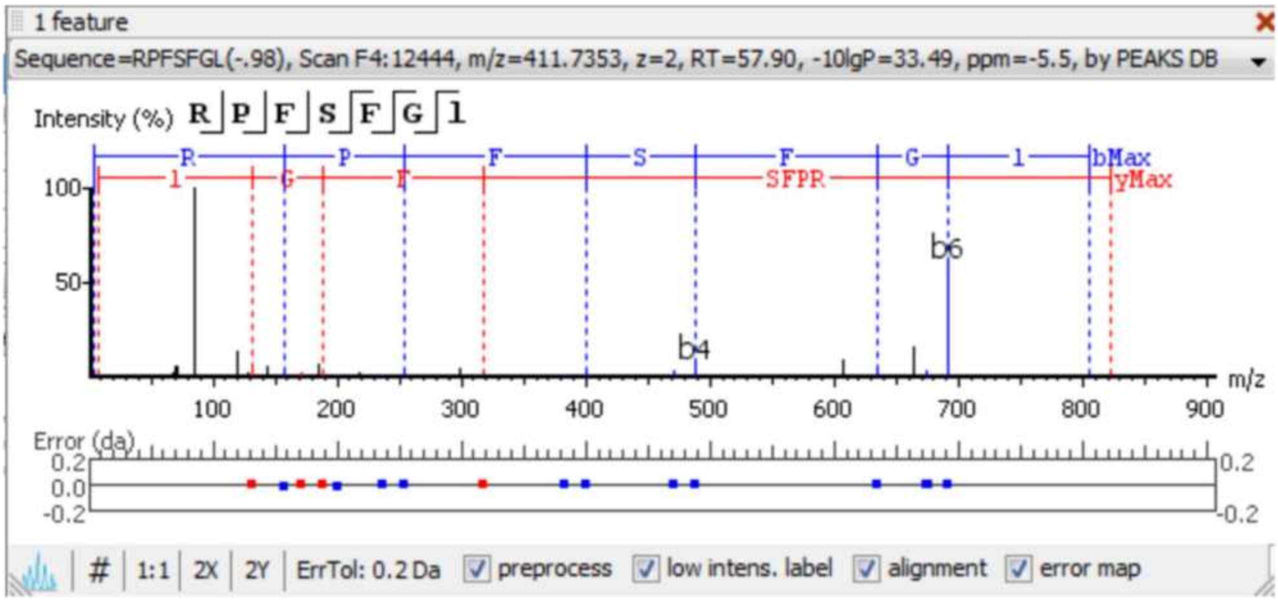

#	b	b-H2O	b-NH3	b (2+)	Seq	y	y-H2O	y-NH3	y (2+)	#
1	157.13	139.10	140.08	79.05	R					7
2	254.16	236.15	237.13	127.58	P	666.36	648.35	649.33	333.68	6
3	401.23	383.22	384.20	201.14	F	569.31	551.30	552.28	285.15	5
4	488.26	470.25	471.24	244.63	S	422.24	404.23	405.21	211.62	4
5	635.33	617.32	618.30	318.17	F	335.21	317.20	318.17	168.10	3
6	692.35	674.33	675.33	346.68	G	188.14	170.13	171.11	94.57	2
7					L(-.98)	131.12	113.11	114.09	66.06	1

Allatostatin-A-13 (AST-A-13)

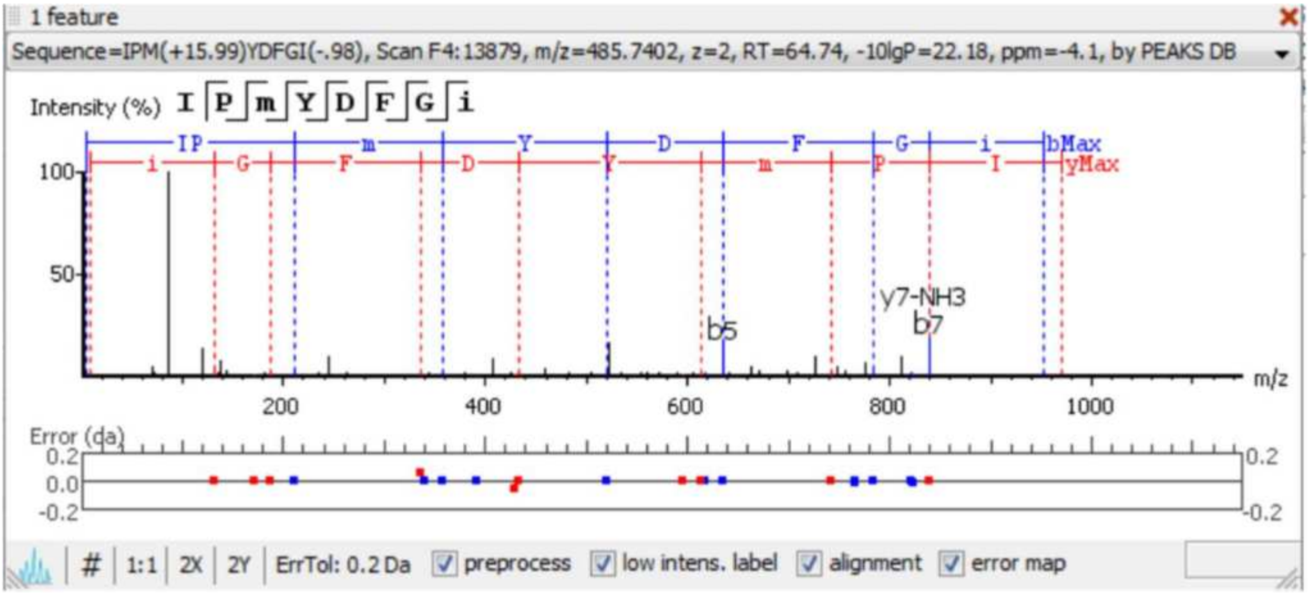

#	b	b-H2O	b-NH3	b (2+)	Seq	y	y-H2O	y-NH3	y (2+)	#
1	114.09	96.08	97.06	57.55	I					8
2	211.14	193.13	194.12	106.07	P	857.39	839.38	840.36	429.25	7
3	358.18	340.17	341.15	179.59	M(+15.99)	760.33	742.32	743.31	380.67	6
4	521.24	503.23	504.22	261.12	Y	613.30	595.29	596.27	307.15	5
5	636.27	618.26	619.24	318.64	D	450.23	432.22	433.21	225.62	4
6	783.34	765.33	766.33	392.17	F	335.14	317.20	318.18	168.10	3
7	840.36	822.35	823.35	420.68	G	188.14	170.13	171.11	94.57	2
8					I(-.98)	131.12	113.11	114.09	66.06	1

Allatostatine-B/Myoinhibitory peptide-PP-1, (Ast-B/MIP-PP-1)

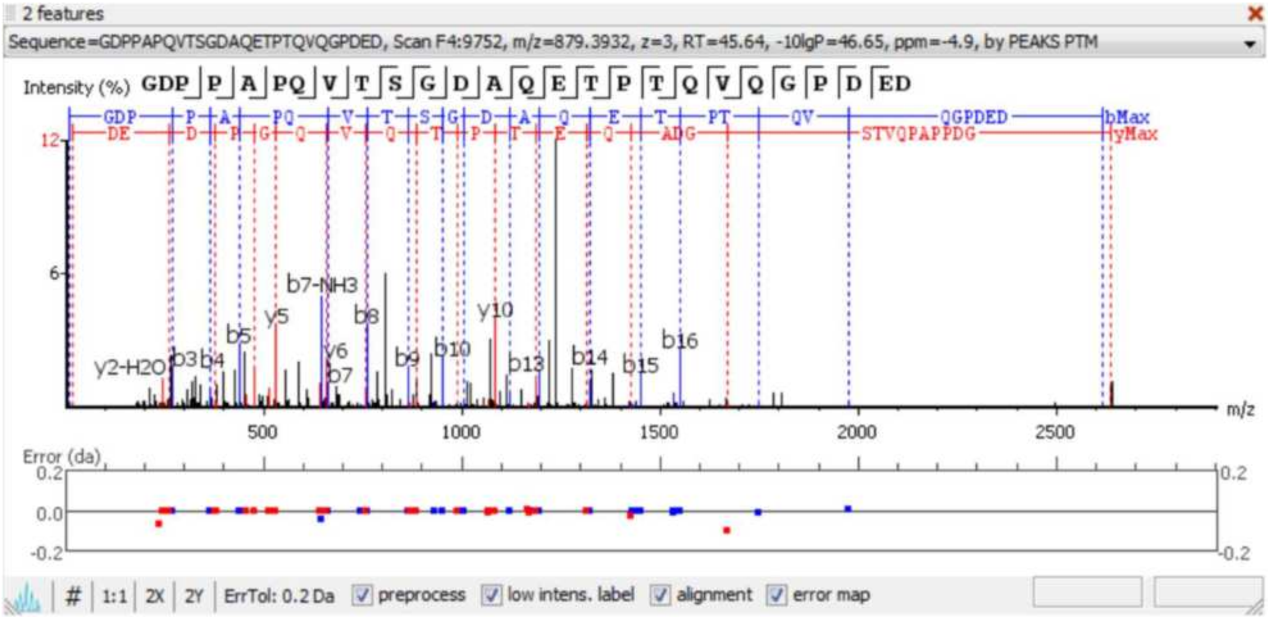

#	b	b-H2O	b-NH3	b (2+)	Seq	y	y-H2O	y-NH3	y (2+)	#
1	58.03	40.02	41.00	29.51	G					26
2	173.06	155.05	156.03	87.03	D	2579.14	2561.13	2562.11	1290.07	25
3	270.11	252.10	253.08	135.55	P	2464.11	2446.10	2447.08	1232.56	24
4	367.16	349.15	350.13	184.08	P	2367.06	2349.05	2350.03	1184.03	23
5	438.20	420.19	421.17	219.60	A	2270.01	2251.99	2252.98	1135.50	22
6	535.25	517.24	518.22	268.13	P	2198.97	2180.96	2181.94	1099.98	21
7	663.31	645.30	646.32	332.16	Q	2101.92	2083.91	2084.89	1051.46	20
8	762.38	744.37	745.35	381.69	V	1973.86	1955.85	1956.83	987.43	19
9	863.43	845.42	846.40	432.21	T	1874.79	1856.78	1857.76	937.89	18
10	950.46	932.44	933.43	475.73	S	1773.74	1755.73	1756.71	887.38	17
11	1007.48	989.48	990.45	504.24	G	1686.71	1668.70	1669.78	843.85	16
12	1122.50	1104.50	1105.48	561.75	D	1629.69	1611.68	1612.66	815.34	15
13	1193.54	1175.53	1176.52	597.27	A	1514.66	1496.65	1497.63	757.83	14
14	1321.60	1303.59	1304.58	661.30	Q	1443.62	1425.64	1426.60	722.31	13
15	1450.65	1432.63	1433.62	725.82	E	1315.56	1297.55	1298.54	658.28	12
16	1551.69	1533.68	1534.68	776.35	T	1186.53	1168.50	1169.51	593.76	11
17	1648.75	1630.73	1631.72	824.87	P	1085.48	1067.47	1068.46	543.24	10
18	1749.80	1731.78	1732.77	875.40	T	988.42	970.41	971.39	494.71	9
19	1877.85	1859.84	1860.82	939.43	Q	887.38	869.36	870.35	444.19	8
20	1976.91	1958.91	1959.89	988.96	V	759.32	741.30	742.29	380.16	7
21	2104.98	2086.97	2087.95	1052.99	Q	660.25	642.24	643.22	330.62	6
22	2162.00	2143.99	2144.97	1081.50	G	532.19	514.18	515.16	266.59	5
23	2259.05	2241.04	2242.03	1130.03	P	475.17	457.16	458.14	238.15	4
24	2374.08	2356.07	2357.05	1187.54	D	378.11	360.10	361.09	189.56	3
25	2503.12	2485.11	2486.10	1252.06	E	263.09	245.08	246.06	132.04	2
26					D	134.04	116.03	117.02	67.52	1

Allatostatine-B/Myoinhibitory peptide-1, (MIP-1)_partial

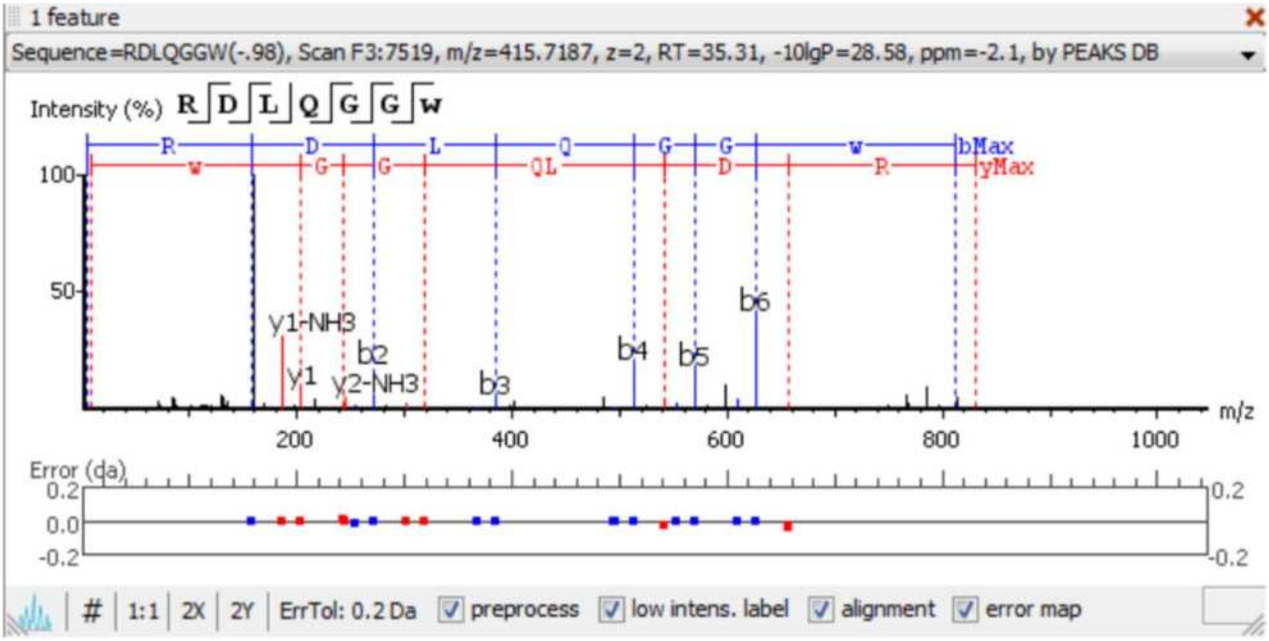

#	b	b-H2O	b-NH3	b (2+)	Seq	y	y-H2O	y-NH3	y (2+)	#
1	157.11	139.10	140.08	79.05	R					7
2	272.14	254.13	255.12	136.57	D	674.33	656.34	657.34	337.66	6
3	385.22	367.21	368.19	193.11	L	559.30	541.29	542.30	280.15	5
4	513.28	495.27	496.25	257.14	Q	446.21	428.20	429.19	223.61	4
5	570.30	552.29	553.27	285.65	G	318.16	300.15	301.13	159.58	3
6	627.32	609.31	610.29	314.16	G	261.13	243.11	244.11	131.07	2
7					W(-.98)	204.11	186.10	187.09	102.56	1

Allatostatine-B/Myoinhibitory peptide-2, (MIP-2)

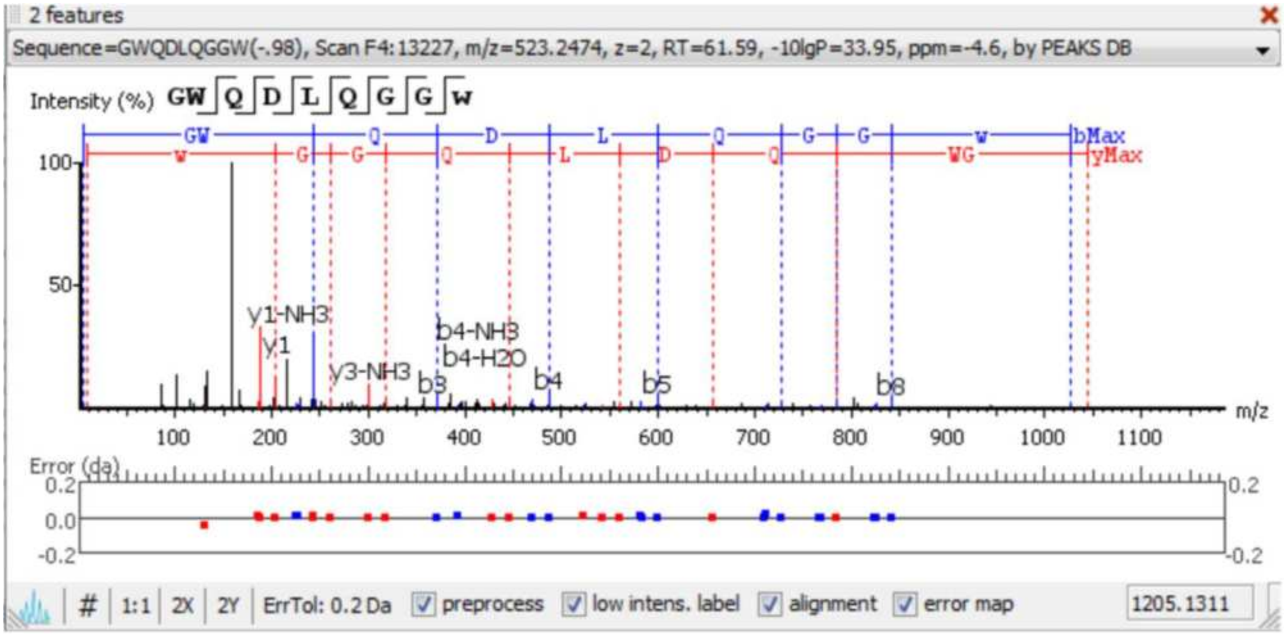

#	b	b-H2O	b-NH3	b (2+)	Seq	y	y-H2O	y-NH3	y (2+)	#
1	58.03	40.02	41.00	29.51	G					9
2	244.11	226.08	227.07	122.55	W	988.46	970.45	971.44	494.73	8
3	372.17	354.16	355.14	186.58	Q	802.38	784.37	785.36	401.69	7
4	487.19	469.18	470.17	244.11	D	674.33	656.32	657.30	337.66	6
5	600.28	582.26	583.26	300.64	L	559.30	541.29	542.27	280.15	5
6	728.34	710.33	711.28	364.67	Q	446.21	428.20	429.19	223.61	4
7	785.36	767.35	768.33	393.16	G	318.16	300.15	301.13	159.58	3
8	842.38	824.37	825.36	421.69	G	261.13	243.11	244.11	131.12	2
9					W(-.98)	204.11	186.09	187.09	102.56	1

Allatostatine-B/Myoinhibitory peptide-3, (MIP-3)

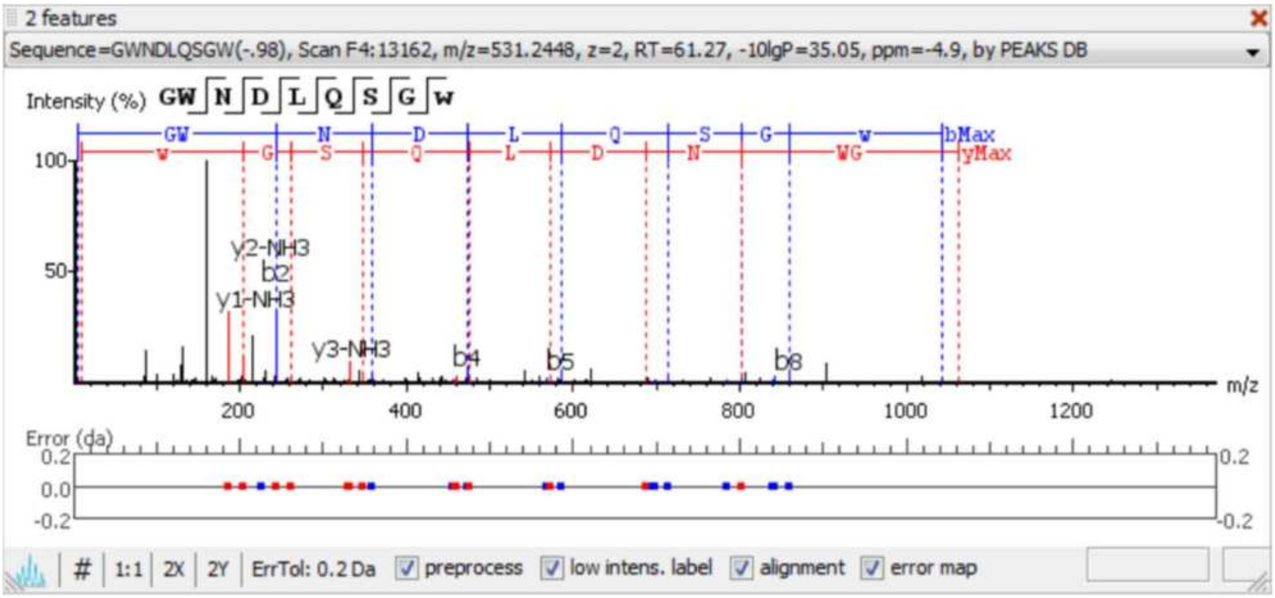

#	b	b-H2O	b-NH3	b (2+)	Seq	y	y-H2O	y-NH3	y (2+)	#
1	58.03	40.02	41.00	29.51	G					9
2	244.11	226.10	227.08	122.55	W	1004.46	986.45	987.43	502.73	8
3	358.15	340.14	341.12	179.58	N	818.38	800.37	801.35	409.69	7
4	473.18	455.17	456.15	237.09	D	704.34	686.32	687.31	352.67	6
5	586.26	568.25	569.23	293.63	L	589.31	571.30	572.28	295.15	5
6	714.32	696.31	697.29	357.66	Q	476.23	458.22	459.20	238.61	4
7	801.35	783.34	784.33	401.18	S	348.17	330.16	331.14	174.58	3
8	858.37	840.36	841.35	429.69	G	261.13	243.12	244.11	131.07	2
9					W(-.98)	204.11	186.10	187.09	102.56	1

Allatostatine-B/Myoinhibitory peptide-4, (MIP-4)

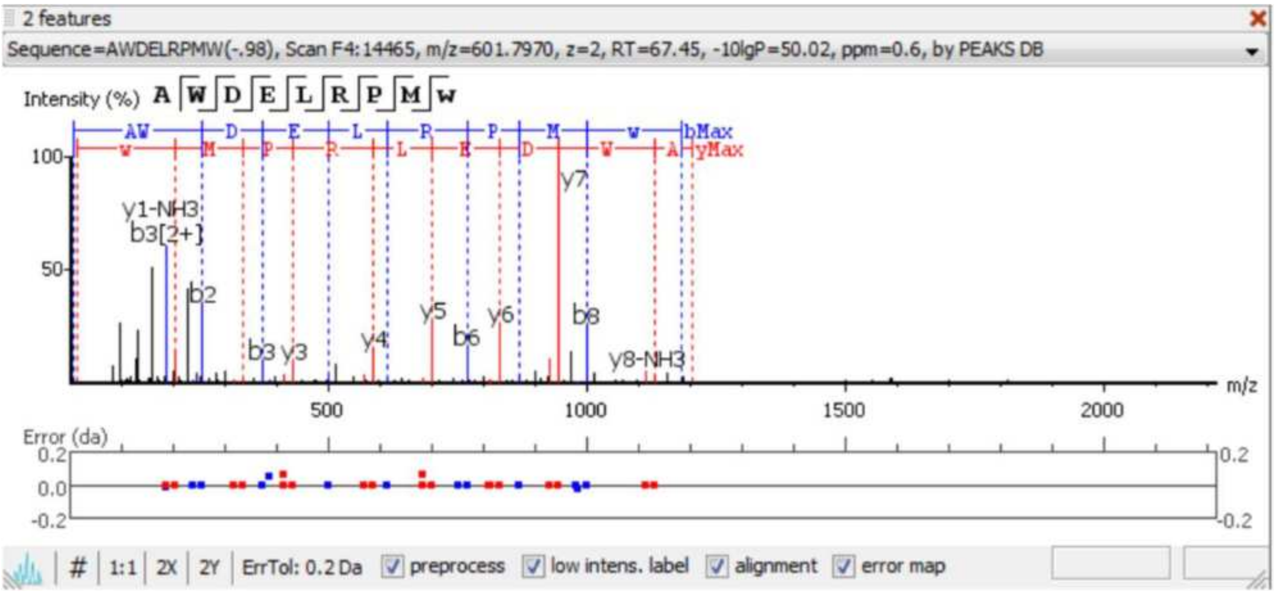

#	b	b-H2O	b-NH3	b (2+)	Seq	y	y-H2O	y-NH3	y (2+)	#
1	72.04	54.03	55.02	36.52	A					9
2	258.12	240.11	241.10	129.56	W	1131.54	1113.53	1114.51	566.27	8
3	373.15	355.14	356.12	187.09	D	945.46	927.45	928.44	473.23	7
4	502.19	484.18	485.17	251.60	E	830.43	812.42	813.41	415.72	6
5	615.28	597.27	598.25	308.14	L	701.39	683.32	684.36	351.20	5
6	771.38	753.37	754.35	386.13	R	588.31	570.30	571.28	294.65	4
7	868.43	850.42	851.40	434.72	P	432.21	414.13	415.18	216.60	3
8	999.47	981.46	982.47	500.24	M	335.15	317.14	318.13	168.08	2
9					W(-.98)	204.11	186.10	187.09	102.56	1

Allatostatine-B/Myoinhibitory peptide-5, (MIP-5)

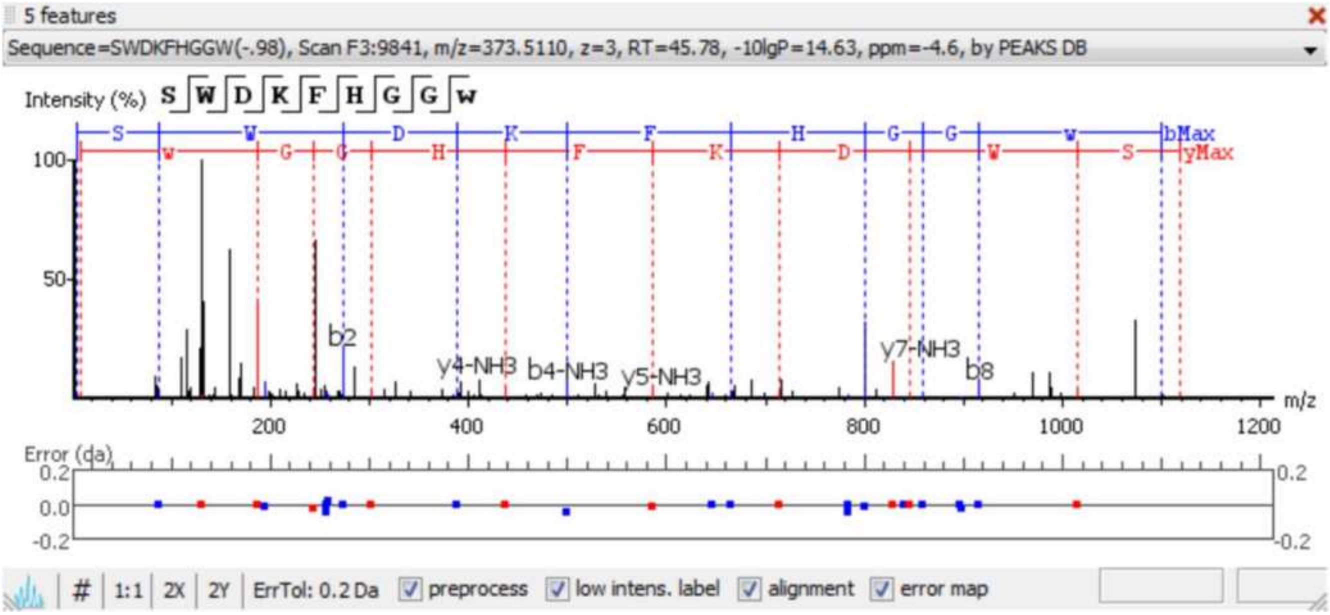

#	b	b-H2O	b-NH3	b (2+)	Seq	y	y-H2O	y-NH3	y (2+)	#
1	88.04	70.03	71.01	44.52	S					9
2	274.12	256.11	257.14	137.56	W	1031.48	1013.47	1014.46	516.24	8
3	389.15	371.14	372.12	195.09	D	845.41	827.40	828.38	423.20	7
4	517.24	499.23	500.26	259.12	K	730.38	712.37	713.35	365.69	6
5	664.31	646.30	647.28	332.65	F	602.28	584.27	585.27	301.64	5
6	801.37	783.36	784.39	401.18	H	455.22	437.20	438.19	228.11	4
7	858.39	840.38	841.36	429.69	G	318.16	300.15	301.13	159.58	3
8	915.41	897.40	898.40	458.21	G	261.13	243.12	244.11	131.07	2
9					W(-.98)	204.11	186.10	187.09	102.56	1

Allatostatine-B/Myoinhibitory peptide-PP-2, (MIP-PP-2)

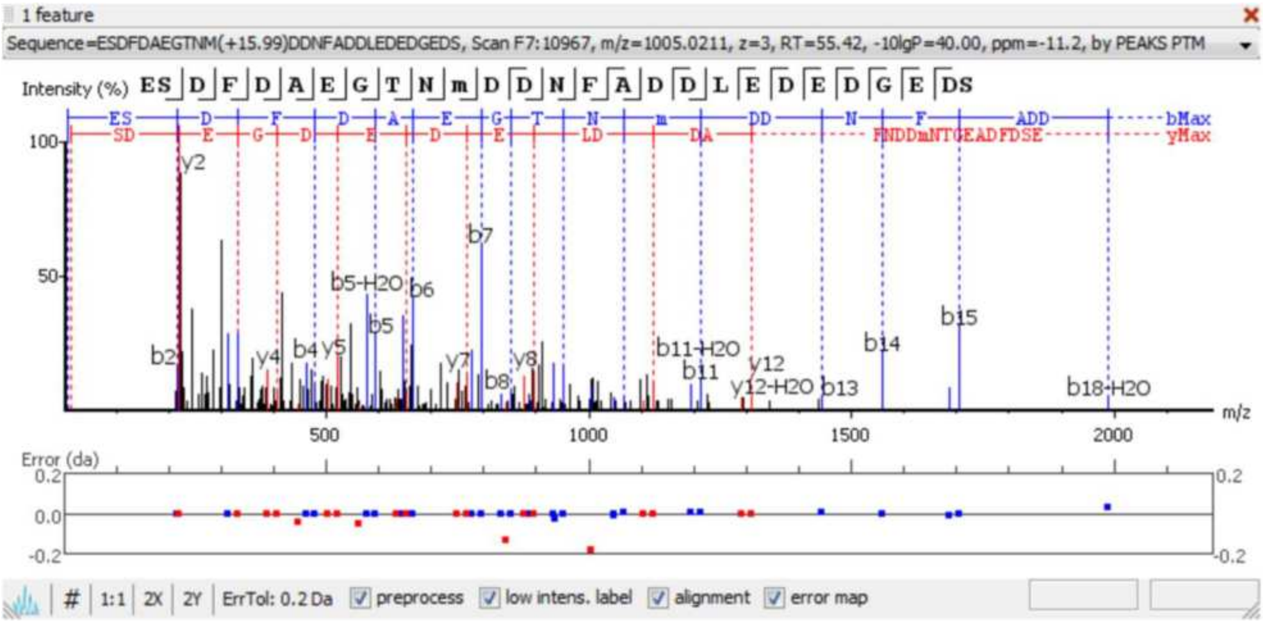

#	b	b-H2O	b-NH3	b (2+)	Seq	y	y-H2O	y-NH3	y (2+)	#
1	130.05	112.04	113.02	65.53	E					27
2	217.08	199.07	200.06	109.04	S	2884.00	2865.99	2866.97	1442.50	26
3	332.11	314.10	315.08	166.55	D	2796.97	2778.96	2779.94	1398.98	25
4	479.18	461.17	462.15	240.09	F	2681.94	2663.93	2664.92	1341.47	24
5	594.20	576.19	577.18	297.60	D	2534.87	2516.86	2517.85	1267.94	23
6	665.24	647.23	648.21	333.12	A	2419.85	2401.84	2402.82	1210.42	22
7	794.29	776.27	777.26	397.64	E	2348.81	2330.80	2331.78	1174.91	21
8	851.31	833.29	834.28	426.15	G	2219.77	2201.76	2202.74	1110.38	20
9	952.36	934.35	935.35	476.68	T	2162.75	2144.74	2145.72	1081.87	19
10	1066.39	1048.38	1049.38	533.70	N	2061.70	2043.69	2044.67	1031.35	18
11	1213.42	1195.41	1196.40	607.22	M(+15.99)	1947.66	1929.65	1930.63	974.33	17
12	1328.46	1310.45	1311.43	664.73	D	1800.62	1782.61	1783.59	900.81	16
13	1443.48	1425.48	1426.46	722.24	D	1685.59	1667.58	1668.57	843.43	15
14	1557.53	1539.52	1540.50	779.26	N	1570.57	1552.56	1553.54	785.78	14
15	1704.60	1686.59	1687.58	852.80	F	1456.52	1438.51	1439.50	728.76	13
16	1775.63	1757.62	1758.61	888.32	A	1309.46	1291.45	1292.43	655.23	12
17	1890.66	1872.65	1873.63	945.83	D	1238.42	1220.41	1221.39	619.71	11
18	2005.69	1987.64	1988.66	1003.34	D	1123.39	1105.38	1106.36	562.25	10
19	2118.77	2100.76	2101.75	1059.89	L	1008.36	990.35	991.34	504.68	9
20	2247.81	2229.80	2230.79	1124.41	E	895.28	877.27	878.25	448.18	8
21	2362.84	2344.83	2345.81	1181.92	D	766.24	748.23	749.21	383.62	7
22	2491.88	2473.87	2474.86	1246.44	E	651.21	633.20	634.18	326.11	6
23	2606.91	2588.90	2589.88	1303.96	D	522.17	504.16	505.14	261.58	5
24	2663.93	2645.92	2646.91	1332.47	G	407.14	389.13	390.11	204.07	4
25	2792.98	2774.96	2775.95	1396.99	E	350.12	332.11	333.09	175.56	3
26	2908.00	2889.99	2890.98	1454.50	D	221.08	203.07	204.05	111.04	2
27					S	106.05	88.04	89.02	53.52	1

Allatostatine-B/Myoinhibitory peptide-6, (MIP-6)

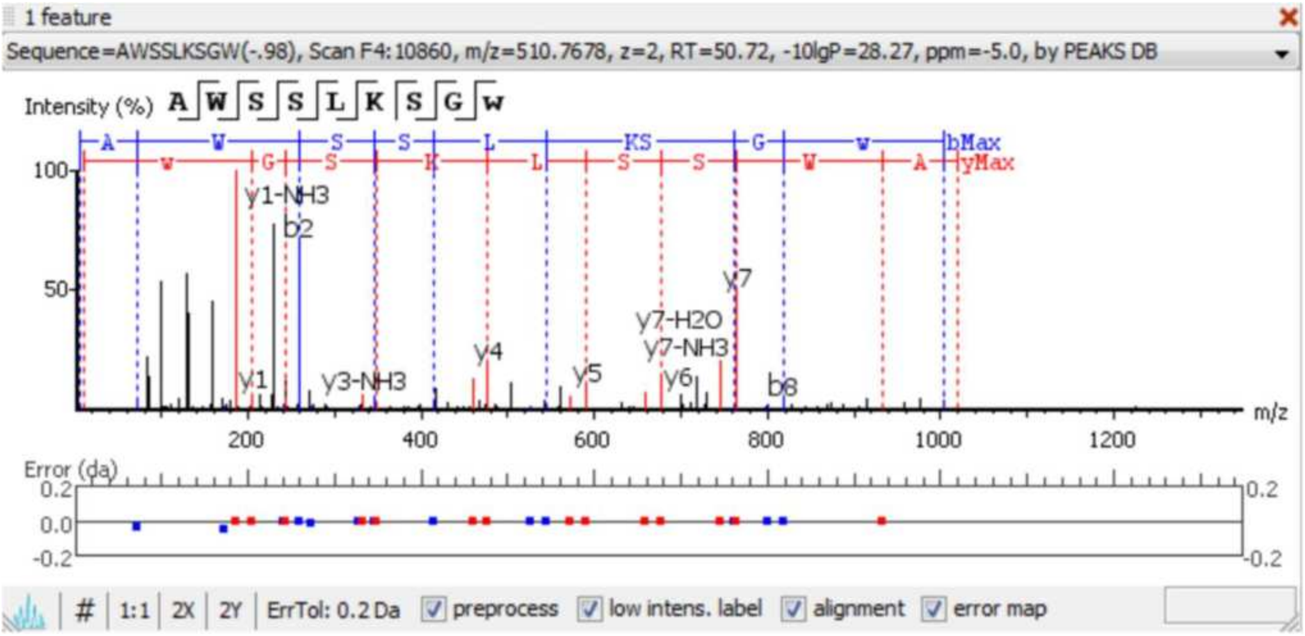

#	b	b-H2O	b-NH3	b (2+)	Seq	y	y-H2O	y-NH3	y (2+)	#
1	72.08	54.03	55.02	36.52	A					9
2	258.12	240.11	241.10	129.56	W	949.49	931.48	932.47	475.24	8
3	345.16	327.15	328.13	173.13	S	763.41	745.40	746.38	382.20	7
4	432.19	414.18	415.16	216.59	S	676.38	658.37	659.35	338.69	6
5	545.28	527.26	528.25	273.16	L	589.35	571.34	572.32	295.17	5
6	673.37	655.36	656.34	337.18	K	476.26	458.25	459.23	238.63	4
7	760.39	742.39	743.37	380.70	S	348.17	330.16	331.14	174.58	3
8	817.42	799.41	800.39	409.21	G	261.13	243.12	244.11	131.07	2
9					W(-.98)	204.11	186.10	187.09	102.56	1

Allatostatine-B/Myoinhibitory peptide-7, (MIP-7)

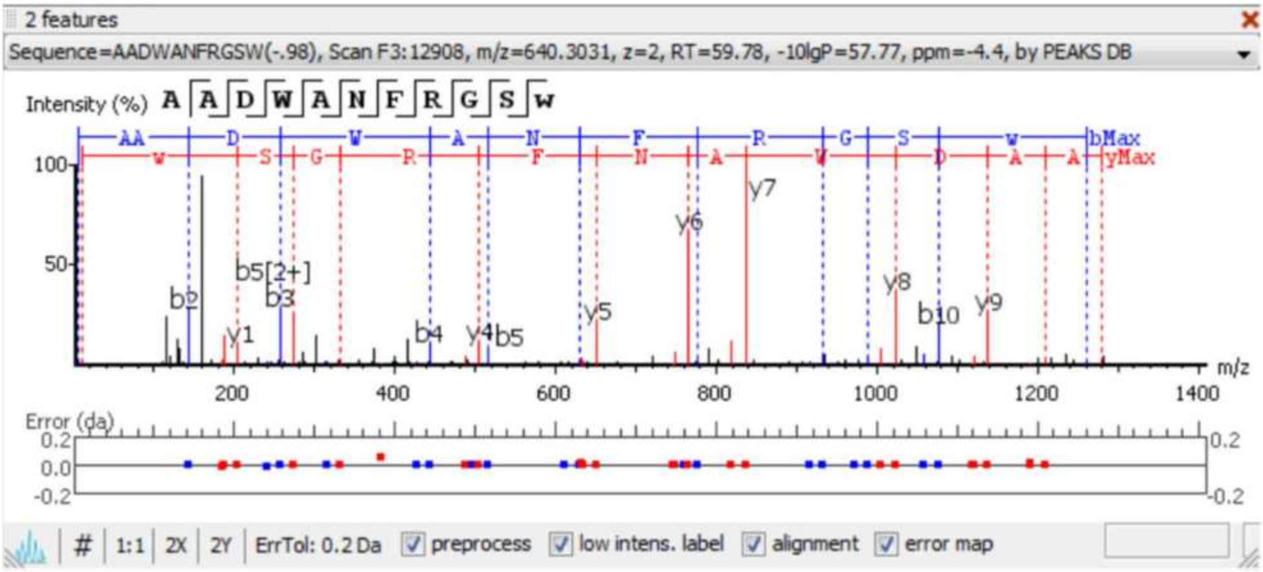

#	b	b-H2O	b-NH3	b (2+)	Seq	y	y-H2O	y-NH3	y (2+)	#
1	72.04	54.03	55.02	36.52	A					11
2	143.08	125.07	126.06	72.04	A	1208.56	1190.55	1191.54	604.78	10
3	258.11	240.10	241.10	129.55	D	1137.52	1119.51	1120.50	569.26	9
4	444.19	426.18	427.16	222.59	W	1022.50	1004.49	1005.46	511.75	8
5	515.23	497.21	498.20	258.11	A	836.42	818.41	819.39	418.71	7
6	629.27	611.26	612.24	315.13	N	765.38	747.37	748.35	383.14	6
7	776.34	758.33	759.31	388.67	F	651.34	633.31	634.31	326.17	5
8	932.44	914.43	915.41	466.72	R	504.27	486.26	487.24	252.63	4
9	989.46	971.44	972.43	495.23	G	348.17	330.16	331.14	174.58	3
10	1076.49	1058.48	1059.47	538.75	S	291.15	273.13	274.12	146.07	2
11					W(-.98)	204.11	186.12	187.09	102.56	1

Allatostatine-B/Myoinhibitory peptide-8, (MIP-8)

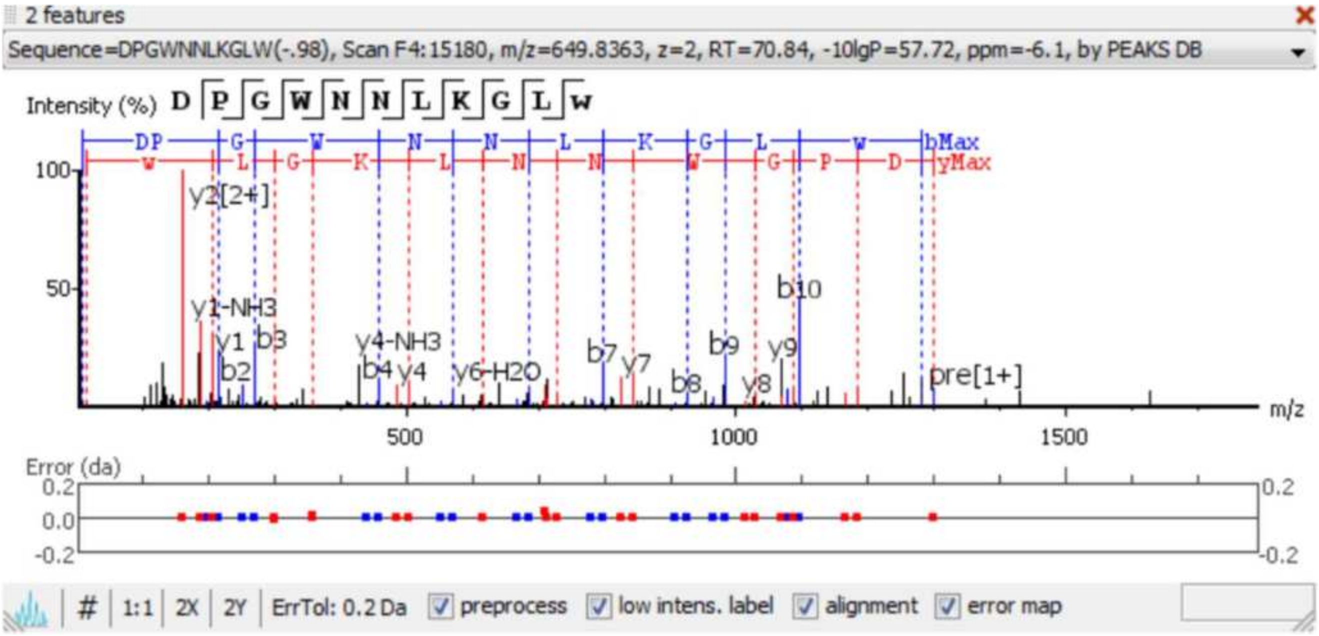

#	b	b-H2O	b-NH3	b (2+)	Seq	y	y-H2O	y-NH3	y (2+)	#
1	116.03	98.02	99.01	58.52	D					11
2	213.09	195.08	196.06	107.04	P	1183.64	1165.63	1166.61	592.32	10
3	270.11	252.10	253.08	135.55	G	1086.58	1068.57	1069.56	543.79	9
4	456.19	438.18	439.16	228.59	W	1029.57	1011.55	1012.54	515.28	8
5	570.23	552.22	553.20	285.62	N	843.48	825.47	826.46	422.24	7
6	684.27	666.26	667.25	342.64	N	729.44	711.39	712.40	365.22	6
7	797.36	779.35	780.33	399.18	L	615.40	597.39	598.37	308.20	5
8	925.46	907.44	908.43	463.23	K	502.31	484.30	485.29	251.66	4
9	982.47	964.46	965.45	491.74	G	374.22	356.19	357.19	187.61	3
10	1095.56	1077.55	1078.53	548.28	L	317.20	299.21	300.17	159.09	2
11					W(-.98)	204.11	186.10	187.09	102.56	1

Allatostatine-B/Myoinhibitory peptide-9, (MIP-9)

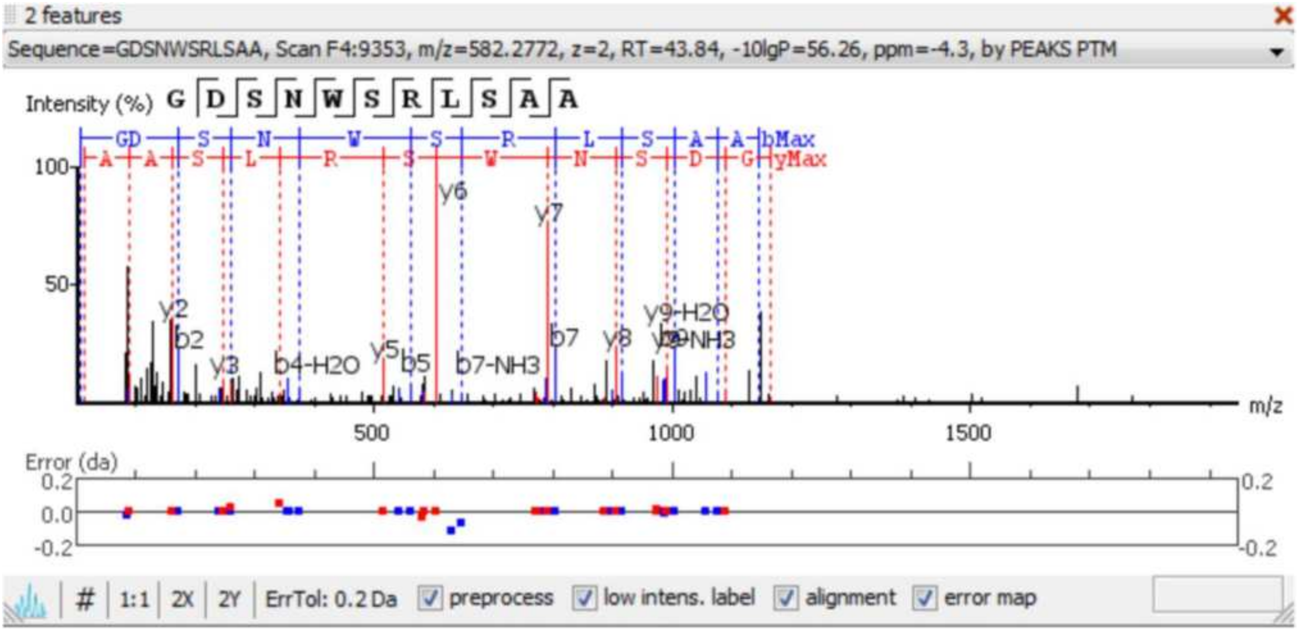

#	b	b-H2O	b-NH3	b (2+)	Seq	y	y-H2O	y-NH3	y (2+)	#
1	58.03	40.02	41.00	29.51	G					11
2	173.06	155.05	156.03	87.06	D	1106.52	1088.51	1089.50	553.76	10
3	260.09	242.08	243.06	130.54	S	991.50	973.47	974.47	496.25	9
4	374.13	356.12	357.10	187.57	N	904.46	886.45	887.44	452.73	8
5	560.21	542.20	543.18	280.61	W	790.42	772.41	773.39	395.71	7
6	647.32	629.23	630.34	324.12	S	604.34	586.33	587.31	302.67	6
7	803.34	785.33	786.32	402.17	R	517.31	499.30	500.28	259.12	5
8	916.43	898.41	899.40	458.71	L	361.21	343.14	344.18	181.10	4
9	1003.46	985.45	986.44	502.23	S	248.12	230.11	231.10	124.56	3
10	1074.50	1056.48	1057.47	537.75	A	161.09	143.08	144.07	81.05	2
11					A	90.06	72.04	73.03	45.53	1

Allatostatine-B/Myoinhibitory peptide-PP-3, (MIP-PP-3)

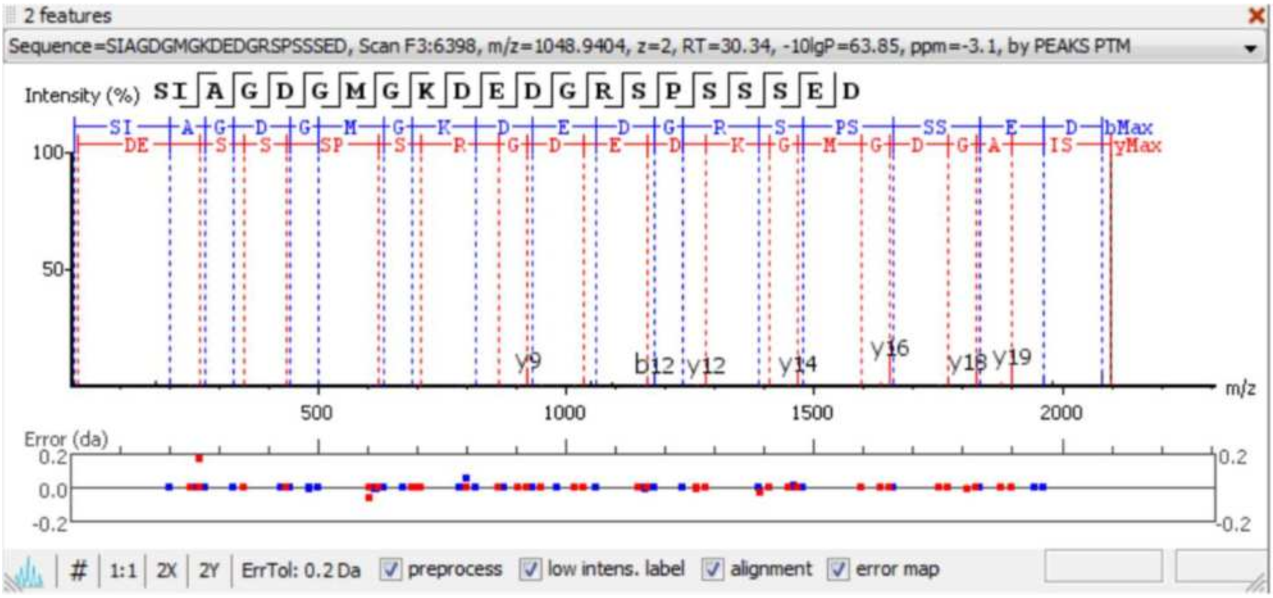

#	b	b-H2O	b-NH3	b (2+)	Seq	y	y-H2O	y-NH3	y (2+)	#
1	88.04	70.03	71.01	44.52	S					21
2	201.12	183.11	184.10	101.06	I	2009.84	1991.82	1992.81	1005.43	20
3	272.16	254.11	255.13	136.58	A	1896.75	1878.74	1879.72	948.88	19
4	329.15	311.17	312.16	165.09	G	1825.72	1807.70	1808.69	913.36	18
5	444.21	426.20	427.18	222.60	D	1768.70	1750.68	1751.67	884.85	17
6	501.23	483.22	484.20	251.12	G	1653.69	1635.68	1636.68	827.33	16
7	632.27	614.26	615.24	316.64	M	1596.66	1578.63	1579.62	798.82	15
8	689.29	671.28	672.27	345.10	G	1465.60	1447.60	1448.59	733.30	14
9	817.39	799.38	800.36	409.19	K	1408.58	1390.58	1391.59	704.79	13
10	932.41	914.40	915.39	466.71	D	1280.49	1262.48	1263.46	640.74	12
11	1061.46	1043.45	1044.43	531.23	E	1165.46	1147.45	1148.43	583.23	11
12	1176.48	1158.47	1159.46	588.74	D	1036.42	1018.41	1019.39	518.71	10
13	1233.51	1215.50	1216.48	617.27	G	921.39	903.38	904.37	461.20	9
14	1389.61	1371.60	1372.58	695.31	R	864.37	846.36	847.34	432.68	8
15	1476.63	1458.62	1459.63	738.82	S	708.27	690.26	691.24	354.63	7
16	1573.69	1555.68	1556.66	787.35	P	621.24	603.22	604.21	311.12	6
17	1660.72	1642.71	1643.70	830.86	S	524.18	506.21	507.16	262.59	5
18	1747.74	1729.74	1730.73	874.38	S	437.15	419.14	420.12	219.08	4
19	1834.79	1816.78	1817.76	917.89	S	350.12	332.11	333.09	175.56	3
20	1963.83	1945.82	1946.80	982.42	E	263.09	245.08	246.06	132.04	2
21					D	134.04	116.03	117.02	67.52	1

Allatostatine-CCC (Ast-CCC)

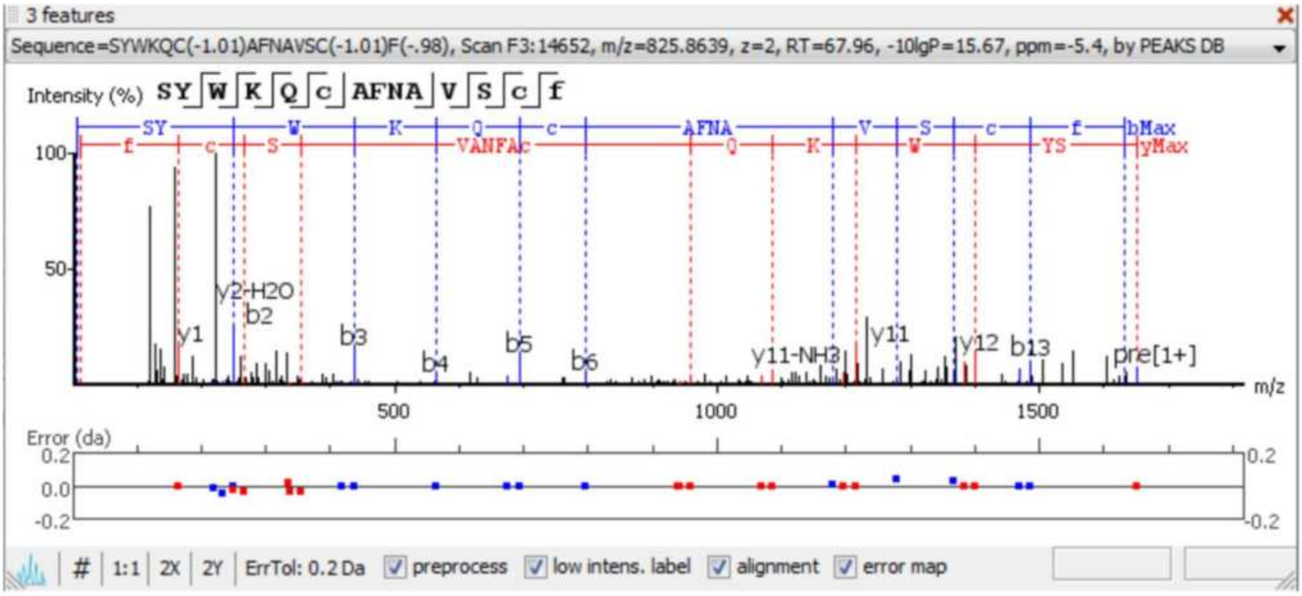

#	b	b-H2O	b-NH3	b (2+)	Seq	y	y-H2O	y-NH3	y (2+)	#
1	88.04	70.03	71.01	44.52	S					14
2	251.10	233.09	234.12	126.05	Y	1563.69	1545.68	1546.66	782.34	13
3	437.18	419.17	420.16	219.11	W	1400.62	1382.61	1383.60	700.81	12
4	565.28	547.27	548.25	283.14	K	1214.55	1196.53	1197.52	607.77	11
5	693.34	675.33	676.31	347.17	Q	1086.45	1068.44	1069.43	543.72	10
6	795.33	777.33	778.31	398.17	C(-1.01)	958.39	940.38	941.37	479.70	9
7	866.37	848.36	849.35	433.69	A	856.39	838.38	839.36	428.69	8
8	1013.44	995.43	996.42	507.22	F	785.35	767.34	768.33	393.18	7
9	1127.49	1109.48	1110.46	564.24	N	638.28	620.27	621.26	319.64	6
10	1198.52	1180.49	1181.50	599.76	A	524.24	506.23	507.21	262.62	5
11	1297.59	1279.54	1280.56	649.30	V	453.20	435.19	436.18	227.10	4
12	1384.62	1366.57	1367.60	692.81	S	354.18	336.10	337.15	177.57	3
13	1486.63	1468.62	1469.61	743.81	C(-1.01)	267.15	249.12	250.08	134.05	2
14					F(-.98)	165.10	147.09	148.08	83.05	1

Calcitonin-like diuretic hormone (Cal)

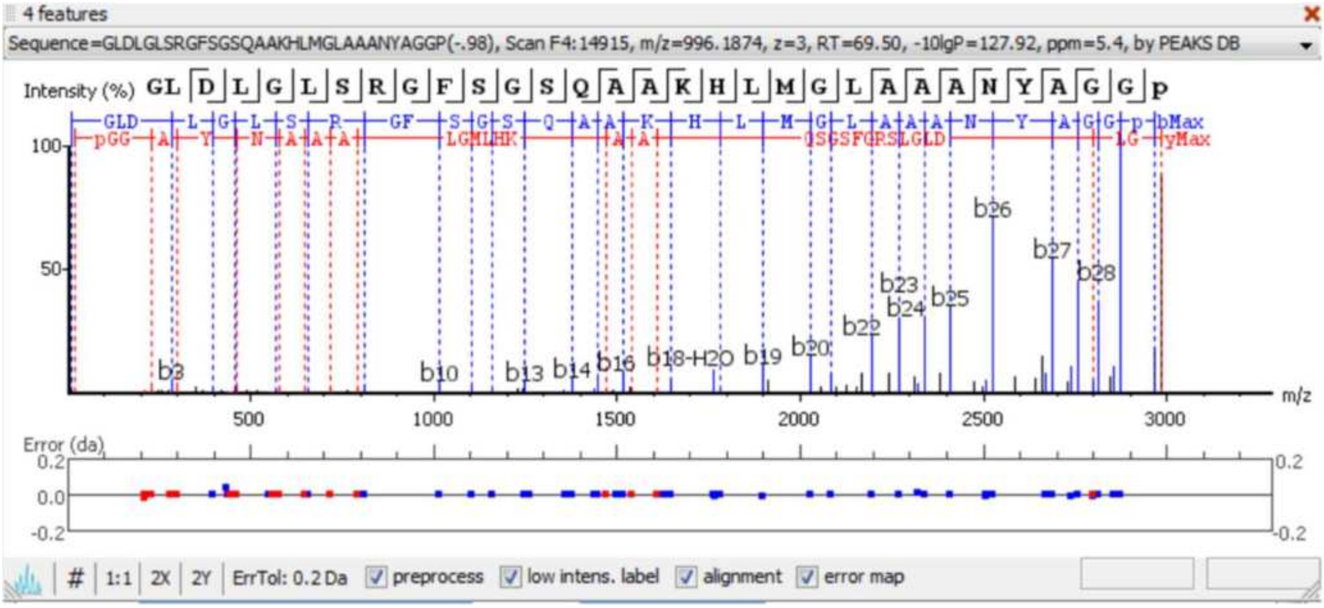

#	b	b-H2O	b-NH3	b (2+)	Seq	y	y-H2O	y-NH3	y (2+)	#
1	58.03	40.02	41.00	29.51	G					31
2	171.11	153.10	154.09	86.06	L	2929.51	2911.50	2912.48	1465.26	30
3	286.14	268.13	269.11	143.57	D	2816.43	2798.42	2799.40	1408.71	29
4	399.22	381.21	382.20	200.11	L	2701.40	2683.39	2684.37	1351.20	28
5	456.25	438.23	439.21	228.62	G	2588.31	2570.30	2571.29	1294.66	27
6	569.33	551.32	552.30	285.16	L	2531.29	2513.28	2514.27	1266.15	26
7	656.36	638.35	639.33	328.68	S	2418.21	2400.20	2401.18	1209.60	25
8	812.47	794.45	795.44	406.73	R	2331.18	2313.17	2314.15	1166.09	24
9	869.48	851.47	852.46	435.20	G	2175.08	2157.07	2158.05	1088.04	23
10	1016.56	998.54	999.53	508.78	F	2118.05	2100.04	2101.03	1059.53	22
11	1103.58	1085.57	1086.56	552.30	S	1970.99	1952.98	1953.96	985.99	21
12	1160.60	1142.60	1143.58	580.80	G	1883.95	1865.94	1866.93	942.48	20
13	1247.64	1229.63	1230.61	624.32	S	1826.93	1808.92	1809.91	913.97	19
14	1375.70	1357.69	1358.67	688.35	Q	1739.90	1721.89	1722.87	870.45	18
15	1446.74	1428.72	1429.71	723.87	A	1611.84	1593.83	1594.82	806.42	17
16	1517.77	1499.76	1500.74	759.39	A	1540.80	1522.79	1523.78	770.90	16
17	1645.87	1627.85	1628.84	823.43	K	1469.77	1451.76	1452.74	735.38	15
18	1782.93	1764.92	1765.91	891.96	H	1341.67	1323.66	1324.65	671.34	14
19	1896.02	1878.00	1878.98	948.50	L	1204.61	1186.60	1187.59	602.81	13
20	2027.05	2009.04	2010.02	1014.02	M	1091.53	1073.52	1074.50	546.27	12
21	2084.07	2066.06	2067.04	1042.54	G	960.49	942.48	943.46	480.74	11
22	2197.16	2179.14	2180.13	1099.08	L	903.47	885.46	886.44	452.23	10
23	2268.19	2250.18	2251.17	1134.60	A	790.39	772.37	773.36	395.69	9
24	2339.23	2321.21	2322.20	1170.11	A	719.35	701.34	702.32	360.17	8
25	2410.27	2392.26	2393.24	1205.63	A	648.31	630.30	631.28	324.65	7
26	2524.31	2506.29	2507.30	1262.66	N	577.27	559.26	560.25	289.14	6
27	2687.38	2669.36	2670.35	1344.19	Y	463.23	445.22	446.20	232.11	5
28	2758.41	2740.40	2741.39	1379.70	A	300.17	282.16	283.14	150.58	4
29	2815.43	2797.42	2798.42	1408.22	G	229.13	211.14	212.10	115.06	3
30	2872.45	2854.44	2855.44	1436.73	G	172.11	154.10	155.08	86.55	2
31					P(-.98)	115.09	97.08	98.06	58.04	1

Periviscerokinin (PVK) / CAPA-peptides; PVK-CAPA-2

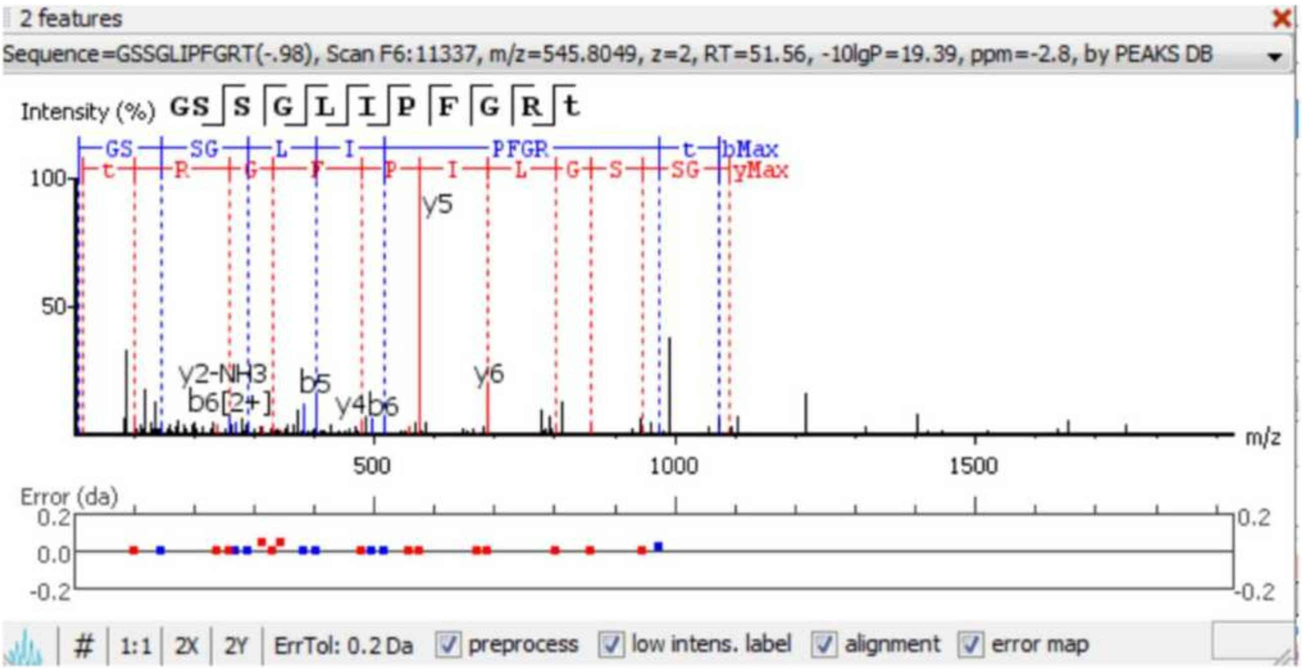

#	b	b-H2O	b-NH3	b (2+)	Seq	y	y-H2O	y-NH3	y (2+)	#
1	58.03	40.02	41.00	29.51	G					11
2	145.06	127.05	128.03	73.03	S	1033.58	1015.57	1016.55	517.29	10
3	232.09	214.08	215.07	116.55	S	946.55	928.54	929.52	473.77	9
4	289.11	271.10	272.09	145.06	G	859.52	841.50	842.49	430.26	8
5	402.20	384.19	385.17	201.60	L	802.49	784.48	785.47	401.75	7
6	515.28	497.27	498.26	258.15	I	689.41	671.40	672.39	345.16	6
7	612.34	594.33	595.31	306.67	P	576.32	558.31	559.30	288.66	5
8	759.40	741.39	742.38	380.20	F	479.26	461.26	462.25	240.13	4
9	816.43	798.41	799.40	408.71	G	332.20	314.19	315.13	166.60	3
10	972.50	954.52	955.50	486.76	R	275.18	257.17	258.15	138.09	2
11					T(-.98)	119.08	101.07	102.06	60.04	1

CNMamide

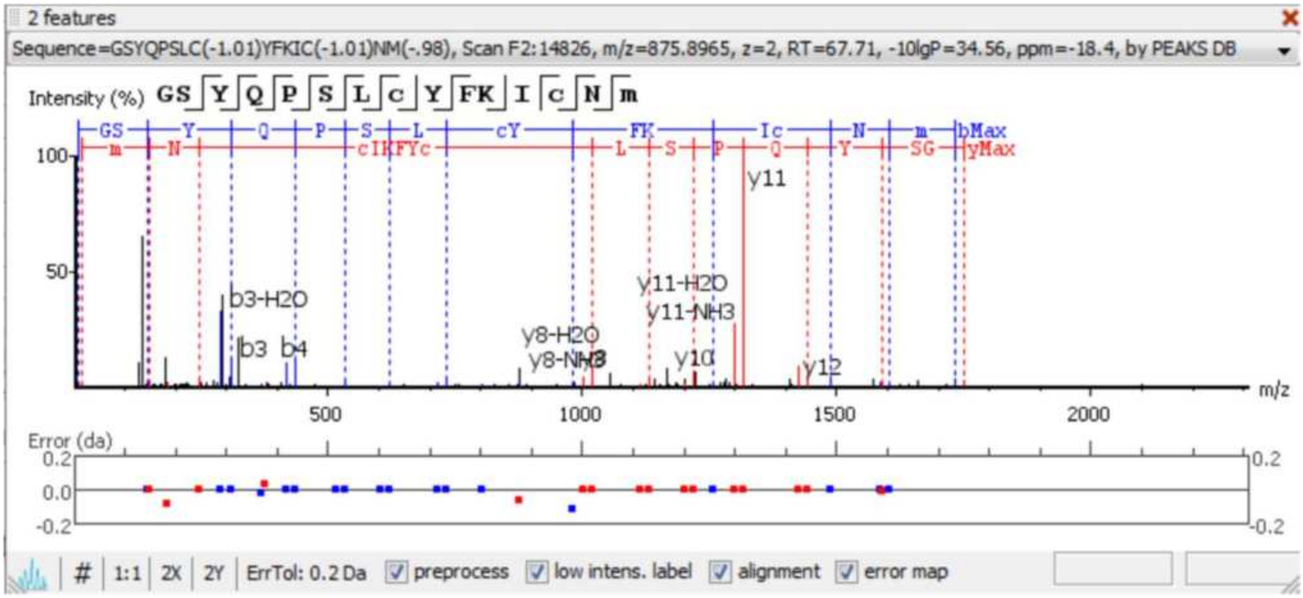

#	b	b-H2O	b-NH3	b (2+)	Seq	y	y-H2O	y-NH3	y (2+)	#
1	58.03	40.02	41.00	29.51	G					15
2	145.06	127.05	128.03	73.03	S	1693.75	1675.74	1676.73	847.38	14
3	308.12	290.11	291.10	154.56	Y	1606.72	1588.71	1589.71	803.86	13
4	436.18	418.17	419.16	218.59	Q	1443.66	1425.64	1426.64	722.33	12
5	533.24	515.22	516.21	267.12	P	1315.60	1297.59	1298.58	658.30	11
6	620.27	602.26	603.24	310.63	S	1218.55	1200.54	1201.52	609.77	10
7	733.35	715.34	716.33	367.20	L	1131.52	1113.50	1114.49	566.26	9
8	835.35	817.34	818.33	418.17	C(-1.01)	1018.43	1000.43	1001.41	509.72	8
9	998.42	980.41	981.52	499.71	Y	916.43	898.42	899.40	458.71	7
10	1145.49	1127.47	1128.46	573.24	F	753.37	735.36	736.34	377.15	6
11	1273.58	1255.56	1256.55	637.29	K	606.30	588.29	589.27	303.65	5
12	1386.66	1368.65	1369.64	693.83	I	478.20	460.19	461.18	239.60	4
13	1488.66	1470.66	1471.64	744.83	C(-1.01)	365.12	347.11	348.09	183.15	3
14	1602.71	1584.70	1585.69	801.86	N	263.12	245.11	246.09	132.06	2
15					M(-.98)	149.07	131.06	132.05	75.04	1

Corazonin (Crz)

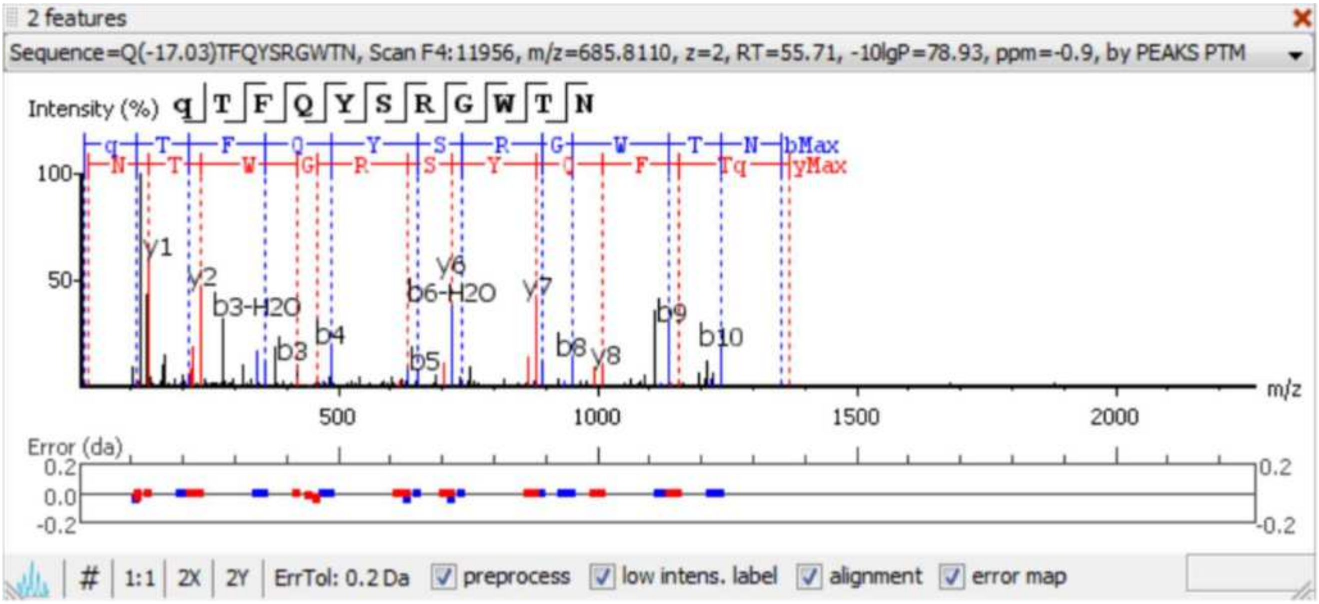

#	b	b-H2O	b-NH3	b (2+)	Seq	y	y-H2O	y-NH3	y (2+)	#
1	112.09	94.03	95.01	56.52	Q(-17.03)					11
2	213.09	195.08	196.06	107.04	T	1259.58	1241.57	1242.55	630.29	10
3	360.16	342.14	343.13	180.58	F	1158.53	1140.52	1141.51	579.77	9
4	488.21	470.20	471.19	244.61	Q	1011.47	993.45	994.44	506.23	8
5	651.28	633.31	634.25	326.14	Y	883.41	865.40	866.38	442.22	7
6	738.31	720.34	721.28	369.65	S	720.34	702.33	703.32	360.67	6
7	894.41	876.40	877.38	447.71	R	633.31	615.30	616.28	317.16	5
8	951.43	933.42	934.41	476.22	G	477.21	459.25	460.22	239.10	4
9	1137.51	1119.50	1120.49	569.26	W	420.19	402.18	403.16	210.59	3
10	1238.56	1220.54	1221.55	619.78	T	234.11	216.10	217.08	117.55	2
11					N	133.06	115.09	116.03	67.03	1

Corazonin (Crz)

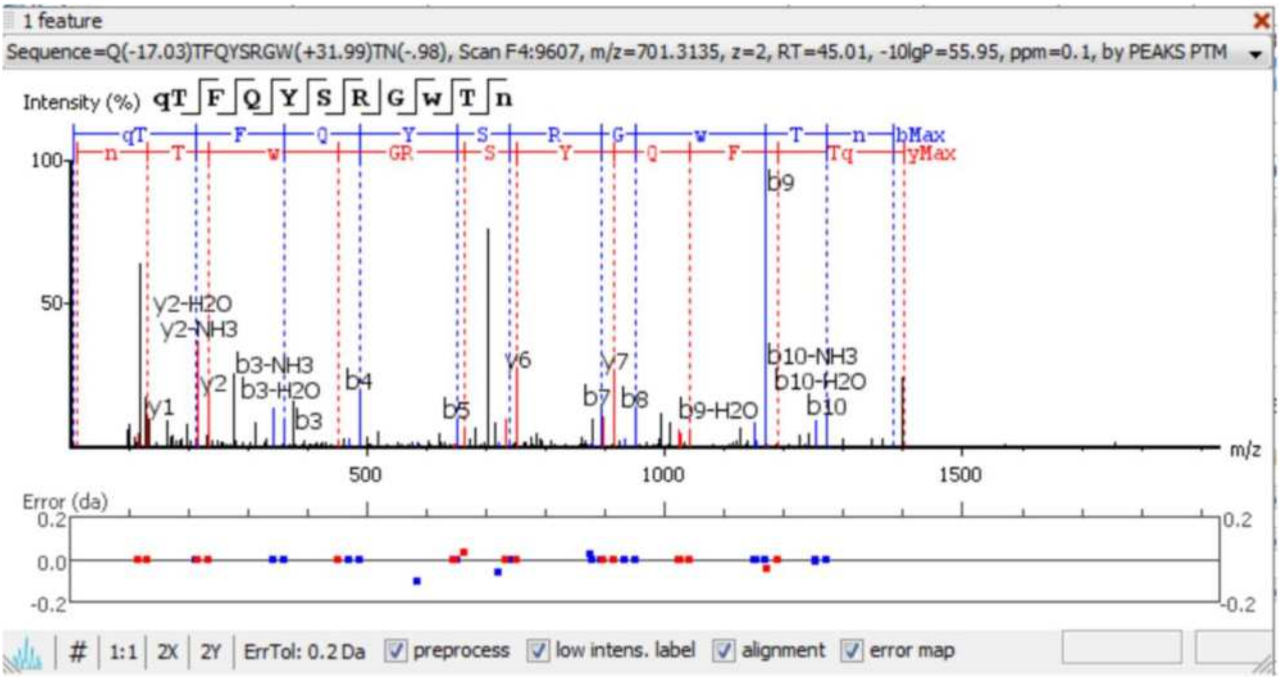

#	b	b-H2O	b-NH3	b (2+)	Seq	y	y-H2O	y-NH3	y (2+)	#
1	112.04	94.03	95.01	56.52	Q(-17.03)					11
2	213.09	195.08	196.06	107.04	T	1290.59	1272.58	1273.56	645.79	10
3	360.16	342.14	343.14	180.58	F	1189.54	1171.53	1172.56	595.27	9
4	488.21	470.20	471.19	244.61	Q	1042.47	1024.46	1025.45	521.74	8
5	651.28	633.27	634.25	326.14	Y	914.41	896.40	897.39	457.71	7
6	738.31	720.30	721.34	369.65	S	751.35	733.34	734.32	376.17	6
7	894.41	876.37	877.39	447.71	R	664.27	646.31	647.29	332.66	5
8	951.43	933.42	934.41	476.22	G	508.22	490.20	491.19	254.61	4
9	1169.50	1151.49	1152.48	585.36	W(+31.99)	451.20	433.18	434.17	226.10	3
10	1270.55	1252.54	1253.54	635.77	T	233.12	215.11	216.10	117.06	2
11					N(-.98)	132.08	114.07	115.05	66.54	1

Corazonin (Crz)-PP-1

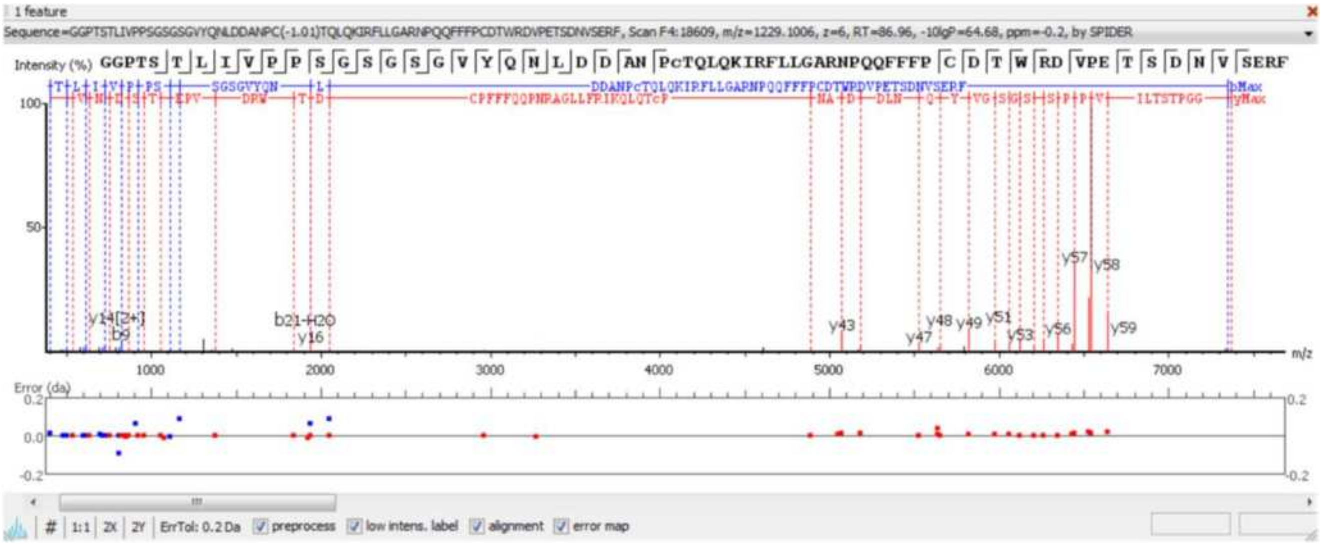

#	b	b-H2O	b-NH3	b (2+)	Seq	y	y-H2O	y-NH3	y (2+)	#
1	58.03	40.02	41.00	29.51	G					67
2	115.05	97.04	98.02	58.03	G	7312.54	7294.53	7295.51	3656.77	66
3	212.10	194.09	195.08	106.55	P	7255.52	7237.51	7238.49	3628.26	65
4	313.15	295.14	296.12	157.08	T	7158.47	7140.46	7141.44	3579.73	64
5	400.17	382.17	383.16	200.59	S	7057.42	7039.41	7040.39	3529.21	63
6	501.23	483.22	484.20	251.12	T	6970.39	6952.38	6953.36	3485.69	62
7	614.32	596.31	597.29	307.66	L	6869.34	6851.33	6852.31	3435.17	61
8	727.40	709.39	710.37	364.20	I	6756.25	6738.24	6739.23	3378.63	60
9	826.47	808.46	809.54	413.73	V	6643.15	6625.16	6626.14	3322.09	59
10	923.52	905.51	906.43	462.26	P	6544.09	6526.07	6527.08	3272.56	58
11	1020.57	1002.56	1003.55	510.79	P	6447.03	6429.03	6430.02	3224.02	57
12	1107.61	1089.59	1090.58	554.30	S	6349.99	6331.99	6332.97	3175.50	56
13	1164.54	1146.62	1147.60	582.81	G	6262.97	6244.95	6245.94	3131.98	55
14	1251.66	1233.65	1234.63	626.33	S	6205.94	6187.93	6188.92	3103.47	54
15	1308.68	1290.67	1291.65	654.84	G	6118.91	6100.90	6101.88	3059.96	53
16	1395.71	1377.70	1378.68	698.35	S	6061.88	6043.88	6044.86	3031.45	52
17	1452.73	1434.72	1435.71	726.87	G	5974.85	5956.85	5957.83	2987.93	51
18	1551.80	1533.79	1534.77	776.40	V	5917.84	5899.83	5900.81	2959.42	50
19	1714.87	1696.85	1697.84	857.93	Y	5818.76	5800.76	5801.74	2909.88	49
20	1842.92	1824.91	1825.90	921.96	Q	5655.70	5637.66	5638.67	2828.35	48
21	1956.97	1938.89	1939.94	978.98	N	5527.64	5509.64	5510.62	2764.32	47
22	2070.05	2052.04	2052.93	1035.53	L	5413.60	5395.59	5396.58	2707.30	46
23	2185.08	2167.07	2168.05	1093.04	D	5300.52	5282.51	5283.49	2650.76	45
24	2300.10	2282.09	2283.08	1150.55	D	5185.48	5167.48	5168.46	2593.25	44
25	2371.14	2353.13	2354.11	1186.07	A	5070.45	5052.45	5053.44	2535.73	43
26	2485.18	2467.17	2468.16	1243.09	N	4999.43	4981.42	4982.40	2500.21	42
27	2582.24	2564.23	2565.21	1291.62	P	4885.38	4867.37	4868.36	2443.19	41
28	2684.24	2666.23	2667.21	1342.62	C(-1.01)	4788.33	4770.32	4771.31	2394.67	40
29	2785.29	2767.28	2768.26	1393.14	T	4686.33	4668.32	4669.30	2343.67	39
30	2913.34	2895.33	2896.32	1457.17	Q	4585.28	4567.27	4568.26	2293.14	38
31	3026.43	3008.42	3009.40	1513.71	L	4457.23	4439.21	4440.20	2229.11	37
32	3154.49	3136.48	3137.46	1577.74	Q	4344.14	4326.13	4327.11	2172.57	36
33	3282.58	3264.57	3265.56	1641.79	K	4216.08	4198.07	4199.05	2108.54	35
34	3395.67	3377.66	3378.64	1698.33	I	4087.99	4069.98	4070.96	2044.49	34
35	3551.77	3533.76	3534.74	1776.38	R	3974.90	3956.89	3957.88	1987.95	33
36	3698.84	3680.83	3681.81	1849.92	F	3818.80	3800.79	3801.77	1909.90	32
37	3811.92	3793.91	3794.89	1906.46	L	3671.73	3653.72	3654.71	1836.37	31
38	3925.00	3906.99	3907.98	1963.00	L	3558.65	3540.64	3541.62	1779.82	30
39	3982.03	3964.02	3965.00	1991.51	G	3445.57	3427.55	3428.54	1723.28	29
40	4053.06	4035.05	4036.04	2027.03	A	3388.54	3370.53	3371.52	1694.77	28
41	4209.16	4191.15	4192.14	2105.08	R	3317.51	3299.50	3300.48	1659.25	27
42	4323.21	4305.20	4306.18	2162.10	N	3161.41	3143.40	3144.38	1581.20	26
43	4420.26	4402.25	4403.23	2210.63	P	3047.36	3029.35	3030.34	1524.18	25
44	4548.32	4530.31	4531.29	2274.66	Q	2950.31	2932.30	2933.28	1475.66	24
45	4676.38	4658.37	4659.35	2338.69	Q	2822.25	2804.24	2805.22	1411.63	23
46	4823.45	4805.43	4806.42	2412.22	F	2694.19	2676.18	2677.17	1347.60	22
47	4970.51	4952.50	4953.49	2485.76	F	2547.12	2529.11	2530.10	1274.06	21
48	5117.58	5099.57	5100.56	2559.29	F	2400.06	2382.05	2383.03	1200.53	20
49	5214.63	5196.62	5197.61	2607.82	P	2252.99	2234.98	2235.96	1126.99	19
50	5317.64	5299.63	5300.62	2659.32	C	2155.93	2137.92	2138.91	1078.49	18
51	5432.67	5414.66	5415.64	2716.84	D	2052.93	2034.92	2035.90	1026.96	17
52	5533.72	5515.71	5516.69	2767.36	T	1937.90	1919.89	1920.89	969.45	16
53	5719.80	5701.79	5702.77	2860.40	W	1836.85	1818.84	1819.82	918.93	15
54	5875.90	5857.89	5858.87	2938.45	R	1650.77	1632.76	1633.74	825.89	14
55	5990.93	5972.92	5973.90	2995.96	D	1494.67	1476.66	1477.64	747.84	13
56	6089.99	6071.98	6072.97	3045.50	V	1379.65	1361.63	1362.62	690.32	12
57	6187.05	6169.04	6170.02	3094.02	P	1280.58	1262.56	1263.55	640.79	11
58	6316.09	6298.08	6299.06	3158.54	E	1183.52	1165.51	1166.50	592.26	10
59	6417.14	6399.13	6400.11	3209.07	T	1054.48	1036.47	1037.45	527.74	9
60	6504.17	6486.16	6487.14	3252.58	S	953.44	935.42	936.41	477.22	8
61	6619.20	6601.19	6602.17	3310.10	D	866.41	848.39	849.38	433.70	7
62	6733.24	6715.23	6716.21	3367.12	N	751.38	733.36	734.35	376.19	6
63	6832.31	6814.30	6815.28	3416.65	V	637.33	619.32	620.30	319.17	5
64	6919.34	6901.33	6902.31	3460.17	S	538.26	520.25	521.23	269.63	4
65	7048.38	7030.37	7031.36	3524.69	E	451.23	433.22	434.20	226.11	3
66	7204.48	7186.47	7187.46	3602.74	R	322.19	304.18	305.16	161.59	2
67					F	166.09	148.08	149.06	83.54	1

Corazonin (Crz)-PP-2

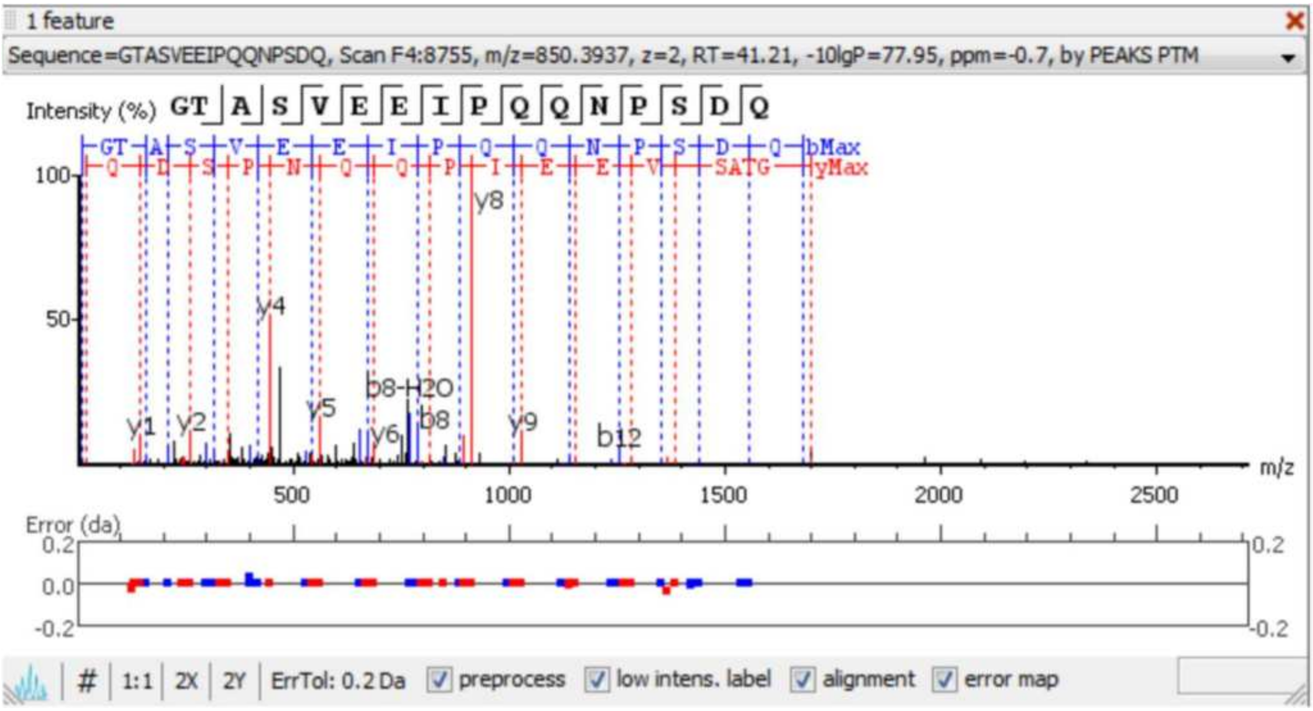

#	b	b-H2O	b-NH3	b (2+)	Seq	y	y-H2O	y-NH3	y (2+)	#
1	58.03	40.02	41.00	29.51	G					16
2	159.08	141.07	142.05	80.04	T	1642.76	1624.74	1625.73	821.88	15
3	230.11	212.10	213.09	115.56	A	1541.71	1523.70	1524.68	771.35	14
4	317.15	299.14	300.12	159.08	S	1470.67	1452.66	1453.64	735.84	13
5	416.21	398.20	399.16	208.61	V	1383.64	1365.63	1366.65	692.32	12
6	545.26	527.25	528.23	273.13	E	1284.57	1266.56	1267.55	642.79	11
7	674.30	656.29	657.27	337.65	E	1155.53	1137.51	1138.52	578.26	10
8	787.38	769.37	770.36	394.19	I	1026.49	1008.48	1009.46	513.74	9
9	884.44	866.43	867.41	442.72	P	913.40	895.39	896.38	457.20	8
10	1012.49	994.49	995.47	506.75	Q	816.35	798.34	799.33	408.67	7
11	1140.56	1122.55	1123.53	570.78	Q	688.29	670.29	671.27	344.64	6
12	1254.60	1236.59	1237.58	627.80	N	560.23	542.22	543.21	280.62	5
13	1351.66	1333.64	1334.62	676.32	P	446.19	428.18	429.16	223.59	4
14	1438.69	1420.66	1421.67	719.84	S	349.14	331.12	332.11	175.07	3
15	1553.71	1535.70	1536.69	777.35	D	262.10	244.09	245.08	131.55	2
16					Q	147.08	129.10	130.05	74.04	1

Crustacean cardioactive peptide-PP (CCAP-PP) [Q]

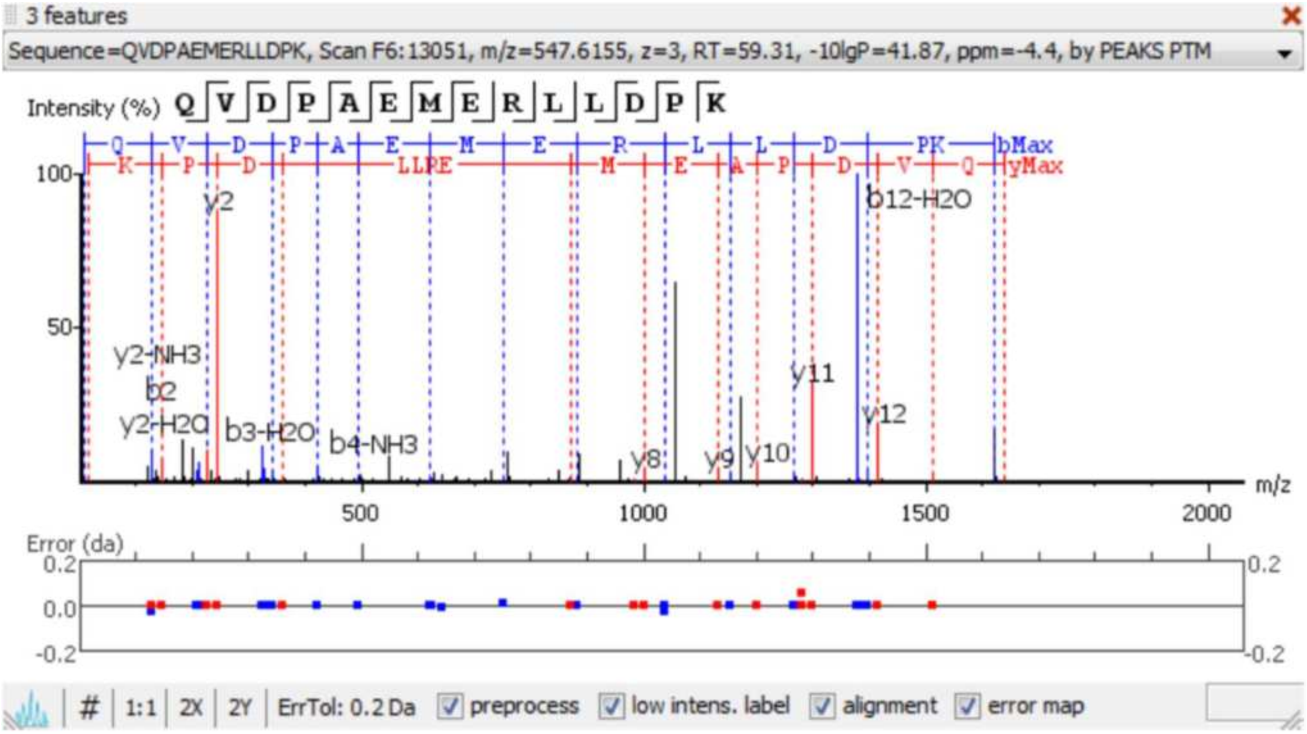

#	b	b-H2O	b-NH3	b (2+)	Seq	y	y-H2O	y-NH3	y (2+)	#
1	129.10	111.06	112.04	65.03	Q					14
2	228.13	210.12	211.11	114.57	V	1512.77	1494.76	1495.75	756.89	13
3	343.16	325.15	326.13	172.08	D	1413.70	1395.69	1396.68	707.35	12
4	440.21	422.20	423.19	220.61	P	1298.68	1280.60	1281.65	649.84	11
5	511.25	493.24	494.22	256.13	A	1201.63	1183.61	1184.60	601.31	10
6	640.29	622.29	623.27	320.65	E	1130.59	1112.58	1113.56	565.79	9
7	771.33	753.31	754.31	386.17	M	1001.54	983.54	984.52	501.27	8
8	900.38	882.37	883.35	450.69	E	870.50	852.49	853.48	435.75	7
9	1056.48	1038.50	1039.45	528.74	R	741.46	723.45	724.43	371.23	6
10	1169.56	1151.55	1152.54	585.28	L	585.36	567.35	568.33	293.18	5
11	1282.65	1264.64	1265.62	641.83	L	472.28	454.27	455.25	236.64	4
12	1397.67	1379.66	1380.65	699.34	D	359.19	341.18	342.17	180.10	3
13	1494.73	1476.72	1477.70	747.86	P	244.17	226.15	227.14	122.58	2
14					K	147.11	129.10	130.09	74.06	1

Crustacean cardioactive peptide-PP (CCAP-PP)_[pQ]

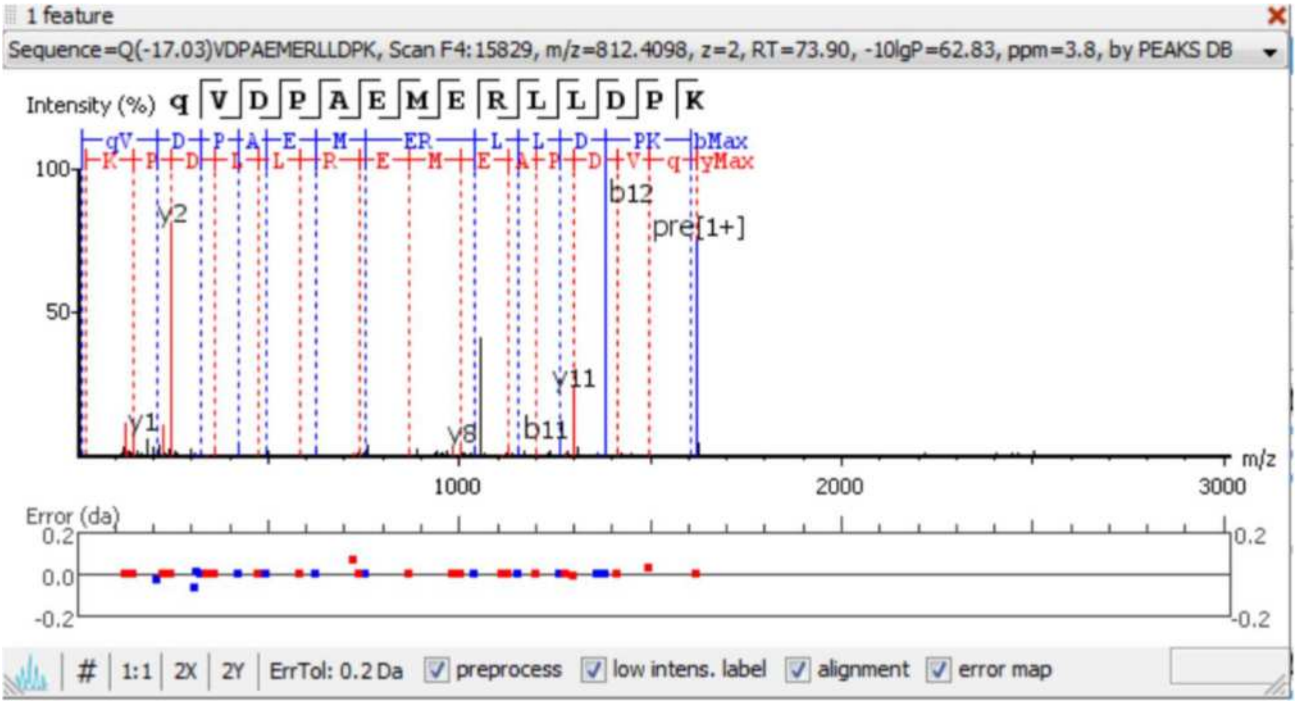

#	b	b-H2O	b-NH3	b (2+)	Seq	y	y-H2O	y-NH3	y (2+)	#
1	112.04	94.03	95.01	56.52	Q(-17.03)					14
2	211.14	193.10	194.08	106.05	V	1512.77	1494.76	1495.71	756.89	13
3	326.13	308.20	309.11	163.57	D	1413.70	1395.69	1396.68	707.35	12
4	423.19	405.18	406.16	212.09	P	1298.69	1280.66	1281.65	649.84	11
5	494.23	476.21	477.20	247.61	A	1201.62	1183.61	1184.60	601.31	10
6	623.27	605.26	606.24	312.12	E	1130.59	1112.57	1113.56	565.79	9
7	754.31	736.30	737.28	377.65	M	1001.55	983.54	984.52	501.27	8
8	883.35	865.34	866.32	442.18	E	870.50	852.49	853.48	435.75	7
9	1039.44	1021.44	1022.42	520.23	R	741.46	723.38	724.43	371.23	6
10	1152.53	1134.53	1135.51	576.77	L	585.36	567.35	568.33	293.18	5
11	1265.62	1247.61	1248.59	633.31	L	472.28	454.27	455.25	236.64	4
12	1380.65	1362.64	1363.62	690.82	D	359.19	341.18	342.17	180.10	3
13	1477.70	1459.69	1460.67	739.35	P	244.17	226.16	227.14	122.58	2
14					K	147.11	129.10	130.09	74.06	1

CNMa_partial

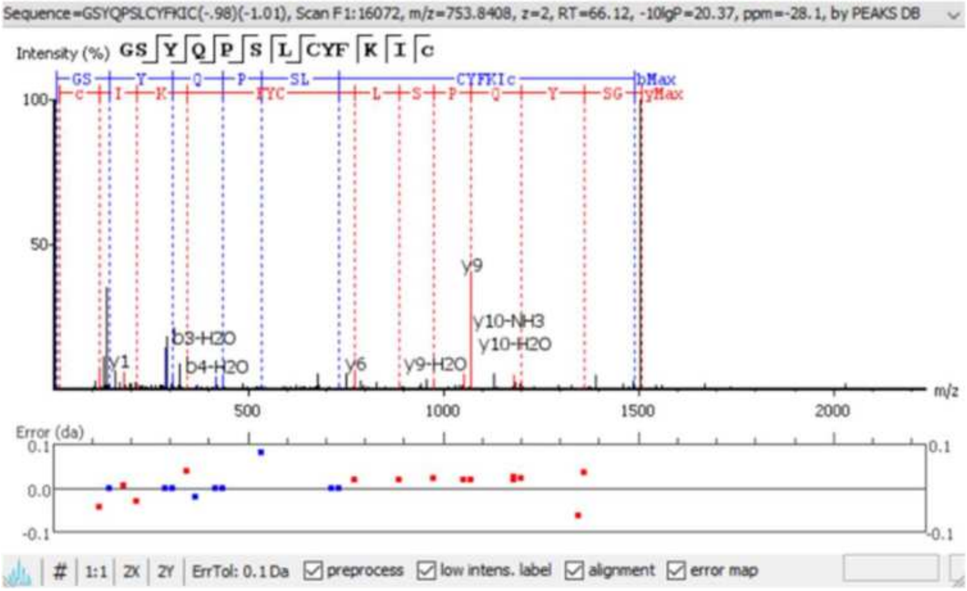

#	b	b-H2O	b-NH3	b (2+)	Seq	y	y-H2O	y-NH3	y (2+)	#
1	58.03	40.02	41.00	29.51	G					13
2	145.06	127.05	128.03	73.03	S	1449.68	1431.67	1432.65	725.34	12
3	308.12	290.11	291.10	154.56	Y	1362.61	1344.64	1345.68	681.82	11
4	436.18	418.17	419.16	218.59	Q	1199.56	1181.55	1182.53	600.29	10
5	533.15	515.23	516.21	267.12	P	1071.50	1053.49	1054.50	536.26	9
6	620.27	602.26	603.24	310.63	S	974.45	956.46	957.44	487.74	8
7	733.35	715.34	716.33	367.20	L	887.42	869.43	870.41	444.22	7
8	836.36	818.35	819.33	418.68	C	774.33	756.34	757.33	387.68	6
9	999.42	981.41	982.40	500.21	Y	671.35	653.34	654.32	336.17	5
10	1146.49	1128.48	1129.47	573.75	F	508.28	490.27	491.26	254.64	4
11	1274.59	1256.58	1257.56	637.79	K	361.21	343.16	344.19	181.10	3
12	1387.67	1369.66	1370.65	694.34	I	233.12	215.14	216.09	117.06	2
13					C(-1.99)	120.08	102.02	103.01	60.52	1

Diuretic hormone-31-PP (DH-31-PP)_partial

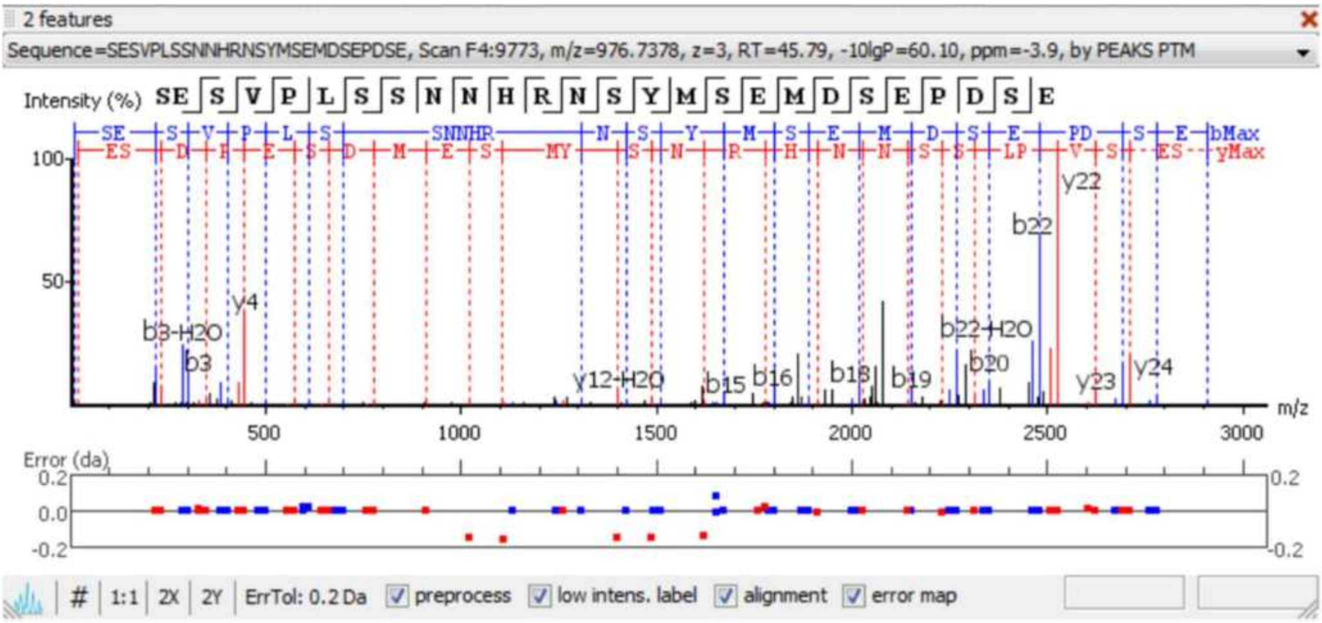

#	b	b-H2O	b-NH3	b (2+)	Seq	y	y-H2O	y-NH3	y (2+)	#
1	88.04	70.03	71.01	44.52	S					26
2	217.08	199.07	200.06	109.04	E	2841.16	2823.15	2824.13	1421.08	25
3	304.11	286.10	287.09	152.56	S	2712.11	2694.10	2695.09	1356.56	24
4	403.18	385.17	386.16	202.09	V	2625.09	2607.05	2608.06	1313.04	23
5	500.23	482.22	483.21	250.62	P	2526.01	2508.00	2508.99	1263.51	22
6	613.29	595.31	596.27	307.16	L	2428.96	2410.95	2411.93	1214.98	21
7	700.35	682.34	683.32	350.68	S	2315.88	2297.87	2298.85	1158.44	20
8	787.38	769.37	770.36	394.19	S	2228.86	2210.83	2211.82	1114.92	19
9	901.43	883.42	884.40	451.21	N	2141.81	2123.80	2124.79	1071.41	18
10	1015.47	997.46	998.44	508.23	N	2027.78	2009.76	2010.74	1014.39	17
11	1152.53	1134.52	1135.50	576.76	H	1913.74	1895.72	1896.70	957.36	16
12	1308.63	1290.62	1291.60	654.81	R	1776.64	1758.67	1759.65	888.83	15
13	1422.67	1404.66	1405.65	711.84	N	1620.71	1602.56	1603.54	810.78	14
14	1509.71	1491.69	1492.68	755.35	S	1506.52	1488.67	1489.50	753.76	13
15	1672.77	1654.77	1655.65	836.88	Y	1419.49	1401.63	1402.47	710.25	12
16	1803.81	1785.79	1786.78	902.40	M	1256.43	1238.42	1239.40	628.71	11
17	1890.83	1872.82	1873.81	945.92	S	1125.39	1107.54	1108.36	563.19	10
18	2019.88	2001.87	2002.86	1010.44	E	1038.36	1020.50	1021.33	519.68	9
19	2150.93	2132.91	2133.90	1075.96	M	909.32	891.30	892.29	455.16	8
20	2265.95	2247.94	2248.92	1133.48	D	778.27	760.27	761.25	389.64	7
21	2352.98	2334.97	2335.96	1176.99	S	663.25	645.24	646.22	332.11	6
22	2482.02	2464.01	2465.00	1241.52	E	576.22	558.21	559.19	288.61	5
23	2579.08	2561.07	2562.05	1290.04	P	447.17	429.16	430.15	224.09	4
24	2694.10	2676.09	2677.08	1347.55	D	350.12	332.11	333.09	175.56	3
25	2781.14	2763.13	2764.11	1391.07	S	235.09	217.08	218.07	118.05	2
26					E	148.06	130.05	131.03	74.53	1

Diuretic hormone-31-PP (DH-31-PP)_part 1_partial

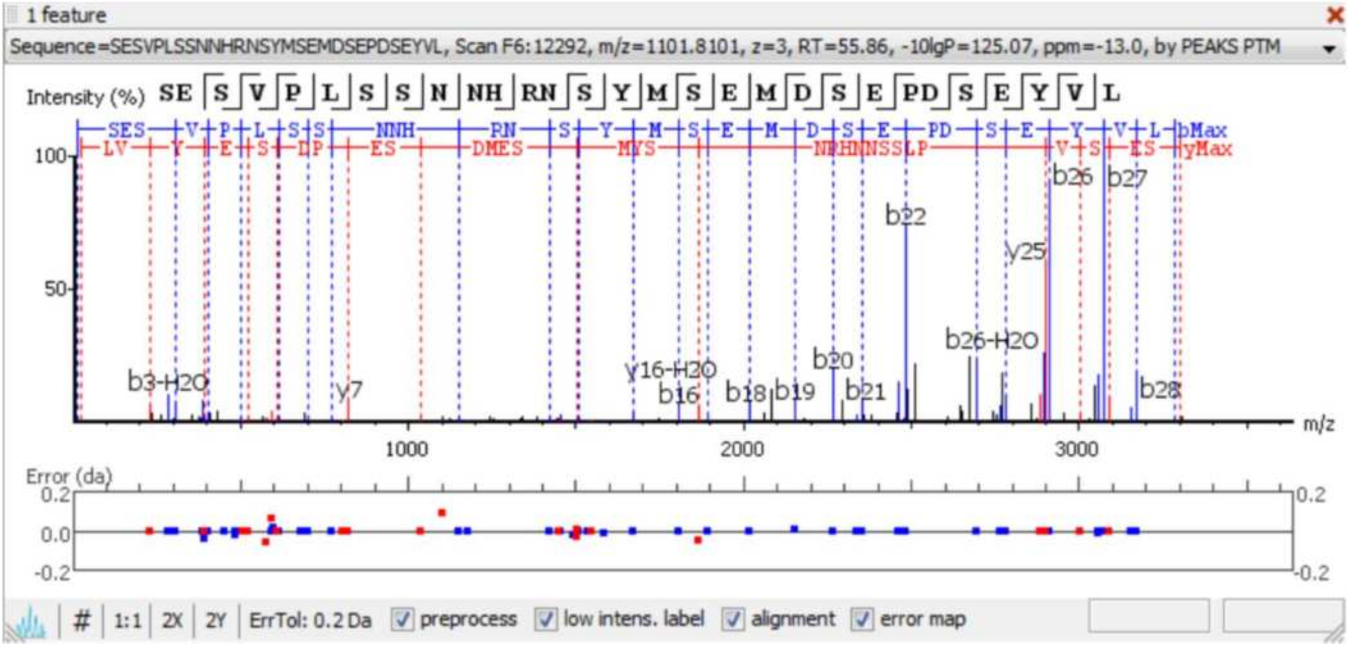

#	b	b-H2O	b-NH3	b (2+)	Seq	y	y-H2O	y-NH3	y (2+)	#
1	88.04	70.03	71.01	44.52	S					29
2	217.08	199.07	200.06	109.04	E	3216.37	3198.36	3199.35	1608.69	28
3	304.11	286.10	287.09	152.56	S	3087.33	3069.32	3070.30	1544.17	27
4	403.18	385.17	386.16	202.09	V	3000.31	2982.29	2983.27	1500.63	26
5	500.24	482.23	483.23	250.62	P	2901.23	2883.21	2884.20	1451.12	25
6	613.33	595.31	596.27	307.16	L	2804.18	2786.17	2787.15	1402.59	24
7	700.35	682.34	683.32	350.68	S	2691.09	2673.08	2674.07	1346.05	23
8	787.38	769.38	770.36	394.23	S	2604.06	2586.05	2587.03	1302.53	22
9	901.43	883.42	884.40	451.22	N	2517.03	2499.02	2500.00	1259.01	21
10	1015.47	997.46	998.44	508.23	N	2402.99	2384.98	2385.96	1201.99	20
11	1152.52	1134.52	1135.50	576.76	H	2288.94	2270.93	2271.92	1144.97	19
12	1308.63	1290.62	1291.60	654.81	R	2151.88	2133.87	2134.86	1076.44	18
13	1422.67	1404.66	1405.65	711.84	N	1995.78	1977.77	1978.76	998.39	17
14	1509.70	1491.69	1492.70	755.35	S	1881.74	1863.78	1864.71	941.37	16
15	1672.77	1654.76	1655.74	836.88	Y	1794.71	1776.70	1777.68	897.85	15
16	1803.81	1785.80	1786.78	902.40	M	1631.65	1613.63	1614.62	816.32	14
17	1890.84	1872.83	1873.81	945.92	S	1500.63	1482.59	1483.58	750.80	13
18	2019.88	2001.87	2002.86	1010.44	E	1413.57	1395.56	1396.55	707.29	12
19	2150.91	2132.91	2133.90	1075.96	M	1284.53	1266.52	1267.50	642.77	11
20	2265.95	2247.94	2248.92	1133.48	D	1153.49	1135.48	1136.46	577.30	10
21	2352.99	2334.97	2335.96	1177.00	S	1038.47	1020.45	1021.44	519.73	9
22	2482.02	2464.02	2465.00	1241.51	E	951.43	933.42	934.40	476.22	8
23	2579.08	2561.07	2562.05	1290.04	P	822.39	804.38	805.36	411.69	7
24	2694.11	2676.09	2677.08	1347.55	D	725.34	707.32	708.31	363.17	6
25	2781.14	2763.13	2764.11	1391.07	S	610.31	592.23	593.28	305.65	5
26	2910.18	2892.17	2893.15	1455.60	E	523.28	505.27	506.25	262.14	4
27	3073.24	3055.23	3056.23	1537.13	Y	394.23	376.22	377.21	197.62	3
28	3172.32	3154.30	3155.28	1586.67	V	231.17	213.16	214.14	116.09	2
29					L	132.10	114.09	115.07	66.55	1

Diuretic hormone-31-PP (DH-31-PP)_part 2_partial

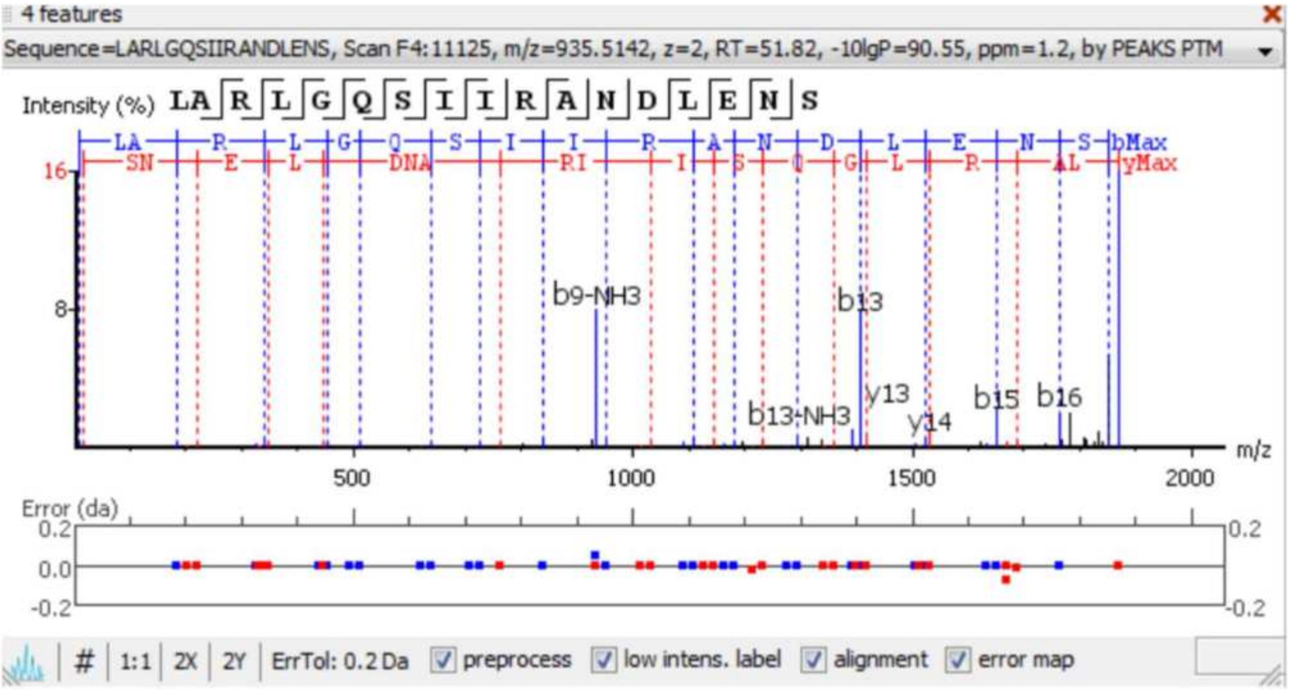

#	b	b-H2O	b-NH3	b (2+)	Seq	y	y-H2O	y-NH3	y (2+)	#
1	114.09	96.08	97.06	57.55	L					17
2	185.13	167.12	168.10	93.06	A	1756.93	1738.92	1739.90	878.96	16
3	341.23	323.22	324.20	171.12	R	1685.90	1667.96	1668.87	843.45	15
4	454.31	436.30	437.29	227.66	L	1529.80	1511.78	1512.77	765.40	14
5	511.34	493.33	494.31	256.17	G	1416.71	1398.70	1399.67	708.85	13
6	639.39	621.38	622.37	320.20	Q	1359.68	1341.68	1342.66	680.34	12
7	726.42	708.42	709.41	363.71	S	1231.63	1213.62	1214.62	616.31	11
8	839.51	821.50	822.48	420.26	I	1144.59	1126.59	1127.58	572.80	10
9	952.59	934.58	935.51	476.80	I	1031.52	1013.50	1014.49	516.26	9
10	1108.70	1090.68	1091.67	554.85	R	918.43	900.42	901.40	459.71	8
11	1179.73	1161.72	1162.71	590.37	A	762.33	744.32	745.30	381.66	7
12	1293.77	1275.77	1276.75	647.39	N	691.29	673.28	674.26	346.14	6
13	1408.80	1390.79	1391.78	704.90	D	577.25	559.24	560.22	289.12	5
14	1521.88	1503.88	1504.86	761.44	L	462.22	444.21	445.19	231.61	4
15	1650.93	1632.92	1633.90	825.96	E	349.13	331.12	332.11	175.07	3
16	1764.96	1746.96	1747.95	882.99	N	220.09	202.08	203.07	110.55	2
17					S	106.05	88.04	89.02	53.52	1

Diuretic hormone-31 (DH-31)

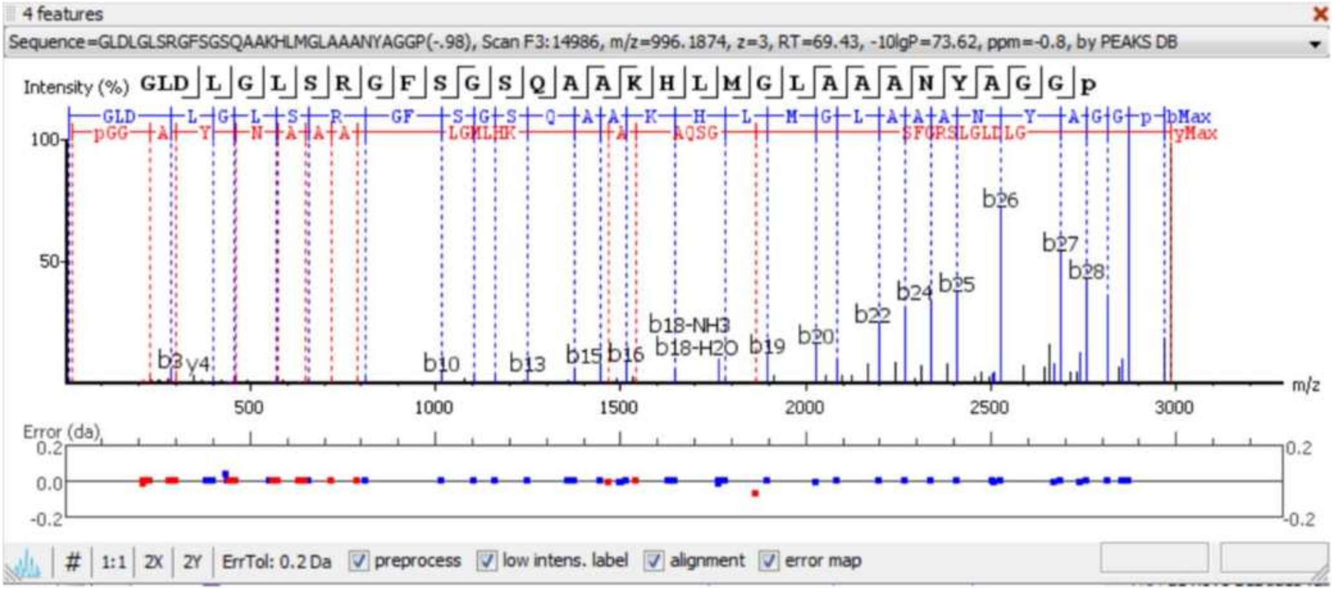

#	b	b-H2O	b-NH3	b (2+)	Seq	y	y-H2O	y-NH3	y (2+)	#
1	58.03	40.02	41.00	29.51	G					31
2	171.11	153.10	154.09	86.06	L	2929.51	2911.50	2912.48	1465.26	30
3	286.14	268.13	269.11	143.57	D	2816.43	2798.42	2799.40	1408.71	29
4	399.22	381.21	382.19	200.11	L	2701.40	2683.39	2684.37	1351.20	28
5	456.25	438.24	439.21	228.62	G	2588.31	2570.30	2571.29	1294.66	27
6	569.33	551.32	552.30	285.16	L	2531.29	2513.28	2514.27	1266.15	26
7	656.36	638.35	639.33	328.68	S	2418.21	2400.20	2401.18	1209.60	25
8	812.47	794.45	795.44	406.73	R	2331.18	2313.17	2314.15	1166.09	24
9	869.48	851.47	852.46	435.20	G	2175.08	2157.07	2158.05	1088.04	23
10	1016.56	998.54	999.53	508.78	F	2118.05	2100.04	2101.03	1059.53	22
11	1103.59	1085.57	1086.56	552.30	S	1970.99	1952.98	1953.96	985.99	21
12	1160.61	1142.60	1143.58	580.80	G	1883.95	1865.94	1867.00	942.48	20
13	1247.64	1229.63	1230.61	624.32	S	1826.93	1808.92	1809.91	913.97	19
14	1375.70	1357.69	1358.67	688.35	Q	1739.90	1721.89	1722.87	870.45	18
15	1446.74	1428.72	1429.71	723.87	A	1611.84	1593.83	1594.82	806.42	17
16	1517.77	1499.77	1500.76	759.39	A	1540.81	1522.79	1523.78	770.90	16
17	1645.87	1627.85	1628.84	823.43	K	1469.78	1451.76	1452.74	735.38	15
18	1782.93	1764.92	1765.92	891.96	H	1341.67	1323.66	1324.65	671.34	14
19	1896.01	1878.00	1878.98	948.50	L	1204.61	1186.60	1187.59	602.81	13
20	2027.06	2009.04	2010.02	1014.02	M	1091.53	1073.52	1074.50	546.27	12
21	2084.08	2066.06	2067.04	1042.54	G	960.49	942.48	943.46	480.74	11
22	2197.16	2179.14	2180.13	1099.08	L	903.47	885.46	886.44	452.23	10
23	2268.20	2250.18	2251.17	1134.60	A	790.39	772.37	773.36	395.69	9
24	2339.23	2321.22	2322.20	1170.11	A	719.35	701.34	702.32	360.17	8
25	2410.27	2392.26	2393.24	1205.63	A	648.31	630.30	631.28	324.65	7
26	2524.31	2506.29	2507.30	1262.65	N	577.27	559.26	560.25	289.14	6
27	2687.38	2669.36	2670.36	1344.19	Y	463.23	445.22	446.20	232.11	5
28	2758.41	2740.40	2741.40	1379.70	A	300.17	282.16	283.14	150.58	4
29	2815.43	2797.42	2798.40	1408.22	G	229.13	211.14	212.10	115.06	3
30	2872.45	2854.44	2855.43	1436.73	G	172.11	154.10	155.08	86.55	2
31					P(-.98)	115.09	97.08	98.06	58.04	1

Diuretic hormone-46 (DH-46)-PP-1

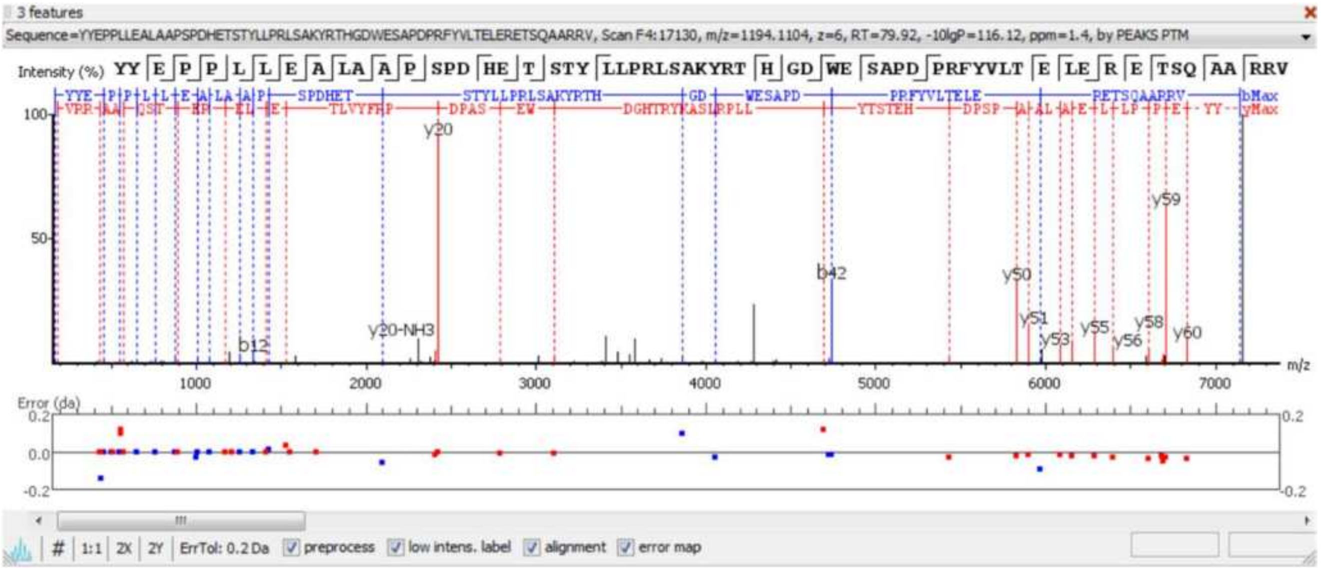

#	b	b-H2O	b-NH3	b (2+)	Seq	y	y-H2O	y-NH3	y (2+)	#
1	164.07	146.06	147.04	82.54	Y					62
2	327.13	309.12	310.11	164.07	Y	6996.53	6978.52	6979.51	3498.77	61
3	456.18	438.17	439.29	228.59	E	6833.51	6815.46	6816.44	3417.24	60
4	553.23	535.22	536.20	277.11	P	6704.46	6686.44	6687.45	3352.71	59
5	650.28	632.27	633.26	325.64	P	6607.41	6589.36	6590.35	3304.19	58
6	763.36	745.36	746.34	382.18	L	6510.32	6492.31	6493.30	3255.66	57
7	876.45	858.44	859.42	438.73	L	6397.27	6379.23	6380.21	3199.12	56
8	1005.49	987.48	988.47	503.25	E	6284.18	6266.14	6267.13	3142.58	55
9	1076.54	1058.52	1059.50	538.77	A	6155.13	6137.10	6138.08	3078.06	54
10	1189.61	1171.60	1172.59	595.31	L	6084.09	6066.06	6067.05	3042.54	53
11	1260.66	1242.64	1243.62	630.83	A	5970.99	5952.98	5953.96	2986.00	52
12	1331.69	1313.68	1314.66	666.34	A	5899.97	5881.94	5882.93	2950.48	51
13	1428.72	1410.73	1411.71	714.87	P	5828.94	5810.91	5811.89	2914.96	50
14	1515.77	1497.76	1498.75	758.39	S	5731.86	5713.85	5714.84	2866.43	49
15	1612.83	1594.82	1595.80	806.91	P	5644.83	5626.82	5627.80	2822.92	48
16	1727.85	1709.84	1710.83	864.43	D	5547.78	5529.77	5530.75	2774.39	47
17	1864.91	1846.90	1847.89	932.96	H	5432.78	5414.74	5415.73	2716.88	46
18	1993.95	1975.94	1976.93	997.51	E	5295.69	5277.68	5278.67	2648.35	45
19	2095.06	2076.99	2077.98	1048.00	T	5166.65	5148.64	5149.62	2583.83	44
20	2182.03	2164.02	2165.01	1091.52	S	5065.60	5047.59	5048.58	2533.30	43
21	2283.08	2265.07	2266.05	1142.04	T	4978.57	4960.56	4961.54	2489.79	42
22	2446.15	2428.13	2429.12	1223.57	Y	4877.52	4859.51	4860.50	2439.26	41
23	2559.23	2541.22	2542.20	1280.11	L	4714.46	4696.32	4697.43	2357.73	40
24	2672.31	2654.30	2655.29	1336.66	L	4601.38	4583.37	4584.35	2301.19	39
25	2769.37	2751.36	2752.34	1385.18	P	4488.29	4470.28	4471.26	2244.65	38
26	2925.47	2907.46	2908.44	1463.23	R	4391.24	4373.23	4374.21	2196.12	37
27	3038.55	3020.54	3021.52	1519.78	L	4235.14	4217.13	4218.11	2118.07	36
28	3125.58	3107.57	3108.56	1563.29	S	4122.05	4104.04	4105.03	2061.53	35
29	3196.62	3178.61	3179.59	1598.81	A	4035.02	4017.01	4017.99	2018.01	34
30	3324.72	3306.71	3307.69	1662.86	K	3963.98	3945.97	3946.96	1982.49	33
31	3487.78	3469.77	3470.75	1744.39	Y	3835.89	3817.88	3818.86	1918.44	32
32	3643.88	3625.87	3626.85	1822.44	R	3672.83	3654.82	3655.80	1836.91	31
33	3744.93	3726.92	3727.90	1872.96	T	3516.73	3498.71	3499.70	1758.86	30
34	3881.99	3863.87	3864.96	1941.49	H	3415.68	3397.67	3398.65	1708.34	29
35	3939.01	3921.00	3921.98	1970.00	G	3278.62	3260.61	3261.59	1639.81	28
36	4054.07	4036.02	4037.01	2027.52	D	3221.60	3203.59	3204.57	1611.30	27
37	4240.11	4222.10	4223.09	2120.56	W	3106.58	3088.56	3089.54	1553.78	26
38	4369.16	4351.15	4352.13	2185.08	E	2920.49	2902.48	2903.46	1460.75	25
39	4456.19	4438.18	4439.16	2228.59	S	2791.46	2773.44	2774.42	1396.22	24
40	4527.23	4509.22	4510.20	2264.11	A	2704.42	2686.41	2687.39	1352.71	23
41	4624.28	4606.27	4607.25	2312.64	P	2633.38	2615.37	2616.35	1317.19	22
42	4739.33	4721.31	4722.28	2370.15	D	2536.33	2518.32	2519.30	1268.66	21
43	4836.36	4818.35	4819.33	2418.68	P	2421.30	2403.29	2404.29	1211.16	20
44	4992.46	4974.45	4975.43	2496.73	R	2324.25	2306.24	2307.22	1162.62	19
45	5139.53	5121.52	5122.50	2570.26	F	2168.15	2150.14	2151.12	1084.57	18
46	5302.59	5284.58	5285.56	2651.80	Y	2021.08	2003.07	2004.05	1011.04	17
47	5401.66	5383.65	5384.63	2701.33	V	1858.01	1840.00	1840.99	929.51	16
48	5514.74	5496.73	5497.72	2757.87	L	1758.95	1740.94	1741.92	879.97	15
49	5615.79	5597.78	5598.76	2808.40	T	1645.86	1627.85	1628.83	823.43	14
50	5744.83	5726.82	5727.81	2872.92	E	1544.81	1526.76	1527.79	772.91	13
51	5857.92	5839.91	5840.89	2929.46	L	1415.76	1397.76	1398.74	708.39	12
52	5986.96	5968.95	5970.03	2993.98	E	1302.69	1284.68	1285.66	651.84	11
53	6143.06	6125.05	6126.03	3072.03	R	1173.65	1155.63	1156.62	587.32	10
54	6272.10	6254.09	6255.08	3136.55	E	1017.54	999.53	1000.52	509.28	9
55	6373.15	6355.14	6356.12	3187.08	T	888.50	870.49	871.47	444.75	8
56	6460.18	6442.17	6443.16	3230.59	S	787.45	769.44	770.43	394.23	7
57	6588.24	6570.23	6571.22	3294.62	Q	700.42	682.41	683.39	350.71	6
58	6659.28	6641.27	6642.25	3330.14	A	572.37	554.23	555.23	286.68	5
59	6730.32	6712.31	6713.29	3365.66	A	501.33	483.32	484.30	251.16	4
60	6886.42	6868.41	6869.39	3443.71	R	430.29	412.28	413.26	215.64	3
61	7042.52	7024.51	7025.49	3521.76	R	274.19	256.18	257.16	137.59	2
62					V	118.09	100.08	101.06	59.54	1

Diuretic hormone-46 (DH-46)_part 1

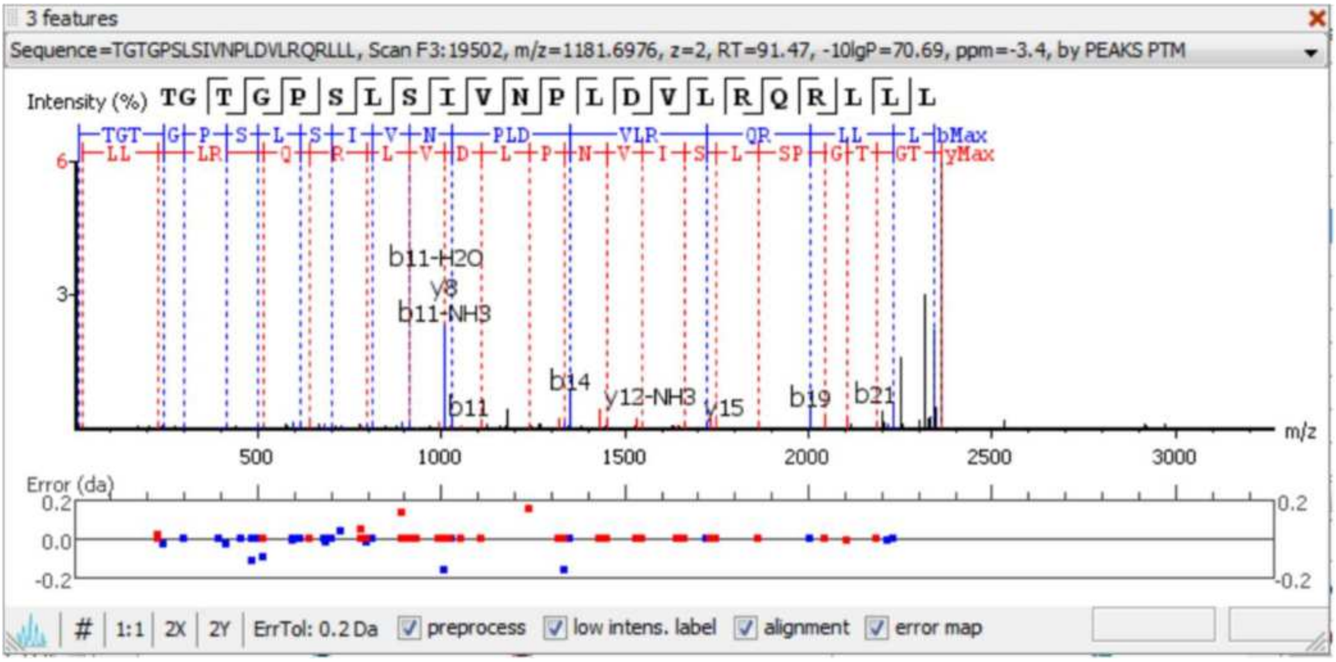

#	b	b-H2O	b-NH3	b (2+)	Seq	y	y-H2O	y-NH3	y (2+)	#
1	102.06	84.04	85.03	51.53	T					22
2	159.08	141.07	142.05	80.04	G	2261.33	2243.32	2244.31	1131.17	21
3	260.12	242.15	243.10	130.56	T	2204.31	2186.30	2187.28	1102.66	20
4	317.15	299.13	300.12	159.07	G	2103.28	2085.25	2086.24	1052.13	19
5	414.23	396.19	397.17	207.60	P	2046.24	2028.23	2029.22	1023.63	18
6	501.23	483.22	484.32	251.12	S	1949.19	1931.18	1932.16	975.10	17
7	614.32	596.30	597.30	307.66	L	1862.16	1844.15	1845.13	931.58	16
8	701.34	683.34	684.34	351.17	S	1749.07	1731.06	1732.05	875.04	15
9	814.43	796.42	797.42	407.72	I	1662.04	1644.03	1645.02	831.52	14
10	913.50	895.49	896.47	457.24	V	1548.96	1530.95	1531.93	774.98	13
11	1027.54	1009.53	1010.69	514.37	N	1449.88	1431.88	1432.87	725.44	12
12	1124.60	1106.58	1107.57	562.80	P	1335.85	1317.84	1318.83	668.42	11
13	1237.68	1219.67	1220.65	619.34	L	1238.63	1220.78	1221.77	619.90	10
14	1352.71	1334.69	1335.85	676.85	D	1125.71	1107.70	1108.69	563.36	9
15	1451.77	1433.76	1434.75	726.34	V	1010.69	992.67	993.66	505.84	8
16	1564.86	1546.85	1547.83	782.93	L	911.61	893.47	894.59	456.31	7
17	1720.96	1702.95	1703.93	860.98	R	798.53	780.46	781.51	399.77	6
18	1849.02	1831.01	1831.99	925.01	Q	642.43	624.42	625.40	321.71	5
19	2005.12	1987.11	1988.09	1003.06	R	514.37	496.36	497.34	257.69	4
20	2118.20	2100.19	2101.18	1059.60	L	358.27	340.26	341.24	179.64	3
21	2231.29	2213.28	2214.28	1116.14	L	245.19	227.18	228.13	123.09	2
22					L	132.10	114.09	115.07	66.55	1

Diuretic hormone-46 (DH-46)_part 2

Diuretic hormone-46$^{31-46}$ (DH-46$^{31-46}$)

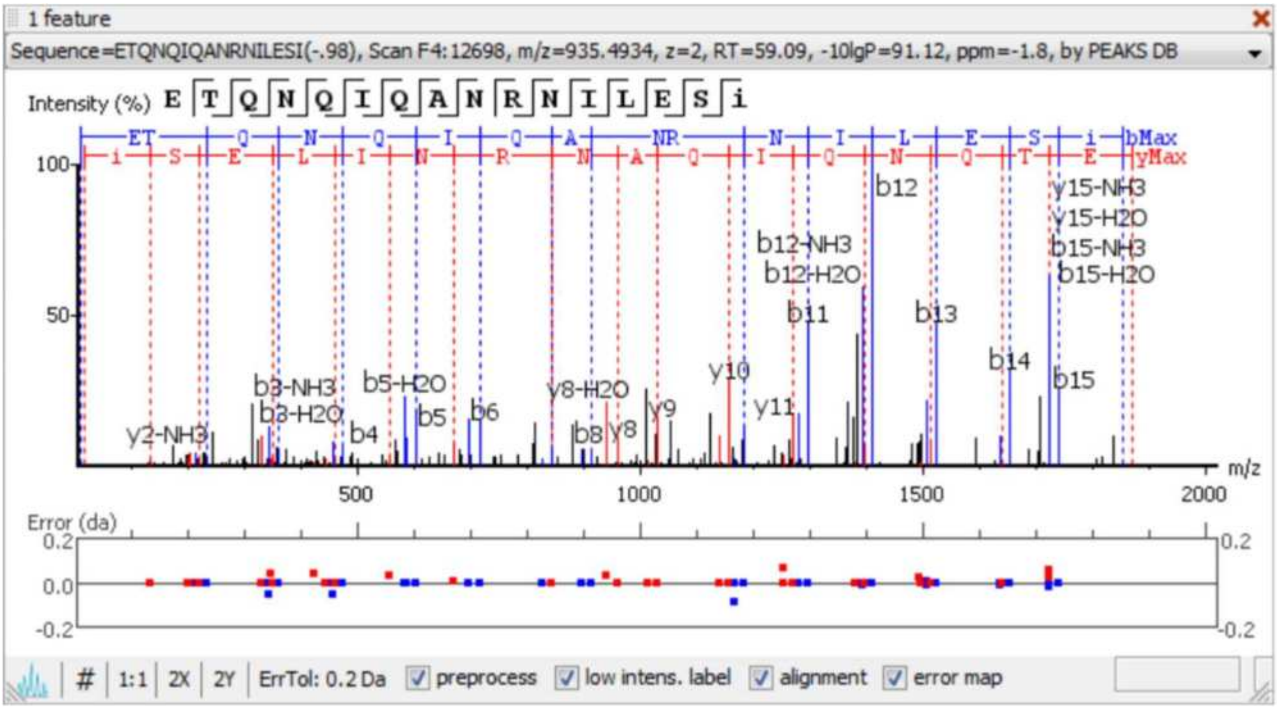

#	b	b-H2O	b-NH3	b (2+)	Seq	y	y-H2O	y-NH3	y (2+)	#
1	130.05	112.04	113.02	65.53	E					16
2	231.10	213.09	214.07	116.05	T	1740.94	1722.86	1723.88	870.97	15
3	359.16	341.15	342.19	180.08	Q	1639.90	1621.88	1622.86	820.44	14
4	473.20	455.19	456.23	237.10	N	1511.83	1493.79	1494.80	756.41	13
5	601.26	583.25	584.24	301.13	Q	1397.79	1379.78	1380.76	699.39	12
6	714.34	696.33	697.32	357.67	I	1269.73	1251.65	1252.70	635.36	11
7	842.40	824.39	825.37	421.70	Q	1156.64	1138.64	1139.62	578.82	10
8	913.44	895.42	896.41	457.22	A	1028.59	1010.57	1011.56	514.79	9
9	1027.48	1009.47	1010.45	514.24	N	957.55	939.50	940.52	479.27	8
10	1183.58	1165.57	1166.64	592.29	R	843.50	825.49	826.48	422.20	7
11	1297.62	1279.61	1280.61	649.31	N	687.40	669.38	670.38	344.16	6
12	1410.71	1392.70	1393.69	705.85	I	573.36	555.35	556.30	287.18	5
13	1523.80	1505.77	1506.78	762.40	L	460.28	442.27	443.25	230.64	4
14	1652.84	1634.82	1635.82	826.92	E	347.19	329.18	330.17	174.10	3
15	1739.87	1721.86	1722.86	870.43	S	218.15	200.14	201.12	109.57	2
16					I(-.98)	131.12	113.11	114.09	66.06	1

Diuretic hormone-46 (DH-46)-PP-2_part 1

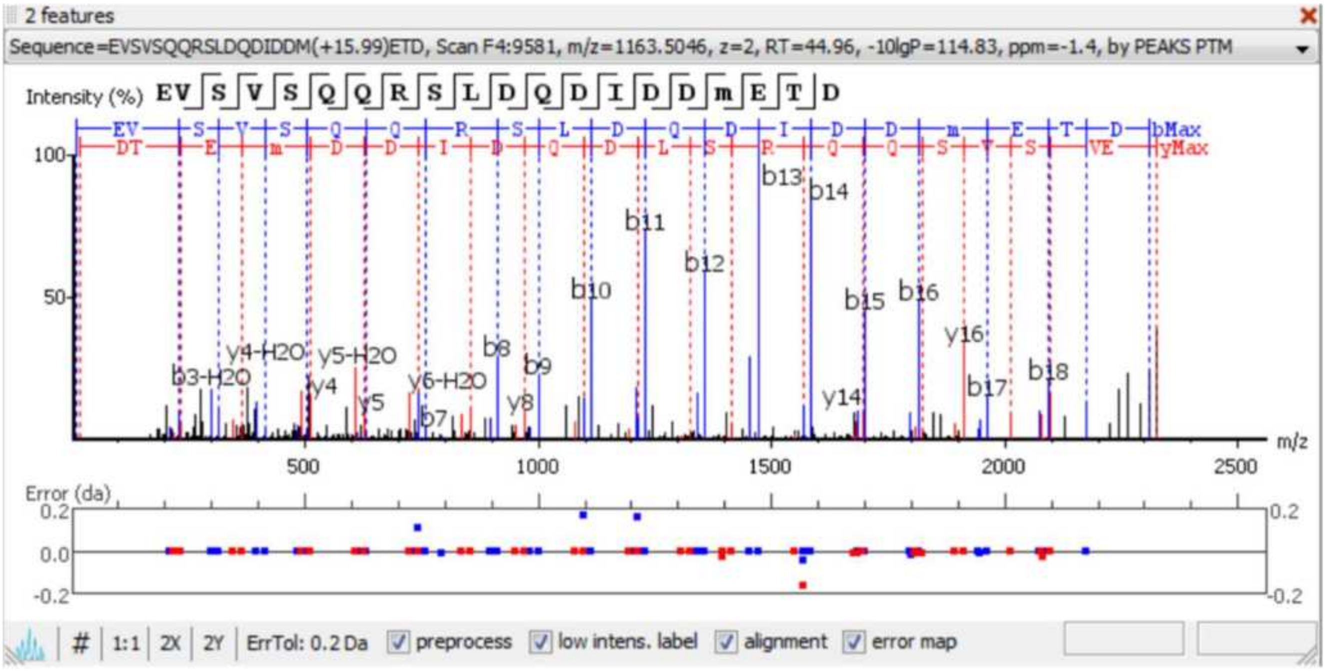

#	b	b-H2O	b-NH3	b (2+)	Seq	y	y-H2O	y-NH3	y (2+)	#
1	130.05	112.04	113.02	65.53	E					20
2	229.12	211.11	212.09	115.06	V	2196.96	2178.95	2179.93	1098.98	19
3	316.15	298.14	299.12	158.58	S	2097.89	2079.87	2080.89	1049.44	18
4	415.22	397.21	398.19	208.11	V	2010.86	1992.85	1993.83	1005.93	17
5	502.25	484.24	485.22	251.63	S	1911.79	1893.78	1894.76	956.39	16
6	630.31	612.30	613.28	315.65	Q	1824.76	1806.75	1807.74	912.88	15
7	758.37	740.36	741.22	379.68	Q	1696.70	1678.70	1679.68	848.85	14
8	914.47	896.46	897.44	457.73	R	1568.80	1550.62	1551.61	784.82	13
9	1001.50	983.49	984.47	501.25	S	1412.54	1394.52	1395.54	706.77	12
10	1114.59	1096.58	1097.39	557.79	L	1325.50	1307.50	1308.48	663.25	11
11	1229.61	1211.60	1212.42	615.31	D	1212.42	1194.41	1195.39	606.71	10
12	1357.67	1339.66	1340.64	679.34	Q	1097.39	1079.38	1080.37	549.20	9
13	1472.70	1454.69	1455.67	736.85	D	969.34	951.33	952.31	485.17	8
14	1585.78	1567.77	1568.80	793.40	I	854.31	836.30	837.28	427.65	7
15	1700.81	1682.80	1683.80	850.90	D	741.22	723.21	724.20	371.11	6
16	1815.84	1797.83	1798.83	908.42	D	626.20	608.19	609.17	313.60	5
17	1962.88	1944.86	1945.85	981.94	M(+15.99)	511.17	493.16	494.14	256.09	4
18	2091.92	2073.90	2074.89	1046.46	E	364.14	346.12	347.11	182.57	3
19	2192.96	2174.95	2175.93	1096.98	T	235.09	217.08	218.07	118.05	2
20					D	134.04	116.03	117.02	67.52	1

Diuretic hormone-46 (DH-46)-PP-2_part 2

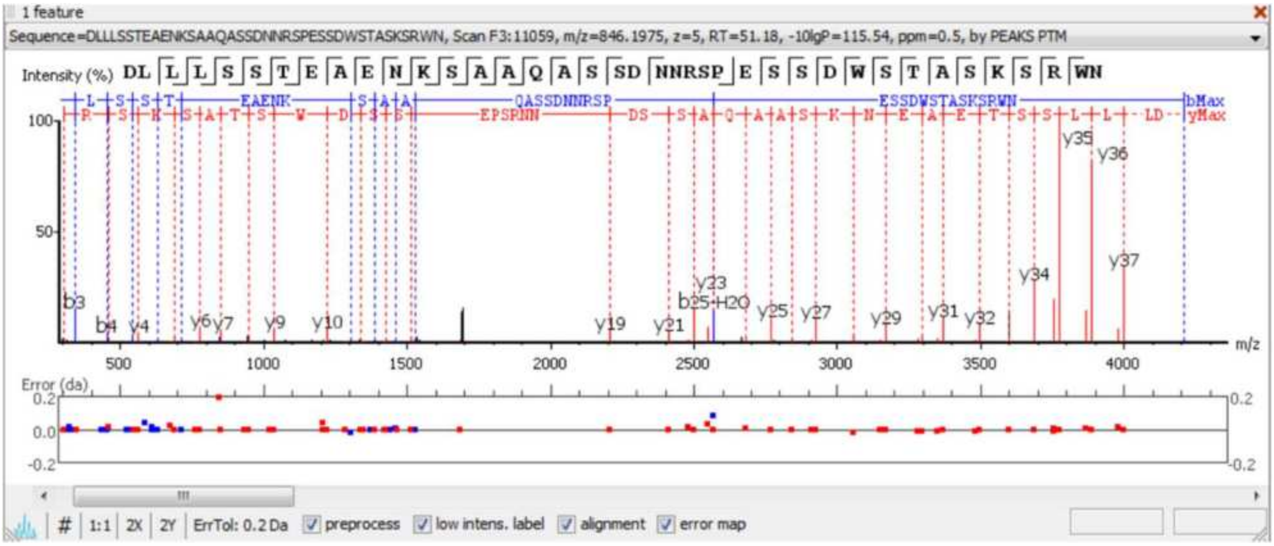

#	b	b-H2O	b-NH3	b (2+)	Seq	y	y-H2O	y-NH3	y (2+)	#
1	116.03	98.02	99.01	58.52	D					39
2	229.12	211.11	212.09	115.06	L	4111.92	4093.91	4094.89	2056.46	38
3	342.20	324.19	325.15	171.60	L	3998.83	3980.80	3981.81	1999.92	37
4	455.29	437.28	438.26	228.14	L	3885.75	3867.73	3868.72	1943.38	36
5	542.32	524.31	525.29	271.66	S	3772.66	3754.65	3755.65	1886.83	35
6	629.35	611.34	612.30	315.18	S	3685.63	3667.62	3668.61	1843.32	34
7	730.40	712.39	713.37	365.70	T	3598.60	3580.59	3581.58	1799.80	33
8	859.44	841.43	842.41	430.22	E	3497.55	3479.54	3480.54	1749.28	32
9	930.48	912.47	913.45	465.74	A	3368.51	3350.50	3351.49	1684.76	31
10	1059.52	1041.51	1042.49	530.26	E	3297.49	3279.46	3280.46	1649.24	30
11	1173.56	1155.55	1156.54	587.23	N	3168.43	3150.41	3151.41	1584.72	29
12	1301.68	1283.65	1284.63	651.33	K	3054.41	3036.38	3037.36	1527.69	28
13	1388.70	1370.68	1371.66	694.85	S	2926.29	2908.28	2909.27	1463.64	27
14	1459.72	1441.72	1442.70	730.36	A	2839.26	2821.25	2822.24	1420.13	26
15	1530.77	1512.75	1513.74	765.88	A	2768.22	2750.21	2751.20	1384.61	25
16	1658.82	1640.81	1641.80	829.91	Q	2697.19	2679.18	2680.15	1349.09	24
17	1729.86	1711.85	1712.83	865.43	A	2569.13	2551.08	2552.10	1285.07	23
18	1816.89	1798.88	1799.87	908.95	S	2498.10	2480.06	2481.07	1249.55	22
19	1903.92	1885.91	1886.90	952.46	S	2411.07	2393.05	2394.03	1206.03	21
20	2018.95	2000.94	2001.92	1009.98	D	2324.03	2306.02	2307.00	1162.51	20
21	2132.99	2114.98	2115.97	1067.00	N	2209.01	2190.99	2191.97	1105.00	19
22	2247.04	2229.03	2230.01	1124.02	N	2094.96	2076.95	2077.93	1047.98	18
23	2403.14	2385.13	2386.11	1202.07	R	1980.92	1962.91	1963.89	990.96	17
24	2490.17	2472.16	2473.14	1245.59	S	1824.81	1806.80	1807.79	912.91	16
25	2587.22	2569.13	2570.20	1294.11	P	1737.78	1719.77	1720.76	869.39	15
26	2716.27	2698.26	2699.24	1358.63	E	1640.73	1622.72	1623.70	820.86	14
27	2803.30	2785.29	2786.27	1402.15	S	1511.68	1493.68	1494.66	756.34	13
28	2890.33	2872.32	2873.30	1445.67	S	1424.66	1406.64	1407.63	712.83	12
29	3005.36	2987.35	2988.33	1503.18	D	1337.62	1319.61	1320.60	669.31	11
30	3191.44	3173.43	3174.41	1596.22	W	1222.60	1204.54	1205.57	611.80	10
31	3278.47	3260.46	3261.44	1639.73	S	1036.52	1018.51	1019.50	518.76	9
32	3379.52	3361.51	3362.49	1690.26	T	949.48	931.47	932.46	475.24	8
33	3450.55	3432.54	3433.53	1725.78	A	848.44	830.43	831.41	424.72	7
34	3537.59	3519.57	3520.56	1769.29	S	777.40	759.39	760.37	389.20	6
35	3665.68	3647.67	3648.65	1833.34	K	690.37	672.33	673.34	345.69	5
36	3752.71	3734.70	3735.69	1876.86	S	562.27	544.26	545.25	281.64	4
37	3908.81	3890.80	3891.79	1954.91	R	475.24	457.23	458.19	238.12	3
38	4094.89	4076.88	4077.87	2047.95	W	319.14	301.13	302.11	160.07	2
39					N	133.06	115.05	116.03	67.03	1

Diuretic hormone-46 (DH-46)-PP-2_part 3

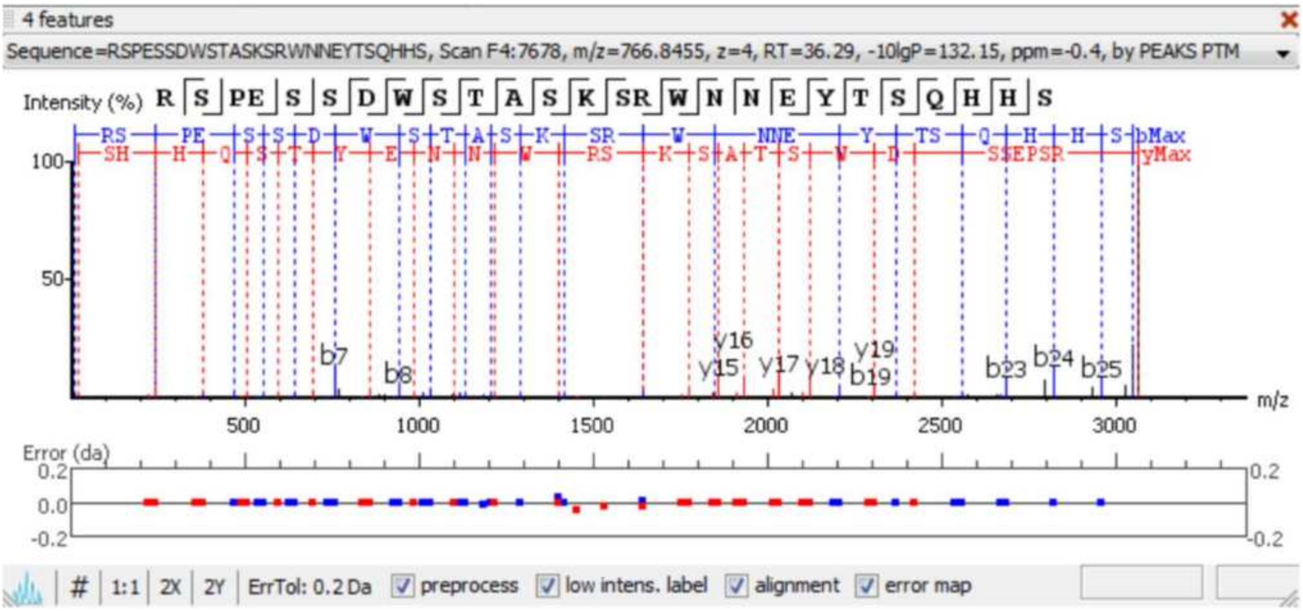

#	b	b-H2O	b-NH3	b (2+)	Seq	y	y-H2O	y-NH3	y (2+)	#
1	157.11	139.10	140.08	79.05	R					26
2	244.14	226.13	227.11	122.57	S	2908.25	2890.24	2891.22	1454.68	25
3	341.19	323.18	324.17	171.10	P	2821.22	2803.21	2804.19	1411.11	24
4	470.24	452.23	453.21	235.62	E	2724.17	2706.16	2707.14	1362.58	23
5	557.27	539.26	540.24	279.13	S	2595.12	2577.11	2578.10	1298.06	22
6	644.30	626.29	627.27	322.65	S	2508.09	2490.08	2491.07	1254.55	21
7	759.33	741.32	742.30	380.17	D	2421.06	2403.05	2404.03	1211.03	20
8	945.41	927.40	928.38	473.20	W	2306.04	2288.02	2289.01	1153.52	19
9	1032.44	1014.43	1015.41	516.72	S	2119.96	2101.95	2102.93	1060.48	18
10	1133.49	1115.48	1116.46	567.24	T	2032.93	2014.91	2015.90	1016.96	17
11	1204.53	1186.53	1187.50	602.76	A	1931.88	1913.86	1914.85	966.44	16
12	1291.55	1273.54	1274.53	646.28	S	1860.84	1842.83	1843.81	930.92	15
13	1419.66	1401.64	1402.58	710.33	K	1773.81	1755.80	1756.78	887.40	14
14	1506.68	1488.67	1489.66	753.84	S	1645.73	1627.70	1628.68	823.36	13
15	1662.78	1644.77	1645.73	831.89	R	1558.68	1540.67	1541.65	779.84	12
16	1848.86	1830.85	1831.84	924.93	W	1402.58	1384.57	1385.55	701.79	11
17	1962.91	1944.90	1945.88	981.95	N	1216.49	1198.49	1199.47	608.75	10
18	2076.95	2058.94	2059.92	1038.97	N	1102.46	1084.44	1085.43	551.73	9
19	2205.99	2187.98	2188.96	1103.50	E	988.42	970.40	971.38	494.71	8
20	2369.06	2351.04	2352.03	1185.03	Y	859.37	841.37	842.34	430.18	7
21	2470.10	2452.09	2453.08	1235.55	T	696.31	678.30	679.28	348.65	6
22	2557.14	2539.13	2540.11	1279.07	S	595.26	577.25	578.23	298.13	5
23	2685.19	2667.18	2668.17	1343.10	Q	508.23	490.22	491.20	254.61	4
24	2822.26	2804.24	2805.22	1411.64	H	380.17	362.16	363.14	190.58	3
25	2959.32	2941.30	2942.28	1480.16	H	243.11	225.10	226.08	122.05	2
26					S	106.05	88.04	89.02	53.52	1

extended FMRFamides-1 (FMRFa-1)_partial

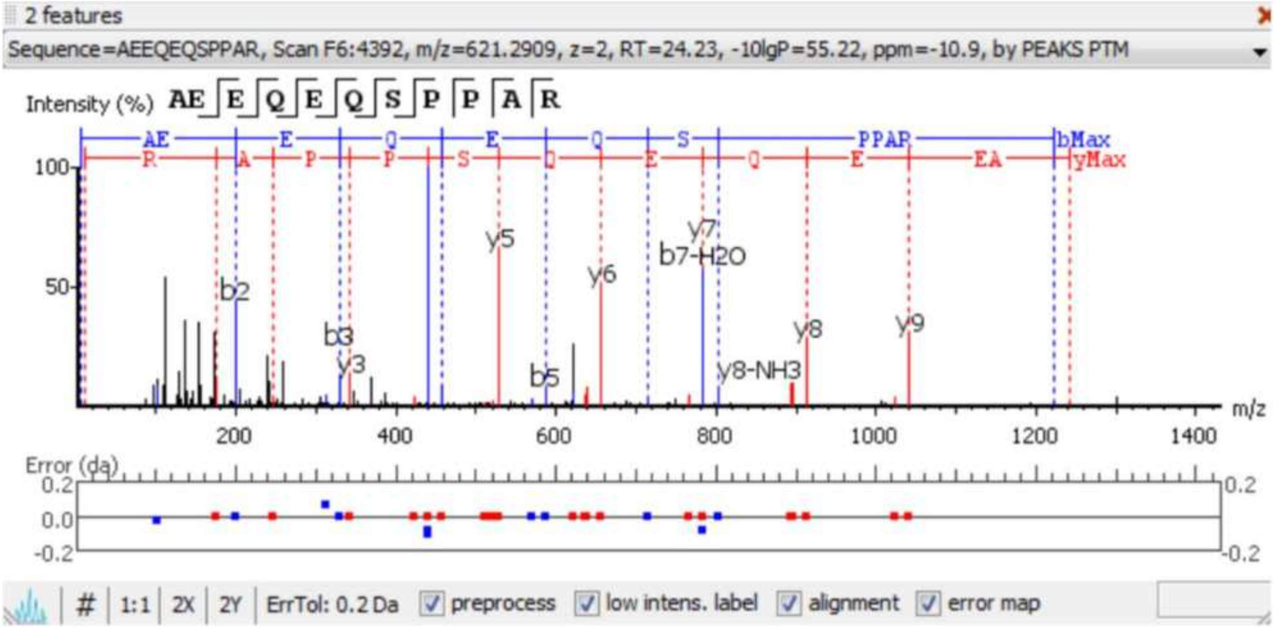

#	b	b-H2O	b-NH3	b (2+)	Seq	y	y-H2O	y-NH3	y (2+)	#
1	72.04	54.03	55.02	36.52	A					11
2	201.09	183.08	184.06	101.07	E	1170.54	1152.53	1153.51	585.77	10
3	330.13	312.05	313.10	165.57	E	1041.50	1023.48	1024.48	521.25	9
4	458.19	440.26	441.27	229.59	Q	912.45	894.44	895.43	456.73	8
5	587.23	569.22	570.20	294.12	E	784.39	766.38	767.38	392.70	7
6	715.29	697.28	698.26	358.14	Q	655.35	637.34	638.33	328.18	6
7	802.32	784.39	785.29	401.66	S	527.29	509.28	510.27	264.15	5
8	899.37	881.36	882.35	450.19	P	440.26	422.25	423.23	220.63	4
9	996.43	978.42	979.40	498.71	P	343.21	325.20	326.18	172.10	3
10	1067.46	1049.45	1050.44	534.23	A	246.16	228.15	229.13	123.58	2
11					R	175.12	157.11	158.09	88.06	1

extended FMRFamides-PP-2 (FMRFa-PP-2)

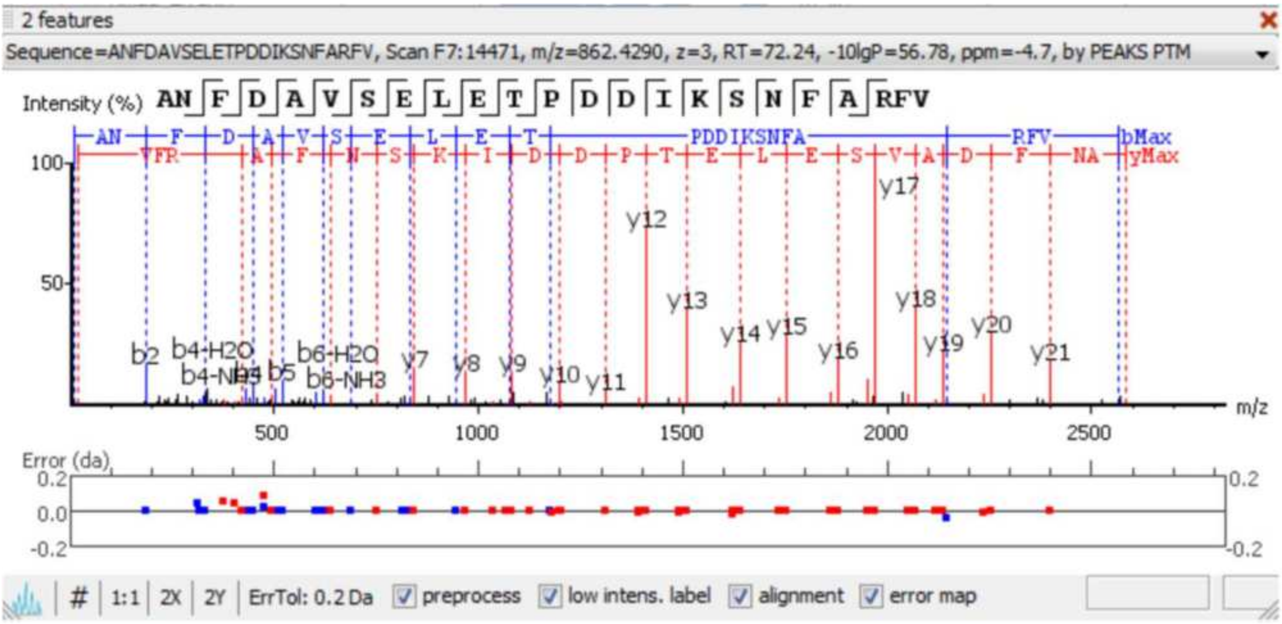

#	b	b-H2O	b-NH3	b (2+)	Seq	y	y-H2O	y-NH3	y (2+)	#
1	72.04	54.03	55.02	36.52	A					23
2	186.09	168.08	169.06	93.54	N	2514.21	2496.20	2497.19	1257.61	22
3	333.16	315.10	316.13	167.08	F	2400.17	2382.16	2383.14	1200.59	21
4	448.18	430.17	431.16	224.59	D	2253.11	2235.11	2236.08	1127.06	20
5	519.22	501.21	502.19	260.11	A	2138.07	2120.07	2121.05	1069.54	19
6	618.29	600.28	601.26	309.64	V	2067.04	2049.03	2050.01	1034.02	18
7	705.32	687.31	688.29	353.16	S	1967.98	1949.96	1950.94	984.49	17
8	834.36	816.35	817.34	417.68	E	1880.94	1862.93	1863.91	940.97	16
9	947.45	929.44	930.42	474.20	L	1751.90	1733.89	1734.88	876.45	15
10	1076.49	1058.48	1059.46	538.74	E	1638.81	1620.80	1621.81	819.91	14
11	1177.54	1159.53	1160.51	589.27	T	1509.77	1491.76	1492.76	755.38	13
12	1274.59	1256.58	1257.56	637.80	P	1408.72	1390.72	1391.71	704.86	12
13	1389.62	1371.61	1372.59	695.31	D	1311.67	1293.66	1294.64	656.33	11
14	1504.64	1486.63	1487.62	752.82	D	1196.65	1178.63	1179.63	598.82	10
15	1617.73	1599.72	1600.70	809.36	I	1081.62	1063.60	1064.59	541.31	9
16	1745.82	1727.81	1728.80	873.41	K	968.53	950.52	951.50	484.77	8
17	1832.86	1814.84	1815.83	916.93	S	840.44	822.43	823.41	420.72	7
18	1946.90	1928.89	1929.87	973.95	N	753.40	735.39	736.38	377.15	6
19	2093.97	2075.96	2076.94	1047.48	F	639.36	621.35	622.33	320.18	5
20	2165.00	2147.04	2147.98	1083.00	A	492.29	474.20	475.27	246.65	4
21	2321.10	2303.09	2304.08	1161.05	R	421.26	403.20	404.23	211.13	3
22	2468.17	2450.16	2451.15	1234.59	F	265.15	247.14	248.13	133.08	2
23					V	118.09	100.08	101.06	59.54	1

extended FMRFamides-2 (FMRFa-2)

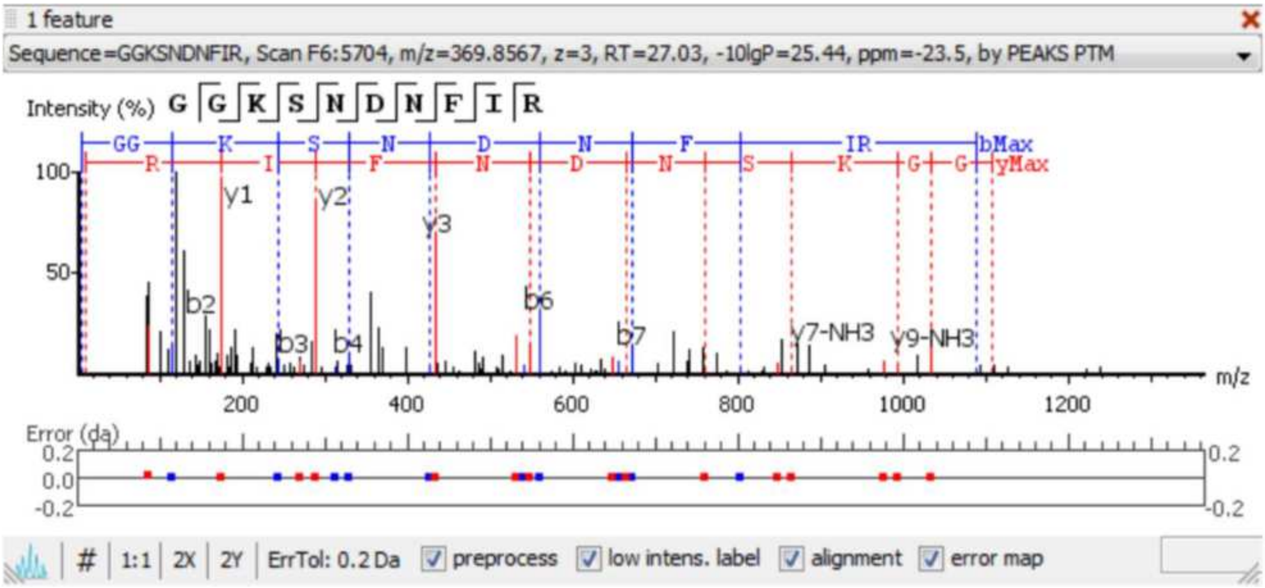

#	b	b-H2O	b-NH3	b (2+)	Seq	y	y-H2O	y-NH3	y (2+)	#
1	58.03	40.02	41.00	29.51	G					10
2	115.05	97.04	98.02	58.03	G	1050.53	1032.52	1033.51	525.77	9
3	243.15	225.14	226.12	122.07	K	993.51	975.50	976.49	497.26	8
4	330.18	312.17	313.15	165.59	S	865.42	847.41	848.39	433.21	7
5	444.22	426.21	427.19	222.61	N	778.38	760.37	761.36	389.69	6
6	559.25	541.24	542.22	280.12	D	664.34	646.33	647.31	332.67	5
7	673.29	655.28	656.26	337.15	N	549.31	531.30	532.29	275.16	4
8	820.36	802.35	803.33	410.68	F	435.27	417.26	418.24	218.14	3
9	933.44	915.43	916.42	467.22	I	288.20	270.19	271.18	144.60	2
10					R	175.12	157.11	158.09	88.04	1

extended FMRFamides-4 (FMRFa-4)

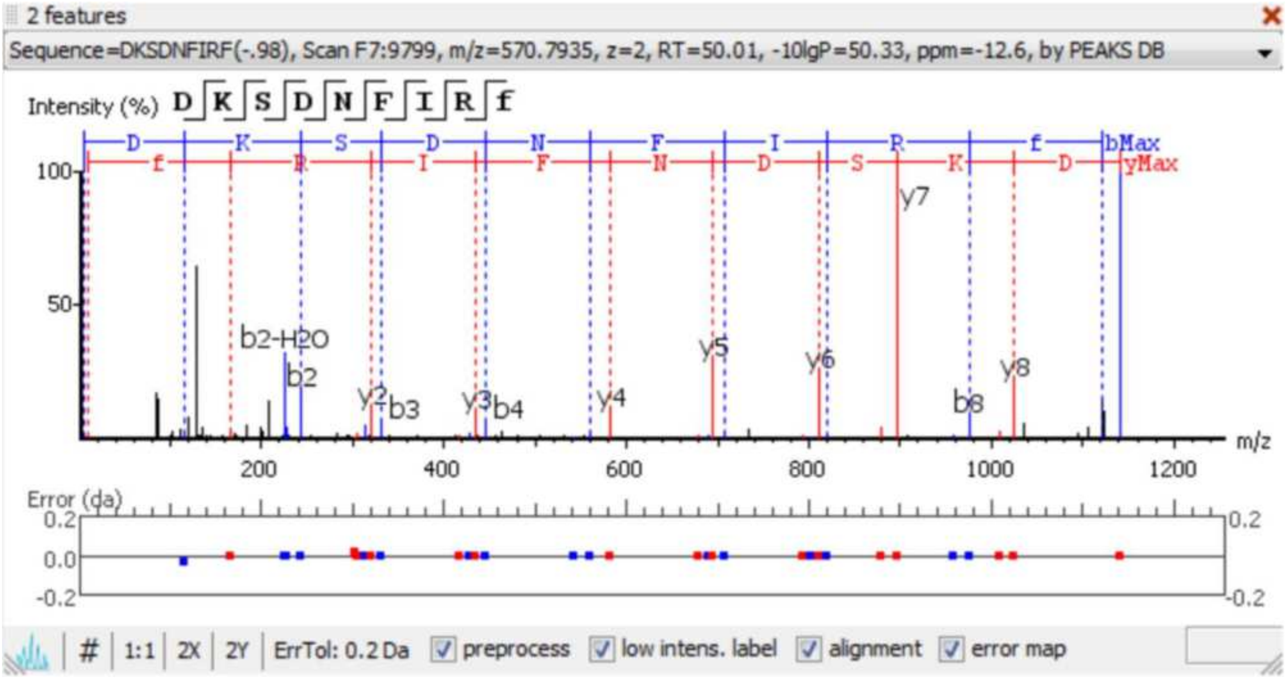

#	b	b-H2O	b-NH3	b (2+)	Seq	y	y-H2O	y-NH3	y (2+)	#
1	116.07	98.02	99.01	58.52	D					9
2	244.13	226.12	227.10	122.56	K	1025.55	1007.54	1008.54	513.28	8
3	331.16	313.15	314.14	166.08	S	897.46	879.45	880.44	449.23	7
4	446.19	428.18	429.16	223.59	D	810.43	792.42	793.40	405.71	6
5	560.23	542.22	543.21	280.62	N	695.40	677.39	678.37	348.20	5
6	707.30	689.29	690.27	354.15	F	581.36	563.35	564.33	291.18	4
7	820.39	802.37	803.36	410.69	I	434.29	416.28	417.26	217.64	3
8	976.49	958.47	959.45	488.74	R	321.20	303.17	304.18	161.10	2
9					F(-.98)	165.10	147.09	148.08	83.05	1

extended FMRFamides-7 (FMRFa-7)

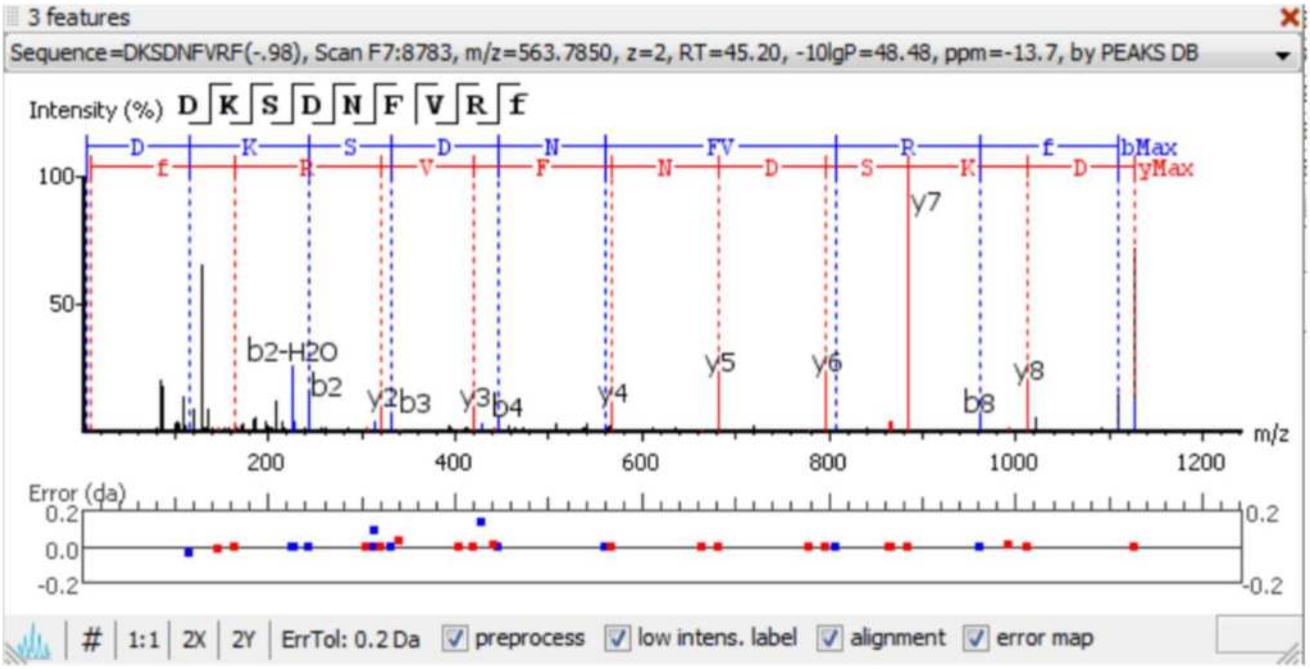

#	b	b-H2O	b-NH3	b (2+)	Seq	y	y-H2O	y-NH3	y (2+)	#
1	116.07	98.02	99.01	58.52	D					9
2	244.13	226.12	227.10	122.56	K	1011.54	993.51	994.51	506.27	8
3	331.16	313.15	314.04	166.08	S	883.44	865.43	866.42	442.20	7
4	446.19	428.04	429.16	223.59	D	796.41	778.40	779.38	398.71	6
5	560.24	542.22	543.20	280.62	N	681.38	663.37	664.36	341.15	5
6	707.30	689.29	690.27	354.15	F	567.34	549.33	550.31	284.17	4
7	806.37	788.36	789.34	403.68	V	420.27	402.26	403.25	210.64	3
8	962.47	944.46	945.44	481.73	R	321.20	303.19	304.18	161.10	2
9					F(-.98)	165.10	147.11	148.08	83.05	1

extended FMRFamides-9 (FMRFa-9)

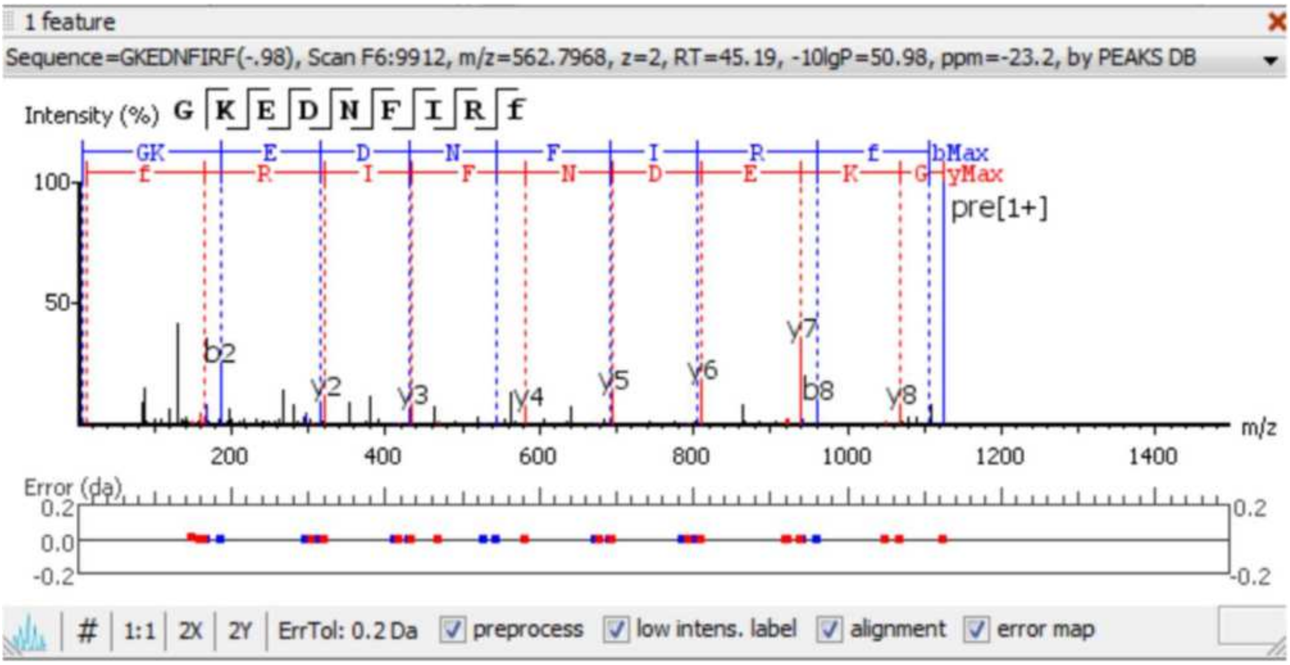

#	b	b-H2O	b-NH3	b (2+)	Seq	y	y-H2O	y-NH3	y (2+)	#
1	58.03	40.02	41.00	29.51	G					9
2	186.12	168.11	169.10	93.56	K	1067.56	1049.55	1050.54	534.28	8
3	315.17	297.16	298.14	158.08	E	939.47	921.46	922.45	470.22	7
4	430.19	412.18	413.17	215.60	D	810.43	792.42	793.40	405.71	6
5	544.24	526.23	527.21	272.62	N	695.40	677.39	678.37	348.20	5
6	691.30	673.29	674.28	346.15	F	581.36	563.35	564.33	291.18	4
7	804.39	786.38	787.36	402.69	I	434.29	416.28	417.26	217.64	3
8	960.49	942.48	943.46	480.75	R	321.20	303.19	304.18	161.09	2
9					F(-.98)	165.10	147.09	148.06	83.05	1

extended FMRFamides-PP-3 (FMRFa-PP-3)

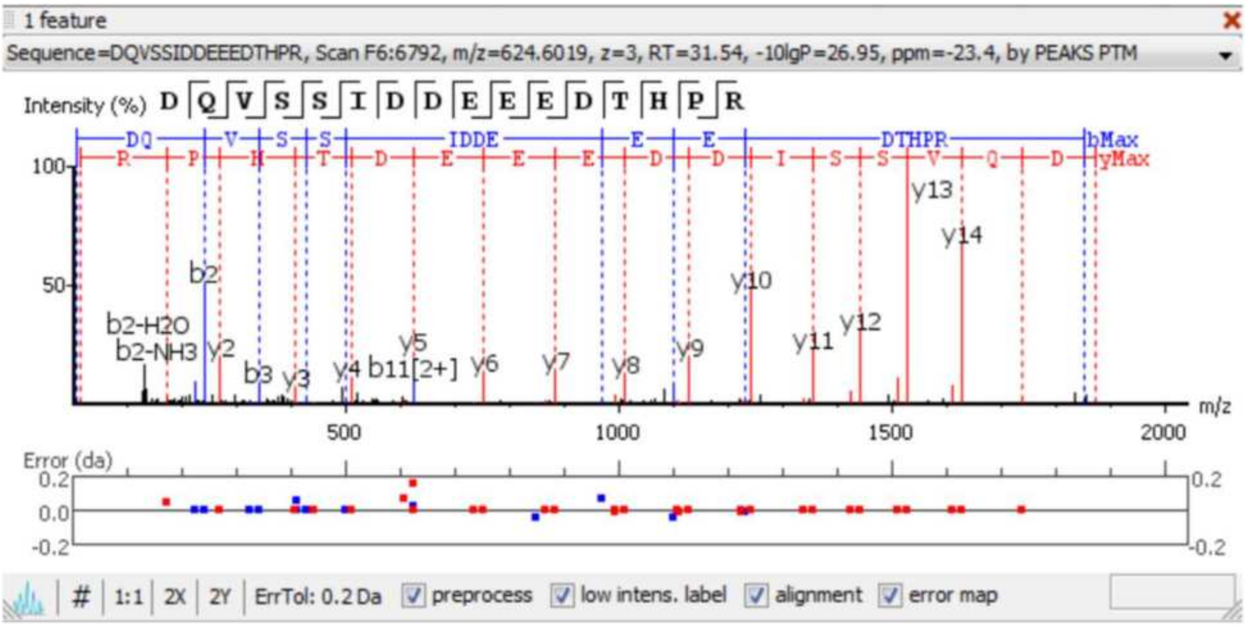

#	b	b-H2O	b-NH3	b (2+)	Seq	y	y-H2O	y-NH3	y (2+)	#
1	116.03	98.02	99.01	58.52	D					16
2	244.09	226.08	227.07	122.55	Q	1756.76	1738.75	1739.73	878.88	15
3	343.16	325.15	326.14	172.08	V	1628.71	1610.70	1611.68	814.85	14
4	430.19	412.12	413.17	215.60	S	1529.64	1511.62	1512.61	765.32	13
5	517.23	499.22	500.20	259.11	S	1442.60	1424.60	1425.58	721.80	12
6	630.31	612.30	613.28	315.65	I	1355.57	1337.56	1338.54	678.29	11
7	745.34	727.33	728.31	373.17	D	1242.49	1224.47	1225.48	621.74	10
8	860.36	842.35	843.34	430.68	D	1127.46	1109.45	1110.45	564.23	9
9	989.41	971.32	972.38	495.20	E	1012.44	994.42	995.42	506.72	8
10	1118.45	1100.44	1101.47	559.72	E	883.39	865.38	866.36	442.20	7
11	1247.49	1229.48	1230.48	624.22	E	754.35	736.34	737.32	377.67	6
12	1362.52	1344.51	1345.49	681.76	D	625.31	607.22	608.28	313.15	5
13	1463.57	1445.56	1446.54	732.28	T	510.28	492.27	493.25	255.64	4
14	1600.62	1582.61	1583.60	800.81	H	409.23	391.22	392.20	205.12	3
15	1697.68	1679.67	1680.65	849.39	P	272.17	254.16	255.14	136.59	2
16					R	175.07	157.11	158.09	88.06	1

extended FMRFamides-11 (FMRFa-11)

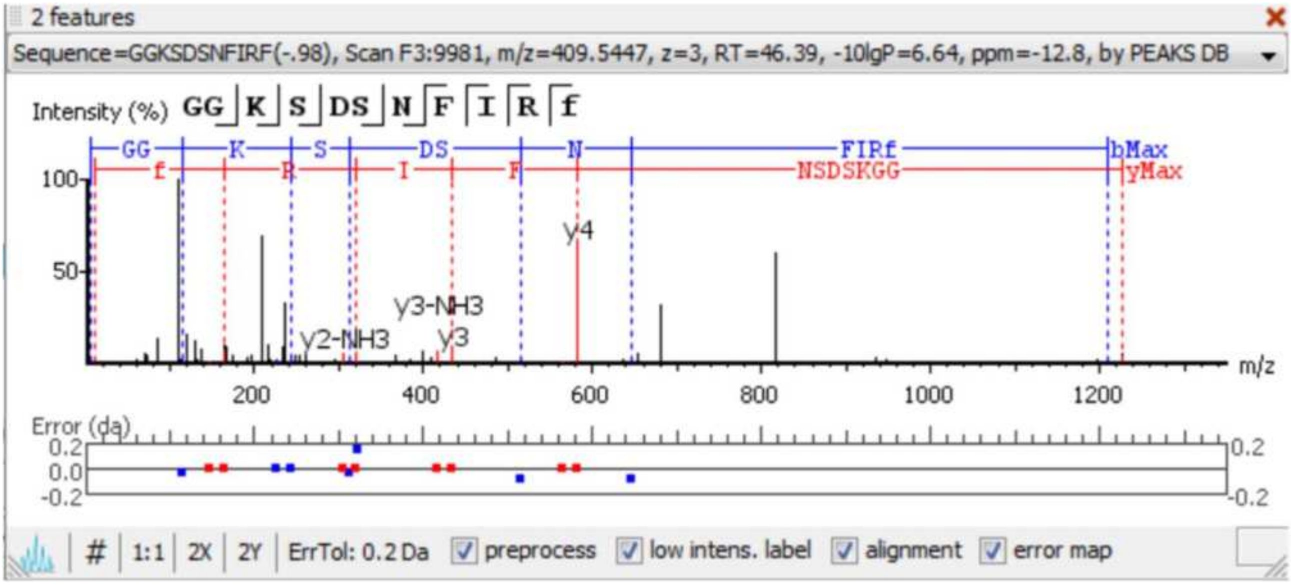

#	b	b-H2O	b-NH3	b (2+)	Seq	y	y-H2O	y-NH3	y (2+)	#
1	58.03	40.02	41.00	29.51	G					11
2	115.09	97.04	98.02	58.03	G	1169.61	1151.60	1152.58	585.30	10
3	243.19	225.14	226.15	122.07	K	1112.58	1094.57	1095.56	556.79	9
4	330.18	312.17	313.15	165.59	S	984.49	966.48	967.46	492.74	8
5	445.20	427.19	428.18	223.10	D	897.46	879.45	880.43	449.23	7
6	532.24	514.23	515.21	266.62	S	782.43	764.42	765.40	391.72	6
7	646.37	628.27	629.25	323.64	N	695.40	677.39	678.37	348.20	5
8	793.35	775.34	776.32	397.17	F	581.36	563.35	564.33	291.18	4
9	906.43	888.42	889.41	453.72	I	434.29	416.28	417.26	217.64	3
10	1062.53	1044.52	1045.51	531.77	R	321.20	303.19	304.18	161.10	2
11					F(-.98)	165.10	147.11	148.08	83.05	1

extended FMRFamides-13$^{1-11}$ (FMRFa-13$^{1-11}$)

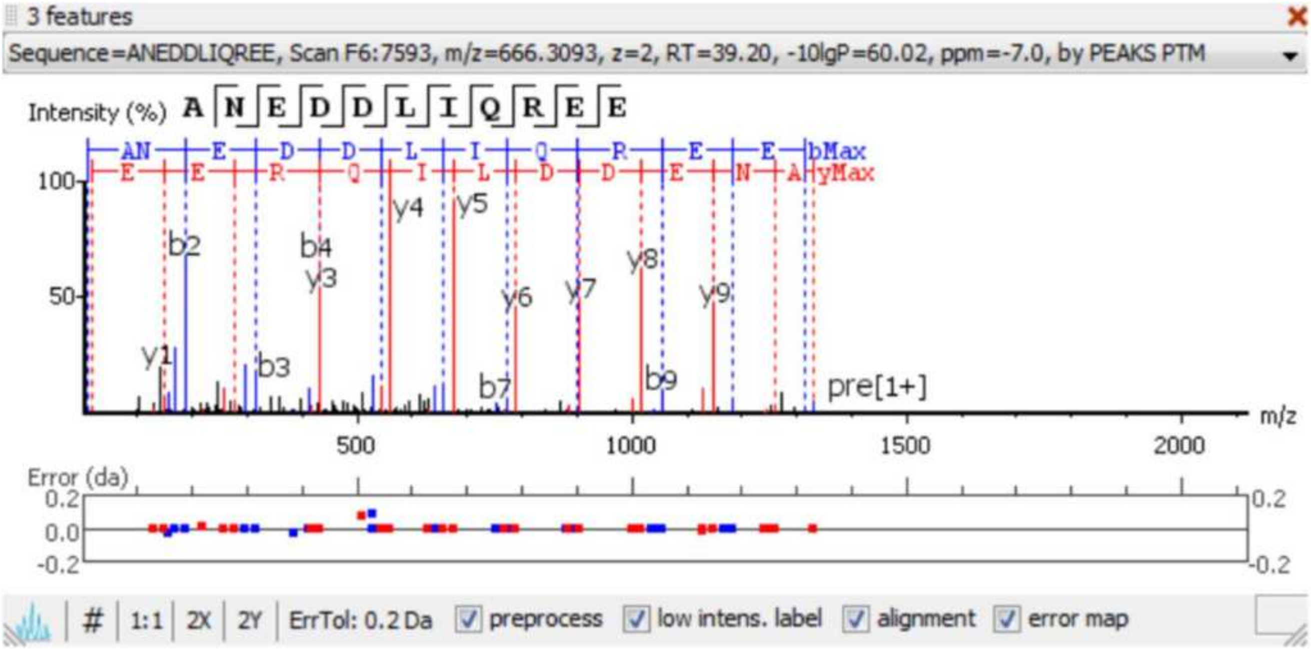

#	b	b-H2O	b-NH3	b (2+)	Seq	y	y-H2O	y-NH3	y (2+)	#
1	72.04	54.03	55.02	36.52	A					11
2	186.09	168.08	169.06	93.54	N	1260.57	1242.55	1243.54	630.79	10
3	315.13	297.12	298.10	158.09	E	1146.53	1128.52	1129.52	573.76	9
4	430.16	412.15	413.13	215.58	D	1017.48	999.47	1000.47	509.16	8
5	545.18	527.17	528.16	273.09	D	902.46	884.45	885.44	451.73	7
6	658.26	640.26	641.24	329.63	L	787.43	769.42	770.41	394.22	6
7	771.35	753.33	754.33	386.21	I	674.35	656.34	657.31	337.67	5
8	899.41	881.40	882.39	450.21	Q	561.26	543.25	544.23	281.13	4
9	1055.51	1037.51	1038.49	528.16	R	433.20	415.19	416.18	217.08	3
10	1184.56	1166.54	1167.53	592.78	E	277.10	259.09	260.08	139.05	2
11					E	148.06	130.05	131.03	74.53	1

extended FMRFamides-14 (FMRFa-14)

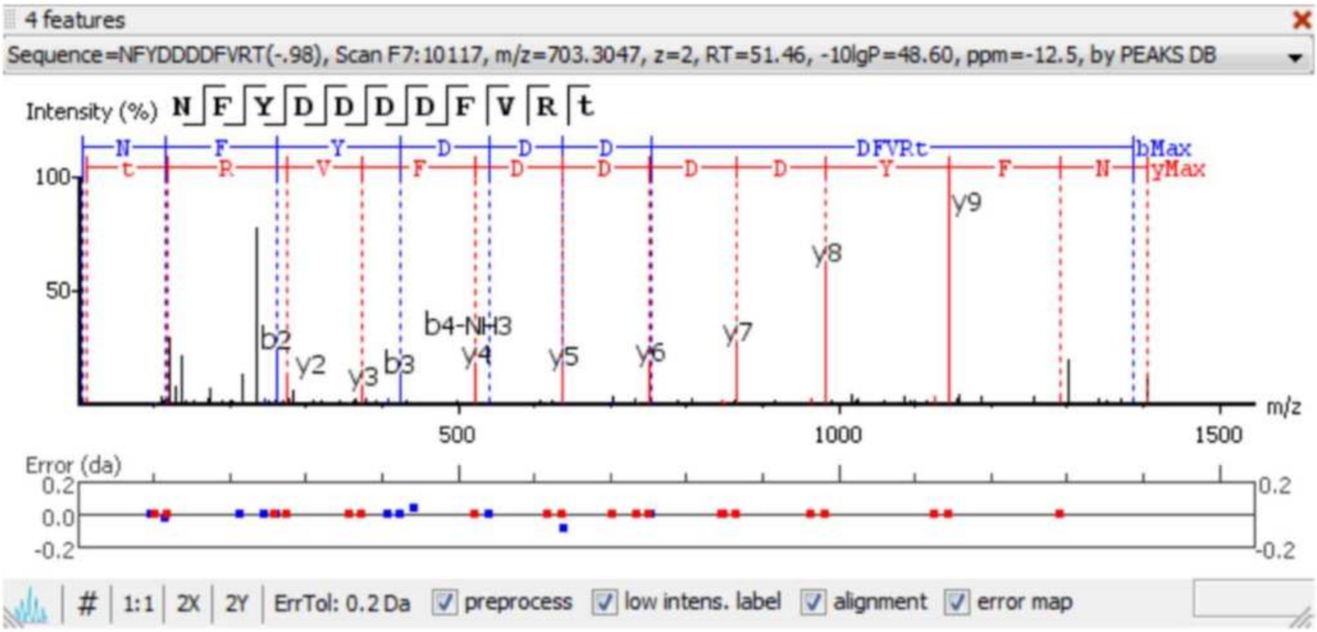

#	b	b-H2O	b-NH3	b (2+)	Seq	y	y-H2O	y-NH3	y (2+)	#
1	115.09	97.04	98.02	58.03	N					11
2	262.12	244.11	245.09	131.56	F	1291.56	1273.55	1274.53	646.28	10
3	425.18	407.17	408.16	213.09	Y	1144.49	1126.49	1127.48	572.75	9
4	540.21	522.20	523.18	270.60	D	981.43	963.42	964.41	491.21	8
5	655.24	637.23	638.30	328.12	D	866.40	848.38	849.38	433.70	7
6	770.26	752.25	753.24	385.63	D	751.37	733.36	734.35	376.19	6
7	885.29	867.28	868.26	443.11	D	636.35	618.34	619.33	318.67	5
8	1032.36	1014.35	1015.33	516.68	F	521.32	503.31	504.29	261.16	4
9	1131.43	1113.42	1114.40	566.21	V	374.25	356.24	357.23	187.63	3
10	1287.53	1269.52	1270.50	644.26	R	275.18	257.17	258.16	138.09	2
11					T(-.98)	119.08	101.07	102.05	60.04	1

extended FMRFamides-16 (FMRFa-16)

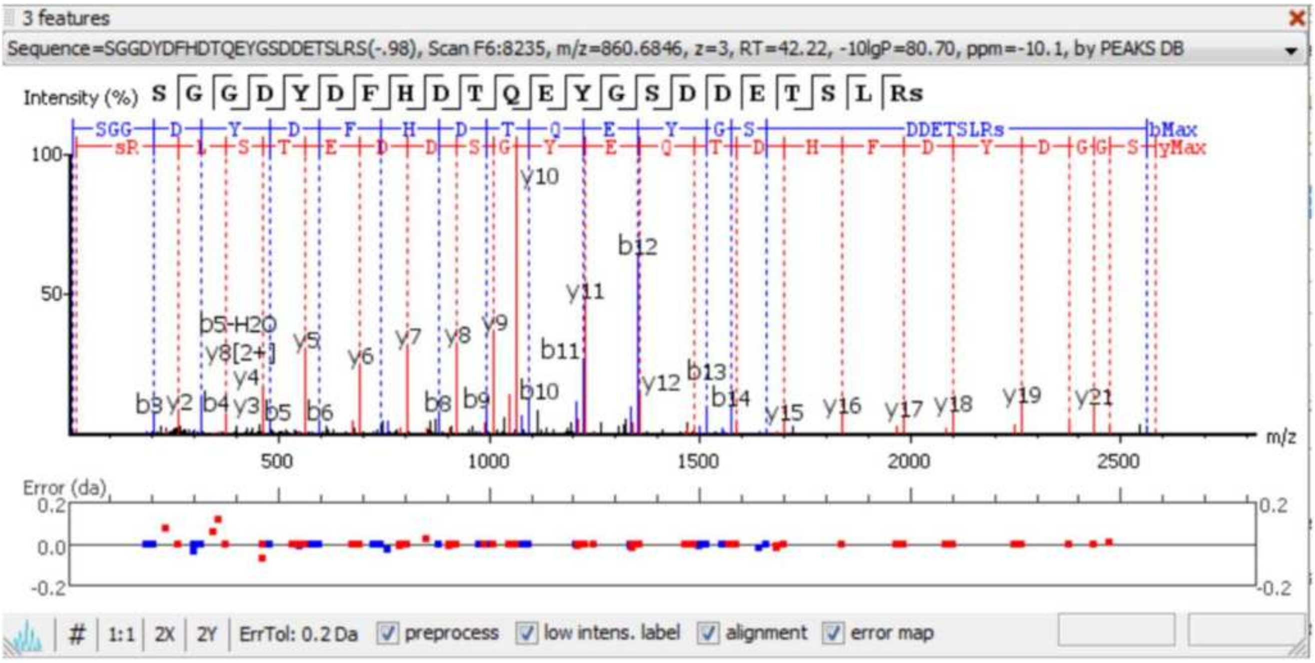

#	b	b-H2O	b-NH3	b (2+)	Seq	y	y-H2O	y-NH3	y (2+)	#
1	88.04	70.03	71.01	44.52	S					23
2	145.06	127.05	128.03	73.03	G	2493.01	2474.98	2475.98	1247.01	22
3	202.08	184.07	185.06	101.54	G	2435.98	2417.98	2418.96	1218.49	21
4	317.11	299.10	300.08	159.05	D	2378.96	2360.95	2361.94	1189.98	20
5	480.17	462.16	463.15	240.59	Y	2263.94	2245.93	2246.91	1132.47	19
6	595.20	577.19	578.17	298.14	D	2100.88	2082.86	2083.85	1050.94	18
7	742.27	724.26	725.24	371.63	F	1985.85	1967.84	1968.82	993.42	17
8	879.32	861.32	862.30	440.16	H	1838.78	1820.77	1821.75	919.89	16
9	994.35	976.34	977.33	497.68	D	1701.72	1683.72	1684.71	851.33	15
10	1095.40	1077.39	1078.37	548.21	T	1586.69	1568.68	1569.67	793.85	14
11	1223.46	1205.45	1206.43	612.23	Q	1485.64	1467.64	1468.62	743.32	13
12	1352.50	1334.49	1335.49	676.75	E	1357.59	1339.58	1340.58	679.29	12
13	1515.57	1497.55	1498.56	758.31	Y	1228.55	1210.54	1211.53	614.77	11
14	1572.59	1554.58	1555.57	786.79	G	1065.48	1047.47	1048.45	533.24	10
15	1659.63	1641.63	1642.59	830.31	S	1008.46	990.45	991.43	504.73	9
16	1774.65	1756.64	1757.62	887.82	D	921.43	903.42	904.41	461.28	8
17	1889.67	1871.66	1872.65	945.34	D	806.40	788.39	789.38	403.70	7
18	2018.72	2000.71	2001.69	1009.86	E	691.37	673.36	674.35	346.13	6
19	2119.76	2101.75	2102.74	1060.39	T	562.33	544.32	545.30	281.67	5
20	2206.80	2188.79	2189.77	1103.90	S	461.28	443.27	444.26	231.06	4
21	2319.88	2301.87	2302.85	1160.44	L	374.25	356.24	357.10	187.63	3
22	2475.98	2457.97	2458.95	1238.49	R	261.17	243.16	244.14	131.08	2
23					S(-.98)	105.07	87.06	88.04	53.03	1

extended FMRFamides-17 (FMRFa-17)

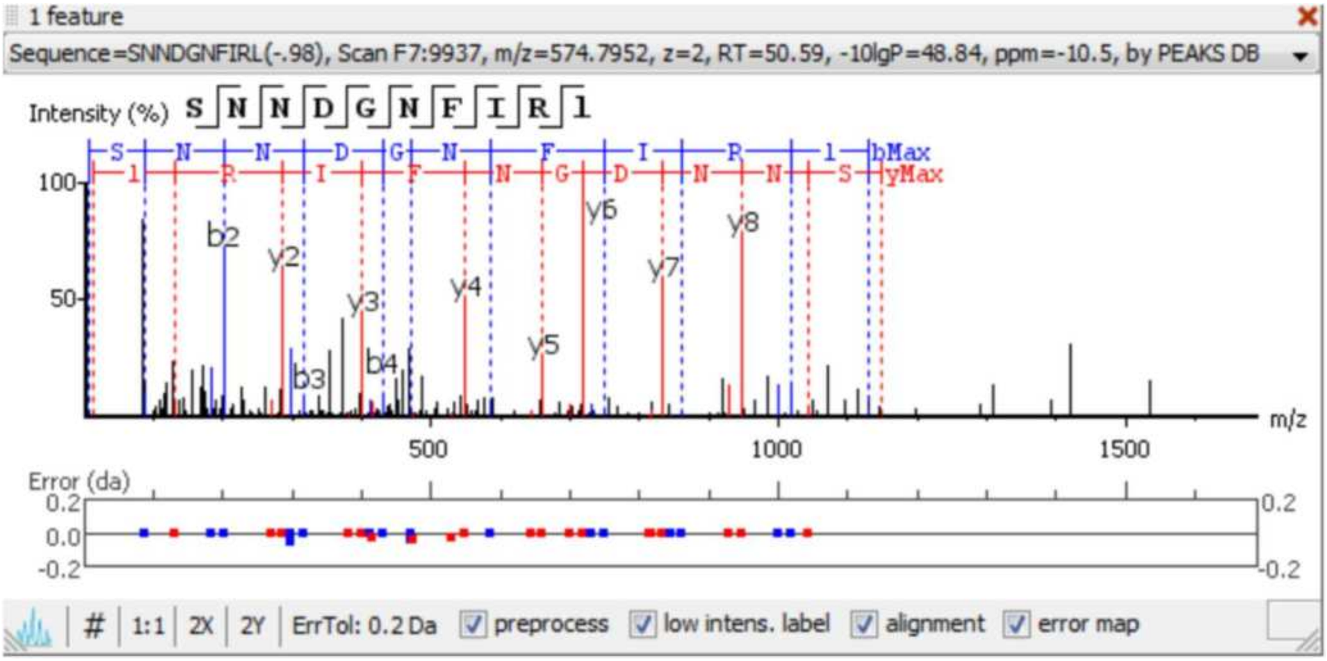

#	b	b-H2O	b-NH3	b (2+)	Seq	y	y-H2O	y-NH3	y (2+)	#
1	88.04	70.03	71.01	44.52	S					10
2	202.08	184.07	185.06	101.54	N	1061.55	1043.54	1044.52	531.31	9
3	316.13	298.18	299.10	158.56	N	947.51	929.49	930.48	474.29	8
4	431.15	413.14	414.13	216.08	D	833.46	815.45	816.43	417.26	7
5	488.17	470.20	471.15	244.59	G	718.44	700.43	701.41	359.72	6
6	602.22	584.21	585.19	301.61	N	661.42	643.40	644.39	331.21	5
7	749.28	731.27	732.26	375.14	F	547.37	529.36	530.34	274.19	4
8	862.37	844.36	845.34	431.68	I	400.30	382.29	383.28	200.65	3
9	1018.47	1000.46	1001.44	509.74	R	287.22	269.21	270.19	144.11	2
10					L(-.98)	131.12	113.11	114.09	66.06	1

extended FMRFamides-18 (FMRFa-18)

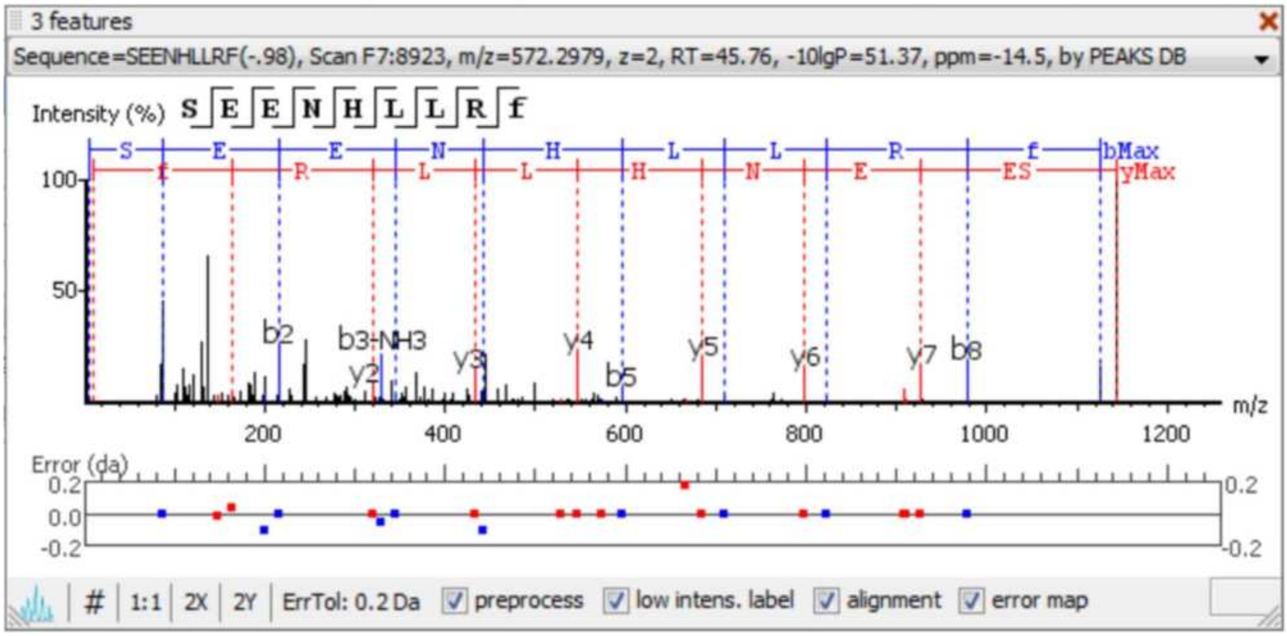

#	b	b-H2O	b-NH3	b (2+)	Seq	y	y-H2O	y-NH3	y (2+)	#
1	88.04	70.03	71.01	44.52	S					9
2	217.08	199.18	200.06	109.04	E	1056.56	1038.55	1039.53	528.79	8
3	346.13	328.11	329.15	173.56	E	927.52	909.51	910.49	464.26	7
4	460.17	442.16	443.25	230.58	N	798.47	780.46	781.45	399.74	6
5	597.23	579.22	580.20	299.11	H	684.43	666.23	667.40	342.72	5
6	710.32	692.30	693.28	355.66	L	547.37	529.36	530.34	274.19	4
7	823.40	805.38	806.37	412.20	L	434.29	416.28	417.26	217.64	3
8	979.50	961.49	962.47	490.25	R	321.20	303.19	304.18	161.10	2
9					F(-.98)	165.05	147.11	148.08	83.05	1

extended FMRFamides-PP-4 (FMRFa-PP-4)

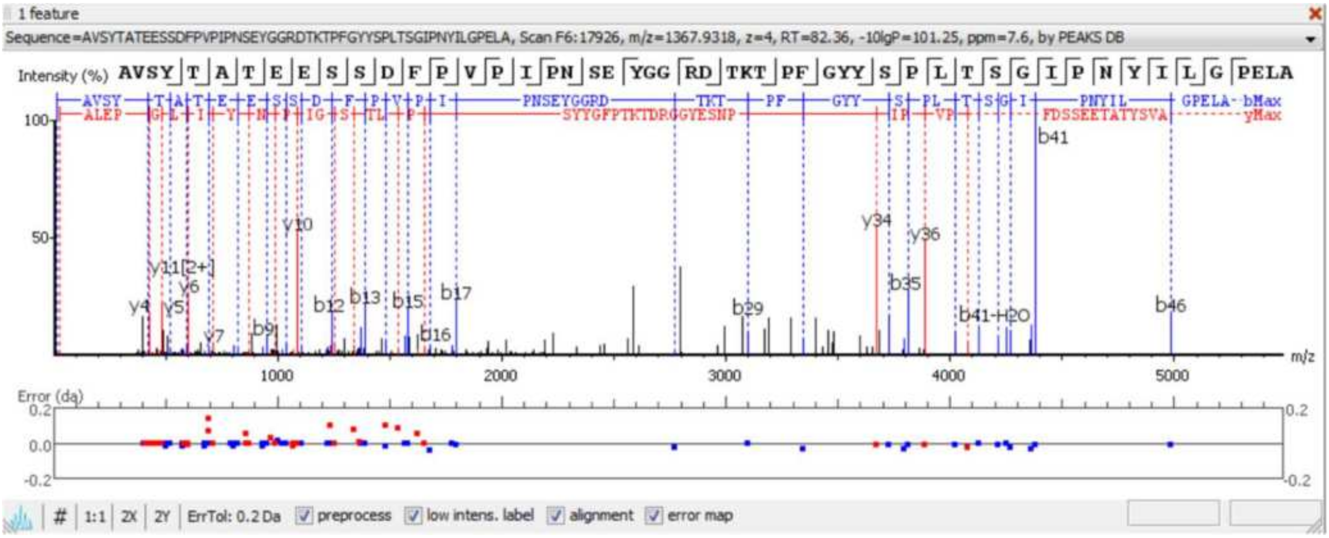

#	b	b-H2O	b-NH3	b (2+)	Seq	y	y-H2O	y-NH3	y (2+)	#
1	72.04	54.03	55.02	36.52	A					51
2	171.11	153.10	154.09	86.06	V	5397.60	5379.59	5380.57	2699.30	50
3	258.15	240.13	241.12	129.57	S	5298.53	5280.52	5281.50	2649.77	49
4	421.21	403.20	404.18	211.10	Y	5211.50	5193.49	5194.47	2606.25	48
5	522.26	504.24	505.25	261.63	T	5048.44	5030.43	5031.41	2524.72	47
6	593.29	575.28	576.28	297.15	A	4947.39	4929.38	4930.36	2474.19	46
7	694.34	676.33	677.33	347.67	T	4876.35	4858.34	4859.32	2438.68	45
8	823.38	805.37	806.38	412.19	E	4775.30	4757.29	4758.28	2388.15	44
9	952.43	934.41	935.42	476.71	E	4646.26	4628.25	4629.23	2323.63	43
10	1039.46	1021.44	1022.43	520.23	S	4517.22	4499.21	4500.19	2259.11	42
11	1126.49	1108.48	1109.46	563.75	S	4430.19	4412.18	4413.16	2215.59	41
12	1241.52	1223.51	1224.49	621.26	D	4343.15	4325.14	4326.13	2172.08	40
13	1388.59	1370.57	1371.56	694.80	F	4228.13	4210.12	4211.10	2114.56	39
14	1485.66	1467.63	1468.61	743.32	P	4081.09	4063.05	4064.03	2041.03	38
15	1584.70	1566.70	1567.68	792.85	V	3984.01	3966.00	3966.98	1992.50	37
16	1681.80	1663.75	1664.73	841.38	P	3884.95	3866.93	3867.91	1942.97	36
17	1794.85	1776.84	1777.82	897.92	I	3787.89	3769.87	3770.86	1894.44	35
18	1891.90	1873.89	1874.87	946.45	P	3674.81	3656.79	3657.77	1837.90	34
19	2005.94	1987.93	1988.91	1003.45	N	3577.75	3559.74	3560.72	1789.37	33
20	2092.97	2074.96	2075.94	1046.99	S	3463.71	3445.70	3446.68	1732.35	32
21	2222.01	2204.00	2204.99	1111.51	E	3376.67	3358.66	3359.65	1688.84	31
22	2385.08	2367.07	2368.05	1193.04	Y	3247.63	3229.62	3230.60	1624.26	30
23	2442.10	2424.09	2425.07	1221.55	G	3084.57	3066.56	3067.54	1542.78	29
24	2499.12	2481.11	2482.09	1250.06	G	3027.55	3009.54	3010.52	1514.27	28
25	2655.22	2637.21	2638.19	1328.11	R	2970.52	2952.51	2953.50	1485.66	27
26	2770.27	2752.24	2753.22	1385.62	D	2814.42	2796.41	2797.40	1407.71	26
27	2871.30	2853.29	2854.27	1436.15	T	2699.40	2681.39	2682.37	1350.20	25
28	2999.39	2981.38	2982.36	1500.20	K	2598.35	2580.34	2581.32	1299.67	24
29	3100.44	3082.43	3083.41	1550.72	T	2470.25	2452.24	2453.23	1235.63	23
30	3197.49	3179.48	3180.46	1599.25	P	2369.21	2351.20	2352.18	1185.10	22
31	3344.59	3326.55	3327.53	1672.78	F	2272.15	2254.14	2255.13	1136.58	21
32	3401.58	3383.57	3384.55	1701.29	G	2125.09	2107.07	2108.06	1063.04	20
33	3564.64	3546.63	3547.62	1782.82	Y	2068.06	2050.05	2051.04	1034.53	19
34	3727.72	3709.70	3710.68	1864.35	Y	1905.00	1886.99	1887.97	953.00	18
35	3814.75	3796.73	3797.75	1907.87	S	1741.94	1723.93	1724.91	871.47	17
36	3911.79	3893.78	3894.77	1956.40	P	1654.91	1636.89	1637.88	827.95	16
37	4024.89	4006.87	4007.85	2012.94	L	1557.85	1539.84	1540.73	779.43	15
38	4125.92	4107.91	4108.90	2063.46	T	1444.77	1426.76	1427.74	722.88	14
39	4212.97	4194.95	4195.93	2106.98	S	1343.64	1325.71	1326.69	672.36	13
40	4270.00	4251.97	4252.95	2135.49	G	1256.69	1238.57	1239.66	628.84	12
41	4383.08	4365.08	4366.04	2192.03	I	1199.67	1181.66	1182.64	600.34	11
42	4480.11	4462.10	4463.09	2240.56	P	1086.58	1068.57	1069.57	543.79	10
43	4594.16	4576.15	4577.13	2297.58	N	989.53	971.52	972.47	495.27	9
44	4757.22	4739.21	4740.19	2379.11	Y	875.49	857.47	858.40	438.24	8
45	4870.31	4852.29	4853.28	2435.65	I	712.43	694.34	695.25	356.71	7
46	4983.40	4965.38	4966.36	2492.19	L	599.34	581.33	582.31	300.17	6
47	5040.41	5022.40	5023.38	2520.71	G	486.26	468.24	469.23	243.63	5
48	5137.46	5119.45	5120.44	2569.23	P	429.24	411.22	412.21	215.12	4
49	5266.51	5248.50	5249.48	2633.75	E	332.18	314.17	315.15	166.59	3
50	5379.59	5361.58	5362.56	2690.29	L	203.14	185.13	186.11	102.07	2
51					A	90.05	72.04	73.03	45.53	1

Pea FERLQ-like

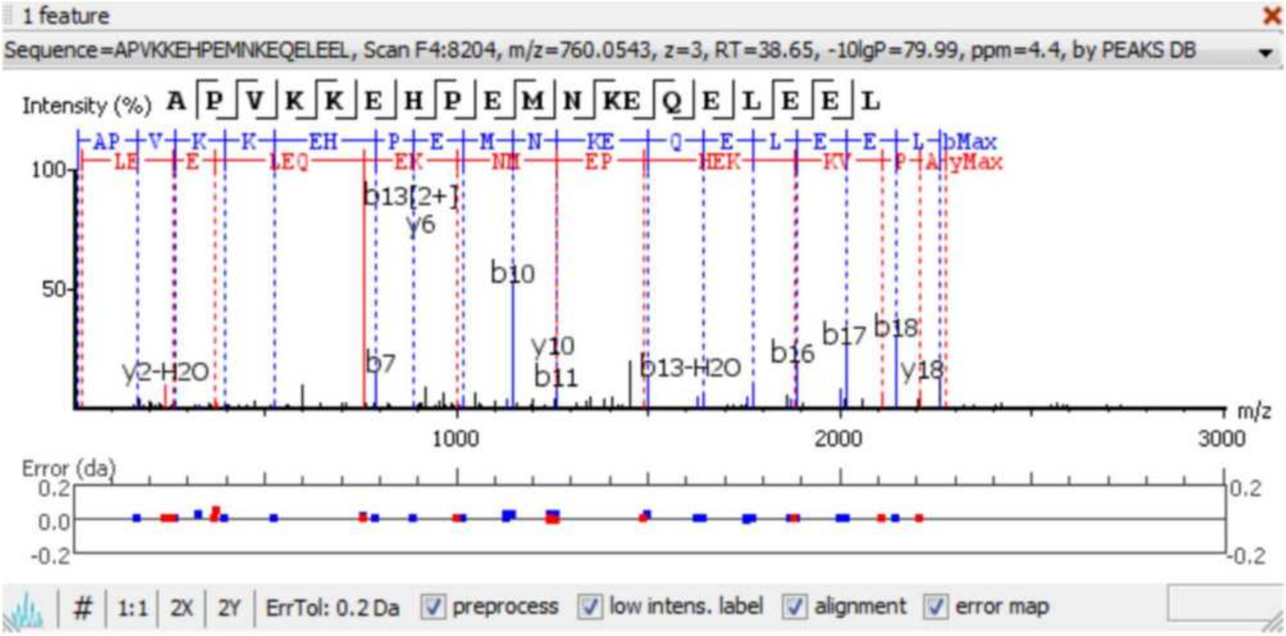

#	b	b-H2O	b-NH3	b (2+)	Seq	y	y-H2O	y-NH3	y (2+)	#
1	72.04	54.03	55.02	36.52	A					19
2	169.10	151.09	152.07	85.05	P	2207.11	2189.09	2190.07	1104.05	18
3	268.17	250.16	251.14	134.58	V	2110.04	2092.04	2093.02	1055.52	17
4	396.26	378.25	379.23	198.63	K	2010.98	1992.97	1993.95	1005.99	16
5	524.36	506.35	507.33	262.68	K	1882.89	1864.87	1865.86	941.94	15
6	653.40	635.39	636.37	327.17	E	1754.79	1736.78	1737.76	877.90	14
7	790.46	772.45	773.43	395.73	H	1625.75	1607.74	1608.72	813.37	13
8	887.51	869.50	870.48	444.26	P	1488.69	1470.68	1471.66	744.84	12
9	1016.55	998.54	999.53	508.78	E	1391.64	1373.63	1374.61	696.32	11
10	1147.56	1129.55	1130.56	574.30	M	1262.61	1244.58	1245.58	631.80	10
11	1261.60	1243.63	1244.58	631.32	N	1131.55	1113.54	1114.53	566.28	9
12	1389.73	1371.72	1372.70	695.37	K	1017.51	999.50	1000.48	509.25	8
13	1518.77	1500.74	1501.75	759.87	E	889.41	871.40	872.39	445.21	7
14	1646.83	1628.82	1629.81	823.92	Q	760.38	742.36	743.35	380.69	6
15	1775.88	1757.86	1758.87	888.44	E	632.31	614.30	615.29	316.66	5
16	1888.96	1870.94	1871.93	944.98	L	503.27	485.26	486.24	252.14	4
17	2018.00	1999.99	2000.97	1009.51	E	390.19	372.18	373.10	195.59	3
18	2147.04	2129.03	2130.02	1074.02	E	261.14	243.13	244.12	131.07	2
19					L	132.10	114.09	115.07	66.55	1

Fliktin-1 (Flik-1)

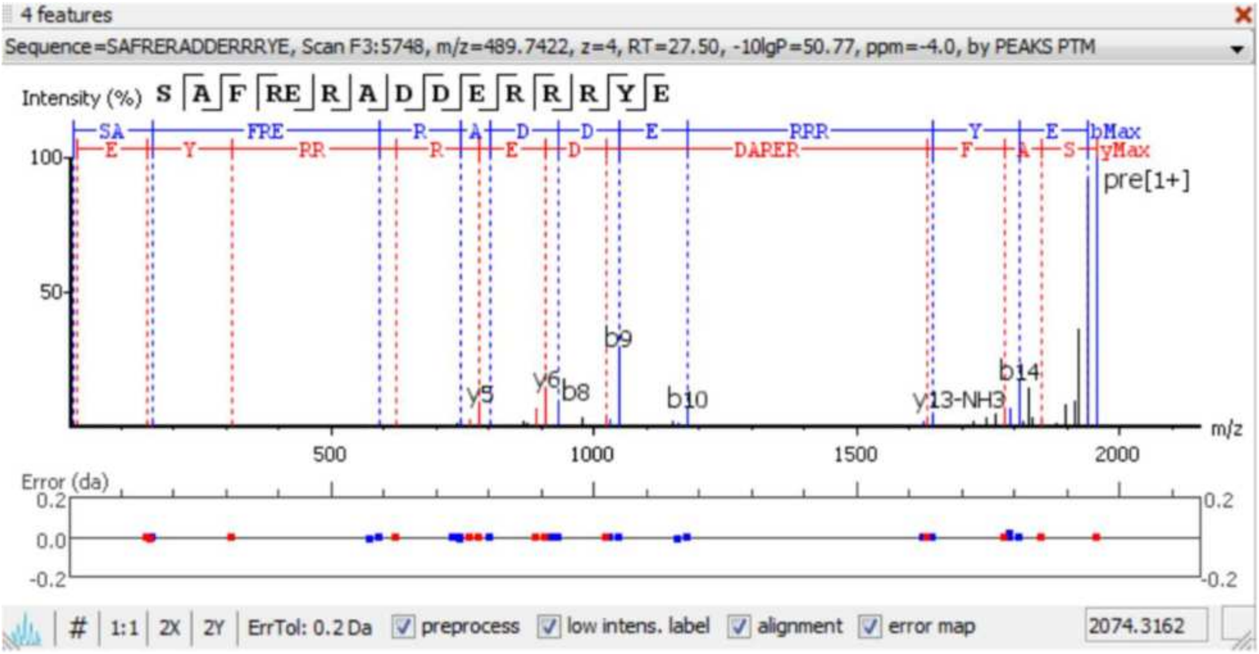

#	b	b-H2O	b-NH3	b (2+)	Seq	y	y-H2O	y-NH3	y (2+)	#
1	88.04	70.03	71.01	44.52	S					15
2	159.08	141.07	142.05	80.04	A	1868.91	1850.90	1851.89	934.96	14
3	306.15	288.13	289.12	153.57	F	1797.87	1779.86	1780.85	899.44	13
4	462.25	444.24	445.22	231.62	R	1650.81	1632.79	1633.78	825.90	12
5	591.29	573.29	574.26	296.14	E	1494.70	1476.69	1477.68	747.85	11
6	747.39	729.38	730.37	374.21	R	1365.66	1347.65	1348.63	683.33	10
7	818.43	800.37	801.40	409.71	A	1209.56	1191.55	1192.53	605.28	9
8	933.46	915.44	916.43	467.23	D	1138.52	1120.51	1121.50	569.76	8
9	1048.48	1030.47	1031.45	524.74	D	1023.50	1005.49	1006.47	512.25	7
10	1177.52	1159.53	1160.50	589.26	E	908.47	890.46	891.42	454.73	6
11	1333.62	1315.61	1316.60	667.31	R	779.36	761.42	762.40	390.21	5
12	1489.73	1471.72	1472.70	745.37	R	623.33	605.32	606.30	312.16	4
13	1645.82	1627.81	1628.80	823.41	R	467.22	449.21	450.20	234.11	3
14	1808.89	1790.88	1791.86	904.95	Y	311.12	293.11	294.10	156.06	2
15					E	148.06	130.05	131.03	74.53	1

Fliktin-2 (Flik-2)

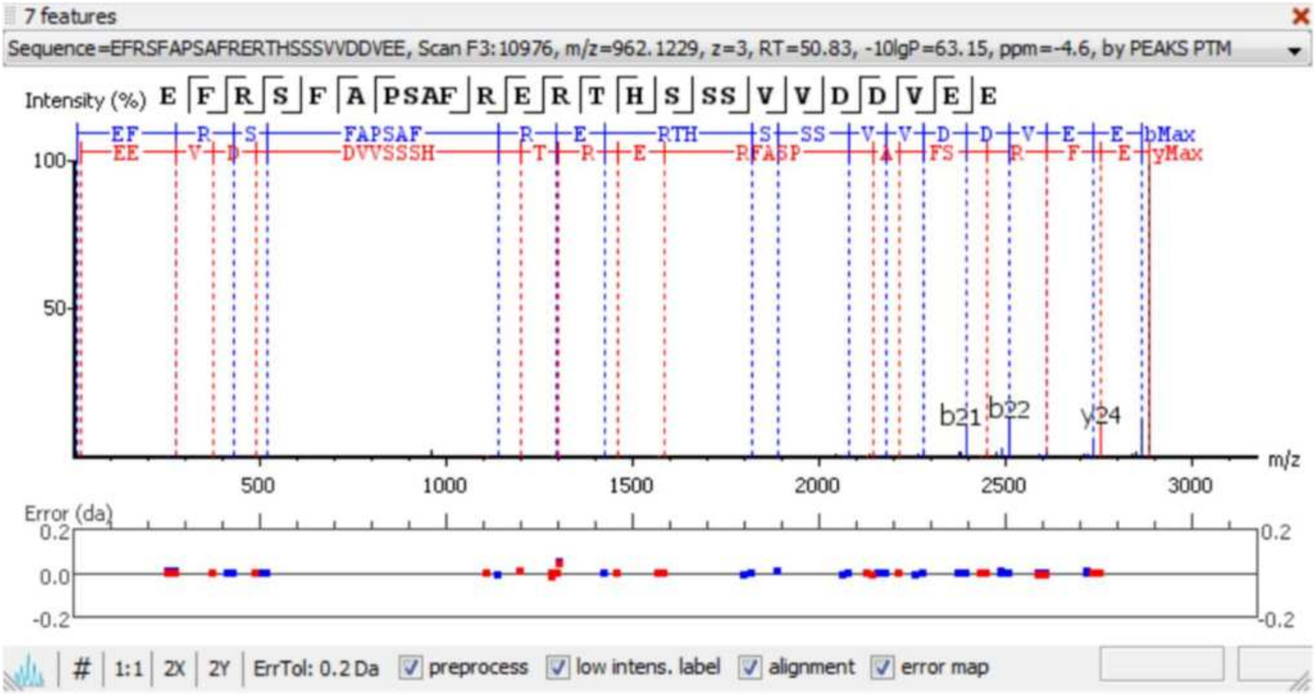

#	b	b-H2O	b-NH3	b (2+)	Seq	y	y-H2O	y-NH3	y (2+)	#
1	130.05	112.04	113.02	65.53	E					25
2	277.10	259.09	260.09	139.06	F	2755.31	2737.30	2738.28	1378.15	24
3	433.22	415.21	416.19	217.11	R	2608.25	2590.23	2591.22	1304.57	23
4	520.25	502.24	503.22	260.63	S	2452.13	2434.13	2435.11	1226.57	22
5	667.32	649.31	650.29	334.16	F	2365.11	2347.09	2348.08	1183.05	21
6	738.36	720.35	721.33	369.68	A	2218.04	2200.03	2201.01	1109.53	20
7	835.41	817.40	818.38	418.21	P	2147.01	2128.99	2129.97	1074.00	19
8	922.44	904.43	905.42	461.72	S	2049.95	2031.94	2032.92	1025.47	18
9	993.48	975.47	976.45	497.24	A	1962.92	1944.90	1945.89	981.96	17
10	1140.56	1122.54	1123.52	570.77	F	1891.88	1873.87	1874.85	946.44	16
11	1296.65	1278.64	1279.62	648.82	R	1744.81	1726.80	1727.78	872.90	15
12	1425.69	1407.68	1408.66	713.35	E	1588.71	1570.70	1571.68	794.85	14
13	1581.79	1563.78	1564.77	791.40	R	1459.67	1441.66	1442.64	730.33	13
14	1682.84	1664.83	1665.81	841.92	T	1303.56	1285.55	1286.56	652.28	12
15	1819.90	1801.89	1802.88	910.45	H	1202.51	1184.51	1185.49	601.76	11
16	1906.93	1888.91	1889.90	953.97	S	1065.46	1047.45	1048.43	533.23	10
17	1993.96	1975.95	1976.94	997.48	S	978.43	960.42	961.40	489.71	9
18	2080.99	2062.98	2063.98	1041.00	S	891.39	873.38	874.37	446.20	8
19	2180.06	2162.05	2163.04	1090.53	V	804.36	786.35	787.34	402.68	7
20	2279.13	2261.12	2262.12	1140.07	V	705.29	687.28	688.27	353.15	6
21	2394.16	2376.14	2377.13	1197.58	D	606.23	588.21	589.20	303.61	5
22	2509.19	2491.16	2492.16	1255.09	D	491.20	473.19	474.17	246.10	4
23	2608.25	2590.24	2591.22	1304.57	V	376.17	358.16	359.14	188.59	3
24	2737.30	2719.28	2720.27	1369.15	E	277.10	259.09	260.08	139.05	2
25					E	148.06	130.05	131.03	74.53	1

Fliktin-3 (Flik-3)

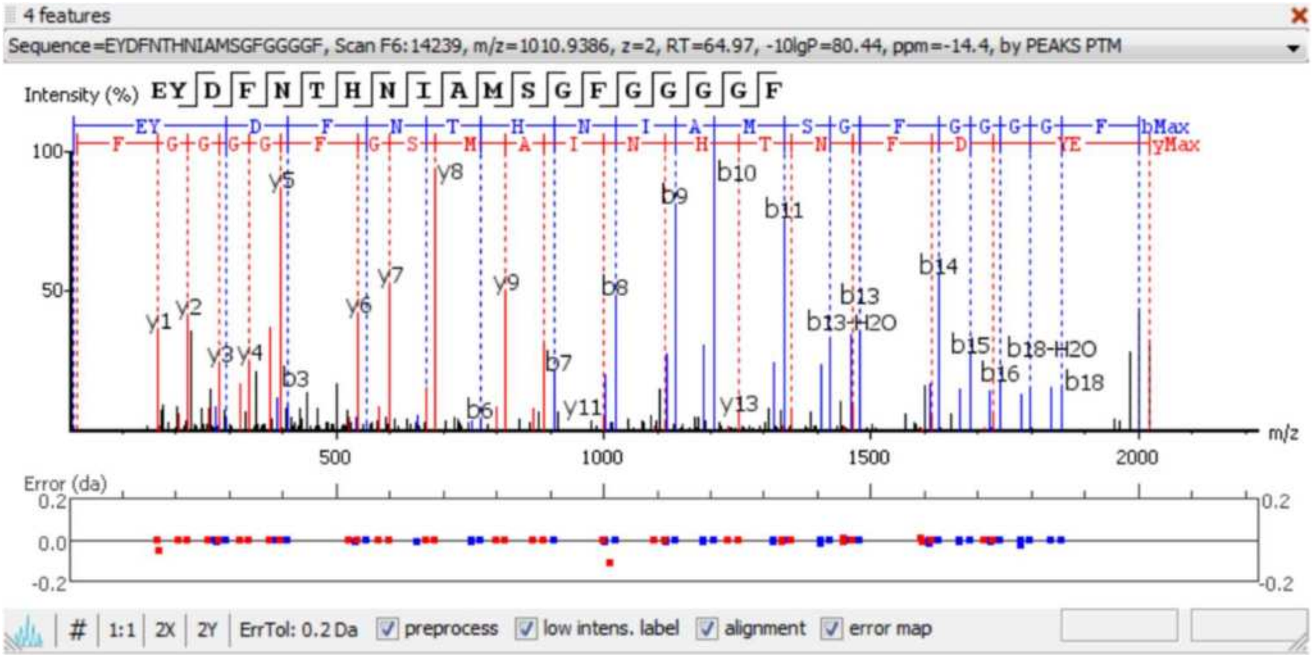

#	b	b-H2O	b-NH3	b (2+)	Seq	y	y-H2O	y-NH3	y (2+)	#
1	130.05	112.04	113.02	65.53	E					19
2	293.11	275.10	276.10	147.06	Y	1891.81	1873.80	1874.78	946.40	18
3	408.14	390.13	391.11	204.57	D	1728.75	1710.73	1711.72	864.87	17
4	555.21	537.21	538.18	278.10	F	1613.72	1595.70	1596.70	807.36	16
5	669.25	651.26	652.23	335.13	N	1466.65	1448.63	1449.63	733.82	15
6	770.29	752.29	753.28	385.65	T	1352.61	1334.60	1335.59	676.80	14
7	907.36	889.35	890.33	454.18	H	1251.56	1233.55	1234.53	626.28	13
8	1021.40	1003.39	1004.39	511.20	N	1114.50	1096.49	1097.47	557.75	12
9	1134.49	1116.48	1117.47	567.74	I	1000.46	982.45	983.43	500.73	11
10	1205.52	1187.51	1188.51	603.26	A	887.37	869.36	870.34	444.19	10
11	1336.56	1318.55	1319.55	668.79	M	816.34	798.33	799.31	408.67	9
12	1423.60	1405.59	1406.59	712.30	S	685.29	667.28	668.27	343.15	8
13	1480.62	1462.61	1463.59	740.81	G	598.26	580.25	581.23	299.63	7
14	1627.69	1609.67	1610.68	814.34	F	541.24	523.23	524.21	271.12	6
15	1684.71	1666.69	1667.70	842.85	G	394.17	376.16	377.15	197.59	5
16	1741.73	1723.71	1724.72	871.36	G	337.15	319.14	320.12	169.13	4
17	1798.74	1780.75	1781.75	899.87	G	280.13	262.12	263.10	140.56	3
18	1855.77	1837.76	1838.74	928.39	G	223.11	205.10	206.08	112.05	2
19					F	166.09	148.08	149.06	83.54	1

Fliktin-4 (Flik-4)

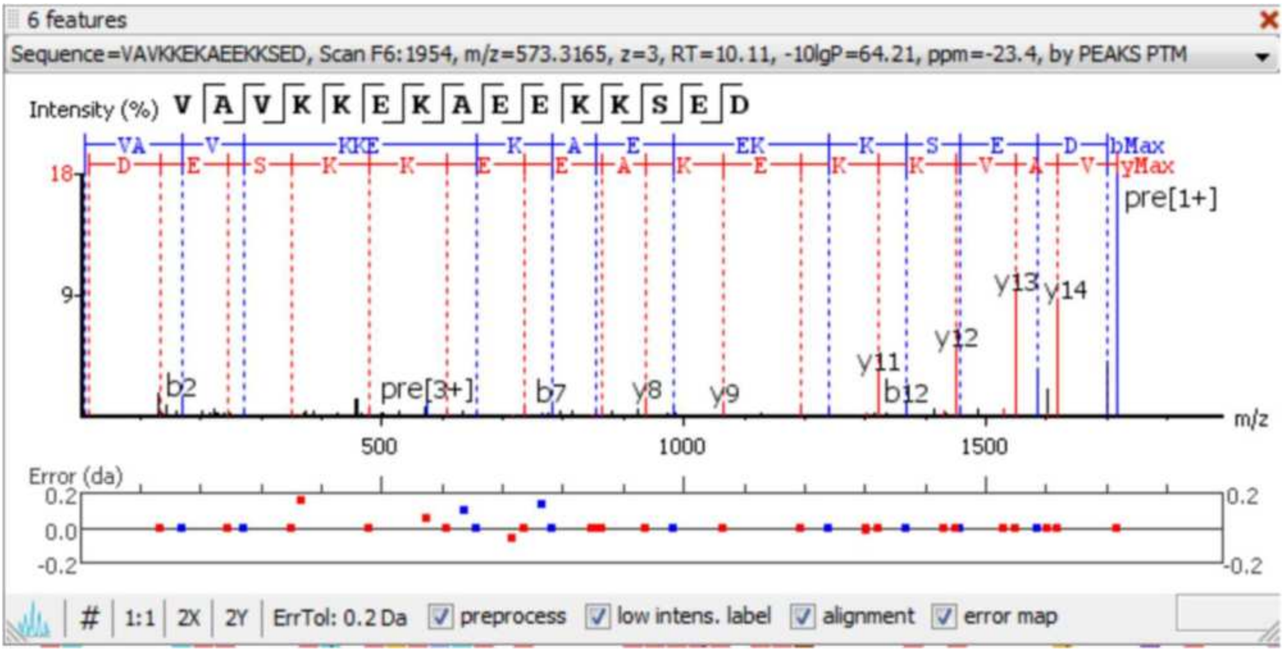

#	b	b-H2O	b-NH3	b (2+)	Seq	y	y-H2O	y-NH3	y (2+)	#
1	100.08	82.07	83.05	50.54	V					15
2	171.11	153.10	154.09	86.06	A	1618.87	1600.85	1601.84	809.93	14
3	270.18	252.17	253.15	135.59	V	1547.83	1529.82	1530.80	774.41	13
4	398.28	380.27	381.25	199.64	K	1448.76	1430.74	1431.73	724.88	12
5	526.37	508.36	509.34	263.69	K	1320.66	1302.65	1303.66	660.83	11
6	655.41	637.40	638.28	328.21	E	1192.57	1174.56	1175.54	596.78	10
7	783.51	765.50	766.34	392.25	K	1063.53	1045.52	1046.50	532.26	9
8	854.55	836.54	837.52	427.77	A	935.43	917.42	918.40	468.22	8
9	983.59	965.58	966.56	492.29	E	864.40	846.39	847.37	432.70	7
10	1112.63	1094.62	1095.60	556.82	E	735.35	717.41	718.32	368.01	6
11	1240.73	1222.72	1223.70	620.86	K	606.31	588.30	589.28	303.65	5
12	1368.82	1350.81	1351.79	684.91	K	478.22	460.20	461.19	239.61	4
13	1455.85	1437.84	1438.83	728.43	S	350.12	332.11	333.09	175.56	3
14	1584.89	1566.89	1567.87	792.95	E	263.09	245.08	246.06	132.04	2
15					D	134.05	116.03	117.02	67.52	1

Hansolin

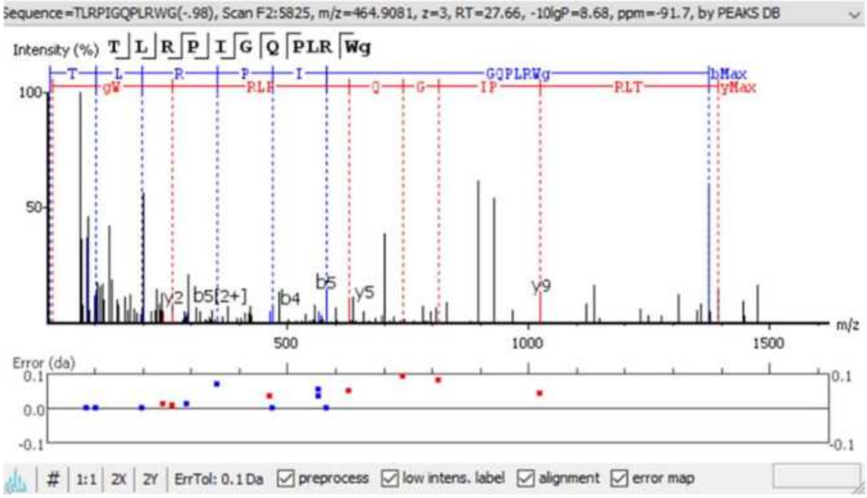

#	b	b-H2O	b-NH3	b (2+)	Seq	y	y-H2O	y-NH3	y (2+)	#
1	102.06	84.04	85.03	51.53	T					12
2	215.14	197.13	198.11	108.07	L	1291.77	1273.76	1274.75	646.39	11
3	371.24	353.16	354.14	186.12	R	1178.69	1160.68	1161.66	589.85	10
4	468.29	450.28	451.27	234.65	P	1022.54	1004.58	1005.56	511.79	9
5	581.38	563.33	564.29	291.18	I	925.54	907.53	908.51	463.27	8
6	638.40	620.39	621.37	319.70	G	812.37	794.44	795.43	406.73	7
7	766.46	748.45	749.43	383.73	Q	755.43	737.42	738.31	378.22	6
8	863.51	845.50	846.48	432.26	P	627.32	609.36	610.35	314.19	5
9	976.59	958.58	959.57	488.80	L	530.32	512.31	513.29	265.66	4
10	1132.70	1114.68	1115.67	566.85	R	417.24	399.23	400.21	209.12	3
11	1318.77	1300.76	1301.75	659.89	W	261.12	243.11	244.11	131.07	2
12					G(-.98)	75.06	57.04	58.03	38.03	1

Ion transport peptide-PP (ITP-PP)

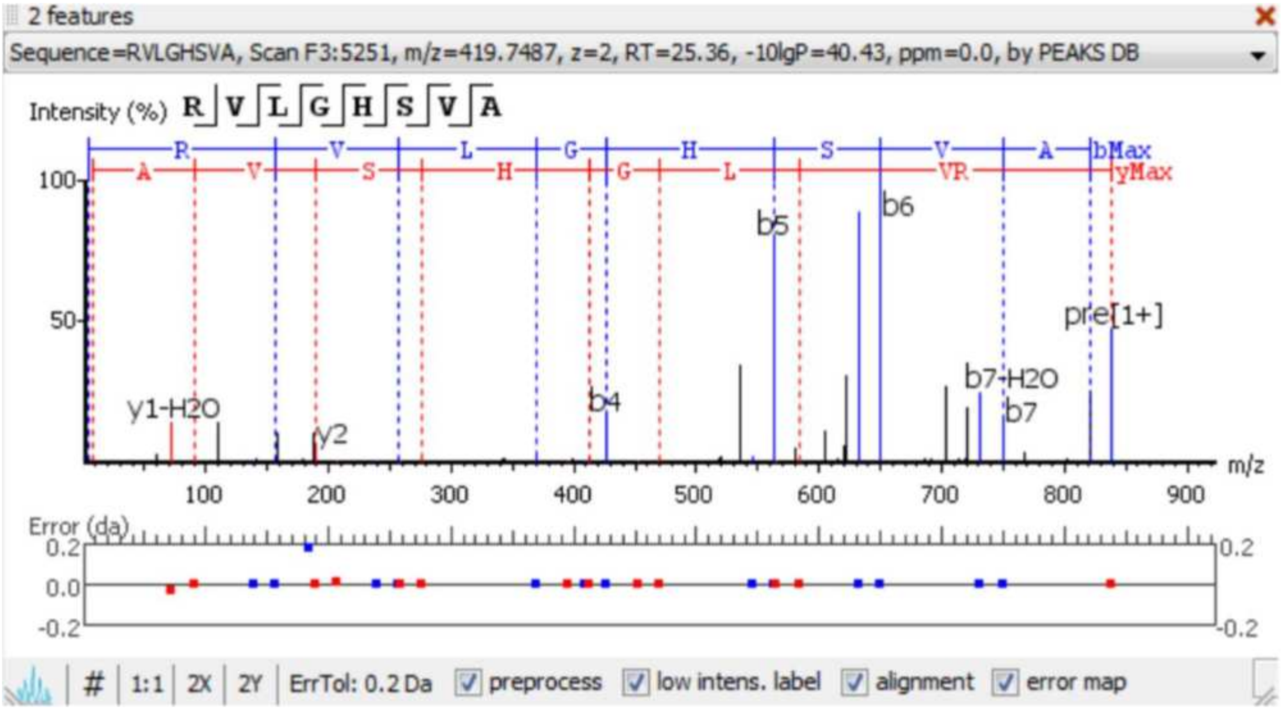

#	b	b-H2O	b-NH3	b (2+)	Seq	y	y-H2O	y-NH3	y (2+)	#
1	157.11	139.10	140.08	79.05	R					8
2	256.18	238.17	239.15	128.59	V	682.39	664.38	665.36	341.69	7
3	369.26	351.25	352.23	184.94	L	583.32	565.31	566.29	292.16	6
4	426.28	408.27	409.26	213.64	G	470.24	452.23	453.21	235.62	5
5	563.34	545.33	546.32	282.17	H	413.21	395.20	396.19	207.09	4
6	650.37	632.36	633.35	325.69	S	276.16	258.14	259.13	138.58	3
7	749.44	731.43	732.42	375.22	V	189.12	171.11	172.10	95.06	2
8					A	90.06	72.08	73.03	45.53	1

Ion transport peptide (ITP)_partial

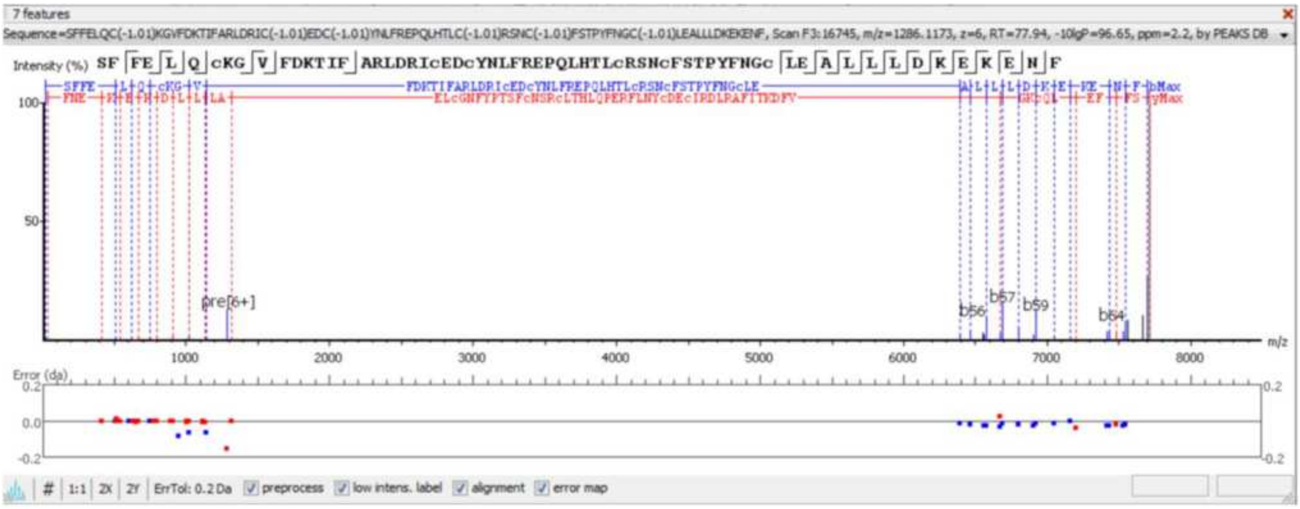

#	b	b-H2O	b-NH3	b (2+)	Seq	y	y-H2O	y-NH3	y (2+)	#
1	88.04	70.03	71.01	44.52	S					65
2	235.11	217.10	218.08	118.05	F	7624.62	7606.61	7607.59	3812.81	64
3	382.18	364.17	365.15	191.59	F	7477.57	7459.54	7460.52	3739.27	63
4	511.22	493.21	494.19	256.11	E	7330.48	7312.47	7313.45	3665.74	62
5	624.30	606.30	607.28	312.65	L	7201.48	7183.43	7184.41	3601.22	61
6	752.36	734.35	735.33	376.68	Q	7088.35	7070.34	7071.33	3544.68	60
7	854.36	836.35	837.34	427.68	C(-1.01)	6960.30	6942.29	6943.27	3480.65	59
8	982.46	964.45	965.43	491.73	K	6858.29	6840.28	6841.27	3429.65	58
9	1039.48	1021.47	1022.52	520.24	G	6730.20	6712.19	6713.17	3365.60	57
10	1138.61	1120.54	1121.52	569.77	V	6673.15	6655.17	6656.15	3337.09	56
11	1285.62	1267.61	1268.59	643.31	F	6574.11	6556.10	6557.08	3287.55	55
12	1400.64	1382.63	1383.62	700.82	D	6427.04	6409.03	6410.01	3214.02	54
13	1528.74	1510.73	1511.71	764.87	K	6312.01	6294.00	6294.99	3156.51	53
14	1629.79	1611.78	1612.76	815.39	T	6183.92	6165.91	6166.89	3092.46	52
15	1742.87	1724.86	1725.84	871.94	I	6082.87	6064.86	6065.84	3041.94	51
16	1889.94	1871.93	1872.91	945.55	F	5969.79	5951.78	5952.76	2985.39	50
17	1960.98	1942.97	1943.95	980.99	A	5822.72	5804.71	5805.69	2911.86	49
18	2117.08	2099.07	2100.05	1059.04	R	5751.68	5733.67	5734.66	2876.34	48
19	2230.16	2212.15	2213.13	1115.58	L	5595.58	5577.57	5578.55	2798.29	47
20	2345.19	2327.18	2328.16	1173.09	D	5482.50	5464.49	5465.47	2741.75	46
21	2501.29	2483.28	2484.26	1251.14	R	5367.47	5349.46	5350.44	2684.24	45
22	2614.37	2596.36	2597.35	1307.69	I	5211.37	5193.36	5194.34	2606.18	44
23	2716.37	2698.36	2699.35	1358.69	C(-1.01)	5098.29	5080.27	5081.26	2549.64	43
24	2845.42	2827.41	2828.39	1423.21	E	4996.28	4978.27	4979.26	2498.64	42
25	2960.44	2942.43	2943.42	1480.72	D	4867.24	4849.23	4850.21	2434.12	41
26	3062.45	3044.43	3045.42	1531.72	C(-1.01)	4752.21	4734.20	4735.19	2376.61	40
27	3225.51	3207.50	3208.48	1613.25	Y	4650.21	4632.20	4633.19	2325.61	39
28	3339.55	3321.54	3322.52	1670.28	N	4487.15	4469.14	4470.12	2244.07	38
29	3452.64	3434.62	3435.61	1726.82	L	4373.11	4355.10	4356.08	2187.05	37
30	3599.70	3581.69	3582.68	1800.35	F	4260.02	4242.01	4243.00	2130.51	36
31	3755.80	3737.79	3738.78	1878.40	R	4112.95	4094.94	4095.93	2056.98	35
32	3884.85	3866.84	3867.82	1942.92	E	3956.85	3938.84	3939.83	1978.93	34
33	3981.90	3963.89	3964.87	1991.45	P	3827.81	3809.80	3810.78	1914.41	33
34	4109.96	4091.95	4092.93	2055.48	Q	3730.76	3712.75	3713.73	1865.88	32
35	4223.04	4205.03	4206.02	2112.02	L	3602.70	3584.69	3585.67	1801.85	31
36	4360.10	4342.09	4343.08	2180.55	H	3489.61	3471.60	3472.59	1745.31	30
37	4461.15	4443.14	4444.12	2231.07	T	3352.56	3334.55	3335.53	1676.78	29
38	4574.23	4556.22	4557.21	2287.62	L	3251.51	3233.50	3234.48	1626.25	28
39	4676.23	4658.22	4659.21	2338.62	C(-1.01)	3138.42	3120.41	3121.40	1569.71	27
40	4832.34	4814.33	4815.31	2416.67	R	3036.42	3018.41	3019.40	1518.71	26
41	4919.37	4901.36	4902.34	2460.18	S	2880.32	2862.31	2863.29	1440.66	25
42	5033.41	5015.40	5016.38	2517.21	N	2793.29	2775.28	2776.26	1397.14	24
43	5135.41	5117.40	5118.39	2568.21	C(-1.01)	2679.25	2661.24	2662.22	1340.12	23
44	5282.48	5264.47	5265.45	2641.74	F	2577.25	2559.23	2560.22	1289.12	22
45	5369.51	5351.50	5352.49	2685.26	S	2430.18	2412.17	2413.15	1215.59	21
46	5470.56	5452.55	5453.53	2735.78	T	2343.15	2325.13	2326.12	1172.07	20
47	5567.61	5549.60	5550.59	2784.31	P	2242.10	2224.09	2225.07	1121.55	19
48	5730.68	5712.67	5713.65	2865.84	Y	2145.04	2127.03	2128.02	1073.02	18
49	5877.75	5859.73	5860.72	2939.37	F	1981.98	1963.97	1964.95	991.49	17
50	5991.79	5973.78	5974.76	2996.39	N	1834.91	1816.90	1817.89	917.96	16
51	6048.81	6030.80	6031.78	3024.90	G	1720.87	1702.86	1703.84	860.93	15
52	6150.81	6132.80	6133.78	3075.91	C(-1.01)	1663.85	1645.84	1646.82	832.42	14
53	6263.90	6245.88	6246.87	3132.45	L	1561.85	1543.84	1544.82	781.42	13
54	6392.96	6374.93	6375.91	3196.97	E	1448.76	1430.75	1431.74	724.88	12
55	6464.00	6445.96	6446.95	3232.49	A	1319.72	1301.71	1302.69	660.36	11
56	6577.09	6559.05	6560.06	3289.03	L	1248.68	1230.67	1231.66	624.85	10
57	6690.16	6672.13	6673.15	3345.57	L	1135.61	1117.59	1118.58	568.30	9
58	6803.25	6785.22	6786.20	3402.11	L	1022.52	1004.51	1005.50	511.76	8
59	6918.27	6900.24	6901.26	3459.63	D	909.44	891.42	892.41	455.22	7
60	7046.36	7028.34	7029.32	3523.67	K	794.41	776.40	777.38	397.70	6
61	7175.39	7157.39	7158.36	3588.20	E	666.31	648.30	649.29	333.65	5
62	7303.49	7285.48	7286.46	3652.24	K	537.27	519.24	520.24	269.13	4
63	7432.56	7414.55	7415.50	3716.76	E	409.17	391.16	392.14	205.09	3
64	7546.59	7528.56	7529.57	3773.79	N	280.13	262.12	263.10	140.56	2
65					F	166.09	148.08	149.06	83.54	1

Kinin-1 (K-1)

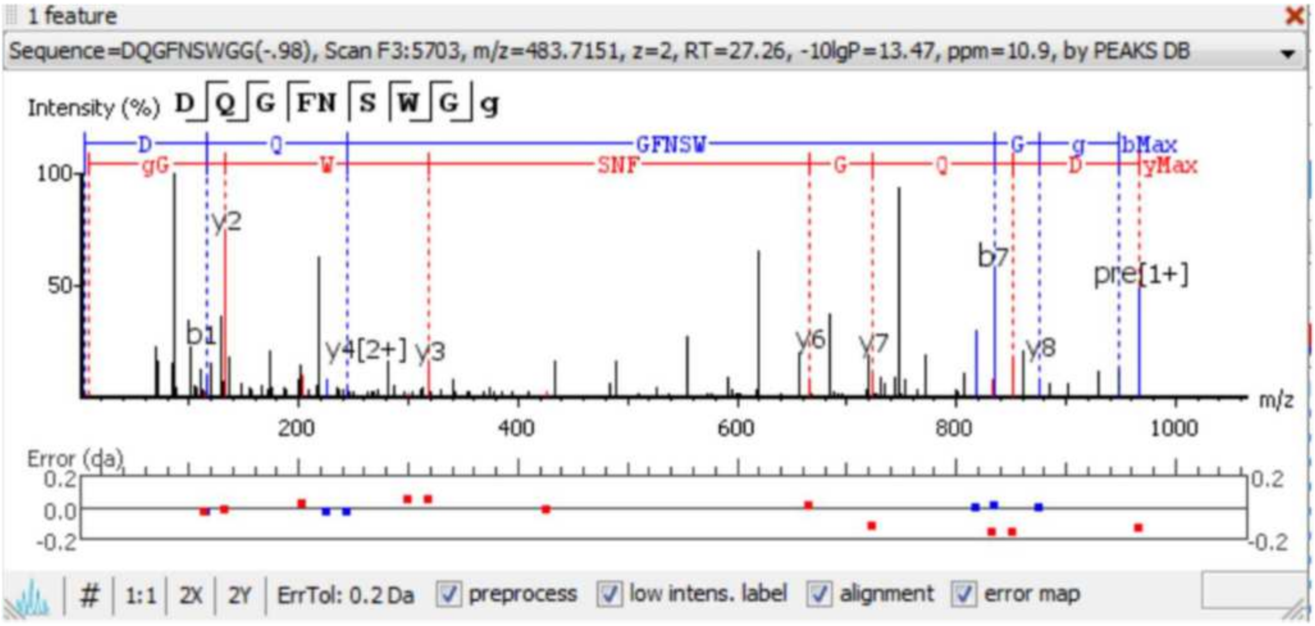

#	b	b-H2O	b-NH3	b (2+)	Seq	y	y-H2O	y-NH3	y (2+)	#
1	116.07	98.02	99.01	58.52	D					9
2	244.13	226.12	227.07	122.55	Q	851.54	833.53	834.35	426.21	8
3	301.11	283.10	284.09	151.06	G	723.44	705.31	706.29	362.16	7
4	448.18	430.17	431.16	224.59	F	666.28	648.29	649.27	333.65	6
5	562.23	544.22	545.20	281.61	N	519.23	501.22	502.20	260.12	5
6	649.26	631.25	632.23	325.13	S	405.19	387.18	388.16	203.07	4
7	835.32	817.31	818.31	418.17	W	318.09	300.08	301.13	159.58	3
8	892.36	874.35	875.33	446.68	G	132.10	114.07	115.09	66.54	2
9					G(-.98)	75.06	57.04	58.03	38.03	1

Kinin-2 (K-2)

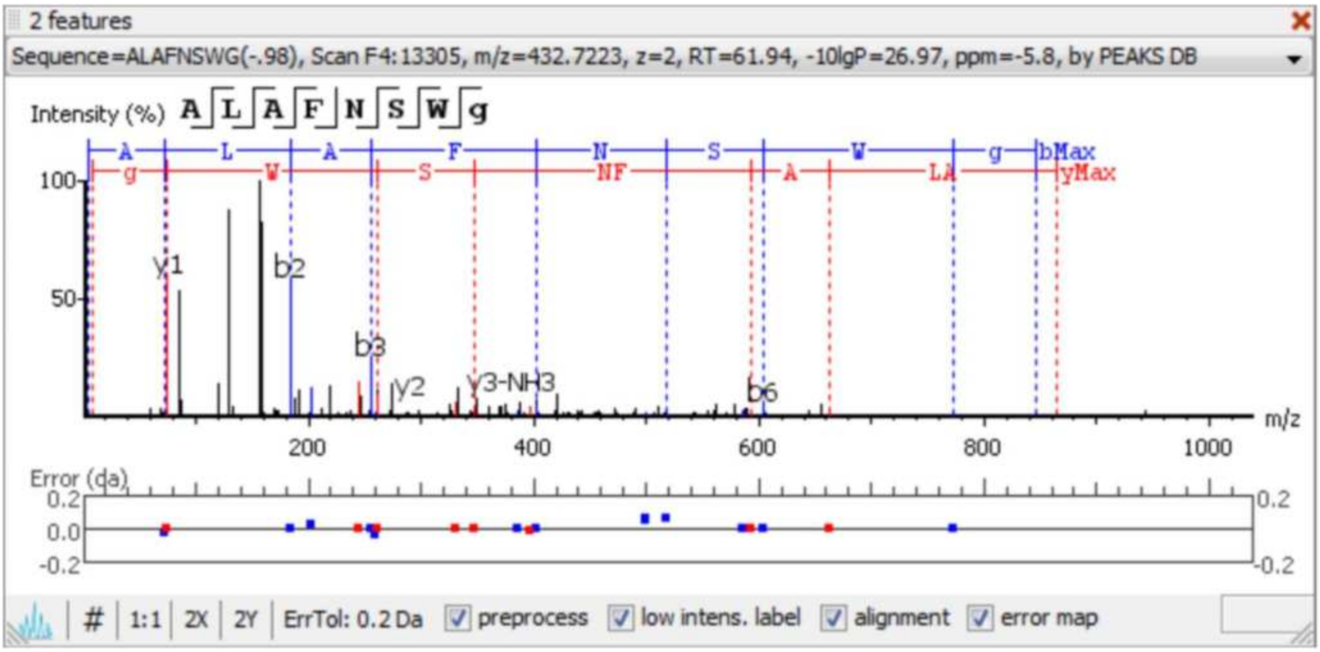

#	b	b-H2O	b-NH3	b (2+)	Seq	y	y-H2O	y-NH3	y (2+)	#
1	72.08	54.03	55.02	36.52	A					8
2	185.13	167.12	168.10	93.06	L	793.40	775.39	776.37	397.22	7
3	256.17	238.16	239.14	128.58	A	680.32	662.30	663.29	340.66	6
4	403.23	385.22	386.20	202.08	F	609.28	591.27	592.25	305.14	5
5	517.21	499.19	500.19	259.18	N	462.21	444.20	445.18	231.60	4
6	604.31	586.30	587.28	302.65	S	348.17	330.16	331.14	174.58	3
7	790.39	772.37	773.36	395.69	W	261.13	243.12	244.11	131.07	2
8					G(-.98)	75.06	57.04	58.03	38.03	1

Kinin-3 (K-3)

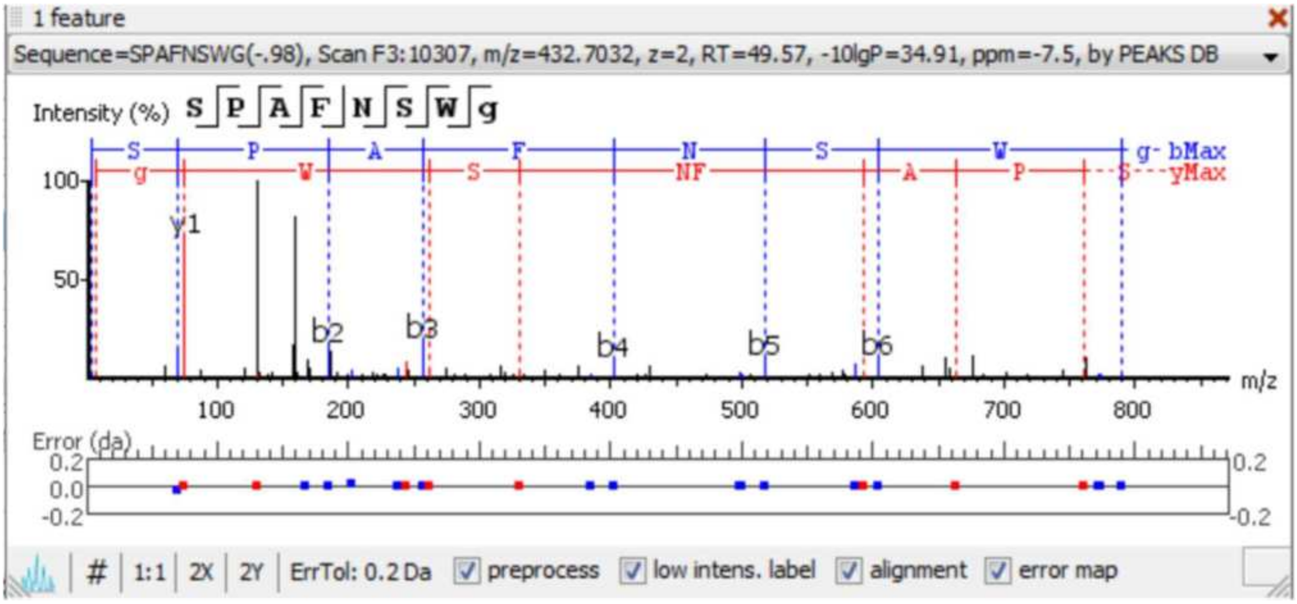

#	b	b-H2O	b-NH3	b (2+)	Seq	y	y-H2O	y-NH3	y (2+)	#
1	88.04	70.07	71.01	44.52	S					8
2	185.09	167.08	168.07	93.05	P	777.37	759.36	760.34	389.18	7
3	256.13	238.12	239.10	128.56	A	680.32	662.30	663.29	340.66	6
4	403.20	385.19	386.17	202.08	F	609.28	591.27	592.25	305.14	5
5	517.24	499.23	500.21	259.12	N	462.21	444.20	445.18	231.60	4
6	604.27	586.26	587.25	302.64	S	348.17	330.16	331.14	174.58	3
7	790.35	772.34	773.33	395.68	W	261.13	243.12	244.11	131.07	2
8					G(-.98)	75.06	57.04	58.03	38.03	1

Kinin-4 (K-4)

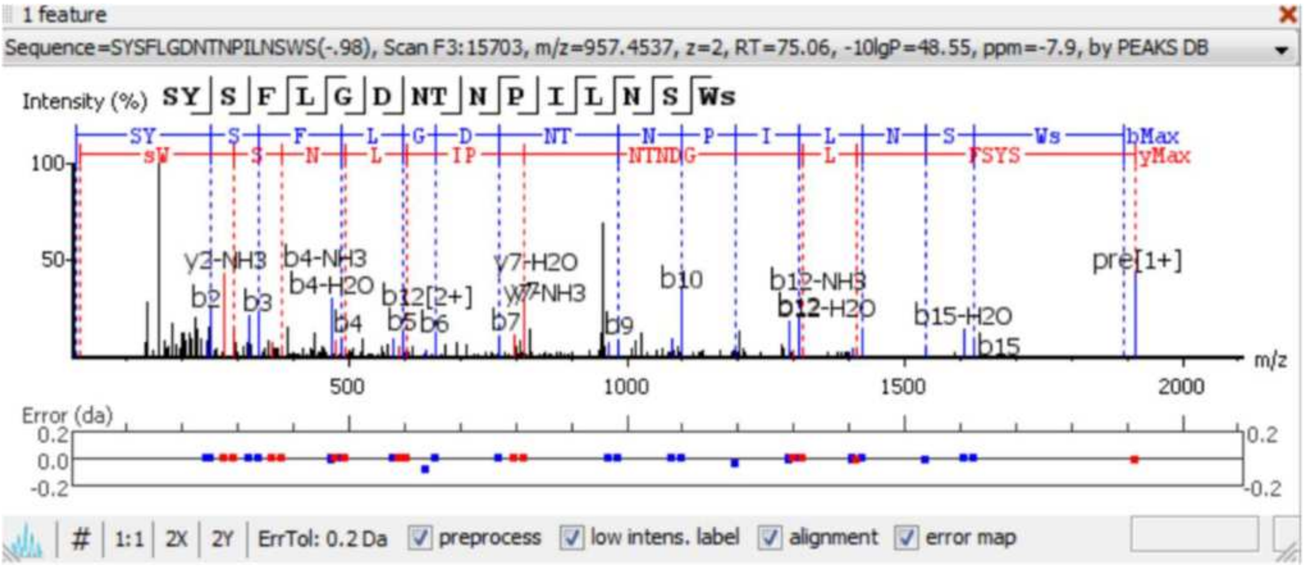

#	b	b-H2O	b-NH3	b (2+)	Seq	y	y-H2O	y-NH3	y (2+)	#
1	88.04	70.03	71.01	44.52	S					17
2	251.10	233.09	234.08	126.05	Y	1826.87	1808.86	1809.84	913.94	16
3	338.14	320.12	321.11	169.57	S	1663.81	1645.80	1646.78	832.40	15
4	485.20	467.19	468.20	243.10	F	1576.78	1558.76	1559.75	788.89	14
5	598.29	580.28	581.26	299.64	L	1429.71	1411.71	1412.68	715.35	13
6	655.31	637.39	638.28	328.15	G	1316.63	1298.61	1299.61	658.81	12
7	770.33	752.33	753.31	385.67	D	1259.60	1241.59	1242.57	630.30	11
8	884.38	866.37	867.35	442.69	N	1144.57	1126.56	1127.55	572.79	10
9	985.42	967.41	968.40	493.21	T	1030.53	1012.52	1013.50	515.77	9
10	1099.47	1081.46	1082.45	550.23	N	929.48	911.47	912.46	465.24	8
11	1196.58	1178.51	1179.50	598.77	P	815.44	797.43	798.42	408.22	7
12	1309.61	1291.60	1292.60	655.31	I	718.39	700.38	701.36	359.69	6
13	1422.69	1404.69	1405.69	711.85	L	605.30	587.29	588.28	303.15	5
14	1536.75	1518.72	1519.71	768.87	N	492.22	474.21	475.19	246.61	4
15	1623.78	1605.75	1606.74	812.38	S	378.18	360.17	361.15	189.59	3
16	1809.84	1791.83	1792.82	905.42	W	291.15	273.13	274.12	146.07	2
17					S(-.98)	105.07	87.06	88.04	53.03	1

Kinin-5 (K-5)_Q

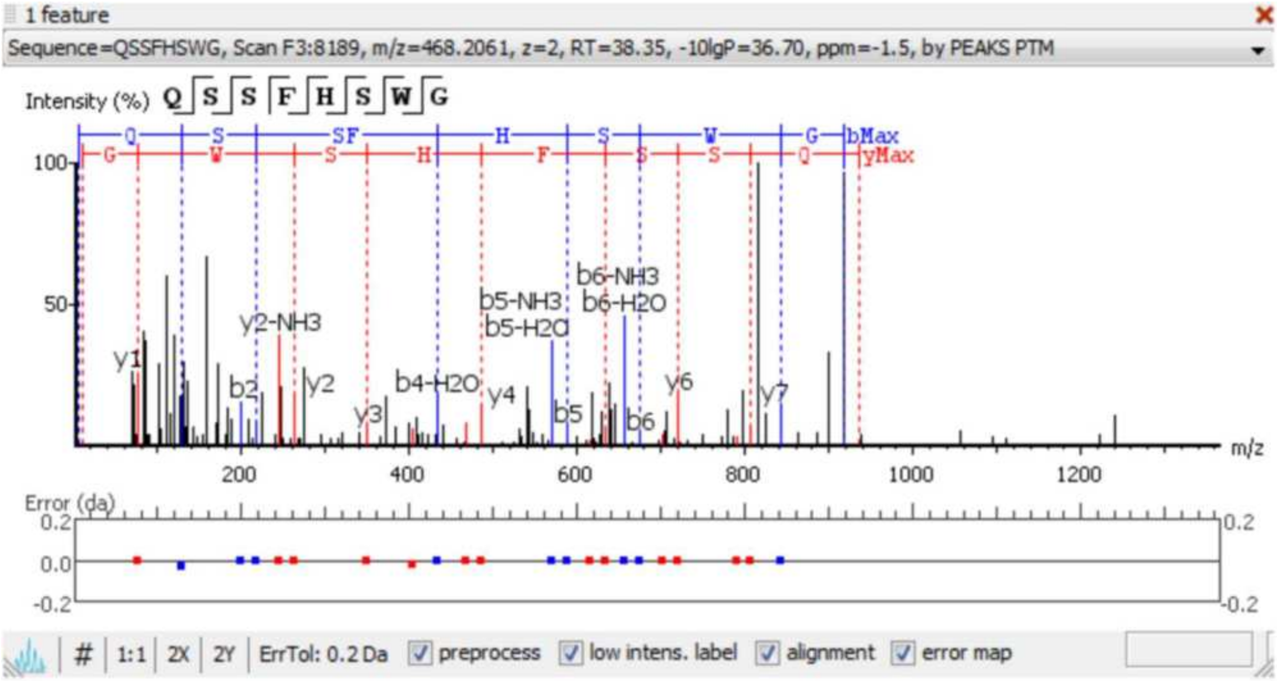

#	b	b-H2O	b-NH3	b (2+)	Seq	y	y-H2O	y-NH3	y (2+)	#
1	129.10	111.06	112.04	65.03	Q					8
2	216.10	198.09	199.07	108.55	S	807.34	789.32	790.32	404.19	7
3	303.13	285.12	286.10	152.07	S	720.31	702.30	703.28	360.65	6
4	450.20	432.19	433.17	225.60	F	633.28	615.27	616.25	317.14	5
5	587.26	569.25	570.23	294.13	H	486.21	468.20	469.18	243.60	4
6	674.29	656.28	657.26	337.64	S	349.15	331.14	332.12	175.08	3
7	860.37	842.36	843.34	430.68	W	262.12	244.11	245.09	131.56	2
8					G	76.04	58.03	59.01	38.52	1

Kinin-5 (K-5)_pQ

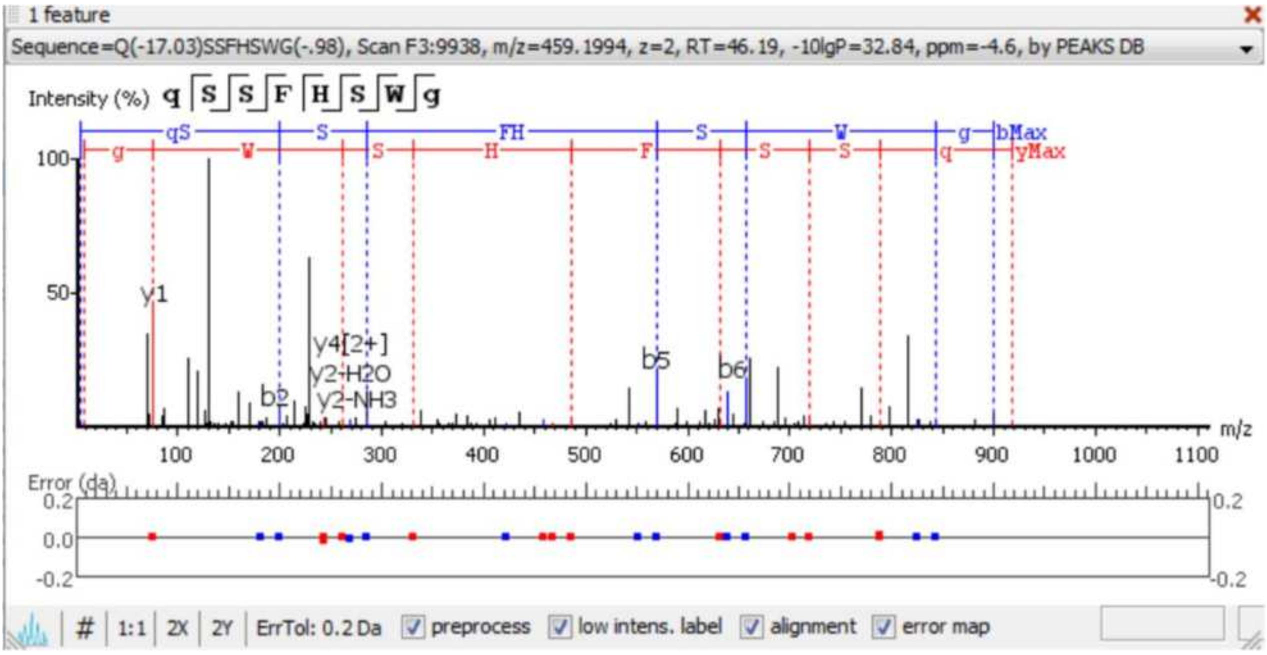

#	b	b-H2O	b-NH3	b (2+)	Seq	y	y-H2O	y-NH3	y (2+)	#
1	112.04	94.03	95.01	56.52	Q(-17.03)					8
2	199.07	181.06	182.04	100.04	S	806.36	788.33	789.33	403.68	7
3	286.10	268.11	269.08	143.55	S	719.33	701.32	702.30	360.16	6
4	433.17	415.16	416.15	217.09	F	632.29	614.28	615.27	316.65	5
5	570.23	552.22	553.20	285.62	H	485.23	467.22	468.20	243.13	4
6	657.26	639.25	640.24	329.13	S	348.17	330.16	331.14	174.58	3
7	843.34	825.33	826.32	422.17	W	261.13	243.13	244.11	131.07	2
8					G(-.98)	75.06	57.04	58.03	38.03	1

Kinin-6 (K-6)

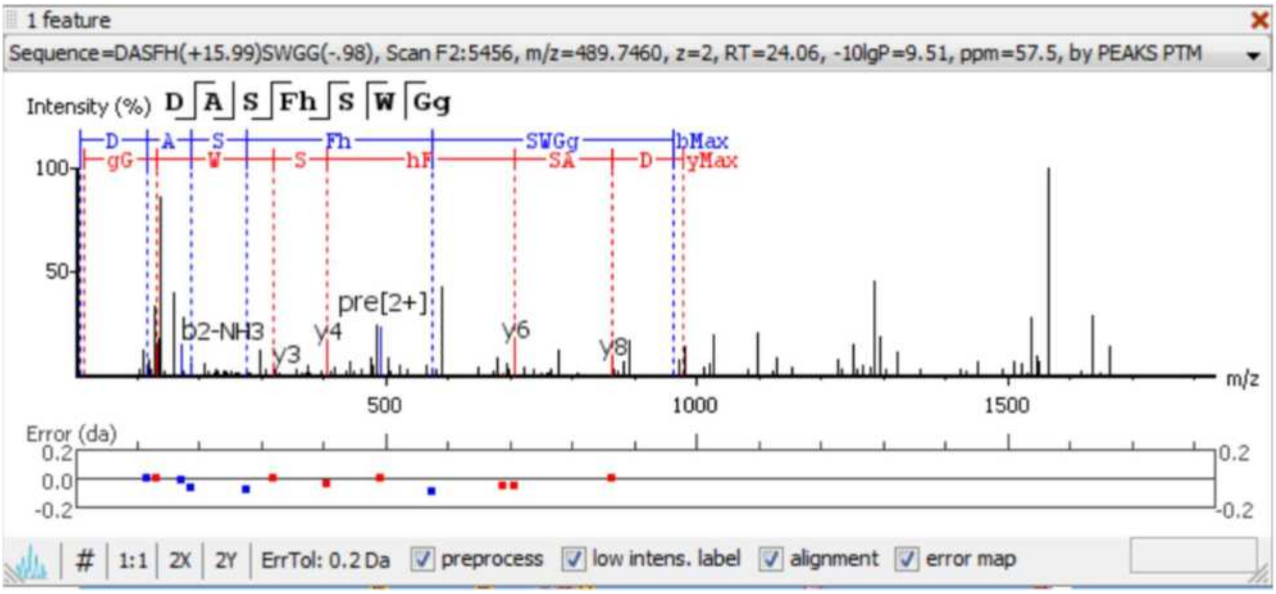

#	b	b-H2O	b-NH3	b (2+)	Seq	y	y-H2O	y-NH3	y (2+)	#
1	116.03	98.02	99.01	58.52	D					9
2	187.14	169.06	170.06	94.04	A	863.37	845.37	846.35	432.19	8
3	274.19	256.09	257.08	137.55	S	792.34	774.33	775.32	396.67	7
4	421.17	403.16	404.15	211.09	F	705.37	687.30	688.34	353.16	6
5	574.33	556.22	557.20	287.61	H(+15.99)	558.24	540.23	541.21	279.62	5
6	661.26	643.25	644.23	331.13	S	405.23	387.18	388.16	203.09	4
7	847.34	829.33	830.31	424.17	W	318.15	300.15	301.13	159.58	3
8	904.36	886.35	887.33	452.68	G	132.08	114.07	115.05	66.54	2
9					G(-.98)	75.06	57.04	58.03	38.03	1

Kinin-PP-1 (K-PP-1)_part 1

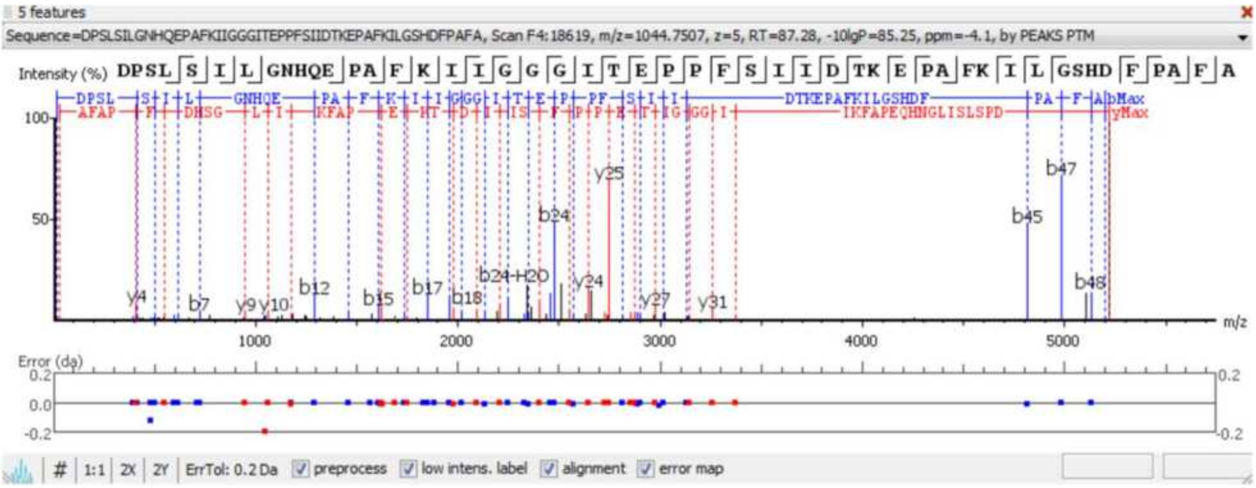

#	b	b-H2O	b-NH3	b (2+)	Seq	y	y-H2O	y-NH3	y (2+)	#
1	116.03	98.02	99.01	58.52	D					49
2	213.09	195.08	196.06	107.04	P	5104.68	5086.67	5087.65	2552.84	48
3	300.12	282.11	283.09	150.56	S	5007.63	4989.62	4990.60	2504.31	47
4	413.20	395.19	396.18	207.10	L	4920.60	4902.59	4903.57	2460.80	46
5	500.24	482.23	483.33	250.62	S	4807.51	4789.50	4790.49	2404.26	45
6	613.32	595.31	596.29	307.16	I	4720.48	4702.47	4703.45	2360.74	44
7	726.40	708.39	709.38	363.70	L	4607.40	4589.39	4590.37	2304.20	43
8	783.43	765.41	766.40	392.21	G	4494.31	4476.30	4477.29	2247.66	42
9	897.47	879.46	880.44	449.23	N	4437.29	4419.28	4420.26	2219.15	41
10	1034.53	1016.52	1017.50	517.76	H	4323.25	4305.24	4306.22	2162.12	40
11	1162.59	1144.58	1145.56	581.79	Q	4186.19	4168.18	4169.16	2093.59	39
12	1291.63	1273.62	1274.60	646.31	E	4058.13	4040.12	4041.10	2029.57	38
13	1388.68	1370.67	1371.65	694.84	P	3929.09	3911.08	3912.06	1965.04	37
14	1459.72	1441.71	1442.69	730.36	A	3832.04	3814.03	3815.01	1916.52	36
15	1606.79	1588.78	1589.76	803.89	F	3761.00	3742.99	3743.97	1881.00	35
16	1734.89	1716.87	1717.85	867.94	K	3613.93	3595.92	3596.90	1807.47	34
17	1847.96	1829.96	1830.94	924.48	I	3485.84	3467.82	3468.81	1743.42	33
18	1961.05	1943.04	1944.02	981.02	I	3372.76	3354.74	3355.72	1686.88	32
19	2018.07	2000.06	2001.04	1009.54	G	3259.67	3241.66	3242.64	1630.35	31
20	2075.09	2057.08	2058.07	1038.05	G	3202.65	3184.64	3185.62	1601.82	30
21	2132.13	2114.10	2115.09	1066.56	G	3145.63	3127.61	3128.60	1573.31	29
22	2245.19	2227.19	2228.17	1123.10	I	3088.60	3070.59	3071.58	1544.80	28
23	2346.26	2328.23	2329.22	1173.63	T	2975.52	2957.51	2958.49	1488.26	27
24	2475.29	2457.28	2458.26	1238.14	E	2874.48	2856.46	2857.44	1437.74	26
25	2572.36	2554.33	2555.31	1286.67	P	2745.43	2727.41	2728.40	1373.21	25
26	2669.39	2651.38	2652.37	1335.20	P	2648.38	2630.37	2631.35	1324.69	24
27	2816.46	2798.45	2799.44	1408.73	F	2551.33	2533.31	2534.30	1276.16	23
28	2903.50	2885.49	2886.47	1452.25	S	2404.26	2386.24	2387.23	1202.63	22
29	3016.58	2998.59	2999.55	1508.79	I	2317.22	2299.21	2300.20	1159.11	21
30	3129.66	3111.65	3112.64	1565.33	I	2204.14	2186.13	2187.11	1102.57	20
31	3244.69	3226.68	3227.66	1622.86	D	2091.06	2073.04	2074.03	1046.03	19
32	3345.74	3327.73	3328.71	1673.37	T	1976.04	1958.02	1959.00	988.51	18
33	3473.83	3455.82	3456.80	1737.42	K	1874.98	1856.97	1857.95	937.99	17
34	3602.87	3584.86	3585.85	1801.94	E	1746.88	1728.87	1729.86	873.94	16
35	3699.93	3681.92	3682.90	1850.46	P	1617.84	1599.83	1600.82	809.42	15
36	3770.96	3752.95	3753.94	1885.99	A	1520.79	1502.78	1503.76	760.89	14
37	3918.03	3900.02	3901.01	1959.52	F	1449.75	1431.74	1432.73	725.38	13
38	4046.13	4028.12	4029.10	2023.56	K	1302.68	1284.67	1285.66	651.84	12
39	4159.21	4141.20	4142.19	2080.11	I	1174.61	1156.58	1157.56	587.79	11
40	4272.30	4254.29	4255.27	2136.65	L	1061.50	1043.49	1044.48	531.25	10
41	4329.32	4311.31	4312.29	2165.16	G	948.42	930.41	931.39	474.71	9
42	4416.35	4398.34	4399.32	2208.67	S	891.40	873.39	874.37	446.20	8
43	4553.41	4535.40	4536.38	2277.20	H	804.37	786.36	787.34	402.68	7
44	4668.44	4650.42	4651.41	2334.72	D	667.31	649.30	650.28	334.15	6
45	4815.51	4797.49	4798.48	2408.25	F	552.28	534.27	535.25	276.64	5
46	4912.56	4894.55	4895.53	2456.78	P	405.21	387.20	388.19	203.11	4
47	4983.60	4965.58	4966.57	2492.30	A	308.16	290.15	291.13	154.58	3
48	5130.67	5112.65	5113.64	2565.83	F	237.12	219.11	220.10	119.06	2
49					A	90.05	72.04	73.03	45.53	1

Kinin-PP-1 (K-PP-1)_part 2

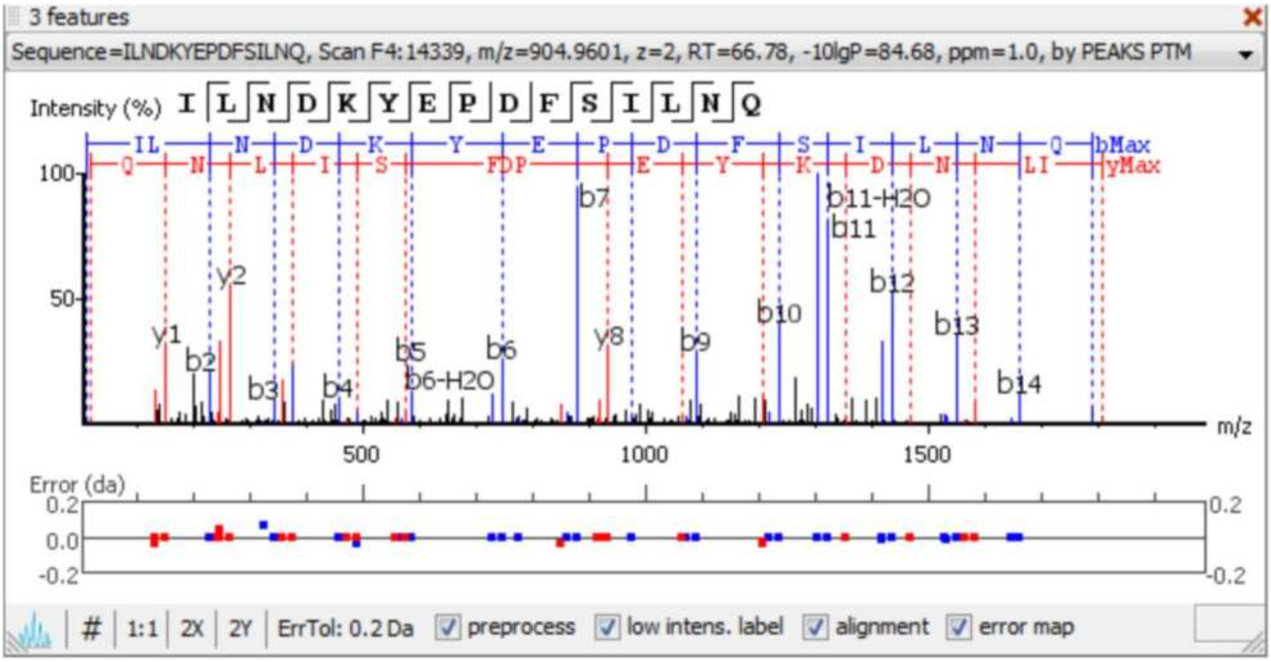

#	b	b-H2O	b-NH3	b (2+)	Seq	y	y-H2O	y-NH3	y (2+)	#
1	114.09	96.08	97.06	57.55	I					15
2	227.18	209.17	210.15	114.09	L	1695.82	1677.81	1678.80	848.45	14
3	341.22	323.13	324.12	171.11	N	1582.74	1564.73	1565.72	791.87	13
4	456.25	438.24	439.22	228.62	D	1468.69	1450.68	1451.67	734.85	12
5	584.34	566.33	567.31	292.67	K	1353.67	1335.66	1336.64	677.33	11
6	747.40	729.39	730.38	374.20	Y	1225.57	1207.59	1208.59	613.29	10
7	876.45	858.43	859.43	438.72	E	1062.51	1044.50	1045.48	531.76	9
8	973.50	955.49	956.47	487.29	P	933.47	915.45	916.44	467.23	8
9	1088.53	1070.52	1071.50	544.76	D	836.41	818.40	819.39	418.71	7
10	1235.60	1217.58	1218.57	618.30	F	721.39	703.38	704.36	361.19	6
11	1322.63	1304.62	1305.60	661.81	S	574.32	556.31	557.29	287.66	5
12	1435.71	1417.70	1418.70	718.36	I	487.29	469.28	470.26	244.09	4
13	1548.80	1530.79	1531.78	774.90	L	374.20	356.19	357.18	187.60	3
14	1662.84	1644.83	1645.81	831.92	N	261.12	243.11	244.09	131.06	2
15					Q	147.08	129.10	130.05	74.04	1

Kinin-PP-1$^{1\text{-}53}$ (K-PP-1$^{1\text{-}53}$)

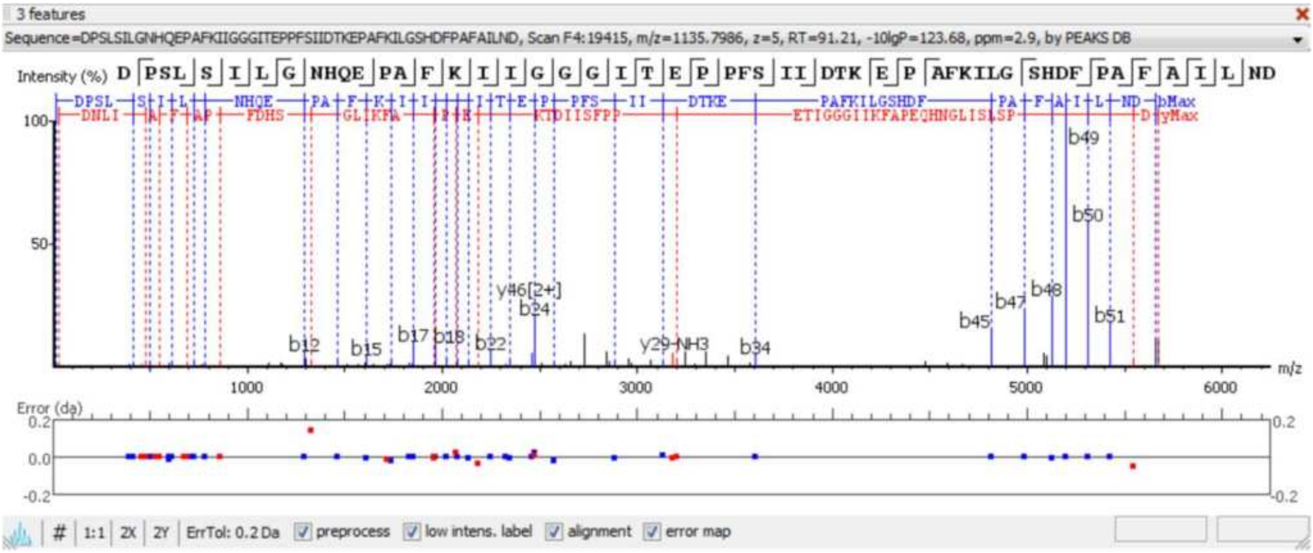

#	b	b-H2O	b-NH3	b (2+)	Seq	y	y-H2O	y-NH3	y (2+)	#
1	116.03	98.02	99.01	58.52	D					53
2	213.09	195.08	196.06	107.04	P	5559.92	5541.91	5542.95	2780.46	52
3	300.12	282.11	283.09	150.56	S	5462.87	5444.86	5445.84	2731.93	51
4	413.20	395.19	396.18	207.10	L	5375.84	5357.82	5358.81	2688.42	50
5	500.24	482.23	483.21	250.62	S	5262.75	5244.74	5245.72	2631.88	49
6	613.32	595.31	596.31	307.16	I	5175.72	5157.71	5158.69	2588.36	48
7	726.40	708.39	709.38	363.70	L	5062.64	5044.62	5045.61	2531.82	47
8	783.42	765.41	766.40	392.21	G	4949.55	4931.54	4932.52	2475.26	46
9	897.47	879.46	880.44	449.23	N	4892.53	4874.52	4875.50	2446.76	45
10	1034.53	1016.52	1017.50	517.76	H	4778.49	4760.48	4761.46	2389.74	44
11	1162.59	1144.58	1145.56	581.79	Q	4641.43	4623.42	4624.40	2321.21	43
12	1291.63	1273.62	1274.60	646.31	E	4513.37	4495.36	4496.34	2257.18	42
13	1388.68	1370.67	1371.65	694.84	P	4384.33	4366.32	4367.30	2192.66	41
14	1459.72	1441.71	1442.69	730.36	A	4287.27	4269.26	4270.25	2144.14	40
15	1606.80	1588.78	1589.76	803.89	F	4216.24	4198.23	4199.21	2108.62	39
16	1734.91	1716.87	1717.85	867.94	K	4069.17	4051.16	4052.14	2035.08	38
17	1847.97	1829.95	1830.94	924.48	I	3941.07	3923.06	3924.05	1971.04	37
18	1961.04	1943.04	1944.02	981.02	I	3827.99	3809.98	3810.96	1914.49	36
19	2018.07	2000.06	2001.04	1009.54	G	3714.91	3696.89	3697.88	1857.95	35
20	2075.09	2057.08	2058.07	1038.05	G	3657.88	3639.87	3640.86	1829.44	34
21	2132.13	2114.10	2115.09	1066.56	G	3600.86	3582.85	3583.84	1800.93	33
22	2245.20	2227.19	2228.17	1123.10	I	3543.84	3525.83	3526.81	1772.42	32
23	2346.25	2328.24	2329.22	1173.62	T	3430.76	3412.75	3413.73	1715.90	31
24	2475.26	2457.28	2458.26	1238.14	E	3329.71	3311.70	3312.68	1665.35	30
25	2572.37	2554.33	2555.31	1286.67	P	3200.66	3182.66	3183.65	1600.83	29
26	2669.39	2651.38	2652.37	1335.20	P	3103.61	3085.60	3086.59	1552.31	28
27	2816.46	2798.45	2799.44	1408.73	F	3006.56	2988.55	2989.53	1503.78	27
28	2903.49	2885.49	2886.47	1452.25	S	2859.49	2841.48	2842.47	1430.25	26
29	3016.58	2998.57	2999.55	1508.79	I	2772.46	2754.45	2755.43	1386.73	25
30	3129.65	3111.65	3112.64	1565.33	I	2659.38	2641.37	2642.35	1330.19	24
31	3244.69	3226.68	3227.66	1622.84	D	2546.29	2528.28	2529.27	1273.65	23
32	3345.74	3327.73	3328.71	1673.37	T	2431.27	2413.26	2414.24	1216.13	22
33	3473.83	3455.82	3456.80	1737.42	K	2330.22	2312.21	2313.19	1165.61	21
34	3602.87	3584.86	3585.85	1801.94	E	2202.12	2184.11	2185.14	1101.56	20
35	3699.93	3681.92	3682.90	1850.46	P	2073.05	2055.07	2056.05	1037.04	19
36	3770.96	3752.95	3753.94	1885.98	A	1976.03	1958.02	1959.02	988.51	18
37	3918.03	3900.02	3901.01	1959.52	F	1904.99	1886.98	1887.96	953.00	17
38	4046.13	4028.12	4029.10	2023.56	K	1757.92	1739.91	1740.90	879.46	16
39	4159.21	4141.20	4142.19	2080.11	I	1629.83	1611.82	1612.80	815.41	15
40	4272.30	4254.29	4255.27	2136.65	L	1516.74	1498.73	1499.72	758.87	14
41	4329.32	4311.31	4312.29	2165.16	G	1403.66	1385.65	1386.63	702.33	13
42	4416.35	4398.34	4399.32	2208.67	S	1346.64	1328.63	1329.46	673.82	12
43	4553.41	4535.40	4536.38	2277.20	H	1259.61	1241.59	1242.58	630.30	11
44	4668.44	4650.42	4651.41	2334.72	D	1122.55	1104.54	1105.52	561.77	10
45	4815.51	4797.49	4798.48	2408.25	F	1007.52	989.51	990.49	504.26	9
46	4912.56	4894.55	4895.53	2456.78	P	860.45	842.44	843.42	430.73	8
47	4983.59	4965.58	4966.57	2492.30	A	763.40	745.39	746.37	382.20	7
48	5130.68	5112.65	5113.64	2565.83	F	692.36	674.35	675.34	346.68	6
49	5201.70	5183.69	5184.67	2601.35	A	545.29	527.28	528.27	273.15	5
50	5314.78	5296.77	5297.76	2657.89	I	474.26	456.25	457.23	237.63	4
51	5427.86	5409.86	5410.84	2714.43	L	361.17	343.16	344.14	181.09	3
52	5541.91	5523.90	5524.88	2771.46	N	248.09	230.08	231.06	124.54	2
53					D	134.04	116.03	117.02	67.52	1

Kinin-8 (K-8)

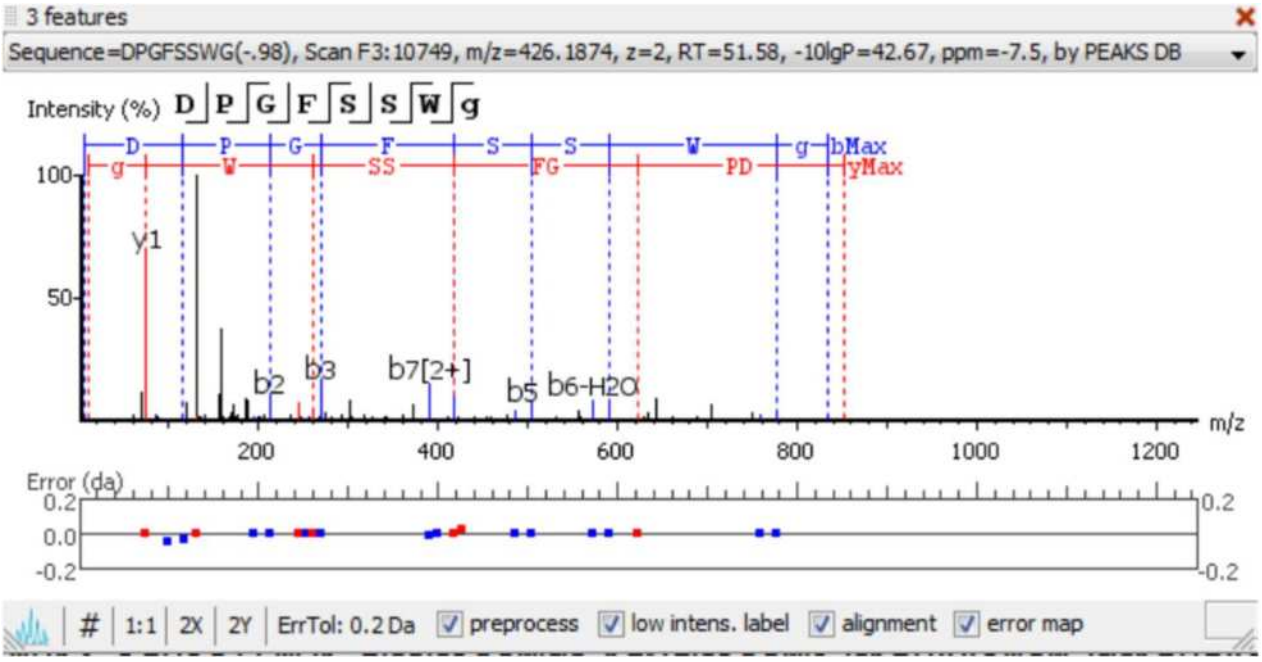

#	b	b-H2O	b-NH3	b (2+)	Seq	y	y-H2O	y-NH3	y (2+)	#
1	116.03	98.02	99.01	58.52	D					8
2	213.09	195.08	196.06	107.04	P	736.34	718.33	719.31	368.67	7
3	270.11	252.10	253.17	135.55	G	639.29	621.28	622.26	320.14	6
4	417.18	399.17	400.16	209.09	F	582.27	564.26	565.24	291.63	5
5	504.21	486.20	487.18	252.60	S	435.20	417.18	418.17	218.10	4
6	591.24	573.23	574.21	296.12	S	348.17	330.16	331.14	174.58	3
7	777.32	759.31	760.29	389.16	W	261.13	243.12	244.09	131.07	2
8					G(-.98)	75.06	57.04	58.03	38.03	1

Kinin-9 (K-9)

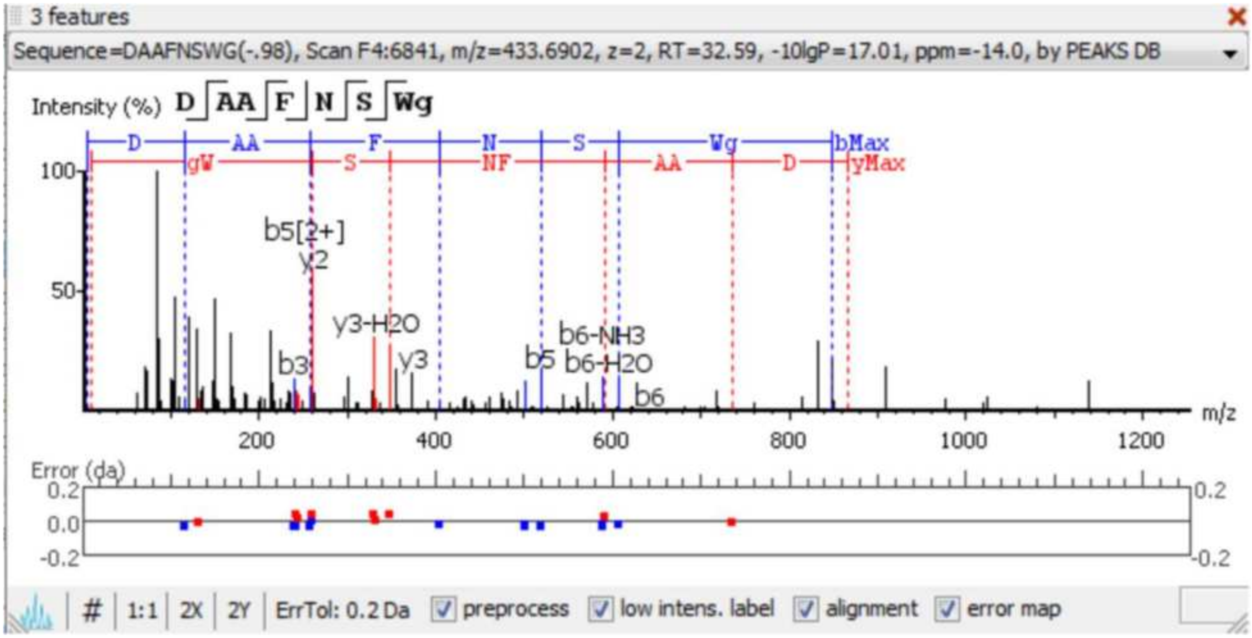

#	b	b-H2O	b-NH3	b (2+)	Seq	y	y-H2O	y-NH3	y (2+)	#
1	116.07	98.02	99.01	58.52	D					8
2	187.07	169.06	170.04	94.04	A	751.35	733.34	734.35	376.18	7
3	258.14	240.13	241.12	129.55	A	680.32	662.30	663.29	340.66	6
4	405.21	387.17	388.15	203.09	F	609.28	591.27	592.22	305.14	5
5	519.26	501.25	502.23	260.11	N	462.21	444.20	445.18	231.60	4
6	606.29	588.28	589.26	303.63	S	348.12	330.11	331.14	174.58	3
7	792.33	774.32	775.30	396.67	W	261.09	243.08	244.09	131.08	2
8					G(-.98)	75.06	57.04	58.03	38.03	1

Kinin-10 (K-10)

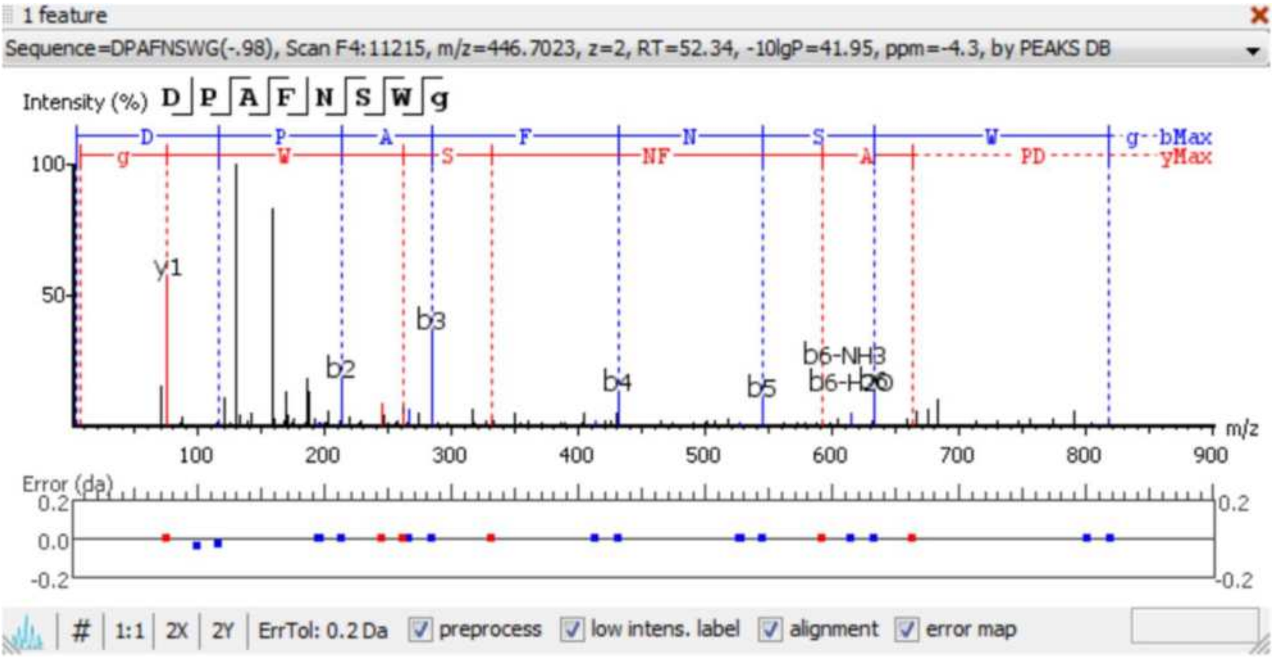

#	b	b-H2O	b-NH3	b (2+)	Seq	y	y-H2O	y-NH3	y (2+)	#
1	116.07	98.02	99.06	58.52	D					8
2	213.09	195.08	196.06	107.04	P	777.37	759.36	760.34	389.18	7
3	284.12	266.11	267.10	142.56	A	680.32	662.30	663.29	340.66	6
4	431.19	413.18	414.17	216.10	F	609.28	591.27	592.25	305.14	5
5	545.24	527.22	528.21	273.12	N	462.21	444.20	445.18	231.60	4
6	632.27	614.26	615.25	316.63	S	348.17	330.16	331.14	174.58	3
7	818.35	800.34	801.33	409.67	W	261.13	243.12	244.11	131.07	2
8					G(-.98)	75.06	57.04	58.03	38.03	1

Kinin-11 (K-11)

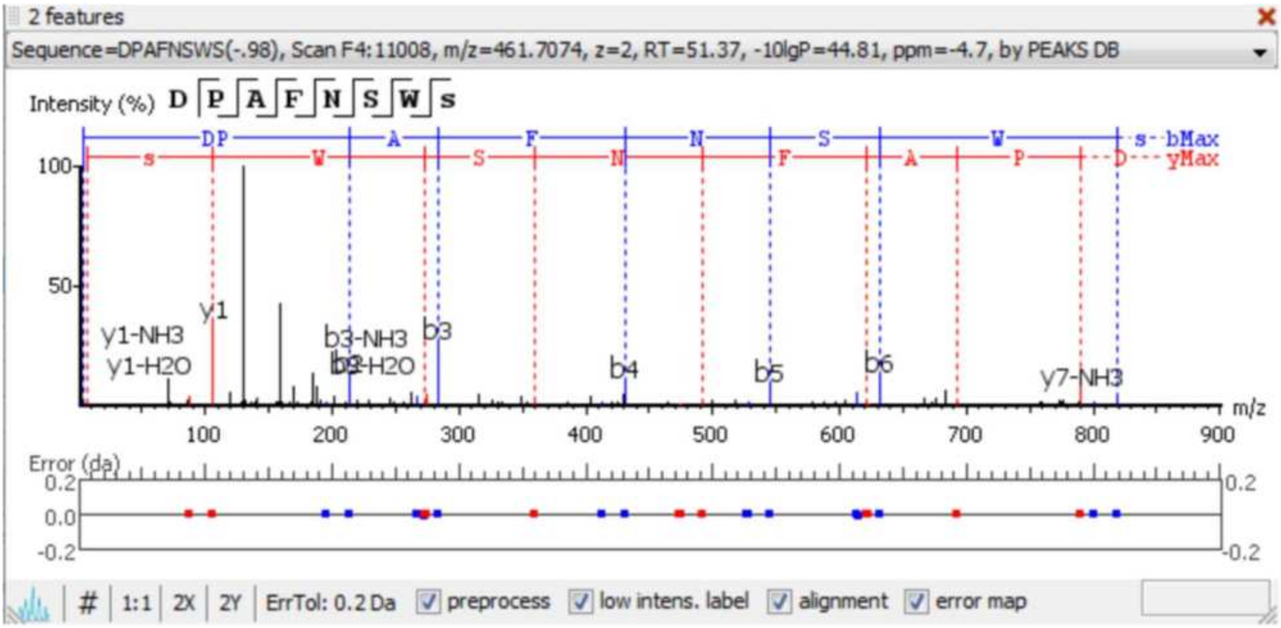

#	b	b-H2O	b-NH3	b (2+)	Seq	y	y-H2O	y-NH3	y (2+)	#
1	116.03	98.02	99.01	58.52	D					8
2	213.09	195.08	196.06	107.04	P	807.38	789.37	790.35	404.19	7
3	284.12	266.11	267.10	142.56	A	710.33	692.31	693.30	355.66	6
4	431.19	413.18	414.17	216.10	F	639.29	621.28	622.26	320.14	5
5	545.24	527.23	528.21	273.13	N	492.22	474.21	475.19	246.61	4
6	632.27	614.26	615.25	316.63	S	378.18	360.17	361.15	189.59	3
7	818.35	800.34	801.32	409.67	W	291.15	273.13	274.12	146.07	2
8					S(-.98)	105.07	87.06	88.04	53.03	1

Kinin-12 (K-12)

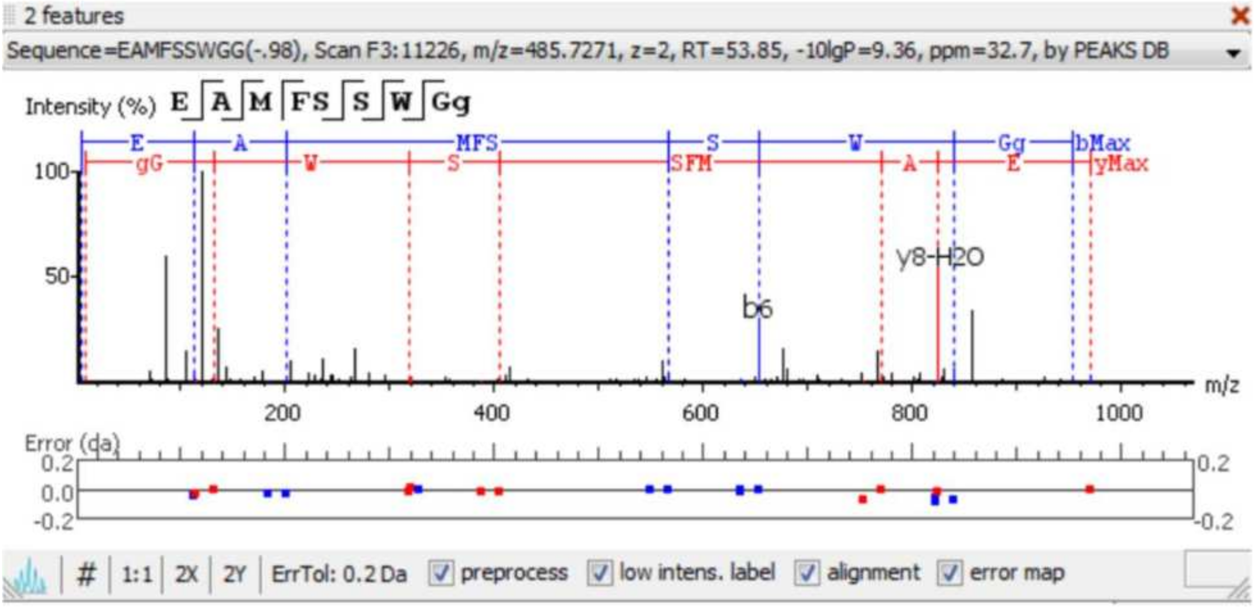

#	b	b-H2O	b-NH3	b (2+)	Seq	y	y-H2O	y-NH3	y (2+)	#
1	130.05	112.04	113.02	65.53	E					9
2	201.12	183.11	184.06	101.04	A	841.37	823.36	824.34	421.18	8
3	332.13	314.12	315.10	166.56	M	770.33	752.47	753.30	385.66	7
4	479.26	461.19	462.17	240.10	F	639.29	621.28	622.26	320.14	6
5	566.29	548.28	549.20	283.61	S	492.22	474.21	475.19	246.61	5
6	653.32	635.31	636.23	327.20	S	405.19	387.18	388.16	203.14	4
7	839.39	821.33	822.44	420.17	W	318.17	300.16	301.13	159.58	3
8	896.36	878.35	879.33	448.68	G	132.10	114.07	115.05	66.54	2
9					G(-.98)	75.06	57.04	58.03	38.03	1

Kinin-13 (K-13)

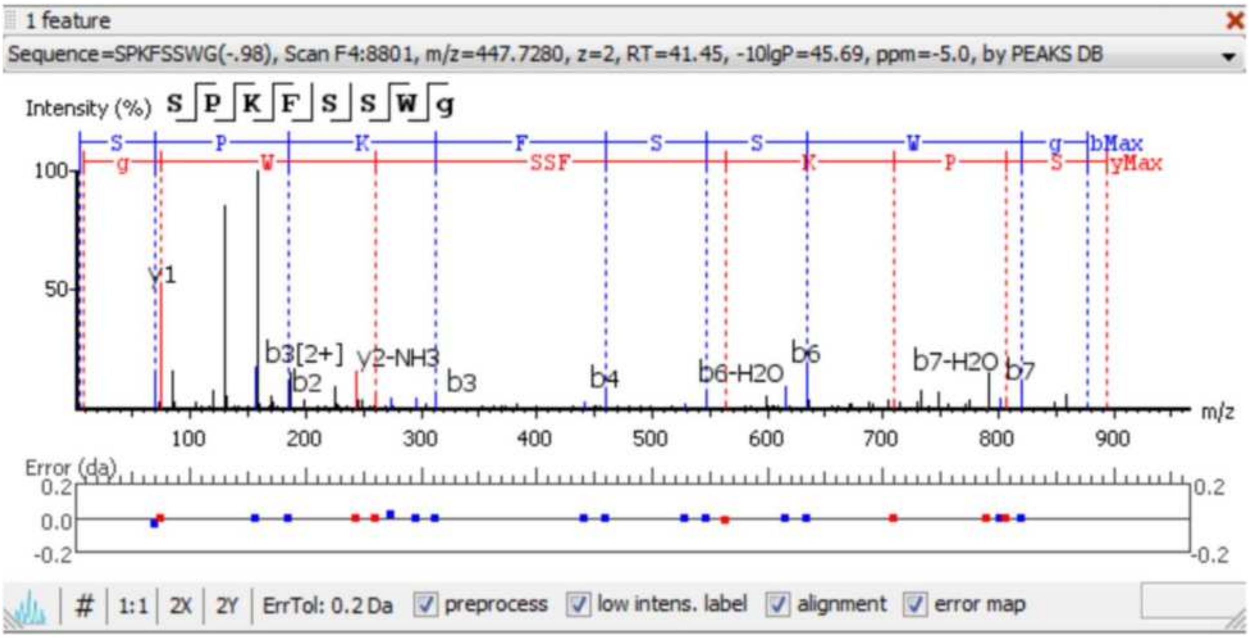

#	b	b-H2O	b-NH3	b (2+)	Seq	y	y-H2O	y-NH3	y (2+)	#
1	116.03	98.02	99.01	58.52	D					8
2	213.09	195.08	196.06	107.04	P	807.38	789.37	790.35	404.19	7
3	284.12	266.11	267.10	142.56	A	710.33	692.31	693.30	355.66	6
4	431.19	413.18	414.17	216.10	F	639.29	621.28	622.26	320.14	5
5	545.24	527.23	528.21	273.13	N	492.22	474.21	475.19	246.61	4
6	632.27	614.26	615.25	316.63	S	378.18	360.17	361.15	189.59	3
7	818.35	800.34	801.32	409.67	W	291.15	273.13	274.12	146.07	2
8					S(-.98)	105.07	87.06	88.04	53.03	1

Kinin-15 (K-15)

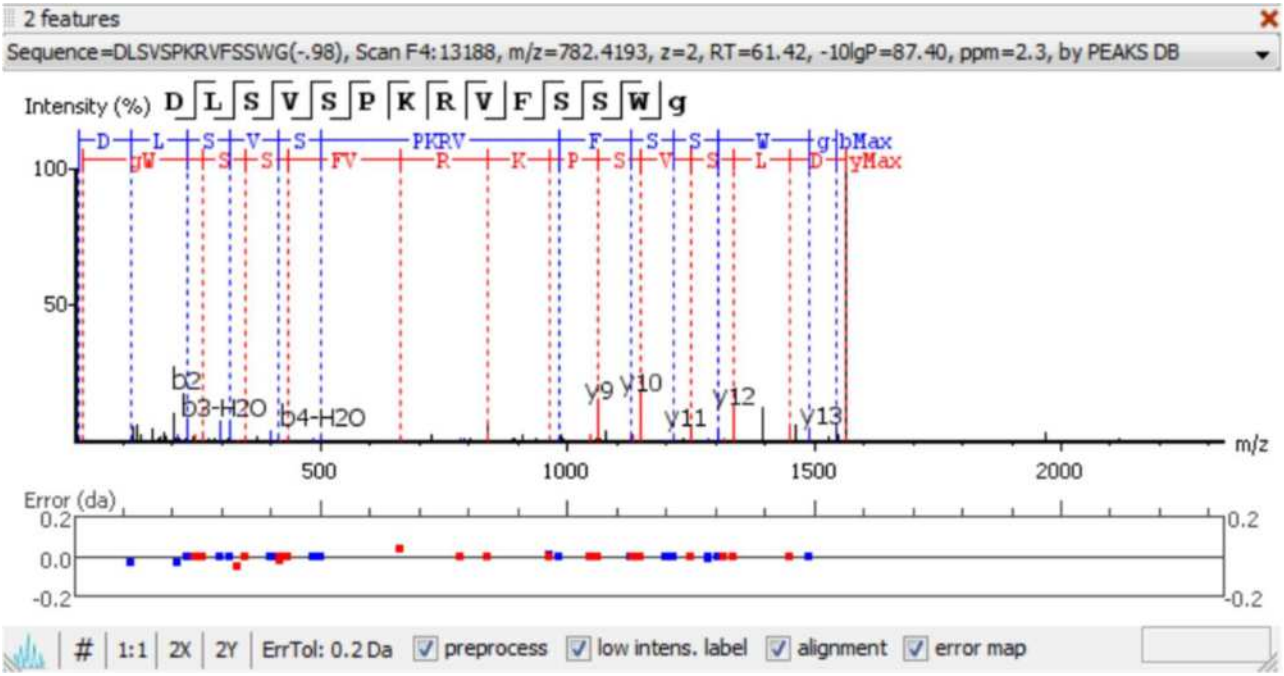

#	b	b-H2O	b-NH3	b (2+)	Seq	y	y-H2O	y-NH3	y (2+)	#
1	116.07	98.02	99.01	58.52	D					14
2	229.12	211.14	212.09	115.06	L	1448.80	1430.79	1431.77	724.90	13
3	316.15	298.14	299.12	158.58	S	1335.72	1317.70	1318.69	668.36	12
4	415.22	397.21	398.19	208.11	V	1248.69	1230.67	1231.66	624.84	11
5	502.25	484.24	485.22	251.63	S	1149.62	1131.60	1132.59	575.31	10
6	599.30	581.29	582.28	300.15	P	1062.59	1044.58	1045.56	531.79	9
7	727.40	709.39	710.37	364.20	K	965.53	947.52	948.50	483.27	8
8	883.50	865.49	866.47	442.25	R	837.43	819.43	820.41	419.24	7
9	982.57	964.56	965.53	491.78	V	681.34	663.28	664.31	341.17	6
10	1129.64	1111.63	1112.61	565.32	F	582.27	564.26	565.24	291.63	5
11	1216.67	1198.66	1199.64	608.83	S	435.20	417.19	418.17	218.10	4
12	1303.70	1285.70	1286.69	652.35	S	348.17	330.21	331.14	174.58	3
13	1489.78	1471.77	1472.75	745.39	W	261.13	243.12	244.11	131.07	2
14					G(-.98)	75.06	57.04	58.03	38.03	1

Kinin-17 (K-17)_partial

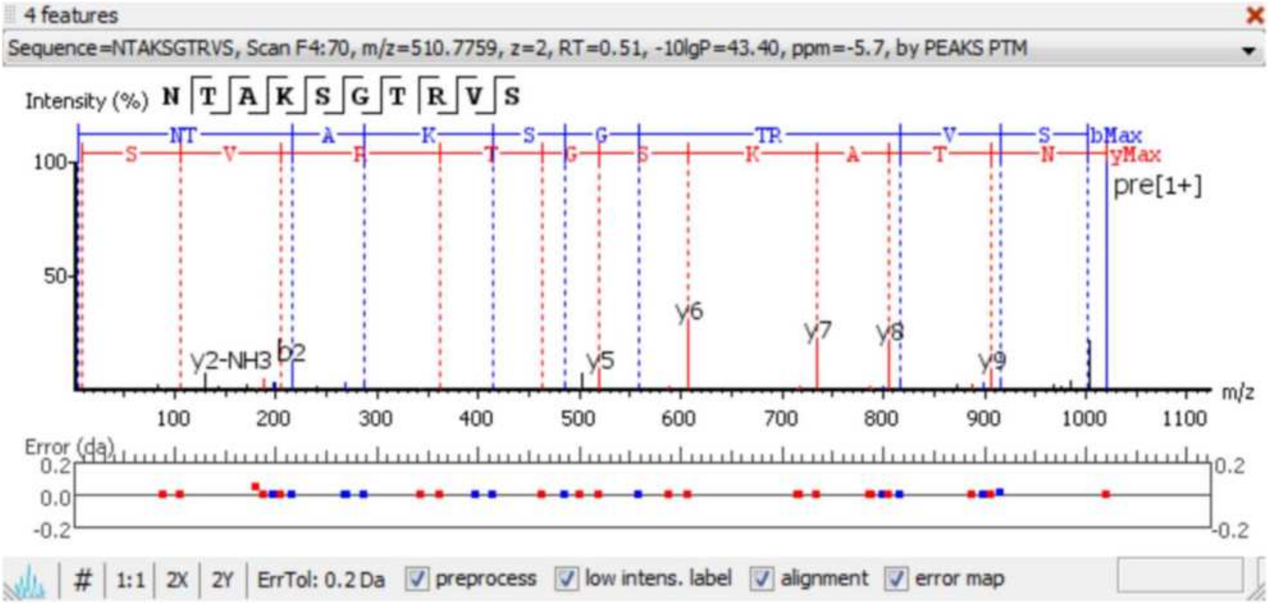

#	b	b-H2O	b-NH3	b (2+)	Seq	y	y-H2O	y-NH3	y (2+)	#
1	115.05	97.04	98.02	58.03	N					10
2	216.10	198.09	199.07	108.55	T	906.50	888.49	889.47	453.75	9
3	287.14	269.16	270.11	144.07	A	805.45	787.44	788.43	403.23	8
4	415.21	397.22	398.20	208.12	K	734.42	716.40	717.39	367.71	7
5	502.26	484.25	485.24	251.63	S	606.32	588.31	589.29	303.66	6
6	559.28	541.45	542.30	280.14	G	519.29	501.28	502.26	260.14	5
7	660.33	642.32	643.35	330.67	T	462.27	444.26	445.24	231.63	4
8	816.43	798.42	799.41	408.72	R	361.22	343.21	344.19	181.10	3
9	915.50	897.49	898.47	458.25	V	205.12	187.11	188.10	103.06	2
10					S	106.05	88.04	89.02	53.52	1

Kinin-PP-2 (K-PP-2)

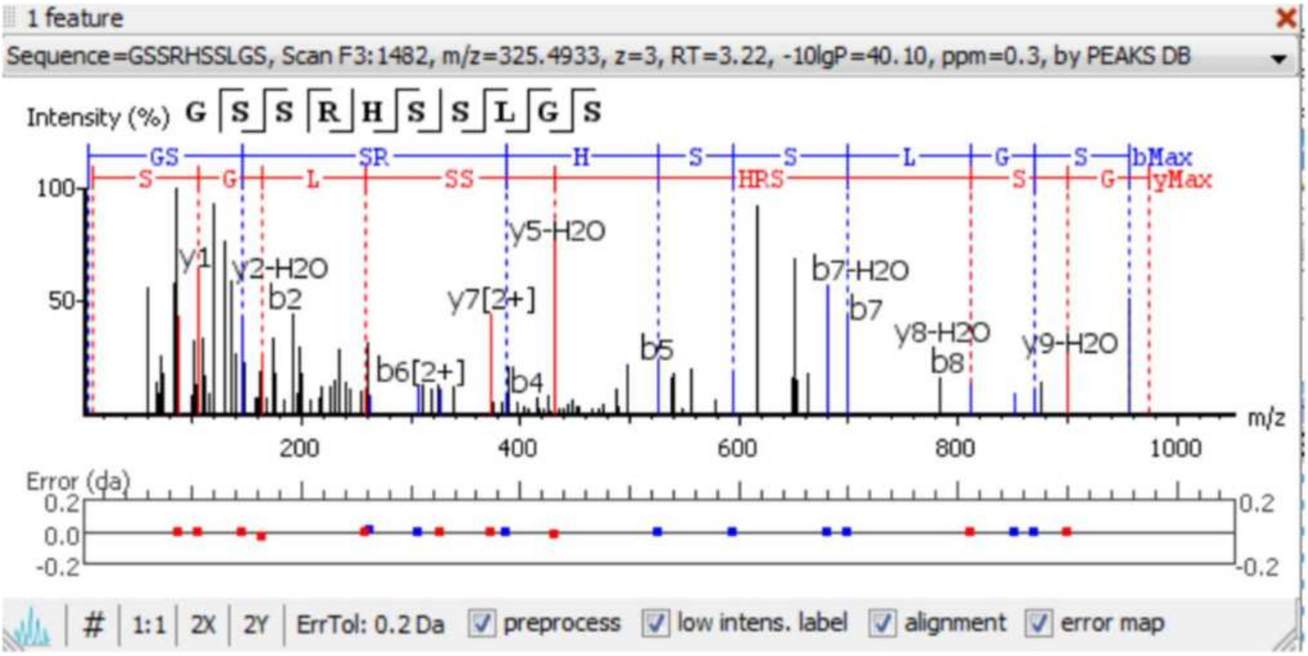

#	b	b-H2O	b-NH3	b (2+)	Seq	y	y-H2O	y-NH3	y (2+)	#
1	58.03	40.02	41.00	29.51	G					10
2	145.06	127.05	128.03	73.03	S	917.44	899.43	900.42	459.22	9
3	232.09	214.08	215.07	116.55	S	830.41	812.40	813.38	415.71	8
4	388.19	370.18	371.17	194.60	R	743.38	725.37	726.35	372.19	7
5	525.25	507.24	508.23	263.10	H	587.28	569.27	570.25	294.14	6
6	612.29	594.27	595.26	306.65	S	450.22	432.23	433.19	225.61	5
7	699.32	681.31	682.29	350.16	S	363.19	345.18	346.16	182.09	4
8	812.40	794.39	795.37	406.70	L	276.16	258.14	259.12	138.58	3
9	869.42	851.41	852.40	435.21	G	163.11	145.06	146.04	82.04	2
10					S	106.05	88.04	89.02	53.52	1

Kinin-18 (K-18)

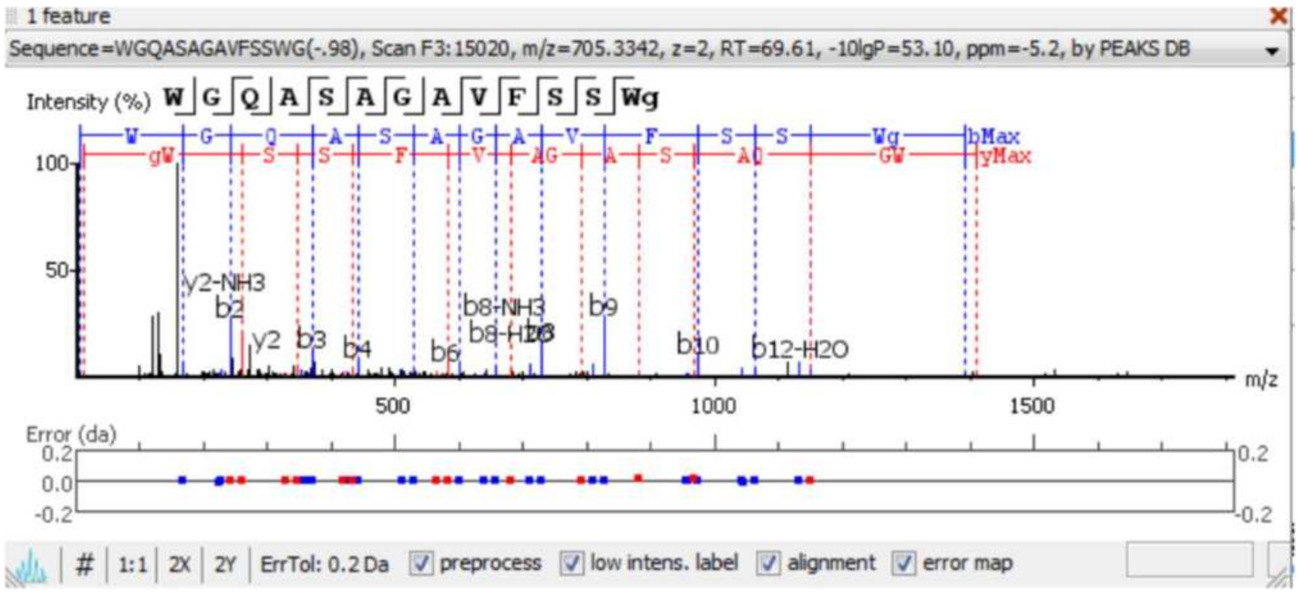

#	b	b-H2O	b-NH3	b (2+)	Seq	y	y-H2O	y-NH3	y (2+)	#
1	187.09	169.08	170.06	94.04	W					14
2	244.11	226.12	227.08	122.55	G	1223.58	1205.57	1206.55	612.29	13
3	372.17	354.16	355.14	186.58	Q	1166.56	1148.55	1149.53	583.78	12
4	443.20	425.19	426.18	222.10	A	1038.50	1020.49	1021.47	519.75	11
5	530.24	512.23	513.21	265.62	S	967.45	949.45	950.44	484.23	10
6	601.27	583.26	584.25	301.14	A	880.41	862.42	863.40	440.72	9
7	658.29	640.28	641.26	329.65	G	809.39	791.38	792.38	405.20	8
8	729.33	711.32	712.31	365.17	A	752.37	734.36	735.35	376.69	7
9	828.40	810.39	811.38	414.70	V	681.34	663.32	664.31	341.17	6
10	975.47	957.46	958.45	488.23	F	582.26	564.26	565.24	291.63	5
11	1062.50	1044.49	1045.49	531.75	S	435.20	417.19	418.17	218.10	4
12	1149.53	1131.52	1132.51	575.27	S	348.17	330.16	331.14	174.58	3
13	1335.61	1317.60	1318.59	668.31	W	261.13	243.12	244.11	131.07	2
14					G(-.98)	75.06	57.04	58.03	38.03	1

Kinin-19 (K-19)

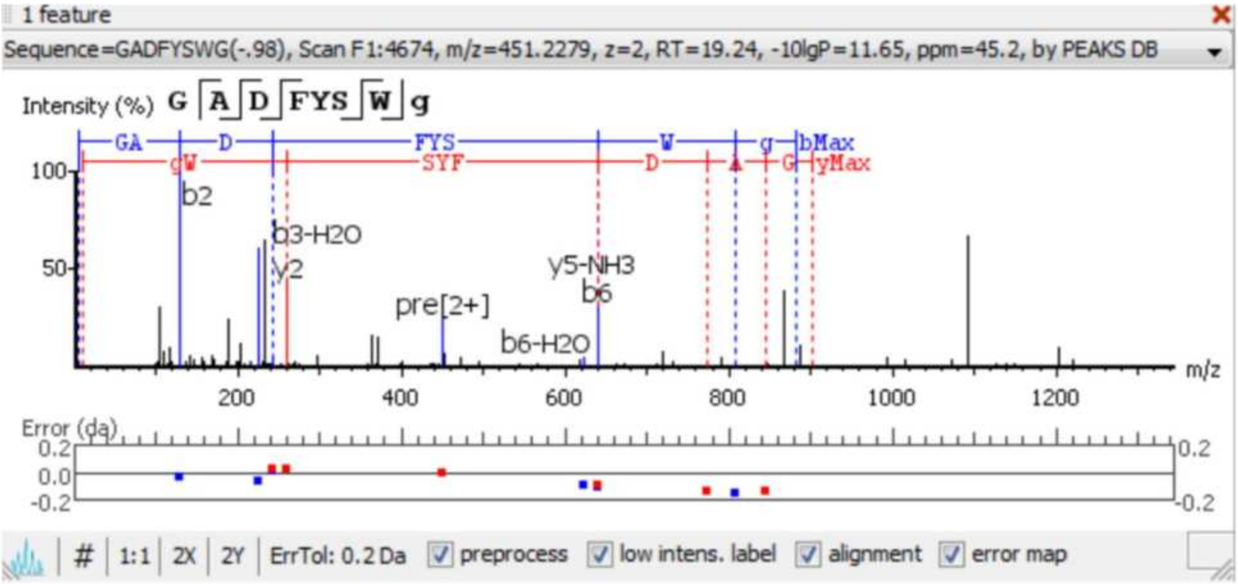

#	b	b-H2O	b-NH3	b (2+)	Seq	y	y-H2O	y-NH3	y (2+)	#
1	58.03	40.02	41.00	29.51	G					8
2	129.10	111.06	112.04	65.03	A	844.50	826.35	827.34	422.68	7
3	244.06	226.15	227.07	122.55	D	773.46	755.31	756.30	387.16	6
4	391.16	373.15	374.13	196.08	F	658.30	640.29	641.36	329.65	5
5	554.23	536.21	537.20	277.61	Y	511.23	493.22	494.20	256.11	4
6	641.36	623.35	624.23	321.13	S	348.17	330.16	331.14	174.58	3
7	827.34	809.49	810.31	414.17	W	261.09	243.08	244.06	131.07	2
8					G(-.98)	75.06	57.04	58.03	38.03	1

Kinin-19 (K-19)

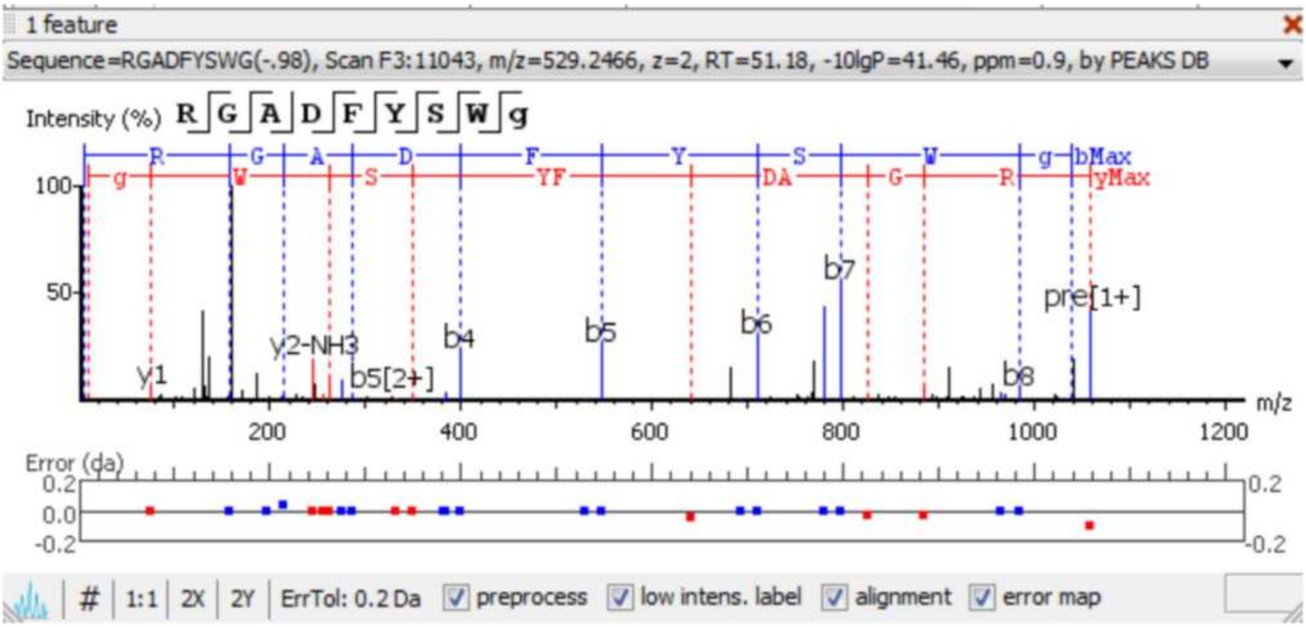

#	b	b-H2O	b-NH3	b (2+)	Seq	y	y-H2O	y-NH3	y (2+)	#
1	157.11	139.10	140.08	79.05	R					9
2	214.08	196.12	197.10	107.57	G	901.38	883.41	884.36	451.19	8
3	285.17	267.16	268.14	143.08	A	844.36	826.38	827.34	422.68	7
4	400.19	382.18	383.17	200.60	D	773.33	755.31	756.30	387.16	6
5	547.26	529.25	530.24	274.12	F	658.30	640.34	641.27	329.65	5
6	710.32	692.31	693.30	355.66	Y	511.23	493.22	494.20	256.11	4
7	797.36	779.35	780.33	399.18	S	348.17	330.16	331.14	174.58	3
8	983.44	965.42	966.41	492.22	W	261.13	243.12	244.11	131.07	2
9					G(-.98)	75.06	57.04	58.03	38.03	1

Myosuppressin (MS)_Q

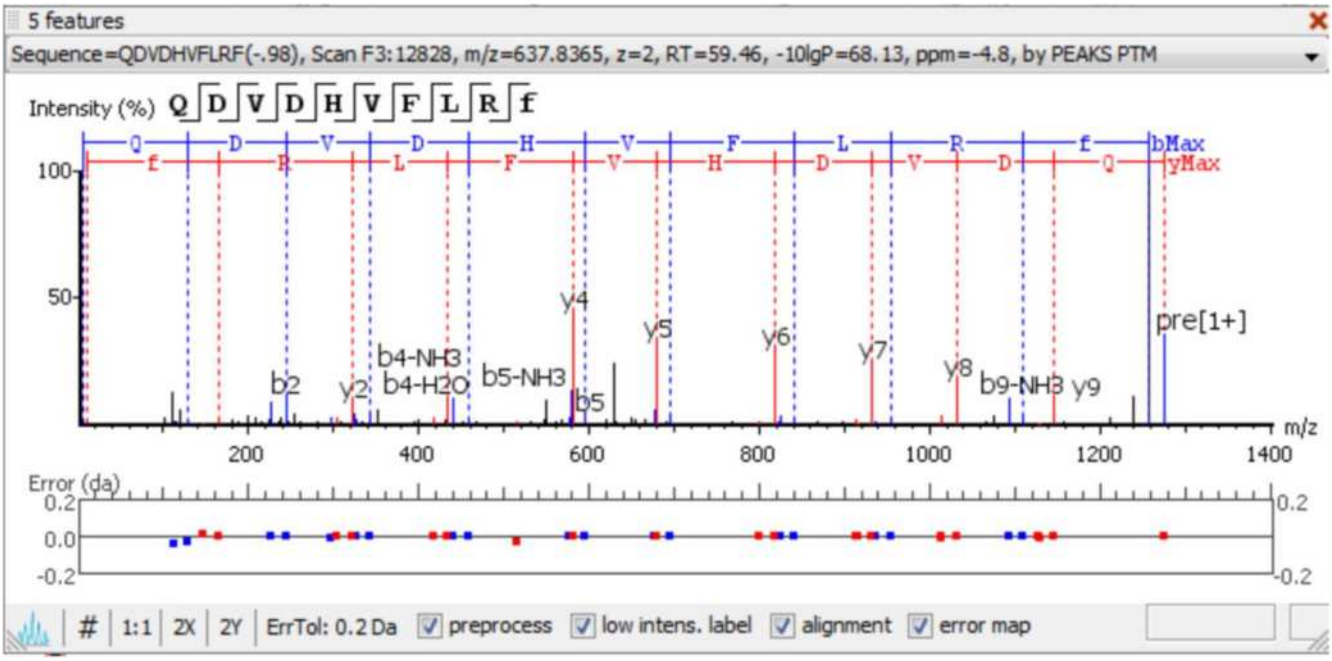

#	b	b-H2O	b-NH3	b (2+)	Seq	y	y-H2O	y-NH3	y (2+)	#
1	129.10	111.06	112.09	65.03	Q					10
2	244.09	226.08	227.07	122.55	D	1146.61	1128.59	1129.59	573.80	9
3	343.16	325.15	326.13	172.08	V	1031.58	1013.56	1014.56	516.33	8
4	458.19	440.18	441.16	229.59	D	932.51	914.50	915.49	466.76	7
5	595.25	577.24	578.22	298.14	H	817.48	799.47	800.46	409.24	6
6	694.32	676.30	677.29	347.66	V	680.42	662.41	663.40	340.71	5
7	841.38	823.37	824.36	421.19	F	581.36	563.35	564.33	291.18	4
8	954.47	936.46	937.45	477.73	L	434.29	416.28	417.26	217.64	3
9	1110.57	1092.56	1093.55	555.78	R	321.20	303.19	304.18	161.10	2
10					F(-.98)	165.10	147.08	148.08	83.05	1

Myosuppressin (MS)_pQ

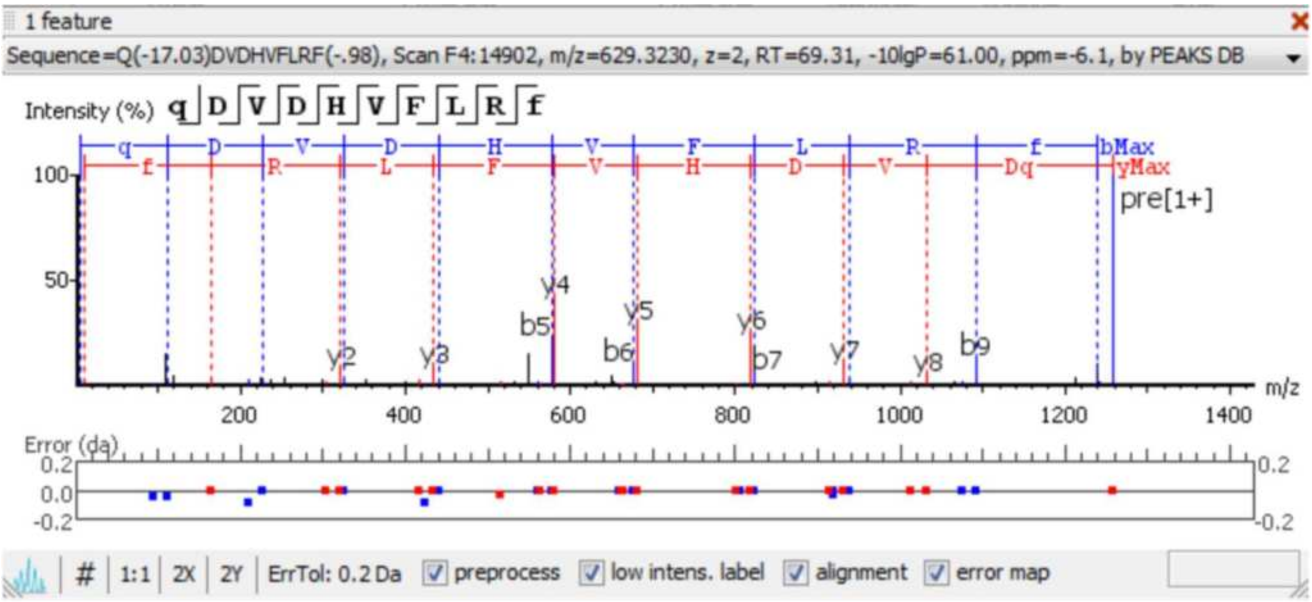

#	b	b-H2O	b-NH3	b (2+)	Seq	y	y-H2O	y-NH3	y (2+)	#
1	112.09	94.03	95.06	56.52	Q(-17.03)					10
2	227.07	209.14	210.04	114.03	D	1146.61	1128.59	1129.58	573.80	9
3	326.13	308.12	309.11	163.57	V	1031.58	1013.57	1014.55	516.33	8
4	441.16	423.24	424.14	221.08	D	932.51	914.50	915.49	466.76	7
5	578.22	560.21	561.19	289.61	H	817.48	799.47	800.46	409.24	6
6	677.29	659.28	660.26	339.14	V	680.42	662.41	663.40	340.71	5
7	824.36	806.35	807.33	412.68	F	581.35	563.35	564.33	291.18	4
8	937.44	919.43	920.45	469.22	L	434.29	416.28	417.26	217.64	3
9	1093.54	1075.53	1076.52	547.27	R	321.20	303.19	304.18	161.10	2
10					F(-.98)	165.10	147.09	148.08	83.05	1

Myosuppressin (MS)-PP_partial_part1

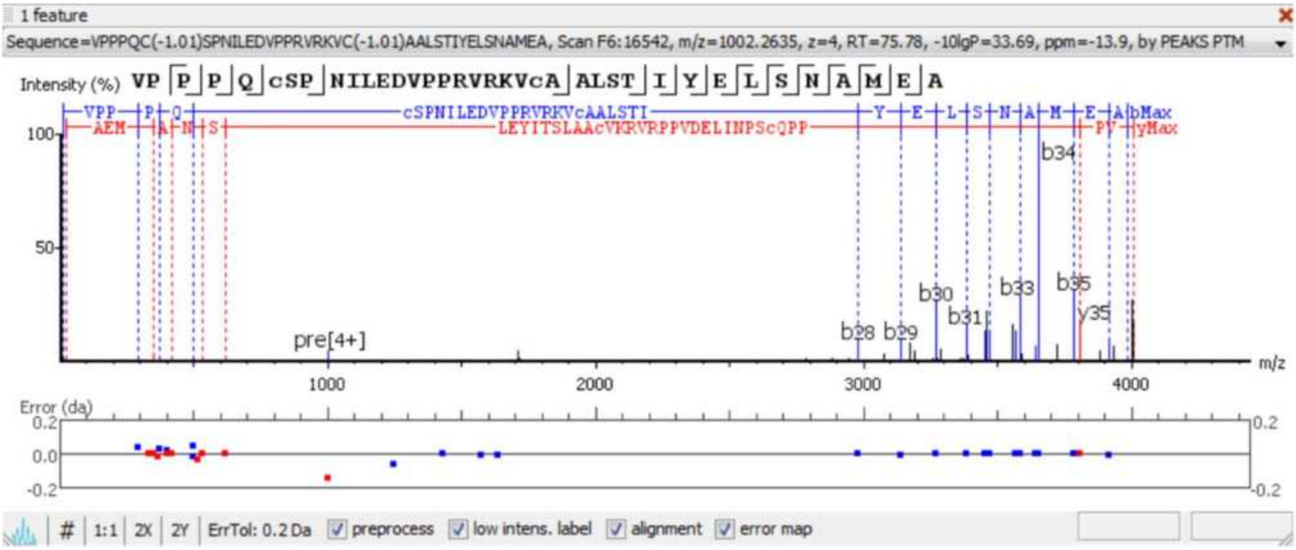

#	b	b-H2O	b-NH3	b (2+)	Seq	y	y-H2O	y-NH3	y (2+)	#
1	100.08	82.07	83.05	50.54	V					37
2	197.13	179.12	180.10	99.06	P	3906.96	3888.94	3889.93	1953.98	36
3	294.14	276.17	277.15	147.59	P	3809.90	3791.89	3792.88	1905.45	35
4	391.23	373.19	374.21	196.12	P	3712.85	3694.84	3695.82	1856.92	34
5	519.29	501.31	502.22	260.15	Q	3615.80	3597.79	3598.77	1808.40	33
6	621.29	603.28	604.27	311.15	C(-1.01)	3487.74	3469.73	3470.71	1744.37	32
7	708.33	690.32	691.30	354.66	S	3385.74	3367.73	3368.71	1693.37	31
8	805.38	787.37	788.35	403.17	P	3298.70	3280.69	3281.68	1649.85	30
9	919.42	901.41	902.40	460.21	N	3201.65	3183.64	3184.62	1601.33	29
10	1032.51	1014.50	1015.48	516.75	I	3087.61	3069.60	3070.58	1544.30	28
11	1145.59	1127.58	1128.56	573.30	L	2974.52	2956.51	2957.50	1487.76	27
12	1274.63	1256.62	1257.61	637.82	E	2861.44	2843.43	2844.41	1431.22	26
13	1389.66	1371.65	1372.63	695.33	D	2732.40	2714.39	2715.37	1366.70	25
14	1488.73	1470.72	1471.70	744.86	V	2617.37	2599.36	2600.34	1309.19	24
15	1585.78	1567.77	1568.75	793.39	P	2518.30	2500.29	2501.28	1259.65	23
16	1682.83	1664.82	1665.81	841.92	P	2421.25	2403.24	2404.22	1211.13	22
17	1838.93	1820.92	1821.91	919.97	R	2324.20	2306.19	2307.17	1162.60	21
18	1938.00	1919.99	1920.98	969.50	V	2168.10	2150.09	2151.07	1084.55	20
19	2094.10	2076.09	2077.08	1047.55	R	2069.03	2051.02	2052.00	1035.01	19
20	2222.20	2204.19	2205.17	1111.60	K	1912.93	1894.92	1895.90	956.96	18
21	2321.27	2303.26	2304.24	1161.13	V	1784.83	1766.82	1767.80	892.92	17
22	2423.27	2405.26	2406.24	1212.13	C(-1.01)	1685.76	1667.75	1668.74	843.38	16
23	2494.31	2476.30	2477.28	1247.72	A	1583.76	1565.75	1566.74	792.38	15
24	2565.34	2547.33	2548.32	1283.17	A	1512.72	1494.71	1495.70	756.86	14
25	2678.43	2660.42	2661.40	1339.71	L	1441.69	1423.68	1424.66	721.34	13
26	2765.46	2747.45	2748.43	1383.23	S	1328.60	1310.59	1311.58	664.80	12
27	2866.51	2848.50	2849.48	1433.76	T	1241.57	1223.56	1224.54	621.29	11
28	2979.59	2961.58	2962.56	1490.30	I	1140.52	1122.51	1123.50	570.76	10
29	3142.67	3124.64	3125.63	1571.84	Y	1027.44	1009.43	1010.41	514.22	9
30	3271.70	3253.69	3254.67	1636.36	E	864.38	846.37	847.35	432.69	8
31	3384.79	3366.77	3367.75	1692.89	L	735.33	717.32	718.31	368.19	7
32	3471.82	3453.80	3454.79	1736.41	S	622.25	604.24	605.22	311.63	6
33	3585.86	3567.84	3568.83	1793.43	N	535.22	517.24	518.19	268.11	5
34	3656.90	3638.88	3639.87	1828.95	A	421.18	403.17	404.15	211.09	4
35	3787.94	3769.92	3770.91	1894.47	M	350.14	332.13	333.11	175.57	3
36	3916.99	3898.97	3899.95	1958.99	E	219.10	201.09	202.07	110.05	2
37					A	90.05	72.04	73.03	45.53	1

Myosuppressin (MS)-PP_partial_part2

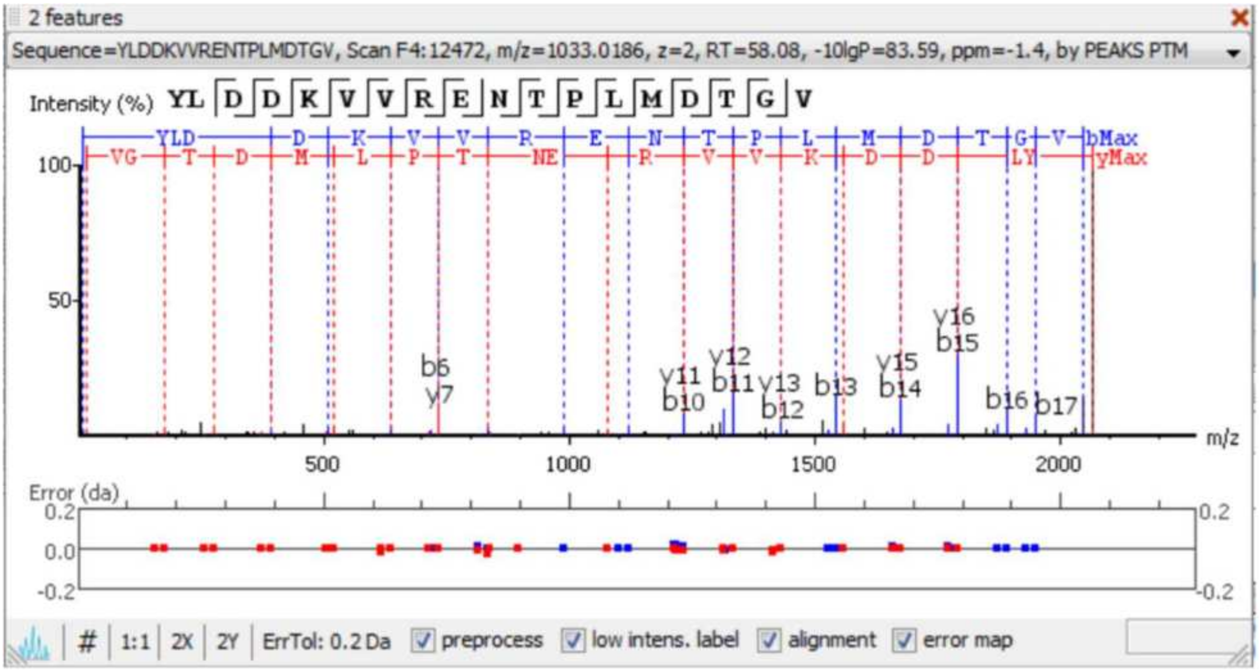

#	b	b-H2O	b-NH3	b (2+)	Seq	y	y-H2O	y-NH3	y (2+)	#
1	164.07	146.06	147.04	82.54	Y					18
2	277.16	259.14	260.13	139.08	L	1901.96	1883.95	1884.94	951.48	17
3	392.18	374.17	375.16	196.59	D	1788.88	1770.87	1771.85	894.95	16
4	507.21	489.20	490.18	254.10	D	1673.85	1655.84	1656.83	837.43	15
5	635.30	617.29	618.28	318.15	K	1558.83	1540.82	1541.80	779.91	14
6	734.37	716.36	717.35	367.69	V	1430.73	1412.74	1413.73	715.87	13
7	833.44	815.41	816.41	417.22	V	1331.66	1313.66	1314.65	666.33	12
8	989.54	971.53	972.52	495.27	R	1232.61	1214.59	1215.58	616.82	11
9	1118.59	1100.57	1101.56	559.79	E	1076.50	1058.48	1059.47	538.75	10
10	1232.61	1214.59	1215.58	616.82	N	947.45	929.44	930.42	474.23	9
11	1333.67	1315.67	1316.67	667.34	T	833.44	815.41	816.38	417.20	8
12	1430.73	1412.74	1413.73	715.87	P	732.36	714.35	715.33	366.68	7
13	1543.81	1525.79	1526.79	772.41	L	635.30	617.29	618.28	318.15	6
14	1674.86	1656.83	1657.83	837.93	M	522.22	504.21	505.20	261.61	5
15	1789.88	1771.85	1772.86	895.44	D	391.18	373.17	374.16	196.09	4
16	1890.93	1872.91	1873.90	945.96	T	276.16	258.14	259.13	138.58	3
17	1947.95	1929.94	1930.92	974.47	G	175.11	157.10	158.08	88.05	2
18					V	118.09	100.08	101.06	59.54	1

Natalisin (Nat)/WAARamide-1 (Nat-1)_partial

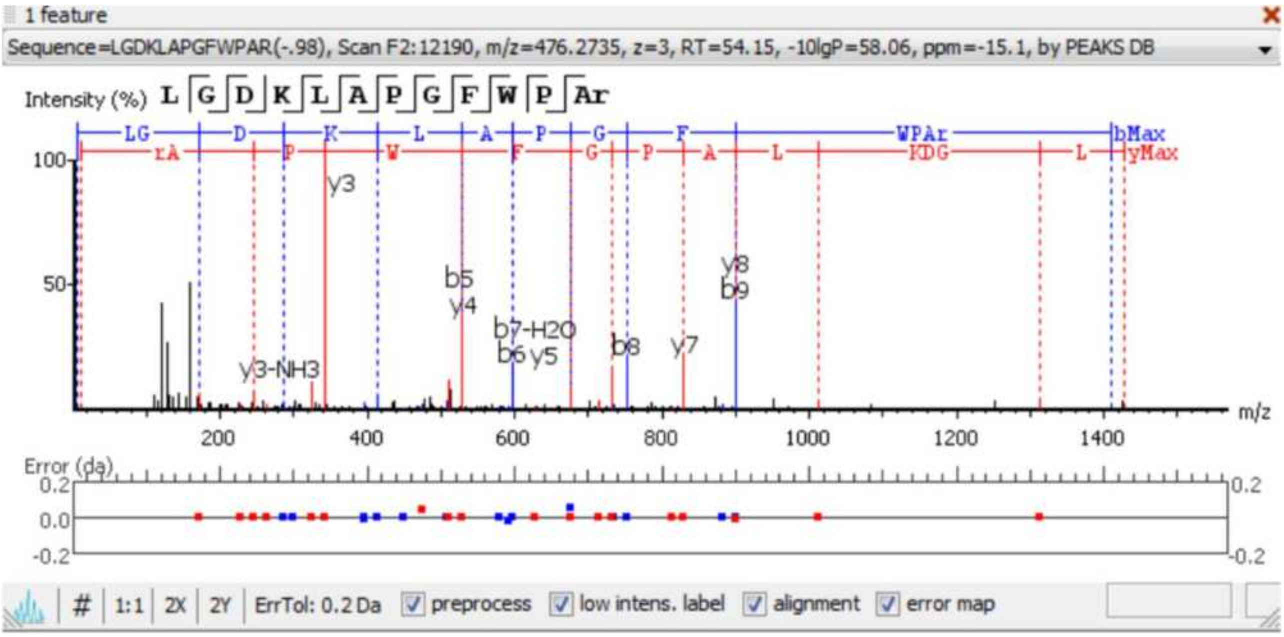

#	b	b-H2O	b-NH3	b (2+)	Seq	y	y-H2O	y-NH3	y (2+)	#
1	114.09	96.08	97.06	57.55	L					13
2	171.11	153.10	154.09	86.06	G	1313.71	1295.70	1296.68	657.36	12
3	286.14	268.13	269.11	143.57	D	1256.69	1238.68	1239.66	628.85	11
4	414.23	396.22	397.23	207.62	K	1141.66	1123.65	1124.64	571.33	10
5	527.32	509.31	510.30	264.16	L	1013.57	995.56	996.54	507.28	9
6	598.36	580.35	581.33	299.69	A	900.49	882.47	883.46	450.74	8
7	695.41	677.35	678.38	348.20	P	829.45	811.44	812.42	415.22	7
8	752.43	734.41	735.40	376.72	G	732.39	714.38	715.37	366.70	6
9	899.50	881.49	882.47	450.25	F	675.37	657.36	658.35	338.19	5
10	1085.58	1067.57	1068.55	543.29	W	528.30	510.30	511.28	264.66	4
11	1182.63	1164.62	1165.60	591.84	P	342.22	324.21	325.20	171.62	3
12	1253.67	1235.66	1236.64	627.33	A	245.17	227.16	228.15	123.09	2
13					R(-.98)	174.13	156.12	157.11	87.57	1

Natalisin (Nat)/WAARamide-6 (Nat-6)

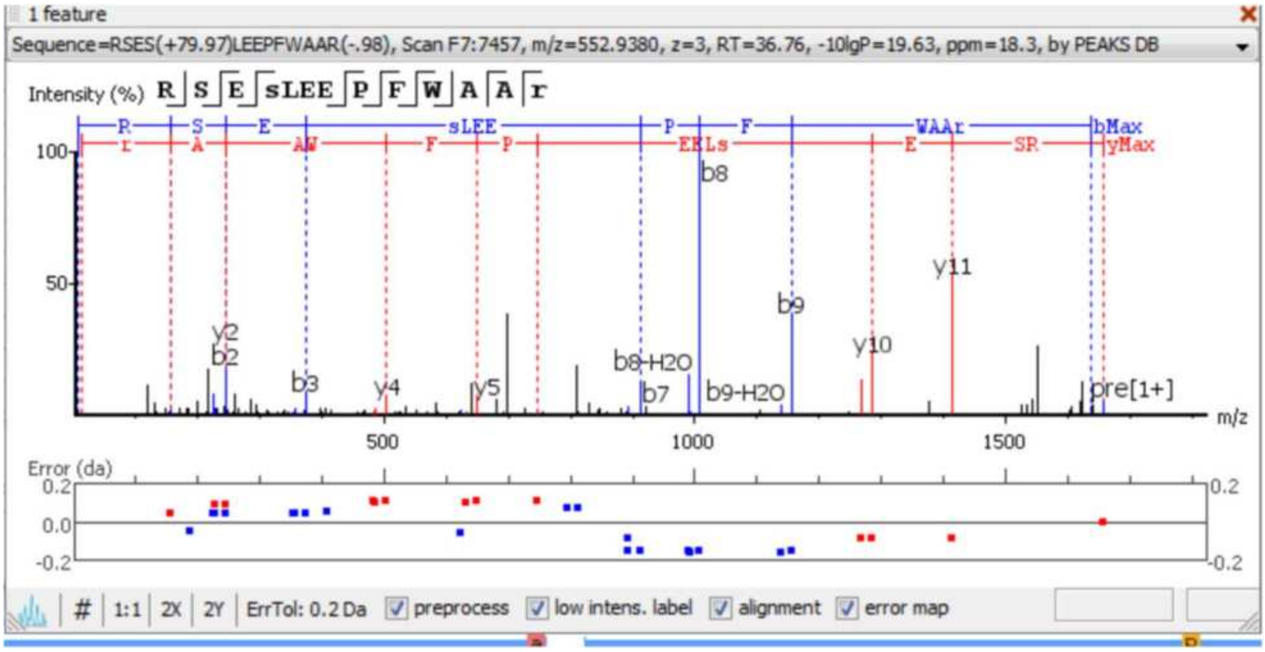

#	b	b-H2O	b-NH3	b (2+)	Seq	y	y-H2O	y-NH3	y (2+)	#
1	157.06	139.10	140.08	79.05	R					13
2	244.09	226.08	227.07	122.57	S	1500.65	1482.64	1483.62	750.83	12
3	373.13	355.12	356.11	187.14	E	1413.71	1395.61	1396.59	707.31	11
4	540.18	522.17	523.15	270.59	S(+79.97)	1284.67	1266.66	1267.55	642.79	10
5	653.27	635.26	636.24	327.13	L	1117.58	1099.57	1100.55	559.29	9
6	782.31	764.30	765.28	391.65	E	1004.49	986.48	987.47	502.75	8
7	911.50	893.49	894.32	456.18	E	875.45	857.44	858.43	438.23	7
8	1008.56	990.55	991.54	504.70	P	746.30	728.40	729.38	373.70	6
9	1155.63	1137.62	1138.45	578.24	F	649.24	631.24	632.22	325.18	5
10	1341.55	1323.54	1324.52	671.28	W	502.18	484.17	485.15	251.64	4
11	1412.59	1394.58	1395.56	706.79	A	316.21	298.20	299.18	158.60	3
12	1483.63	1465.62	1466.60	742.31	A	245.08	227.07	228.15	123.09	2
13					R(-.98)	174.13	156.12	157.06	87.57	1

Natalisin (Nat)/WAARamide-7 (Nat-7)

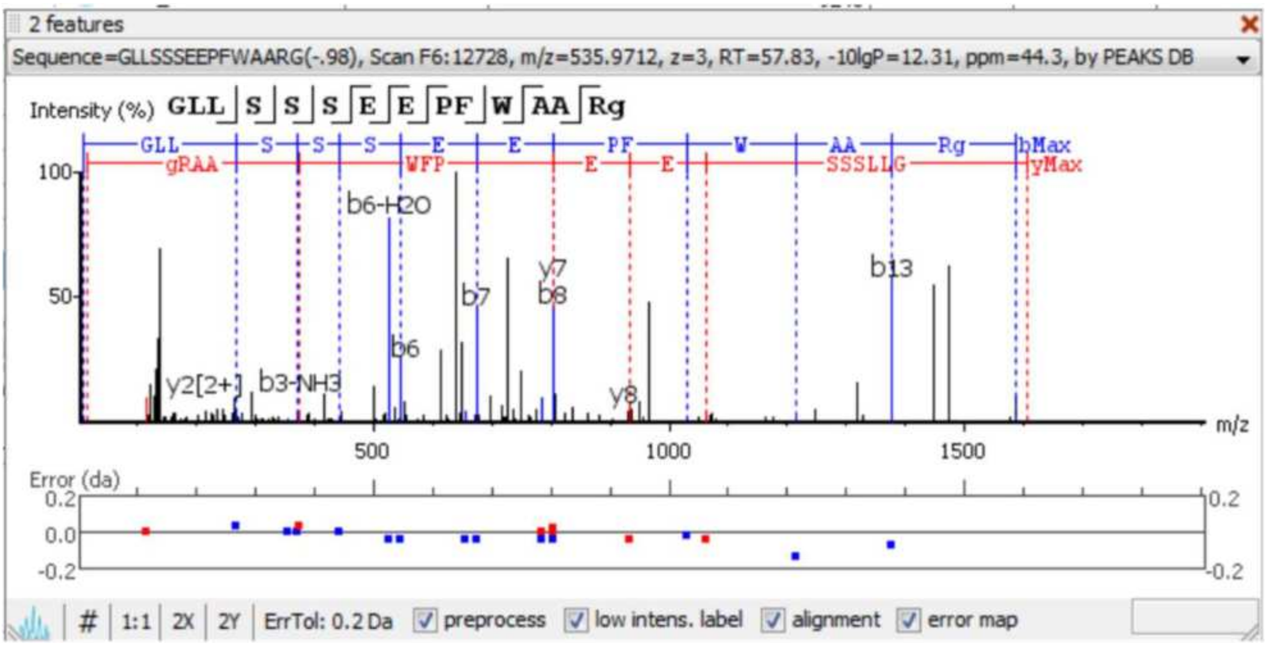

#	b	b-H2O	b-NH3	b (2+)	Seq	y	y-H2O	y-NH3	y (2+)	#
1	58.03	40.02	41.00	29.51	G					15
2	171.11	153.10	154.09	86.06	L	1548.78	1530.77	1531.75	774.89	14
3	284.20	266.19	267.13	142.60	L	1435.70	1417.69	1418.67	718.35	13
4	371.23	353.22	354.20	186.11	S	1322.61	1304.60	1305.59	661.81	12
5	458.26	440.25	441.23	229.63	S	1235.58	1217.57	1218.55	618.29	11
6	545.34	527.33	528.27	273.15	S	1148.55	1130.54	1131.52	574.77	10
7	674.38	656.37	657.31	337.67	E	1061.56	1043.51	1044.49	531.26	9
8	803.43	785.42	786.35	402.19	E	932.52	914.46	915.45	466.74	8
9	900.43	882.42	883.40	450.72	P	803.43	785.42	786.40	402.22	7
10	1047.50	1029.49	1030.50	524.25	F	706.38	688.37	689.35	353.69	6
11	1233.58	1215.57	1216.70	617.29	W	559.31	541.30	542.28	280.15	5
12	1304.62	1286.61	1287.59	652.81	A	373.19	355.22	356.20	187.12	4
13	1375.73	1357.64	1358.63	688.33	A	302.19	284.18	285.17	151.60	3
14	1531.75	1513.74	1514.73	766.38	R	231.16	213.15	214.13	116.07	2
15					G(-.98)	75.06	57.04	58.03	38.03	1

Natalisin (Nat)/WAARamide-10 (Nat-10)

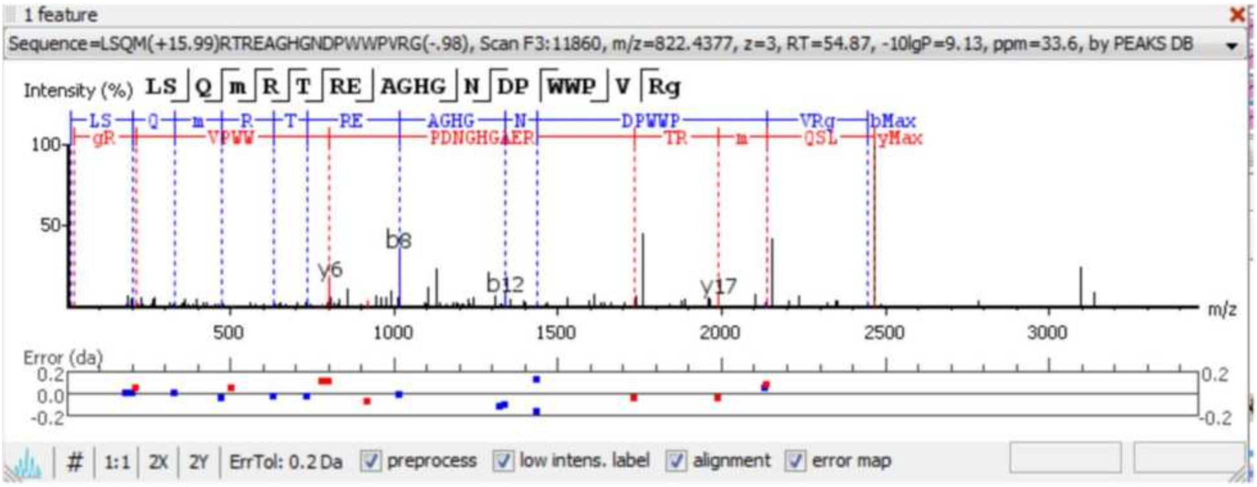

#	b	b-H2O	b-NH3	b (2+)	Seq	y	y-H2O	y-NH3	y (2+)	#
1	114.09	96.08	97.06	57.55	L					21
2	201.12	183.11	184.10	101.06	S	2352.12	2334.11	2335.09	1176.56	20
3	329.18	311.17	312.16	165.09	Q	2265.08	2247.07	2248.06	1133.04	19
4	476.27	458.21	459.19	238.61	M(+15.99)	2136.94	2119.02	2120.00	1069.01	18
5	632.35	614.31	615.29	316.66	R	1990.04	1971.98	1972.96	995.50	17
6	733.41	715.36	716.34	367.18	T	1833.89	1815.88	1816.86	917.53	16
7	889.47	871.46	872.44	445.23	R	1732.90	1714.83	1715.81	866.92	15
8	1018.53	1000.50	1001.48	509.76	E	1576.74	1558.73	1559.71	788.87	14
9	1089.55	1071.54	1072.52	545.27	A	1447.70	1429.69	1430.67	724.35	13
10	1146.57	1128.56	1129.54	573.78	G	1376.66	1358.65	1359.63	688.83	12
11	1283.63	1265.62	1266.60	642.31	H	1319.64	1301.63	1302.61	660.32	11
12	1340.77	1322.76	1323.62	670.82	G	1182.58	1164.57	1165.55	591.79	10
13	1454.69	1436.55	1437.84	727.85	N	1125.56	1107.55	1108.53	563.28	9
14	1569.72	1551.71	1552.69	785.36	D	1011.52	993.51	994.49	506.20	8
15	1666.77	1648.76	1649.74	833.89	P	896.49	878.48	879.46	448.74	7
16	1852.85	1834.84	1835.82	926.93	W	799.32	781.32	782.41	400.22	6
17	2038.93	2020.92	2021.90	1019.97	W	613.36	595.35	596.33	307.18	5
18	2135.93	2117.97	2118.96	1068.49	P	427.28	409.27	410.25	214.14	4
19	2235.05	2217.04	2218.02	1118.03	V	330.22	312.21	313.20	165.61	3
20	2391.15	2373.14	2374.13	1196.08	R	231.16	213.09	214.13	116.08	2
21					G(-.98)	75.06	57.04	58.03	38.03	1

long neuropeptide F-1a (NPF-1a)

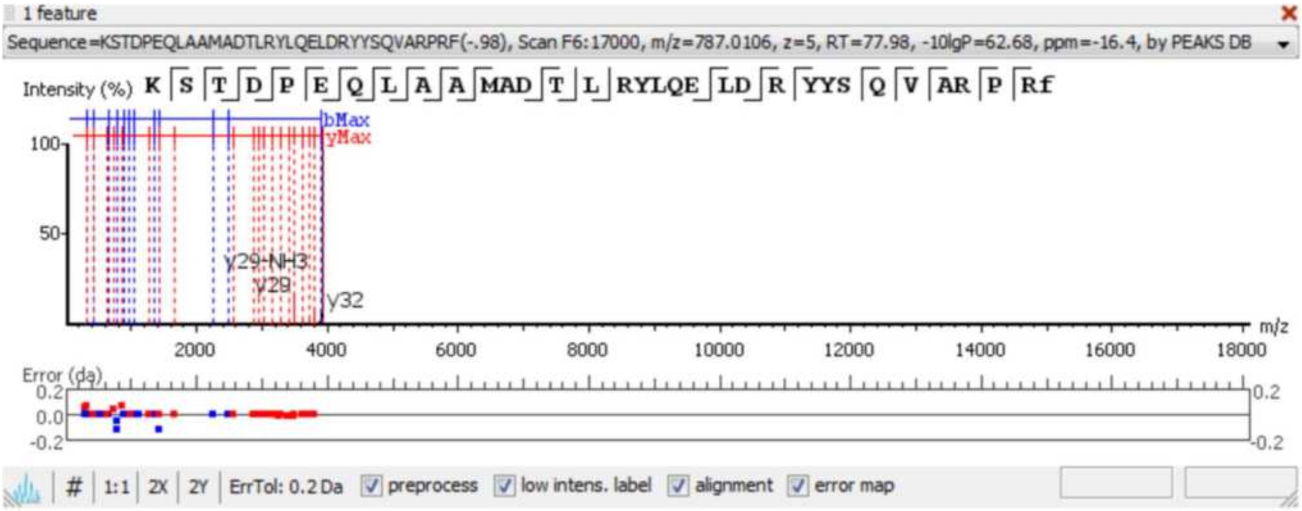

#	b	b-H2O	b-NH3	b (2+)	Seq	y	y-H2O	y-NH3	y (2+)	#
1	129.10	111.09	112.08	65.05	K					33
2	216.13	198.12	199.11	108.57	S	3802.90	3784.87	3785.87	1901.95	32
3	317.18	299.17	300.16	159.09	T	3715.87	3697.85	3698.84	1858.43	31
4	432.21	414.20	415.18	216.60	D	3614.82	3596.81	3597.79	1807.91	30
5	529.26	511.25	512.24	265.13	P	3499.80	3481.78	3482.79	1750.40	29
6	658.31	640.29	641.28	329.65	E	3402.76	3384.73	3385.73	1701.87	28
7	786.43	768.35	769.34	393.69	Q	3273.70	3255.69	3256.69	1637.35	27
8	899.45	881.44	882.42	450.23	L	3145.65	3127.63	3128.62	1573.32	26
9	970.49	952.47	953.46	485.74	A	3032.56	3014.54	3015.52	1516.78	25
10	1041.52	1023.51	1024.49	521.26	A	2961.53	2943.49	2944.49	1481.26	24
11	1172.56	1154.55	1155.54	586.78	M	2890.48	2872.47	2873.46	1445.74	23
12	1243.60	1225.59	1226.57	622.30	A	2759.44	2741.43	2742.41	1380.22	22
13	1358.63	1340.62	1341.60	679.81	D	2688.40	2670.39	2671.37	1344.70	21
14	1459.67	1441.78	1442.65	730.34	T	2573.38	2555.36	2556.35	1287.19	20
15	1572.76	1554.75	1555.73	787.00	L	2472.33	2454.32	2455.30	1236.66	19
16	1728.86	1710.85	1711.83	864.93	R	2359.24	2341.23	2342.21	1180.12	18
17	1891.92	1873.91	1874.90	946.46	Y	2203.14	2185.13	2186.11	1102.07	17
18	2005.01	1987.00	1987.98	1003.00	L	2040.08	2022.07	2023.05	1020.54	16
19	2133.06	2115.05	2116.04	1067.03	Q	1926.99	1908.98	1909.97	964.00	15
20	2262.12	2244.10	2245.08	1131.56	E	1798.93	1780.92	1781.91	899.97	14
21	2375.19	2357.18	2358.16	1188.10	L	1669.89	1651.88	1652.87	835.45	13
22	2490.23	2472.21	2473.19	1245.61	D	1556.81	1538.80	1539.78	778.90	12
23	2646.32	2628.31	2629.29	1323.66	R	1441.78	1423.77	1424.75	721.39	11
24	2809.38	2791.37	2792.36	1405.19	Y	1285.68	1267.67	1268.65	643.34	10
25	2972.45	2954.44	2955.42	1486.72	Y	1122.62	1104.61	1105.59	561.81	9
26	3059.48	3041.47	3042.45	1530.24	S	959.55	941.54	942.53	480.28	8
27	3187.54	3169.53	3170.51	1594.27	Q	872.45	854.51	855.49	436.76	7
28	3286.61	3268.59	3269.58	1643.80	V	744.46	726.45	727.38	372.73	6
29	3357.64	3339.63	3340.62	1679.32	A	645.39	627.38	628.37	323.20	5
30	3513.74	3495.73	3496.72	1757.37	R	574.36	556.35	557.33	287.68	4
31	3610.80	3592.79	3593.77	1805.90	P	418.26	400.25	401.23	209.63	3
32	3766.90	3748.89	3749.87	1883.95	R	321.13	303.19	304.11	161.10	2
33					F(-.98)	165.10	147.09	148.08	83.05	1

long neuropeptide F-1-PP$^{1-21}$ (NPF-1-PP$^{1-21}$)

long neuropeptide F-2-PP$^{1-21}$ (NPF-2-PP$^{1-21}$)

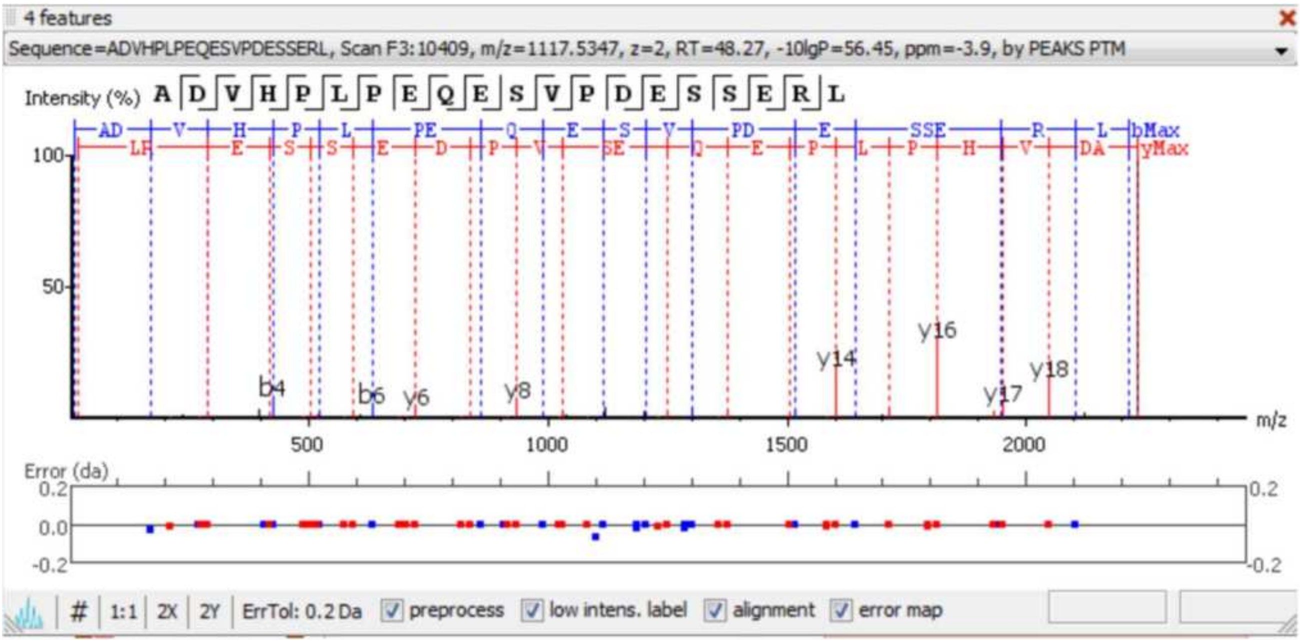

#	b	b-H2O	b-NH3	b (2+)	Seq	y	y-H2O	y-NH3	y (2+)	#
1	72.04	54.03	55.02	36.52	A					20
2	187.07	169.10	170.04	94.04	D	2163.02	2145.01	2145.99	1082.02	19
3	286.14	268.13	269.11	143.57	V	2047.99	2029.98	2030.97	1024.50	18
4	423.20	405.19	406.17	212.10	H	1948.92	1930.91	1931.90	974.96	17
5	520.25	502.24	503.22	260.63	P	1811.87	1793.86	1794.85	906.43	16
6	633.33	615.33	616.31	317.17	L	1714.81	1696.80	1697.79	857.91	15
7	730.39	712.38	713.36	365.69	P	1601.73	1583.71	1584.72	801.36	14
8	859.43	841.42	842.40	430.22	E	1504.68	1486.67	1487.65	752.84	13
9	987.49	969.48	970.46	494.24	Q	1375.63	1357.62	1358.60	688.32	12
10	1116.54	1098.52	1099.57	558.77	E	1247.58	1229.58	1230.55	624.29	11
11	1203.56	1185.56	1186.56	602.28	S	1118.53	1100.52	1101.51	559.77	10
12	1302.63	1284.62	1285.62	651.82	V	1031.50	1013.49	1014.47	516.26	9
13	1399.69	1381.68	1382.66	700.34	P	932.43	914.42	915.41	466.72	8
14	1514.71	1496.70	1497.69	757.86	D	835.38	817.37	818.36	418.19	7
15	1643.76	1625.74	1626.73	822.38	E	720.35	702.34	703.33	360.68	6
16	1730.79	1712.78	1713.76	865.89	S	591.31	573.30	574.29	296.15	5
17	1817.82	1799.81	1800.79	909.40	S	504.28	486.27	487.25	252.64	4
18	1946.86	1928.85	1929.83	973.93	E	417.24	399.24	400.22	209.14	3
19	2102.96	2084.95	2085.94	1051.98	R	288.20	270.19	271.18	144.60	2
20					L	132.10	114.09	115.07	66.55	1

Neuropeptide-like precursor1-1$^{1-20}$ (NPLP1-1$^{1-20}$)

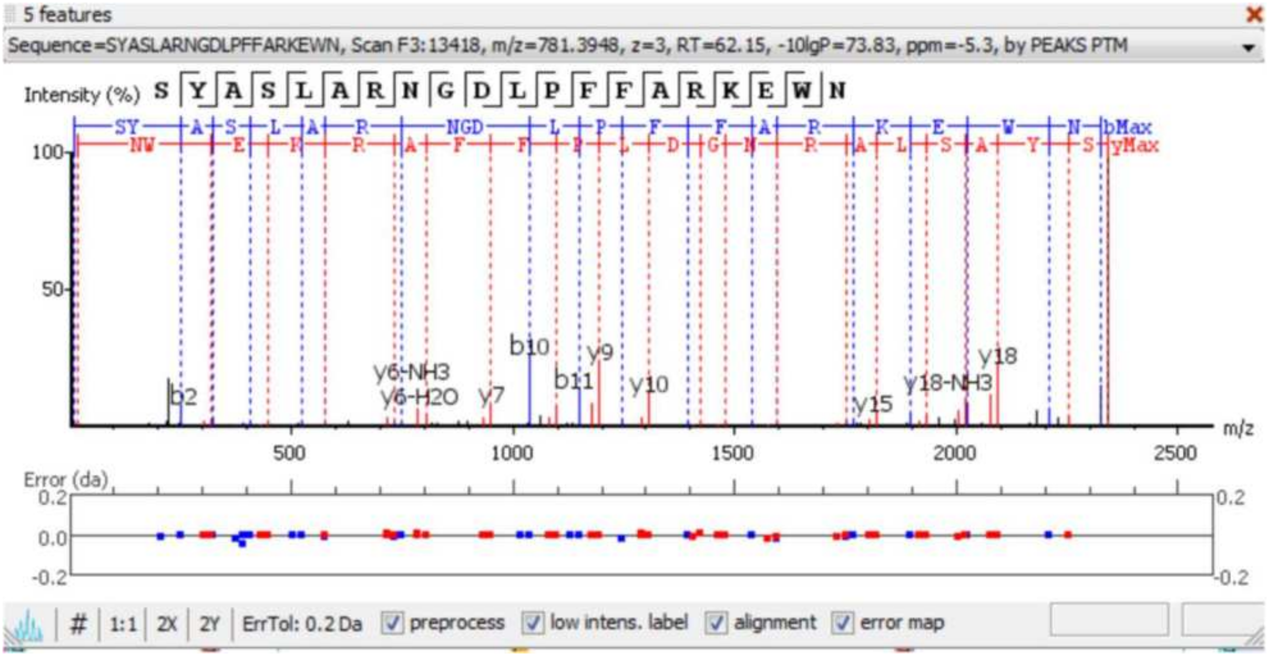

#	b	b-H2O	b-NH3	b (2+)	Seq	y	y-H2O	y-NH3	y (2+)	#
1	88.04	70.03	71.01	44.52	S					20
2	251.10	233.09	234.08	126.05	Y	2255.14	2237.12	2238.11	1128.07	19
3	322.14	304.13	305.11	161.57	A	2092.08	2074.06	2075.05	1046.54	18
4	409.17	391.16	392.20	205.10	S	2021.04	2003.02	2004.02	1011.02	17
5	522.25	504.25	505.23	261.63	L	1934.01	1915.99	1916.98	967.50	16
6	593.29	575.28	576.28	297.15	A	1820.92	1802.91	1803.90	910.96	15
7	749.39	731.38	732.38	375.22	R	1749.88	1731.87	1732.86	875.44	14
8	863.44	845.43	846.41	432.22	N	1593.79	1575.77	1576.77	797.39	13
9	920.46	902.45	903.43	460.73	G	1479.73	1461.73	1462.71	740.37	12
10	1035.49	1017.47	1018.46	518.24	D	1422.70	1404.72	1405.70	711.86	11
11	1148.57	1130.57	1131.55	574.78	L	1307.69	1289.67	1290.67	654.34	10
12	1245.65	1227.61	1228.60	623.31	P	1194.61	1176.60	1177.58	597.80	9
13	1392.70	1374.68	1375.66	696.85	F	1097.56	1079.54	1080.53	549.28	8
14	1539.76	1521.75	1522.73	770.38	F	950.49	932.47	933.46	475.74	7
15	1610.80	1592.79	1593.79	805.90	A	803.42	785.39	786.39	402.21	6
16	1766.90	1748.89	1749.88	883.95	R	732.38	714.35	715.36	366.69	5
17	1894.99	1876.98	1877.97	948.00	K	576.28	558.27	559.25	288.64	4
18	2024.04	2006.02	2007.01	1012.52	E	448.18	430.17	431.16	224.59	3
19	2210.12	2192.10	2193.09	1105.56	W	319.14	301.13	302.11	160.07	2
20					N	133.06	115.05	116.03	67.03	1

Neuropeptide-like precursor1-2 (NPLP1-2)

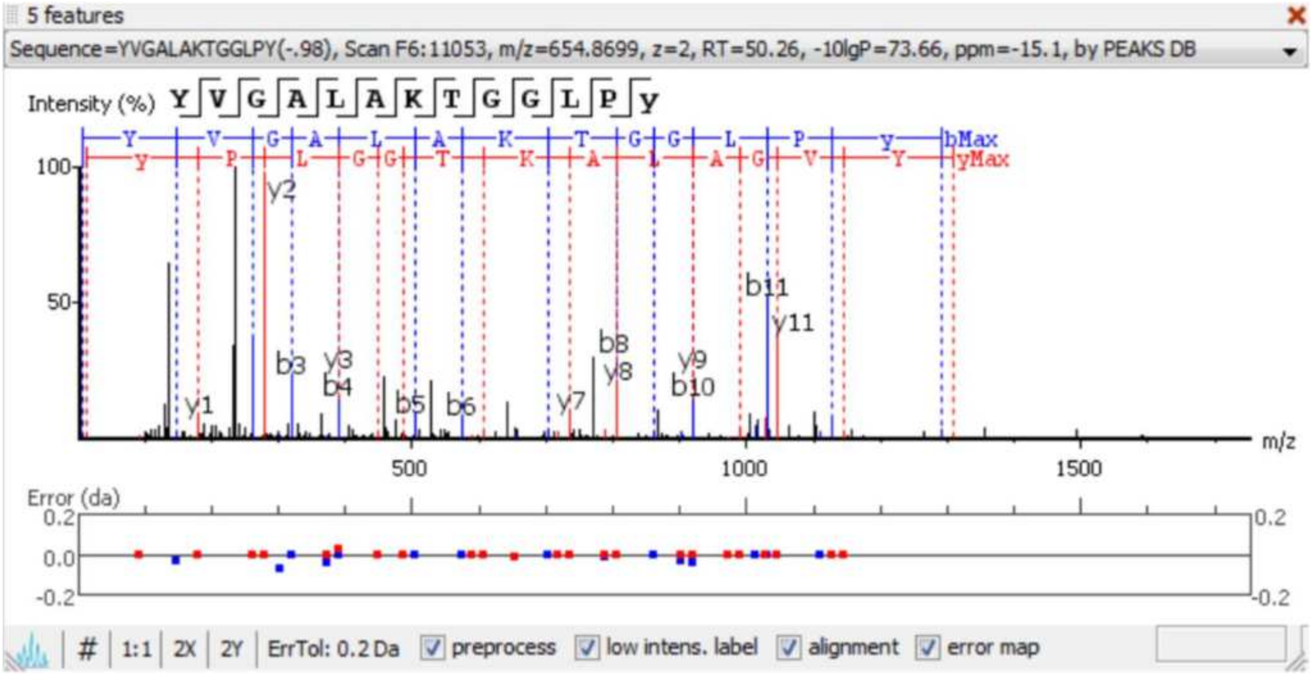

#	b	b-H2O	b-NH3	b (2+)	Seq	y	y-H2O	y-NH3	y (2+)	#
1	164.07	146.06	147.08	82.54	Y					13
2	263.14	245.13	246.11	132.07	V	1145.67	1127.66	1128.64	573.33	12
3	320.16	302.15	303.21	160.58	G	1046.60	1028.59	1029.57	523.80	11
4	391.20	373.19	374.21	196.10	A	989.58	971.57	972.55	495.29	10
5	504.28	486.27	487.26	252.64	L	918.54	900.53	901.52	459.77	9
6	575.32	557.31	558.29	288.16	A	805.46	787.45	788.43	403.23	8
7	703.41	685.40	686.39	352.21	K	734.42	716.41	717.39	367.71	7
8	804.47	786.45	787.45	402.73	T	606.33	588.31	589.30	303.66	6
9	861.48	843.47	844.46	431.24	G	505.28	487.27	488.25	253.14	5
10	918.54	900.49	901.52	459.75	G	448.26	430.24	431.23	224.63	4
11	1031.59	1013.58	1014.56	516.29	L	391.20	373.22	374.21	196.12	3
12	1128.64	1110.63	1111.61	564.82	P	278.15	260.14	261.12	139.57	2
13					Y(-.98)	181.10	163.09	164.07	91.05	1

Neuropeptide-like precursor1-3 (NPLP1-3) _partial

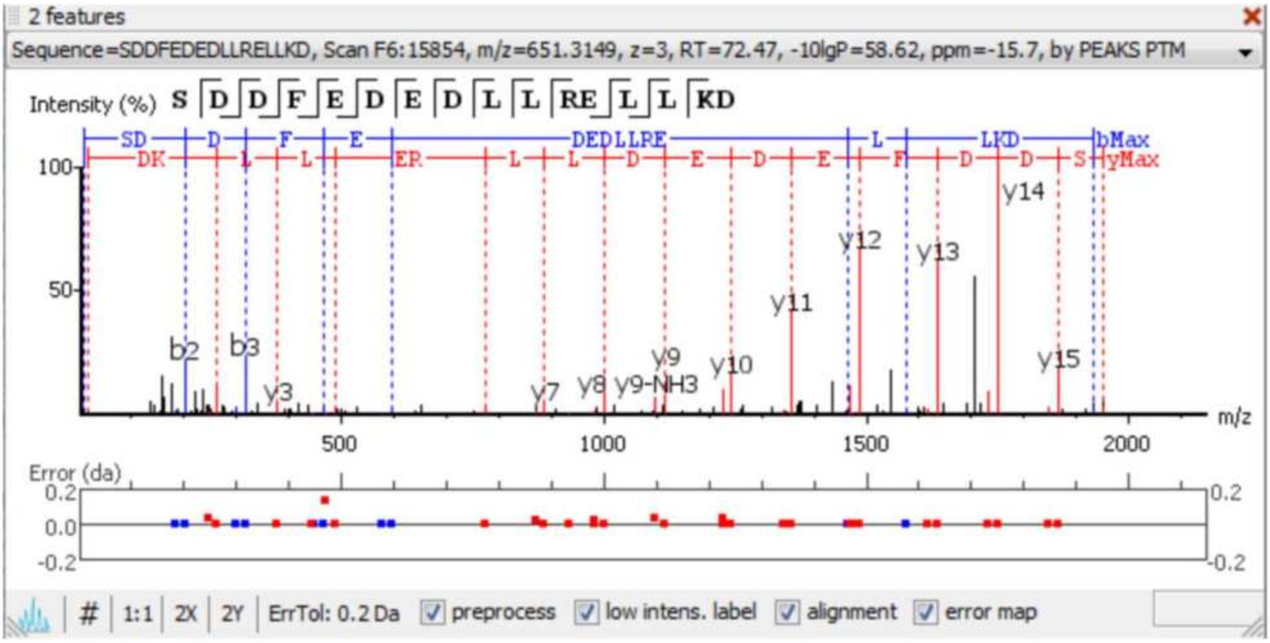

#	b	b-H2O	b-NH3	b (2+)	Seq	y	y-H2O	y-NH3	y (2+)	#
1	88.04	70.03	71.01	44.52	S					16
2	203.07	185.06	186.04	102.03	D	1864.88	1846.87	1847.85	932.95	15
3	318.09	300.08	301.07	159.55	D	1749.86	1731.84	1732.83	875.43	14
4	465.16	447.15	448.14	233.08	F	1634.83	1616.83	1617.80	817.91	13
5	594.21	576.19	577.18	297.60	E	1487.76	1469.75	1470.72	744.38	12
6	709.23	691.22	692.20	355.12	D	1358.72	1340.71	1341.69	679.86	11
7	838.27	820.26	821.25	419.64	E	1243.69	1225.68	1226.62	622.34	10
8	953.30	935.29	936.27	477.15	D	1114.65	1096.64	1097.59	557.82	9
9	1066.39	1048.37	1049.36	533.69	L	999.62	981.61	982.57	500.31	8
10	1179.47	1161.46	1162.44	590.23	L	886.54	868.53	869.48	443.77	7
11	1335.57	1317.56	1318.54	668.29	R	773.46	755.44	756.42	387.23	6
12	1464.62	1446.60	1447.59	732.81	E	617.35	599.34	600.32	309.18	5
13	1577.70	1559.69	1560.67	789.35	L	488.31	470.30	471.14	244.65	4
14	1690.78	1672.77	1673.75	845.89	L	375.22	357.21	358.20	188.11	3
15	1818.88	1800.87	1801.85	909.94	K	262.14	244.13	245.08	131.57	2
16					D	134.04	116.03	117.02	67.52	1

Neuropeptide-like precursor1-4 (NPLP1-4)

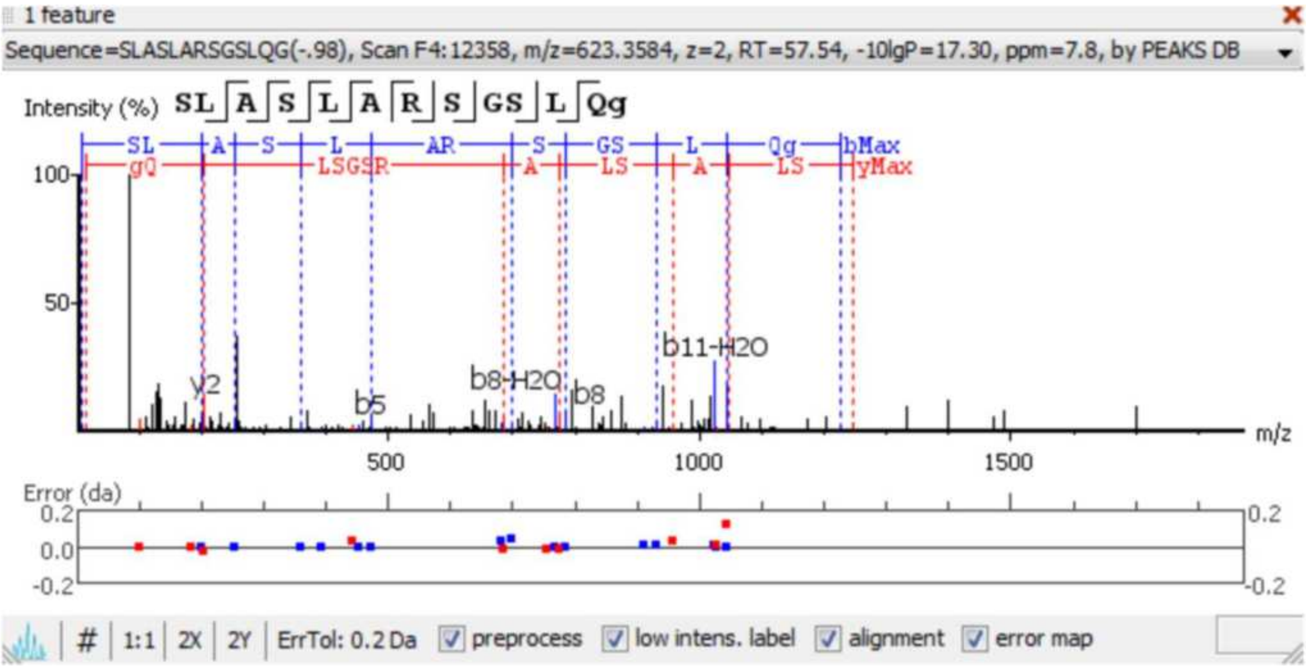

#	b	b-H2O	b-NH3	b (2+)	Seq	y	y-H2O	y-NH3	y (2+)	#
1	88.04	70.03	71.01	44.52	S					13
2	201.12	183.11	184.10	101.06	L	1158.66	1140.65	1141.63	579.83	12
3	272.16	254.16	255.13	136.58	A	1045.44	1027.55	1028.55	523.29	11
4	359.19	341.18	342.17	180.10	S	974.54	956.49	957.51	487.77	10
5	472.28	454.27	455.25	236.64	L	887.51	869.50	870.48	444.21	9
6	543.31	525.30	526.29	272.16	A	774.44	756.42	757.39	387.71	8
7	699.37	681.37	682.39	350.21	R	703.38	685.39	686.36	352.19	7
8	786.44	768.43	769.42	393.72	S	547.28	529.27	530.26	274.14	6
9	843.47	825.46	826.44	422.23	G	460.25	442.24	443.22	230.63	5
10	930.49	912.47	913.47	465.75	S	403.23	385.22	386.20	202.11	4
11	1043.58	1025.56	1026.56	522.29	L	316.20	298.19	299.17	158.60	3
12	1171.64	1153.63	1154.62	586.32	Q	203.14	185.09	186.09	102.06	2
13					G(-.98)	75.06	57.04	58.03	38.03	1

Neuropeptide-like precursor1-5 (NPLP1-5)

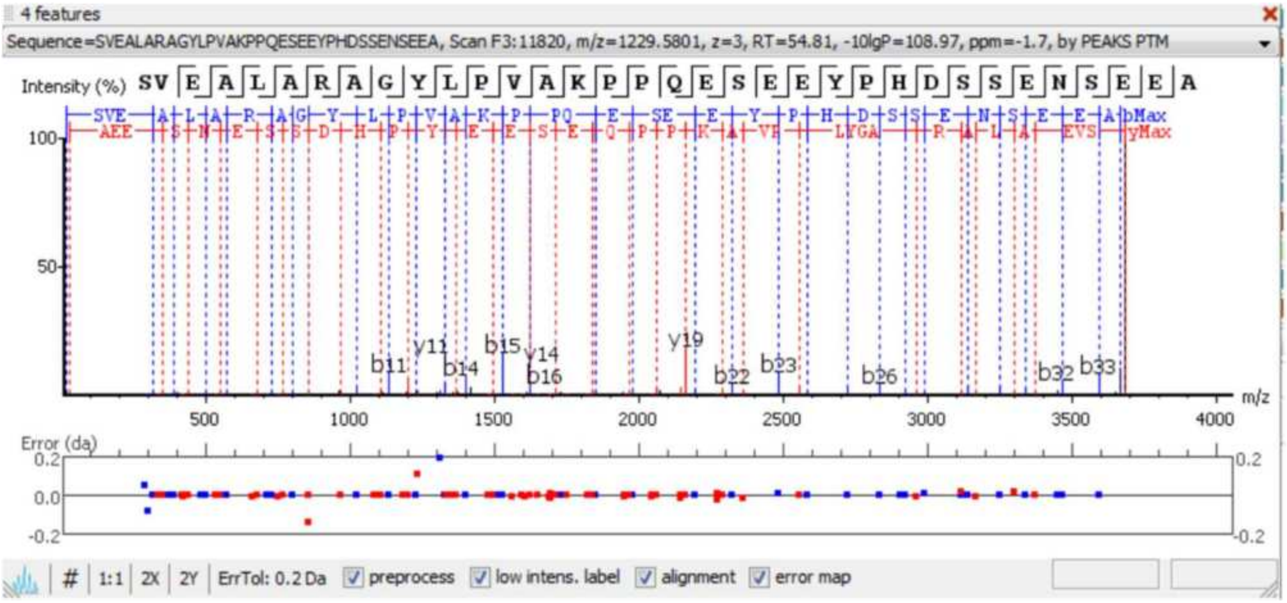

#	b	b-H2O	b-NH3	b (2+)	Seq	y	y-H2O	y-NH3	y (2+)	#
1	88.04	70.03	71.01	44.52	S					34
2	187.11	169.10	170.08	94.05	V	3599.68	3581.67	3582.65	1800.34	33
3	316.15	298.14	299.21	158.58	E	3500.61	3482.60	3483.58	1750.81	32
4	387.19	369.18	370.16	194.09	A	3371.56	3353.56	3354.54	1686.30	31
5	500.27	482.26	483.25	250.64	L	3300.51	3282.52	3283.50	1650.77	30
6	571.31	553.30	554.28	286.10	A	3187.44	3169.43	3170.43	1594.23	29
7	727.41	709.40	710.38	364.21	R	3116.39	3098.40	3099.38	1558.71	28
8	798.45	780.44	781.42	399.72	A	2960.32	2942.30	2943.28	1480.65	27
9	855.47	837.46	838.44	428.23	G	2889.27	2871.26	2872.24	1445.13	26
10	1018.53	1000.52	1001.51	509.77	Y	2832.25	2814.24	2815.22	1416.62	25
11	1131.62	1113.61	1114.59	566.31	L	2669.18	2651.17	2652.16	1335.09	24
12	1228.67	1210.66	1211.64	614.83	P	2556.10	2538.09	2539.07	1278.55	23
13	1327.74	1309.53	1310.71	664.37	V	2459.05	2441.04	2442.02	1229.91	22
14	1398.78	1380.76	1381.75	699.89	A	2360.00	2341.97	2342.95	1180.49	21
15	1526.87	1508.86	1509.84	763.93	K	2288.95	2270.92	2271.94	1144.97	20
16	1623.92	1605.91	1606.90	812.46	P	2160.85	2142.84	2143.84	1080.93	19
17	1720.97	1702.96	1703.95	860.99	P	2063.80	2045.78	2046.78	1032.40	18
18	1849.03	1831.02	1832.01	925.02	Q	1966.74	1948.73	1949.73	983.87	17
19	1978.08	1960.07	1961.05	989.54	E	1838.69	1820.67	1821.66	919.84	16
20	2065.11	2047.10	2048.08	1033.05	S	1709.64	1691.62	1692.64	855.47	15
21	2194.15	2176.14	2177.12	1097.58	E	1622.61	1604.60	1605.60	811.80	14
22	2323.19	2305.18	2306.17	1162.10	E	1493.57	1475.56	1476.54	747.28	13
23	2486.24	2468.25	2469.23	1243.63	Y	1364.52	1346.51	1347.50	682.76	12
24	2583.31	2565.30	2566.28	1292.15	P	1201.46	1183.45	1184.43	601.23	11
25	2720.37	2702.36	2703.34	1360.68	H	1104.41	1086.40	1087.38	552.70	10
26	2835.39	2817.38	2818.37	1418.20	D	967.35	949.34	950.32	484.17	9
27	2922.43	2904.42	2905.41	1461.71	S	852.32	834.31	835.29	426.66	8
28	3009.46	2991.43	2992.43	1505.24	S	765.29	747.28	748.27	383.14	7
29	3138.50	3120.49	3121.47	1569.75	E	678.26	660.25	661.24	339.63	6
30	3252.55	3234.53	3235.52	1626.77	N	549.22	531.20	532.19	275.11	5
31	3339.58	3321.57	3322.55	1670.29	S	435.17	417.16	418.16	218.09	4
32	3468.62	3450.61	3451.59	1734.81	E	348.14	330.13	331.11	174.57	3
33	3597.66	3579.65	3580.64	1799.33	E	219.10	201.09	202.07	110.05	2
34					A	90.05	72.04	73.03	45.53	1

Neuropeptide-like precursor1-6 (NPLP1-6)_partial

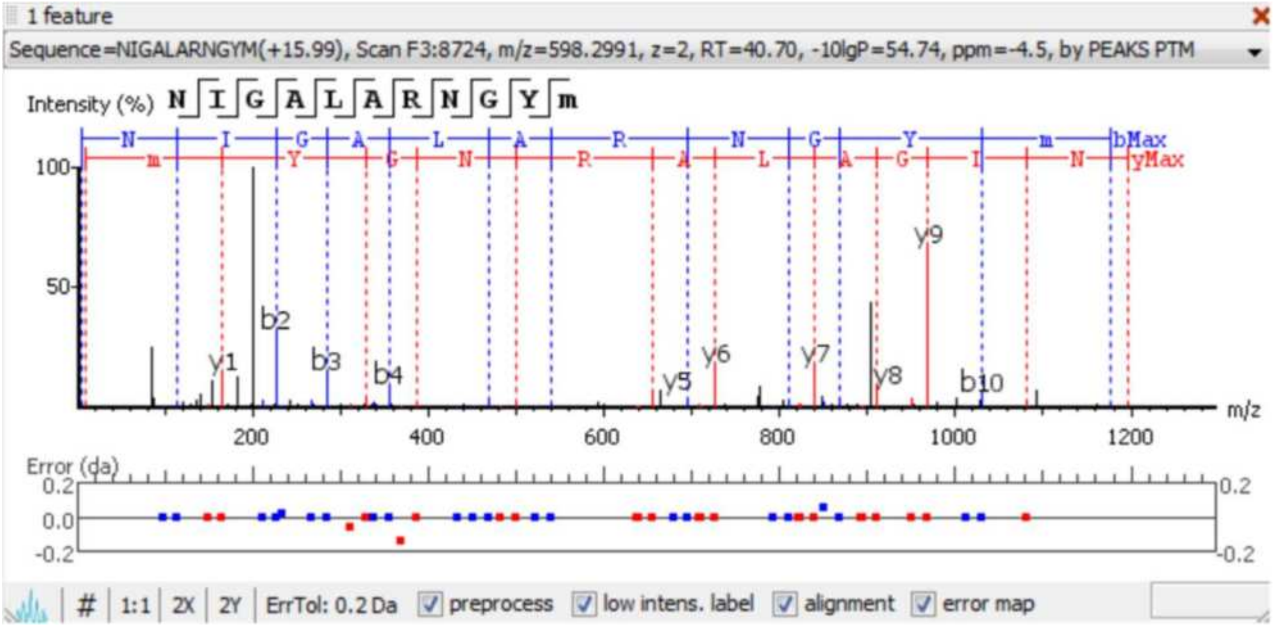

#	b	b-H2O	b-NH3	b (2+)	Seq	y	y-H2O	y-NH3	y (2+)	#
1	115.05	97.04	98.02	58.03	N					11
2	228.13	210.12	211.11	114.57	I	1081.55	1063.54	1064.52	541.27	10
3	285.16	267.14	268.13	143.08	G	968.46	950.45	951.45	484.73	9
4	356.19	338.18	339.17	178.60	A	911.44	893.43	894.42	456.22	8
5	469.29	451.27	452.25	235.11	L	840.40	822.39	823.38	420.70	7
6	540.31	522.30	523.29	270.66	A	727.32	709.31	710.30	364.16	6
7	696.42	678.41	679.39	348.71	R	656.28	638.27	639.25	328.64	5
8	810.46	792.45	793.43	405.73	N	500.18	482.17	483.15	250.59	4
9	867.48	849.47	850.39	434.24	G	386.14	368.13	369.25	193.57	3
10	1030.54	1012.53	1013.52	515.77	Y	329.12	311.17	312.09	165.06	2
11					M(+15.99)	166.05	148.04	149.03	83.53	1

Neuropeptide-like precursor1-7 (NPLP1-7)

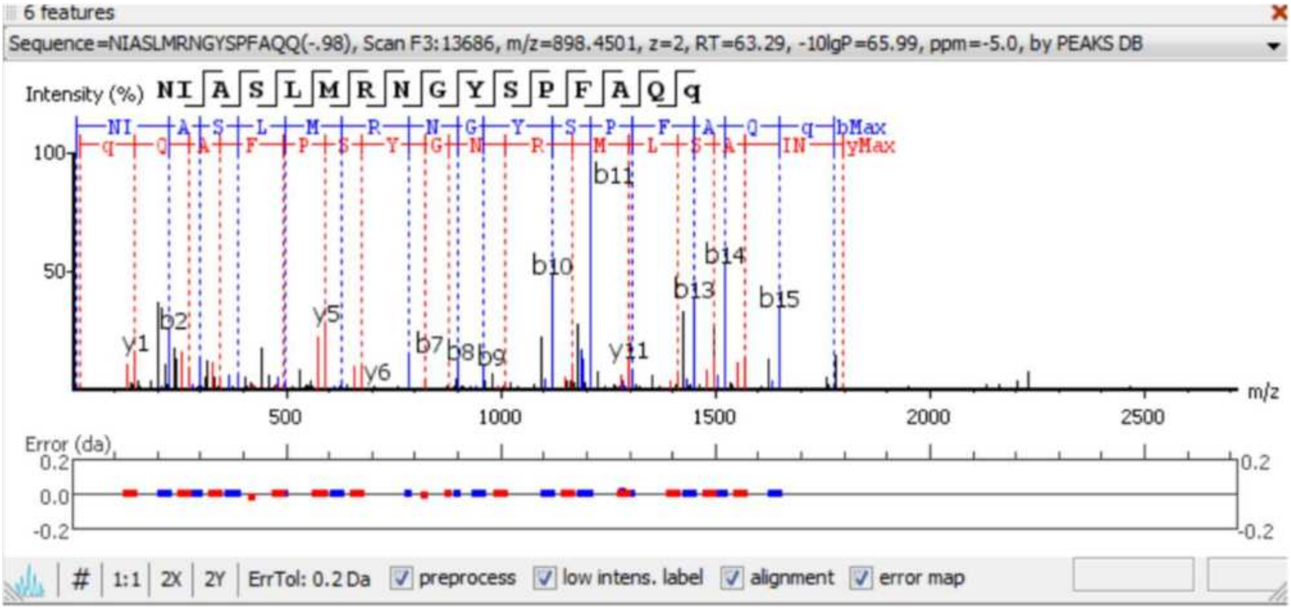

#	b	b-H2O	b-NH3	b (2+)	Seq	y	y-H2O	y-NH3	y (2+)	#
1	115.05	97.04	98.02	58.03	N					16
2	228.13	210.12	211.14	114.57	I	1681.85	1663.84	1664.82	841.42	15
3	299.17	281.16	282.15	150.09	A	1568.77	1550.75	1551.74	784.88	14
4	386.20	368.19	369.18	193.60	S	1497.73	1479.72	1480.70	749.36	13
5	499.29	481.28	482.26	250.14	L	1410.69	1392.68	1393.67	705.85	12
6	630.33	612.32	613.30	315.66	M	1297.61	1279.60	1280.58	649.31	11
7	786.43	768.42	769.40	393.71	R	1166.57	1148.56	1149.55	583.79	10
8	900.47	882.42	883.47	450.74	N	1010.46	992.46	993.44	505.73	9
9	957.49	939.48	940.47	479.25	G	896.43	878.42	879.40	448.71	8
10	1120.56	1102.56	1103.54	560.78	Y	839.40	821.39	822.39	420.23	7
11	1207.59	1189.58	1190.57	604.29	S	676.34	658.33	659.32	338.67	6
12	1304.64	1286.63	1287.62	652.82	P	589.31	571.30	572.28	295.15	5
13	1451.71	1433.70	1434.69	726.36	F	492.26	474.25	475.23	246.63	4
14	1522.75	1504.73	1505.73	761.87	A	345.19	327.18	328.16	173.09	3
15	1650.81	1632.79	1633.79	825.90	Q	274.15	256.14	257.12	137.58	2
16					Q(-.98)	146.09	128.08	129.07	73.55	1

Neuropeptide-like precursor1-8 (NPLP1-8)

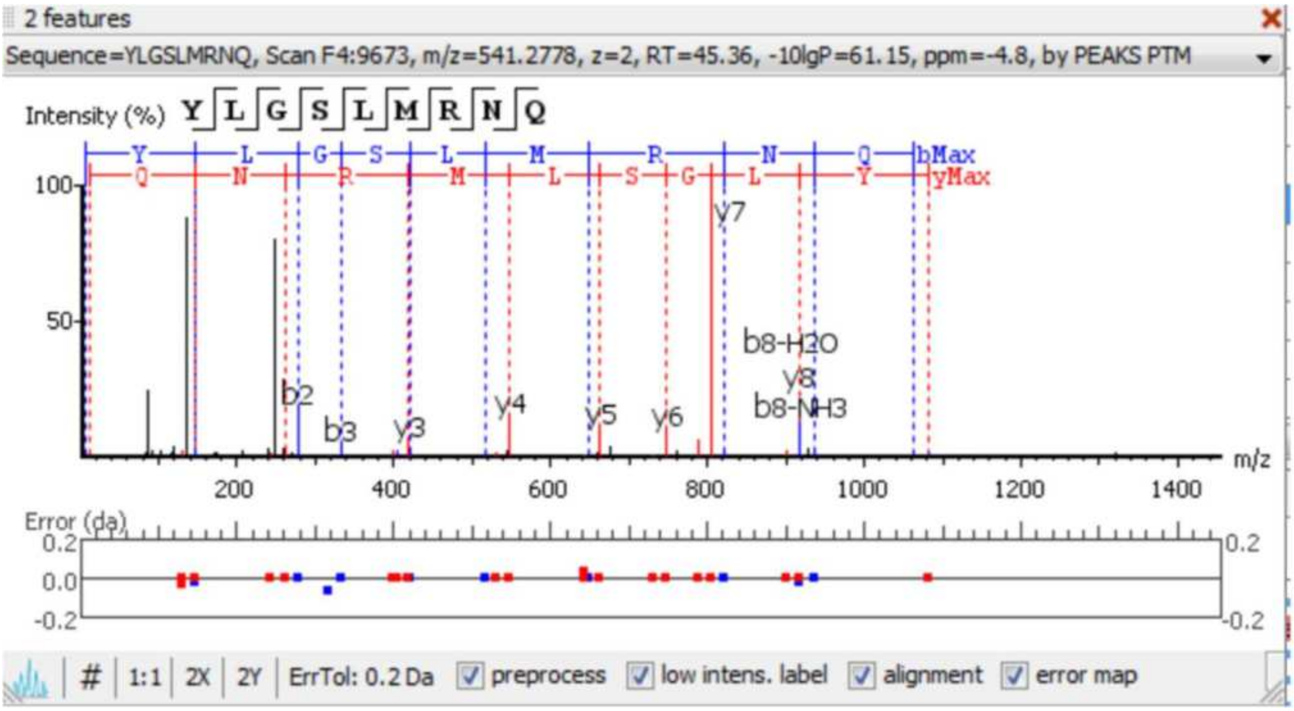

#	b	b-H2O	b-NH3	b (2+)	Seq	y	y-H2O	y-NH3	y (2+)	#
1	164.07	146.06	147.08	82.54	Y					9
2	277.15	259.14	260.13	139.08	L	918.48	900.47	901.46	459.74	8
3	334.18	316.17	317.22	167.59	G	805.40	787.39	788.37	403.20	7
4	421.21	403.20	404.18	211.10	S	748.38	730.36	731.35	374.69	6
5	534.29	516.28	517.27	267.65	L	661.34	643.29	644.32	331.17	5
6	665.33	647.32	648.31	333.17	M	548.26	530.25	531.23	274.63	4
7	821.43	803.42	804.41	411.22	R	417.22	399.21	400.19	209.11	3
8	935.48	917.46	918.48	468.24	N	261.12	243.11	244.09	131.06	2
9					Q	147.08	129.10	130.05	74.04	1

Neuropeptide-like precursor1-9 (NPLP1-9)

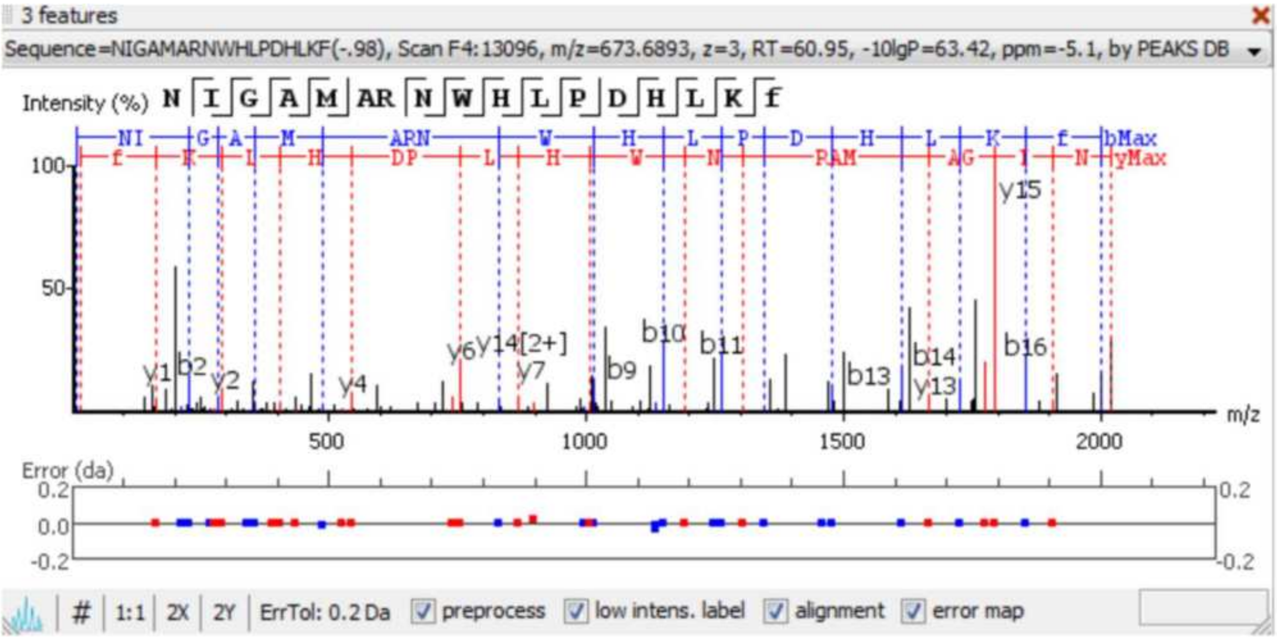

#	b	b-H2O	b-NH3	b (2+)	Seq	y	y-H2O	y-NH3	y (2+)	#
1	115.05	97.04	98.02	58.03	N					17
2	228.13	210.12	211.11	114.57	I	1905.01	1887.00	1887.98	953.00	16
3	285.16	267.15	268.13	143.08	G	1791.93	1773.91	1774.90	896.43	15
4	356.19	338.18	339.17	178.60	A	1734.90	1716.89	1717.87	867.95	14
5	487.25	469.22	470.21	244.12	M	1663.87	1645.85	1646.84	832.43	13
6	558.27	540.26	541.24	279.64	A	1532.82	1514.81	1515.80	766.91	12
7	714.37	696.36	697.35	357.69	R	1461.79	1443.78	1444.76	731.39	11
8	828.42	810.40	811.39	414.71	N	1305.69	1287.67	1288.66	653.34	10
9	1014.49	996.48	997.47	507.75	W	1191.63	1173.63	1174.62	596.32	9
10	1151.56	1133.58	1134.54	576.28	H	1005.56	987.55	988.54	503.28	8
11	1264.64	1246.63	1247.61	632.82	L	868.50	850.49	851.48	434.76	7
12	1361.69	1343.68	1344.67	681.35	P	755.42	737.41	738.39	378.21	6
13	1476.72	1458.71	1459.70	738.86	D	658.37	640.36	641.34	329.68	5
14	1613.78	1595.77	1596.75	807.39	H	543.34	525.33	526.31	272.17	4
15	1726.86	1708.85	1709.83	863.93	L	406.28	388.27	389.26	203.64	3
16	1854.96	1836.94	1837.93	927.98	K	293.20	275.19	276.17	147.10	2
17					F(-.98)	165.10	147.09	148.08	83.05	1

Neuropeptide-like precursor1-10 (NPLP1-10)

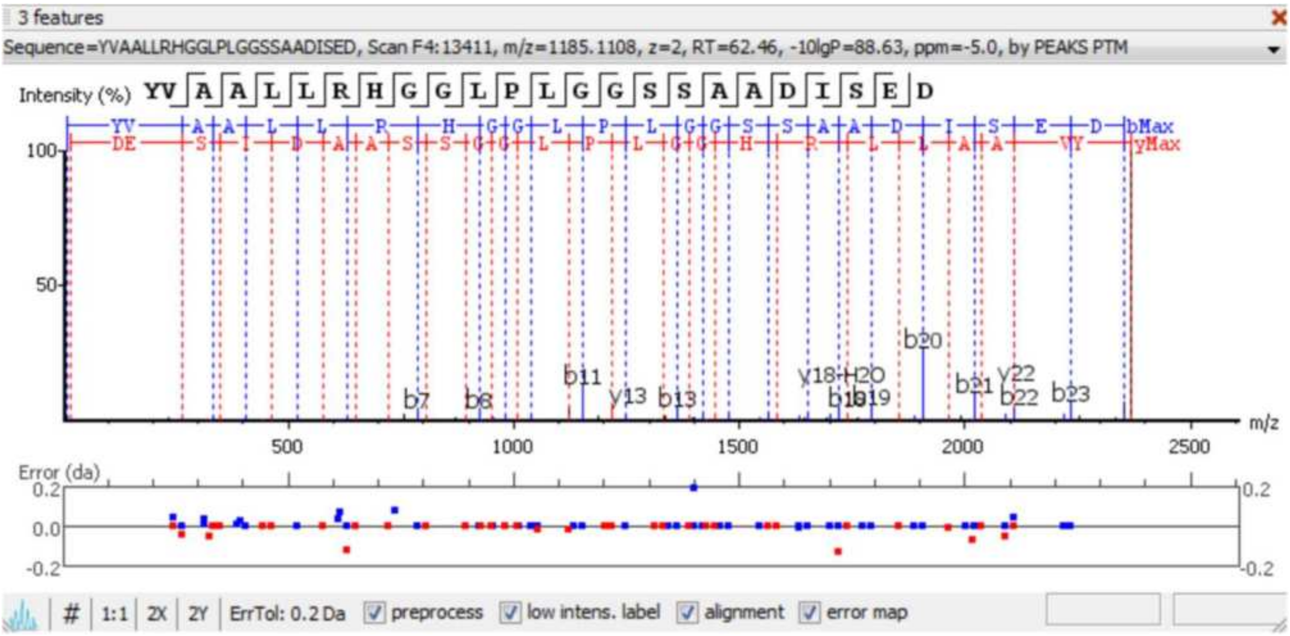

#	b	b-H2O	b-NH3	b (2+)	Seq	y	y-H2O	y-NH3	y (2+)	#
1	164.07	146.06	147.04	82.54	Y					24
2	263.14	245.08	246.11	132.07	V	2206.15	2188.14	2189.12	1103.57	23
3	334.18	316.15	317.15	167.59	A	2107.08	2089.12	2090.05	1054.06	22
4	405.21	387.19	388.19	203.11	A	2036.04	2018.03	2019.09	1018.52	21
5	518.30	500.29	501.27	259.65	L	1965.02	1946.99	1947.98	983.01	20
6	631.38	613.33	614.28	316.15	L	1851.92	1833.91	1834.89	926.46	19
7	787.48	769.47	770.46	394.21	R	1738.84	1720.96	1721.81	869.92	18
8	924.54	906.53	907.51	462.77	H	1582.73	1564.72	1565.71	791.87	17
9	981.56	963.55	964.54	491.28	G	1445.68	1427.66	1428.65	723.34	16
10	1038.59	1020.57	1021.56	519.79	G	1388.65	1370.64	1371.63	694.83	15
11	1151.67	1133.66	1134.64	576.33	L	1331.64	1313.62	1314.61	666.32	14
12	1248.72	1230.71	1231.69	624.86	P	1218.55	1200.54	1201.52	609.77	13
13	1361.81	1343.80	1344.78	681.40	L	1121.52	1103.49	1104.47	561.25	12
14	1418.83	1400.63	1401.80	709.91	G	1008.41	990.40	991.38	504.71	11
15	1475.85	1457.84	1458.82	738.34	G	951.39	933.38	934.36	476.20	10
16	1562.89	1544.86	1545.86	781.94	S	894.37	876.36	877.34	447.68	9
17	1649.92	1631.90	1632.90	825.46	S	807.34	789.33	790.31	404.17	8
18	1720.96	1702.94	1703.92	860.97	A	720.30	702.29	703.28	360.65	7
19	1791.99	1773.98	1774.96	896.49	A	649.27	631.38	632.24	325.19	6
20	1907.02	1889.01	1889.99	954.01	D	578.23	560.22	561.20	289.62	5
21	2020.09	2002.09	2003.07	1010.55	I	463.20	445.19	446.18	232.10	4
22	2107.08	2089.12	2090.10	1054.06	S	350.12	332.11	333.09	175.56	3
23	2236.17	2218.16	2219.15	1118.59	E	263.14	245.08	246.06	132.04	2
24					D	134.04	116.03	117.02	67.52	1

Neuropeptide-like precursor1-11 (NPLP1-11)

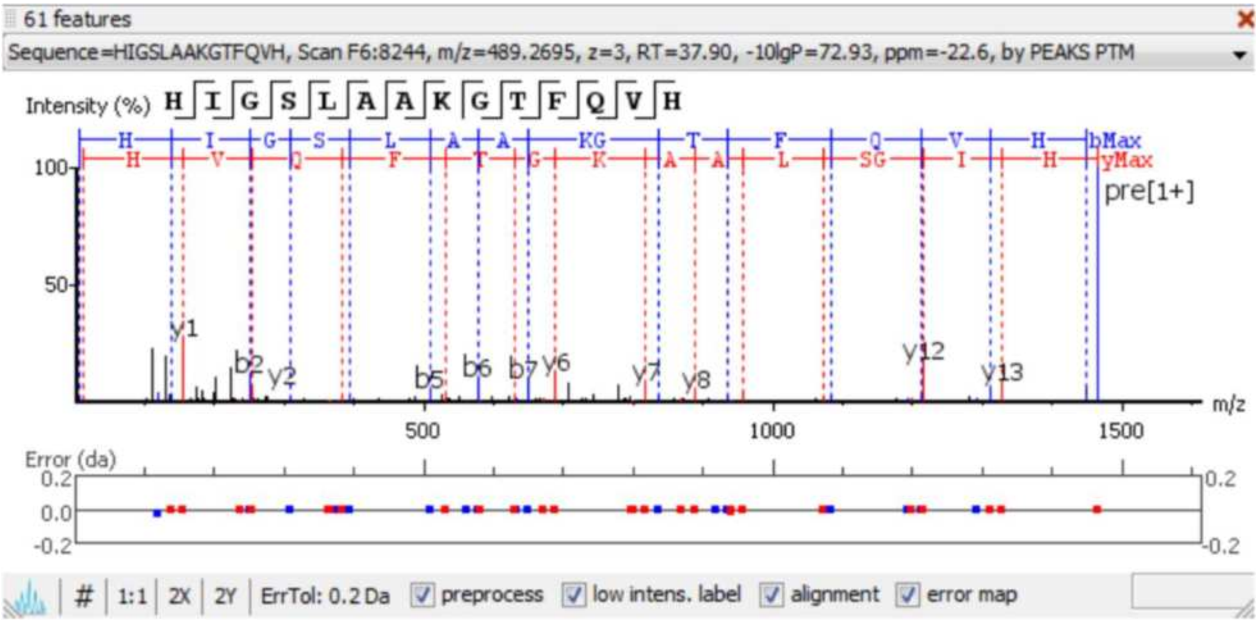

#	b	b-H2O	b-NH3	b (2+)	Seq	y	y-H2O	y-NH3	y (2+)	#
1	138.07	120.08	121.04	69.53	H					14
2	251.15	233.14	234.12	126.08	I	1328.73	1310.72	1311.71	664.87	13
3	308.17	290.16	291.15	154.59	G	1215.64	1197.64	1198.62	608.32	12
4	395.20	377.19	378.18	198.10	S	1158.63	1140.62	1141.60	579.82	11
5	508.29	490.28	491.26	254.64	L	1071.59	1053.58	1054.57	536.30	10
6	579.33	561.31	562.30	290.16	A	958.51	940.49	941.50	479.76	9
7	650.36	632.36	633.34	325.68	A	887.47	869.46	870.45	444.24	8
8	778.46	760.45	761.43	389.73	K	816.44	798.43	799.42	408.72	7
9	835.48	817.47	818.45	418.24	G	688.34	670.33	671.31	344.67	6
10	936.53	918.52	919.50	468.76	T	631.32	613.31	614.29	316.16	5
11	1083.60	1065.58	1066.57	542.30	F	530.27	512.26	513.25	265.64	4
12	1211.65	1193.64	1194.64	606.33	Q	383.20	365.20	366.18	192.10	3
13	1310.72	1292.71	1293.70	655.86	V	255.15	237.13	238.12	128.07	2
14					H	156.08	138.07	139.05	78.54	1

Neuropeptide-like precursor1-12 (NPLP1-12)_partial

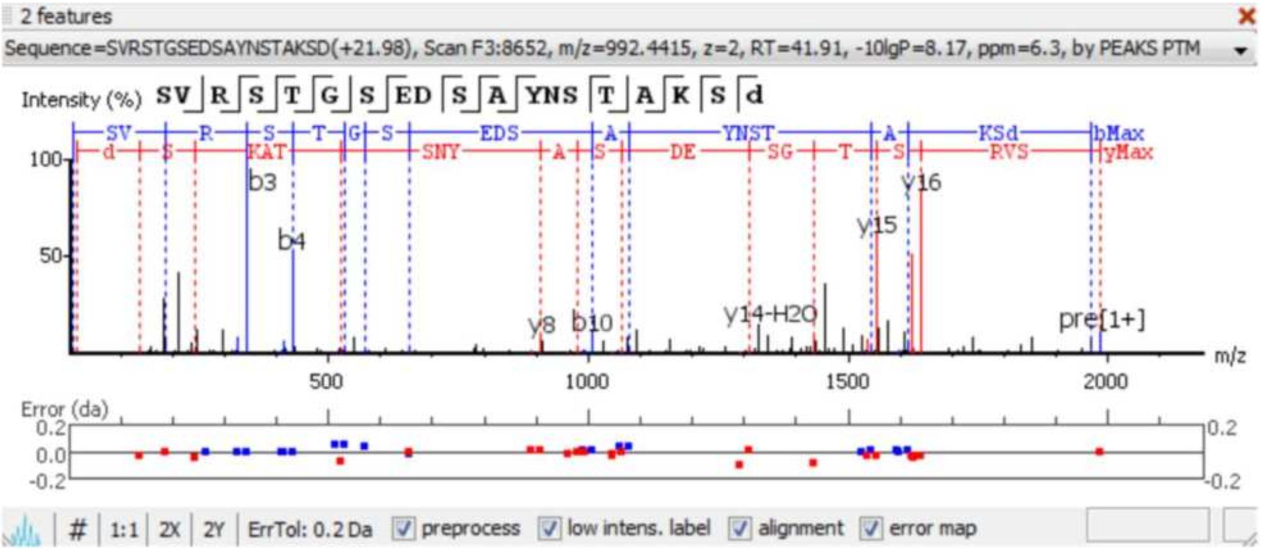

#	b	b-H2O	b-NH3	b (2+)	Seq	y	y-H2O	y-NH3	y (2+)	#
1	88.04	70.03	71.01	44.52	S					19
2	187.11	169.10	170.08	94.05	V	1896.82	1878.81	1879.79	948.91	18
3	343.20	325.19	326.17	172.07	R	1797.75	1779.74	1780.72	899.37	17
4	430.23	412.22	413.20	215.62	S	1641.68	1623.67	1624.68	821.32	16
5	531.22	513.28	514.20	266.14	T	1554.65	1536.61	1537.61	777.81	15
6	588.31	570.30	571.28	294.66	G	1453.57	1435.56	1436.54	727.28	14
7	675.34	657.33	658.32	338.17	S	1396.55	1378.54	1379.50	698.77	13
8	804.39	786.37	787.36	402.69	E	1309.51	1291.50	1292.59	655.26	12
9	919.41	901.40	902.39	460.21	D	1180.47	1162.46	1163.45	590.74	11
10	1006.42	988.41	989.40	503.72	S	1065.46	1047.45	1048.44	533.22	10
11	1077.48	1059.41	1060.42	539.24	A	978.43	960.40	961.40	489.71	9
12	1240.50	1222.53	1223.52	620.77	Y	907.35	889.34	890.33	454.19	8
13	1354.59	1336.58	1337.56	677.79	N	744.31	726.30	727.29	372.66	7
14	1441.62	1423.61	1424.59	721.31	S	630.27	612.26	613.24	315.64	6
15	1542.62	1524.62	1525.64	771.83	T	543.24	525.23	526.21	272.12	5
16	1613.70	1595.69	1596.68	807.35	A	442.19	424.18	425.16	221.60	4
17	1741.80	1723.79	1724.77	871.40	K	371.15	353.14	354.14	186.08	3
18	1828.83	1810.82	1811.80	914.92	S	243.11	225.05	226.08	122.03	2
19					D(+21.98)	156.03	138.02	139.00	78.51	1

Neuropeptide-like precursor1-13 (NPLP1-13)_partial

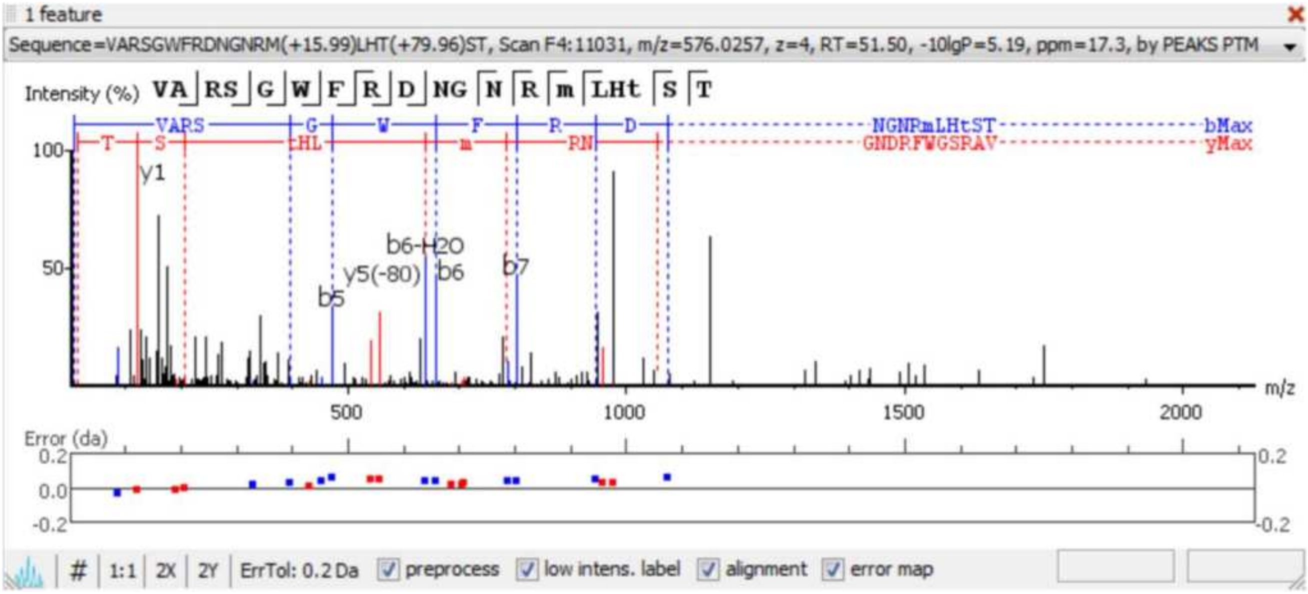

#	b	b-H2O	b-NH3	b (2+)	Seq	y	y-H2O	y-NH3	y (2+)	#
1	100.08	82.07	83.05	50.54	V					19
2	171.11	153.10	154.09	86.10	A	2201.96	2183.95	2184.93	1101.48	18
3	327.21	309.20	310.19	164.11	R	2130.92	2112.91	2113.89	1065.96	17
4	414.25	396.20	397.22	207.62	S	1974.82	1956.81	1957.79	987.91	16
5	471.20	453.21	454.24	236.13	G	1887.79	1869.78	1870.76	944.39	15
6	657.30	639.29	640.32	329.15	W	1830.76	1812.75	1813.74	915.88	14
7	804.37	786.36	787.39	402.71	F	1644.69	1626.67	1627.66	822.84	13
8	960.52	942.51	943.43	480.76	R	1497.62	1479.61	1480.59	749.31	12
9	1075.48	1057.53	1058.52	538.27	D	1341.52	1323.51	1324.49	671.26	11
10	1189.59	1171.58	1172.56	595.29	N	1226.49	1208.48	1209.46	613.74	10
11	1246.61	1228.60	1229.58	623.80	G	1112.45	1094.44	1095.42	556.72	9
12	1360.65	1342.64	1343.62	680.83	N	1055.42	1037.41	1038.40	528.21	8
13	1516.75	1498.74	1499.73	758.88	R	941.38	923.37	924.35	471.19	7
14	1663.79	1645.78	1646.76	832.39	M(+15.99)	785.28	767.27	768.25	393.14	6
15	1776.87	1758.86	1759.84	888.94	L	638.24	620.23	621.22	319.62	5
16	1913.93	1895.92	1896.90	957.47	H	525.16	507.15	508.13	263.08	4
17	2094.94	2076.92	2077.91	1047.97	T(+79.96)	388.10	370.09	371.08	194.55	3
18	2181.97	2163.96	2164.94	1091.48	S	207.10	189.09	190.08	104.05	2
19					T	120.08	102.05	103.04	60.53	1

Neuropeptide-like precursor1-15 (NPLP1-15)

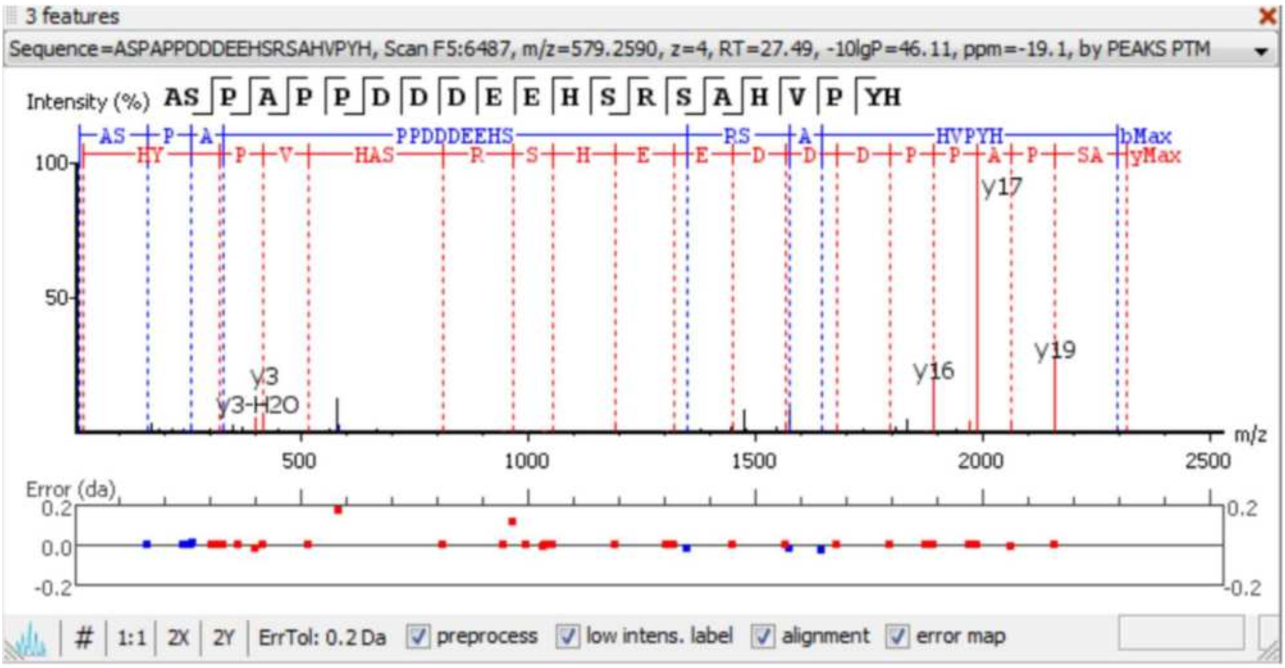

#	b	b-H2O	b-NH3	b (2+)	Seq	y	y-H2O	y-NH3	y (2+)	#
1	72.04	54.03	55.02	36.52	A					21
2	159.08	141.07	142.05	80.04	S	2242.97	2224.96	2225.95	1121.99	20
3	256.13	238.12	239.10	128.56	P	2155.94	2137.93	2138.92	1078.47	19
4	327.17	309.16	310.14	164.08	A	2058.89	2040.88	2041.86	1029.94	18
5	424.22	406.21	407.19	212.61	P	1987.85	1969.84	1970.83	994.43	17
6	521.27	503.26	504.25	261.12	P	1890.80	1872.79	1873.77	945.90	16
7	636.30	618.29	619.27	318.65	D	1793.75	1775.73	1776.74	897.37	15
8	751.33	733.32	734.30	376.16	D	1678.72	1660.71	1661.69	839.86	14
9	866.35	848.34	849.33	433.68	D	1563.69	1545.68	1546.67	782.35	13
10	995.40	977.39	978.37	498.20	E	1448.67	1430.66	1431.64	724.83	12
11	1124.42	1106.43	1107.41	562.72	E	1319.62	1301.61	1302.60	660.31	11
12	1261.50	1243.49	1244.47	631.25	H	1190.58	1172.57	1173.55	595.79	10
13	1348.55	1330.52	1331.50	674.76	S	1053.53	1035.51	1036.51	527.26	9
14	1504.63	1486.62	1487.60	752.82	R	966.49	948.48	949.46	483.75	8
15	1591.66	1573.65	1574.64	796.46	S	810.39	792.38	793.36	405.69	7
16	1662.70	1644.69	1645.67	831.85	A	723.36	705.35	706.33	362.22	6
17	1799.76	1781.75	1782.73	900.38	H	652.32	634.31	635.29	326.66	5
18	1898.83	1880.82	1881.80	949.91	V	515.26	497.25	498.23	258.13	4
19	1995.88	1977.87	1978.85	998.44	P	416.19	398.18	399.17	208.60	3
20	2158.94	2140.93	2141.92	1079.97	Y	319.14	301.13	302.11	160.07	2
21					H	156.08	138.07	139.05	78.54	1

Carausius-Neuropeptide-like precursor-1-1 (CNPLP-1-1)

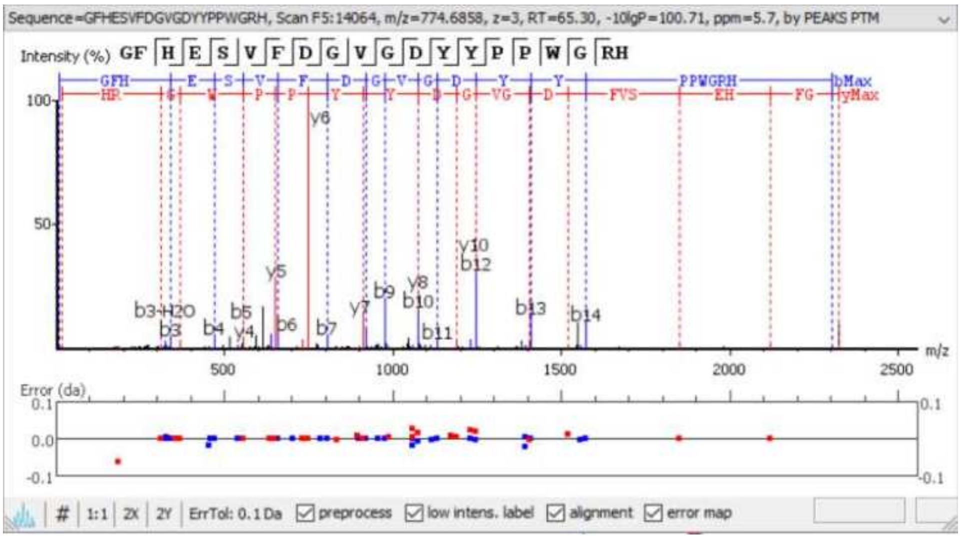

#	b	b-H2O	b-NH3	b (2+)	Seq	y	y-H2O	y-NH3	y (2+)	#
1	58.03	40.02	41.00	29.51	G					20
2	205.10	187.09	188.07	103.05	F	2265.01	2247.00	2247.99	1133.01	19
3	342.16	324.15	325.13	171.58	H	2117.95	2099.94	2100.92	1059.47	18
4	471.20	453.21	454.17	236.10	E	1980.89	1962.88	1963.86	990.94	17
5	558.23	540.22	541.20	279.62	S	1851.84	1833.83	1834.82	926.42	16
6	657.30	639.29	640.27	329.15	V	1764.81	1746.80	1747.79	882.91	15
7	804.37	786.36	787.34	402.68	F	1665.74	1647.73	1648.72	833.38	14
8	919.40	901.39	902.37	460.20	D	1518.66	1500.67	1501.65	759.84	13
9	976.42	958.40	959.39	488.71	G	1403.65	1385.64	1386.62	702.32	12
10	1075.49	1057.47	1058.48	538.24	V	1346.63	1328.62	1329.60	673.81	11
11	1132.51	1114.50	1115.48	566.75	G	1247.54	1229.52	1230.53	624.28	10
12	1247.54	1229.52	1230.51	624.27	D	1190.53	1172.52	1173.51	595.77	9
13	1410.60	1392.58	1393.60	705.80	Y	1075.49	1057.47	1058.48	538.26	8
14	1573.66	1555.65	1556.63	787.33	Y	912.45	894.43	895.42	456.72	7
15	1670.71	1652.70	1653.69	835.86	P	749.38	731.37	732.36	375.19	6
16	1767.77	1749.75	1750.74	884.38	P	652.33	634.32	635.31	326.67	5
17	1953.84	1935.83	1936.82	977.42	W	555.28	537.27	538.25	278.14	4
18	2010.87	1992.86	1993.84	1005.93	G	369.20	351.19	352.17	185.16	3
19	2166.97	2148.96	2149.94	1083.98	R	312.18	294.17	295.15	156.59	2
20					H	156.08	138.07	139.05	78.54	1

Carausius-Neuropeptide-like precursor-1-2 (CNPLP-1-2)

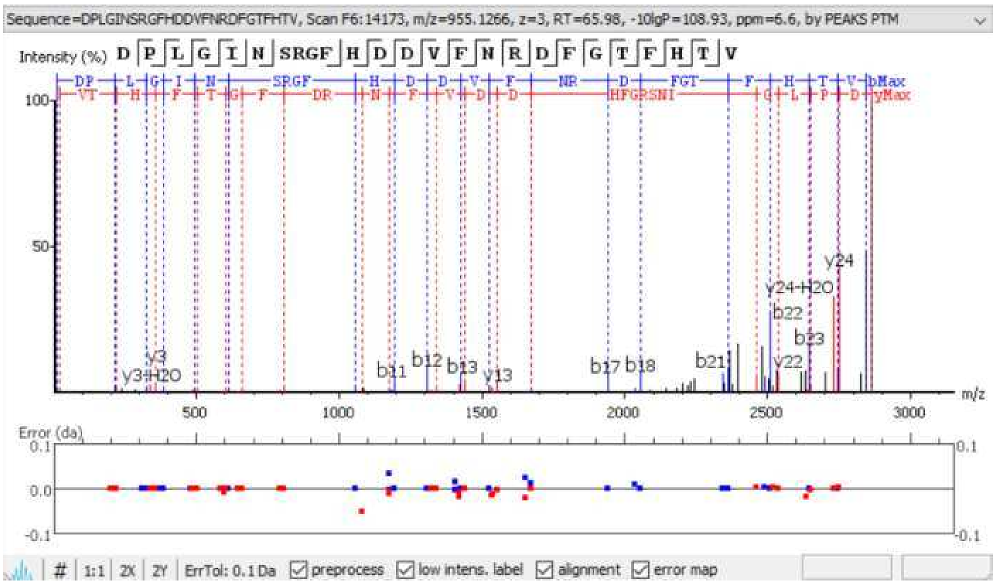

#	b	b-H2O	b-NH3	b (2+)	Seq	y	y-H2O	y-NH3	y (2+)	#
1	116.03	98.02	99.01	58.52	D					25
2	213.09	195.08	196.06	107.04	P	2748.32	2730.32	2731.30	1374.66	24
3	326.17	308.16	309.14	163.59	L	2651.28	2633.26	2634.27	1326.14	23
4	383.19	365.18	366.16	192.10	G	2538.19	2520.18	2521.16	1269.60	22
5	496.28	478.27	479.25	248.64	I	2481.17	2463.15	2464.14	1241.08	21
6	610.32	592.31	593.29	305.66	N	2368.09	2350.07	2351.06	1184.54	20
7	697.35	679.34	680.33	349.18	S	2254.04	2236.03	2237.02	1127.52	19
8	853.45	835.44	836.43	427.23	R	2167.01	2149.00	2149.98	1084.01	18
9	910.47	892.46	893.45	455.74	G	2010.91	1992.90	1993.88	1005.95	17
10	1057.54	1039.53	1040.52	529.27	F	1953.89	1935.88	1936.86	977.44	16
11	1194.60	1176.56	1177.58	597.80	H	1806.82	1788.81	1789.79	903.91	15
12	1309.63	1291.62	1292.60	655.31	D	1669.76	1651.75	1652.76	835.38	14
13	1424.66	1406.63	1407.63	712.83	D	1554.74	1536.74	1537.72	777.87	13
14	1523.72	1505.71	1506.70	762.36	V	1439.71	1421.72	1422.69	720.35	12
15	1670.78	1652.76	1653.77	835.90	F	1340.64	1322.63	1323.61	670.82	11
16	1784.84	1766.83	1767.81	892.92	N	1193.57	1175.57	1176.56	597.29	10
17	1940.93	1922.93	1923.91	970.97	R	1079.58	1061.52	1062.50	540.26	9
18	2055.97	2037.94	2038.94	1028.48	D	923.43	905.42	906.40	462.21	8
19	2203.03	2185.02	2186.00	1102.02	F	808.40	790.39	791.37	404.70	7
20	2260.05	2242.04	2243.03	1130.53	G	661.33	643.32	644.30	331.17	6
21	2361.10	2343.09	2344.07	1181.05	T	604.31	586.30	587.28	302.65	5
22	2508.17	2490.16	2491.14	1254.58	F	503.26	485.25	486.23	252.13	4
23	2645.23	2627.22	2628.20	1323.11	H	356.19	338.18	339.17	178.60	3
24	2746.28	2728.27	2729.25	1373.64	T	219.13	201.12	202.11	110.07	2
25					V	118.09	100.08	101.06	59.54	1

Carausius-Neuropeptide-like precursor-1-2$^{1-16}$ (CNPLP-1-2$^{1-16}$)

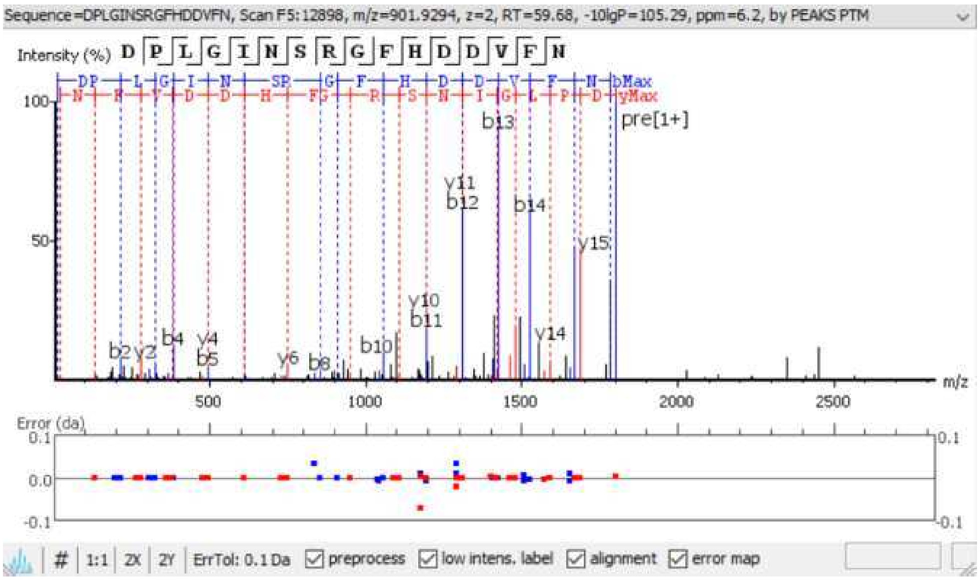

#	b	b-H2O	b-NH3	b (2+)	Seq	y	y-H2O	y-NH3	y (2+)	#
1	116.03	98.02	99.01	58.52	D					16
2	213.09	195.08	196.06	107.04	P	1687.82	1669.81	1670.79	844.41	15
3	326.17	308.16	309.14	163.59	L	1590.77	1572.76	1573.74	795.88	14
4	383.19	365.18	366.17	192.10	G	1477.68	1459.67	1460.66	739.34	13
5	496.28	478.27	479.25	248.64	I	1420.66	1402.65	1403.63	710.83	12
6	610.32	592.31	593.29	305.66	N	1307.58	1289.59	1290.55	654.29	11
7	697.35	679.34	680.33	349.18	S	1193.53	1175.52	1176.58	597.27	10
8	853.46	835.41	836.43	427.23	R	1106.50	1088.49	1089.47	553.75	9
9	910.48	892.46	893.45	455.74	G	950.40	932.39	933.37	475.70	8
10	1057.54	1039.54	1040.52	529.27	F	893.38	875.37	876.35	447.19	7
11	1194.61	1176.58	1177.57	597.80	H	746.31	728.30	729.28	373.66	6
12	1309.63	1291.61	1292.57	655.31	D	609.25	591.24	592.22	305.13	5
13	1424.66	1406.65	1407.63	712.83	D	494.23	476.21	477.20	247.61	4
14	1523.73	1505.70	1506.71	762.36	V	379.20	361.19	362.17	190.10	3
15	1670.79	1652.77	1653.77	835.90	F	280.13	262.12	263.10	140.56	2
16					N	133.06	115.05	116.03	67.03	1

Carausius-Neuropeptide-like precursor-1-3 (CNPLP-1-3)

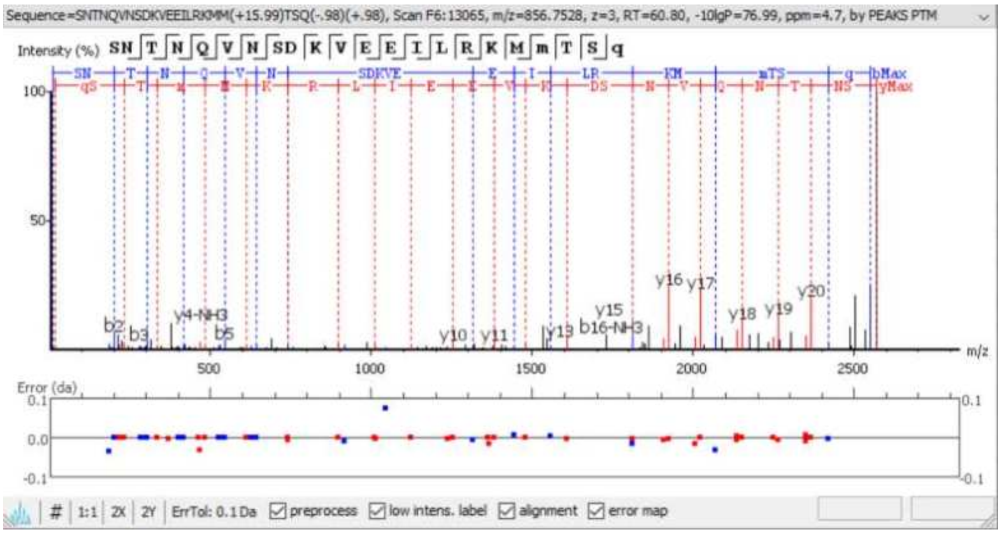

#	b	b-H2O	b-NH3	b (2+)	Seq	y	y-H2O	y-NH3	y (2+)	#
1	88.04	70.03	71.01	44.52	S					22
2	202.08	184.07	185.09	101.54	N	2481.21	2463.20	2464.18	1241.10	21
3	303.13	285.12	286.10	152.07	T	2367.17	2349.14	2350.15	1184.08	20
4	417.17	399.16	400.15	209.09	N	2266.12	2248.11	2249.09	1133.56	19
5	545.23	527.22	528.20	273.12	Q	2152.08	2134.06	2135.05	1076.54	18
6	644.30	626.29	627.27	322.65	V	2024.02	2006.00	2007.01	1012.51	17
7	758.34	740.33	741.33	379.67	N	1924.95	1906.94	1907.93	962.97	16
8	845.38	827.36	828.35	423.19	S	1810.91	1792.89	1793.88	905.95	15
9	960.40	942.39	943.38	480.70	D	1723.87	1705.86	1706.84	862.44	14
10	1088.50	1070.49	1071.47	544.75	K	1608.85	1590.83	1591.82	804.92	13
11	1187.57	1169.56	1170.54	594.28	V	1480.75	1462.74	1463.72	740.88	12
12	1316.61	1298.60	1299.58	658.80	E	1381.68	1363.67	1364.67	691.34	11
13	1445.64	1427.64	1428.62	723.33	E	1252.64	1234.63	1235.62	626.82	10
14	1558.73	1540.72	1541.71	779.87	I	1123.60	1105.59	1106.57	562.30	9
15	1671.82	1653.81	1654.79	836.41	L	1010.51	992.50	993.49	505.76	8
16	1827.92	1809.91	1810.91	914.47	R	897.43	879.42	880.40	449.21	7
17	1956.02	1938.00	1938.99	978.51	K	741.33	723.32	724.30	371.17	6
18	2087.06	2069.04	2070.06	1043.95	M	613.23	595.22	596.21	307.12	5
19	2234.09	2216.08	2217.06	1117.55	M(+15.99)	482.19	464.18	465.20	241.60	4
20	2335.14	2317.13	2318.11	1168.07	T	335.16	317.15	318.13	168.08	3
21	2422.18	2404.16	2405.14	1211.59	S	234.11	216.10	217.08	117.55	2
22					Q(.00)	147.08	129.07	130.05	74.04	1

Carausius-Neuropeptide-like precursor-1-3$^{1-15}$ (CNPLP-1-3$^{1-15}$)

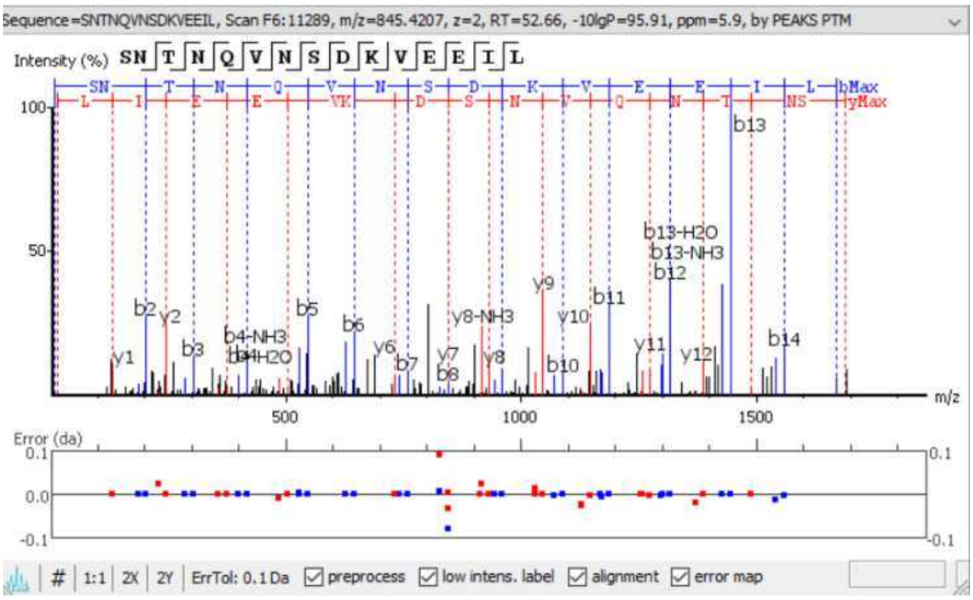

#	b	b-H2O	b-NH3	b (2+)	Seq	y	y-H2O	y-NH3	y (2+)	#
1	88.04	70.03	71.01	44.52	S					15
2	202.08	184.07	185.06	101.54	N	1602.80	1584.79	1585.77	801.90	14
3	303.13	285.12	286.10	152.07	T	1488.75	1470.74	1471.73	744.88	13
4	417.17	399.16	400.15	209.09	N	1387.71	1369.70	1370.70	694.35	12
5	545.23	527.22	528.20	273.12	Q	1273.67	1255.65	1256.64	637.33	11
6	644.30	626.29	627.27	322.65	V	1145.61	1127.62	1128.60	573.30	10
7	758.34	740.33	741.32	379.67	N	1046.54	1028.53	1029.49	523.77	9
8	845.46	827.36	828.34	423.19	S	932.49	914.48	915.44	466.75	8
9	960.40	942.39	943.38	480.70	D	845.46	827.45	828.34	423.23	7
10	1088.50	1070.49	1071.47	544.75	K	730.43	712.42	713.41	365.72	6
11	1187.57	1169.56	1170.55	594.28	V	602.34	584.33	585.31	301.67	5
12	1316.61	1298.60	1299.58	658.80	E	503.27	485.26	486.26	252.14	4
13	1445.65	1427.64	1428.63	723.33	E	374.23	356.22	357.20	187.61	3
14	1558.74	1540.72	1541.72	779.87	I	245.19	227.18	228.13	123.09	2
15					L	132.10	114.09	115.07	66.55	1

Carausius-Neuropeptide-like precursor-1-4 (CNPLP-1-4)

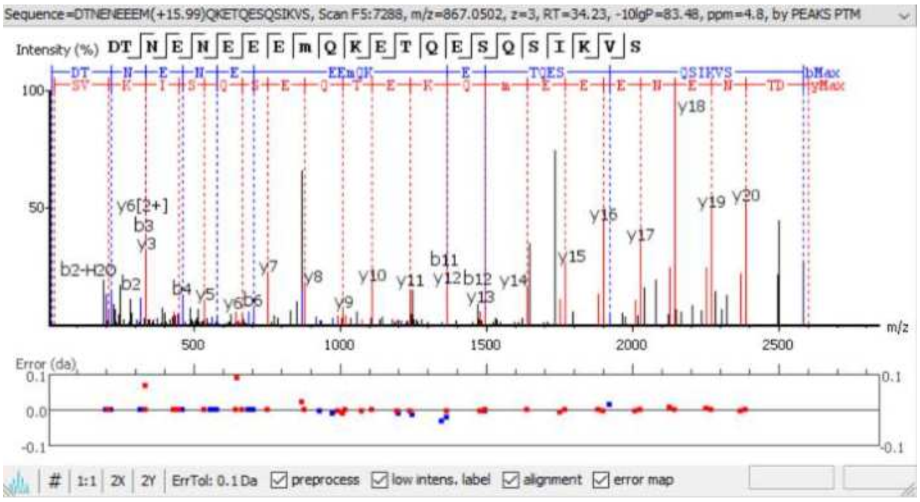

#	b	b-H2O	b-NH3	b (2+)	Seq	y	y-H2O	y-NH3	y (2+)	#
1	116.03	98.02	99.01	58.52	D					22
2	217.08	199.07	200.06	109.04	T	2484.10	2466.09	2467.08	1242.55	21
3	331.12	313.11	314.10	166.06	N	2383.06	2365.05	2366.03	1192.03	20
4	460.17	442.16	443.14	230.58	E	2269.01	2251.00	2251.99	1135.01	19
5	574.21	556.20	557.18	287.61	N	2139.97	2121.95	2122.94	1070.49	18
6	703.25	685.24	686.22	352.13	E	2025.93	2007.92	2008.90	1013.47	17
7	832.30	814.29	815.27	416.65	E	1896.89	1878.87	1879.86	948.94	16
8	961.34	943.33	944.31	481.17	E	1767.85	1749.84	1750.82	884.42	15
9	1108.37	1090.36	1091.35	554.69	M(+15.99)	1638.80	1620.79	1621.77	819.90	14
10	1236.43	1218.42	1219.41	618.72	Q	1491.77	1473.75	1474.74	746.38	13
11	1364.55	1346.52	1347.53	682.76	K	1363.71	1345.70	1346.68	682.35	12
12	1493.58	1475.56	1476.54	747.29	E	1235.62	1217.60	1218.58	618.31	11
13	1594.62	1576.61	1577.59	797.81	T	1106.57	1088.56	1089.54	553.78	10
14	1722.68	1704.67	1705.65	861.84	Q	1005.54	987.51	988.50	503.26	9
15	1851.72	1833.71	1834.69	926.37	E	877.46	859.45	860.44	439.23	8
16	1938.75	1920.73	1921.72	969.89	S	748.42	730.41	731.39	374.71	7
17	2066.81	2048.80	2049.78	1033.90	Q	661.39	643.38	644.27	331.12	6
18	2153.84	2135.83	2136.81	1077.42	S	533.33	515.32	516.30	267.16	5
19	2266.93	2248.92	2249.90	1133.96	I	446.30	428.29	429.27	223.65	4
20	2395.02	2377.01	2377.99	1198.02	K	333.21	315.20	316.19	167.11	3
21	2494.09	2476.08	2477.06	1247.56	V	205.12	187.11	188.09	103.06	2
22					S	106.05	88.04	89.02	53.52	1

Carausius-Neuropeptide-like precursor-1-5 (CNPLP-1-5)

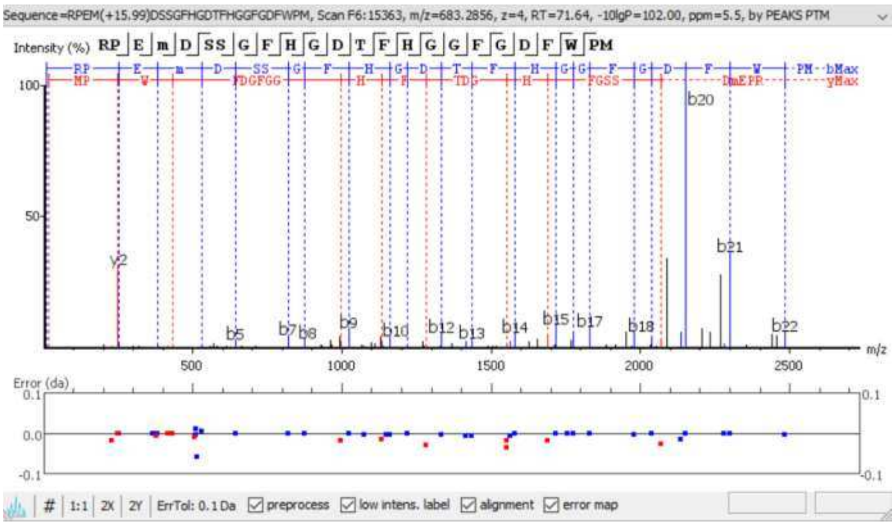

#	b	b-H2O	b-NH3	b (2+)	Seq	y	y-H2O	y-NH3	y (2+)	#
1	157.11	139.10	140.08	79.05	R					24
2	254.16	236.15	237.13	127.58	P	2574.01	2556.00	2556.99	1287.51	23
3	383.21	365.19	366.18	192.10	E	2476.96	2458.95	2459.93	1238.98	22
4	530.23	512.22	513.27	265.62	M(+15.99)	2347.92	2329.91	2330.89	1174.46	21
5	645.27	627.26	628.24	323.13	D	2200.88	2182.87	2183.85	1100.94	20
6	732.30	714.29	715.27	366.65	S	2085.85	2067.84	2068.86	1043.43	19
7	819.33	801.32	802.30	410.17	S	1998.82	1980.81	1981.80	999.91	18
8	876.35	858.34	859.33	438.68	G	1911.79	1893.78	1894.76	956.40	17
9	1023.42	1005.41	1006.39	512.22	F	1854.77	1836.76	1837.74	927.88	16
10	1160.48	1142.47	1143.45	580.74	H	1707.70	1689.71	1690.67	854.35	15
11	1217.50	1199.49	1200.47	609.25	G	1570.64	1552.65	1553.65	785.82	14
12	1332.53	1314.52	1315.50	666.76	D	1513.62	1495.61	1496.59	757.31	13
13	1433.58	1415.57	1416.55	717.29	T	1398.59	1380.58	1381.57	699.80	12
14	1580.64	1562.64	1563.62	790.82	F	1297.55	1279.54	1280.55	649.27	11
15	1717.70	1699.69	1700.68	859.35	H	1150.48	1132.48	1133.45	575.74	10
16	1774.73	1756.71	1757.70	887.86	G	1013.42	995.43	996.39	507.22	9
17	1831.75	1813.74	1814.72	916.37	G	956.40	938.39	939.37	478.70	8
18	1978.82	1960.80	1961.79	989.91	F	899.38	881.37	882.35	450.19	7
19	2035.83	2017.83	2018.81	1018.42	G	752.31	734.30	735.28	376.66	6
20	2150.86	2132.85	2133.85	1075.94	D	695.29	677.28	678.26	348.14	5
21	2297.93	2279.92	2280.90	1149.47	F	580.26	562.25	563.23	290.63	4
22	2484.01	2466.00	2466.98	1242.51	W	433.19	415.18	416.16	217.10	3
23	2581.06	2563.05	2564.04	1291.03	P	247.11	229.12	230.08	124.06	2
24					M	150.06	132.05	133.03	75.53	1

Carausius-Neuropeptide-like precursor-1-6 (CNPLP-1-6)

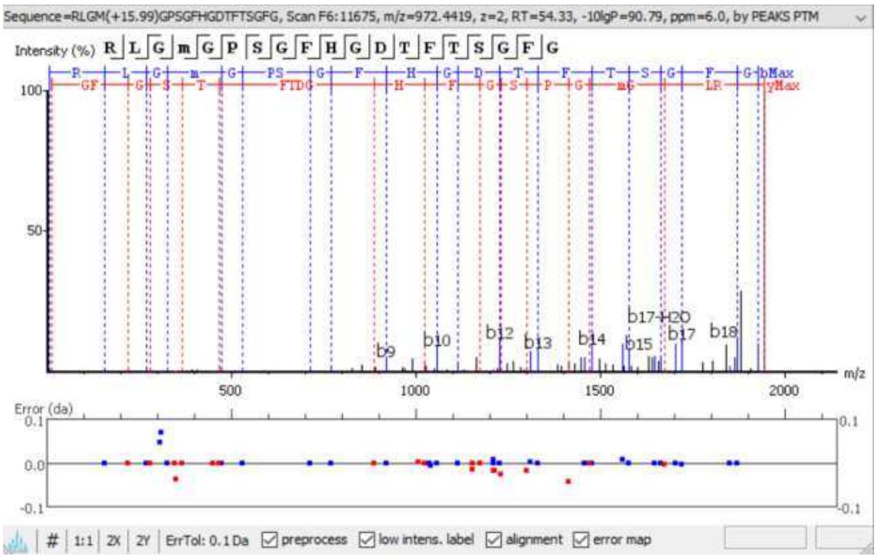

#	b	b-H2O	b-NH3	b (2+)	Seq	y	y-H2O	y-NH3	y (2+)	#
1	157.11	139.10	140.08	79.05	R					19
2	270.19	252.18	253.17	135.60	L	1787.77	1769.76	1770.74	894.38	18
3	327.21	309.16	310.11	164.11	G	1674.69	1656.67	1657.66	837.84	17
4	474.25	456.24	457.22	237.62	M(+15.99)	1617.66	1599.65	1600.64	809.33	16
5	531.27	513.26	514.24	266.14	G	1470.63	1452.62	1453.60	735.81	15
6	628.32	610.31	611.30	314.66	P	1413.65	1395.60	1396.58	707.30	14
7	715.36	697.35	698.33	358.18	S	1316.55	1298.54	1299.55	658.78	13
8	772.38	754.37	755.35	386.69	G	1229.55	1211.53	1212.51	615.26	12
9	919.45	901.44	902.42	460.22	F	1172.50	1154.49	1155.49	586.75	11
10	1056.51	1038.50	1039.48	528.75	H	1025.43	1007.42	1008.41	513.22	10
11	1113.53	1095.52	1096.50	557.26	G	888.37	870.36	871.35	444.69	9
12	1228.55	1210.53	1211.53	614.78	D	831.35	813.34	814.32	416.18	8
13	1329.60	1311.59	1312.57	665.30	T	716.32	698.31	699.30	358.66	7
14	1476.67	1458.66	1459.64	738.83	F	615.28	597.27	598.25	308.14	6
15	1577.72	1559.70	1560.69	789.36	T	468.21	450.20	451.18	234.60	5
16	1664.75	1646.74	1647.72	832.87	S	367.16	349.15	350.17	184.08	4
17	1721.77	1703.76	1704.74	861.39	G	280.13	262.12	263.10	140.56	3
18	1868.84	1850.83	1851.81	934.92	F	223.11	205.10	206.08	112.05	2
19					G	76.04	58.03	59.01	38.52	1

Carausius-Neuropeptide-like precursor-1-7 (CNPLP-1-7)

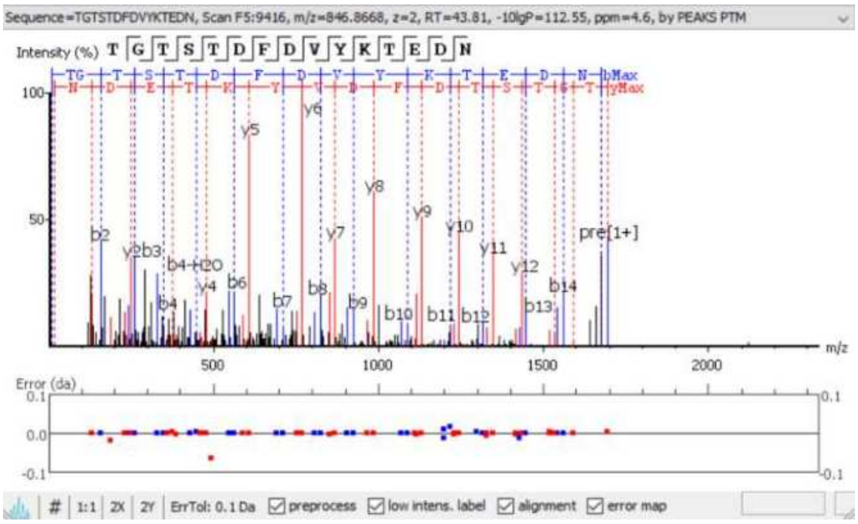

#	b	b-H2O	b-NH3	b (2+)	Seq	y	y-H2O	y-NH3	y (2+)	#
1	102.06	84.04	85.03	51.53	T					15
2	159.08	141.07	142.05	80.04	G	1591.68	1573.67	1574.65	796.34	14
3	260.12	242.11	243.10	130.56	T	1534.65	1516.64	1517.63	767.83	13
4	347.16	329.15	330.13	174.08	S	1433.61	1415.60	1416.59	717.30	12
5	448.20	430.19	431.18	224.60	T	1346.57	1328.57	1329.55	673.79	11
6	563.23	545.22	546.20	282.12	D	1245.53	1227.52	1228.51	623.26	10
7	710.30	692.29	693.27	355.65	F	1130.50	1112.49	1113.48	565.75	9
8	825.33	807.32	808.30	413.16	D	983.43	965.42	966.41	492.28	8
9	924.40	906.38	907.37	462.70	V	868.40	850.39	851.38	434.70	7
10	1087.46	1069.45	1070.43	544.23	Y	769.34	751.33	752.31	385.17	6
11	1215.54	1197.53	1198.54	608.28	K	606.27	588.26	589.25	303.64	5
12	1316.60	1298.59	1299.57	658.80	T	478.18	460.17	461.15	239.59	4
13	1445.64	1427.64	1428.63	723.32	E	377.13	359.12	360.10	189.09	3
14	1560.67	1542.66	1543.64	780.84	D	248.09	230.08	231.06	124.54	2
15					N	133.06	115.05	116.03	67.03	1

Carausius-Neuropeptide-like precursor-1-8 (CNPLP-1-8)

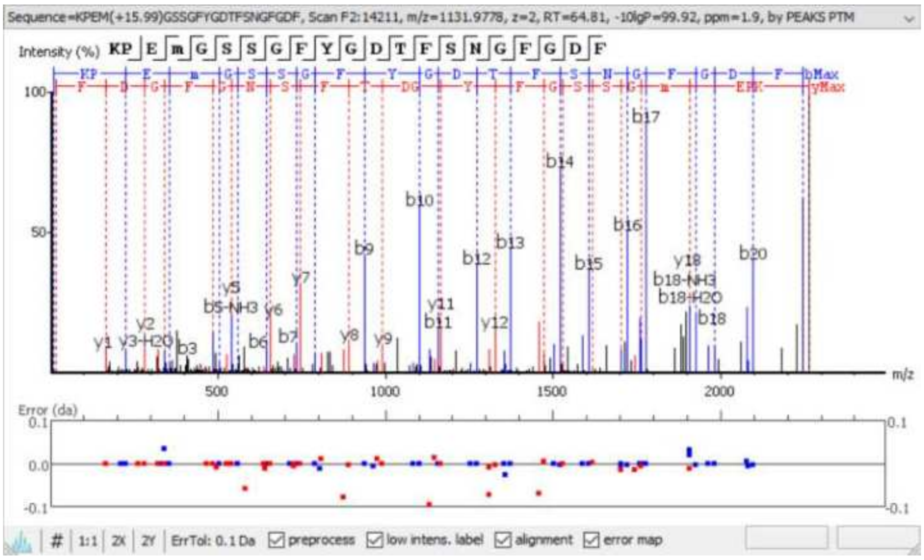

#	b	b-H2O	b-NH3	b (2+)	Seq	y	y-H2O	y-NH3	y (2+)	#
1	129.10	111.09	112.08	65.05	K					21
2	226.16	208.14	209.13	113.58	P	2134.83	2116.82	2117.81	1067.92	20
3	355.20	337.19	338.14	178.10	E	2037.78	2019.77	2020.75	1019.39	19
4	502.23	484.22	485.20	251.62	M(+15.99)	1908.75	1890.73	1891.71	954.87	18
5	559.25	541.24	542.23	280.13	G	1761.71	1743.71	1744.69	881.35	17
6	646.29	628.28	629.26	323.64	S	1704.70	1686.67	1687.65	852.84	16
7	733.32	715.31	716.29	367.16	S	1617.64	1599.64	1600.62	809.31	15
8	790.34	772.33	773.31	395.67	G	1530.62	1512.61	1513.59	765.81	14
9	937.41	919.40	920.38	469.20	F	1473.59	1455.59	1456.64	737.30	13
10	1100.47	1082.46	1083.45	550.74	Y	1326.53	1308.53	1309.57	663.76	12
11	1157.49	1139.48	1140.47	579.25	G	1163.47	1145.44	1146.44	582.29	11
12	1272.52	1254.51	1255.49	636.76	D	1106.44	1088.43	1089.42	553.72	10
13	1373.57	1355.56	1356.57	687.28	T	991.41	973.39	974.39	496.22	9
14	1520.64	1502.63	1503.61	760.82	F	890.37	872.36	873.42	445.68	8
15	1607.67	1589.66	1590.64	804.35	S	743.30	725.29	726.28	372.15	7
16	1721.72	1703.70	1704.70	861.36	N	656.27	638.26	639.25	328.63	6
17	1778.73	1760.72	1761.71	889.87	G	542.23	524.22	525.20	271.61	5
18	1925.81	1907.76	1908.75	963.41	F	485.20	467.19	468.18	243.10	4
19	1982.82	1964.81	1965.80	991.91	G	338.14	320.12	321.11	169.57	3
20	2097.85	2079.83	2080.83	1049.42	D	281.11	263.10	264.09	141.06	2
21					F	166.09	148.08	149.06	83.54	1

Carausius-Neuropeptide-like precursor-1-9 (CNPLP-1-9)

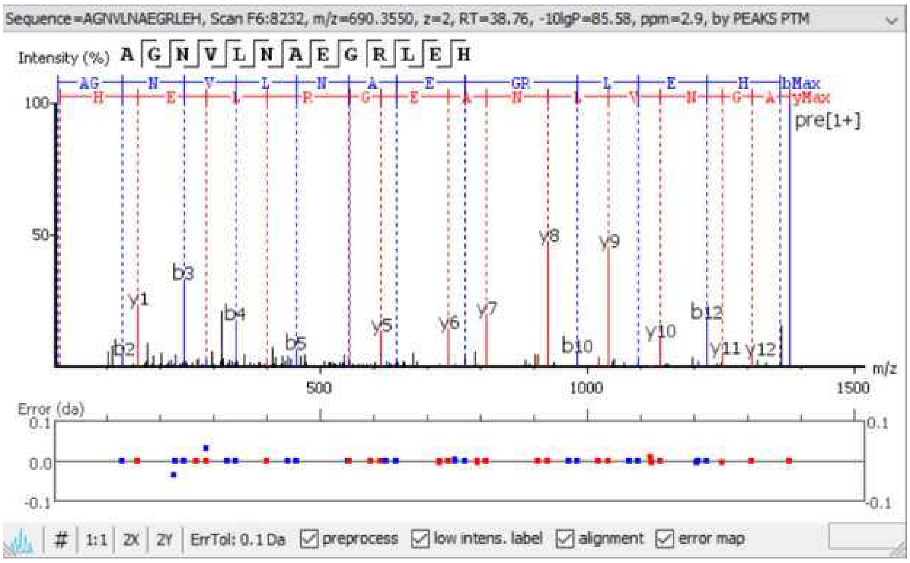

#	b	b-H2O	b-NH3	b (2+)	Seq	y	y-H2O	y-NH3	y (2+)	#
1	72.04	54.03	55.02	36.52	A					13
2	129.07	111.06	112.04	65.03	G	1308.66	1290.65	1291.64	654.83	12
3	243.11	225.10	226.12	122.05	N	1251.65	1233.63	1234.62	626.32	11
4	342.18	324.17	325.15	171.59	V	1137.60	1119.58	1120.58	569.30	10
5	455.26	437.25	438.23	228.13	L	1038.53	1020.52	1021.51	519.77	9
6	569.30	551.29	552.28	285.12	N	925.45	907.44	908.42	463.22	8
7	640.34	622.33	623.32	320.67	A	811.41	793.40	794.38	406.20	7
8	769.38	751.37	752.36	385.19	E	740.37	722.36	723.35	370.68	6
9	826.41	808.40	809.38	413.70	G	611.33	593.32	594.30	306.16	5
10	982.51	964.50	965.48	491.75	R	554.31	536.29	537.28	277.65	4
11	1095.59	1077.58	1078.56	548.30	L	398.20	380.19	381.18	199.60	3
12	1224.63	1206.63	1207.61	612.82	E	285.12	267.11	268.09	143.06	2
13					H	156.08	138.07	139.05	78.54	1

Carausius-Neuropeptide-like precursor-1-10 (CNPLP-1-10)

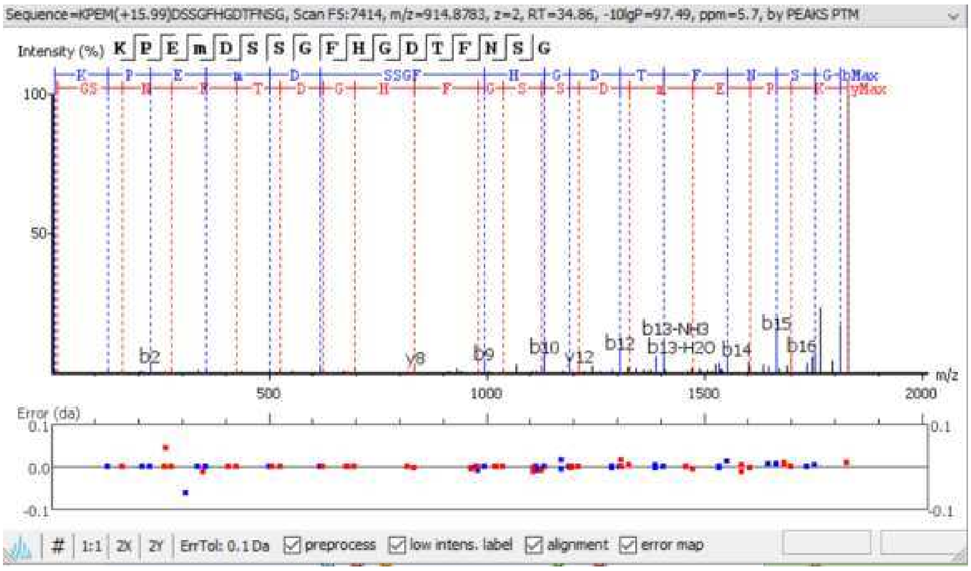

#	b	b-H2O	b-NH3	b (2+)	Seq	y	y-H2O	y-NH3	y (2+)	#
1	157.11	139.10	140.08	79.05	R					21
2	254.16	236.15	237.13	127.58	P	2166.83	2148.82	2149.81	1083.92	20
3	383.20	365.19	366.18	192.10	E	2069.78	2051.77	2052.75	1035.39	19
4	530.24	512.23	513.21	265.62	M(+15.99)	1940.79	1922.73	1923.71	970.87	18
5	645.27	627.26	628.24	323.13	D	1793.73	1775.69	1776.68	897.35	17
6	732.30	714.29	715.27	366.65	S	1678.68	1660.67	1661.65	839.84	16
7	819.33	801.33	802.30	410.17	S	1591.64	1573.63	1574.62	796.32	15
8	876.35	858.34	859.33	438.68	G	1504.61	1486.60	1487.59	752.81	14
9	1023.42	1005.41	1006.39	512.21	F	1447.59	1429.58	1430.56	724.30	13
10	1160.48	1142.47	1143.47	580.74	H	1300.52	1282.52	1283.50	650.76	12
11	1217.50	1199.49	1200.47	609.25	G	1163.48	1145.46	1146.44	582.23	11
12	1332.53	1314.52	1315.50	666.76	D	1106.44	1088.43	1089.42	553.72	10
13	1433.58	1415.57	1416.55	717.29	T	991.42	973.40	974.39	496.21	9
14	1580.65	1562.64	1563.62	790.82	F	890.37	872.36	873.34	445.68	8
15	1694.69	1676.68	1677.66	847.84	N	743.30	725.29	726.27	372.15	7
16	1781.72	1763.71	1764.69	891.36	S	629.26	611.25	612.23	315.13	6
17	1838.74	1820.73	1821.73	919.87	G	542.22	524.21	525.20	271.61	5
18	1985.81	1967.79	1968.78	993.40	F	485.20	467.19	468.18	243.13	4
19	2042.83	2024.81	2025.80	1021.92	G	338.13	320.12	321.11	169.57	3
20	2157.86	2139.84	2140.84	1079.43	D	281.11	263.10	264.09	141.06	2
21					F	166.09	148.08	149.06	83.54	1

Carausius-Neuropeptide-like precursor-1-11 (CNPLP-1-11)

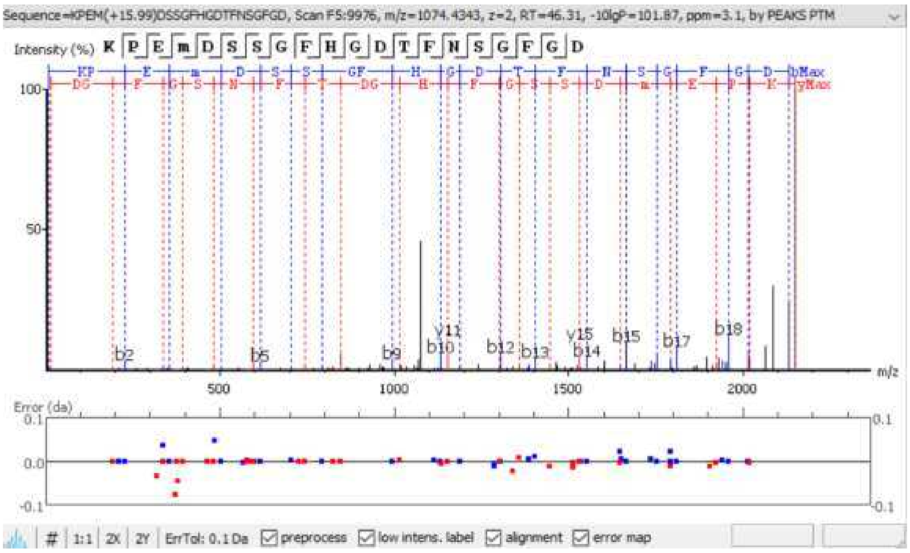

#	b	b-H2O	b-NH3	b (2+)	Seq	y	y-H2O	y-NH3	y (2+)	#
1	129.10	111.09	112.08	65.05	K					20
2	226.15	208.14	209.13	113.58	P	2019.77	2001.76	2002.74	1010.38	19
3	355.20	337.19	338.13	178.10	E	1922.72	1904.72	1905.69	961.86	18
4	502.23	484.22	485.16	251.62	M(+15.99)	1793.68	1775.66	1776.64	897.34	17
5	617.26	599.25	600.23	309.13	D	1646.64	1628.62	1629.61	823.82	16
6	704.29	686.28	687.27	352.65	S	1531.61	1513.60	1514.60	766.30	15
7	791.32	773.31	774.30	396.16	S	1444.59	1426.57	1427.55	722.79	14
8	848.35	830.34	831.32	424.67	G	1357.54	1339.56	1340.52	679.27	13
9	995.41	977.40	978.39	498.21	F	1300.52	1282.51	1283.50	650.76	12
10	1132.47	1114.46	1115.45	566.74	H	1153.45	1135.45	1136.44	577.22	11
11	1189.49	1171.48	1172.47	595.25	G	1016.39	998.38	999.37	508.70	10
12	1304.52	1286.52	1287.51	652.76	D	959.37	941.36	942.35	480.19	9
13	1405.56	1387.55	1388.54	703.28	T	844.35	826.34	827.32	422.67	8
14	1552.64	1534.63	1535.61	776.82	F	743.30	725.29	726.27	372.23	7
15	1666.68	1648.64	1649.65	833.84	N	596.23	578.22	579.20	298.62	6
16	1753.71	1735.70	1736.69	877.36	S	482.19	464.18	465.16	241.59	5
17	1810.73	1792.72	1793.68	905.87	G	395.16	377.15	378.18	198.08	4
18	1957.80	1939.79	1940.78	979.40	F	338.13	320.16	321.11	169.57	3
19	2014.83	1996.81	1997.80	1007.91	G	191.07	173.06	174.04	96.03	2
20					D	134.04	116.03	117.02	67.52	1

Carausius-Neuropeptide-like precursor-1-12 (CNPLP-1-12)

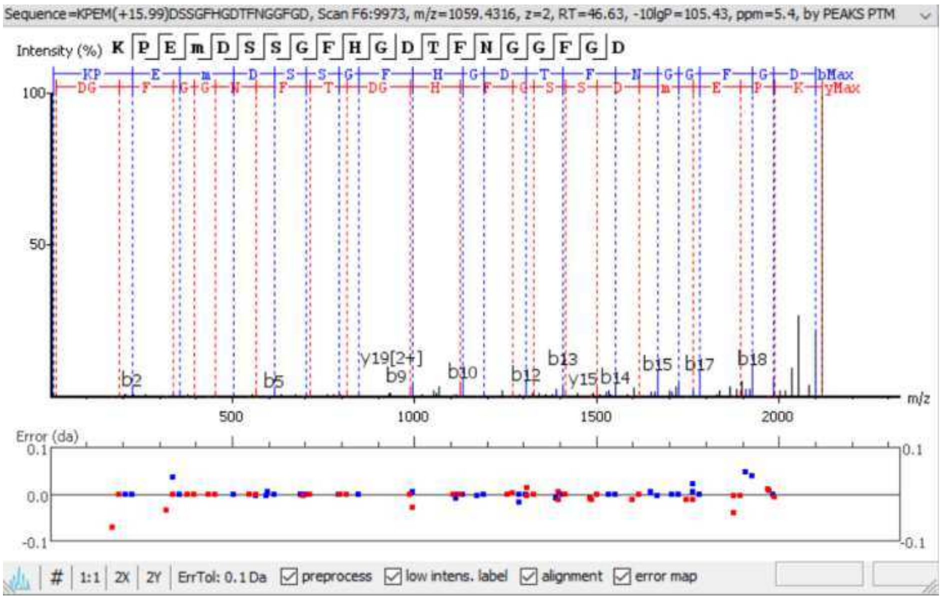

#	b	b-H2O	b-NH3	b (2+)	Seq	y	y-H2O	y-NH3	y (2+)	#
1	129.10	111.09	112.08	65.05	K					20
2	226.15	208.14	209.13	113.58	P	1989.76	1971.73	1972.72	995.41	19
3	355.20	337.19	338.13	178.10	E	1892.71	1874.70	1875.72	946.85	18
4	502.23	484.22	485.21	251.62	M(+15.99)	1763.67	1745.65	1746.65	882.33	17
5	617.26	599.24	600.23	309.13	D	1616.63	1598.63	1599.61	808.81	16
6	704.29	686.28	687.27	352.65	S	1501.60	1483.60	1484.58	751.30	15
7	791.32	773.31	774.30	396.16	S	1414.57	1396.55	1397.55	707.78	14
8	848.35	830.34	831.32	424.67	G	1327.53	1309.51	1310.51	664.27	13
9	995.41	977.40	978.39	498.21	F	1270.51	1252.50	1253.49	635.76	12
10	1132.47	1114.46	1115.46	566.74	H	1123.45	1105.43	1106.42	562.22	11
11	1189.49	1171.49	1172.47	595.25	G	986.39	968.37	969.36	493.69	10
12	1304.52	1286.51	1287.51	652.76	D	929.36	911.35	912.34	465.18	9
13	1405.57	1387.57	1388.55	703.28	T	814.34	796.33	797.31	407.67	8
14	1552.64	1534.63	1535.61	776.82	F	713.29	695.28	696.27	357.14	7
15	1666.69	1648.66	1649.65	833.84	N	566.22	548.21	549.19	283.61	6
16	1723.70	1705.69	1706.68	862.35	G	452.18	434.17	435.15	226.59	5
17	1780.73	1762.71	1763.67	890.86	G	395.16	377.14	378.13	198.08	4
18	1927.75	1909.73	1910.77	964.40	F	338.13	320.16	321.11	169.57	3
19	1984.81	1966.80	1967.79	992.91	G	191.07	173.13	174.04	96.03	2
20					D	134.04	116.03	117.02	67.52	1

Carausius-Neuropeptide-like precursor-1-13 (CNPLP-1-13)

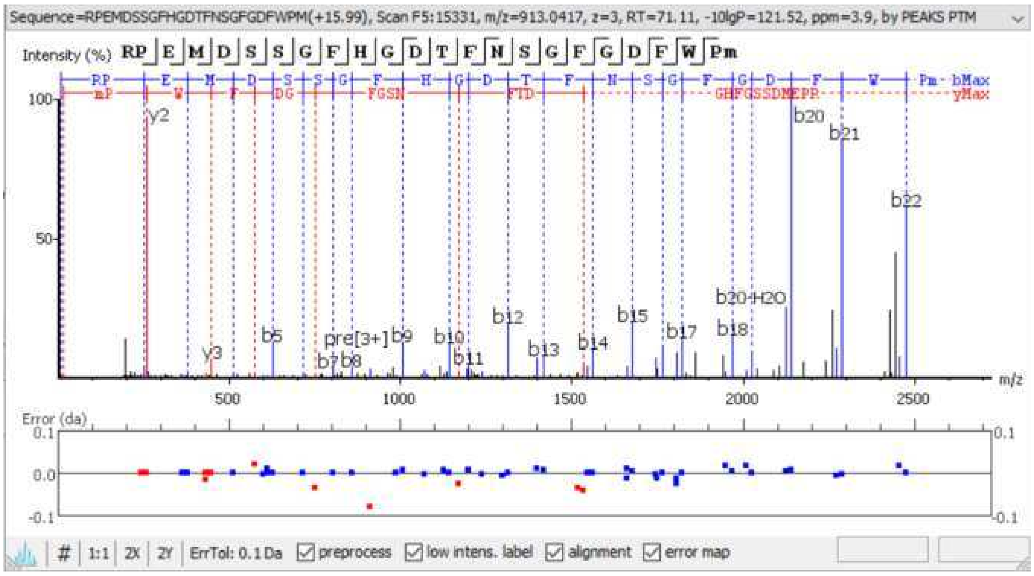

#	b	b-H2O	b-NH3	b (2+)	Seq	y	y-H2O	y-NH3	y (2+)	#
1	157.11	139.10	140.08	79.05	R					24
2	254.16	236.15	237.13	127.58	P	2581.01	2563.00	2563.98	1291.00	23
3	383.20	365.19	366.18	192.10	E	2483.95	2465.94	2466.93	1242.48	22
4	514.24	496.23	497.22	257.62	M	2354.91	2336.90	2337.88	1177.96	21
5	629.27	611.25	612.24	315.14	D	2223.87	2205.86	2206.84	1112.44	20
6	716.30	698.29	699.28	358.65	S	2108.84	2090.83	2091.82	1054.92	19
7	803.34	785.33	786.31	402.17	S	2021.81	2003.80	2004.79	1011.41	18
8	860.36	842.35	843.33	430.68	G	1934.78	1916.77	1917.75	967.89	17
9	1007.42	989.41	990.40	504.21	F	1877.76	1859.75	1860.73	939.38	16
10	1144.48	1126.47	1127.45	572.74	H	1730.69	1712.68	1713.66	865.85	15
11	1201.50	1183.50	1184.48	601.26	G	1593.63	1575.62	1576.60	797.32	14
12	1316.53	1298.52	1299.52	658.77	D	1536.65	1518.64	1519.58	768.80	13
13	1417.57	1399.56	1400.55	709.29	T	1421.58	1403.57	1404.56	711.29	12
14	1564.65	1546.64	1547.62	782.82	F	1320.54	1302.52	1303.51	660.77	11
15	1678.69	1660.67	1661.68	839.85	N	1173.49	1155.46	1156.44	587.23	10
16	1765.72	1747.72	1748.71	883.36	S	1059.42	1041.41	1042.40	530.21	9
17	1822.75	1804.76	1805.73	911.87	G	972.39	954.38	955.36	486.70	8
18	1969.81	1951.79	1952.79	985.41	F	915.37	897.36	898.34	458.19	7
19	2026.83	2008.81	2009.81	1013.92	G	768.30	750.29	751.31	384.65	6
20	2141.85	2123.85	2124.84	1071.43	D	711.28	693.27	694.25	356.14	5
21	2288.94	2270.92	2271.91	1144.97	F	596.25	578.22	579.23	298.63	4
22	2475.01	2456.98	2457.98	1238.01	W	449.19	431.19	432.16	225.09	3
23	2572.06	2554.05	2555.04	1286.53	P	263.11	245.10	246.08	132.05	2
24					M(+15.99)	166.05	148.04	149.03	83.53	1

Carausius-Neuropeptide-like precursor-1-14 (CNPLP-1-14)

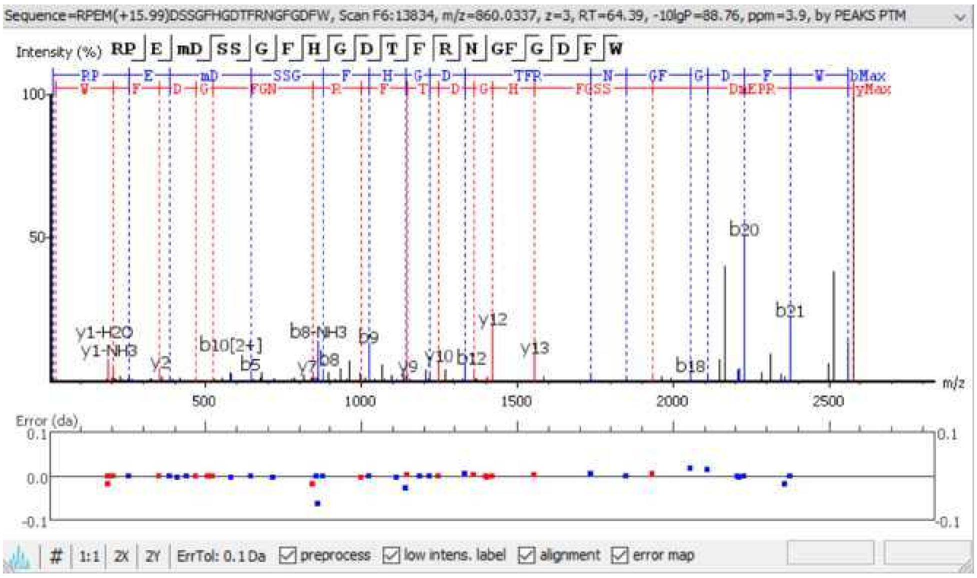

#	b	b-H2O	b-NH3	b (2+)	Seq	y	y-H2O	y-NH3	y (2+)	#
1	157.11	139.10	140.08	79.05	R					22
2	254.16	236.15	237.13	127.58	P	2421.98	2403.97	2404.96	1211.49	21
3	383.20	365.19	366.18	192.10	E	2324.93	2306.92	2307.90	1162.97	20
4	530.24	512.23	513.21	265.62	M(+15.99)	2195.89	2177.88	2178.86	1098.44	19
5	645.26	627.26	628.24	323.13	D	2048.85	2030.84	2031.83	1024.93	18
6	732.30	714.29	715.27	366.65	S	1933.82	1915.81	1916.80	967.41	17
7	819.33	801.32	802.30	410.17	S	1846.79	1828.78	1829.77	923.90	16
8	876.35	858.34	859.39	438.68	G	1759.76	1741.75	1742.73	880.38	15
9	1023.42	1005.41	1006.39	512.21	F	1702.74	1684.73	1685.71	851.87	14
10	1160.48	1142.50	1143.45	580.74	H	1555.67	1537.66	1538.64	778.34	13
11	1217.50	1199.49	1200.47	609.25	G	1418.61	1400.60	1401.59	709.81	12
12	1332.52	1314.52	1315.50	666.76	D	1361.59	1343.58	1344.56	681.30	11
13	1433.58	1415.57	1416.55	717.29	T	1246.56	1228.55	1229.54	623.78	10
14	1580.64	1562.63	1563.62	790.82	F	1145.51	1127.51	1128.49	573.26	9
15	1736.74	1718.73	1719.72	868.87	R	998.45	980.44	981.42	499.72	8
16	1850.79	1832.78	1833.76	925.89	N	842.37	824.34	825.32	421.67	7
17	1907.81	1889.80	1890.78	954.40	G	728.30	710.29	711.28	364.65	6
18	2054.86	2036.87	2037.85	1027.94	F	671.28	653.27	654.26	336.14	5
19	2111.88	2093.89	2094.87	1056.45	G	524.21	506.20	507.19	262.61	4
20	2226.93	2208.91	2209.90	1113.97	D	467.19	449.18	450.17	234.10	3
21	2374.00	2355.98	2356.99	1187.50	F	352.17	334.15	335.14	176.58	2
22					W	205.10	187.11	188.07	103.05	1

NVP-like (NVP-1)

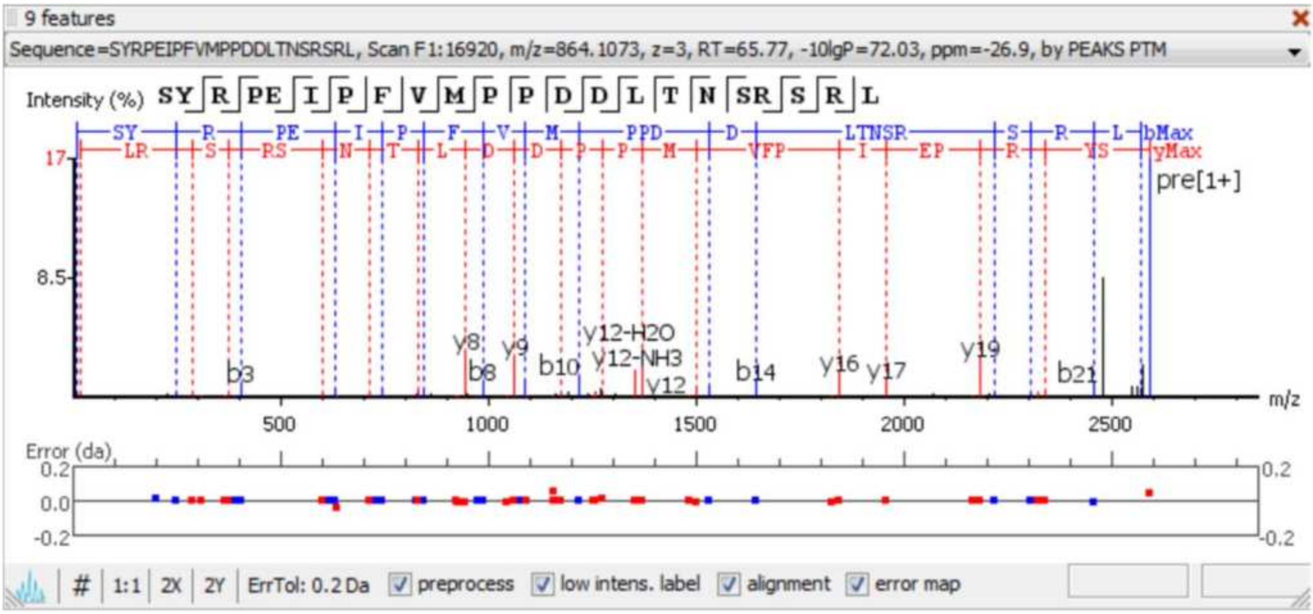

#	b	b-H2O	b-NH3	b (2+)	Seq	y	y-H2O	y-NH3	y (2+)	#
1	88.04	70.03	71.01	44.52	S					22
2	251.10	233.09	234.08	126.05	Y	2503.28	2485.27	2486.25	1252.14	21
3	407.21	389.19	390.18	204.09	R	2340.22	2322.20	2323.19	1170.61	20
4	504.26	486.25	487.23	252.63	P	2184.12	2166.10	2167.08	1092.57	19
5	633.30	615.29	616.28	317.15	E	2087.06	2069.05	2070.03	1044.03	18
6	746.39	728.37	729.36	373.69	I	1958.03	1940.01	1940.99	979.51	17
7	843.44	825.43	826.42	422.22	P	1844.94	1826.92	1827.92	922.97	16
8	990.51	972.49	973.48	495.75	F	1747.88	1729.87	1730.85	874.44	15
9	1089.58	1071.57	1072.55	545.29	V	1600.81	1582.80	1583.78	800.91	14
10	1220.62	1202.60	1203.59	610.81	M	1501.76	1483.73	1484.71	751.37	13
11	1317.67	1299.66	1300.64	659.33	P	1370.71	1352.69	1353.68	685.85	12
12	1414.72	1396.71	1397.69	707.86	P	1273.64	1255.64	1256.63	637.37	11
13	1529.75	1511.74	1512.72	765.37	D	1176.59	1158.53	1159.56	588.80	10
14	1644.78	1626.76	1627.75	822.89	D	1061.58	1043.56	1044.55	531.28	9
15	1757.86	1739.85	1740.83	879.43	L	946.56	928.53	929.53	473.77	8
16	1858.90	1840.89	1841.88	929.95	T	833.46	815.45	816.43	417.23	7
17	1972.95	1954.94	1955.92	986.97	N	732.41	714.40	715.39	366.71	6
18	2059.98	2041.97	2042.95	1030.49	S	618.37	600.36	601.35	309.69	5
19	2216.07	2198.07	2199.05	1108.54	R	531.34	513.33	514.31	266.17	4
20	2303.11	2285.10	2286.09	1152.06	S	375.24	357.22	358.21	188.12	3
21	2459.23	2441.20	2442.19	1230.11	R	288.20	270.19	271.18	144.60	2
22					L	132.10	114.09	115.07	66.55	1

NVP-like (NVP-2)

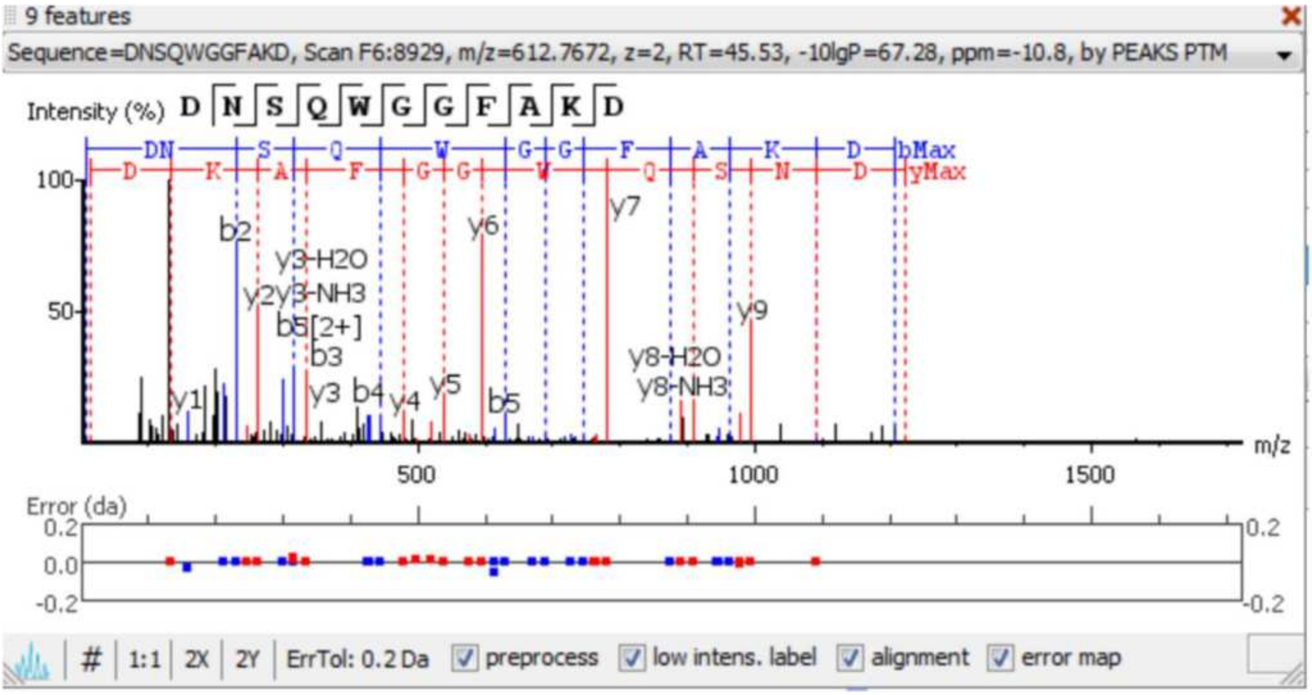

#	b	b-H2O	b-NH3	b (2+)	Seq	y	y-H2O	y-NH3	y (2+)	#
1	116.03	98.02	99.01	58.52	D					11
2	230.08	212.07	213.05	115.54	N	1109.50	1091.49	1092.47	555.25	10
3	317.11	299.10	300.08	159.09	S	995.46	977.45	978.45	498.21	9
4	445.17	427.16	428.14	223.08	Q	908.43	890.42	891.40	454.71	8
5	631.25	613.30	614.22	316.13	W	780.37	762.36	763.34	390.68	7
6	688.27	670.26	671.24	344.63	G	594.29	576.28	577.26	297.64	6
7	745.29	727.28	728.27	373.15	G	537.27	519.23	520.24	269.13	5
8	892.36	874.35	875.33	446.68	F	480.25	462.23	463.22	240.62	4
9	963.40	945.38	946.38	482.20	A	333.18	315.15	316.13	167.09	3
10	1091.49	1073.48	1074.46	546.25	K	262.14	244.13	245.11	131.57	2
11					D	134.04	116.03	117.02	67.52	1

NVP-like (NVP-3)_[pQ]

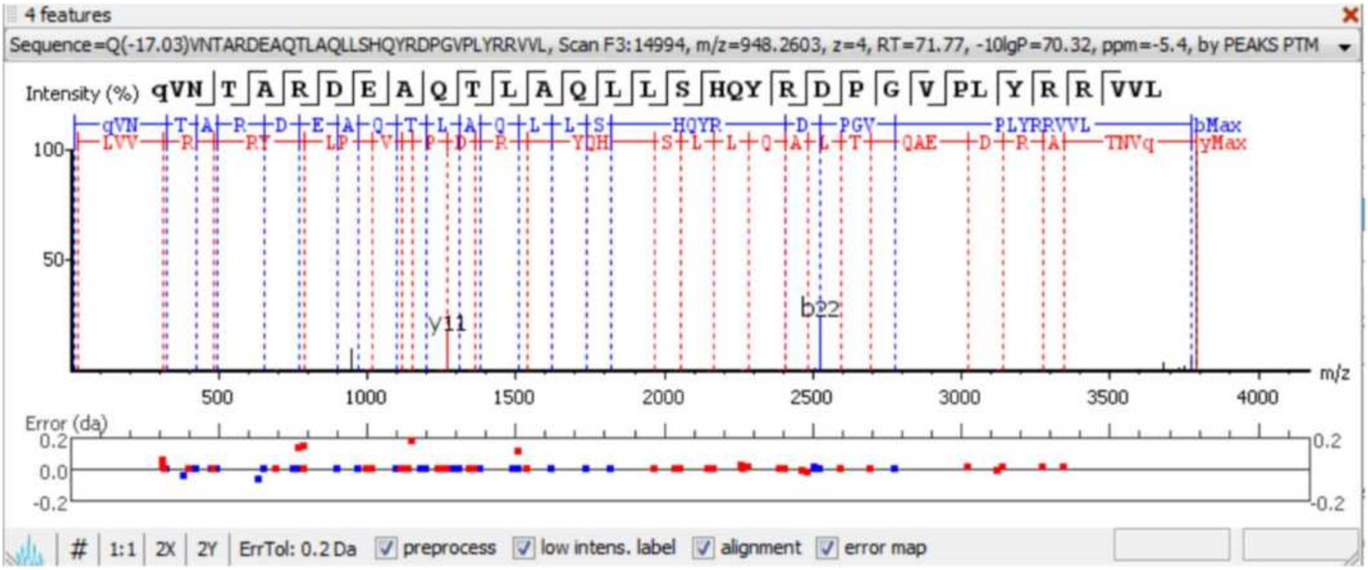

#	b	b-H2O	b-NH3	b (2+)	Seq	y	y-H2O	y-NH3	y (2+)	#
1	112.04	94.03	95.01	56.52	Q(-17.03)					33
2	211.11	193.10	194.08	106.05	V	3678.98	3660.97	3661.96	1839.99	32
3	325.15	307.14	308.16	163.08	N	3579.91	3561.90	3562.89	1790.46	31
4	426.20	408.19	409.17	213.60	T	3465.87	3447.86	3448.84	1733.44	30
5	497.24	479.22	480.21	249.12	A	3364.81	3346.81	3347.80	1682.91	29
6	653.34	635.33	636.31	327.17	R	3293.79	3275.78	3276.76	1647.39	28
7	768.36	750.35	751.34	384.68	D	3137.69	3119.67	3120.66	1569.34	27
8	897.41	879.39	880.38	449.20	E	3022.66	3004.65	3005.63	1511.83	26
9	968.44	950.43	951.43	484.72	A	2893.60	2875.61	2876.59	1447.31	25
10	1096.50	1078.48	1079.49	548.75	Q	2822.58	2804.56	2805.56	1411.79	24
11	1197.55	1179.54	1180.52	599.27	T	2694.52	2676.51	2677.50	1347.76	23
12	1310.63	1292.62	1293.61	655.82	L	2593.47	2575.46	2576.45	1297.24	22
13	1381.67	1363.66	1364.65	691.34	A	2480.40	2462.38	2463.36	1240.70	21
14	1509.73	1491.72	1492.71	755.36	Q	2409.35	2391.33	2392.33	1205.18	20
15	1622.82	1604.80	1605.79	811.91	L	2281.28	2263.28	2264.29	1141.16	19
16	1735.90	1717.89	1718.87	868.45	L	2168.21	2150.20	2151.18	1084.62	18
17	1822.93	1804.92	1805.90	911.96	S	2055.12	2037.11	2038.09	1028.06	17
18	1959.99	1941.98	1942.96	980.49	H	1968.09	1950.08	1951.07	984.55	16
19	2088.05	2070.04	2071.02	1044.52	Q	1831.03	1813.02	1814.01	916.02	15
20	2251.11	2233.10	2234.08	1126.06	Y	1702.98	1684.96	1685.95	851.99	14
21	2407.21	2389.20	2390.18	1204.11	R	1539.91	1521.90	1522.88	770.46	13
22	2522.24	2504.23	2505.21	1261.62	D	1383.81	1365.80	1366.78	692.41	12
23	2619.29	2601.28	2602.26	1310.15	P	1268.78	1250.58	1251.75	634.89	11
24	2676.31	2658.30	2659.29	1338.66	G	1171.73	1153.52	1154.70	586.37	10
25	2775.38	2757.37	2758.35	1388.19	V	1114.71	1096.50	1097.68	557.85	9
26	2872.43	2854.42	2855.41	1436.72	P	1015.64	997.63	998.62	508.32	8
27	2985.52	2967.51	2968.49	1493.26	L	918.59	900.43	901.56	459.79	7
28	3148.58	3130.57	3131.55	1574.79	Y	805.50	787.49	788.48	403.25	6
29	3304.68	3286.67	3287.66	1652.84	R	642.44	624.43	625.41	321.72	5
30	3460.78	3442.77	3443.76	1730.89	R	486.34	468.33	469.31	243.67	4
31	3559.85	3541.84	3542.82	1780.43	V	330.24	312.23	313.19	165.62	3
32	3658.92	3640.91	3641.89	1829.96	V	231.17	213.16	214.14	116.09	2
33					L	132.10	114.09	115.07	66.55	1

NVP-like (NVP-3)_[Q]_partial

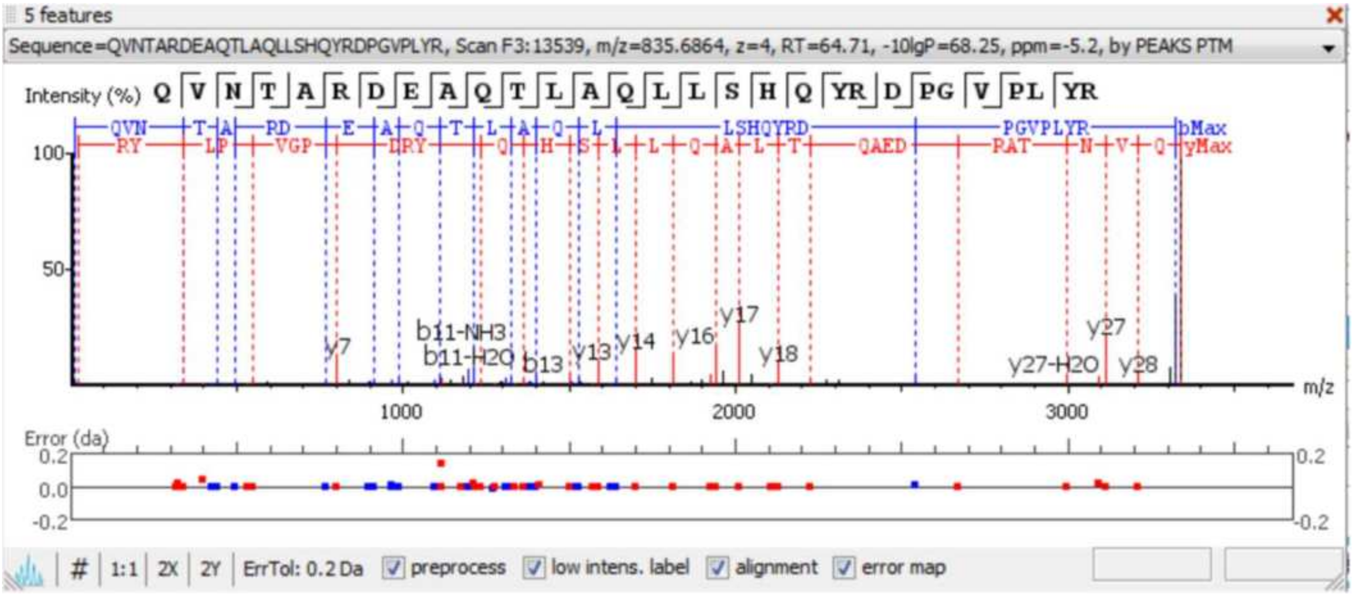

#	b	b-H2O	b-NH3	b (2+)	Seq	y	y-H2O	y-NH3	y (2+)	#
1	129.07	111.06	112.04	65.03	Q					29
2	228.13	210.12	211.11	114.57	V	3211.66	3193.65	3194.63	1606.33	28
3	342.18	324.17	325.15	171.59	N	3112.59	3094.56	3095.57	1556.80	27
4	443.23	425.21	426.20	222.11	T	2998.55	2980.54	2981.52	1499.77	26
5	514.26	496.25	497.23	257.63	A	2897.50	2879.49	2880.47	1449.25	25
6	670.36	652.35	653.34	335.68	R	2826.46	2808.45	2809.44	1413.71	24
7	785.39	767.38	768.36	393.20	D	2670.35	2652.35	2653.34	1335.69	23
8	914.43	896.42	897.41	457.72	E	2555.34	2537.33	2538.31	1278.17	22
9	985.47	967.44	968.44	493.24	A	2426.29	2408.28	2409.27	1213.62	21
10	1113.53	1095.52	1096.50	557.26	Q	2355.26	2337.25	2338.23	1178.13	20
11	1214.57	1196.57	1197.55	607.79	T	2227.19	2209.19	2210.17	1114.10	19
12	1327.66	1309.65	1310.64	664.33	L	2126.15	2108.14	2109.12	1063.58	18
13	1398.70	1380.68	1381.67	699.85	A	2013.06	1995.06	1996.04	1007.03	17
14	1526.76	1508.74	1509.72	763.88	Q	1942.03	1924.02	1925.00	971.51	16
15	1639.85	1621.83	1622.82	820.42	L	1813.97	1795.96	1796.94	907.49	15
16	1752.92	1734.91	1735.90	876.96	L	1700.88	1682.88	1683.86	850.94	14
17	1839.96	1821.95	1822.93	920.48	S	1587.80	1569.79	1570.77	794.40	13
18	1977.02	1959.00	1959.99	989.01	H	1500.77	1482.76	1483.74	750.89	12
19	2105.07	2087.06	2088.05	1053.04	Q	1363.71	1345.70	1346.68	682.36	11
20	2268.14	2250.13	2251.11	1134.57	Y	1235.65	1217.64	1218.62	618.33	10
21	2424.24	2406.23	2407.21	1212.61	R	1072.59	1054.58	1055.56	536.79	9
22	2539.25	2521.25	2522.24	1270.15	D	916.49	898.48	899.46	458.74	8
23	2636.32	2618.31	2619.29	1318.66	P	801.46	783.45	784.43	401.18	7
24	2693.34	2675.33	2676.31	1347.17	G	704.41	686.40	687.38	352.70	6
25	2792.41	2774.40	2775.38	1396.71	V	647.39	629.38	630.36	324.17	5
26	2889.46	2871.45	2872.43	1445.23	P	548.32	530.31	531.29	274.66	4
27	3002.54	2984.53	2985.52	1501.77	L	451.27	433.26	434.24	226.13	3
28	3165.61	3147.60	3148.58	1583.30	Y	338.18	320.17	321.15	169.59	2
29					R	175.12	157.11	158.09	88.06	1

NVP-like (NVP-3)_[Q]

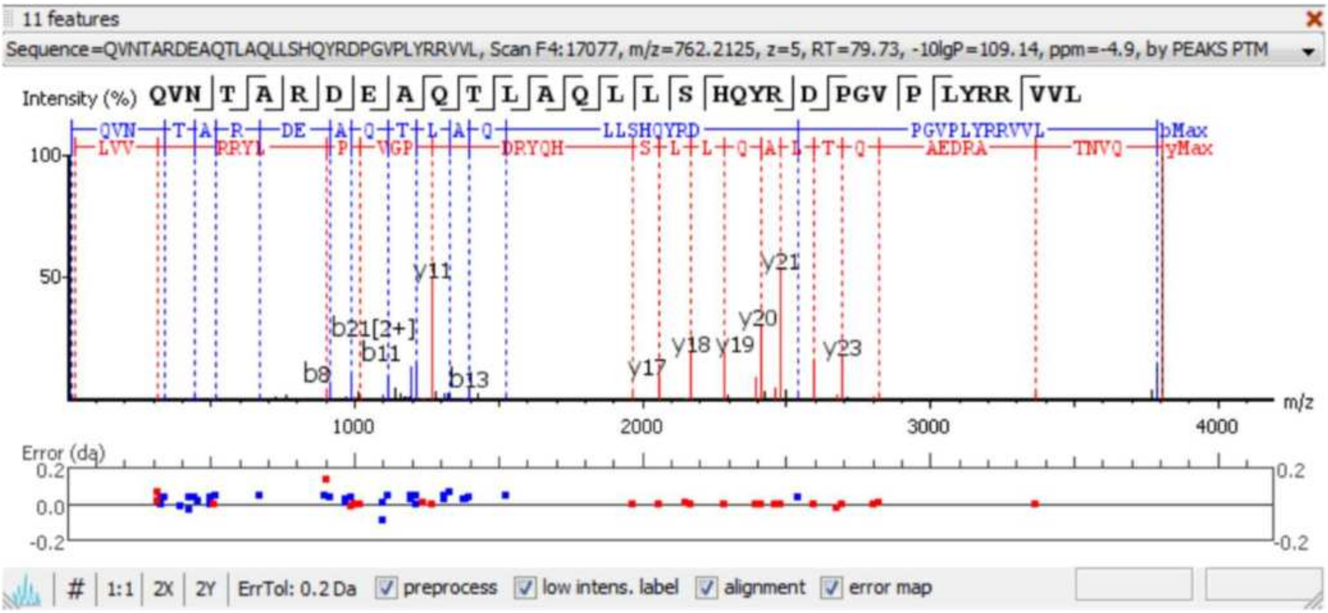

#	b	b-H2O	b-NH3	b (2+)	Seq	y	y-H2O	y-NH3	y (2+)	#
1	129.07	111.06	112.04	65.03	Q					33
2	228.13	210.12	211.11	114.57	V	3678.98	3660.97	3661.96	1839.99	32
3	342.13	324.12	325.15	171.59	N	3579.91	3561.90	3562.89	1790.46	31
4	443.18	425.17	426.24	222.11	T	3465.87	3447.86	3448.84	1733.44	30
5	514.21	496.20	497.24	257.63	A	3364.82	3346.81	3347.80	1682.91	29
6	670.31	652.35	653.34	335.68	R	3293.79	3275.78	3276.76	1647.39	28
7	785.39	767.38	768.36	393.21	D	3137.69	3119.67	3120.66	1569.34	27
8	914.39	896.37	897.41	457.70	E	3022.66	3004.65	3005.63	1511.83	26
9	985.42	967.42	968.42	493.24	A	2893.62	2875.61	2876.59	1447.31	25
10	1113.48	1095.61	1096.49	557.26	Q	2822.57	2804.57	2805.55	1411.79	24
11	1214.52	1196.51	1197.52	607.79	T	2694.51	2676.51	2677.52	1347.76	23
12	1327.59	1309.60	1310.60	664.33	L	2593.47	2575.46	2576.45	1297.24	22
13	1398.65	1380.65	1381.64	699.85	A	2480.39	2462.38	2463.36	1240.68	21
14	1526.71	1508.75	1509.73	763.88	Q	2409.35	2391.34	2392.33	1205.18	20
15	1639.84	1621.83	1622.81	820.42	L	2281.29	2263.28	2264.27	1141.15	19
16	1752.92	1734.91	1735.90	876.96	L	2168.21	2150.20	2151.17	1084.60	18
17	1839.96	1821.95	1822.93	920.48	S	2055.12	2037.11	2038.10	1028.06	17
18	1977.02	1959.00	1959.99	989.01	H	1968.09	1950.08	1951.07	984.56	16
19	2105.07	2087.06	2088.05	1053.04	Q	1831.03	1813.02	1814.01	916.02	15
20	2268.14	2250.13	2251.11	1134.57	Y	1702.98	1684.96	1685.95	851.99	14
21	2424.24	2406.23	2407.21	1212.61	R	1539.91	1521.90	1522.88	770.46	13
22	2539.22	2521.25	2522.24	1270.13	D	1383.81	1365.80	1366.78	692.41	12
23	2636.32	2618.31	2619.29	1318.66	P	1268.78	1250.77	1251.76	634.89	11
24	2693.34	2675.33	2676.31	1347.17	G	1171.73	1153.72	1154.70	586.37	10
25	2792.41	2774.40	2775.38	1396.70	V	1114.71	1096.70	1097.68	557.85	9
26	2889.46	2871.45	2872.43	1445.23	P	1015.63	997.63	998.62	508.32	8
27	3002.54	2984.53	2985.52	1501.77	L	918.59	900.43	901.56	459.79	7
28	3165.61	3147.60	3148.58	1583.30	Y	805.50	787.49	788.48	403.25	6
29	3321.71	3303.70	3304.68	1661.35	R	642.44	624.43	625.41	321.72	5
30	3477.81	3459.80	3460.78	1739.41	R	486.34	468.33	469.31	243.67	4
31	3576.88	3558.87	3559.85	1788.94	V	330.24	312.15	313.19	165.62	3
32	3675.95	3657.94	3658.92	1838.47	V	231.17	213.16	214.14	116.09	2
33					L	132.10	114.09	115.07	66.55	1

Orcokinin-A-1 (Orc-A-1)

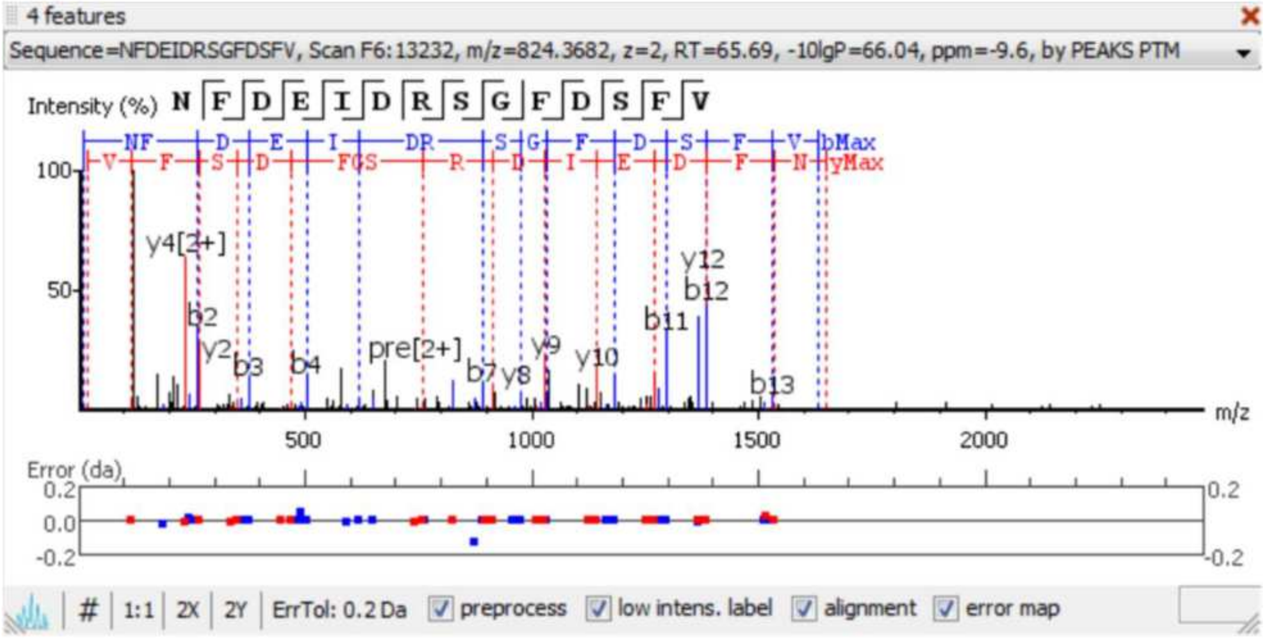

#	b	b-H2O	b-NH3	b (2+)	Seq	y	y-H2O	y-NH3	y (2+)	#
1	115.05	97.04	98.02	58.03	N					14
2	262.12	244.09	245.09	131.56	F	1533.69	1515.65	1516.66	767.34	13
3	377.15	359.14	360.12	189.10	D	1386.62	1368.60	1369.59	693.81	12
4	506.19	488.18	489.16	253.59	E	1271.59	1253.59	1254.56	636.30	11
5	619.27	601.26	602.25	310.14	I	1142.55	1124.53	1125.52	571.77	10
6	734.30	716.29	717.27	367.65	D	1029.46	1011.45	1012.44	515.23	9
7	890.40	872.39	873.51	445.70	R	914.44	896.43	897.42	457.72	8
8	977.43	959.43	960.41	489.16	S	758.34	740.32	741.33	379.67	7
9	1034.45	1016.44	1017.43	517.73	G	671.30	653.29	654.28	336.17	6
10	1181.52	1163.50	1164.50	591.27	F	614.28	596.27	597.26	307.64	5
11	1296.55	1278.54	1279.52	648.78	D	467.21	449.20	450.19	234.12	4
12	1383.58	1365.57	1366.57	692.29	S	352.19	334.18	335.16	176.59	3
13	1530.65	1512.65	1513.63	765.83	F	265.15	247.14	248.13	133.08	2
14					V	118.09	100.08	101.06	59.54	1

Orcokinin-A-2 (Orc-A-2)

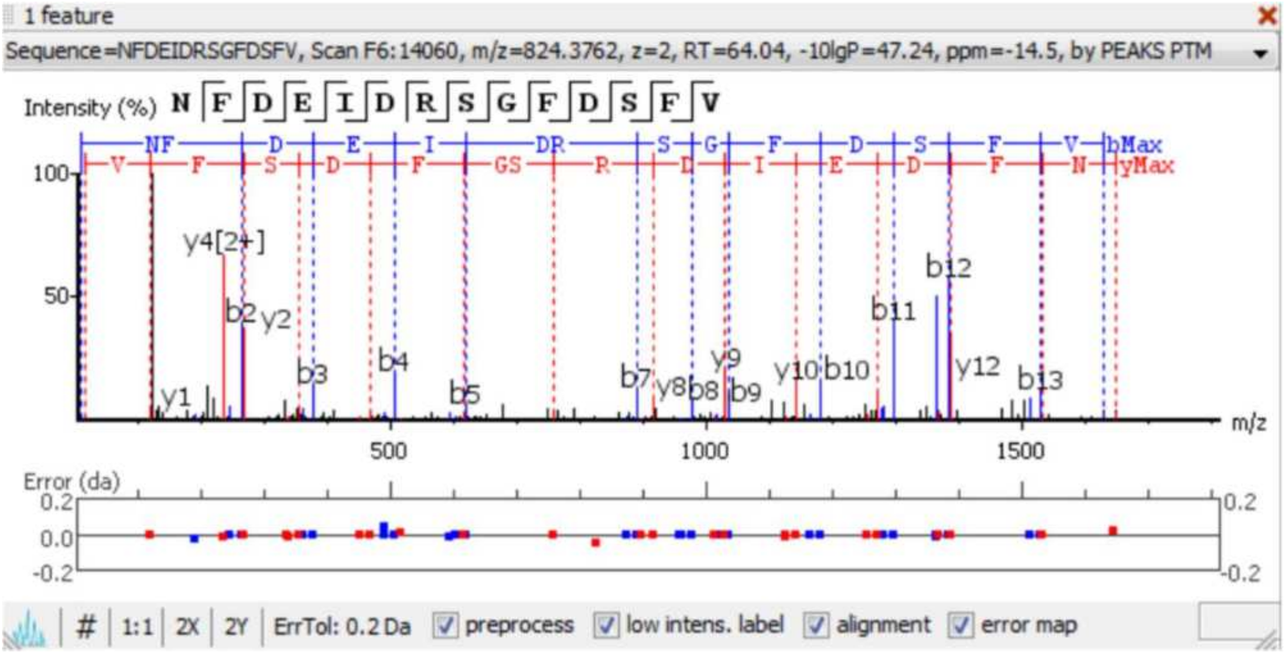

#	b	b-H2O	b-NH3	b (2+)	Seq	y	y-H2O	y-NH3	y (2+)	#
1	115.05	97.04	98.02	58.03	N					14
2	262.12	244.11	245.09	131.56	F	1533.68	1515.68	1516.66	767.34	13
3	377.15	359.14	360.12	189.10	D	1386.62	1368.60	1369.59	693.81	12
4	506.19	488.18	489.16	253.59	E	1271.59	1253.59	1254.56	636.30	11
5	619.27	601.26	602.25	310.14	I	1142.55	1124.54	1125.54	571.77	10
6	734.30	716.29	717.27	367.65	D	1029.47	1011.45	1012.44	515.21	9
7	890.40	872.39	873.38	445.70	R	914.44	896.43	897.41	457.72	8
8	977.43	959.43	960.41	489.16	S	758.34	740.32	741.31	379.67	7
9	1034.45	1016.45	1017.43	517.73	G	671.30	653.29	654.28	336.17	6
10	1181.52	1163.51	1164.50	591.27	F	614.28	596.27	597.26	307.64	5
11	1296.55	1278.54	1279.53	648.77	D	467.21	449.20	450.19	234.12	4
12	1383.58	1365.57	1366.57	692.29	S	352.19	334.18	335.16	176.59	3
13	1530.65	1512.63	1513.63	765.83	F	265.15	247.14	248.13	133.08	2
14					V	118.09	100.08	101.06	59.54	1

Orcokinin-A-3 (Orc-A-3)

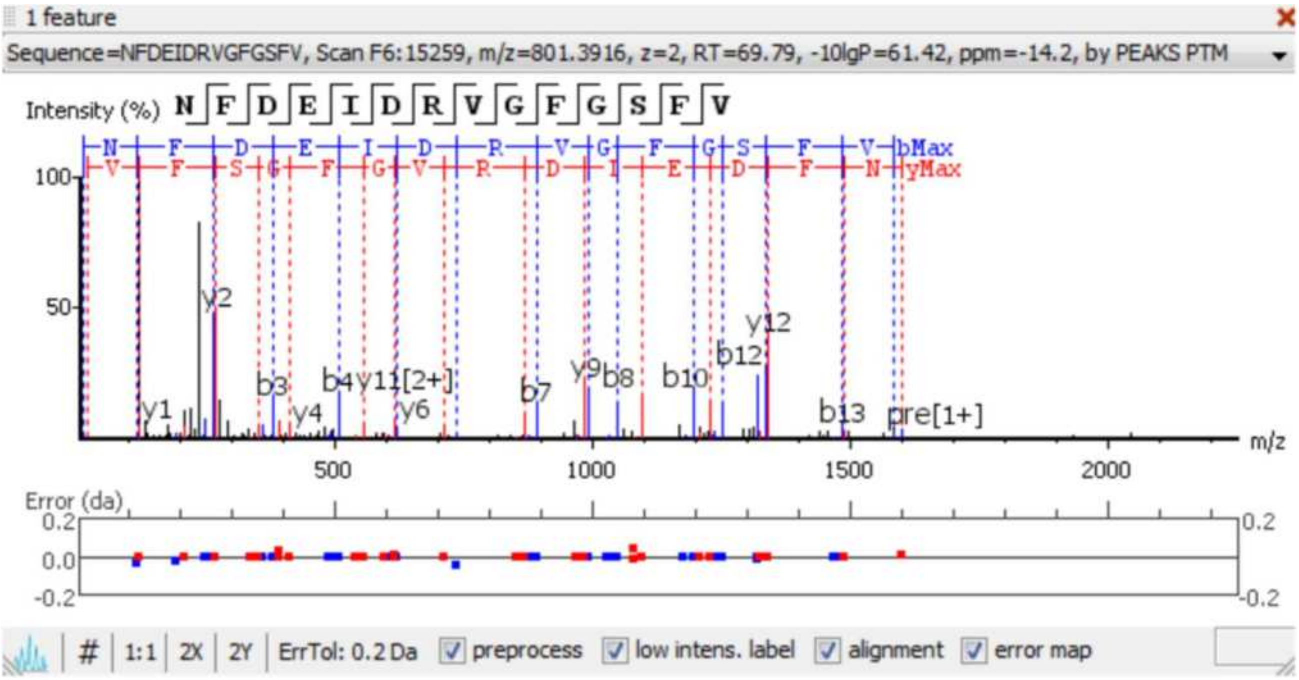

#	b	b-H2O	b-NH3	b (2+)	Seq	y	y-H2O	y-NH3	y (2+)	#
1	115.09	97.04	98.02	58.03	N					14
2	262.12	244.11	245.09	131.56	F	1487.71	1469.71	1470.69	744.36	13
3	377.15	359.14	360.12	189.10	D	1340.65	1322.64	1323.63	670.82	12
4	506.19	488.18	489.16	253.59	E	1225.62	1207.61	1208.59	613.30	11
5	619.27	601.26	602.25	310.14	I	1096.58	1078.58	1079.51	548.79	10
6	734.35	716.29	717.27	367.65	D	983.50	965.48	966.47	492.25	9
7	890.40	872.39	873.37	445.70	R	868.47	850.45	851.44	434.73	8
8	989.47	971.46	972.44	495.23	V	712.37	694.36	695.34	356.68	7
9	1046.49	1028.48	1029.46	523.75	G	613.30	595.29	596.27	307.15	6
10	1193.56	1175.55	1176.53	597.28	F	556.28	538.27	539.25	278.64	5
11	1250.58	1232.57	1233.55	625.79	G	409.21	391.20	392.15	205.10	4
12	1337.61	1319.60	1320.60	669.31	S	352.19	334.18	335.16	176.59	3
13	1484.68	1466.66	1467.66	742.84	F	265.15	247.14	248.13	133.08	2
14					V	118.09	100.08	101.06	59.54	1

Orcokinin-A-4 (Orc-A-4)

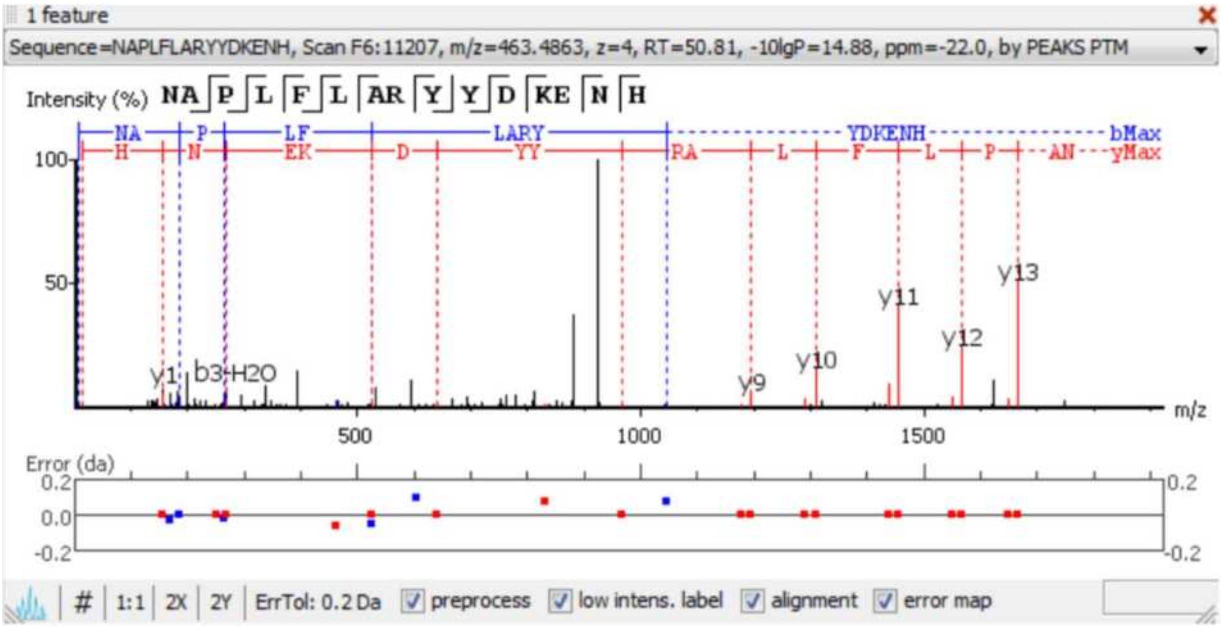

#	b	b-H2O	b-NH3	b (2+)	Seq	y	y-H2O	y-NH3	y (2+)	#
1	115.05	97.04	98.02	58.03	N					15
2	186.09	168.08	169.10	93.54	A	1736.88	1718.86	1719.85	868.94	14
3	283.14	265.16	266.11	142.07	P	1665.85	1647.83	1648.82	833.34	13
4	396.22	378.21	379.20	198.61	L	1568.79	1550.78	1551.77	784.89	12
5	543.29	525.34	526.27	272.15	F	1455.70	1437.69	1438.68	728.35	11
6	656.38	638.37	639.35	328.69	L	1308.63	1290.62	1291.61	654.82	10
7	727.41	709.40	710.39	364.21	A	1195.55	1177.54	1178.53	598.27	9
8	883.52	865.50	866.49	442.26	R	1124.51	1106.50	1107.48	562.76	8
9	1046.50	1028.57	1029.55	523.79	Y	968.41	950.40	951.38	484.71	7
10	1209.64	1191.63	1192.61	605.23	Y	805.35	787.34	788.32	403.17	6
11	1324.67	1306.66	1307.64	662.83	D	642.28	624.27	625.26	321.64	5
12	1452.76	1434.75	1435.74	726.88	K	527.26	509.25	510.23	264.13	4
13	1581.81	1563.80	1564.78	791.40	E	399.16	381.15	382.14	200.08	3
14	1695.85	1677.84	1678.82	848.42	N	270.12	252.11	253.09	135.56	2
15					H	156.08	138.07	139.05	78.54	1

Orcokinin-B-1 (Orc-B-1)

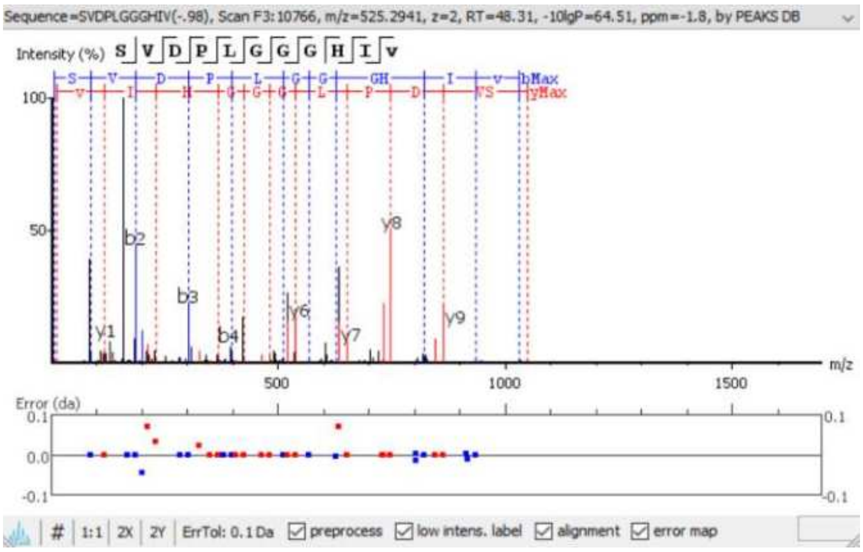

#	b	b-H2O	b-NH3	b (2+)	Seq	y	y-H2O	y-NH3	y (2+)	#
1	88.04	70.03	71.01	44.52	S					11
2	187.11	169.10	170.08	94.05	V	962.54	944.53	945.51	481.77	10
3	302.13	284.12	285.11	151.57	D	863.47	845.46	846.45	432.24	9
4	399.19	381.18	382.16	200.14	P	748.44	730.43	731.42	374.72	8
5	512.27	494.26	495.25	256.64	L	651.39	633.38	634.29	326.17	7
6	569.29	551.28	552.27	285.15	G	538.31	520.30	521.28	269.65	6
7	626.32	608.30	609.29	313.66	G	481.29	463.28	464.26	241.14	5
8	683.34	665.33	666.31	342.17	G	424.27	406.26	407.24	212.63	4
9	820.39	802.38	803.38	410.70	H	367.25	349.23	350.22	184.12	3
10	933.48	915.46	916.47	467.24	I	230.15	212.18	213.09	115.59	2
11					V(-.98)	117.10	99.09	100.08	59.05	1

Pyrokinin (PK)/FXPRLamides-PP-1 (PK-PP-1)_partial_part 1

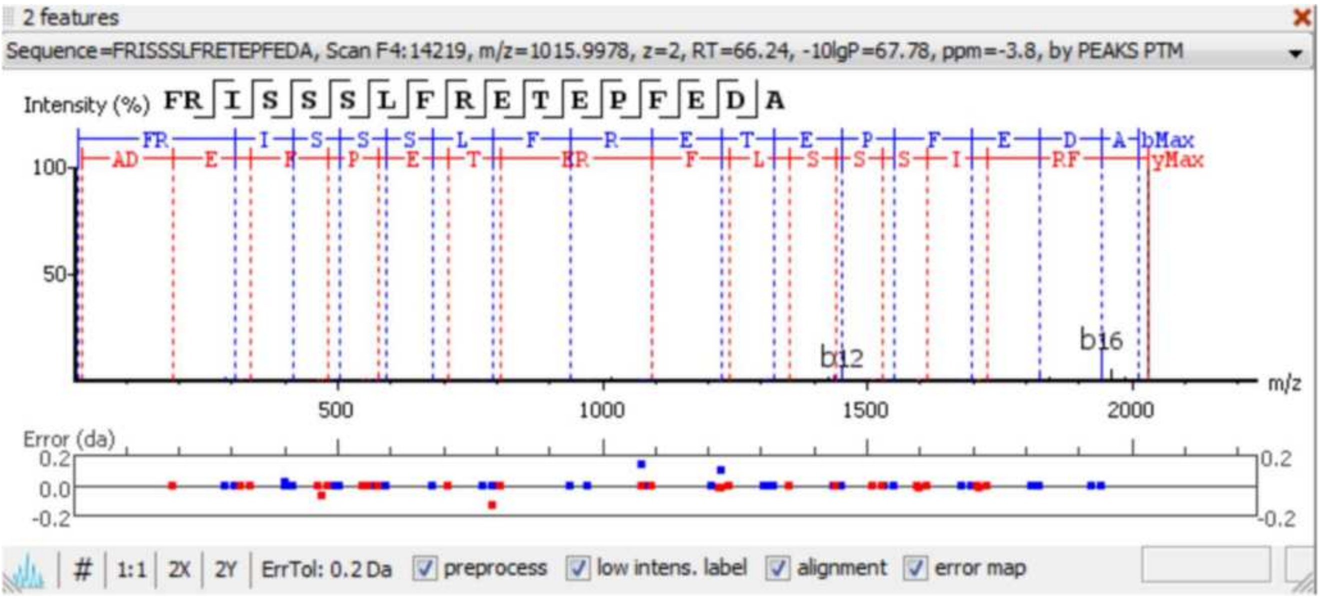

#	b	b-H2O	b-NH3	b (2+)	Seq	y	y-H2O	y-NH3	y (2+)	#
1	148.08	130.07	131.05	74.54	F					17
2	304.18	286.17	287.15	152.59	R	1883.91	1865.90	1866.89	942.46	16
3	417.26	399.22	400.23	209.13	I	1727.81	1709.80	1710.81	864.41	15
4	504.29	486.28	487.27	252.65	S	1614.73	1596.73	1597.72	807.86	14
5	591.32	573.31	574.30	296.16	S	1527.70	1509.68	1510.67	764.35	13
6	678.36	660.35	661.33	339.68	S	1440.67	1422.65	1423.64	720.83	12
7	791.44	773.43	774.42	396.22	L	1353.64	1335.62	1336.61	677.32	11
8	938.51	920.50	921.48	469.75	F	1240.55	1222.55	1223.54	620.77	10
9	1094.61	1076.45	1077.58	547.81	R	1093.48	1075.47	1076.45	547.23	9
10	1223.54	1205.64	1206.63	612.33	E	937.38	919.37	920.35	469.26	8
11	1324.70	1306.69	1307.68	662.85	T	808.34	790.33	791.44	404.67	7
12	1453.75	1435.73	1436.72	727.37	E	707.29	689.28	690.26	354.14	6
13	1550.80	1532.79	1533.77	775.90	P	578.25	560.24	561.22	289.62	5
14	1697.87	1679.85	1680.84	849.43	F	481.19	463.18	464.17	241.10	4
15	1826.91	1808.90	1809.89	913.95	E	334.12	316.11	317.10	167.56	3
16	1941.94	1923.92	1924.91	971.47	D	205.08	187.07	188.05	103.04	2
17					A	90.05	72.04	73.03	45.53	1

Pyrokinin (PK)/FXPRLamides-PP-1 (PK-PP-1)_partial_part 2

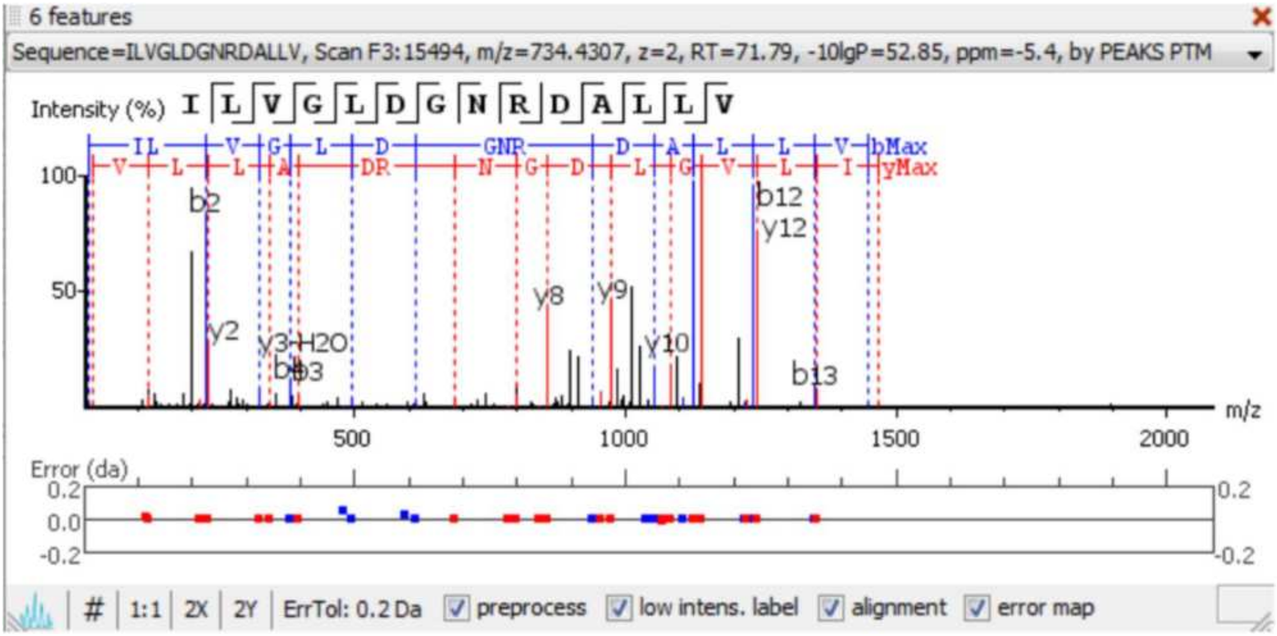

#	b	b-H2O	b-NH3	b (2+)	Seq	y	y-H2O	y-NH3	y (2+)	#
1	114.09	96.08	97.06	57.55	I					14
2	227.18	209.17	210.15	114.09	L	1354.77	1336.76	1337.74	677.88	13
3	326.24	308.23	309.22	163.62	V	1241.68	1223.67	1224.67	621.34	12
4	383.27	365.26	366.24	192.13	G	1142.62	1124.61	1125.59	571.81	11
5	496.35	478.34	479.26	248.67	L	1085.60	1067.59	1068.58	543.30	10
6	611.38	593.37	594.31	306.19	D	972.51	954.50	955.49	486.76	9
7	668.40	650.39	651.37	334.70	G	857.48	839.47	840.46	429.24	8
8	782.44	764.43	765.41	391.72	N	800.46	782.45	783.44	400.73	7
9	938.54	920.53	921.52	469.77	R	686.42	668.41	669.39	343.71	6
10	1053.57	1035.56	1036.55	527.28	D	530.32	512.31	513.29	265.66	5
11	1124.61	1106.59	1107.59	562.80	A	415.29	397.28	398.26	208.15	4
12	1237.69	1219.68	1220.67	619.35	L	344.25	326.24	327.23	172.63	3
13	1350.77	1332.76	1333.75	675.89	L	231.17	213.16	214.14	116.07	2
14					V	118.09	100.08	101.06	59.54	1

Pyrokinin (PK)/FXPRLamides-1 (PK-1)

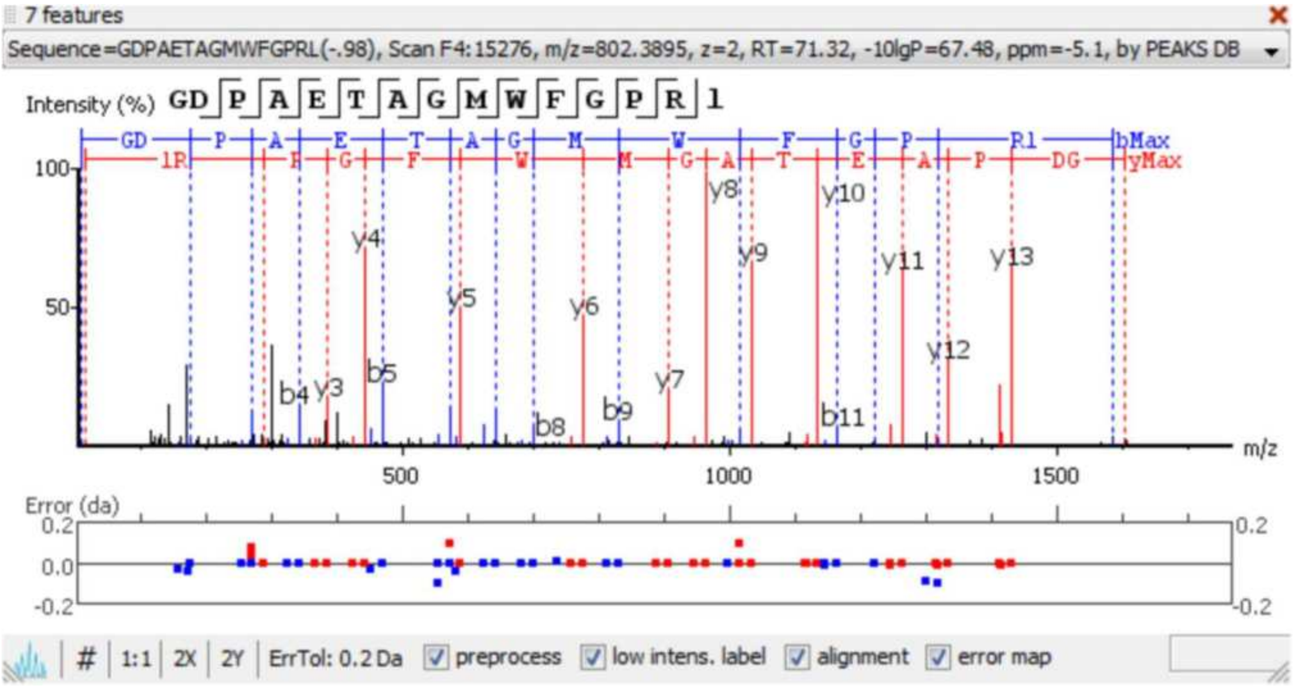

#	b	b-H2O	b-NH3	b (2+)	Seq	y	y-H2O	y-NH3	y (2+)	#
1	58.03	40.02	41.00	29.51	G					15
2	173.06	155.08	156.03	87.03	D	1546.75	1528.74	1529.72	773.87	14
3	270.11	252.10	253.08	135.55	P	1431.72	1413.72	1414.71	716.36	13
4	341.15	323.13	324.12	171.11	A	1334.67	1316.66	1317.66	667.83	12
5	470.19	452.21	453.16	235.59	E	1263.63	1245.62	1246.62	632.32	11
6	571.24	553.23	554.31	286.12	T	1134.59	1116.59	1117.57	567.79	10
7	642.27	624.26	625.25	321.64	A	1033.54	1015.53	1016.42	517.27	9
8	699.29	681.28	682.27	350.15	G	962.50	944.49	945.48	481.75	8
9	830.34	812.32	813.31	415.67	M	905.48	887.47	888.46	453.24	7
10	1016.42	998.40	999.39	508.71	W	774.44	756.43	757.41	387.72	6
11	1163.49	1145.47	1146.47	582.29	F	588.36	570.35	571.24	294.68	5
12	1220.50	1202.49	1203.48	610.75	G	441.29	423.28	424.27	221.15	4
13	1317.66	1299.64	1300.53	659.28	P	384.27	366.26	367.25	192.64	3
14	1473.66	1455.65	1456.63	737.32	R	287.22	269.16	270.11	144.11	2
15					L(-.98)	131.12	113.11	114.09	66.06	1

Pyrokinin (PK)/FXPRLamides-PP-2 (PK-PP-2)_partial_part 1

Pyrokinin (PK)/FXPRLamides-PP-2$^{1-33}$ (PK-PP-2$^{1-33}$)

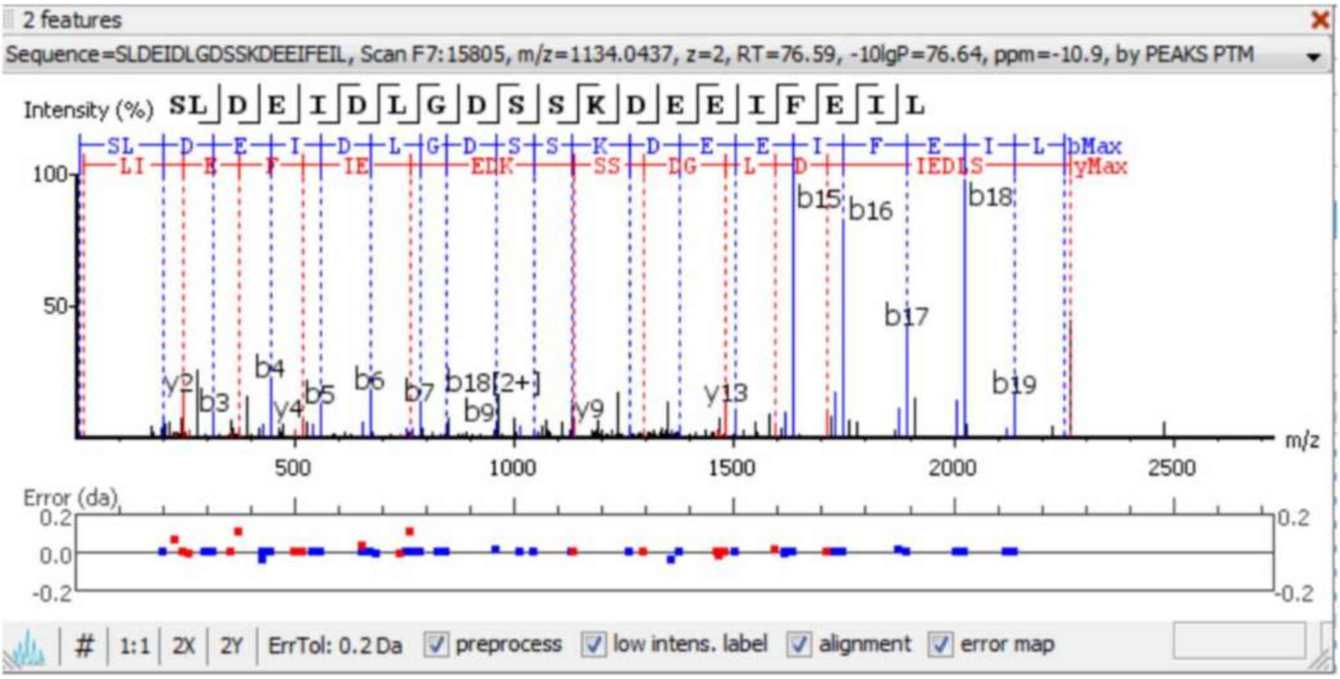

#	b	b-H2O	b-NH3	b (2+)	Seq	y	y-H2O	y-NH3	y (2+)	#
1	88.04	70.03	71.01	44.52	S					20
2	201.12	183.11	184.10	101.06	L	2180.05	2162.04	2163.02	1090.52	19
3	316.15	298.14	299.12	158.58	D	2066.97	2048.95	2049.94	1033.98	18
4	445.19	427.18	428.21	223.10	E	1951.94	1933.93	1934.91	976.47	17
5	558.28	540.26	541.25	279.64	I	1822.90	1804.89	1805.87	911.95	16
6	673.30	655.29	656.28	337.15	D	1709.81	1691.80	1692.78	855.41	15
7	786.39	768.38	769.36	393.69	L	1594.77	1576.77	1577.76	797.89	14
8	843.41	825.41	826.38	422.20	G	1481.71	1463.69	1464.70	741.37	13
9	958.43	940.43	941.41	479.72	D	1424.68	1406.67	1407.65	712.84	12
10	1045.46	1027.46	1028.44	523.23	S	1309.65	1291.64	1292.63	655.29	11
11	1132.50	1114.49	1115.47	566.75	S	1222.62	1204.61	1205.59	611.81	10
12	1260.60	1242.59	1243.57	630.80	K	1135.59	1117.58	1118.56	568.29	9
13	1375.63	1357.61	1358.65	688.33	D	1007.49	989.48	990.47	504.25	8
14	1504.66	1486.65	1487.64	752.83	E	892.47	874.46	875.44	446.73	7
15	1633.71	1615.69	1616.69	817.35	E	763.32	745.41	746.40	382.21	6
16	1746.79	1728.79	1729.77	873.90	I	634.38	616.37	617.35	317.69	5
17	1893.85	1875.83	1876.83	947.43	F	521.30	503.29	504.27	261.16	4
18	2022.90	2004.89	2005.88	1011.95	E	374.12	356.22	357.20	187.61	3
19	2136.00	2117.98	2118.96	1068.49	I	245.19	227.10	228.16	123.09	2
20					L	132.10	114.09	115.07	66.55	1

Pyrokinin (PK)/FXPRLamides-PP-2$^{22-33}$

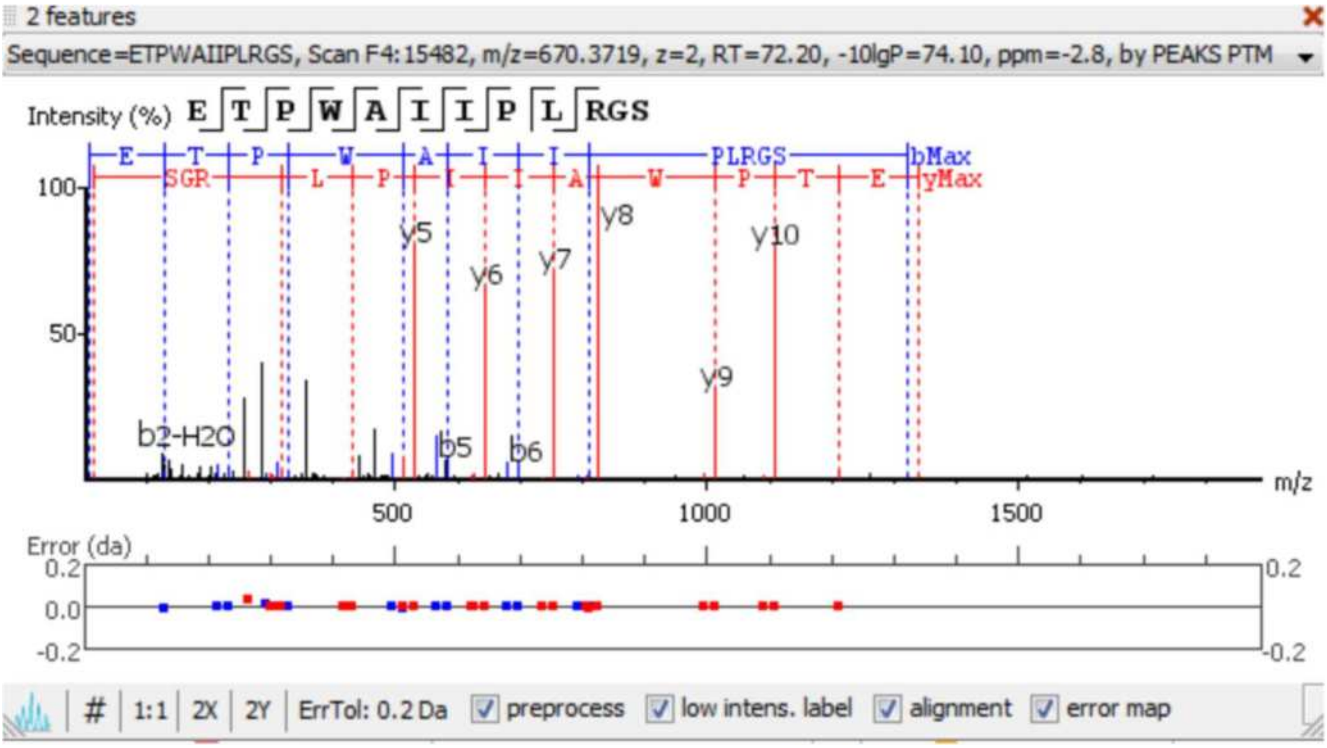

#	b	b-H2O	b-NH3	b (2+)	Seq	y	y-H2O	y-NH3	y (2+)	#
1	130.07	112.04	113.02	65.53	E					12
2	231.10	213.09	214.07	116.05	T	1210.69	1192.68	1193.67	605.85	11
3	328.15	310.14	311.12	164.58	P	1109.65	1091.63	1092.61	555.32	10
4	514.23	496.22	497.20	257.62	W	1012.59	994.59	995.57	506.80	9
5	585.27	567.26	568.24	293.11	A	826.51	808.51	809.50	413.76	8
6	698.35	680.34	681.32	349.68	I	755.48	737.47	738.45	378.24	7
7	811.43	793.43	794.41	406.22	I	642.39	624.38	625.37	321.70	6
8	908.49	890.48	891.46	454.74	P	529.31	511.30	512.28	265.12	5
9	1021.57	1003.56	1004.55	511.30	L	432.26	414.25	415.23	216.63	4
10	1177.67	1159.66	1160.65	589.34	R	319.17	301.17	302.15	160.09	3
11	1234.69	1216.68	1217.67	617.85	G	163.07	145.06	146.04	82.04	2
12					S	106.05	88.04	89.02	53.52	1

Pyrokinin (PK)/FXPRLamides-PP-2 (PK-PP-2)_partial_part 2

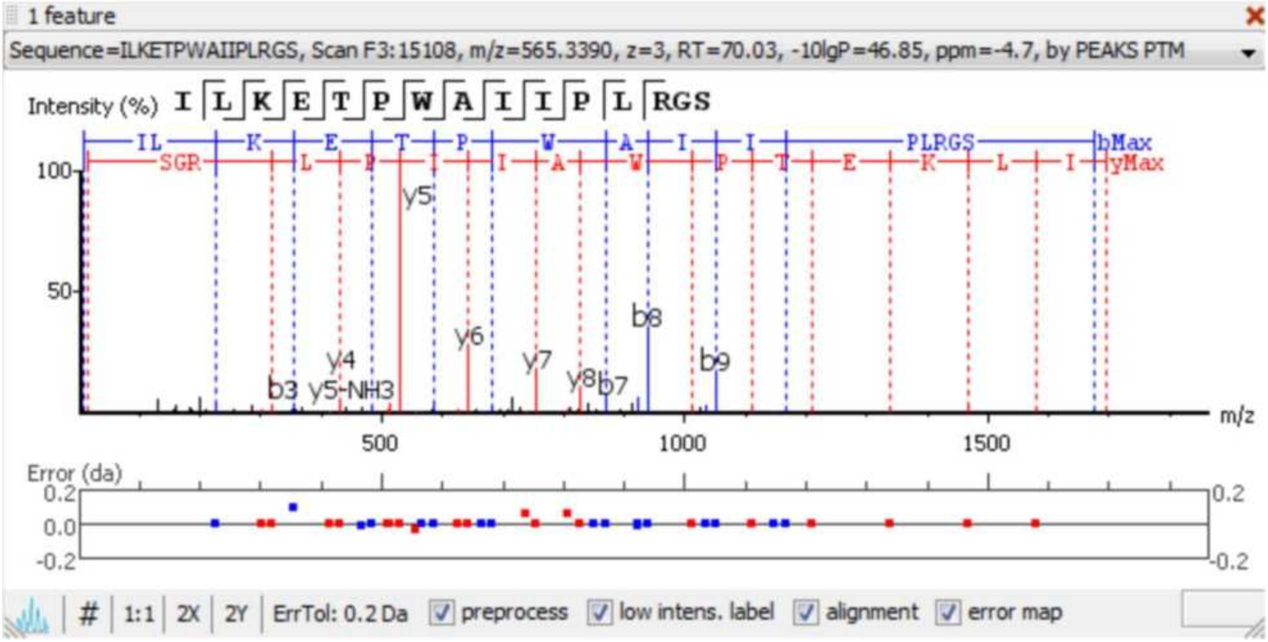

#	b	b-H2O	b-NH3	b (2+)	Seq	y	y-H2O	y-NH3	y (2+)	#
1	114.09	96.08	97.06	57.55	I					15
2	227.18	209.17	210.15	114.09	L	1580.91	1562.91	1563.89	790.96	14
3	355.18	337.26	338.24	178.14	K	1467.83	1449.82	1450.80	734.42	13
4	484.31	466.30	467.31	242.66	E	1339.73	1321.73	1322.71	670.37	12
5	585.36	567.35	568.33	293.18	T	1210.69	1192.68	1193.67	605.85	11
6	682.41	664.41	665.39	341.71	P	1109.65	1091.64	1092.62	555.36	10
7	868.49	850.48	851.47	434.75	W	1012.59	994.58	995.57	506.80	9
8	939.53	921.52	922.52	470.27	A	826.52	808.44	809.49	413.76	8
9	1052.61	1034.60	1035.59	526.81	I	755.48	737.40	738.45	378.24	7
10	1165.70	1147.68	1148.67	583.35	I	642.39	624.39	625.37	321.70	6
11	1262.75	1244.74	1245.72	631.88	P	529.31	511.30	512.28	265.15	5
12	1375.84	1357.82	1358.81	688.42	L	432.26	414.25	415.23	216.63	4
13	1531.94	1513.93	1514.91	766.47	R	319.17	301.17	302.15	160.09	3
14	1588.96	1570.95	1571.93	794.98	G	163.07	145.06	146.04	82.04	2
15					S	106.05	88.04	89.02	53.52	1

Pyrokinin (PK)/FXPRLamides-2_[pQ] (PK-2)

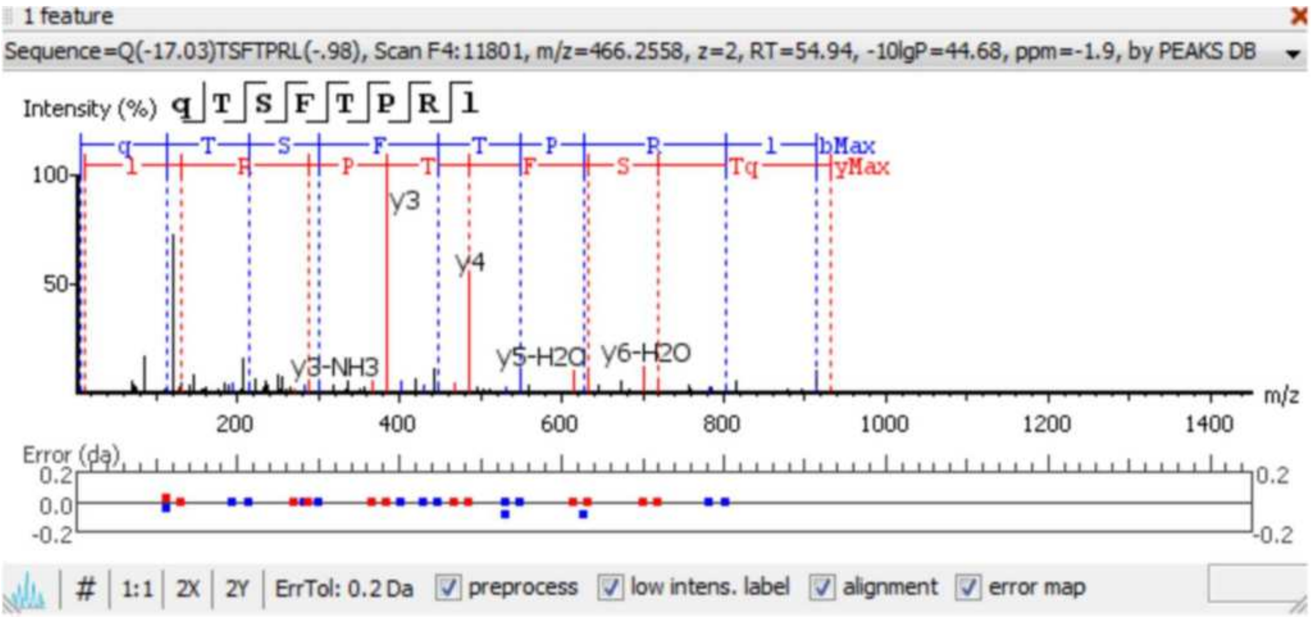

#	b	b-H2O	b-NH3	b (2+)	Seq	y	y-H2O	y-NH3	y (2+)	#
1	112.09	94.03	95.01	56.52	Q(-17.03)					8
2	213.09	195.08	196.06	107.04	T	820.47	802.46	803.44	410.73	7
3	300.12	282.11	283.09	150.56	S	719.42	701.41	702.39	360.21	6
4	447.19	429.18	430.16	224.09	F	632.39	614.38	615.36	316.69	5
5	548.24	530.22	531.30	274.62	T	485.32	467.31	468.29	243.16	4
6	645.29	627.36	628.26	323.14	P	384.27	366.26	367.25	192.64	3
7	801.39	783.38	784.36	401.18	R	287.22	269.21	270.19	144.11	2
8					L(-.98)	131.12	113.07	114.09	66.06	1

Pyrokinin (PK)/FXPRLamides-3$_{long}$ (PK-3$_{long}$)

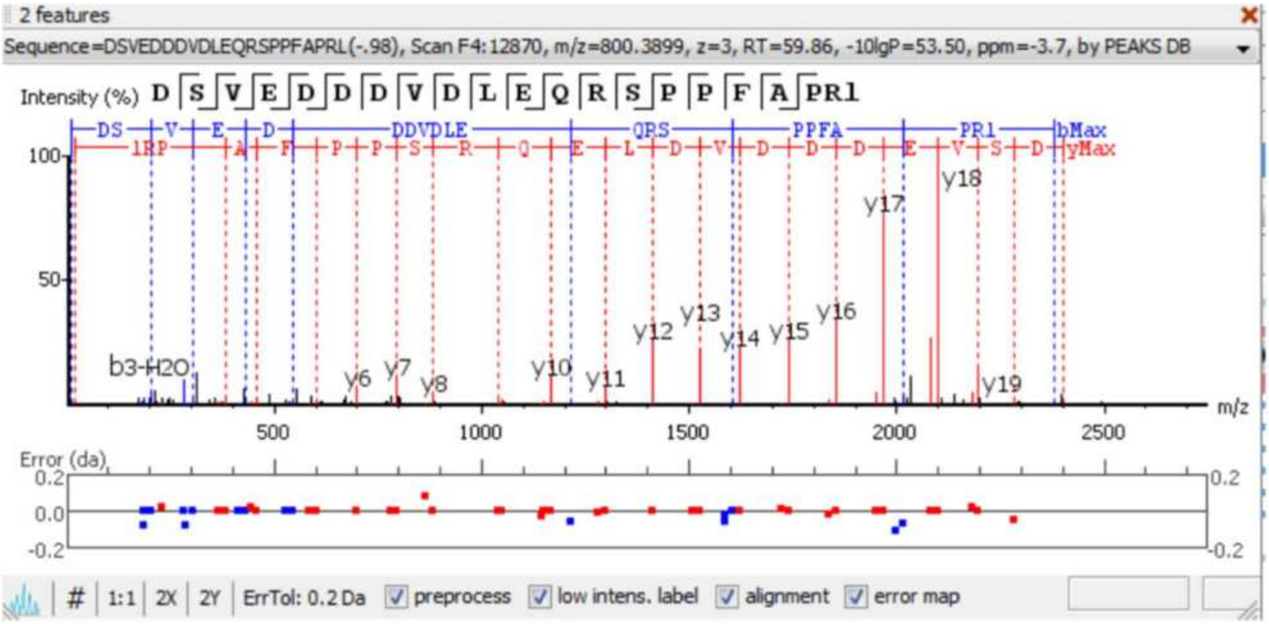

#	b	b-H2O	b-NH3	b (2+)	Seq	y	y-H2O	y-NH3	y (2+)	#
1	116.03	98.02	99.01	58.52	D					21
2	203.07	185.06	186.12	102.03	S	2284.18	2266.11	2267.09	1142.59	20
3	302.13	284.12	285.19	151.57	V	2197.09	2179.08	2180.04	1099.04	19
4	431.18	413.17	414.15	216.09	E	2098.02	2080.01	2080.99	1049.52	18
5	546.21	528.19	529.18	273.60	D	1968.98	1950.96	1951.95	984.99	17
6	661.23	643.22	644.20	331.12	D	1853.95	1835.94	1836.95	927.48	16
7	776.26	758.25	759.23	388.63	D	1738.93	1720.90	1721.90	869.96	15
8	875.33	857.32	858.30	438.16	V	1623.90	1605.89	1606.87	812.45	14
9	990.35	972.34	973.33	495.68	D	1524.83	1506.82	1507.80	762.91	13
10	1103.44	1085.43	1086.41	552.22	L	1409.80	1391.79	1392.77	705.40	12
11	1232.48	1214.47	1215.52	616.74	E	1296.72	1278.71	1279.70	648.86	11
12	1360.54	1342.53	1343.51	680.77	Q	1167.67	1149.66	1150.65	584.34	10
13	1516.64	1498.63	1499.61	758.82	R	1039.62	1021.61	1022.59	520.31	9
14	1603.67	1585.69	1586.71	802.34	S	883.52	865.42	866.49	442.23	8
15	1700.73	1682.71	1683.70	850.86	P	796.48	778.47	779.46	398.74	7
16	1797.78	1779.77	1780.75	899.39	P	699.43	681.42	682.40	350.21	6
17	1944.85	1926.84	1927.82	972.92	F	602.38	584.37	585.34	301.69	5
18	2015.96	1997.87	1998.97	1008.44	A	455.31	437.30	438.28	228.13	4
19	2112.94	2094.93	2095.91	1056.97	P	384.27	366.26	367.25	192.64	3
20	2269.04	2251.03	2252.01	1135.02	R	287.22	269.21	270.19	144.11	2
21					L(-.98)	131.12	113.11	114.09	66.06	1

Pyrokinin (PK)/FXPRLamides-3 (PK-3)

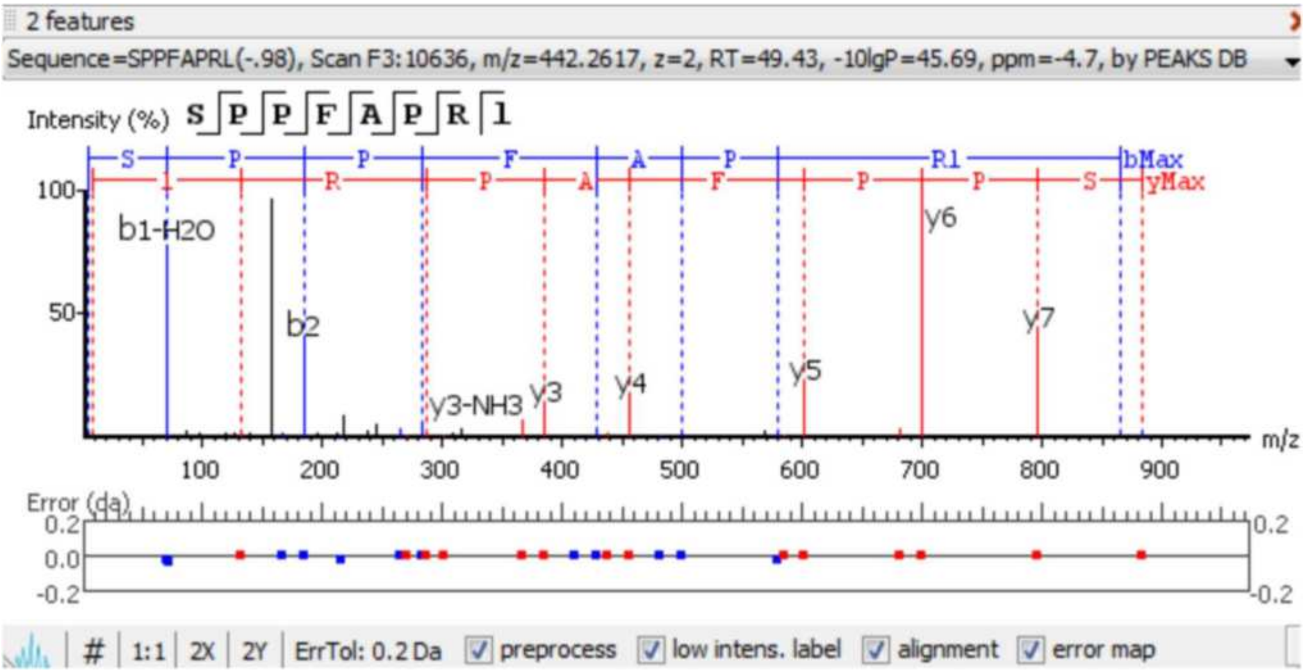

#	b	b-H2O	b-NH3	b (2+)	Seq	y	y-H2O	y-NH3	y (2+)	#
1	88.04	70.07	71.06	44.52	S					8
2	185.09	167.08	168.07	93.05	P	796.48	778.47	779.46	398.74	7
3	282.14	264.13	265.12	141.57	P	699.43	681.42	682.40	350.21	6
4	429.21	411.20	412.19	215.14	F	602.38	584.37	585.35	301.69	5
5	500.25	482.24	483.22	250.63	A	455.31	437.30	438.28	228.15	4
6	597.30	579.33	580.28	299.15	P	384.27	366.26	367.25	192.64	3
7	753.40	735.39	736.38	377.20	R	287.22	269.21	270.19	144.11	2
8					L(-.98)	131.12	113.11	114.09	66.06	1

Pyrokinin (PK)/FXPRLamides-5 (PK-5)

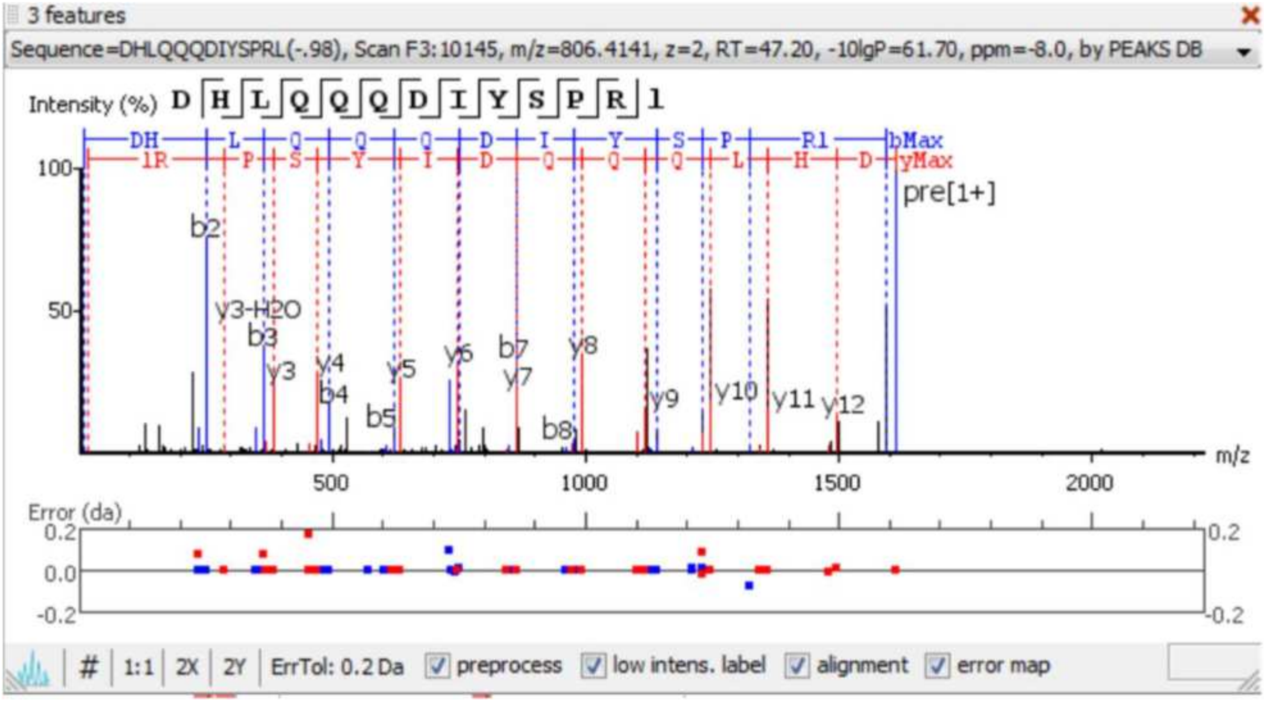

#	b	b-H2O	b-NH3	b (2+)	Seq	y	y-H2O	y-NH3	y (2+)	#
1	116.03	98.02	99.01	58.52	D					13
2	253.09	235.08	236.07	127.05	H	1496.79	1478.79	1479.78	748.90	12
3	366.18	348.17	349.15	183.59	L	1359.74	1341.72	1342.71	680.37	11
4	494.24	476.23	477.21	247.62	Q	1246.65	1228.55	1229.65	623.83	10
5	622.30	604.29	605.27	311.65	Q	1118.60	1100.59	1101.57	559.80	9
6	750.34	732.24	733.33	375.68	Q	990.54	972.53	973.51	495.77	8
7	865.38	847.37	848.36	433.19	D	862.48	844.47	845.45	431.74	7
8	978.47	960.45	961.44	489.73	I	747.45	729.44	730.42	374.23	6
9	1141.53	1123.52	1124.50	571.27	Y	634.37	616.36	617.34	317.68	5
10	1228.55	1210.55	1211.52	614.78	S	471.30	453.29	454.10	236.07	4
11	1325.69	1307.60	1308.59	663.31	P	384.27	366.18	367.25	192.64	3
12	1481.71	1463.70	1464.69	741.38	R	287.22	269.21	270.19	144.11	2
13					L(-.98)	131.12	113.11	114.09	66.06	1

Pyrokinin (PK)/FXPRLamides-PP-3 (PK-PP-3)

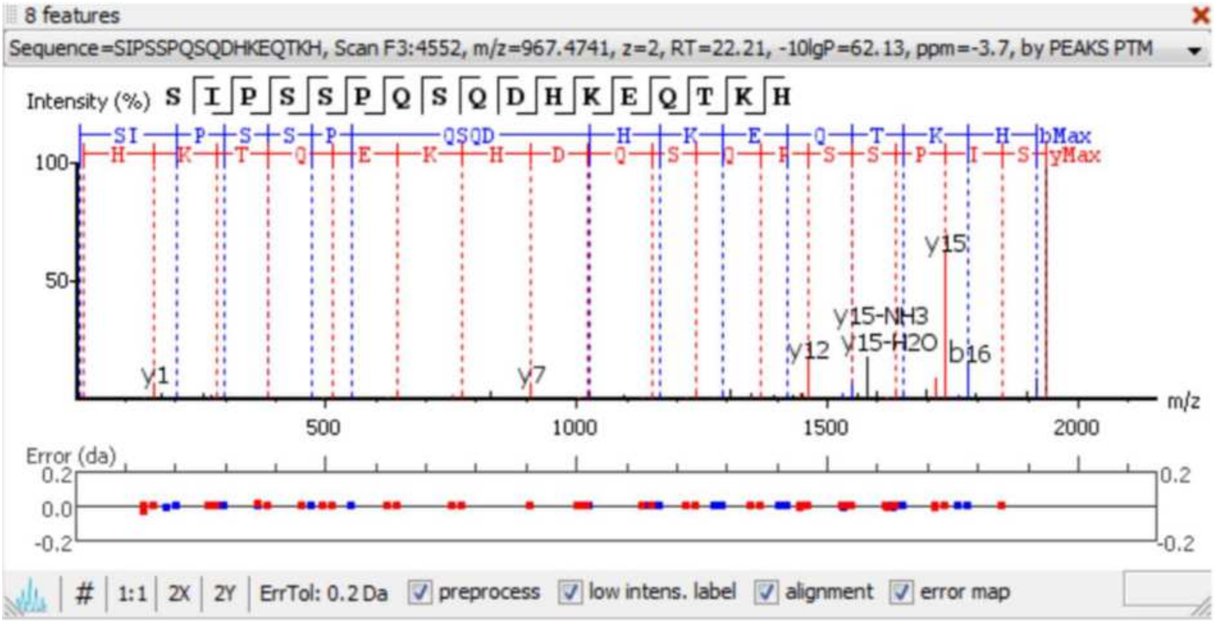

#	b	b-H2O	b-NH3	b (2+)	Seq	y	y-H2O	y-NH3	y (2+)	#
1	88.04	70.03	71.01	44.52	S					17
2	201.12	183.11	184.11	101.06	I	1846.90	1828.89	1829.88	923.95	16
3	298.18	280.17	281.15	149.59	P	1733.82	1715.80	1716.81	867.41	15
4	385.22	367.20	368.18	193.10	S	1636.77	1618.75	1619.76	818.88	14
5	472.24	454.23	455.21	236.62	S	1549.73	1531.72	1532.72	775.37	13
6	569.29	551.28	552.27	285.15	P	1462.70	1444.69	1445.69	731.85	12
7	697.35	679.34	680.33	349.18	Q	1365.65	1347.63	1348.62	683.33	11
8	784.38	766.37	767.36	392.69	S	1237.59	1219.57	1220.57	619.30	10
9	912.44	894.43	895.42	456.72	Q	1150.56	1132.55	1133.54	575.78	9
10	1027.47	1009.46	1010.45	514.23	D	1022.50	1004.49	1005.48	511.75	8
11	1164.53	1146.52	1147.51	582.76	H	907.47	889.46	890.45	454.23	7
12	1292.62	1274.60	1275.60	646.81	K	770.41	752.40	753.39	385.71	6
13	1421.66	1403.65	1404.64	711.33	E	642.32	624.31	625.29	321.66	5
14	1549.73	1531.72	1532.72	775.36	Q	513.28	495.27	496.25	257.14	4
15	1650.78	1632.76	1633.76	825.89	T	385.22	367.20	368.19	193.11	3
16	1778.86	1760.85	1761.85	889.93	K	284.17	266.16	267.14	142.59	2
17					H	156.08	138.07	139.09	78.54	1

Pigment dispersing factor (PDF)

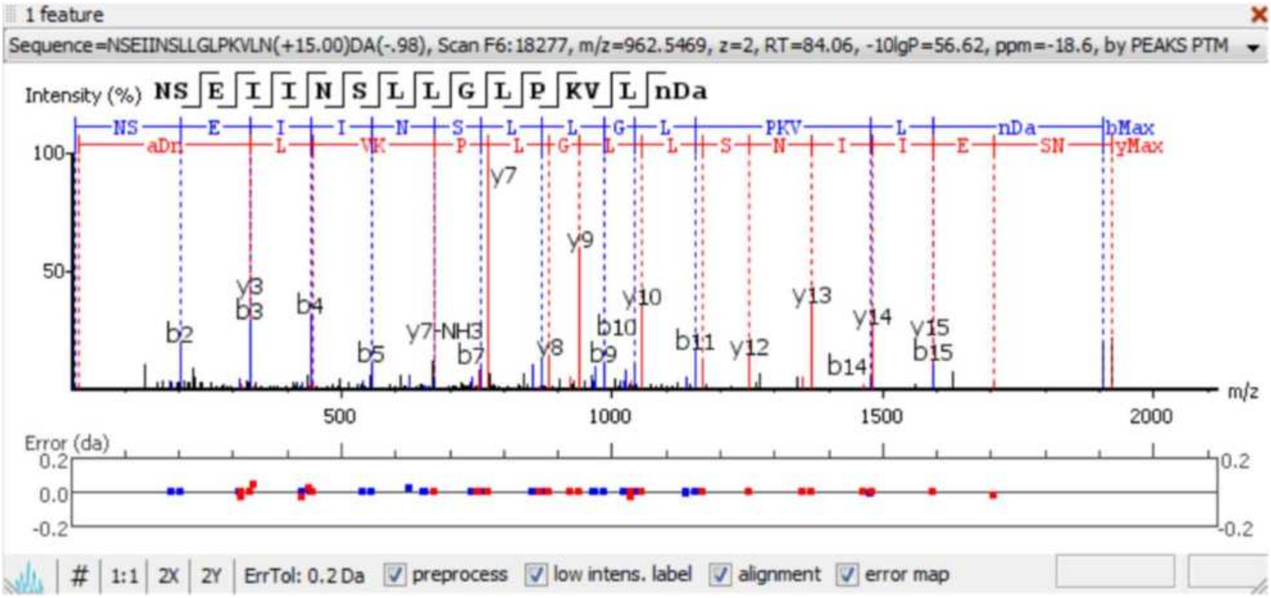

#	b	b-H2O	b-NH3	b (2+)	Seq	y	y-H2O	y-NH3	y (2+)	#
1	115.05	97.04	98.02	58.03	N					18
2	202.08	184.07	185.06	101.54	S	1810.03	1792.02	1793.01	905.52	17
3	331.13	313.11	314.10	166.06	E	1723.00	1704.99	1706.00	862.00	16
4	444.21	426.20	427.18	222.60	I	1593.96	1575.95	1576.93	797.48	15
5	557.29	539.28	540.27	279.15	I	1480.88	1462.86	1463.86	740.94	14
6	671.34	653.32	654.31	336.17	N	1367.79	1349.78	1350.77	684.39	13
7	758.37	740.36	741.34	379.68	S	1253.75	1235.74	1236.72	627.37	12
8	871.45	853.44	854.43	436.23	L	1166.72	1148.70	1149.69	583.86	11
9	984.54	966.53	967.52	492.77	L	1053.63	1035.65	1036.59	527.32	10
10	1041.56	1023.54	1024.54	521.28	G	940.55	922.54	923.53	470.77	9
11	1154.65	1136.63	1137.63	577.82	L	883.53	865.51	866.50	442.23	8
12	1251.69	1233.68	1234.67	626.32	P	770.44	752.43	753.42	385.72	7
13	1379.79	1361.78	1362.76	690.39	K	673.39	655.38	656.36	337.15	6
14	1478.88	1460.85	1461.83	739.93	V	545.29	527.28	528.27	273.15	5
15	1591.95	1573.93	1574.92	796.47	L	446.22	428.25	429.20	223.61	4
16	1720.98	1702.97	1703.96	860.99	N(+15.00)	333.14	315.17	316.11	167.07	3
17	1836.01	1818.00	1818.98	918.51	D	204.10	186.09	187.07	102.55	2
18					A(-.98)	89.07	71.06	72.04	45.04	1

Pigment dispersing factor (PDF)

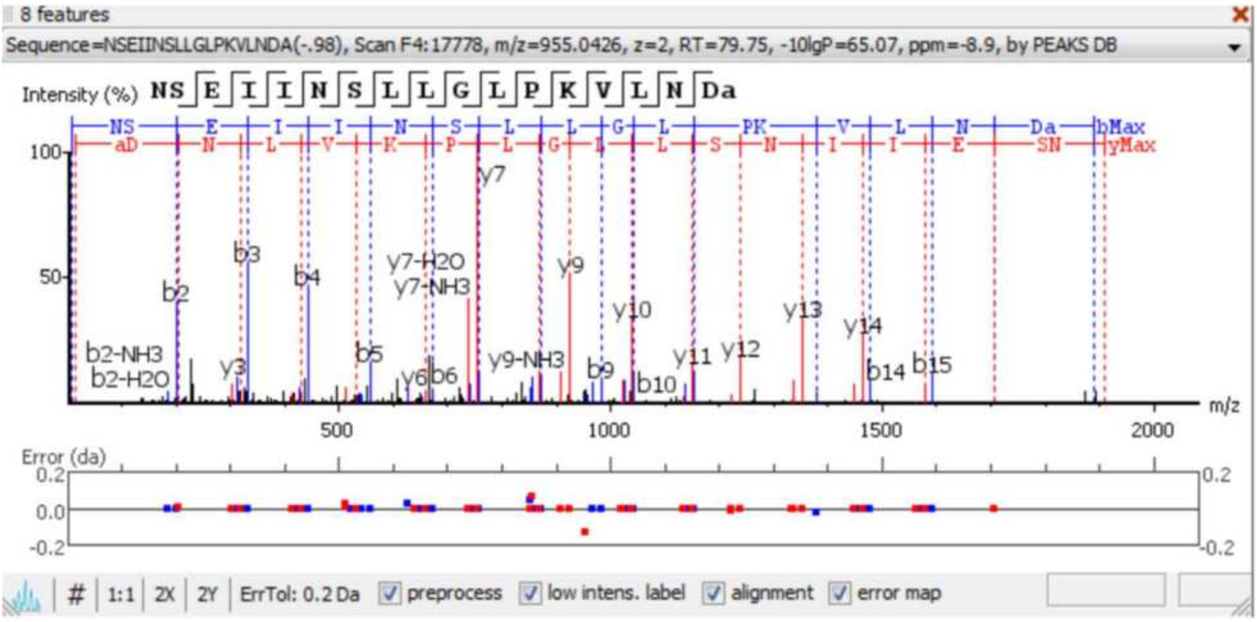

#	b	b-H2O	b-NH3	b (2+)	Seq	y	y-H2O	y-NH3	y (2+)	#
1	115.05	97.04	98.02	58.03	N					18
2	202.08	184.07	185.06	101.54	S	1795.03	1777.02	1778.01	898.02	17
3	331.12	313.11	314.10	166.06	E	1707.99	1689.99	1690.97	854.43	16
4	444.21	426.20	427.18	222.60	I	1578.96	1560.95	1561.94	789.98	15
5	557.29	539.28	540.27	279.15	I	1465.88	1447.87	1448.85	733.44	14
6	671.33	653.32	654.31	336.17	N	1352.79	1334.78	1335.77	676.89	13
7	758.37	740.35	741.34	379.68	S	1238.75	1220.74	1221.73	619.87	12
8	871.45	853.44	854.43	436.23	L	1151.72	1133.70	1134.69	576.36	11
9	984.54	966.53	967.51	492.77	L	1038.63	1020.62	1021.60	519.82	10
10	1041.56	1023.54	1024.54	521.27	G	925.55	907.54	908.52	463.27	9
11	1154.64	1136.63	1137.62	577.82	L	868.53	850.51	851.50	434.76	8
12	1251.69	1233.68	1234.67	626.31	P	755.44	737.43	738.41	378.22	7
13	1379.81	1361.78	1362.76	690.39	K	658.39	640.38	641.36	329.69	6
14	1478.86	1460.85	1461.84	739.93	V	530.29	512.27	513.23	265.65	5
15	1591.94	1573.93	1574.92	796.47	L	431.22	413.21	414.20	216.11	4
16	1705.99	1687.97	1688.96	853.44	N	318.14	300.13	301.11	159.57	3
17	1821.01	1803.00	1803.99	911.01	D	204.09	186.09	187.07	102.55	2
18					A(-.98)	89.07	71.06	72.04	45.04	1

Proctolin-PP_partial

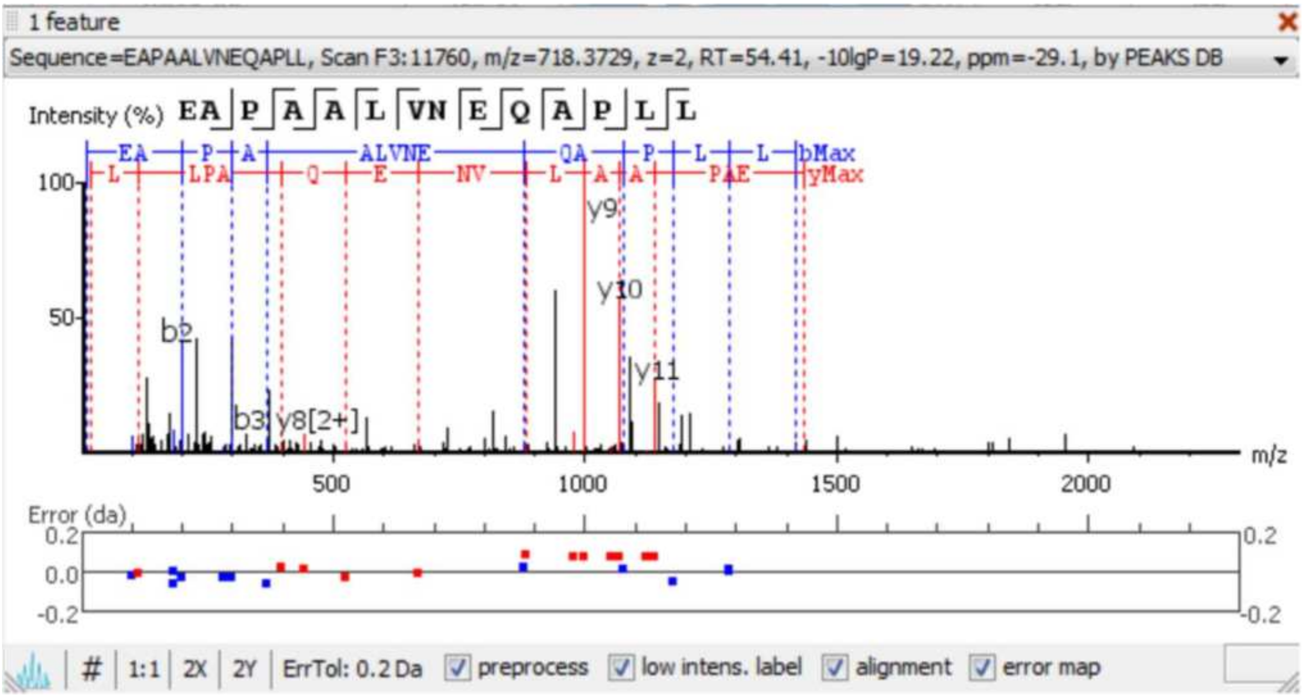

#	b	b-H2O	b-NH3	b (2+)	Seq	y	y-H2O	y-NH3	y (2+)	#
1	130.05	112.04	113.02	65.53	E					14
2	201.12	183.15	184.06	101.07	A	1306.74	1288.73	1289.71	653.87	13
3	298.18	280.17	281.11	149.57	P	1235.70	1217.69	1218.67	618.35	12
4	369.25	351.17	352.15	185.09	A	1138.56	1120.64	1121.54	569.82	11
5	440.21	422.20	423.19	220.61	A	1067.53	1049.60	1050.50	534.30	10
6	553.30	535.29	536.27	277.15	L	996.49	978.56	979.46	498.79	9
7	652.37	634.36	635.34	326.68	V	883.40	865.48	866.46	442.23	8
8	766.41	748.40	749.38	383.70	N	784.42	766.41	767.39	392.71	7
9	895.45	877.42	878.43	448.23	E	670.39	652.37	653.35	335.69	6
10	1023.51	1005.50	1006.48	512.26	Q	541.33	523.36	524.31	271.17	5
11	1094.55	1076.54	1077.50	547.77	A	413.28	395.27	396.22	207.14	4
12	1191.60	1173.59	1174.63	596.30	P	342.24	324.23	325.21	171.62	3
13	1304.68	1286.66	1287.65	652.84	L	245.19	227.18	228.16	123.09	2
14					L	132.10	114.09	115.09	66.55	1

RFLamide

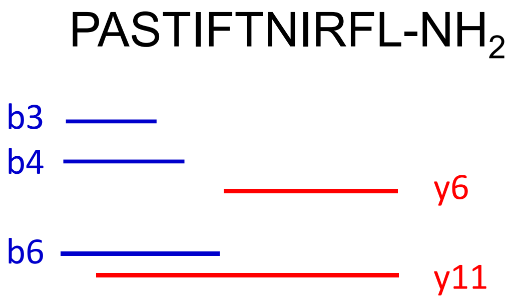

PASTIFTNIRFL-NH2
b3
b4
y6
b6
y11

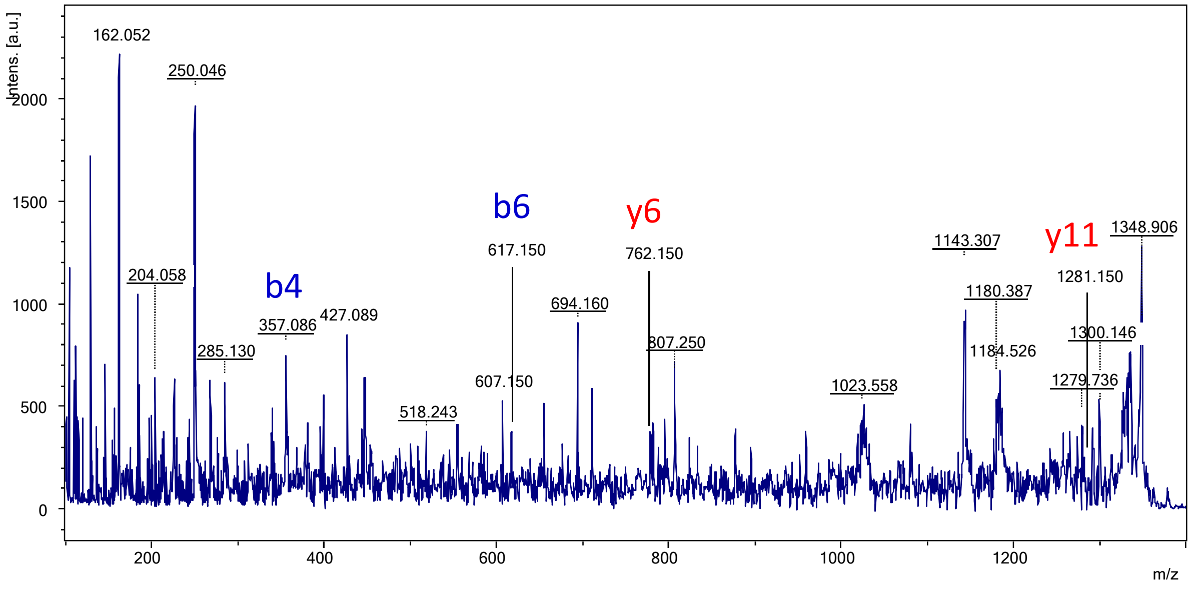

Intens. [a.u.]
2000
1500
1000
500
0
162.052
250.046
204.058
285.130
b4
357.086
427.089
518.243
b6
617.150
607.150
694.160
y6
762.150
807.250
1023.558
1143.307
1180.387
1184.526
y11
1281.150
1279.736
1300.146
1348.906
200
400
600
800
1000
1200
m/z

RYamide (RYa-1)_[pQ]

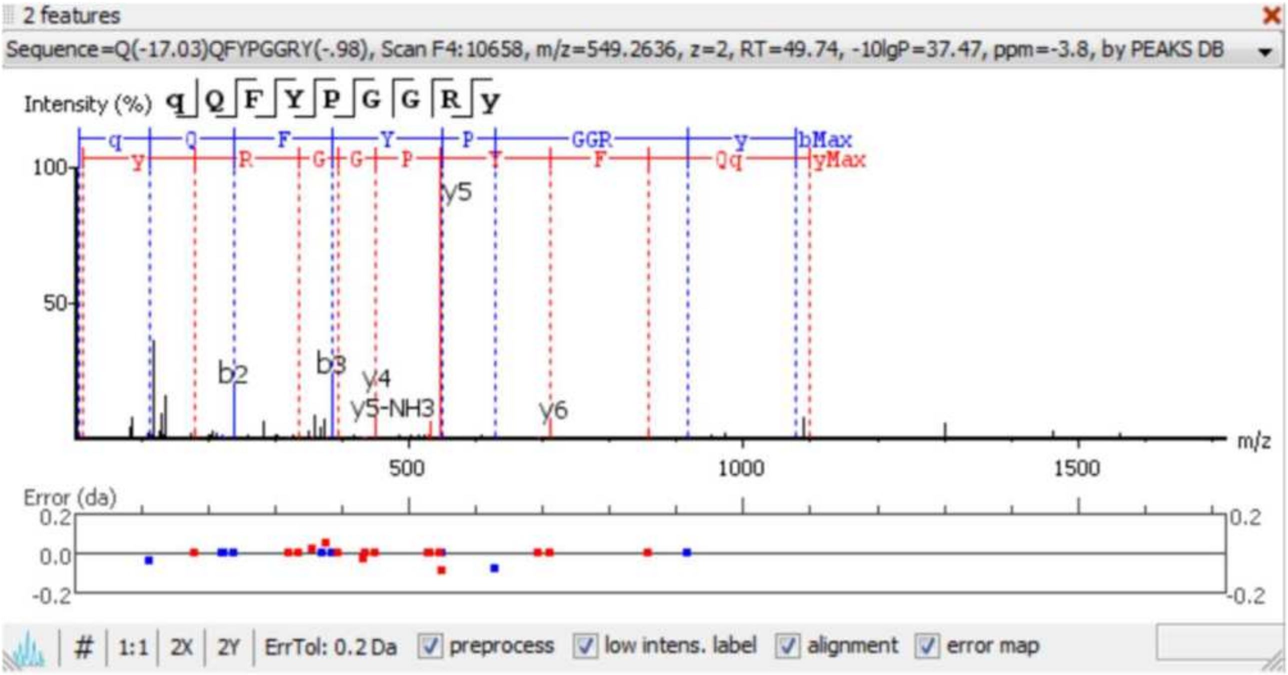

#	b	b-H2O	b-NH3	b (2+)	Seq	y	y-H2O	y-NH3	y (2+)	#
1	112.09	94.03	95.01	56.52	Q(-17.03)					9
2	240.10	222.09	223.07	120.55	Q	986.48	968.47	969.46	493.74	8
3	387.17	369.16	370.14	194.08	F	858.43	840.42	841.40	429.71	7
4	550.23	532.22	533.20	275.62	Y	711.36	693.35	694.33	356.16	6
5	647.28	629.35	630.26	324.14	P	548.29	530.28	531.27	274.65	5
6	704.30	686.29	687.28	352.65	G	451.24	433.26	434.22	226.12	4
7	761.33	743.32	744.30	381.16	G	394.22	376.15	377.19	197.61	3
8	917.43	899.42	900.40	459.21	R	337.20	319.19	320.17	169.10	2
9					Y(-.98)	181.10	163.09	164.07	91.05	1

short neuropeptide F-PP-1 (sNPF-PP-1)_part 1

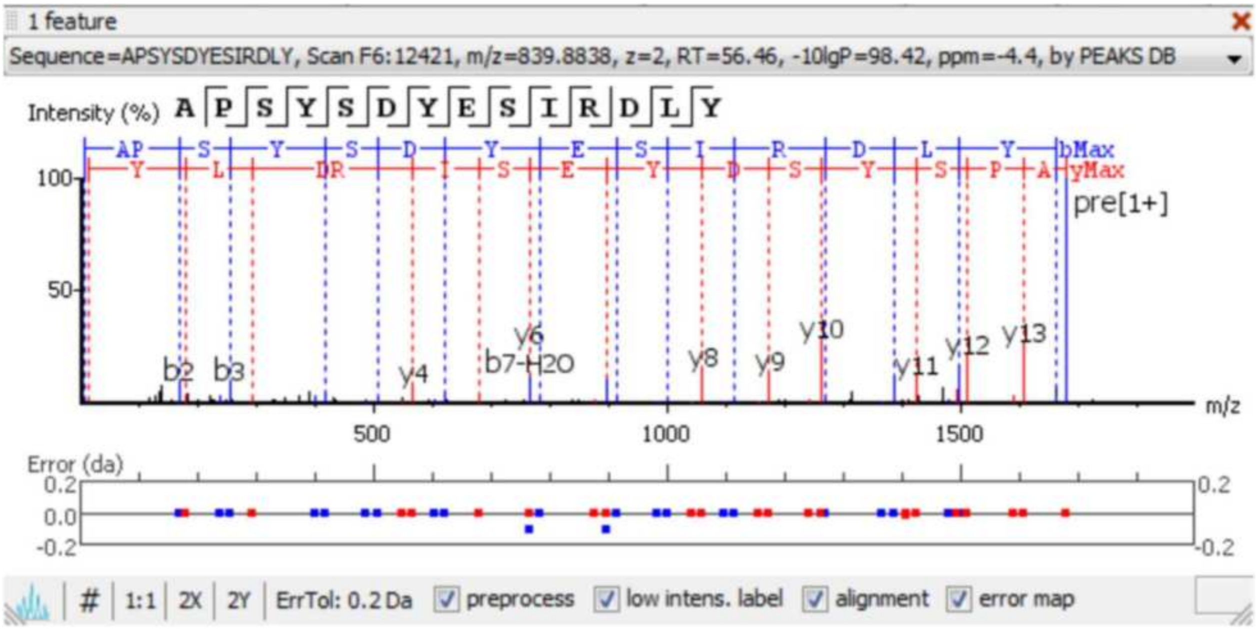

#	b	b-H2O	b-NH3	b (2+)	Seq	y	y-H2O	y-NH3	y (2+)	#
1	72.04	54.03	55.02	36.52	A					14
2	169.10	151.09	152.07	85.05	P	1607.73	1589.71	1590.70	804.36	13
3	256.13	238.12	239.10	128.56	S	1510.67	1492.66	1493.64	755.83	12
4	419.19	401.18	402.17	210.10	Y	1423.64	1405.63	1406.63	712.32	11
5	506.23	488.21	489.20	253.61	S	1260.58	1242.57	1243.55	630.79	10
6	621.25	603.24	604.23	311.13	D	1173.54	1155.54	1156.53	587.27	9
7	784.32	766.41	767.29	392.66	Y	1058.52	1040.51	1041.49	529.76	8
8	913.36	895.45	896.33	457.18	E	895.45	877.44	878.42	448.23	7
9	1000.39	982.38	983.36	500.69	S	766.41	748.40	749.38	383.70	6
10	1113.47	1095.46	1096.45	557.24	I	679.38	661.37	662.35	340.19	5
11	1269.58	1251.56	1252.55	635.29	R	566.30	548.28	549.27	283.65	4
12	1384.61	1366.59	1367.58	692.80	D	410.19	392.18	393.17	205.60	3
13	1497.69	1479.67	1480.66	749.34	L	295.17	277.15	278.14	148.08	2
14					Y	182.08	164.07	165.05	91.54	1

short neuropeptide F-PP-1 (sNPF-PP-1)_part 2

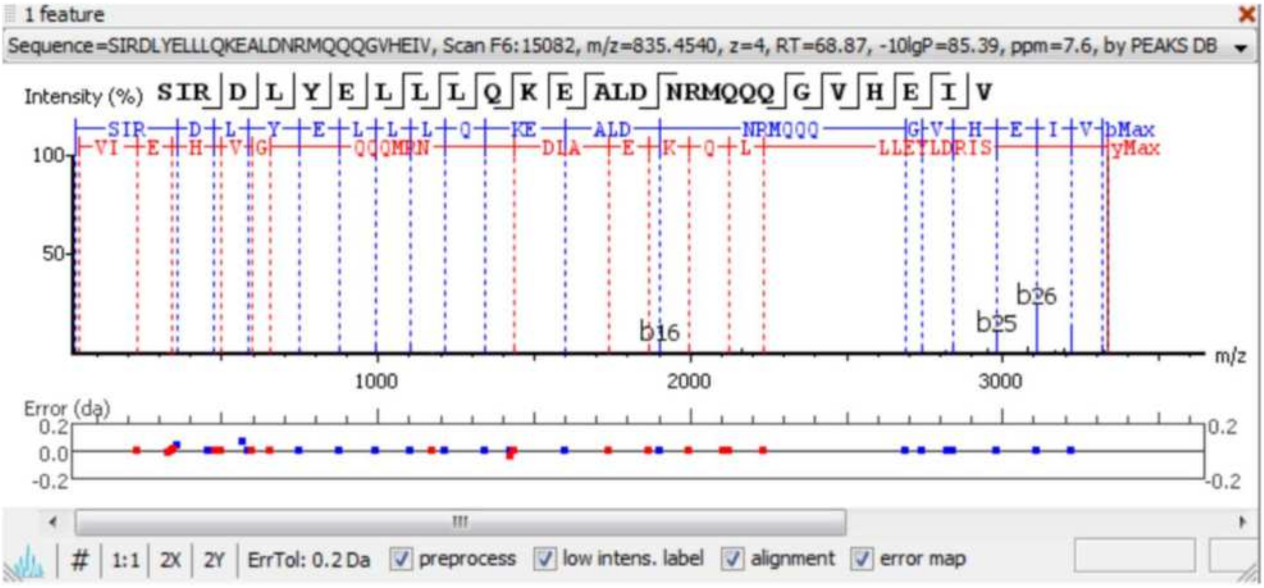

#	b	b-H2O	b-NH3	b (2+)	Seq	y	y-H2O	y-NH3	y (2+)	#
1	88.04	70.03	71.01	44.52	S					28
2	201.12	183.11	184.10	101.06	I	3251.72	3233.71	3234.69	1626.36	27
3	357.18	339.21	340.20	179.11	R	3138.64	3120.63	3121.61	1569.82	26
4	472.26	454.23	455.22	236.63	D	2982.54	2964.52	2965.51	1491.77	25
5	585.34	567.25	568.31	293.17	L	2867.51	2849.50	2850.48	1434.25	24
6	748.40	730.39	731.37	374.70	Y	2754.42	2736.41	2737.40	1377.71	23
7	877.44	859.43	860.41	439.22	E	2591.36	2573.35	2574.33	1296.18	22
8	990.53	972.52	973.50	495.76	L	2462.32	2444.31	2445.29	1231.66	21
9	1103.61	1085.60	1086.58	552.30	L	2349.23	2331.22	2332.21	1175.12	20
10	1216.70	1198.68	1199.67	608.85	L	2236.16	2218.14	2219.12	1118.58	19
11	1344.76	1326.74	1327.73	672.88	Q	2123.07	2105.06	2106.04	1062.03	18
12	1472.85	1454.84	1455.82	736.92	K	1995.01	1977.00	1977.98	998.00	17
13	1601.88	1583.88	1584.86	801.45	E	1866.92	1848.90	1849.89	933.96	16
14	1672.93	1654.92	1655.90	836.96	A	1737.87	1719.86	1720.84	869.43	15
15	1786.01	1768.00	1768.98	893.51	L	1666.83	1648.82	1649.81	833.92	14
16	1901.04	1883.03	1884.01	951.02	D	1553.75	1535.74	1536.72	777.37	13
17	2015.08	1997.07	1998.05	1008.04	N	1438.72	1420.71	1421.74	719.86	12
18	2171.18	2153.17	2154.16	1086.09	R	1324.68	1306.67	1307.65	662.84	11
19	2302.22	2284.21	2285.20	1151.61	M	1168.58	1150.57	1151.55	584.79	10
20	2430.28	2412.27	2413.25	1215.64	Q	1037.54	1019.53	1020.51	519.27	9
21	2558.34	2540.33	2541.31	1279.67	Q	909.48	891.47	892.45	455.24	8
22	2686.39	2668.39	2669.37	1343.70	Q	781.42	763.41	764.39	391.21	7
23	2743.43	2725.41	2726.39	1372.21	G	653.37	635.35	636.33	327.20	6
24	2842.49	2824.48	2825.46	1421.74	V	596.34	578.33	579.31	298.67	5
25	2979.55	2961.54	2962.52	1490.27	H	497.27	479.26	480.24	249.14	4
26	3108.60	3090.58	3091.56	1554.80	E	360.21	342.21	343.16	180.61	3
27	3221.68	3203.66	3204.65	1611.34	I	231.17	213.16	214.14	116.09	2
28					V	118.09	100.08	101.06	59.54	1

short neuropeptide F-PP-2 (sNPF-PP-2)

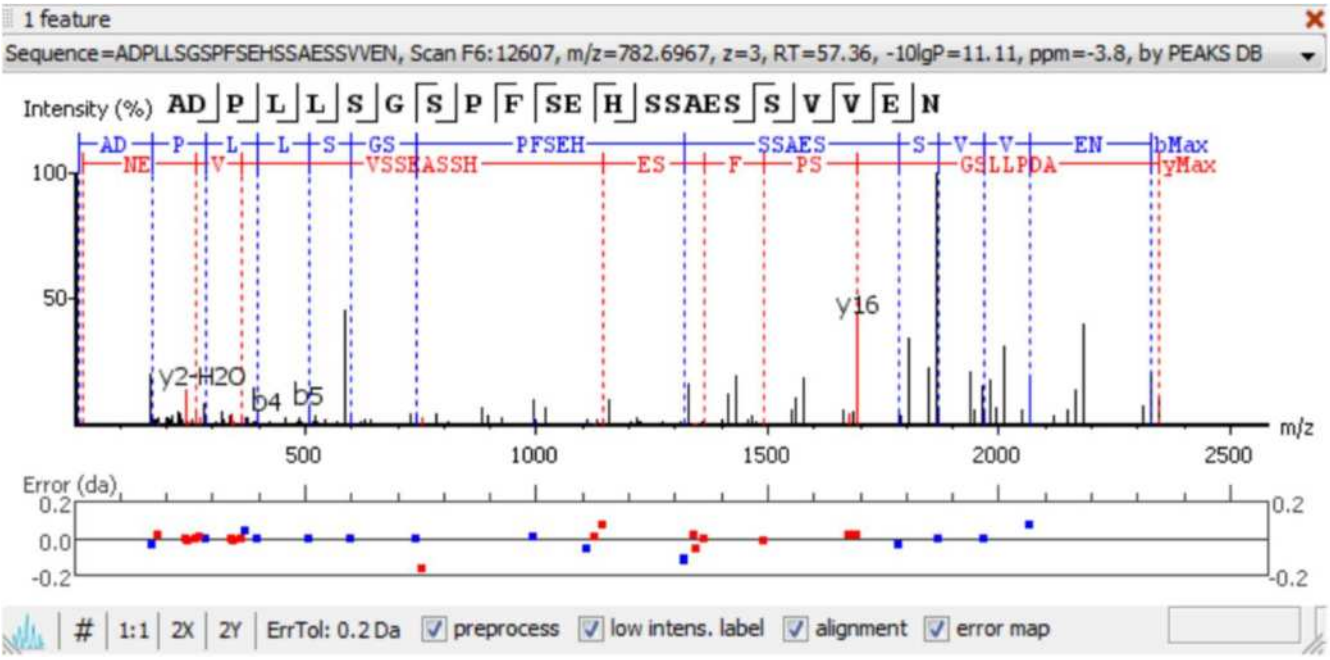

#	b	b-H2O	b-NH3	b (2+)	Seq	y	y-H2O	y-NH3	y (2+)	#
1	72.04	54.03	55.02	36.52	A					23
2	187.07	169.10	170.04	94.04	D	2275.04	2257.03	2258.01	1138.02	22
3	284.12	266.11	267.10	142.56	P	2160.01	2142.00	2142.98	1080.50	21
4	397.21	379.20	380.18	199.10	L	2062.96	2044.95	2045.93	1031.98	20
5	510.29	492.28	493.27	255.65	L	1949.87	1931.86	1932.85	975.44	19
6	597.33	579.31	580.30	299.16	S	1836.79	1818.78	1819.76	918.89	18
7	654.35	636.34	637.32	327.67	G	1749.76	1731.75	1732.73	875.38	17
8	741.38	723.37	724.35	371.14	S	1692.71	1674.70	1675.71	846.87	16
9	838.43	820.42	821.40	419.72	P	1605.70	1587.69	1588.68	803.35	15
10	985.50	967.49	968.47	493.25	F	1508.65	1490.66	1491.62	754.99	14
11	1072.53	1054.52	1055.50	536.77	S	1361.57	1343.54	1344.61	681.29	13
12	1201.57	1183.56	1184.55	601.29	E	1274.55	1256.54	1257.52	637.77	12
13	1338.63	1320.74	1321.73	669.82	H	1145.43	1127.50	1128.46	573.25	11
14	1425.67	1407.65	1408.64	713.33	S	1008.45	990.44	991.42	504.72	10
15	1512.70	1494.69	1495.67	756.85	S	921.42	903.41	904.39	461.21	9
16	1583.73	1565.72	1566.71	792.37	A	834.38	816.37	817.36	417.69	8
17	1712.78	1694.77	1695.75	856.89	E	763.35	745.34	746.32	382.17	7
18	1799.81	1781.84	1782.78	900.40	S	634.30	616.29	617.28	317.65	6
19	1886.84	1868.83	1869.81	943.92	S	547.27	529.26	530.25	274.12	5
20	1985.91	1967.91	1968.88	993.44	V	460.24	442.23	443.21	230.62	4
21	2084.98	2066.97	2067.86	1042.99	V	361.17	343.16	344.16	181.06	3
22	2214.02	2196.01	2196.99	1107.57	E	262.10	244.09	245.10	131.55	2
23					N	133.06	115.05	116.03	67.03	1

short neuropeptide F (sNPF)

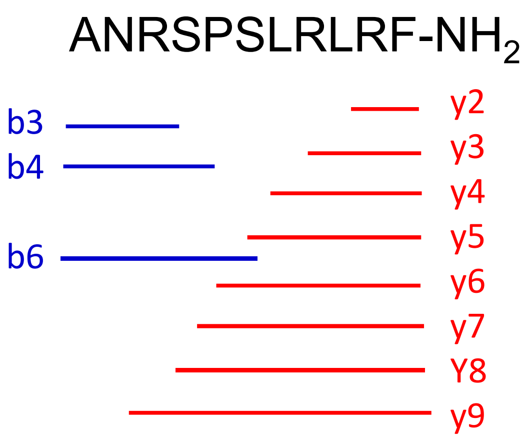

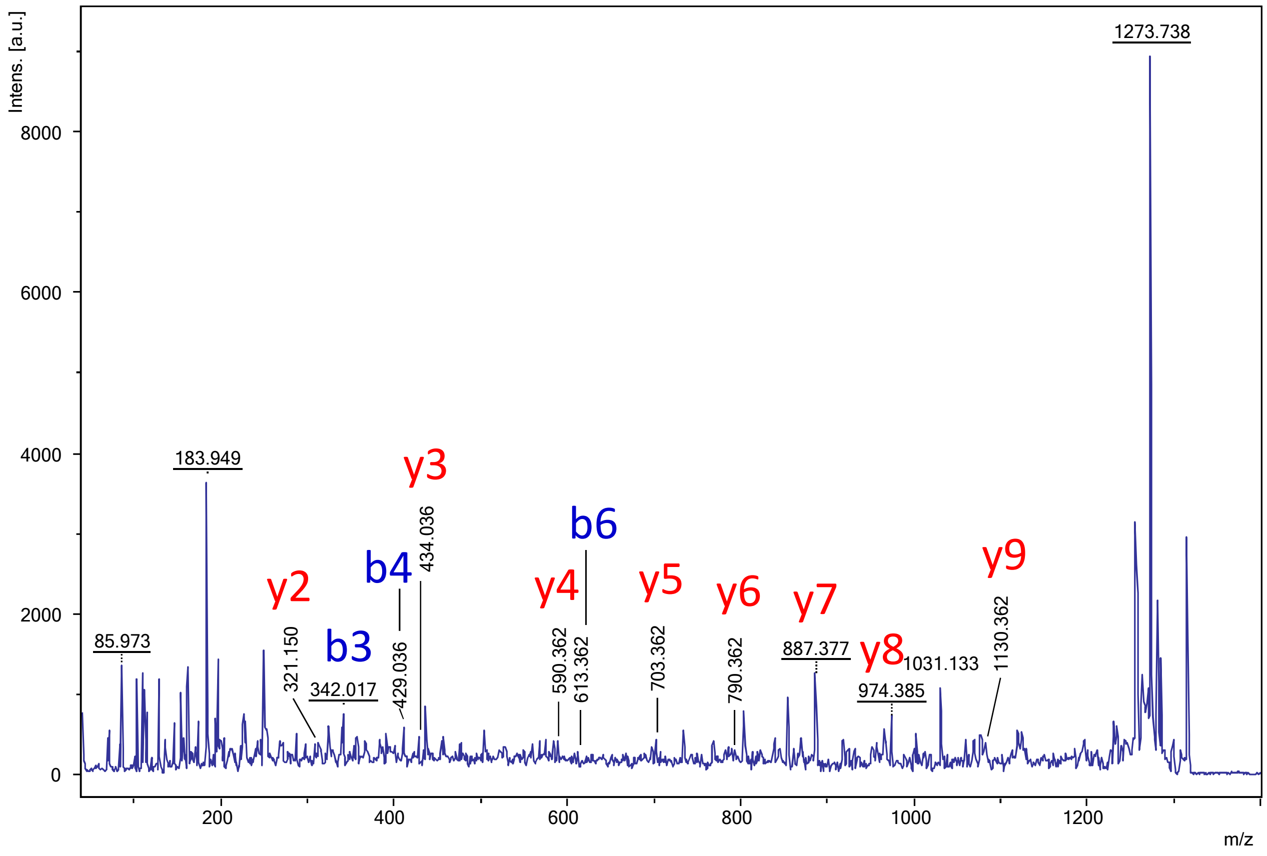

SMYamide_partial

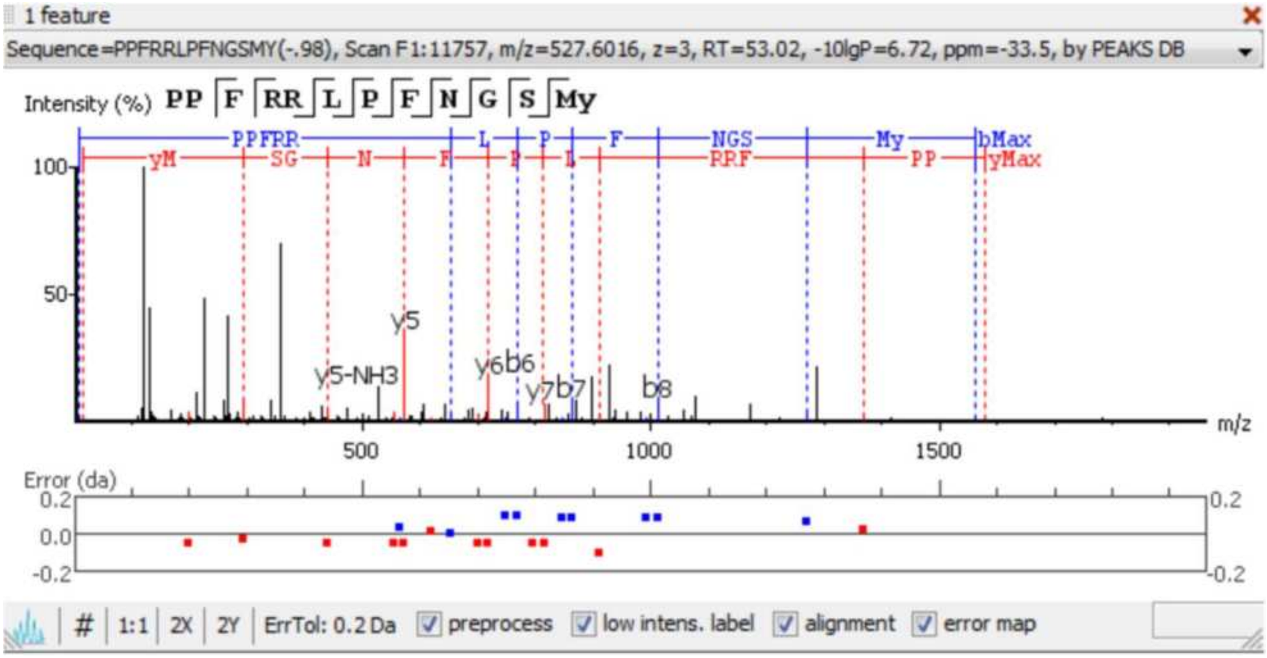

#	b	b-H2O	b-NH3	b (2+)	Seq	y	y-H2O	y-NH3	y (2+)	#
1	98.06	80.05	81.03	49.53	P					13
2	195.11	177.10	178.09	98.06	P	1483.76	1465.75	1466.74	742.38	12
3	342.18	324.17	325.15	171.59	F	1386.71	1368.67	1369.68	693.85	11
4	498.28	480.27	481.26	249.64	R	1239.64	1221.63	1222.61	620.31	10
5	654.38	636.37	637.36	327.69	R	1083.54	1065.53	1066.51	542.27	9
6	767.37	749.36	750.44	384.23	L	927.44	909.54	910.41	464.22	8
7	864.43	846.42	847.49	432.76	P	814.41	796.41	797.33	407.68	7
8	1011.50	993.49	994.56	506.29	F	717.36	699.35	700.28	359.15	6
9	1125.63	1107.62	1108.61	563.28	N	570.29	552.22	553.26	285.62	5
10	1182.65	1164.64	1165.63	591.83	G	456.19	438.23	439.16	228.60	4
11	1269.61	1251.68	1252.66	635.34	S	399.17	381.16	382.14	200.14	3
12	1400.73	1382.72	1383.70	700.86	M	312.14	294.13	295.14	156.57	2
13					Y(-.98)	181.10	163.09	164.07	91.05	1

Sulfakinin-PP-1 (SK-PP-1)_partial

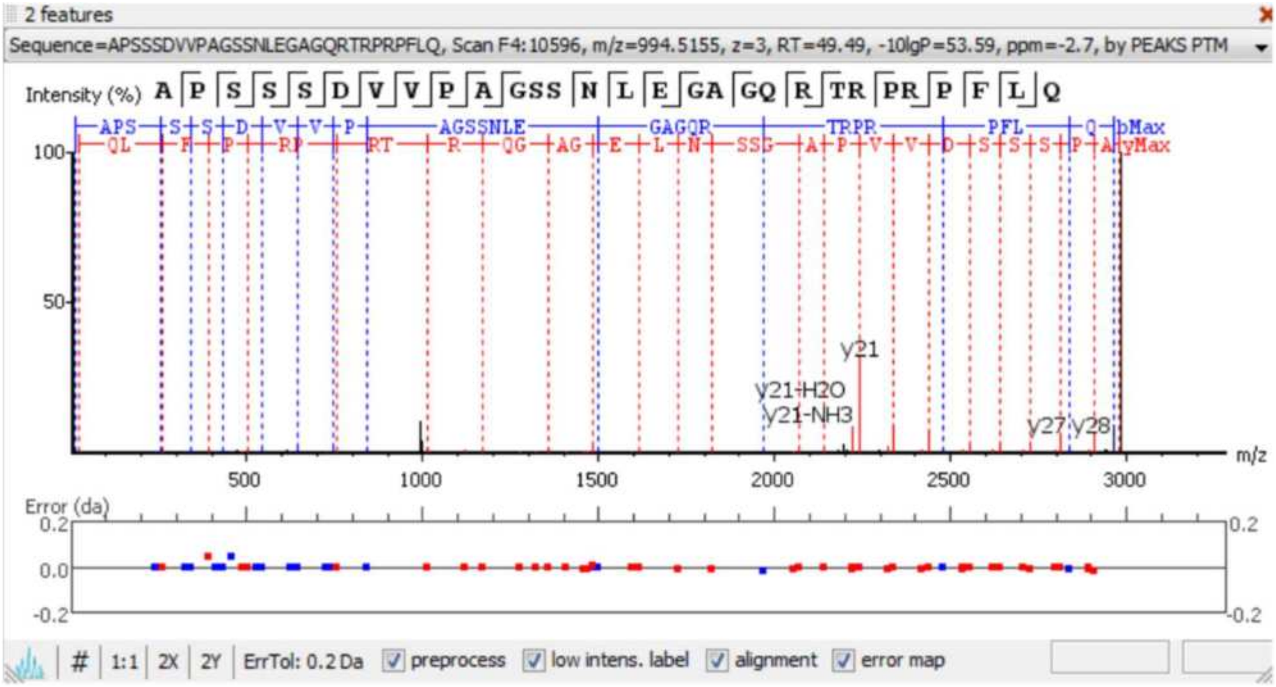

#	b	b-H2O	b-NH3	b (2+)	Seq	y	y-H2O	y-NH3	y (2+)	#
1	72.04	54.03	55.02	36.52	A					29
2	169.10	151.09	152.07	85.05	P	2910.49	2892.47	2893.45	1455.76	28
3	256.13	238.12	239.10	128.56	S	2813.43	2795.41	2796.41	1407.21	27
4	343.16	325.15	326.13	172.08	S	2726.40	2708.37	2709.37	1363.70	26
5	430.19	412.18	413.17	215.60	S	2639.37	2621.35	2622.34	1320.19	25
6	545.22	527.21	528.19	273.11	D	2552.34	2534.31	2535.32	1276.67	24
7	644.29	626.28	627.26	322.64	V	2437.31	2419.30	2420.29	1219.16	23
8	743.36	725.35	726.33	372.18	V	2338.24	2320.22	2321.22	1169.62	22
9	840.41	822.40	823.38	420.71	P	2239.16	2221.16	2222.15	1120.09	21
10	911.44	893.44	894.42	456.17	A	2142.12	2124.11	2125.10	1071.56	20
11	968.47	950.46	951.44	484.73	G	2071.08	2053.07	2054.05	1036.04	19
12	1055.50	1037.49	1038.47	528.25	S	2014.06	1996.05	1997.03	1007.53	18
13	1142.53	1124.52	1125.51	571.77	S	1927.03	1909.02	1910.00	964.01	17
14	1256.58	1238.57	1239.55	628.79	N	1839.99	1821.98	1822.97	920.50	16
15	1369.66	1351.65	1352.63	685.33	L	1725.96	1707.94	1708.92	863.48	15
16	1498.71	1480.69	1481.68	749.85	E	1612.87	1594.85	1595.84	806.93	14
17	1555.72	1537.71	1538.70	778.36	G	1483.81	1465.81	1466.80	742.41	13
18	1626.76	1608.75	1609.73	813.88	A	1426.80	1408.79	1409.78	713.90	12
19	1683.78	1665.77	1666.76	842.39	G	1355.77	1337.75	1338.74	678.38	11
20	1811.84	1793.83	1794.81	906.42	Q	1298.74	1280.73	1281.72	649.87	10
21	1967.94	1949.93	1950.92	984.47	R	1170.69	1152.67	1153.66	585.84	9
22	2068.99	2050.98	2051.96	1034.99	T	1014.59	996.57	997.56	507.79	8
23	2225.09	2207.08	2208.06	1113.05	R	913.54	895.53	896.51	457.27	7
24	2322.14	2304.13	2305.12	1161.57	P	757.44	739.42	740.41	379.22	6
25	2478.24	2460.23	2461.22	1239.62	R	660.38	642.37	643.36	330.69	5
26	2575.30	2557.29	2558.27	1288.15	P	504.28	486.27	487.25	252.64	4
27	2722.37	2704.36	2705.34	1361.68	F	407.23	389.17	390.20	204.11	3
28	2835.45	2817.44	2818.42	1418.23	L	260.16	242.15	243.13	130.58	2
29					Q	147.08	129.07	130.05	74.04	1

Sulfakinin-PP-2 (SK-PP-2)_partial

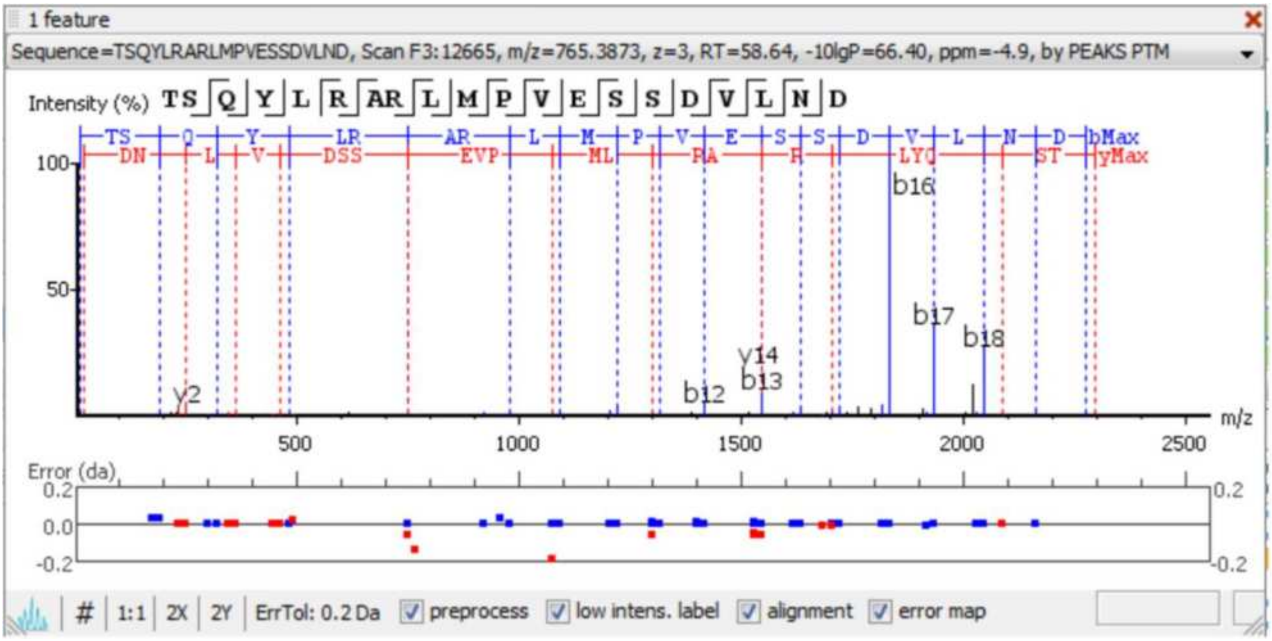

#	b	b-H2O	b-NH3	b (2+)	Seq	y	y-H2O	y-NH3	y (2+)	#
1	102.06	84.04	85.03	51.53	T					20
2	189.05	171.04	172.06	95.04	S	2193.10	2175.09	2176.07	1097.05	19
3	317.15	299.14	300.12	159.07	Q	2106.06	2088.05	2089.04	1053.53	18
4	480.21	462.20	463.18	240.60	Y	1978.01	1960.00	1960.98	989.50	17
5	593.29	575.28	576.27	297.15	L	1814.94	1796.93	1797.92	907.97	16
6	749.39	731.38	732.37	375.20	R	1701.87	1683.86	1684.83	851.43	15
7	820.43	802.42	803.40	410.72	A	1545.82	1527.80	1528.79	773.38	14
8	976.53	958.49	959.51	488.77	R	1474.72	1456.71	1457.69	737.86	13
9	1089.62	1071.61	1072.59	545.31	L	1318.62	1300.68	1301.59	659.81	12
10	1220.65	1202.65	1203.63	610.83	M	1205.54	1187.52	1188.51	603.27	11
11	1317.71	1299.69	1300.68	659.36	P	1074.69	1056.48	1057.47	537.75	10
12	1416.78	1398.76	1399.76	708.89	V	977.44	959.43	960.42	489.20	9
13	1545.82	1527.80	1528.79	773.41	E	878.37	860.36	861.35	439.69	8
14	1632.85	1614.84	1615.83	816.93	S	749.39	731.32	732.30	375.17	7
15	1719.89	1701.87	1702.86	860.44	S	662.30	644.29	645.27	331.65	6
16	1834.91	1816.90	1817.88	917.96	D	575.27	557.26	558.24	288.13	5
17	1933.98	1915.97	1916.97	967.49	V	460.24	442.23	443.21	230.62	4
18	2047.06	2029.06	2030.04	1024.03	L	361.17	343.16	344.15	181.09	3
19	2161.11	2143.10	2144.08	1081.05	N	248.09	230.08	231.06	124.54	2
20					D	134.04	116.03	117.02	67.52	1

Sulfakinin-1 (SK-1)_[Q]

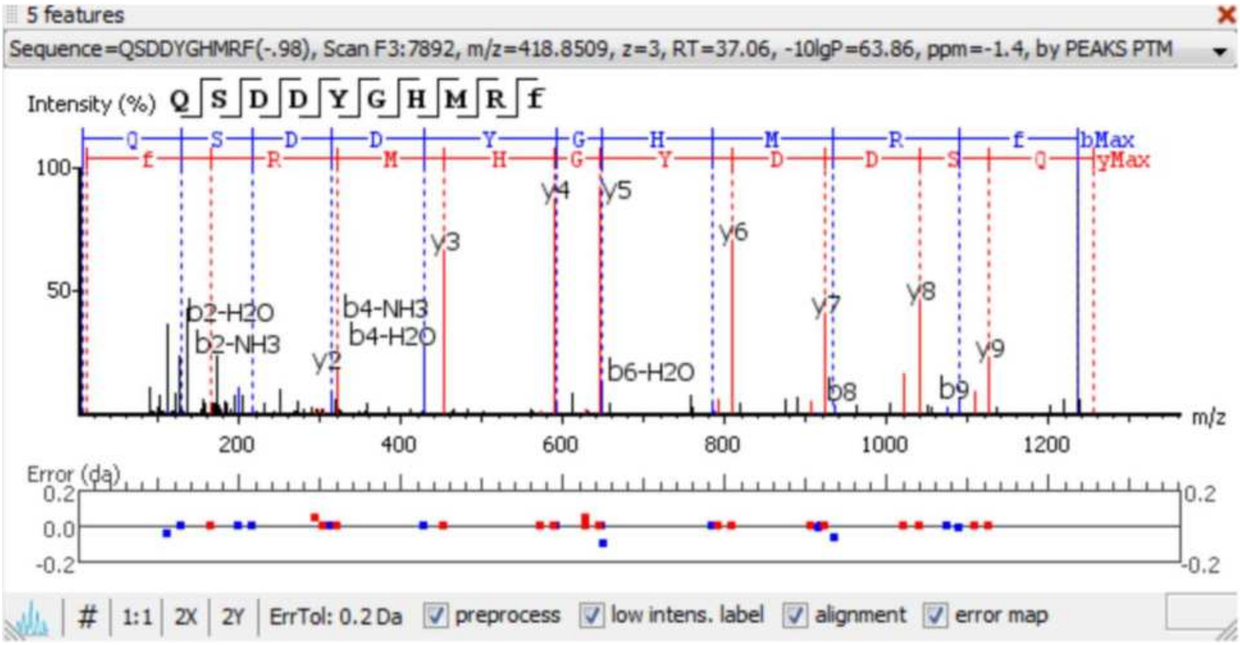

#	b	b-H2O	b-NH3	b (2+)	Seq	y	y-H2O	y-NH3	y (2+)	#
1	129.07	111.06	112.09	65.03	Q					10
2	216.10	198.09	199.07	108.55	S	1126.47	1108.46	1109.45	563.74	9
3	331.13	313.11	314.10	166.06	D	1039.44	1021.43	1022.41	520.22	8
4	446.15	428.14	429.12	223.58	D	924.42	906.40	907.40	462.71	7
5	609.22	591.21	592.19	305.11	Y	809.39	791.38	792.36	405.19	6
6	666.24	648.23	649.32	333.62	G	646.32	628.27	629.30	323.66	5
7	803.30	785.28	786.27	402.15	H	589.30	571.29	572.28	295.10	4
8	934.40	916.34	917.31	467.67	M	452.24	434.23	435.22	226.62	3
9	1090.45	1072.43	1073.41	545.72	R	321.20	303.19	304.18	161.10	2
10					F(-.98)	165.10	147.09	148.08	83.05	1

Sulfakinin-1 (SK-1)_[Q]_SO_3

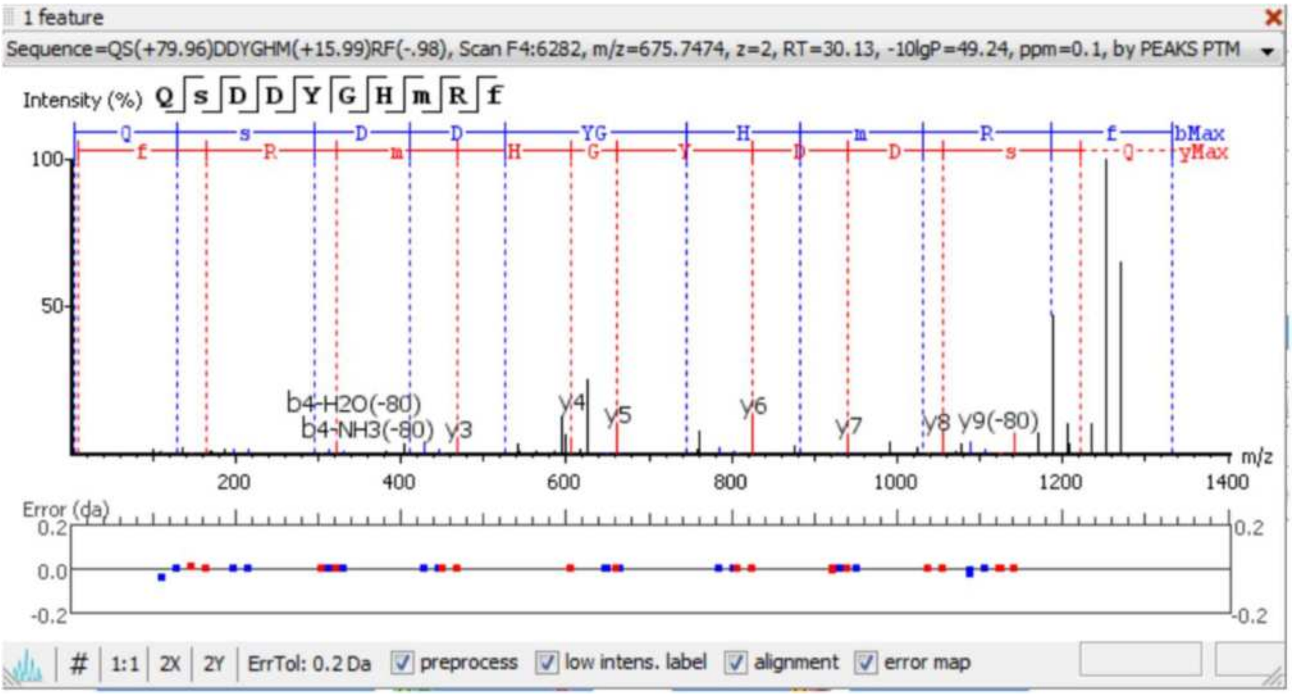

#	b	b-H2O	b-NH3	b (2+)	Seq	y	y-H2O	y-NH3	y (2+)	#
1	129.07	111.06	112.09	65.03	Q					10
2	296.06	278.04	279.03	148.53	S(+79.96)	1222.43	1204.41	1205.40	611.71	9
3	411.08	393.07	394.06	206.04	D	1055.44	1037.42	1038.41	528.22	8
4	526.11	508.10	509.08	263.55	D	940.41	922.40	923.39	470.70	7
5	689.17	671.16	672.15	345.09	Y	825.38	807.37	808.36	413.19	6
6	746.19	728.18	729.17	373.60	G	662.32	644.31	645.29	331.66	5
7	883.25	865.24	866.23	442.13	H	605.30	587.29	588.27	303.15	4
8	1030.29	1012.28	1013.26	515.64	M(+15.99)	468.24	450.23	451.21	234.62	3
9	1186.39	1168.38	1169.36	593.69	R	321.20	303.19	304.18	161.10	2
10					F(-.98)	165.10	147.08	148.06	83.05	1

Sulfakinin-1 (SK-1)_[pQ]

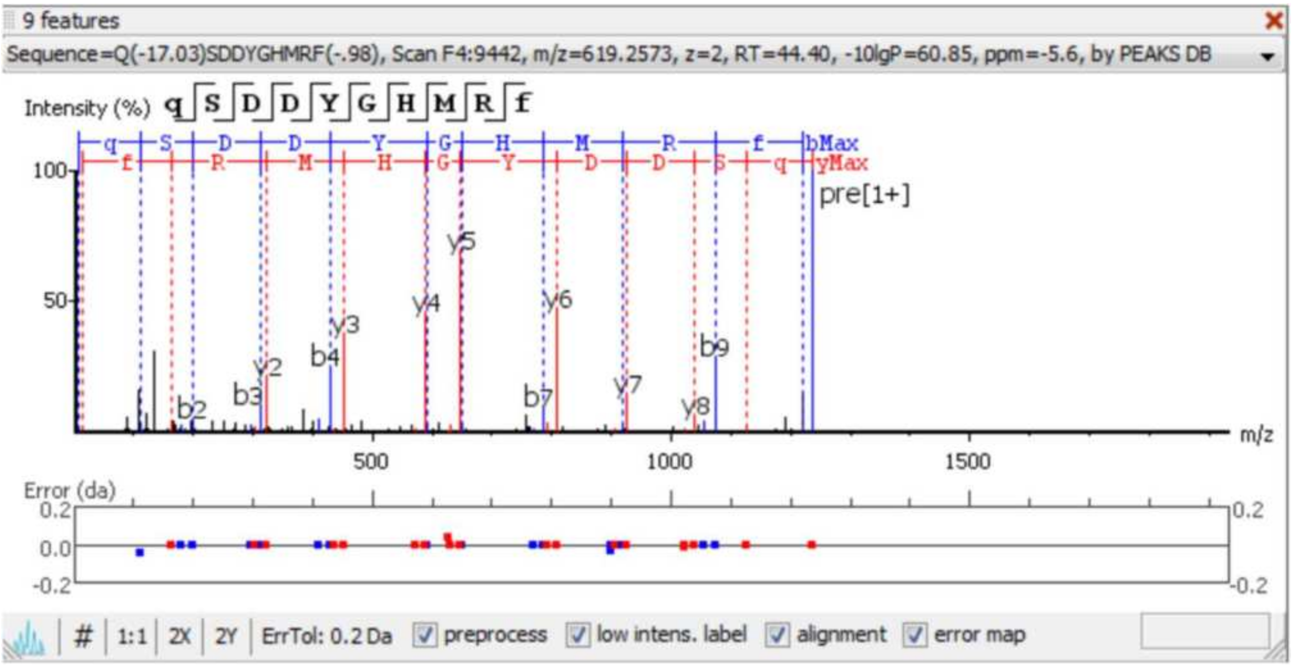

#	b	b-H2O	b-NH3	b (2+)	Seq	y	y-H2O	y-NH3	y (2+)	#
1	112.09	94.03	95.01	56.52	Q(-17.03)					10
2	199.07	181.06	182.04	100.04	S	1126.47	1108.46	1109.45	563.74	9
3	314.10	296.09	297.07	157.55	D	1039.45	1021.43	1022.43	520.22	8
4	429.13	411.11	412.10	215.06	D	924.42	906.40	907.39	462.71	7
5	592.19	574.18	575.16	296.59	Y	809.39	791.38	792.36	405.19	6
6	649.21	631.20	632.18	325.11	G	646.32	628.27	629.30	323.66	5
7	786.27	768.26	769.24	393.63	H	589.30	571.29	572.28	295.15	4
8	917.31	899.30	900.32	459.15	M	452.24	434.23	435.22	226.62	3
9	1073.41	1055.39	1056.39	537.21	R	321.20	303.19	304.18	161.10	2
10					F(-.98)	165.10	147.09	148.08	83.05	1

Sulfakinin-1 (SK-1)_[pQ]_SO_3

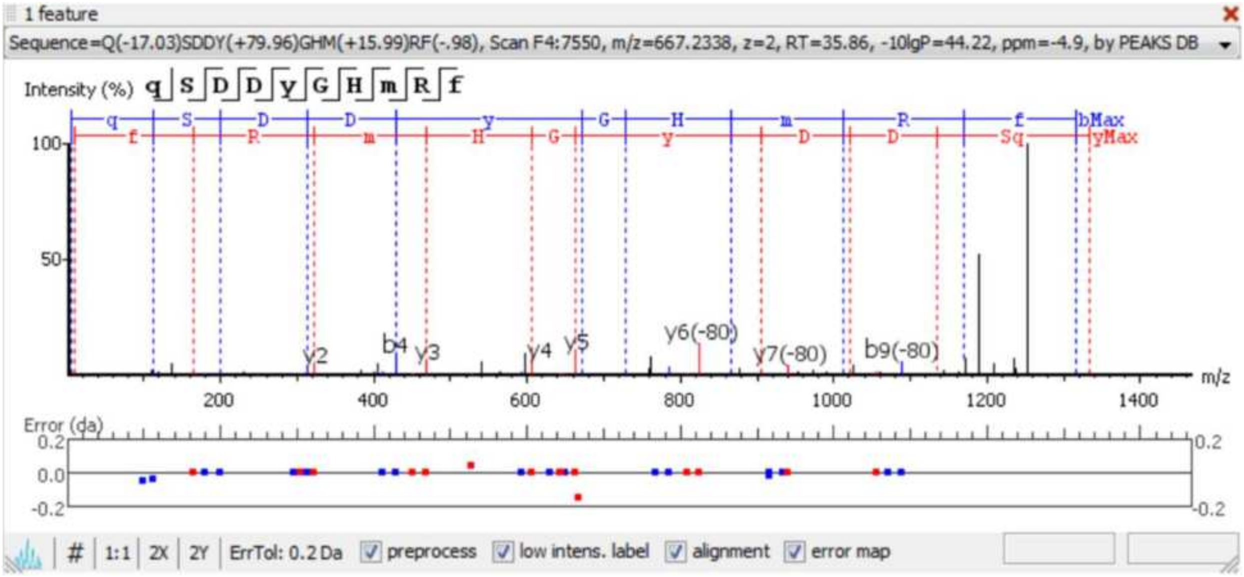

#	b	b-H2O	b-NH3	b (2+)	Seq	y	y-H2O	y-NH3	y (2+)	#
1	112.09	94.03	95.01	56.52	Q(-17.03)					10
2	199.07	181.06	182.04	100.09	S	1222.43	1204.41	1205.40	611.71	9
3	314.10	296.09	297.07	157.55	D	1135.39	1117.38	1118.37	568.20	8
4	429.13	411.12	412.10	215.06	D	1020.37	1002.36	1003.34	510.68	7
5	672.15	654.14	655.12	336.57	Y(+79.96)	905.34	887.33	888.31	453.17	6
6	729.17	711.16	712.14	365.08	G	662.32	644.31	645.29	331.66	5
7	866.23	848.22	849.20	433.61	H	605.30	587.29	588.27	303.15	4
8	1013.26	995.25	996.23	507.13	M(+15.99)	468.24	450.23	451.21	234.62	3
9	1169.36	1151.35	1152.34	585.18	R	321.20	303.19	304.18	161.10	2
10					F(-.98)	165.10	147.09	148.08	83.05	1

Sulfakinin-2 (SK-2)_[E]

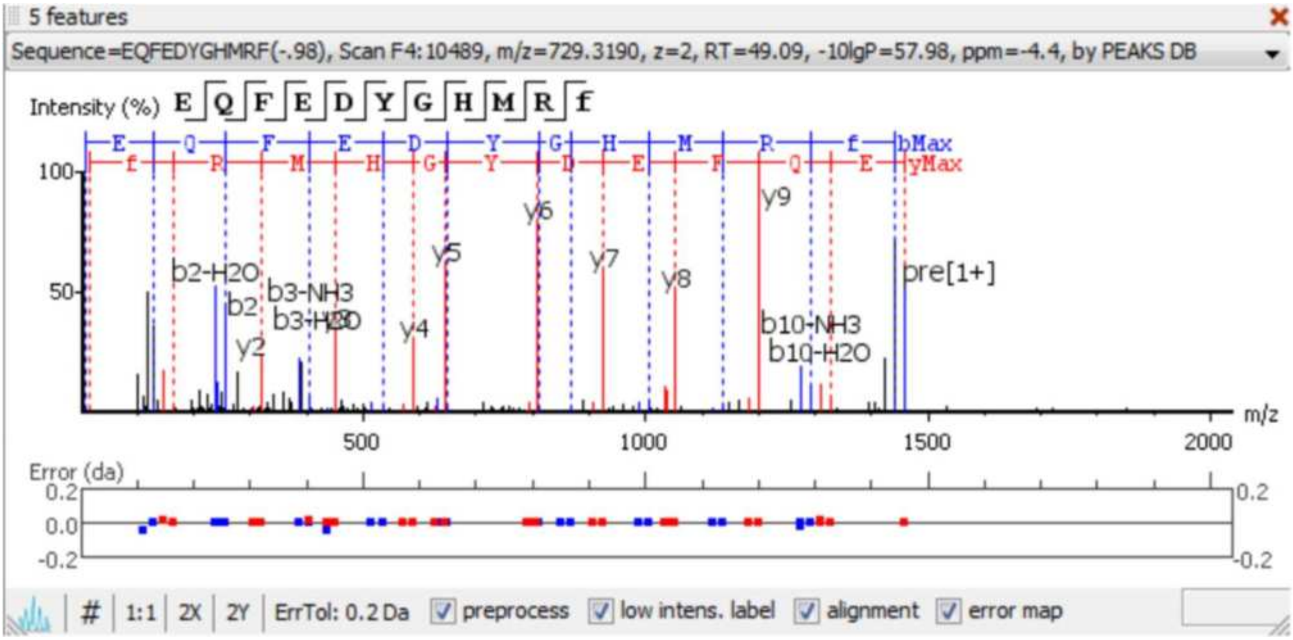

#	b	b-H2O	b-NH3	b (2+)	Seq	y	y-H2O	y-NH3	y (2+)	#
1	130.05	112.09	113.02	65.53	E					11
2	258.11	240.10	241.08	129.55	Q	1328.59	1310.56	1311.56	664.79	10
3	405.18	387.17	388.15	203.09	F	1200.53	1182.52	1183.50	600.76	9
4	534.22	516.21	517.19	267.61	E	1053.46	1035.45	1036.44	527.23	8
5	649.25	631.24	632.22	325.12	D	924.41	906.40	907.39	462.71	7
6	812.31	794.30	795.28	406.66	Y	809.39	791.38	792.36	405.18	6
7	869.33	851.32	852.30	435.22	G	646.32	628.31	629.30	323.66	5
8	1006.39	988.38	989.36	503.70	H	589.30	571.29	572.28	295.15	4
9	1137.43	1119.42	1120.40	569.22	M	452.24	434.23	435.22	226.62	3
10	1293.54	1275.52	1276.53	647.27	R	321.20	303.19	304.18	161.10	2
11					F(-.98)	165.10	147.08	148.08	83.05	1

Sulfakinin-2 (SK-2)_[E]_SO_3

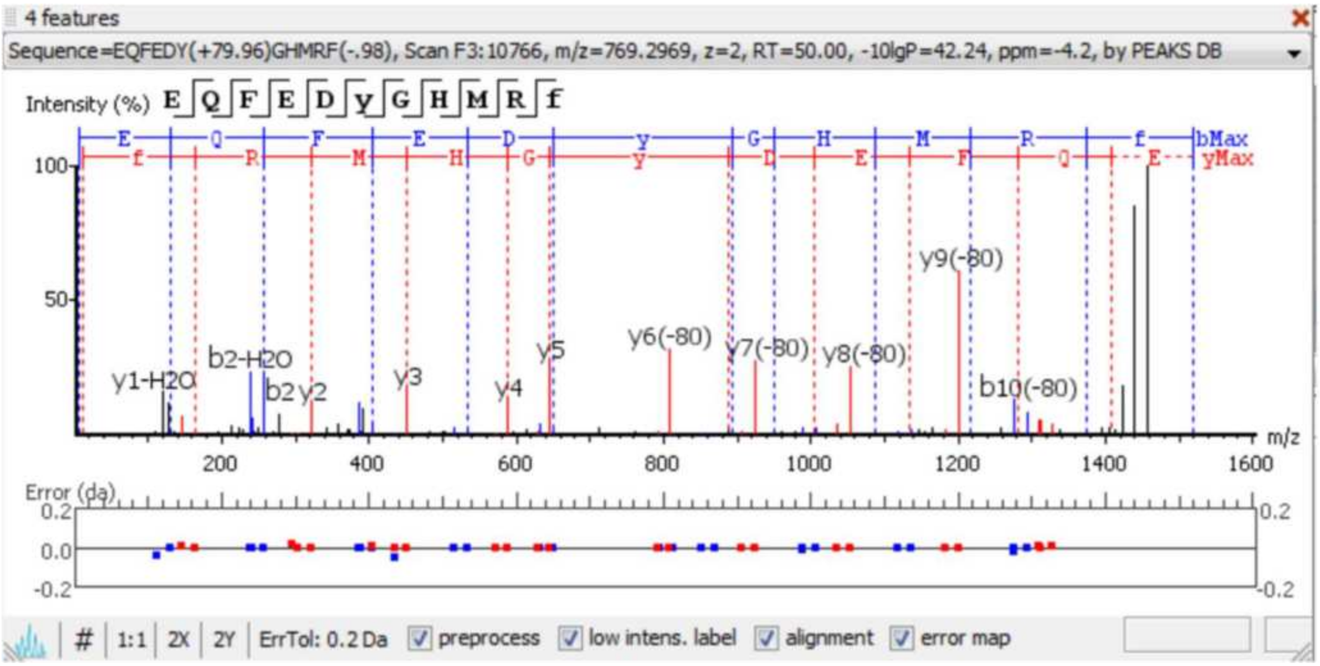

#	b	b-H2O	b-NH3	b (2+)	Seq	y	y-H2O	y-NH3	y (2+)	#
1	130.05	112.09	113.02	65.53	E					11
2	258.11	240.10	241.08	129.55	Q	1408.54	1390.53	1391.51	704.77	10
3	405.18	387.17	388.15	203.09	F	1280.48	1262.47	1263.46	640.74	9
4	534.22	516.21	517.19	267.61	E	1133.41	1115.40	1116.39	567.21	8
5	649.25	631.24	632.22	325.12	D	1004.37	986.36	987.34	502.69	7
6	892.27	874.26	875.24	446.63	Y(+79.96)	889.34	871.33	872.32	445.17	6
7	949.29	931.28	932.26	475.14	G	646.32	628.31	629.30	323.66	5
8	1086.35	1068.34	1069.32	543.67	H	589.30	571.29	572.28	295.13	4
9	1217.39	1199.38	1200.36	609.19	M	452.24	434.23	435.22	226.62	3
10	1373.49	1355.48	1356.46	687.24	R	321.20	303.19	304.18	161.10	2
11					F(-.98)	165.10	147.08	148.08	83.05	1

Sulfakinin-2 (SK-2)_[pE]

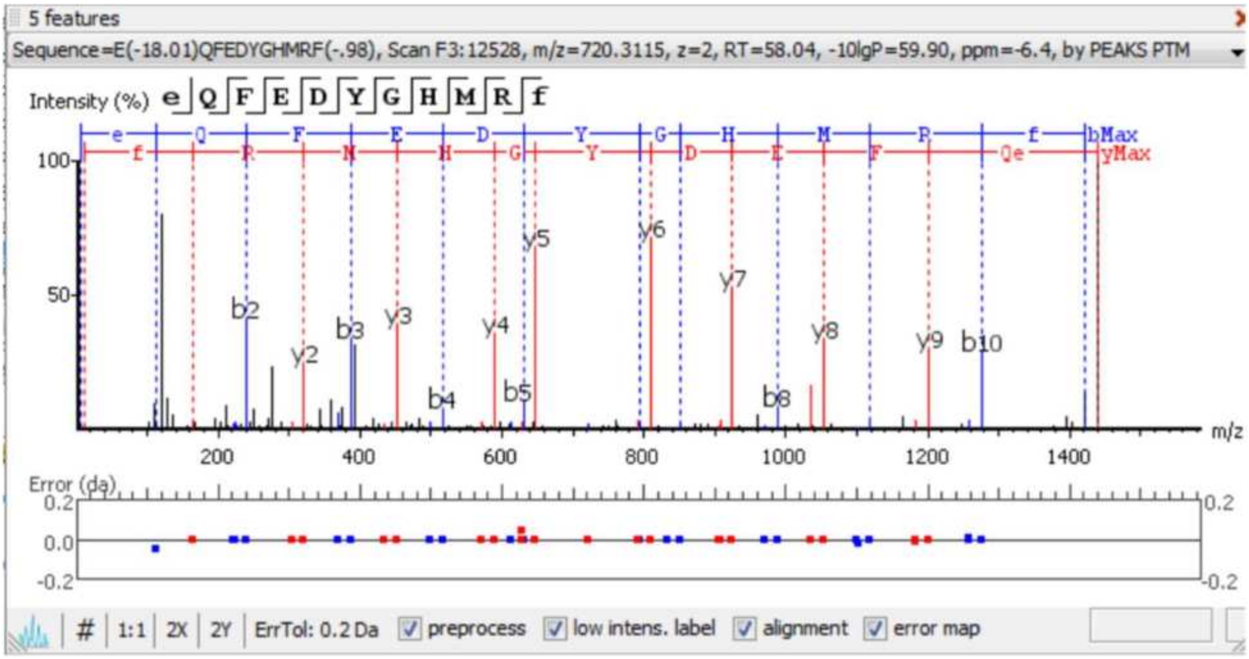

#	b	b-H2O	b-NH3	b (2+)	Seq	y	y-H2O	y-NH3	y (2+)	#
1	112.09	94.03	95.01	56.52	E(-18.01)					11
2	240.10	222.09	223.07	120.55	Q	1328.58	1310.57	1311.56	664.79	10
3	387.17	369.16	370.14	194.08	F	1200.53	1182.52	1183.51	600.76	9
4	516.21	498.20	499.18	258.60	E	1053.46	1035.45	1036.44	527.23	8
5	631.24	613.23	614.21	316.12	D	924.41	906.40	907.39	462.71	7
6	794.30	776.29	777.27	397.65	Y	809.39	791.38	792.36	405.19	6
7	851.32	833.31	834.29	426.16	G	646.32	628.27	629.30	323.66	5
8	988.38	970.37	971.36	494.69	H	589.30	571.29	572.28	295.15	4
9	1119.42	1101.42	1102.42	560.21	M	452.24	434.23	435.22	226.62	3
10	1275.52	1257.50	1258.50	638.26	R	321.20	303.19	304.18	161.10	2
11					F(-.98)	165.10	147.09	148.08	83.05	1

Sulfakinin-2 (SK-2)_[pE]_SO_3

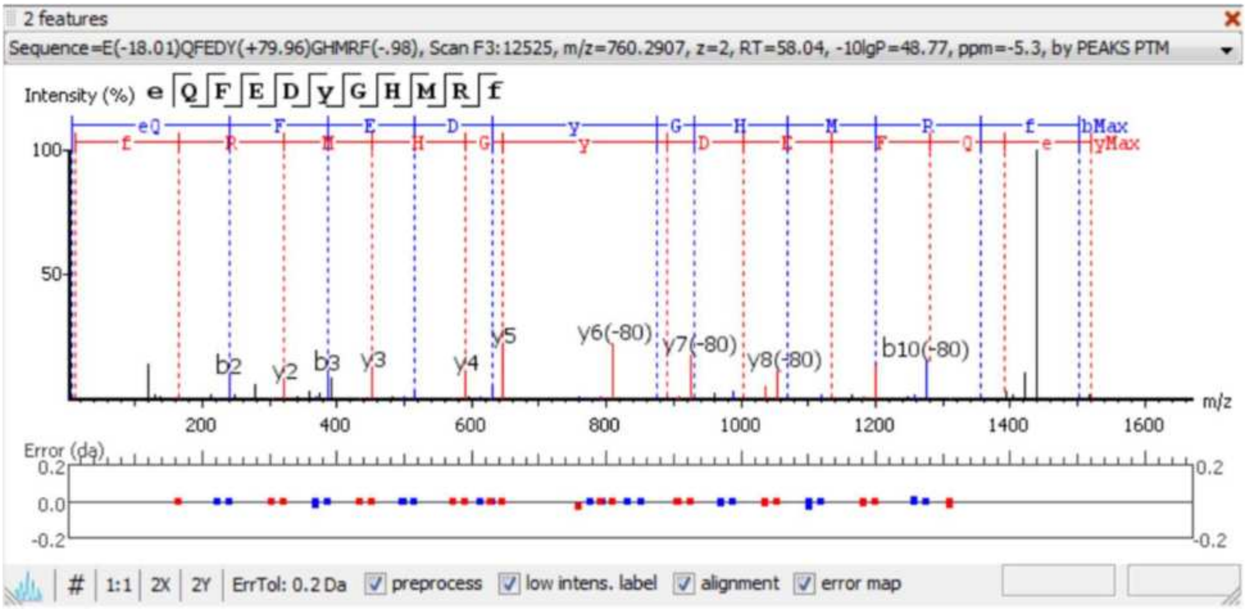

#	b	b-H2O	b-NH3	b (2+)	Seq	y	y-H2O	y-NH3	y (2+)	#
1	112.04	94.03	95.01	56.52	E(-18.01)					11
2	240.10	222.09	223.07	120.55	Q	1408.54	1390.53	1391.51	704.77	10
3	387.17	369.18	370.14	194.08	F	1280.48	1262.47	1263.46	640.74	9
4	516.21	498.20	499.18	258.60	E	1133.41	1115.40	1116.39	567.21	8
5	631.24	613.23	614.21	316.12	D	1004.37	986.36	987.34	502.69	7
6	874.26	856.25	857.23	437.63	Y(+79.96)	889.34	871.33	872.32	445.17	6
7	931.28	913.27	914.25	466.14	G	646.32	628.31	629.30	323.66	5
8	1068.34	1050.33	1051.31	534.67	H	589.30	571.29	572.28	295.15	4
9	1199.38	1181.37	1182.35	600.19	M	452.24	434.23	435.22	226.62	3
10	1355.48	1337.47	1338.45	678.24	R	321.20	303.19	304.18	161.10	2
11					F(-.98)	165.10	147.09	148.08	83.05	1

Tachykinin-related peptide-1$^{9\text{-}17}$ (TK-1$^{9\text{-}17}$)

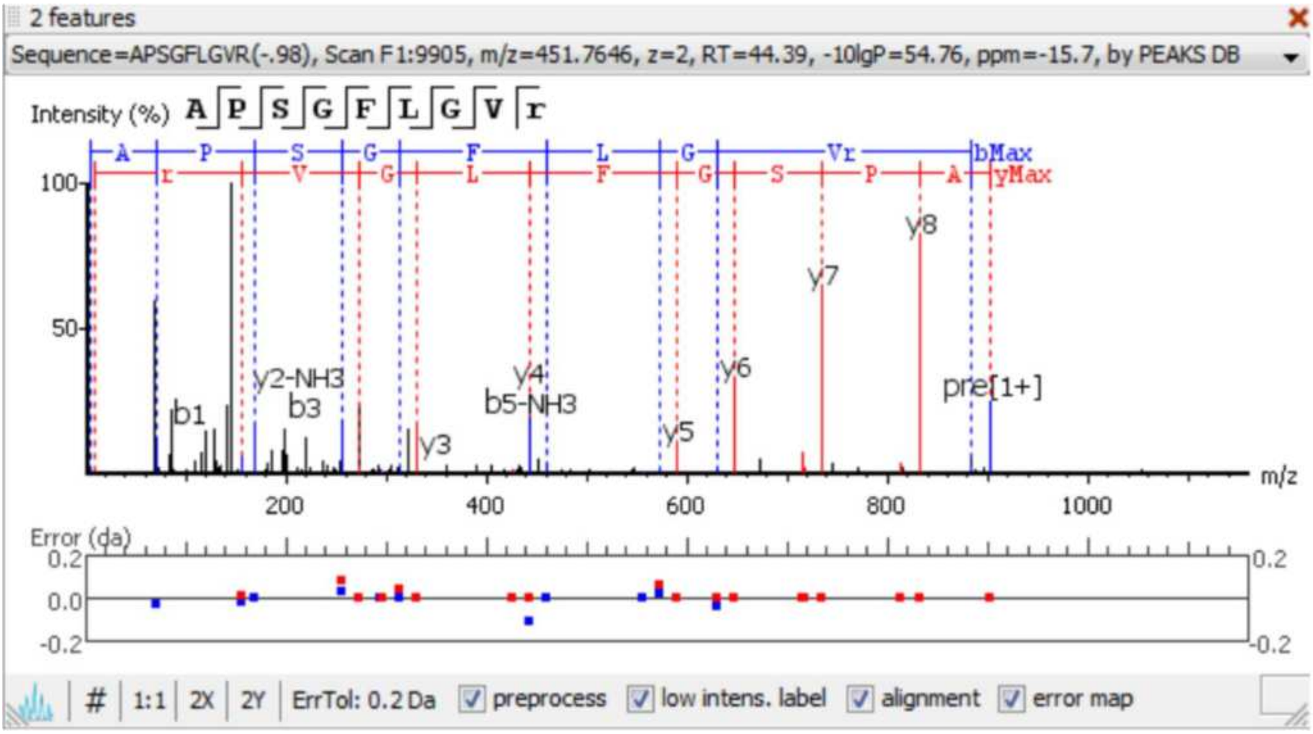

#	b	b-H2O	b-NH3	b (2+)	Seq	y	y-H2O	y-NH3	y (2+)	#
1	72.08	54.03	55.02	36.52	A					9
2	169.10	151.09	152.07	85.05	P	831.48	813.47	814.46	416.24	8
3	256.09	238.12	239.10	128.56	S	734.43	716.42	717.41	367.72	7
4	313.15	295.14	296.12	157.10	G	647.40	629.39	630.37	324.20	6
5	460.22	442.21	443.31	230.61	F	590.38	572.37	573.28	295.69	5
6	573.28	555.29	556.28	287.15	L	443.31	425.30	426.28	222.15	4
7	630.37	612.31	613.30	315.66	G	330.22	312.21	313.15	165.61	3
8	729.39	711.38	712.37	365.20	V	273.20	255.19	256.09	137.10	2
9					R(-.98)	174.13	156.12	157.10	87.57	1

Tachykinin-related peptide-2 (TK-2)

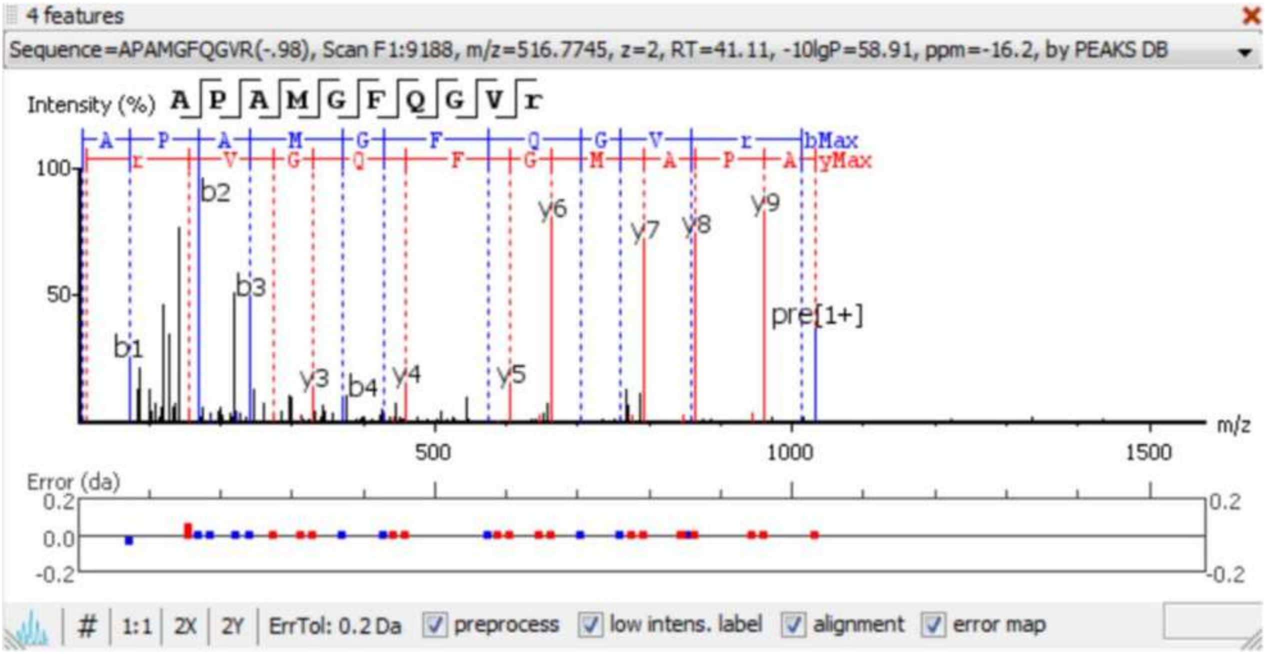

#	b	b-H2O	b-NH3	b (2+)	Seq	y	y-H2O	y-NH3	y (2+)	#
1	72.08	54.03	55.02	36.52	A					10
2	169.10	151.09	152.07	85.05	P	961.50	943.49	944.48	481.25	9
3	240.13	222.12	223.11	120.57	A	864.45	846.44	847.42	432.73	8
4	371.17	353.16	354.15	186.09	M	793.41	775.41	776.39	397.21	7
5	428.20	410.19	411.17	214.60	G	662.37	644.36	645.35	331.69	6
6	575.26	557.25	558.24	288.13	F	605.35	587.34	588.33	303.18	5
7	703.32	685.31	686.30	352.16	Q	458.28	440.27	441.26	229.64	4
8	760.35	742.33	743.32	380.67	G	330.22	312.21	313.20	165.61	3
9	859.41	841.40	842.39	430.21	V	273.20	255.19	256.18	137.10	2
10					R(-.98)	174.13	156.08	157.11	87.57	1

Tachykinin-related peptide-3 (TK-3)

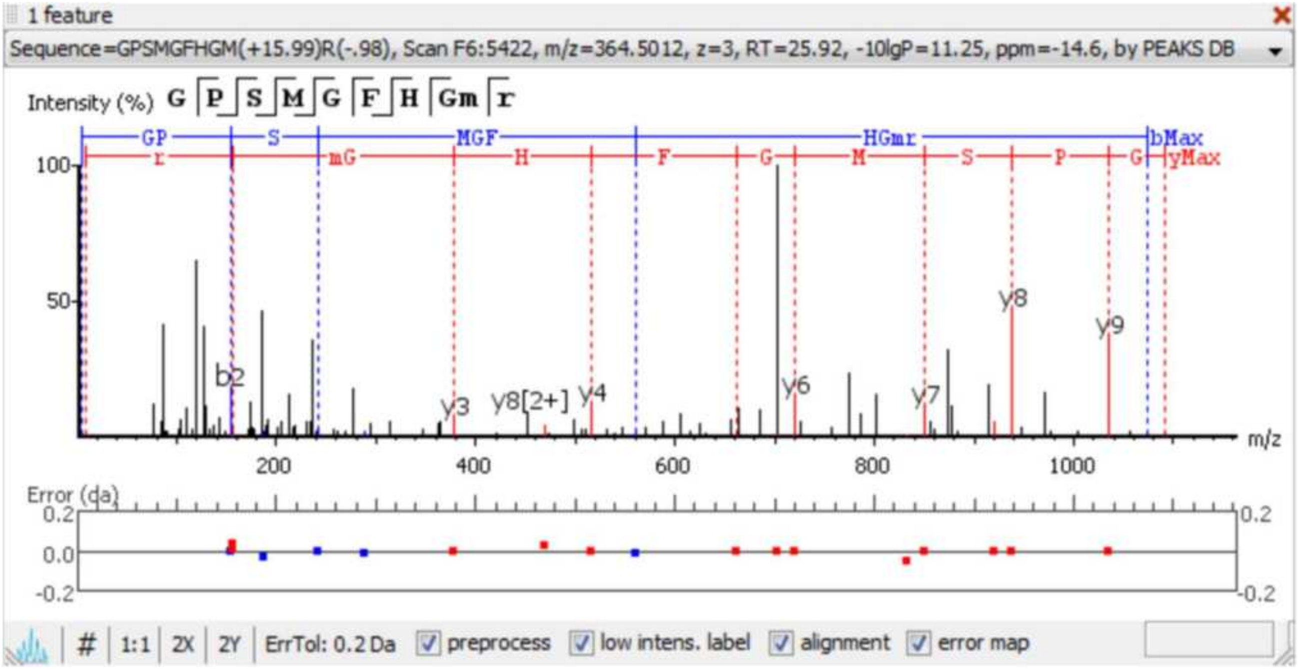

#	b	b-H2O	b-NH3	b (2+)	Seq	y	y-H2O	y-NH3	y (2+)	#
1	58.03	40.02	41.00	29.51	G					10
2	155.08	137.07	138.06	78.04	P	1034.47	1016.46	1017.44	517.73	9
3	242.11	224.10	225.09	121.56	S	937.41	919.40	920.39	469.17	8
4	373.15	355.14	356.13	187.11	M	850.38	832.42	833.35	425.69	7
5	430.18	412.17	413.15	215.59	G	719.34	701.33	702.31	360.17	6
6	577.24	559.23	560.23	289.14	F	662.32	644.31	645.29	331.66	5
7	714.30	696.29	697.28	357.65	H	515.25	497.24	498.22	258.13	4
8	771.32	753.31	754.30	386.16	G	378.19	360.18	361.16	189.60	3
9	918.36	900.35	901.33	459.68	M(+15.99)	321.17	303.16	304.14	161.09	2
10					R(-.98)	174.13	156.08	157.10	87.57	1

Tachykinin-related peptide-5 (TK-5)

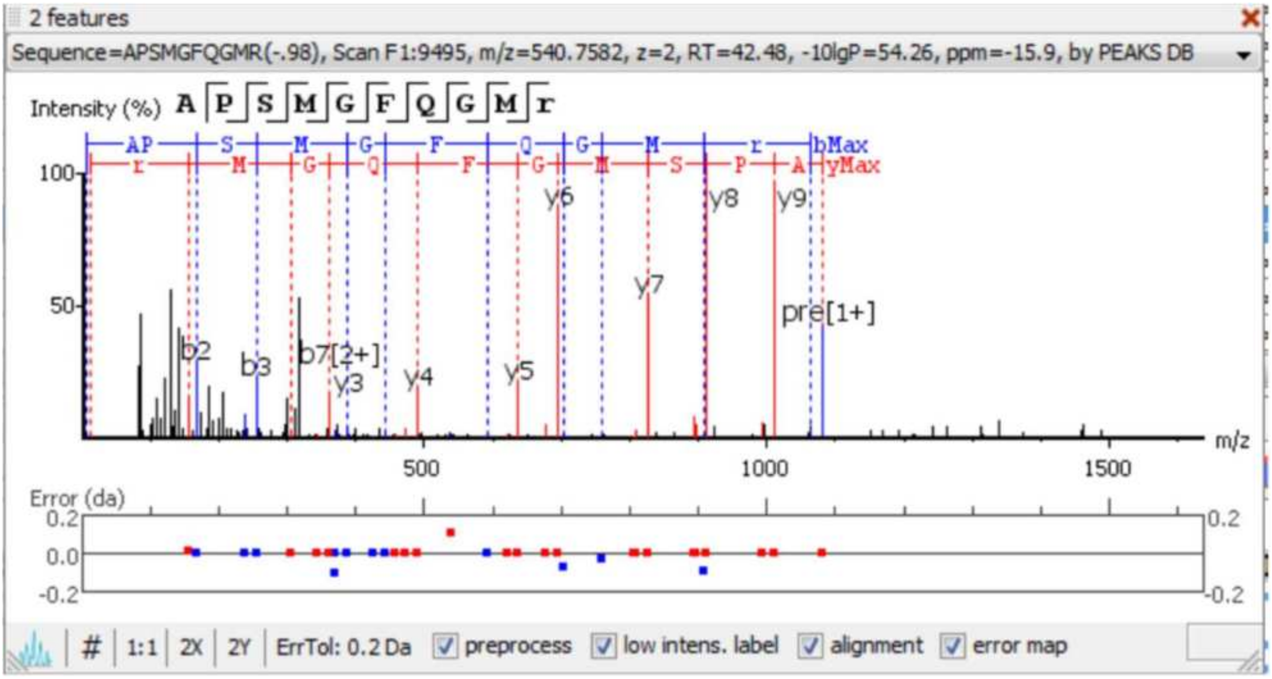

#	b	b-H2O	b-NH3	b (2+)	Seq	y	y-H2O	y-NH3	y (2+)	#
1	72.04	54.03	55.02	36.52	A					10
2	169.10	151.09	152.07	85.05	P	1009.47	991.46	992.45	505.24	9
3	256.13	238.12	239.10	128.56	S	912.42	894.40	895.40	456.71	8
4	387.17	369.16	370.26	194.09	M	825.39	807.37	808.36	413.19	7
5	444.19	426.18	427.16	222.60	G	694.34	676.33	677.32	347.67	6
6	591.26	573.25	574.23	296.13	F	637.32	619.31	620.30	319.16	5
7	719.32	701.31	702.37	360.15	Q	490.26	472.24	473.23	245.63	4
8	776.34	758.37	759.31	388.67	G	362.20	344.19	345.17	181.60	3
9	907.49	889.37	890.35	454.19	M	305.18	287.16	288.15	153.09	2
10					R(-.98)	174.13	156.12	157.10	87.57	1

Tachykinin-related peptide-7 (TK-7)

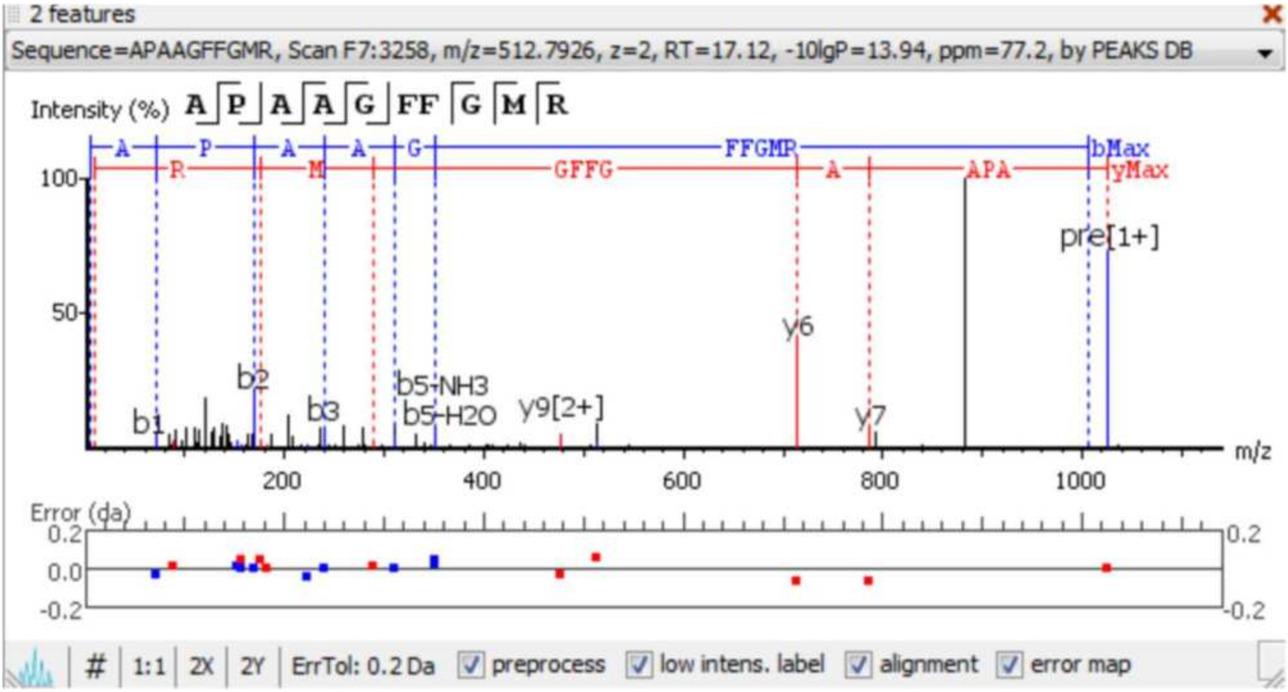

#	b	b-H2O	b-NH3	b (2+)	Seq	y	y-H2O	y-NH3	y (2+)	#
1	72.08	54.03	55.02	36.52	A					10
2	169.10	151.09	152.06	85.05	P	953.47	935.46	936.44	477.27	9
3	240.13	222.12	223.15	120.57	A	856.41	838.40	839.39	428.71	8
4	311.17	293.16	294.14	156.08	A	785.45	767.37	768.35	393.19	7
5	368.19	350.13	351.14	184.60	G	714.41	696.33	697.31	357.67	6
6	515.26	497.25	498.23	258.13	F	657.32	639.31	640.29	329.16	5
7	662.33	644.32	645.30	331.67	F	510.25	492.24	493.22	255.62	4
8	719.35	701.34	702.32	360.18	G	363.18	345.17	346.15	182.10	3
9	850.39	832.38	833.37	425.70	M	306.16	288.15	289.11	153.58	2
10					R	175.07	157.06	158.09	88.04	1

Tachykinin-related peptide-8 (TK-8)

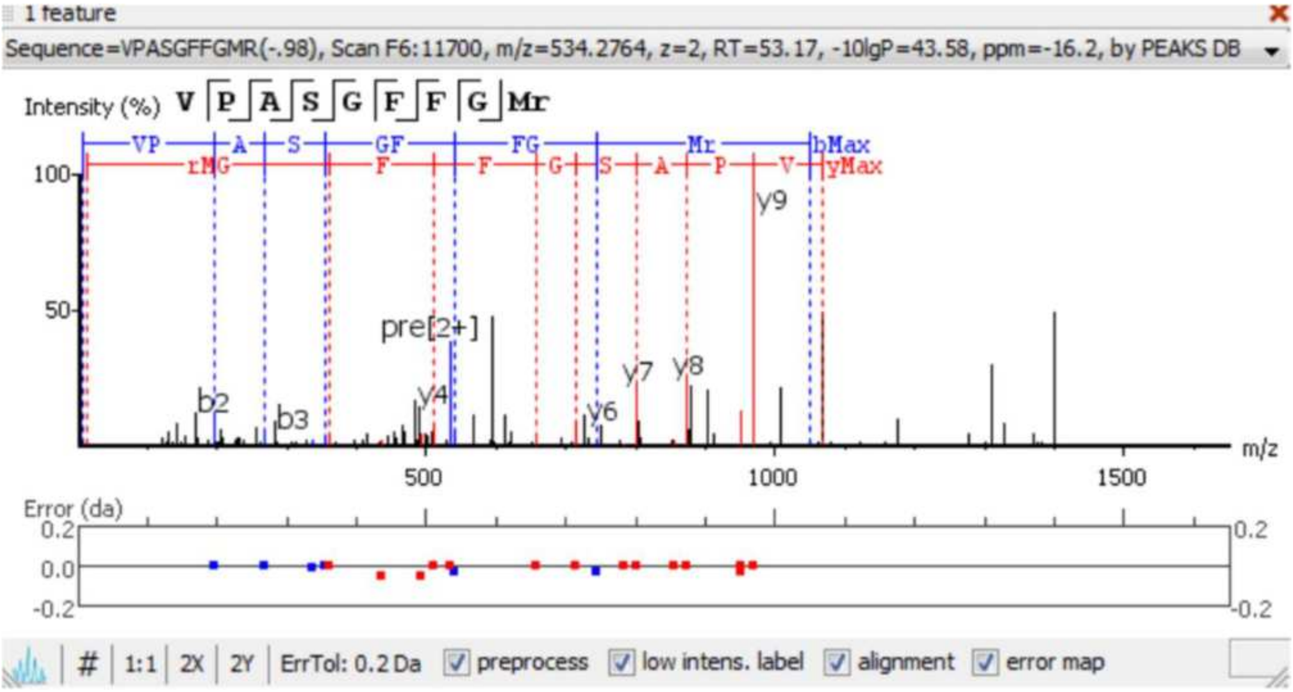

#	b	b-H2O	b-NH3	b (2+)	Seq	y	y-H2O	y-NH3	y (2+)	#
1	100.08	82.07	83.05	50.54	V					10
2	197.13	179.05	180.10	99.06	P	968.47	950.47	951.49	484.74	9
3	268.17	250.15	251.15	134.58	A	871.43	853.41	854.48	436.27	8
4	355.20	337.20	338.20	178.10	S	800.39	782.38	783.36	400.70	7
5	412.22	394.21	395.20	206.61	G	713.36	695.34	696.33	357.18	6
6	559.29	541.31	542.31	280.18	F	656.41	638.35	639.36	328.67	5
7	706.36	688.40	689.36	353.71	F	509.27	491.32	492.24	255.13	4
8	763.38	745.40	746.35	382.19	G	362.20	344.20	345.17	181.60	3
9	894.48	876.51	877.39	447.71	M	305.18	287.18	288.15	153.14	2
10					R(-.98)	174.14	156.08	157.13	87.57	1

Tachykinin-related peptide-10 (TK-10)

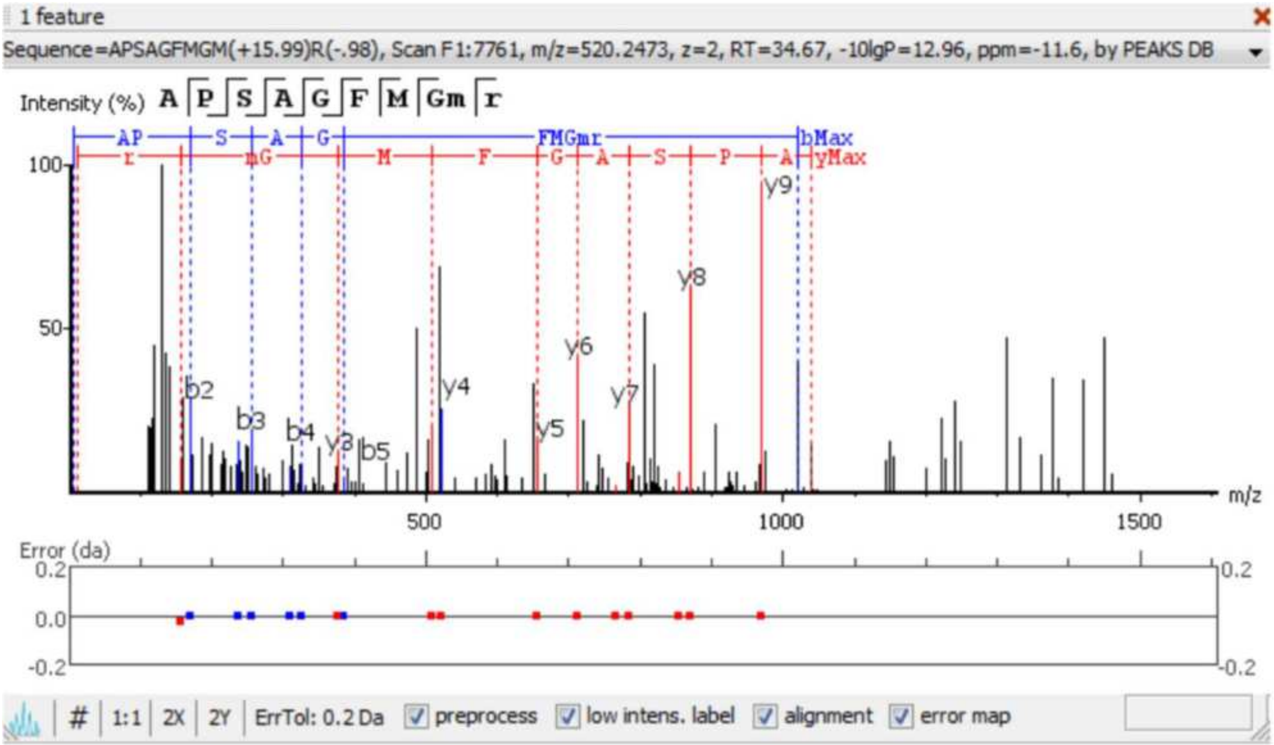

#	b	b-H2O	b-NH3	b (2+)	Seq	y	y-H2O	y-NH3	y (2+)	#
1	72.04	54.03	55.02	36.52	A					10
2	169.10	151.09	152.07	85.05	P	968.44	950.43	951.42	484.72	9
3	256.13	238.12	239.10	128.56	S	871.39	853.39	854.36	436.20	8
4	327.17	309.16	310.14	164.08	A	784.36	766.36	767.33	392.68	7
5	384.19	366.18	367.16	192.59	G	713.32	695.31	696.30	357.16	6
6	531.26	513.25	514.23	266.13	F	656.30	638.29	639.27	328.65	5
7	662.30	644.29	645.27	331.65	M	509.23	491.22	492.21	255.12	4
8	719.32	701.31	702.29	360.16	G	378.19	360.18	361.16	189.60	3
9	866.35	848.34	849.33	433.68	M(+15.99)	321.17	303.16	304.14	161.09	2
10					R(-.98)	174.13	156.12	157.13	87.57	1

Tachykinin-related peptide-12$^{1\text{-}8}$ (TK-12$^{1\text{-}8}$)

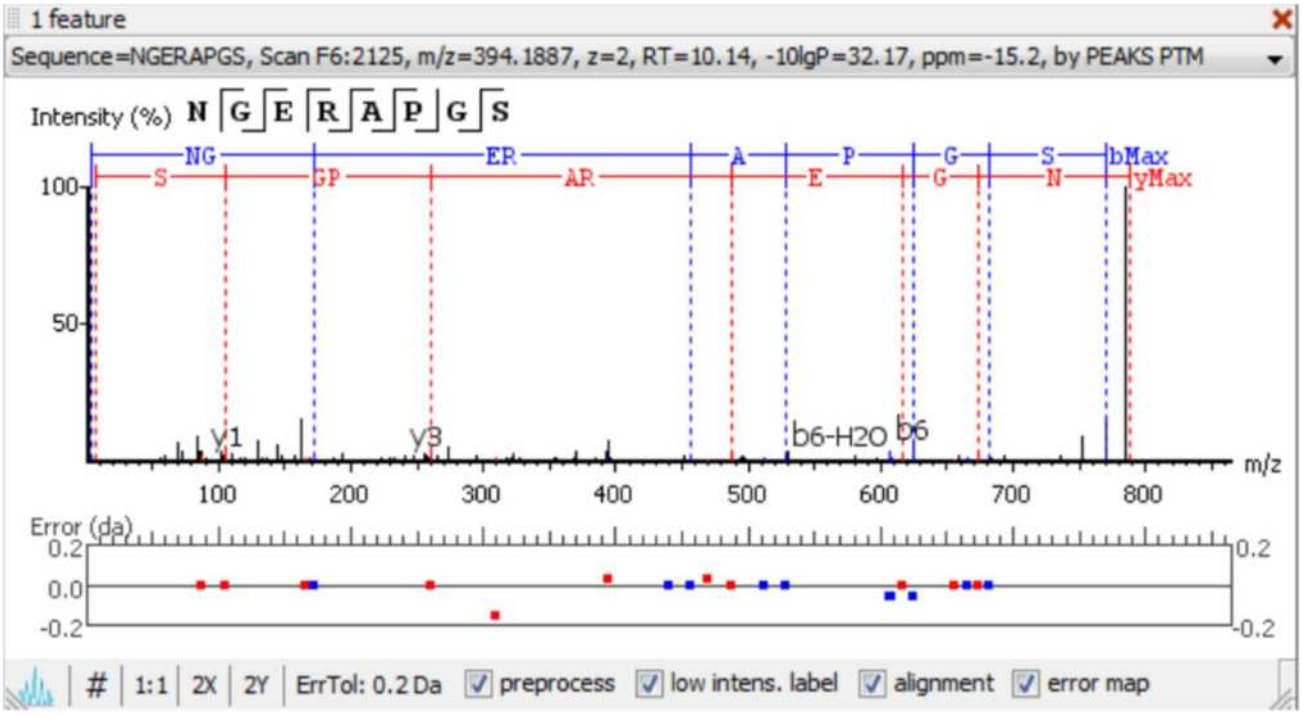

#	b	b-H2O	b-NH3	b (2+)	Seq	y	y-H2O	y-NH3	y (2+)	#
1	115.09	97.03	98.06	58.07	N					8
2	172.07	154.09	155.08	86.49	G	673.33	655.32	656.32	337.17	7
3	301.11	283.10	284.12	151.03	E	616.30	598.29	599.28	308.81	6
4	457.22	439.21	440.19	229.11	R	487.26	469.22	470.24	244.09	5
5	528.25	510.24	511.23	264.63	A	331.01	313.19	313.99	166.09	4
6	625.36	607.36	608.34	313.19	P	260.12	242.11	243.04	130.56	3
7	682.32	664.32	665.30	341.67	G	163.08	145.06	146.04	82.04	2
8					S	106.05	88.04	89.07	53.52	1

Tachykinin-related peptide-12$^{11\text{-}19}$ (TK-12$^{11\text{-}19}$)

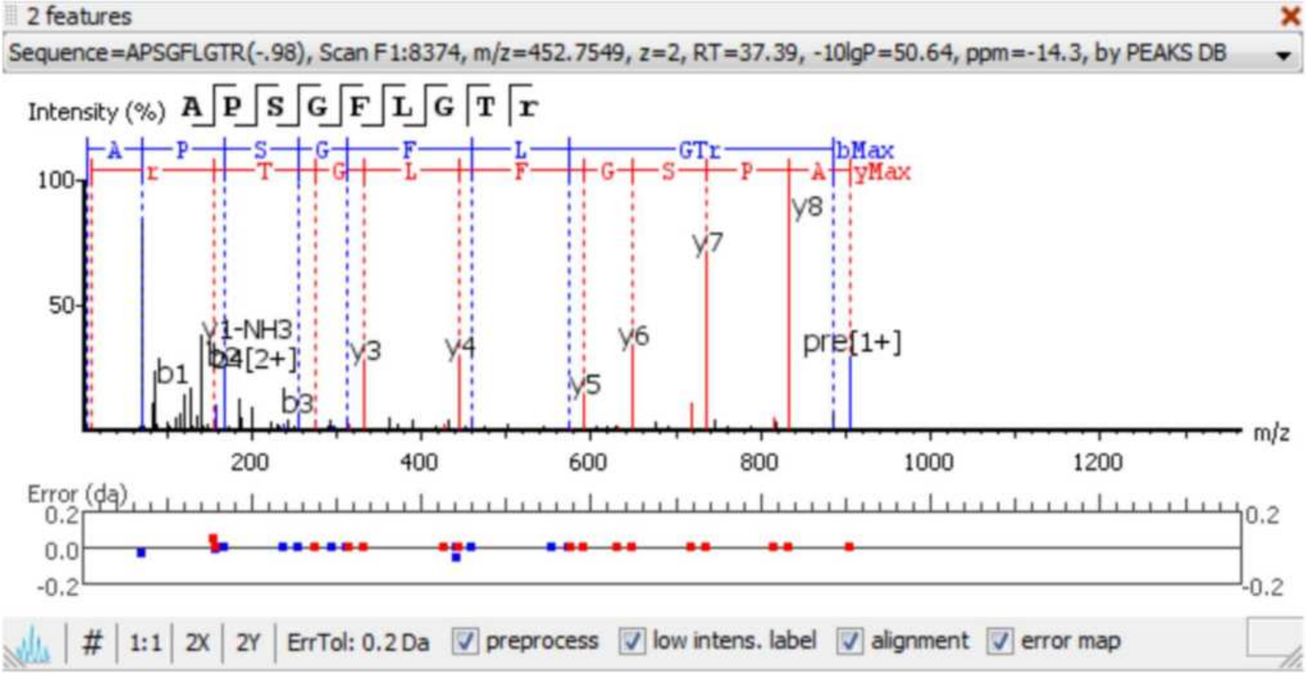

#	b	b-H2O	b-NH3	b (2+)	Seq	y	y-H2O	y-NH3	y (2+)	#
1	72.08	54.03	55.02	36.52	A					9
2	169.10	151.09	152.07	85.08	P	833.46	815.45	816.45	417.23	8
3	256.13	238.12	239.17	128.56	S	736.41	718.40	719.39	368.71	7
4	313.15	295.14	296.12	157.10	G	649.37	631.37	632.35	325.19	6
5	460.22	442.21	443.26	230.61	F	592.36	574.36	575.33	296.68	5
6	573.30	555.29	556.28	287.15	L	445.29	427.28	428.26	223.11	4
7	630.33	612.31	613.30	315.66	G	332.20	314.15	315.18	166.60	3
8	731.37	713.36	714.35	366.19	T	275.18	257.13	258.16	138.07	2
9					R(-.98)	174.13	156.08	157.10	87.57	1

Tachykinin-related peptide-PP-3 (TK-PP-3)_partial

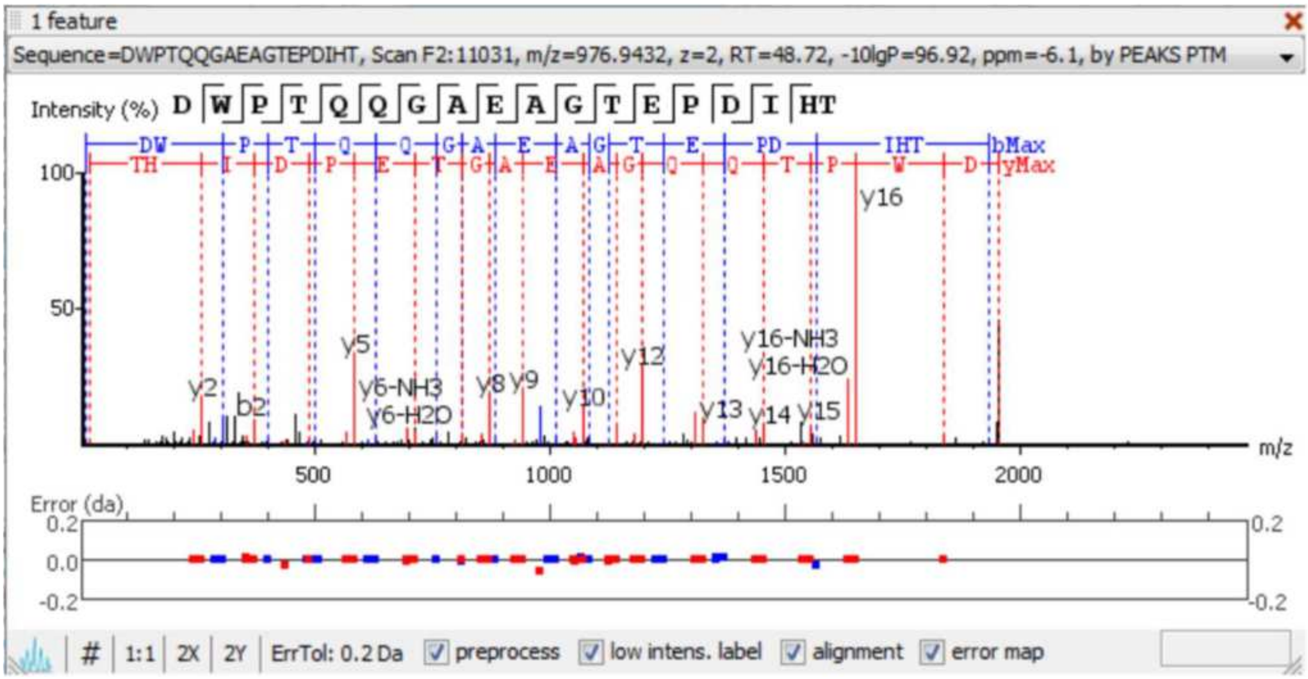

#	b	b-H2O	b-NH3	b (2+)	Seq	y	y-H2O	y-NH3	y (2+)	#
1	116.03	98.02	99.01	58.52	D					18
2	302.11	284.10	285.11	151.56	W	1837.84	1819.82	1820.81	919.42	17
3	399.17	381.16	382.21	200.11	P	1651.77	1633.74	1634.74	826.38	16
4	500.22	482.21	483.21	250.61	T	1554.71	1536.69	1537.69	777.85	15
5	628.27	610.26	611.25	314.64	Q	1453.65	1435.66	1436.64	727.33	14
6	756.33	738.31	739.30	378.67	Q	1325.60	1307.59	1308.57	663.30	13
7	813.37	795.36	796.35	407.18	G	1197.53	1179.53	1180.53	599.25	12
8	884.38	866.38	867.37	442.70	A	1140.51	1122.51	1123.50	570.76	11
9	1013.42	995.54	996.41	507.22	E	1069.48	1051.48	1052.46	535.21	10
10	1084.46	1066.45	1067.44	542.73	A	940.44	922.43	923.42	470.72	9
11	1141.51	1123.50	1124.46	571.24	G	869.40	851.39	852.39	435.24	8
12	1242.55	1224.53	1225.51	621.77	T	812.38	794.37	795.36	406.69	7
13	1371.56	1353.55	1354.55	686.31	E	711.33	693.32	694.32	356.20	6
14	1468.63	1450.62	1451.61	734.82	P	582.29	564.28	565.27	291.64	5
15	1583.66	1565.69	1566.70	792.32	D	485.24	467.23	468.22	243.13	4
16	1696.75	1678.73	1679.72	848.87	I	370.21	352.20	353.16	185.60	3
17	1833.80	1815.79	1816.78	917.47	H	257.12	239.11	240.10	129.06	2
18					T	120.07	102.05	103.04	60.53	1

Supplementary Information S5: Baratin *vs.* NVP-like peptide. **(A)** Multiple sequence alignment of NVP-like precursors identified from the transcriptomes of *R. maderae* (this study), *P. americana*[33] and *C. morosus*[34] shows highly sequential similarities in amino acid sequences (asterisks) suggesting that Baratin is a product of the NVP-like precursor. The previous identified of Baratin[67] is marked in bold. Resulting alignment **(B)** Resulting mass spectrum of a portion of the accessory medullae (AME) at the mass range m/z 950-1250 Da. Ion signals corresponding to NVP-like peptide-2 (NVP-2) and a truncated form NVP-2_{1-9} are marked. Both, the sequence of NVP-2 (yellow) and NVP-2_{1-9} were confirmed by fragmentation experiments (**Supplementary Fig. S4).** Peptide cleavage sites are labelled in red.

```
Rhm NVP-like    ---MMTGVVLVALLVVAVSGLPSTLLEDTKQAAQIAS-----------------------
Pea NVP-like    MAGVAATCALVTLLVAAVVALPNTLLEDTKQVVLESEPIISKVKRAQEVMMFGNQQNRPS
Cam NVP-like    MRRCLEVVLLLGAALAAVGAVPATLMEDTKQAQLHASG----------------------
                         *:   :.** .:* **:*****.   :.

Rhm NVP-like    ------------------------------NSDPKS-----------------------
Pea NVP-like    TENKLYPSAHKRASNKQEKDEELPTALPIAISSTSKTWENGNKLAHHEQHGNGGKTNARG
Cam NVP-like    ----------------------------KVKRAQEVMLFGNQQNRPSEAAYFQGLKRGEG
                                                :

Rhm NVP-like    ------------------------------------------------------------
Pea NVP-like    NINYPGGQRAQYRQELGNGDMADQQEDHGRGKTVSQYEKGYQYGVGKAALDKHVENALLK
Cam NVP-like    DEAGDESATTILPADGFQDAEEDGGRLEEEGKSPAQYEKGYRYGVGKEAMDKHVENALLK


Rhm NVP-like    --------AVNQDRYYGGASERKRNQHLTYQPPSKR----SYRPEIPFVMPPDDLTNSRS
Pea NVP-like    SELYGDPGSVNQYRYYGGSSERKRNQHLSYPPPSKR----SYQPEMPFVLPTDDLTSSRS
Cam NVP-like    SELFGEPSSMNQYRYYGGG-ERRGLQDVGFAPPSKRGGVSSFRPMVPRALP------STP
                        ::** *****. **:  *.: : *****    *::* :* .:*      * .

Rhm NVP-like    RLKRDLELDPEDFLTVLSLWEAEHRAKSE-TPVVDPSWYSYYGMESPDQFQDEEDESGEE
Pea NVP-like    RLKRDLGLDPEDVLTVLSLWEAEHRAKAESNPGLDPSWFTYYGLDTAEPFRDDEDEGEED
Cam NVP-like    RLKRDLELDPEDVLALLSLWEAEHHKQFPYRQEPSP-WSQLNGVEPSEDFETQDEDDQTN
                ****** *****.*::********: :       .* *    *::..: *. ::::.  :

Rhm NVP-like    EEEPSQLEGGWLDGPVVH--PSTSPHRYRLERRGGYYY-----PMQYPMYPAPKRDNSQW
Pea NVP-like    EEDTSPIDGGWLEGPVAH--PSSTPHRYRLERRGGYYYPMLTSPQQYPLYPAQKRDSSQW
Cam NVP-like    SEDDNPNAGGWWDGPVYPSAPSTYPGHFSLEKRVPMYYPAPVPGAPFPLFPAQKREG-QW
                .*: .   *** :***    **: * :: **:*   **        :*::** **:. **

Rhm NVP-like    GGFAKDKRFMVTRKRQVNTARDEAQTLAQLLS-HQYRDPGVPLYRRVVL-
Pea NVP-like    GGFAKDKRFMVSRKRQVTSPRDEVQTLAQLLN-HPYRDPGVPLYRRVVL-
Cam NVP-like    GGFTKDKRFMVSRKRQAP--RDGVHSLAQLLNSTPRRDPGVPVLRRMVL-
                ***:*******:****.   ** .::*****.    ******: **:**
```

A

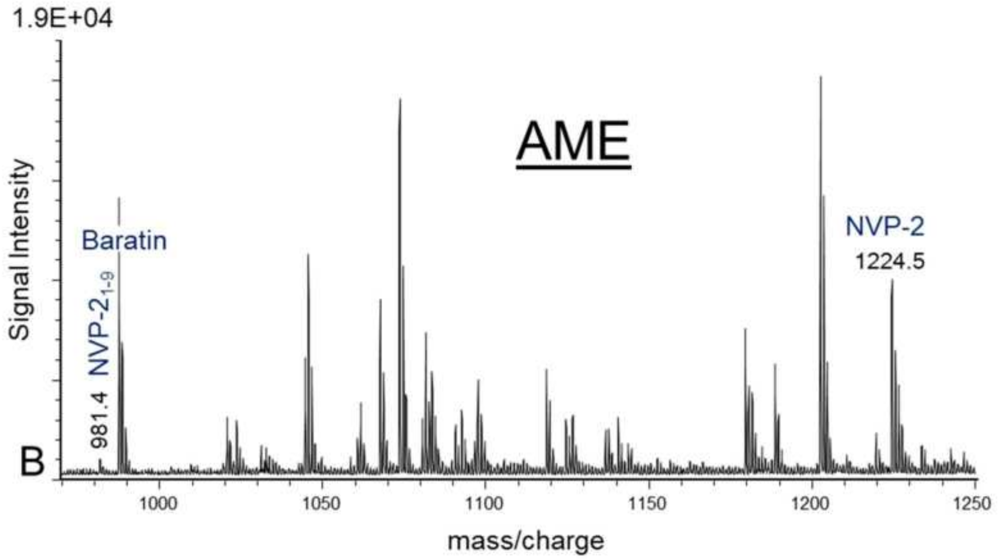

B
